# Supplementary material for: Atroposelective synthesis of biaxial bridged eight-membered terphenyls via a Co/SPDO-catalyzed aerobic oxidative coupling/desymmetrization of phenols
Source: Nat Commun. 2024 May 30;15:4591. doi: 10.1038/s41467-024-48858-1 (PMC11139896; doi:10.1038/s41467-024-48858-1)
Supplement: Supplementary file 1 — Supplementary Information [file 41467_2024_48858_MOESM1_ESM.pdf]

Supplementary Information

Atroposelective synthesis of biaxial bridged eight-membered terphenyls via a Co/SPDO-catalyzed aerobic oxidative coupling/desymmetrization of phenols

Shuang-Hu Wang,[1] Shi-Qiang Wei,[1] Ye Zhang,[1] Xiao-Ming Zhang,[2] Shu-Yu Zhang,[1] Kun-Long Dai,[1] Yong-Qiang Tu,[1,2*] Ka Lu[2] and Tong-Mei Ding[1]

1, School of Chemistry and Chemical Engineering, Frontier Scientific Center of Transformative Molecules, Shanghai key Laboratory of Chiral Drugs and Engineering, Shanghai Jiao Tong University, Shanghai Minhang 200240, China

2, State Key Laboratory of Applied Organic Chemistry & College of Chemistry and Chemical Engineering, Lanzhou University, Lanzhou 730000, China

E-mail: tuyq@sjtu.edu.cn, tuyq@lzu.edu.cn

Table of Contents

Table of Contents 1

Supplementary Methods 2

1. General information 2

2. Preparation of 1a-1ap: 3

3. Preparation of 1aq-1as, 1ba: 26

4. Preparation of 1at: 29

5. Preparation of 1au, 1av, 1aw, 1ax: 30

6. Preparation of 1az: 34

7. Preparation of racemic samples: 37

8. Preparation of chiral 2: 37

9. Preparation of 3a: 128

10. Mechanistic studies: 130

11. X-ray crystallographic data for 2ad and 3a 133

12. Unsuccessful substrates 137

13. Gram scale reaction and transformation 137

14. Stability testing 138

15. Copies of NMR spectra 140

16. Supplementary References 245

Supplementary Methods

1. General information

All reactions were performed using oven-dried or flame-dried glassware equipped with a magnetic stir bar before used. All reagents were purchased from commercial suppliers and used without further purification. All solvents were purified by standard operating method. Toluene, tetrahydrofuran (THF), were distilled from sodium; dichloromethane (DCM) were distilled from calcium hydride; acetone and ethanol are purchased from commercial suppliers and used without further purification . Thin-layer chromatography was performed with EMD silica gel 60 F_{254} plates eluting with solvents indicated, visualized by a 254 nm UV lamp and stained with phosphomolybdic acid (PMA). **^{1}H NMR**, **^{13}C NMR** and **^{19}F NMR** spectra were obtained on Bruker AM-400, Bruker AM-500. Chemical shifts (δ) were quoted in ppm relative to tetramethylsilane or residual solvent as internal standard $CDCl_3$: 7.26 ppm for ^{1}H NMR, 77.0 ppm for ^{13}C NMR, D_4-CD_3OD: 3.31 ppm for ^{1}H NMR, 49.00 ppm for ^{13}C NMR; D_6-Acetone: 2.05 ppm for ^{1}H NMR, 206.68 ppm and 29.92 ppm for ^{13}C NMR; multiplicities are as indicated: s = singlet, d = doublet, t = triplet, q = quartet, m = multiplet. **High-resolution mass spectral analysis (HRMS) data** was measured on a Bruker impact Ⅱ (Q-TOF) mass spectrum by means of the ESI technique. **Crystallographic data** were obtained from a Bruker D8 VENTURE diffractometer. Optical rotations were detected on RUDOLPH A21202-J APTV/GW. The enantiomeric excesses (ee) of the products were determined by high performance liquid chromatography (HPLC) analysis or UPC^2.

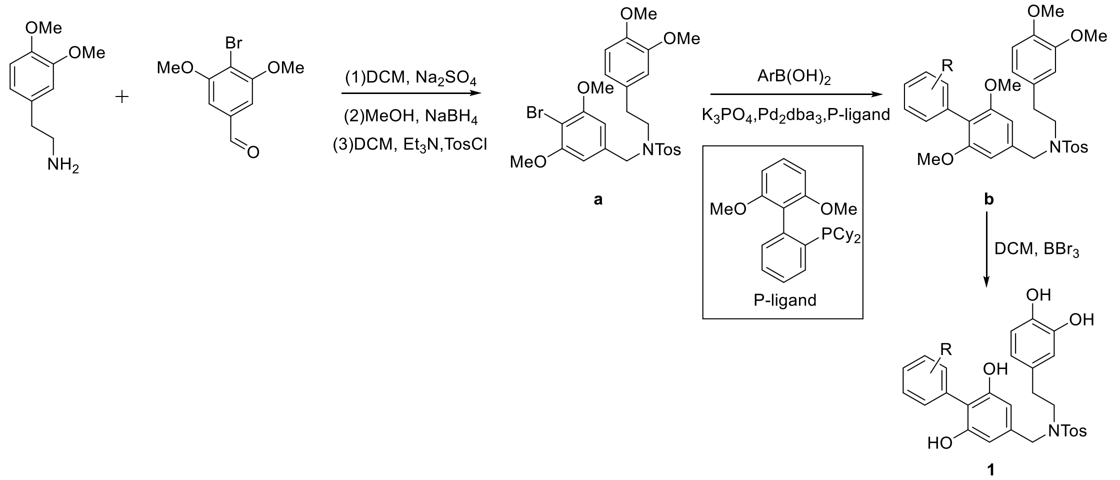

Supplementary Fig. 1: General procedure for preparation of **1**

2. Preparation of 1a-1ap:

General procedure for preparation of **1** (**Supplementary Fig. 1**): Under argon atmosphere, 3, 4-dimethoxyphenethylamine (21.9 g, 1.0 equiv) was dissolved in dry DCM (200 mL) at room temperature, Na_2SO_4 (46 g, 5 equiv) and 4-bromo-3,5-dimethoxybenzaldehyde(27 g, 1.0 equiv) was added subsequently. The resulted solution was stirred at room temperature for 4 hours, after which the system was filtered to remove sodium sulfate and concentrate under reduced pressure, then cooled to 0 ℃, the residue dissolved in MeOH (200 mL), $NaBH_4$ (4.2 g, 1.0 equiv) was added portionwise and the reaction mixture was stirred for additional 4 hours at room temperature. The reaction was quenched by addition of a saturated solution of H_2O (10 mL) then concentrated under vacuum and the residue was extracted with EtOAc (3 × 150 mL). The combined organic layer was wash with brine, dried over Na_2SO_4 and concentrated under vacuum.[1] Without purification, the obtained residue was dissolved in anhydrous DCM (250 mL) in a 500 mL dried round bottom flask under argon atmosphere. Et_3N (12 mL, 2.4 eq) and TosCl (8.4 g, 1.2 eq) was added successively under argon atmosphere at 0 °C. The reaction mixture was stirred for 45 mins with natural warming. Then the reaction was quenched by addition of a saturated solution of $NaHCO_3$ (100 mL) and the reaction mixture was extracted with DCM (3 × 150 mL). The combined organic layer was washed with brine, dried over Na_2SO_4 and concentrated under vacuum, the residue was purified by a flash column chromatography on silica gel (petroleum ether/ethyl acetate = 20:1 to 1:1) to give product **a** (65 g, 96%) as colorless solid.

Under argon atmosphere, **a** (1 equiv), $Pd_2(dba)_3$ (0.015 equiv), ligand (0.06 equiv), K_3PO_4 (3 equiv) and arylboronic acid (2 equiv) were mixed together in dry toluene (50 mL) , the system replaced argon for three times and stirred at 100 ℃ for 48 hours,[2] subsequently filtered through celite and concentrated under vacuum to give crude product **b** without purification.

Under argon atmosphere, **b** (1.0 eq) was dissolved in dry DCM (50 mL) and BBr_3 (8.0 eq) was added successively under argon atmosphere at 0 °C, stirred for 12 hours. The reaction was quenched by addition of MeOH (50 mL) then concentrated under vacuum, and the residue was extracted with EtOAc (3 × 100 mL). The combined organic layer was wash with brine, dried over Na_2SO_4 and concentrated under vacuum, and chromatographied on silica gel (DCM: acetone = 20:1 as eluent) to give **1**.

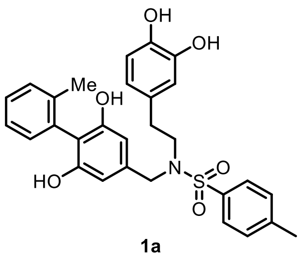

1a

The **1a** was obtained in 90% (6g), colorless foam solid (silica gel flash chromatography: DCM: Acetone = 20:1). R_f = 0.40 (DCM: Acetone = 8:1).

^{1}H NMR (500 MHz, $CDCl_3$): δ 7.62 (d, *J* = 8.0 Hz, 2H), 7.22 – 7.14 (m, 5H), 7.10 (d, *J* = 7.0 Hz, 1H), 6.60 (d, *J* = 8.0 Hz, 1H), 6.44 (s, 3H), 6.37 (s, 1H), 6.27 (d, *J* = 8.0 Hz, 1H), 6.19 (s, 1H), 5.40 (s, 2H), 4.12 (s, 2H), 3.19 (d, *J* = 7.0 Hz, 2H), 2.44 (s, 2H), 2.32 (s, 3H), 2.02 (s, 3H).

^{13}C NMR (125 MHz, $CDCl_3$): δ 153.5, 143.55, 142.2, 139.1, 137.7, 136.2, 131.2, 130.9, 129.9, 129.8 129.2, 127.0, 126.8, 120.9, 115.8, 115.4, 114.4, 107.5, 51.8, 49.7, 34.2, 21.4, 19.3.

HRMS (ESI) *m/z* calcd. for $C_{29}H_{29}NO_6S$ $(M+Na)^+$: 542.1608, found: 542.1603

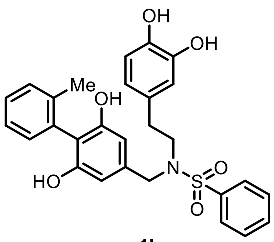

1b

The **1b** was obtained in 70% (1.45 g), colorless foam solid (silica gel flash chromatography: DCM: Acetone = 20:1). R_f = 0.40 (DCM: Acetone = 8:1).

^{1}H NMR (500 MHz, CD_3OD): δ 7.82 (d, *J* = 7.0 Hz, 2H), 7.61 – 7.57 (m, 1H), 7.53 (m, 2H), 7.26 – 7.23 (m, 1H), 7.21 – 7.16 (m, 2H), 7.16 – 7.13 (m, 1H), 6.63 (d, *J* = 8.0 Hz, 1H), 6.50 (s, 2H), 6.49 (d, *J* = 2.0 Hz, 1H), 6.30 (dd, *J* = 8.0, 2.0 Hz, 1H), 4.86 (s, 1H), 4.24 (s, 2H), 3.30 – 3.24 (m, 2H), 2.53 – 2.46 (m, 2H), 2.13 (s, 3H).

^{13}C NMR (125 MHz, CD_3OD): δ 156.5, 146.1, 144.7, 141.1, 139.0, 138.3, 135.1, 133.8, 132.1, 131.4, 130.6, 130.3, 128.3, 128.0, 126.4, 121.0, 117.0, 116.7, 116.4, 108.2, 53.1, 51.0, 35.5, 20.0.

HRMS (ESI) *m/z* calcd. for $C_{28}H_{27}NO_6S$ $(M+Na)^+$: 528.1451, found: 528.1458

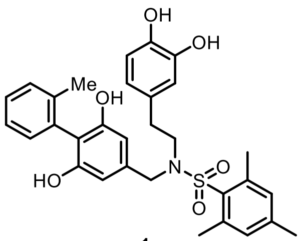

1c

The **1c** was obtained in 87% (1.1 g), colorless foam solid (silica gel flash chromatography: DCM: Acetone = 20:1). R_f = 0.40 (DCM: Acetone = 8:1).

^{1}H NMR (500 MHz, Acetone-d_6): δ 7.84 (s, 2H), 7.71 (s, 2H), 7.28 – 7.10 (m, 4H), 7.04 (s, 2H), 6.64 (d, J = 8.0 Hz, 1H), 6.53 (s, 2H), 6.48 (d, J = 2.0 Hz, 1H), 6.30 (dd, J = 8.0, 2.0 Hz, 1H), 4.41 (s, 2H), 3.35 – 3.20 (m, 2H), 2.61 (d, J = 6.0 Hz, 6H), 2.58 – 2.50 (m, 2H), 2.31 (s, 3H), 2.14 (s, 3H).

^{13}C NMR (125 MHz, Acetone-d_6): δ 156.0, 145.5, 144.0, 142.9, 140.3, 138.5, 137.8, 134.4, 134.4, 132.5, 131.8, 130.8, 130.1, 127.8, 125.9, 120.2, 115.9, 115.9, 115.7, 107.7, 50.3, 47.8, 33.3, 30.3, 22.8, 20.6, 19.7.

HRMS (ESI) *m/z* calcd. for $C_{31}H_{33}NO_6S$ $(M+Na)^+$: 570.1921, found: 570.1919

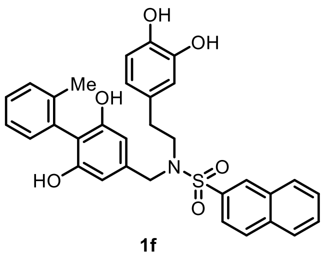

1f

The **1f** was obtained in 75% (1.5g), colorless foam solid (silica gel flash chromatography: DCM: Acetone = 20:1). R_f = 0.40 (DCM: Acetone = 8:1).

^{1}H NMR (500 MHz, CD_3OD): δ 8.43 (d, J = 11.5 Hz, 1H), 7.99 (d, J = 8.5 Hz, 2H), 7.92 (d, J = 8.0 Hz, 1H), 7.81 – 7.77 (m, 1H), 7.69 – 7.52 (m, 3H), 7.24 (d, J = 7.0 Hz, 1H), 7.22 – 7.16 (m, 2H), 7.14 – 7.10 (m, 1H), 6.61 (t, J = 9.0 Hz, 1H), 6.55 – 6.50 (m, 3H), 6.29 (dd, J = 8.0, 2.0 Hz, 1H), 4.32 (s, 2H), 3.39 – 3.33 (m, 2H), 2.56 – 2.50 (m, 2H), 2.12 (d, J = 5.5 Hz, 3H).

^{13}C NMR (125 MHz, CD_3OD): δ 156.6, 146.1, 144.7, 139.0, 138.4, 138.2, 136.1, 135.1, 133.5, 132.1, 131.4, 130.6, 130.6, 130.2, 129.8, 129.2, 128.9, 128.9, 128.6, 128.3, 126.4, 123.4, 121.0, 117.1, 116.7, 116.4, 108.2, 53.1, 51.1, 35.6, 20.0.

HRMS (ESI) *m/z* calcd. for $C_{32}H_{29}NO_6S$ $(M+Na)^+$: 578.1608, found: 578.1598

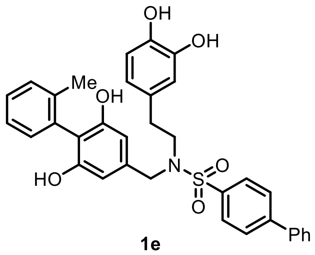

1e

The **1e** was obtained in 92% (2.4 g), colorless foam solid (silica gel flash chromatography: DCM: Acetone = 20:1). R_f = 0.40 (DCM: Acetone = 8:1).

^{1}H NMR (500 MHz, CD_3OD): δ 7.87 (d, *J* = 8.5 Hz, 2H), 7.77 (d, *J* = 8.5 Hz, 2H), 7.65 (d, *J* = 7.5 Hz, 2H), 7.45 (t, *J* = 7.5 Hz, 2H), 7.38 (t, *J* = 7.0 Hz, 1H), 7.25 – 7.09 (m, 4H), 6.63 (d, *J* = 8.0 Hz, 1H), 6.52 – 6.48 (m, 3H), 6.33 (dd, *J* = 8.0, 2.0 Hz, 1H), 4.27 (s, 2H), 3.36 – 3.28 (m, 2H), 2.56 (dd, *J* = 17.0, 9.0 Hz, 2H), 2.11 (s, 3H).

^{13}C NMR (125 MHz, CD_3OD): δ 156.6, 146.7, 146.2, 144.8, 140.5, 139.8, 139.1, 138.3, 135.2, 132.2, 131.5, 130.7, 130.1, 129.5, 128.8, 128.7, 128.3, 126.4, 121.0, 117.1, 116.8, 116.4, 108.3, 53.2, 51.2, 35.8, 20.1.

HRMS (ESI) *m/z* calcd. for $C_{34}H_{31}NO_6S$ $(M+Na)^+$: 604.1764, found: 604.1760

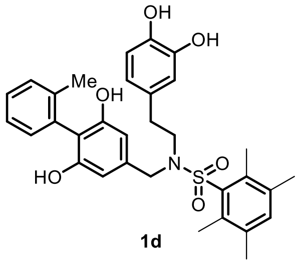

1d

The **1d** was obtained in 82% (0.9 g), colorless foam solid (silica gel flash chromatography: DCM: Acetone = 20:1). R_f = 0.40 (DCM: Acetone = 8:1).

^{1}H NMR (500 MHz, CD_3OD): δ 7.26 – 7.16 (m, 5H), 7.14 – 7.10 (m, 1H), 6.54 (d, *J* = 8.0 Hz, 1H), 6.44 (s, 2H), 6.36 (d, *J* = 2.0 Hz, 1H), 6.23 (dd, *J* = 8.0, 2.0 Hz, 1H), 4.36 (s, 2H), 3.30 – 3.25 (m, 2H), 2.57 – 2.52 (m, *J* = 12.5, 5.0 Hz, 2H), 2.49 (s, 6H), 2.26 (s, 6H), 2.11 (s, 3H).

^{13}C NMR (125 MHz, CD_3OD): δ 156.5, 146.0, 144.6, 139.1, 137.9, 137.8, 137.4, 137.3, 137.2, 135.1, 132.1, 131.4, 130.7, 128.3, 126.4, 120.8, 117.1, 116.5, 116.2, 108.5, 50.8, 48.4, 33.9, 21.2, 20.0, 18.1.

HRMS (ESI) *m/z* calcd. for $C_{32}H_{35}NO_6S$ $(M+Na)^+$: 584.2077, found: 584.2070

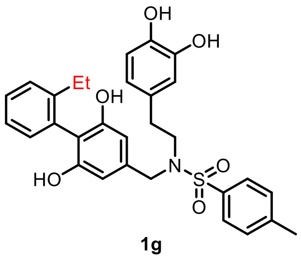

1g

The **1g** was obtained in 91% (0.9 g), colorless foam solid (silica gel flash chromatography: DCM: Acetone = 20:1). R_f = 0.40 (DCM: Acetone = 8:1).

^{1}H NMR (500 MHz, Acetone-d_6): δ 7.81 – 7.67 (m, 6H), 7.43 (d, *J* = 8.0 Hz, 2H), 7.31 – 7.23 (m, 2H), 7.19 (td, *J* = 7.0, 1.5 Hz, 1H), 7.13 (dd, *J* = 7.5, 1.0 Hz, 1H), 6.68 (dd, *J* = 8.0, 4.5 Hz, 1H), 6.58 (s, 2H), 6.56 (d, *J* = 2.0 Hz, 1H), 6.37 (dd, *J* = 8.0, 2.0 Hz, 1H), 4.29 (s, 2H), 3.31 – 3.25 (m, 2H), 2.98 (s, 3H), 2.56 – 2.47 (m, 4H), 2.44 (s, 3H), 1.05 (t, *J* = 7.5 Hz, 3H).

^{13}C NMR (125 MHz, Acetone-d_6): δ 156.2, 145.6, 144.5, 144.1, 143.8, 138.4, 138.2, 133.9, 132.1, 131.0, 130.3, 128.7, 128.1, 127.7, 126.1, 120.4, 116.2, 115.9, 115.8, 107.6, 52.5, 50.8, 35.1, 27.0, 21.2, 15.0.

HRMS (ESI) *m/z* calcd. for $C_{30}H_{31}NO_6S$ $(M+Na)^+$: 556.1764, found: 556.1752

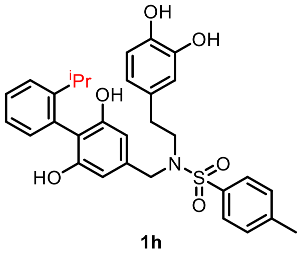

1h

The **1h** was obtained in 87% (0.87g), colorless foam solid (silica gel flash chromatography: DCM: Acetone = 20:1). R_f = 0.40 (DCM: Acetone = 8:1).

^{1}H NMR (500 MHz, Acetone-d_6): δ 7.82 – 7.66 (m, 6H), 7.43 (d, *J* = 8.0 Hz, 2H), 7.37 (d, *J* = 8.0 Hz, 1H), 7.33 – 7.27 (m, 1H), 7.18 (td, *J* = 7.5, 1.0 Hz, 1H), 7.11 (dd, *J* = 7.5, 1.0 Hz, 1H), 6.69 (t, *J* = 7.0 Hz, 1H), 6.59 – 6.56 (m, 3H), 6.37 (dd, *J* = 8.0, 2.0 Hz, 1H), 4.29 (s, 2H), 3.31 – 3.25 (m, 2H), 3.05 (s, 2H), 2.90 (dt, *J* = 14.0, 7.0 Hz, 1H), 2.54 (dd, *J* = 10.0, 7.0 Hz, 2H), 2.43 (s, 3H), 1.13 (d, *J* = 7.0 Hz, 6H).

^{13}**C NMR** (125 MHz, Acetone-d_6): δ 156.3, 149.0, 145.6, 144.0, 143.7, 138.3, 138.1, 133.2, 131.8, 130.9, 130.3, 128.2, 127.6, 125.9, 125.5, 120.4, 116.1, 115.9, 115.7, 107.5, 52.4, 50.7, 35.0, 30.8, 23.8, 21.1.

HRMS (ESI) *m/z* calcd. for $C_{31}H_{33}NO_6S$ $(M+Na)^+$: 570.1921, found: 570.1910

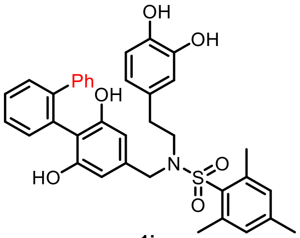

1i

The **1i** was obtained in 46% (0.56g), colorless foam solid (silica gel flash chromatography: DCM: Acetone = 20:1). R_f = 0.40 (DCM: Acetone = 8:1).

^{1}**H NMR** (500 MHz, CD_3OD): δ 7.36 (m, 3H), 7.26 (m, 1H), 7.20 (m, 2H), 7.00 (m, 5H), 6.59 (d, *J* = 8.0 Hz, 1H), 6.36 (d, *J* = 2.0 Hz, 1H), 6.25 – 6.20 (m, 3H), 4.22 (s, 2H), 3.14 – 3.09 (m, 2H), 2.55 (s, 6H), 2.45 – 2.40 (m, 2H), 2.31 (s, 3H).

^{13}**C NMR** (125 MHz, CD_3OD): δ 156.8, 146.2, 144.8, 144.3, 143.4, 141.2, 137.8, 134.2, 133.1, 133.0, 131.4, 130.7, 129.7, 128.5, 128.3, 127.9, 127.3, 120.8, 117.6, 116.6, 116.3, 108.2, 51.0, 34.1, 29.5, 23.2, 21.1.

HRMS (ESI) *m/z* calcd. for $C_{36}H_{35}NO_6S$ $(M+Na)^+$: 632.2077, found: 632.2073

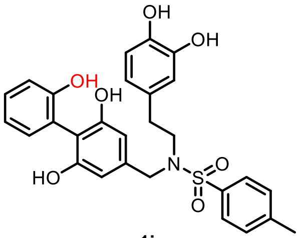

1j

The **1j** was obtained in 53% (0.4g), colorless foam solid (silica gel flash chromatography: DCM: Acetone = 20:1). R_f = 0.40 (DCM: Acetone = 8:1).

^{1}**H NMR** (500 MHz, Acetone-d_6): δ 7.78 (d, *J* = 8.0 Hz, 2H), 7.44 (dd, *J* = 14.0, 6.5 Hz, 2H), 7.25 (dd, *J* = 11.5, 6.0 Hz, 1H), 7.22 – 7.16 (m, 1H), 6.96 (d, *J* = 8.0 Hz, 1H), 6.89 (td, *J* = 7.5, 1.0 Hz, 1H), 6.70 – 6.65 (m, 1H), 6.58 (d, *J* = 6.0 Hz, 2H), 6.55 (d, *J* = 2.0 Hz, 1H), 6.38 (dd, *J*

= 8.0, 2.0 Hz, 1H), 4.30 (s, 2H), 3.30 (dd, J = 9.5, 7.0 Hz, 2H), 2.55 (dd, J = 9.5, 7.0 Hz, 2H), 2.43 (s, 3H).

^{13}C NMR (125 MHz, Acetone-d_6): δ 156.2, 155.3, 145.3, 143.9, 143.6, 138.5, 138.1, 133.3, 130.7, 130.1, 129.0, 127.5, 121.3, 120.3, 119.9, 116.6, 116.0 115.6, 112.6, 107.8, 51.8, 50.3, 34.4, 20.9.

HRMS (ESI) *m/z* calcd. for $C_{28}H_{27}NO_7S$ $(M+Na)^+$: 544.1400, found: 544.1399

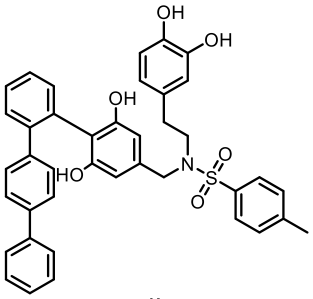

1k

The **1k** was obtained in 95% (1.05 g), colorless foam solid (silica gel flash chromatography: DCM: Acetone = 20:1). R_f = 0.40 (DCM: Acetone = 8:1).

^{1}H NMR (500 MHz, Acetone-d_6): δ 7.93 (s, 1H), 7.79 (s, 1H), 7.74 (d, J = 8.0 Hz, 2H), 7.42 – 7.32 (m, 14H), 6.70 (d, J = 8.0 Hz, 1H), 6.61 (d, J = 2.0 Hz, 1H), 6.46 (d, J = 4.0 Hz, 2H), 6.41 (dd, J = 8.0, 2.0 Hz, 1H), 4.19 (s, 2H), 3.23 – 3.15 (m, 2H), 3.10 (d, J = 14.0 Hz, 1H), 2.47 – 2.41 (m, 2H), 2.39 (s, 3H).

^{13}C NMR (125 MHz, Acetone-d_6): δ 156.1, 145.5, 144.0, 143.6, 142.7, 141.7, 140.7, 139.0, 138.4, 137.9, 133.3, 132.7, 130.9, 130.2, 130.0, 129.6, 129.2, 127.9, 127.4, 127.4, 126.9, 126.1, 120.4, 116.1, 115.8, 107.4, 52.5, 51.0, 35.1, 21.0.

HRMS (ESI) *m/z* calcd. for $C_{40}H_{35}NO_6S$ $(M+Na)^+$: 680.2077, found: 680.2065

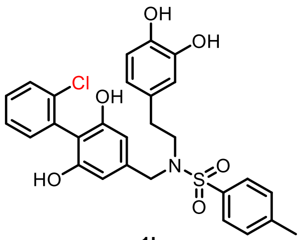

1l

The **1l** was obtained in 69% (0.63g), colorless foam solid (silica gel flash chromatography: DCM: Acetone = 20:1). R_f = 0.40 (DCM: Acetone = 8:1).

¹H NMR (500 MHz, Acetone-d_6): δ 8.18 (s, 1H), 7.78 (d, J = 8.0 Hz, 2H), 7.74(s, 1H), 7.46 (dd, J = 6.0, 2.0 Hz, 1H), 7.42 (d, J = 8.0 Hz, 2H), 7.35 – 7.29 (m, 3H), 6.68 (d, J = 8.0 Hz, 1H), 6.58 (s, 2H), 6.57 (d, J = 2.0 Hz, 1H), 6.38 (dd, J = 8.0, 2.0 Hz, 1H), 4.29 (s, 2H), 3.29 (dd, J = 9.5, 7.0 Hz, 2H), 3.17 (d, J = 13.5 Hz, 1H), 2.54 (dd, J = 9.5, 7.0 Hz, 2H), 2.43 (s, 3H).

¹³C NMR (125 MHz, Acetone-d_6): δ 156.2, 145.4, 143.9, 143.6, 139.0, 138.0, 135.4, 134.5, 133.5, 130.8, 130.2, 129.4, 128.9, 127.5, 126.8, 120.3, 116.0, 115.6, 107.3, 52.1, 50.6, 34.7, 21.0.

HRMS (ESI) *m/z* calcd. for $C_{28}H_{26}NO_6SCl$ $(M+Na)^+$: 562.1062, found: 562.1053

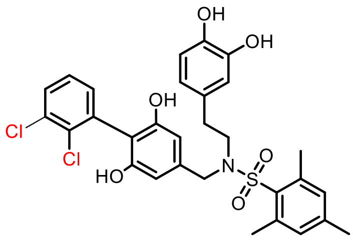

1m

The **1m** was obtained in 37% (0.4g), colorless foam solid (silica gel flash chromatography: DCM: Acetone = 20:1). R_f = 0.40 (DCM: Acetone = 8:1).

¹H NMR (500 MHz, Acetone-d_6): δ 7.52 (dd, J = 8.0, 1.5 Hz, 1H), 7.35 (t, J = 8.0 Hz, 1H), 7.29 (dd, J = 7.5, 1.5 Hz, 1H), 7.06 (s, 2H), 6.64 (d, J = 8.0 Hz, 1H), 6.55 (s, 2H), 6.47 (d, J = 2.0 Hz, 1H), 6.30 (dd, J = 8.0, 2.0 Hz, 1H), 4.43 (s, 2H), 3.34 – 3.17 (m, 2H), 2.99 (d, J = 15.0 Hz, 4H), 2.65 – 2.60 (m, 6H), 2.57 – 2.48 (m, 2H), 2.33 (s, 3H).

¹³C NMR (125 MHz, Acetone-d_6): δ 156.2, 145.5, 144.0, 143.0, 140.3, 139.0, 137.5, 134.4, 133.7, 132.7, 132.5, 132.1, 130.8, 129.6, 127.8, 120.2, 115.9, 115.7, 107.5, 50.3, 47.8, 33.3, 22.8, 20.7.

HRMS (ESI) *m/z* calcd. for $C_{30}H_{29}NO_6SCl_2$ $(M+Na)^+$: 624.0985, found: 624.0983

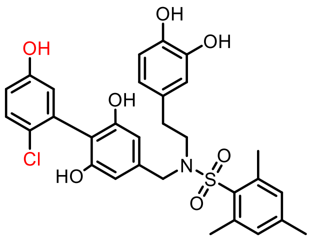

1n

The **1n** was obtained in 43% (0.5g), colorless foam solid (silica gel flash chromatography: DCM: Acetone = 20:1). R_f = 0.40 (DCM: Acetone = 8:1).

^{1}H NMR (500 MHz, Acetone-d_6): δ 8.77 (s, 1H), 8.02 (s, 2H), 7.74 (d, J = 29.0 Hz, 2H), 7.12 (d, J = 8.5 Hz, 1H), 7.04 (s, 2H), 6.96 (s, 3H), 6.83 (d, J = 8.5 Hz, 1H), 6.65 (d, J = 8.0 Hz, 1H), 6.52 – 6.47 (m, J = 13.0 Hz, 3H), 6.31 (d, J = 8.0 Hz, 1H), 5.60 (s, 1H), 4.40 (s, 2H), 3.39 (s, 2H), 3.29 – 3.23 (m, 2H), 2.62 (s, 6H), 2.58 – 2.51 (m, 2H), 2.31 (s, 3H).

^{13}C NMR (125 MHz, Acetone-d_6): δ 157.6, 156.2, 156.1, 145.1, 143.6, 142.6, 139.9, 137.8, 135.5, 133.9, 133.8, 132.1, 130.4, 124.4, 119.9, 116.1, 115.6, 115.3, 114.2, 113.7, 107.1, 107.0, 49.9, 47.3, 32.9, 29.9, 22.4, 20.3.

HRMS (ESI) m/z calcd. for $C_{30}H_{30}NO_7S$ Cl$(M+Na)^+$: 606.1324, found: 606.1320

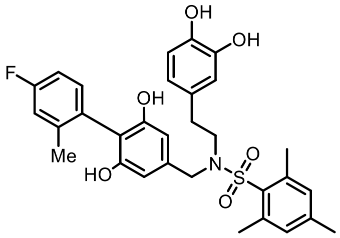

1o

The **1o** was obtained in 58% (0.4 g), colorless foam solid (silica gel flash chromatography: DCM: Acetone = 20:1). R_f = 0.40 (DCM: Acetone = 8:1).

^{1}H NMR (500 MHz, Acetone-d_6): δ 7.88 (m, 3H), 7.16 (dd, J = 8.5, 6.0 Hz, 1H), 7.04 (s, 2H), 7.01 (d, J = 2.5 Hz, 1H), 6.94 (td, J = 8.5, 3.0 Hz, 1H), 6.64 (d, J = 8.0 Hz, 1H), 6.53 (s, 2H), 6.46 (d, J = 2.0 Hz, 1H), 6.30 (dd, J = 8.0, 2.0 Hz, 1H), 4.42 (s, 2H), 3.28 – 3.24 (m, 2H), 3.05 (s, 1H), 2.61 (s, 6H), 2.56 – 2.52 (m, 2H), 2.31 (s, 3H), 2.15 (s, 3H).

^{13}C NMR (125 MHz, Acetone-d_6): δ162.9 (d, J = 242.3 Hz), 156.2, 145.5, 144.0, 143.0, 141.3, 141.2, 140.3, 138.1, 134.4, 133.8 (d, J = 8.4 Hz), 132.5, 130.8, 130.7, 130.6, 120.2, 116.7 (d, J = 21.1 Hz), 116.2 (d, J = 32.9 Hz), 114.8, 112.7 (d, J = 21.0 Hz), 107.7, 50.3, 47.8, 33.3, 22.8, 20.6, 19.8.

^{19}F NMR (470 MHz, CD_3OD): δ -118.8.

HRMS (ESI) m/z calcd. for $C_{31}H_{32}NO_6SF$ $(M+Na)^+$: 588.1827, found: 588.1823

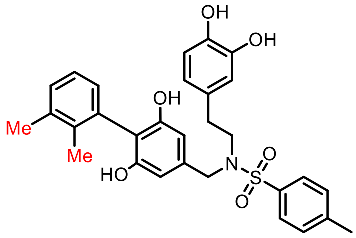

1p

The **1p** was obtained in 78% (0.7g), colorless foam solid (silica gel flash chromatography: DCM: Acetone = 20:1). R_f = 0.40 (DCM: Acetone = 8:1).

^{1}H NMR (500 MHz, Acetone-d_6): δ 7.78 (d, J = 8.0 Hz, 2H), 7.76 – 7.61 (m, 2H), 7.42 (d, J = 8.0 Hz, 2H), 7.12 – 7.06 (m, 2H), 7.01 (m, 1H), 6.70 – 6.66 (m, 1H), 6.58 – 6.55 (m, 3H), 6.38 (dd, J = 8.0, 2.0 Hz, 1H), 4.29 (s, 2H), 3.29 (dd, J = 9.5, 7.0 Hz, 2H), 2.55 (dd, J = 9.5, 7.0 Hz, 2H), 2.43 (s, 3H), 2.29 (s, 3H), 2.05 (s, 3H).

^{13}C NMR (125 MHz, Acetone-d_6): δ 156.0, 145.5, 144.0, 143.7, 138.2, 138.2, 136.9, 136.9, 134.1, 130.9, 130.2, 129.5, 129.3, 127.6, 125.6, 120.4, 116.4, 116.1, 115.7, 107.5, 52.3, 50.6, 34.9, 21.1, 20.3, 16.2.

HRMS (ESI) *m/z* calcd. for $C_{30}H_{31}NO_6S$ $(M+Na)^+$: 556.1764, found: 556.1762

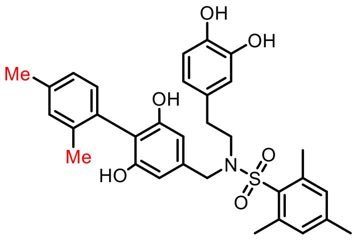

1q

The **1q** was obtained in 73% (0.8g), colorless foam solid (silica gel flash chromatography: DCM: Acetone = 20:1). R_f = 0.40 (DCM: Acetone = 8:1).

^{1}H NMR (500 MHz, Acetone-d_6): δ 7.73 (s, 3H), 7.08 – 7.01 (m, 4H), 6.99 (s, 1H), 6.65 (d, J = 8.0 Hz, 1H), 6.55 – 6.46 (m, 3H), 6.31 (dd, J = 8.0, 2.0 Hz, 1H), 4.40 (s, 2H), 3.31 – 3.23 (m, 2H), 2.62 (s, 6H), 2.58 – 2.50 (m, 2H), 2.30 (d, J = 2.0 Hz, 6H), 2.10 (s, 3H).

^{13}C NMR (125 MHz, Acetone-d_6): δ 155.6, 144.9, 143.5, 142.5, 139.8, 137.8, 137.2, 136.6, 133.8, 131.9, 131.2, 130.5, 130.3, 126.2, 119.7, 115.5, 115.2, 107.2, 49.8, 47.3, 32.8, 22.3, 20.4, 20.2, 19.2.

HRMS (ESI) *m/z* calcd. for $C_{32}H_{35}NO_6S$ $(M+Na)^+$: 584.2077, found: 584.2074

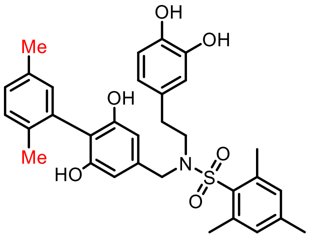

1r

The **1r** was obtained in 88% (0.8 g), colorless foam solid (silica gel flash chromatography: DCM: Acetone = 20:1). R_f = 0.40 (DCM: Acetone = 8:1).

^{1}H NMR (500 MHz, Acetone-d_6): δ 7.76 – 7.72 (m, 3H), 7.67 (s, 1H), 7.12 (d, *J* = 8.0 Hz, 1H), 7.05 (s, 2H), 7.01 (d, *J* = 8.0 Hz, 1H), 6.96 (s, 1H), 6.64 (d, *J* = 8.0 Hz, 1H), 6.52 (d, *J* = 9.0 Hz, 2H), 6.48 (d, *J* = 2.0 Hz, 1H), 6.31 (dd, *J* = 8.0, 2.0 Hz, 1H), 4.40 (s, 2H), 3.29 – 3.23 (m, 2H), 2.99 (s, 3H), 2.62 (s, 6H), 2.56 (dd, *J* = 13.5, 5.0 Hz, 2H), 2.32 (s, 3H), 2.28 (s, 3H), 2.08 (s, 3H).

^{13}C NMR (125 MHz, Acetone-d_6): δ 156.1, 145.6, 144.1, 143.0, 140.4, 137.7, 135.4, 135.0, 134.4, 134.1, 132.5, 132.4, 130.9, 130.2, 128.6, 120.3, 116.0, 116.0, 115.8, 107.8, 50.4, 47.8, 33.4, 22.9, 20.7, 20.7, 19.3.

HRMS (ESI) *m/z* calcd. for $C_{32}H_{35}NO_6S$ $(M+Na)^+$: 584.2077, found: 584.2069

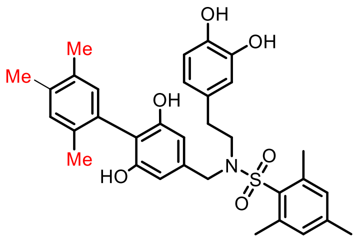

1s

The **1s** was obtained in 91% (1.1 g), colorless foam solid (silica gel flash chromatography: DCM: Acetone = 20:1). R_f = 0.40 (DCM: Acetone = 8:1).

^{1}H NMR (500 MHz, Acetone-d_6): δ 7.81 – 7.61 (m, *J* = 25.0 Hz, 3H), 7.04 (s, 2H), 7.00 (s, 1H), 6.90 (s, 1H), 6.66 (d, *J* = 6.5 Hz, 1H), 6.54 – 6.47 (m, 1H), 6.32 (d, *J* = 8.0 Hz, 1H), 4.40 (s, 1H), 3.33 – 3.20 (m, 1H), 2.62 (s, 2H), 2.59 – 2.54 (m, 1H), 2.32 (d, *J* = 15.0 Hz, 1H), 2.22 (s, 3H), 2.19 (s, 3H), 2.08 – 2.05 (m, 3H).

^{13}C NMR (125 MHz, Acetone-d_6): δ 155.8, 155.7, 145.2, 143.7, 142.7, 140.1, 137.3, 135.5, 135.5, 134.1, 133.4, 132.6, 132.2, 131.4, 130.8, 130.6, 119.9, 115.7, 115.6, 115.5, 107.4, 107.3, 50.1, 47.5, 33.1, 22.6, 20.4, 18.9, 18.7.

HRMS (ESI) *m/z* calcd. for $C_{33}H_{37}NO_6S$ $(M+Na)^+$: 598.2234, found: 598.2228

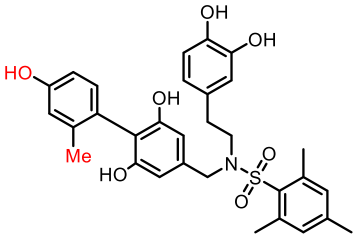

1t

The **1t** was obtained in 56% (0.596 g), colorless foam solid (silica gel flash chromatography: DCM: Acetone = 20:1). R_f = 0.40 (DCM: Acetone = 8:1).

^{1}H NMR (500 MHz, Acetone-d_6): δ 7.71 (s, 4H), 7.05 (s, 2H), 6.96 (d, J = 8.0 Hz, 1H), 6.75 (d, J = 2.5 Hz, 1H), 6.68 (dd, J = 8.0, 2.5 Hz, 1H), 6.64 (d, J = 8.0 Hz, 1H), 6.49 (s, 2H), 6.48 (d, J = 2.0 Hz, 1H), 6.31 (dd, J = 8.0, 2.0 Hz, 1H), 4.39 (s, 2H), 3.29 – 3.23 (m, 2H), 2.98 (s, 2H), 2.62 (s, 6H), 2.58 – 2.52 (m, 2H), 2.32 (s, 3H).

^{13}C NMR (125 MHz, Acetone-d_6): δ 157.5, 156.4, 145.6, 144.1, 143.1, 140.4, 140.2, 137.6, 134.5, 132.9, 132.6, 130.9, 124.5, 120.3, 117.3, 116.1, 115.8, 115.7, 113.3, 107.7, 50.4, 47.9, 33.4, 22.9, 20.7, 19.9.

HRMS (ESI) m/z calcd. for $C_{31}H_{33}NO_7S$ $(M+Na)^+$: 586.1870, found: 586.1865

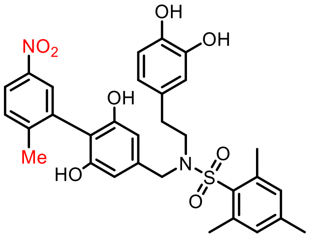

1u

The **1u** was obtained in 50% (0.60 g), colorless foam solid (silica gel flash chromatography: DCM: Acetone = 20:1). R_f = 0.40 (DCM: Acetone = 8:1).

^{1}H NMR (500 MHz, Acetone-d_6): δ 8.09 (dd, J = 8.5, 2.5 Hz, 1H), 8.04 (d, J = 2.5 Hz, 1H), 7.54 (d, J = 8.5 Hz, 1H), 7.05 (s, 2H), 6.64 (d, J = 8.0 Hz, 1H), 6.58 (s, 2H), 6.48 (d, J = 2.0 Hz, 1H), 6.30 (dd, J = 8.0, 2.0 Hz, 1H), 4.44 (s, 2H), 3.82 (s, 1H), 3.33 – 3.19 (m, 2H), 2.91 (s, 5H), 2.61 (d, J = 3.5 Hz, 6H), 2.59 – 2.48 (m, 2H), 2.32 (s, 3H), 2.28 (s, 3H).

^{13}C NMR (125 MHz, Acetone-d_6): δ 156.2, 147.3, 146.7, 145.7, 144.2, 143.2, 140.5, 139.3, 137.1, 134.6, 132.6, 131.2, 130.9, 126.8, 122.5, 120.3, 116.1, 115.9, 113.9, 107.9, 55.3, 50.5, 48.1, 33.5, 31.8, 22.9, 20.7, 20.0.

HRMS (ESI) *m/z* calcd. for $C_{31}H_{32}N_2O_8S$ $(M+Na)^+$: 615.1772, found: 615.1768

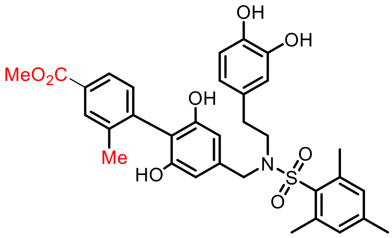

1v

The **1v** was obtained in 38% (0.45 g), colorless foam solid (silica gel flash chromatography: DCM: Acetone = 20:1). R_f = 0.40 (DCM: Acetone = 8:1).

^{1}H NMR (500 MHz, Acetone-d_6): δ 7.89 (s, 1H), 7.85 – 7.79 (m, 1H), 7.29 (d, *J* = 8.0 Hz, 1H), 7.05 (s, 2H), 6.64 (d, *J* = 8.0 Hz, 1H), 6.55 (s, 2H), 6.47 (d, *J* = 2.0 Hz, 1H), 6.30 (dd, *J* = 8.0, 2.0 Hz, 1H), 4.42 (s, 2H), 3.88 (s, 3H), 3.34 – 3.18 (m, 2H), 2.94 (s, 4H), 2.61 (d, *J* = 5.0 Hz, 6H), 2.59 – 2.48 (m, 2H), 2.32 (s, 3H), 2.22 (s, 3H).

^{13}C NMR (126 MHz, Acetone-d_6): δ 167.3, 156.0, 145.6, 144.2, 143.1, 140.5, 140.4, 139.1, 138.6, 134.5, 132.6, 132.25, 130.9, 130.9, 129.6, 126.8, 120.3, 116.1, 115.8, 115.2, 107.9, 55.2, 51.9, 50.5, 47.9, 33.4, 22.9, 20.7, 19.7.

HRMS (ESI) *m/z* calcd. for $C_{33}H_{35}NO_8S$ $(M+Na)^+$: 628.1976, found: 628.1961

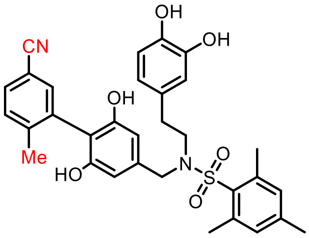

1w

The **1w** was obtained in 55% (0.5 g), colorless foam solid (silica gel flash chromatography: DCM: Acetone = 20:1). R_f = 0.40 (DCM: Acetone = 8:1).

^{1}H NMR (500 MHz, Acetone-d_6): δ 7.59 (d, *J* = 8.0 Hz, 1H), 7.52 (s, 1H), 7.46 (d, *J* = 8.0 Hz, 1H), 7.05 (s, 2H), 6.64 (d, *J* = 8.0 Hz, 1H), 6.56 (d, *J* = 6.5 Hz, 2H), 6.47 (s, 1H), 6.29 (d, *J* = 6.5 Hz, 1H), 4.43 (s, 3H), 3.29 – 3.23 (m, 2H), 2.61 (s, 6H), 2.56 – 2.51 (m, 2H), 2.32 (s, 3H), 2.23 (s, 3H).

^{13}C NMR (125 MHz, Acetone-d_6): δ 156.1, 145.6, 144.9, 144.2, 143.1, 140.4, 139.0, 136.9, 135.4, 134.5, 132.6, 131.2, 131.1, 130.8, 120.3, 119.5, 116.1, 115.8, 113.9, 109.7, 107.8, 55.2, 50.4, 48.0, 33.4, 22.9, 20.7, 20.1.

HRMS (ESI) *m/z* calcd. for $C_{32}H_{32}N_2O_6S$ $(M+Na)^+$: 595.1873, found: 595.1872

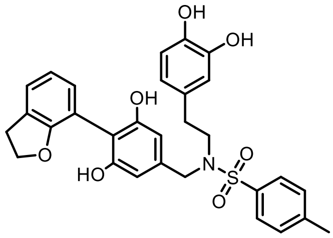

1x

The **1x** was obtained in 95% (0.836 g), colorless foam solid (silica gel flash chromatography: DCM: Acetone = 20:1). R_f = 0.40 (DCM: Acetone = 8:1).

¹H NMR (500 MHz, CD_3OD): δ 7.69 (d, *J* = 8.0 Hz, 2H), 7.36 (d, *J* = 8.0 Hz, 2H), 7.16 – 7.08 (m, 1H), 6.88 – 6.83 (m, 1H), 6.61 (dd, *J* = 8.0, 2.5 Hz, 1H), 6.51 (s, 1H), 6.45 (s, 3H), 6.33 – 6.28 (m, 1H), 4.89 (s, 4H), 4.25 – 4.19 (m, 2H), 3.62 (s, 2H), 3.32 – 3.17 (m, 4H), 2.55 – 2.47 (m, 2H), 2.42 (s, 3H).

¹³C NMR (125 MHz, CD_3OD): δ 159.2, 157.0, 146.2, 144.9, 144.8, 138.6, 138.3, 131.9, 131.5, 130.9, 128.3, 128.2, 125.0, 121.2, 121.0, 117.2, 116.7, 116.4, 113.3, 108.4, 71.8, 62.8, 60.9, 36.3, 30.8, 21.6.

HRMS (ESI) *m/z* calcd. for $C_{30}H_{29}NO_7S$ $(M+Na)^+$: 570.1557, found: 570.1553

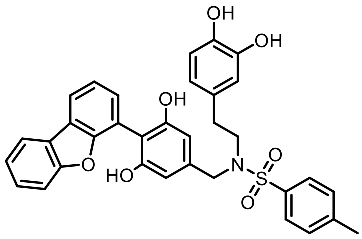

1y

The **1y** was obtained in 81% (0.85 g), colorless foam solid (silica gel flash chromatography: DCM: Acetone = 20:1). R_f = 0.40 (DCM: Acetone = 8:1).

¹H NMR (500 MHz, Acetone-*d*$_6$): δ 8.19 (s, 2H), 8.11 – 8.07 (m, 1H), 8.04 (dd, *J* = 7.5, 1.5 Hz, 1H), 7.83 – 7.77 (m, 3H), 7.76 – 7.74 (m, 1H), 7.53 (d, *J* = 8.0 Hz, 1H), 7.50 – 7.39 (m, 5H), 7.38 – 7.33 (m, 1H), 6.75 – 6.70 (m, 1H), 6.67 (s, 2H), 6.62 (d, *J* = 2.0 Hz, 1H), 6.47 – 6.42 (m, *J* = 8.0, 2.0 Hz, 1H), 4.36 (s, 2H), 3.39 – 3.32 (m, 2H), 3.22 – 3.15 (m, 1H), 2.61 (dd, *J* = 9.5, 7.0 Hz, 2H), 2.42 (s, 3H).

¹³C NMR (125 MHz, Acetone-*d*$_6$): δ 156.7, 156.3, 155.3, 145.4, 143.9, 143.6, 139.2, 138.1, 130.8, 130.7, 130.1, 127.5, 127.4, 124.9, 124.4, 123.0, 122.9, 121.0, 120.3, 120.0, 119.4, 116.0, 115.7, 111.9, 110.9, 107.4, 52.0, 50.4, 34.5, 20.9.

HRMS (ESI) *m/z* calcd. for $C_{34}H_{29}NO_7S$ $(M+Na)^+$: 618.1557, found: 618.1555

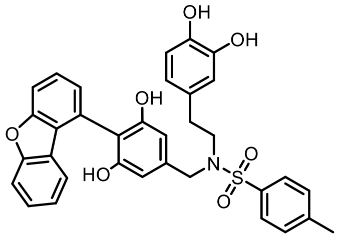

1z

The **1z** was obtained in 97% (1.07 g), colorless foam solid (silica gel flash chromatography: DCM: Acetone = 20:1). R_f = 0.40 (DCM: Acetone = 8:1).

¹H NMR (500 MHz, Acetone-*d₆*): δ 7.99 (s, 2H), 7.83 (d, *J* = 8.0 Hz, 2H), 7.61 – 7.51 (m, 3H), 7.45 (d, *J* = 8.0 Hz, 2H), 7.40 – 7.36 (m, 1H), 7.32 (dd, *J* = 10.5, 4.0 Hz, 2H), 7.03 (t, *J* = 7.5 Hz, 1H), 6.73 (s, 2H), 6.71 (d, *J* = 8.0 Hz, 1H), 6.62 (d, *J* = 2.0 Hz, 1H), 6.45 (dd, *J* = 8.0, 2.0 Hz, 1H), 4.38 (s, 2H), 3.36 (dd, *J* = 10.0, 7.0 Hz, 2H), 3.02 (s, 2H), 2.66 – 2.60 (dd, *J* = 9.5, 7.0 Hz, 2H), 2.44 (s, 3H).

¹³C NMR (125 MHz, Acetone-*d₆*): δ 156.8, 156.6, 145.6, 144.1, 143.9, 139.7, 138.1, 131.0, 130.4, 130.2, 127.7, 127.5, 127.4, 126.6, 125.2, 124.4, 123.1, 122.7, 120.4, 116.2, 115.9, 113.8, 111.6, 110.6, 107.8, 52.7, 51.1, 35.3, 21.1.

HRMS (ESI) *m/z* calcd. for $C_{34}H_{29}NO_7S$ $(M+Na)^+$: 618.1557, found: 618.1553

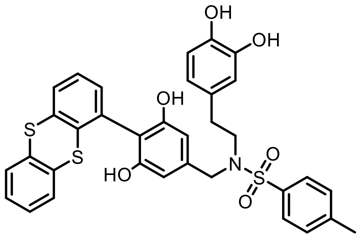

1aa

The **1aa** was obtained in 87% (1.116 g), colorless foam solid (silica gel flash chromatography: DCM: Acetone = 20:1). R_f = 0.40 (DCM: Acetone = 8:1).

¹H NMR (500 MHz, CD_3OD): δ 7.73 (d, *J* = 8.0 Hz, 2H), 7.48 – 7.36 (m, 5H), 7.28 – 7.20 (m, 3H), 7.18 (dd, *J* = 13.0, 6.5 Hz, 1H), 7.10 (dt, *J* = 15.0, 7.5 Hz, 2H), 6.63 (d, *J* = 8.0 Hz, 1H), 6.52 (s, 3H), 6.39 (dd, *J* = 14.0, 8.0 Hz, 1H), 4.25 (s, 2H), 3.29 – 3.25 (m, 2H), 2.55 – 2.49 (m, 2H), 2.42 (s, 3H).

¹³C NMR (125 MHz, CD_3OD): δ 157.0, 146.3, 145.0, 144.8, 139.4, 138.3, 138.1, 137.9, 137.5, 136.4, 136.2, 132.0, 131.6, 130.9, 130.7, 129.8, 129.1, 128.5, 128.5, 128.5, 128.2, 128.0, 127.9, 121.2, 116.8,

116.4, 115.9, 108.2, 53.6, 51.4, 36.0, 21.5.

HRMS (ESI) *m/z* calcd. for $C_{34}H_{29}NO_6S_3$ $(M+Na)^+$: 666.1049, found: 666.1041

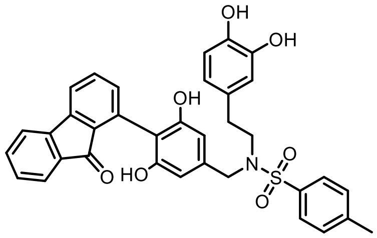

1ab

The **1ab** was obtained in 60% (0.438 g), yellow foam solid (silica gel flash chromatography: DCM: Acetone = 20:1). R_f = 0.40 (DCM: Acetone = 8:1).

^{1}H NMR (500 MHz, Acetone-d_6) : δ 7.81 (d, *J* = 9.0 Hz, 2H), 7.76 (d, *J* = 7.5 Hz, 1H), 7.71 (dd, *J* = 14.0, 7.5 Hz, 2H), 7.57 (td, *J* = 7.5, 4.0 Hz, 2H), 7.53 (d, *J* = 7.0 Hz, 1H), 7.45 (d, *J* = 8.0 Hz, 2H), 7.40 – 7.31 (m, 2H), 7.28 (d, *J* = 8.0 Hz, 1H), 6.70 (d, *J* = 8.0 Hz, 1H), 6.64 – 6.61 (m, *J* = 7.5 Hz, 1H), 6.48 – 6.44 (m, *J* = 8.0 Hz, 1H), 4.30 (s, 2H), 3.33 – 3.25 (m, 2H), 2.66 – 2.55 (m, *J* = 16.5, 8.5 Hz, 3H), 2.44 (s, 3H).

^{13}C NMR (125 MHz, Acetone-d_6) δ 193.3, 156.2, 145.4, 145.2, 144.7, 144.1, 143.8, 138.5, 138.1, 135.3, 134.8, 134.7, 134.5, 134.2, 132.7, 134.0, 130.4, 130.1, 129.6, 127.8, 127.6, 124.0, 121.0, 120.7, 120.6, 119.9, 116.3, 116.2, 115.8, 115.7, 113.1, 52.3, 50.6, 34.8, 21.2.

HRMS (ESI) *m/z* calcd. for $C_{35}H_{29}NO_7S$ $(M+Na)^+$: 630.1557, found: 630.1555

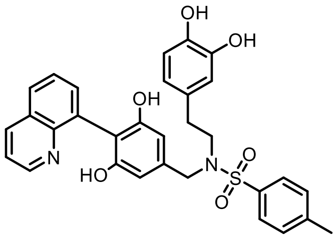

1ac

The **1ac** was obtained in 75% (0.196 g), colorless foam solid (silica gel flash chromatography: DCM: Acetone = 20:1). R_f = 0.40 (DCM: Acetone = 8:1).

^{1}H NMR (500 MHz, Acetone-d_6): δ 8.88 (d, *J* = 4.0 Hz, 1H), 8.44 (d, *J* = 8.0 Hz, 1H), 8.26 (s, 2H), 7.98 (d, *J* = 8.0 Hz, 1H), 7.88 (d, *J* = 7.0 Hz, 1H), 7.80 (d, *J* = 8.0 Hz, 3H), 7.68 (t, *J* = 7.5 Hz, 2H), 7.56 (dd, *J* = 8.0, 4.0 Hz, 1H), 7.45 (d, *J* = 8.0 Hz, 2H), 6.69 (d, *J* = 8.0 Hz, 1H), 6.64

(s, 2H), 6.55 (s, 1H), 6.43 (d, *J* = 8.0 Hz, 1H), 4.34 (s, 2H), 3.38 – 3.32 (m, 2H), 2.88 (d, *J* = 15.5 Hz, 8H), 2.88 (d, *J* = 15.5 Hz, 8H), 2.62 – 2.57 (m, 2H), 2.45 (s, 3H).

^{13}C NMR (125 MHz, Acetone-d_6): δ 157.0, 150.6, 147.5, 145.7, 144.3, 143.9, 139.0, 138.4, 138.0, 134.5, 134.3, 131.0, 130.5, 129.5, 128.4, 127.8, 127.1, 121.8, 120.6, 116.4, 115.9, 115.7, 108.9, 52.3, 50.8, 34.8, 21.2.

HRMS (ESI) *m/z* calcd. for $C_{31}H_{28}N_2O_6S$ $(M+Na)^+$: 579.1560, found: 579.1556

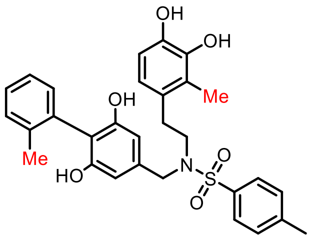

1ad

The **1ad** was obtained in 61% (0.49 g), colorless foam solid (silica gel flash chromatography: DCM: Acetone = 20:1). R_f = 0.40 (DCM: Acetone = 8:1).

^{1}H NMR (500 MHz, Acetone-d_6): δ 8.16 (s, 1H), 7.86 (s, 2H), 7.81 (d, *J* = 8.5 Hz, 2H), 7.44 (d, *J* = 8.0 Hz, 2H), 7.27 – 7.14 (m, 4H), 7.03 (s, 1H), 6.61 (s, 2H), 6.59 – 6.55 (m, 1H), 6.36 – 6.32 (m, 1H), 4.30 (s, 2H), 3.20 – 3.13 (m, 2H), 3.04 (s, 2H), 2.66 – 2.58 (m, 2H), 2.43 (s, 3H), 2.16 (s, 3H), 2.04 (s, 3H).

^{13}C NMR (125 MHz, Acetone-d_6): δ 156.1, 144.0, 143.8, 143.4, 138.4, 138.3, 137.9, 134.5, 131.8, 130.3, 130.1, 129.5, 127.8, 127.7, 125.9, 123.3, 120.6, 116.0, 112.7, 107.7, 52.8, 49.7, 33.4, 21.1, 19.7, 11.3.

HRMS (ESI) *m/z* calcd. for $C_{30}H_{31}NO_6S$ $(M+Na)^+$: 556.1764, found: 556.1757

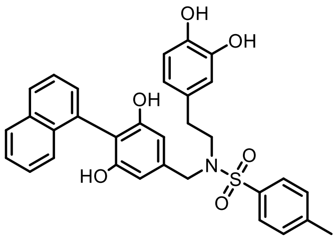

1ae

The **1ae** was obtained in 85% (0.85 g), colorless foam solid (silica gel flash chromatography: DCM: Acetone = 20:1). R_f = 0.40 (DCM: Acetone = 8:1).

^{1}H NMR (500 MHz, Acetone-d_6): δ 7.94 – 7.84 (m, 4H), 7.81 (d, J = 8.0 Hz, 3H), 7.75 (s, 1H), 7.63 (d, J = 8.5 Hz, 1H), 7.55 – 7.51 (m, 1H), 7.48 – 7.42 (m, 4H), 7.41 – 7.36 (m, 1H), 6.71 (d, J = 8.0 Hz, 1H), 6.66 (s, 2H), 6.61 (d, J = 2.0 Hz, 1H), 6.42 (dd, J = 8.0, 2.0 Hz, 1H), 4.36 (s, 2H), 3.35 (dd, J = 9.5, 7.0 Hz, 2H), 3.11 (s, 2H), 2.61 (dd, J = 9.5, 7.0 Hz, 2H), 2.43 (s, 3H).

^{13}C NMR (125 MHz, Acetone-d_6): δ 156.7, 145.5, 144.0, 143.7, 138.8, 138.1, 134.4, 133.5, 132.7, 130.8, 130.2, 129.4, 128.5, 128.1, 127.6, 126.6, 126.1, 126.0, 125.9, 120.3, 116.1, 115.7, 114.3, 107.6, 52.2, 50.6, 34.8, 21.0.

HRMS (ESI) *m/z* calcd. for $C_{32}H_{29}NO_6S$ $(M+Na)^+$: 578.1608, found: 578.1595.

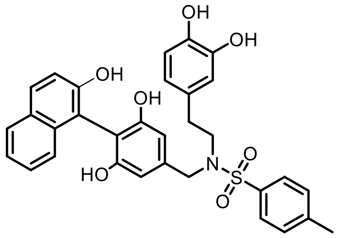

1af

The **1af** was obtained in 51% (0.45 g), colorless foam solid (silica gel flash chromatography: DCM: Acetone = 20:1). R_f = 0.40 (DCM: Acetone = 8:1).

^{1}H NMR (500 MHz, Acetone-d_6): δ 7.79 (dd, J = 14.0, 8.0 Hz, 4H), 7.44 (d, J = 8.0 Hz, 2H), 7.38 (d, J = 8.0 Hz, 1H), 7.31 – 7.22 (m, 3H), 6.70 (d, J = 8.0 Hz, 1H), 6.64 (s, 2H), 6.60 (d, J = 2.0 Hz, 1H), 6.43 (dd, J = 8.0, 2.0 Hz, 1H), 4.36 (s, 2H), 3.38 – 3.31 (m, 2H), 2.63 – 2.57 (m, 2H), 2.43 (s, 3H).

^{13}C NMR (125 MHz, Acetone-d_6): δ 157.4, 153.9, 145.4 , 144.0, 143.7 , 139.2, 138.2, 134.9, 130.9, 130.2, 129.8, 129.5, 128.3, 127.6, 126.4, 125.2, 123.0, 120.4, 118.9, 116.1, 115.7, 113.1, 108.9, 107.6, 52.2, 50.5, 34.8, 21.0.

HRMS (ESI) *m/z* calcd. for $C_{32}H_{29}NO_7S$ $(M+Na)^+$: 594.1557, found: 594.1546.

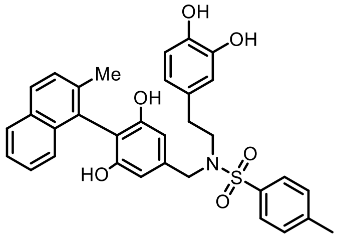

1ag

The **1ag** was obtained in 60% (0.709 g), colorless foam solid (silica gel flash chromatography:

DCM: Acetone = 20:1). R_f = 0.40 (DCM: Acetone = 8:1).

^{1}H NMR (500 MHz, Acetone-d_6): δ 7.86 – 7.71 (m, 7H), 7.49 (d, J = 8.5 Hz, 1H), 7.43 (dd, J = 8.0, 3.5 Hz, 3H), 7.37 (dd, J = 11.0, 4.0 Hz, 1H), 7.34 – 7.30 (m, 1H), 6.75 – 6.66 (m, 3H), 6.62 (d, J = 2.0 Hz, 1H), 6.42 (dd, J = 8.0, 2.0 Hz, 1H), 4.36 (s, 2H), 3.34 (dd, J = 9.5, 7.0 Hz, 2H), 2.66 – 2.57 (m, 2H), 2.42 (s, 3H), 2.30 (s, 3H).

^{13}C NMR (125 MHz, Acetone-d_6): δ 156.4, 145.4, 143.9, 143.6, 138.7, 137.9, 136.0, 133.7, 132.8, 130.8, 130.4, 130.1, 129.0, 128.1, 127.8, 127.5, 126.0, 126.0, 124.9, 120.3, 116.0, 115.6, 112.8, 107.6, 52.4, 50.7, 35.0, 21.0, 20.1.

HRMS (ESI) *m/z* calcd. for $C_{33}H_{31}NO_6S$ $(M+Na)^+$: 592.1764, found: 592.1764.

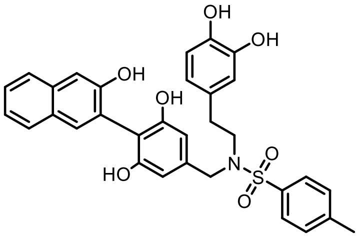

1ah

The **1ah** was obtained in 65% (0.70 g), colorless foam solid (silica gel flash chromatography: DCM: Acetone = 20:1). R_f = 0.40 (DCM: Acetone = 4:1).

^{1}H NMR (500 MHz, Acetone-d_6): δ 8.13 (s, 1H), 8.01 (s, 2H), 7.82 – 7.77 (m, 3H), 7.76 – 7.72 (m, 3H), 7.70 (d, J = 8.0 Hz, 1H), 7.44 (d, J = 8.0 Hz, 2H), 7.41 – 7.37 (m, 1H), 7.31 (s, 1H), 7.30 – 7.26 (m, 1H), 6.69 (d, J = 8.0 Hz, 1H), 6.61 (s, 2H), 6.57 (d, J = 2.0 Hz, 1H), 6.41 (dd, J = 8.0, 2.0 Hz, 1H), 4.33 (s, 2H), 3.32 (dd, J = 9.5, 7.0 Hz, 2H), 3.00 (s, 4H), 2.61 – 2.54 (m, 2H), 2.44 (s, 3H).

^{13}C NMR (125 MHz, Acetone-d_6): δ 156.8, 154.6, 145.6, 144.2, 143.8, 139.0, 138.4, 135.3, 132.8, 130.9, 130.3, 129.3, 128.2, 127.7, 126.5, 126.4, 124.5, 123.4, 120.5, 116.2, 115.8, 112.7, 110.4, 107.9, 52.1, 50.5, 34.7 21.1.

HRMS (ESI) *m/z* calcd. for $C_{32}H_{29}NO_7S$ $(M+Na)^+$: 594.1557, found: 594.1544.

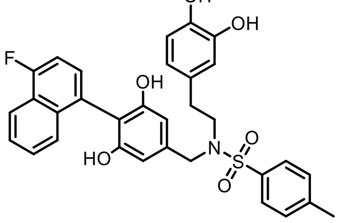

1ai

The **1ai** was obtained in 79% (0.868 g), colorless foam solid (silica gel flash chromatography: DCM: Acetone = 20:1). R_f = 0.40 (DCM: Acetone = 8:1).

^{1}H NMR (500 MHz,) ^{1}H NMR (500 MHz, Acetone) δ 8.11 (d, *J* = 8.0 Hz, 1H), 7.82 (dd, *J* = 12.5, 8.5 Hz, 3H), 7.64 (d, *J* = 8.5 Hz, 1H), 7.58 (t, *J* = 7.5 Hz, 1H), 7.52 – 7.40 (m, 5H), 7.29 (dd, *J* = 11.0, 8.0 Hz, 1H), 6.70 (dd, *J* = 10.5, 4.0 Hz, 1H), 6.66 (s, 2H), 6.60 (d, *J* = 2.0 Hz, 1H), 6.42 (dd, *J* = 8.0, 2.0 Hz, 1H), 4.36 (s, 2H), 3.34 (dd, *J* = 9.5, 7.0 Hz, 2H), 2.60 (dd, *J* = 9.5, 7.0 Hz, 2H), 2.43 (s, 3H).

^{13}C NMR (125 MHz, Acetone-d_6): δ159.02 (d, *J* = 249.2 Hz), 157.7, 156.8, 145.5, 144.0, 143.7, 139.2, 138.1, 134.9, 134.8, 130.9, 130.3, 129.68 (d, *J* = 8.2 Hz), 127.7, 127.6, 127.1, 126.9, 126.9, 126.5, 124.58 (d, *J* = 16.3 Hz), 120.83 (d, *J* = 5.5 Hz)., 120.4, 119.5, 117.8, 116.1, 115.8, 113.5, 109.7, 109.6, 107.7, 107.6, 52.3, 50.6, 34.9, 21.1.

^{19}F NMR (470 MHz, CD_3OD): δ -127.0.

HRMS (ESI) *m/z* calcd. for $C_{32}H_{28}NO_6SF$ $(M+Na)^+$: 596.1514, found: 596.1504.

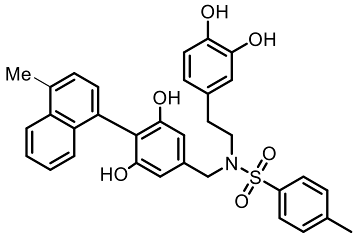

1aj

The **1aj** was obtained in 68% (0.748 g), colorless foam solid (silica gel flash chromatography: DCM: Acetone = 20:1). R_f = 0.40 (DCM: Acetone = 8:1).

^{1}H NMR (500 MHz, Acetone-d_6): δ 8.04 (d, *J* = 8.4 Hz, 1H), 7.80 (d, *J* = 8.0 Hz, 2H), 7.64 (d, *J* = 8.0 Hz, 1H), 7.50 (ddd, *J* = 8.0, 7.0, 1.2 Hz, 1H), 7.43 (d, *J* = 8.0 Hz, 2H), 7.41 – 7.36 (m, 2H), 7.32 (d, *J* = 7.0 Hz, 1H), 6.70 (dd, *J* = 11.0, 5.0 Hz, 1H), 6.65 (s, 2H), 6.61 (d, *J* = 2.0 Hz, 1H), 6.42 (dd, *J* = 8.0, 2.0 Hz, 1H), 4.35 (s, 2H), 3.34 (dt, *J* = 19.5, 8.5 Hz, 2H), 2.71 (s, 3H), 2.60 (dd, *J* = 9.5, 7.0 Hz, 2H), 2.43 (s, 3H).

^{13}C NMR (125 MHz, Acetone-d_6): δ 156.8, 145.5, 144.0, 143.7, 138.7, 138.2, 134.1, 133.5, 133.5, 130.9, 130.8, 130.2, 129.2, 127.6, 127.2, 126.9, 125.9, 125.7, 124.6, 120.4, 116.1, 115.7, 114.5, 107.6, 52.3, 50.6, 34.9, 21.1, 19.2.

HRMS (ESI) *m/z* calcd. for $C_{33}H_{31}NO_6S$ $(M+Na)^+$: 592.1764, found: 592.1759.

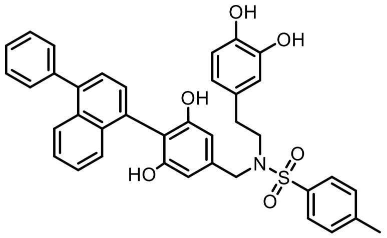

1ak

The **1ak** was obtained in 81% (0.89 g), colorless foam solid (silica gel flash chromatography: DCM: Acetone = 20:1). R_f = 0.40 (DCM: Acetone = 8:1).

^{1}H NMR (500 MHz, Acetone-d_6): δ 7.91 (dd, J = 7.0, 2.0 Hz, 1H), 7.81 (d, J = 8.0 Hz, 2H), 7.75 – 7.71 (m, 1H), 7.55 – 7.50 (m, 5H), 7.50 – 7.36 (m, 7H), 6.73 – 6.70 (m, 1H), 6.69 (s, 2H), 6.62 (d, J = 2.0 Hz, 1H), 6.43 (dd, J = 8.0, 2.0 Hz, 1H), 4.37 (s, 2H), 3.36 (dd, J = 9.5, 7.0 Hz, 2H), 2.62 (dd, J = 9.5, 7.0 Hz, 2H), 2.43 (s, 3H).

^{13}C NMR (125 MHz, Acetone-d_6): δ 156.8, 145.6, 144.1, 143.7, 141.5, 140.1, 139.0, 138.2, 133.8, 132.7, 132.3, 130.9, 130.5, 130.3, 129.0, 128.9, 127.7, 127.6, 127.2, 127.1, 126.3, 126.1, 125.9, 120.4, 116.2, 115.8, 114.4, 107.7, 52.3, 50.7, 34.9, 21.1.

HRMS (ESI) *m/z* calcd. for $C_{38}H_{33}NO_6S$ $(M+Na)^+$: 654.1921, found: 654.1909.

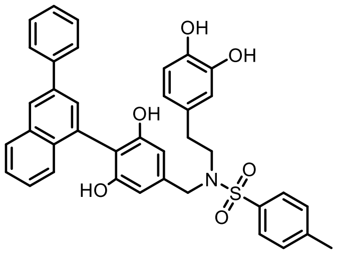

1al

The **1al** was obtained in 44% (0.56 g), colorless foam solid (silica gel flash chromatography: DCM: Acetone = 20:1). R_f = 0.40 (DCM: Acetone = 8:1).

^{1}H NMR (500 MHz, Acetone-d_6): δ 7.95 (dd, J = 8.0, 2.5 Hz, 2H), 7.81 (s, 1H), 7.78 (d, J = 8.0 Hz, 2H), 7.61 (d, J = 8.5 Hz, 1H), 7.54 (d, J = 8.5 Hz, 1H), 7.48 (t, J = 7.0 Hz, 1H), 7.45 – 7.38 (m, 5H), 7.12 (t, J = 7.5 Hz, 2H), 7.05 (t, J = 7.5 Hz, 1H), 6.73 (d, J = 8.0 Hz, 1H), 6.58 (d, J = 2.0 Hz, 1H), 6.49 (s, 2H), 6.39 (dd, J = 8.0, 2.0 Hz, 1H), 4.24 (s, 2H), 3.22 (dd, J = 10.0, 7.0 Hz, 2H), 2.93 (s, 3H), 2.46 (dd, J = 10.0, 7.0 Hz, 2H), 2.42 (s, 3H).

^{13}C NMR (125 MHz, Acetone-d_6): δ 157.0, 145.7, 144.2, 143.8, 143.1, 141.2, 139.0, 138.2, 134.1, 133.7, 131.2, 130.4, 129.5, 128.9, 128.5, 128.4, 127.9, 127.7, 127.1, 126.6, 126.1, 120.6, 116.4, 115.9,

113.5, 107.55, 52.7, 51.0, 35.3, 21.2.

HRMS (ESI) *m/z* calcd. for $C_{38}H_{33}NO_6S$ $(M+Na)^+$: 654.1921, found: 654.1927.

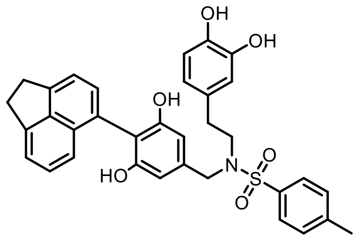

1am

The **1am** was obtained in 73% (0.758 g), colorless foam solid (silica gel flash chromatography: DCM: Acetone = 20:1). R_f = 0.40 (DCM: Acetone = 8:1).

^{1}H NMR (500 MHz, Acetone-*d_6*): δ 7.81 (d, *J* = 8.5 Hz, 2H), 7.78 – 7.70 (m, 2H), 7.47 – 7.43 (m, 2H), 7.41 – 7.37 (m, 1H), 7.37 – 7.32 (m, 2H), 7.31 – 7.23 (m, 2H), 6.70 (dd, *J* = 7.5, 3.5 Hz, 1H), 6.66 – 6.63 (m, 2H), 6.60 (d, *J* = 2.0 Hz, 1H), 6.44 – 6.39 (m, 1H), 4.35 (s, 3H), 3.47 – 3.38 (m, 4H), 3.36 – 3.31 (m, 2H), 2.95 (s, 2H), 2.63 – 2.57 (m, 2H), 2.44 (s, 3H).

^{13}C NMR (125 MHz, Acetone-*d_6*): δ 156.9, 146.5, 146.1, 145.7, 144.2, 143.9, 140.2, 138.8, 138.4, 132.1, 131.3, 131.1, 130.4, 128.0, 127.8, 127.6, 122.0, 120.5, 120.0, 119.5, 116.3, 115.9, 114.0, 107.8, 52.4, 50.7, 35.0, 30.7, 30.4, 21.2.

HRMS (ESI) *m/z* calcd. for $C_{34}H_{31}NO_6S$ $(M+Na)^+$: 604.1764, found: 604.1764.

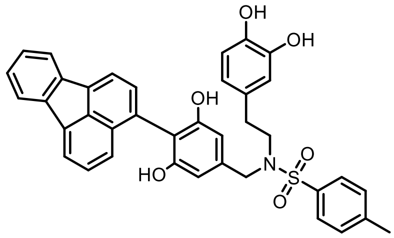

1an

The **1an** was obtained in 59% (0.70 g), colorless foam solid (silica gel flash chromatography: DCM: Acetone = 20:1). R_f = 0.40 (DCM: Acetone = 8:1).

^{1}H NMR (500 MHz, Acetone-*d_6*): δ 8.09 (d, *J* = 7.0 Hz, 1H), 8.01 (ddd, *J* = 6.5, 5.5, 3.0 Hz, 3H), 7.81 (d, *J* = 8.0 Hz, 2H), 7.64 (dd, *J* = 7.5, 5.5 Hz, 2H), 7.58 (dd, *J* = 8.0, 7.0 Hz, 1H), 7.43 (d, *J* = 8.0 Hz, 2H), 7.41 – 7.37 (m, 2H), 6.73 (d, *J* = 8.0 Hz, 1H), 6.70 (s, 2H), 6.63 (d, *J* = 2.0 Hz, 1H), 6.44 (dd, *J* = 8.0, 2.0 Hz, 1H), 4.38 (s, 2H), 3.40 – 3.33 (m, 2H), 3.18 (s, 1H), 2.65 –

2.59 (m, 2H), 2.42 (s, 3H).

^{13}C NMR (125 MHz, Acetone-d_6): δ 156.7, 145.4, 143.9, 143.6, 139.8, 139.7, 139.1, 138.0, 137.2, 136.6 , 133.0, 131.4 , 130.8, 130.6, 130.2, 128.0, 127.5, 126.5, 121.9, 120.7, 120.3, 116.1, 115.7, 113.3, 107.6 , 52.2, 50.6, 34.8, 21.0.

HRMS (ESI) *m/z* calcd. for $C_{38}H_{31}NO_6S$ $(M+Na)^+$: 652.1764, found: 652.1750.

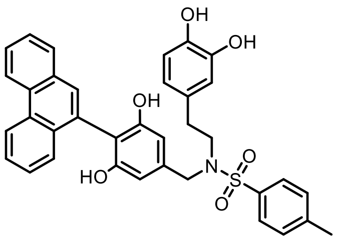

1ao

The **1ao** was obtained in 63% (0.7 g), colorless foam solid (silica gel flash chromatography: DCM: Acetone = 20:1). R_f = 0.40 (DCM: Acetone = 8:1).

^{1}H NMR (500 MHz, Acetone-d_6): δ 8.83 (t, *J* = 8.0 Hz, 2H), 7.98 – 7.95 (m, 1H), 7.83 (m, 5H), 7.77 (s, 1H), 7.73 – 7.68 (m, 2H), 7.68 – 7.61 (m, 2H), 7.54 – 7.49 (m, 1H), 7.44 (d, *J* = 8.0 Hz, 2H), 6.73 (d, *J* = 8.0 Hz, 1H), 6.70 (s, 2H), 6.63 (d, *J* = 2.0 Hz, 1H), 6.48 – 6.44 (m, 1H), 4.39 (s, 2H), 3.40 – 3.34 (m, 2H), 2.63 (dd, *J* = 9.5, 7.0 Hz, 2H), 2.43 (s, 3H).

^{13}C NMR (125 MHz, Acetone-d_6): δ 156.4, 145.1, 143.6, 143.3, 138.6, 137.7, 132.2, 132.1, 130.7, 130.7 , 130.4, 130.4, 129.9, 129.8, 129.5, 128.6, 127.2, 126.9, 126.7, 126.6, 126.6, 126.4, 126.4, 122.7, 122.5 , 119.9, 115.7, 115.3, 113.7, 107.2, 51.8, 50.2, 34.4, 20.6.

HRMS (ESI) *m/z* calcd. for $C_{36}H_{31}NO_6S$ $(M+Na)^+$: 628.1764, found: 628.1755

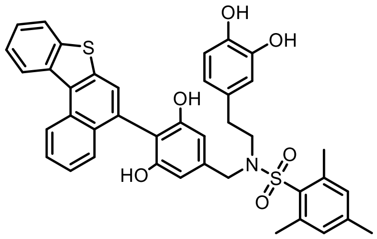

1ap

The **1ap** was obtained in 58% (0.7 g), colorless foam solid (silica gel flash chromatography: DCM: Acetone = 20:1). R_f = 0.40 (DCM: Acetone = 8:1).

^{1}H NMR (500 MHz, Acetone-d_6): δ 9.15 (d, *J* = 8.5 Hz, 2H), 9.01 (d, *J* = 8.0 Hz, 1H), 8.20 –

8.10 (m, J = 8.0 Hz, 2H), 7.96 (s, 1H), 7.89 – 7.75 (m, 3H), 7.68 (t, J = 7.5 Hz, 1H), 7.55 (dt, J = 22.5, 7.5 Hz, 2H), 7.07 (s, 2H), 6.73 – 6.62 (m, 3H), 6.54 (s, 1H), 6.37 (d, J = 7.5 Hz, 1H), 4.51 (s, 2H), 3.35 (s, 2H), 3.10 – 2.94 (m, 5H), 2.69 – 2.60 (m, 8H), 2.32 (s, 3H).

^{13}C NMR (125 MHz, Acetone-d_6): δ 156.9, 145.5, 144.1, 143.0, 140.3, 140.1, 138.9, 138.9, 137.1, 134.4, 133.0, 132.5, 132.2, 131.5, 130.8, 129.1, 128.1, 127.6, 125.9, 125.7, 125.4, 125.3, 124.5, 123.8, 123.8, 120.2, 116.0, 115.7, 107.9, 50.4, 47.9, 33.4, 22.8, 20.6.

HRMS (ESI) *m/z* calcd. for $C_{40}H_{35}NO_6S_2$ $(M+Na)^+$: 712.1798, found: 712.1781

3. Preparation of 1aq-1as, 1ba:

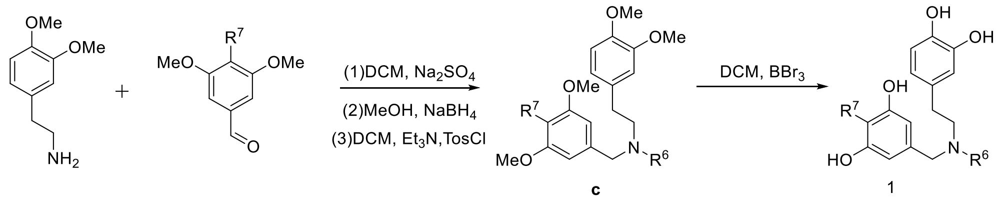

Supplementary Fig. 2: General procedure for **1aq-1as**, **1ba** preparation

General procedure for **1aq-1as**, **1ba** preparation (**Supplementary Fig. 2**): Under argon atmosphere, 3, 4-dimethoxyphenethylamine (1.0 equiv) was dissolved in dry DCM (200 mL) at room temperature, Na_2SO_4 (5 equiv) and substituted benzaldehyde (1.0 equiv) was added subsequently. The resulted solution was stirred at room temperature for 4 hours, after which the system was filtered to remove sodium sulfate and concentrate under reduced pressure, then cooled to 0 ℃, the residue dissolved in MeOH (200 mL), $NaBH_4$ (1.0 equiv) was added portionwise and the reaction mixture was stirred for additional 4 hours at room temperature. The reaction was quenched by addition of a saturated solution of H_2O (10 mL) then concentrated under vacuum and the residue was extracted with EtOAc (3 × 150 mL). The combined organic layer was wash with brine, dried over Na_2SO_4 and concentrated under vacuum. Without purification, the obtained residue was dissolved in anhydrous DCM in a dried round bottom flask under argon atmosphere. Et_3N (2.4 eq) and TosCl (1.2 eq) was added successively under argon atmosphere at 0 ℃. The reaction mixture was stirred for 45 mins with natural warming. Then the reaction was quenched by addition of a saturated solution of $NaHCO_3$ (100 mL) and the reaction mixture was extracted with DCM (3 × 150 mL). The combined organic layer was washed with brine, dried over Na_2SO_4 and concentrated under vacuum, the residue was purified by a flash column chromatography on silica

gel (petroleum ether/ethyl acetate = 20:1 to 1:1) to give product **c** as colorless solid.

Under argon atmosphere, **c** (1.0 eq) was dissolved in dry DCM (50 mL) and BBr_3 (4 eq) was added successively under argon atmosphere at 0 ℃, stirred for 12 hours. The reaction was quenched by addition of MeOH (50 mL) then concentrated under vacuum, and the residue was extracted with EtOAc (3 × 100 mL). The combined organic layer was wash with brine, dried over Na_2SO_4 and concentrated under vacuum, and chromatographied on silica gel (DCM: Acetone = 20:1 as eluent) to give **1aq-1as** and **1ba**.

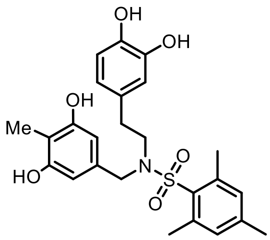

1aq

The **1aq** was obtained in 62% (0.4 g), colorless foam solid (silica gel flash chromatography: DCM: Acetone = 20:1). R_f = 0.50 (DCM: Acetone = 8:1).

^{1}H NMR (500 MHz, CD_3OD): δ 6.98 (s, 2H), 6.56 (d, *J* = 8.0 Hz, 1H), 6.33 (d, *J* = 2.0 Hz, 1H), 6.31 (s, 2H), 6.22 (dd, *J* = 8.0, 2.0 Hz, 1H), 4.25 (s, 2H), 3.21 – 3.07 (m, 2H), 2.56 (s, 6H), 2.51 – 2.41 (m, 2H), 2.31 (s, 3H), 2.04 (s, 3H).

^{13}C NMR (125 MHz, CD_3OD): δ 157.5, 146.0, 144.7, 144.1, 141.1, 135.2, 134.2, 133.1, 131.3, 120.8, 116.5, 116.3, 111.82, 108.1, 50.8, 47.9, 33.8, 23.2, 21.1, 8.5.

HRMS (ESI) *m/z* calcd. for $C_{17}H_{20}ClN_2O$ $(M+Na)^+$: 494.1608, found: 494.1589.

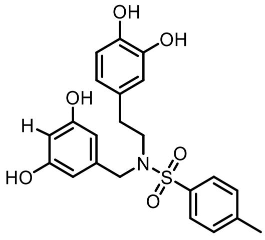

1ar

The **1ar** was obtained in 65% (0.51 g), colorless foam solid (silica gel flash chromatography: DCM: Acetone = 20:1). R_f = 0.40 (DCM: Acetone = 8:1).

^{1}H NMR (500 MHz, CD_3OD): δ 7.68 (d, *J* = 8.0 Hz, 2H), 7.35 (d, *J* = 5.5 Hz, 2H), 6.60 (d, *J* =

8.0 Hz, 1H), 6.42 (s, 1H), 6.31 (s, 2H), 6.27 (dd, J = 8.0, 2.0 Hz, 1H), 6.23 (s, 1H), 4.17 (s, 2H), 3.25 – 3.14 (m, 2H), 2.50 – 2.38 (m, 5H).

^{13}C NMR (125 MHz, CD_3OD): δ 159.8, 146.2, 144.9, 144.8, 140.1, 138.4, 131.4, 130.9, 128.2, 120.9, 116.7, 116.4, 107.9, 102.9, 52.9, 50.8, 35.3, 21.5.

HRMS (ESI) *m/z* calcd. for $C_{17}H_{20}ClN_2O$ $(M+Na)^+$: 452.1138, found: 452.1135.

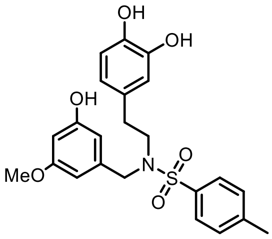

1as

The **1as** was obtained in 30% (0.6 g), colorless foam solid (silica gel flash chromatography: DCM: Acetone = 20:1). R_f = 0.60 (DCM: Acetone = 8:1).

^{1}H NMR (500 MHz, CD_3OD): δ 7.68 (d, J = 8.0 Hz, 2H), 7.35 (d, J = 8.0 Hz, 2H), 6.60 (d, J = 8.0 Hz, 1H), 6.48 – 6.34 (m, 2H), 6.33 – 6.21 (m, 3H), 4.88 (s, 3H), 4.20 (s, 2H), 3.68 (s, 3H), 3.28 – 3.13 (m, 2H), 2.48 – 2.42 (m, 2H), 2.41 (s, 3H).

^{13}C NMR (125 MHz, CD_3OD): δ 162.5, 159.8, 146.2, 144.9, 144.8, 140.2, 138.5, 131.4, 130.9, 128.2, 120.9, 116.7, 116.36, 109.1, 106.1, 101.8, 55.6, 52.9, 50.7, 35.4, 21.5.

HRMS (ESI) *m/z* calcd. for $C_{23}H_{25}NO_6S$ $(M+Na)^+$: 466.1295, found: 466.1293

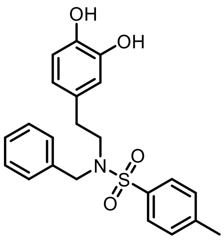

1ba

The **1ba** was obtained as colorless foam solid (silica gel flash chromatography: PE: EA = 5:1). R_f = 0.50 (PE: EA = 1:1).

^{1}H NMR (400 MHz, $CDCl_3$):δ 7.67 (d, J = 7.9 Hz, 2H), 7.23 (m, 7H), 6.68 (d, J = 8.0 Hz, 1H), 6.48 (s, 1H), 6.29 (d, J = 7.8 Hz, 1H), 6.15 (d, J = 34.6 Hz, 2H), 4.27 (s, 2H), 3.34 – 3.04 (m, 2H), 2.51 – 2.41 (m, 2H), 2.38 (s, 3H).

^{13}C NMR (100 MHz, $CDCl_3$):δ 143.6, 142.2, 136.4, 135.9, 130.9, 129.8, 128.6, 128.3, 127.9, 127.0,

120.8, 115.6, 115.3, 52.1, 49.5, 34.3, 21.4.

HRMS (ESI) *m/z* calcd. for $C_{22}H_{23}NO_4S$ $(M+Na)^+$: 420.1348, found: 420.1346

4. Preparation of 1at:

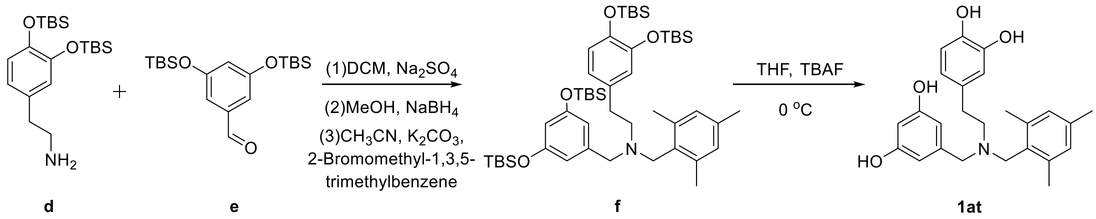

Supplementary Fig. 3: Procedure for **1at** preparation

Procedure for **1at** preparation (**Supplementary Fig. 3**): Under argon atmosphere, compound **d** (1.0 equiv) was dissolved in dry DCM at room temperature, Na_2SO_4 (5 equiv) and substituted benzaldehyde **e** (1.0 equiv) was added subsequently. The resulted solution was stirred at room temperature for 4 hours, after which the system was filtered to remove sodium sulfate and concentrate under reduced pressure, then cooled to 0 ℃, the residue dissolved in MeOH, $NaBH_4$ (1.0 equiv) was added portionwise and the reaction mixture was stirred for additional 4 hours at room temperature. The reaction was quenched by addition of a saturated solution of H_2O (10 mL) then concentrated under vacuum and the residue was extracted with EtOAc (3 × 150 mL). The combined organic layer was wash with brine, dried over Na_2SO_4 and concentrated under vacuum. Without purification, the obtained residue was dissolved in anhydrous CH_3CN in a dried round bottom flask under argon atmosphere. K_2CO_3 (1.5 eq) and substituted benzyl bromide (1.1eq) was added successively under argon atmosphere at r.t. The reaction mixture was stirred for 1hours at 50 ℃. Then the reaction was quenched by addition of H_2O and the reaction mixture was extracted with EtOAc (3 ×150 mL). The combined organic layer was washed with brine, dried over Na_2SO_4 and concentrated under vacuum. Without purification, under argon atmosphere, **f** (1.0 eq) was dissolved in dry THF and TBAF (6 eq) was added successively under argon atmosphere at 0 ℃, stirred for 2 hours. The reaction was quenched by addition of H_2O (50 mL) then the residue was extracted with EtOAc (3 ×100 mL). The combined organic layer was wash with brine, dried over Na_2SO_4 and concentrated under vacuum, and chromatographied on silica gel (DCM: Acetone = 20:1 as eluent) to give **1at**.

The **1at** was obtained in 40% (0.7 g), colorless foam solid (silica gel flash chromatography: DCM:

Acetone = 20:1). R_f = 0.30 (DCM: Acetone = 8:1).

^{1}H NMR (500 MHz, CD_3OD): δ 6.77 (s, 2H), 6.62 (d, *J* = 8.0 Hz, 1H), 6.48 (d, *J* = 2.0 Hz, 1H), 6.35 (dd, *J* = 8.0, 2.0 Hz, 1H), 6.29 (d, *J* = 2.0 Hz, 1H), 6.17 (s, 1H), 5.07 (s, 2H), 3.57 (s, 2H), 3.35 (s, 2H), 2.64 – 2.57 (m, 2H), 2.57 – 2.48 (m, 2H), 2.31 (s, 6H), 2.20 (s, 3H).

^{13}C NMR (125 MHz, CD_3OD): δ 158.9, 145.8, 144.0, 143.8, 139.0, 137.1, 133.7, 133.5, 129.8, 120.9, 116.7, 116.2, 108.6, 101.9, 59.1, 59.0, 56.7, 53.5, 33.8, 21.0, 20.7.

HRMS (ESI) *m/z* calcd. for $C_{25}H_{29}NO_4$ $(M+H)^+$: 408.2169, found: 408.2169

5. Preparation of 1au, 1av, 1aw, 1ax:

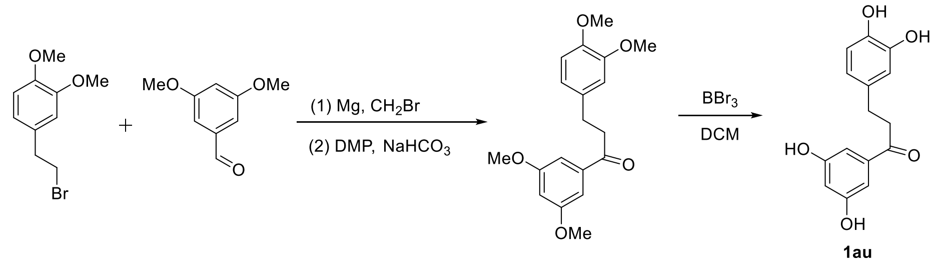

Supplementary Fig. 4: Procedure for **1au** preparation

Procedure for **1au** preparation (**Supplementary Fig. 4**): Under argon atmosphere, bromide (1.0 equiv) was dissolved in dry THF at room temperature, Mg (1.5 equiv) and I_2 (0.1 equiv) was added subsequently. The resulted solution was slowly rises to 60 °C, after stirred for 0.5 hours, then cooled to 0 °C, benzaldehyde (0.8 equiv) was added. The reaction mixture was stirred for additional 0.5 hours at room temperature. The reaction was quenched by addition of a saturated solution of NH_4Cl (10 mL) then the residue was extracted with EtOAc (3 × 30 mL). The combined organic layer was wash with brine, dried over Na_2SO_4 and concentrated under vacuum, chromatographied on silica gel (petroleum ether/ethyl acetate = 2:1 as eluent) to give hydroxy compound.

Next, the obtained hydroxy compound (1 equiv)) was dissolved in anhydrous DCM in a dried round bottom flask under argon atmosphere, $NaHCO_3$ (3 equiv) and DMP (1.5 equiv) was added successively at 0 °C. The reaction mixture was stirred for 1 hour. Then the reaction was quenched by addition of $NaHCO_3$ and the reaction mixture was extracted with DCM (3 × 30 mL). The combined organic layer was washed with brine, dried over Na_2SO_4 and concentrated under vacuum, and chromatographied on silica gel (petroleum ether/ethyl acetate = 4:1 as eluent) to give

ketone.

Under argon atmosphere, ketone (1.0 equiv) was dissolved in dry DCM and BBr_3 (6 equiv) was added successively under argon atmosphere at 0 ℃, stirred for 12 hours. The reaction was quenched by addition of MeOH (20 mL), then concentrated under vacuum, and the residue was extracted with EtOAc (3 × 50 mL). The combined organic layer was wash with brine, dried over Na_2SO_4 and concentrated under vacuum, and chromatographied on silica gel (DCM: Acetone = 20:1 as eluent) to give **1au**.

The **1au** was obtained as colorless foam solid (silica gel flash chromatography: DCM: Acetone = 20:1). R_f = 0.30 (DCM: Acetone = 8:1).

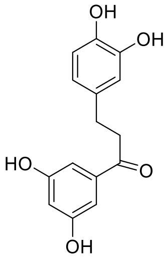

1au

^{1}H NMR (400 MHz, CD_3OD) δ 6.87 (d, *J* = 2.2 Hz, 2H), 6.70 – 6.63 (m, 2H), 6.54 (dd, *J* = 8.0, 2.0 Hz, 1H), 6.47 (t, *J* = 2.2 Hz, 1H), 4.90 (s, 4H), 3.15 (t, *J* = 8.0 Hz, 2H), 2.82 (t, *J* = 8.0 Hz, 2H).

^{13}C NMR (100 MHz, CD_3OD) δ 202.0, 160.0, 146.1, 144.5, 140.2, 134.2, 120.6, 116.5, 116.3, 108.3, 107.4, 41.9, 30.9.

HRMS (ESI) *m/z* calcd. for $C_{15}H_{14}O_5$ $(M-H)^-$: 273.0768, found: 273.0766

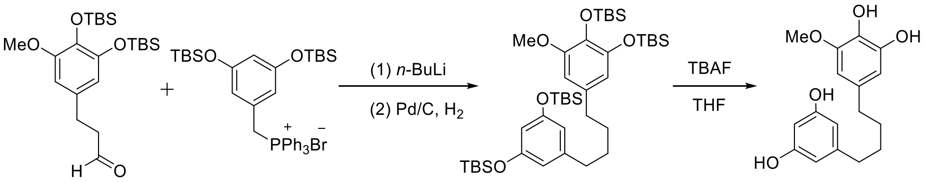

1av

Supplementary Fig. 5: Procedure for **1av** preparation

Procedure for **1av** preparation (**Supplementary Fig. 5**): Under argon atmosphere, witting reagent (1.2 equiv) was dissolved in dry THF at 0 ℃, *n*-BuLi (1.2 equiv) was added subsequently. The resulted solution was stirred for 0.5 hours, then aldehyde (1.0 equiv) was added and the

reaction mixture was stirred for additional 4 hours at room temperature. The reaction was quenched by addition of a saturated solution of NH_4Cl (10 mL) then the system was extracted with EtOAc (3 × 100 mL). The combined organic layer was wash with brine, dried over Na_2SO_4 and concentrated under vacuum.

Without purification, the obtained residue was dissolved in MeOH, Pd/C (10%) was added. The the reaction system was filled with hydrogen atmosphere and was stirred for 8 hours at room temperature. Then the reaction system was filtered through celite. Without purification, the obtained residue was dissolved in dry THF and TBAF (6 equiv) was added successively at 0 ℃, stirred for 1 hours. The reaction was quenched by addition of H_2O (50 mL) then the residue was extracted with EtOAc (3 × 100 mL). The combined organic layer was wash with brine, dried over Na_2SO_4 and concentrated under vacuum, and chromatographied on silica gel (DCM: Acetone = 20:1 as eluent) to give **1av** .

The **1av** was obtained as colorless foam solid (silica gel flash chromatography: DCM: Acetone = 20:1). R_f = 0.30 (DCM: Acetone = 8:1).

^{1}H NMR (400 MHz, CD_3OD) δ 6.28 (dd, *J* = 6.4, 1.8 Hz, 2H), 6.14 (d, *J* = 2.0 Hz, 2H), 6.10 (t, *J* = 2.0 Hz, 1H), 4.89 (s, 2H), 3.77 (s, 3H), 2.45 (d, *J* = 2.4 Hz, 4H), 1.56 (t, *J* = 3.0 Hz, 4H).

^{13}C NMR (100 MHz, CD_3OD) δ 159.2, 149.4, 146.3, 146.2, 134.8, 132.9, 109.8, 108.0, 104.7, 100.9, 56.5, 36.8, 36.5, 32.3, 31.8.

HRMS (ESI) *m/z* calcd. for $C_{17}H_{20}O_5$ $(M-H)^-$: 303,1238, found: 304.1237

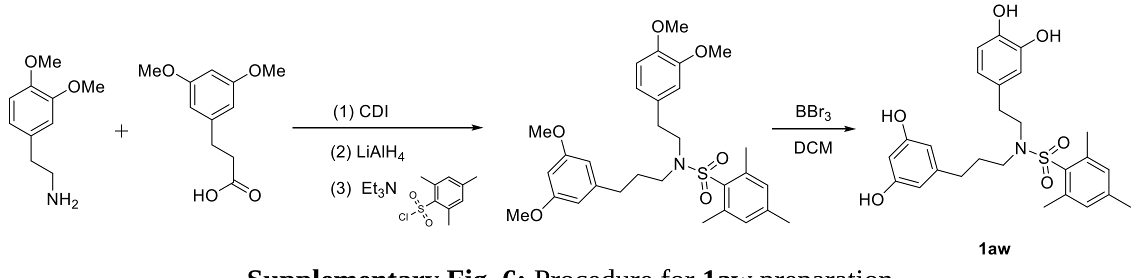

Supplementary Fig. 6: Procedure for **1aw** preparation

Procedure for **1aw** preparation (**Supplementary Fig. 6**): Under argon atmosphere, carboxylic acid (1.0 equiv) was dissolved in dry DCM at 0 ℃, CDI (1.2 equiv) was added subsequently. The resulted solution was stirred for 0.5 hours, then amine (1.0 equiv) was added and the reaction mixture was stirred for additional 4 hours at room temperature. The reaction was quenched by addition of a saturated solution of NH_4Cl (10 mL) then the system was extracted with DCM (3 × 50 mL). The combined organic layer was wash with brine, dried over Na_2SO_4 and concentrated

under vacuum.

Without purification, the obtained residue was dissolved in THF, $LiAlH_4$ (5 equiv) was added at 0 ℃. then the system was stirred for 8 h at 60 ℃. Then the reaction system was quenched by addition of H_2O (10 mL) and extracted with EtOAc (3 × 50 mL). The combined organic layer was wash with brine, dried over Na_2SO_4 and concentrated under vacuum. Without purification, the obtained residue was dissolved in dry DCM, Et_3N (2 equiv) and sulfonyl chloride (1.2 equiv) were added successively under argon atmosphere at 0 ℃, stirred for 2 hours. The reaction was quenched by addition of $NaHCO_3$ and the reaction mixture was extracted with DCM (3 × 30 mL). The combined organic layer was washed with brine, dried over Na_2SO_4 and concentrated under vacuum, and chromatographied on silica gel (petroleum ether/ethyl acetate = 5:1 as eluent).

The obtained above compound was dissolved in dry DCM and BBr_3 (6 equiv) was added successively under argon atmosphere at 0 ℃, stirred for 12 hours. The reaction was quenched by addition of MeOH (20 mL), then concentrated under vacuum, and the residue was extracted with EtOAc (3 × 50 mL). The combined organic layer was wash with brine, dried over Na_2SO_4 and concentrated under vacuum, and chromatographied on silica gel (DCM: Acetone = 20:1 as eluent) to give **1aw**.

The **1aw** was obtained as colorless foam solid (silica gel flash chromatography: DCM: Acetone = 20:1). R_f = 0.30 (DCM: Acetone = 8:1).

^{1}H NMR (400 MHz, CD_3OD) δ 6.81 (s, 2H), 6.50 (d, *J* = 8.0 Hz, 1H), 6.33 (d, *J* = 2.0 Hz, 1H), 6.21 (dd, *J* = 8.0, 2.0 Hz, 1H), 6.01 (t, *J* = 2.0 Hz, 1H), 5.93 (d, *J* = 2.0 Hz, 2H), 4.78 (s, 3H), 3.23 – 3.14 (m, 2H), 3.09 – 3.00 (m, 2H), 2.48 – 2.39 (m, 2H), 2.34 (s, 6H), 2.22 (t, *J* = 7.0 Hz, 2H), 2.17 (s, 3H), 1.67 – 1.50 (m, 2H).

^{13}C NMR (100 MHz, CD_3OD) δ 159.4, 146.1, 144.8, 144.7, 144.2, 141.1, 133.9, 133.0, 131.4, 120.9, 116.6, 116.4, 107.9, 101.3, 48.7, 46.3, 34.5, 33.9, 29.7, 23.0, 21.1.

HRMS (ESI) *m/z* calcd. for $C_{26}H_{31}NO_6S$ $(M-H)^-$: 484.1799, found: 484.1798

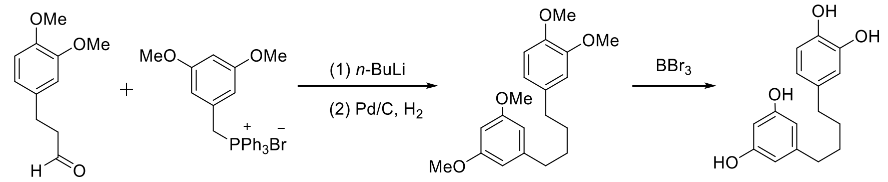

1ax

Supplementary Fig. 7: Procedure for **1ax** preparation

Procedure for **1ax** preparation (**Supplementary Fig. 7**): Under argon atmosphere, witting reagent (1.2 equiv) was dissolved in dry THF at 0 ℃, *n*-BuLi (1.2 equiv) was added subsequently. The resulted solution was stirred for 0.5 hours, then aldehyde (1.0 equiv) was added and the reaction mixture was stirred for additional 4 hours at room temperature. The reaction was quenched by addition of a saturated solution of NH_4Cl (10 mL) then the system was extracted with EtOAc (3 × 100 mL). The combined organic layer was wash with brine, dried over Na_2SO_4 and concentrated under vacuum.

Without purification, the obtained residue was dissolved in MeOH, Pd/C (10%) was added. The the reaction system was filled with hydrogen atmosphere and was stirred for 8 hours at room temperature. Then the reaction system was filtered through celite. Without purification, the obtained residue was dissolved in dry DCM and BBr_3 (6 equiv) was added successively under argon atmosphere at 0 ℃, stirred for 12 hours. The reaction was quenched by addition of MeOH (20 mL), then concentrated under vacuum, and the residue was extracted with EtOAc (3 ×50 mL). The combined organic layer was wash with brine, dried over Na_2SO_4 and concentrated under vacuum, and chromatographied on silica gel (DCM: Acetone = 20:1 as eluent) to give 1ax.

The **1ax** was obtained as colorless foam solid (silica gel flash chromatography: DCM: Acetone = 20:1). R_f = 0.30 (DCM: Acetone = 8:1).

^{1}H NMR (400 MHz, CD_3OD) δ 6.66 (d, *J* = 8.0 Hz, 1H), 6.60 (d, *J* = 2.0 Hz, 1H), 6.47 (dd, *J* = 8.0, 2.0 Hz, 1H), 6.14 (d, *J* = 2.0 Hz, 2H), 6.10 (t, *J* = 2.0 Hz, 1H), 4.90 (s, 1H), 2.44 (d, *J* = 3.0 Hz, 4H), 1.62 – 1.41 (m, 4H).

^{13}C NMR (100 MHz, CD_3OD) δ 159.1, 146.3, 145.8, 143.9, 135.6, 120.7, 116.5, 116.2, 108.0, 100.9, 49.5, 49.2, 49.0, 48.8, 48.6, 36.8, 36.0, 32.4, 31.9.

HRMS (ESI) *m/z* calcd. for $C_{16}H_{18}O_4$ $(M-H)^-$: 273.1132, found: 273.1132

6. Preparation of 1az:

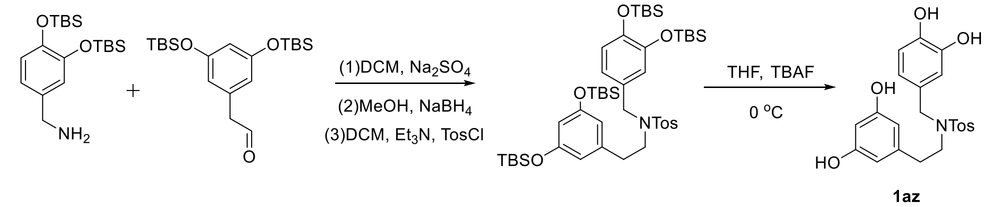

1az

Supplementary Fig. 8: Procedure for **1az** preparation

Procedure for **1az** preparation (**Supplementary Fig. 8**): Under argon atmosphere, amine (1.0

equiv) was dissolved in dry DCM at room temperature, Na_2SO_4 (5 equiv) and substituted benzaldehyde (1.0 equiv) was added subsequently. The resulted solution was stirred at room temperature for 4 hours, after which the system was filtered to remove sodium sulfate and concentrate under reduced pressure, then cooled to 0 ℃, the residue dissolved in MeOH, $NaBH_4$ (1.0 equiv) was added portionwise and the reaction mixture was stirred for additional 4 hours at room temperature. The reaction was quenched by addition of a saturated solution of H_2O (10 mL) then concentrated under vacuum and the residue was extracted with EtOAc. The combined organic layer was wash with brine, dried over Na_2SO_4 and concentrated under vacuum. Without purification, the obtained residue was dissolved in anhydrous DCM in a dried round bottom flask under argon atmosphere. Et_3N (1.5 eq) and TosCl (1.1eq) was added successively under argon atmosphere at r.t. The reaction mixture was stirred for 1 hour. Then the reaction was quenched by addition of $NaHCO_3$ and the reaction mixture was extracted with DCM. The combined organic layer was washed with brine, dried over Na_2SO_4 and concentrated under vacuum. Without purification, under argon atmosphere, the above crude products was dissolved in dry THF and TBAF (6 eq) was added successively under argon atmosphere at 0 ℃, stirred for 2 hours. The reaction was quenched by addition of H_2O (50 mL) then the residue was extracted with EtOAc. The combined organic layer was wash with brine, dried over Na_2SO_4 and concentrated under vacuum, and chromatographied on silica gel (DCM: Acetone = 20:1 as eluent) to give 1az.

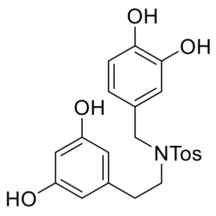

1az

^{1}H NMR (400 MHz, CD_3OD) δ 7.70 (d, *J* = 7.9 Hz, 2H), 7.38 (d, *J* = 7.9 Hz, 2H), 6.80 (s, 1H), 6.73 (d, *J* = 8.0 Hz, 1H), 6.61 (d, *J* = 8.0 Hz, 1H), 6.06 (s, 1H), 5.91 (s, 2H), 4.18 (s, 2H), 3.25 – 3.10 (m, 2H), 2.43 (s, 3H), 2.41 – 2.33 (m, 2H).

^{13}C NMR (100 MHz, CD_3OD) δ 159.54, 146.58, 146.30, 144.91, 142.20, 138.43, 130.91, 128.97, 128.20, 121.42, 116.83, 116.21, 108.05, 101.65, 52.70, 50.10, 36.20, 21.47.

HRMS (ESI) *m/z* calcd. for $C_{17}H_{20}ClN_2O$ $(M+Na)^+$: 452.1138, found: 452.1137.

7. Preparation of racemic samples:

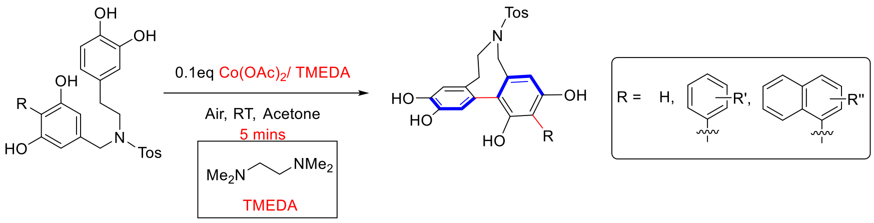

Supplementary Fig. 9: General procedure for racemic samples preparation

General procedure for racemic samples preparation (**Supplementary Fig. 9**): Unless otherwise noted, reactions were performed: $Co(OAc)_2$ (1.7 mg, 10 mol%) and TMEDA (1.5 µl, 10 mol%) were added to acetone (1.0 mL) at room temperature, and stirred for 30 mins, then substrate (0.05 mmol) were added, the reaction was stirred for 5-10 mins. The reaction was quenched by addition of 20 µl HCl (2 mol/L in EtOAc). Then the product purifiction by flash chromatography (DCM: Acetone= 8:1) on silica gel directly and the racemic samples were obtained.

8. Preparation of chiral 2:

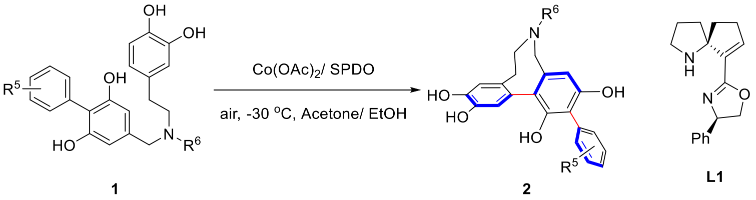

Supplementary Fig. 10: General procedure for **2** preparation

General procedure for **2** preparation (**Supplementary Fig. 10**): Unless otherwise noted, reactions were performed: $Co(OAc)_2$ (1.7 mg, 10 mol%) and **L1** (3.2 mg, 12 mol%) were added to acetone (3.0 mL) at room temperature, and stirred for 10 mins, then DABCO (4.48mg, 40 mol%) was added and stirred for 1.5 hours, then 1 mL EtOH was added to the system, 10 mins later the system was drops to -30 °C, and substrates (0.1 mmol) were added, the reaction was stirred for 5-10 hours. The reaction was quenched by addition of 40 µl HCl (2 mol/L in EtOAc). Then the product purifiction by flash chromatography (DCM: Acetone= 8:1) on silica gel directly.

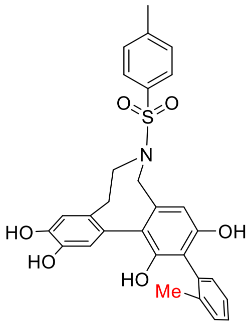

2a

According to the procedure, **2a** was obtained using **1a** (57.5 mg, 0.1 mmol) in 95% yield (54.4mg), light gray foam solid, 5/1 d r and 95% ee, (silica gel flash chromatography: DCM/ acetone = 20:1). R_f = 0.40 (DCM/acetone = 10:1).

^{1}H NMR (500 MHz, CD_3OD): δ 7.64 (d, *J* = 8.0 Hz, 2H), 7.30 (dd, *J* = 19.5, 8.0 Hz, 4H), 7.25 – 7.10 (m, 4H), 6.90 (s, 1H), 6.72 (s, 1H), 6.65 (s, 1H), 4.62 (d, *J* = 14.0 Hz, 1H), 3.99 (dd, *J* = 12.0, 7.0 Hz, 1H), 2.97 (d, *J* = 14.0 Hz, 1H), 2.73 (dd, *J* = 14.0, 7.0 Hz, 1H), 2.47 (d, *J* = 11.5 Hz, 1H), 2.44 – 2.34 (m, 4H), 2.21 (s, 3H).

^{13}C NMR (125 MHz, CD_3OD): δ 155.5, 152.4, 146.5, 144.8, 144.5, 139.3, 138.4, 137.2, 135.1, 133.8, 132.3, 132.2, 130.9, 130.7, 128.5, 128.2, 127.5, 126.6, 120.4, 118.5, 117.5, 117.3, 110.1, 51.0, 50.6, 34.0, 21.4, 20.1.

HRMS (ESI) *m/z* calcd. for $C_{29}H_{27}NO_6S$ $(M+Na)^+$: 540.1451, found: 540.1447

Enantiomeric excess of 2**a** is determined by HPLC (Chiralpak IB, Hexane/Isopropanol = 60/40, flow rate = 1.0 mL/min, 220 nm): major isomer: tr = 9.73 min; minor isomer: tr = 18.25. $[\alpha]^{20}{}_D$ = -209.53 (c = 1.0, MeOH).

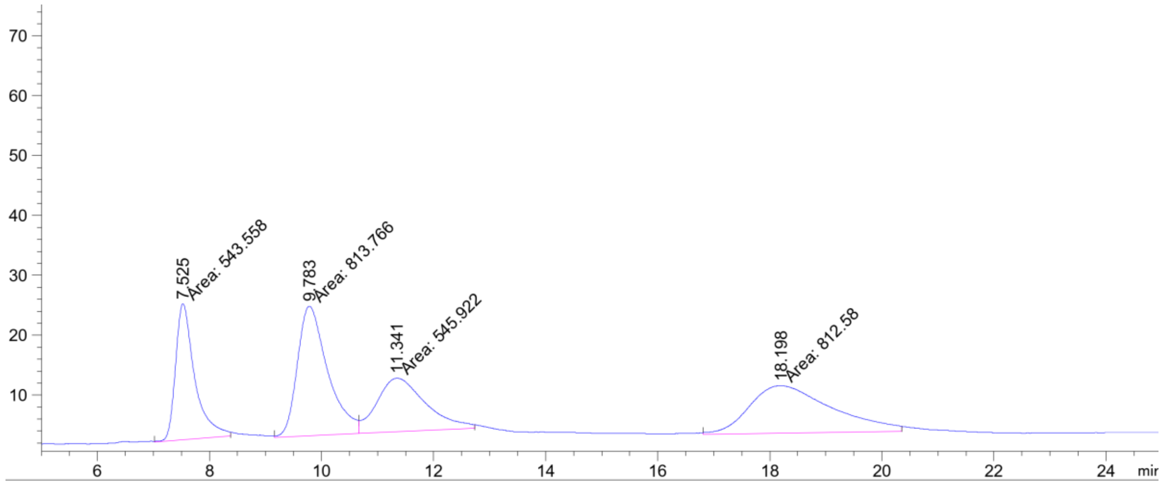

Signal 1: DAD1 A, Sig=220,4 Ref=off

Peak #	RetTime [min]	Type	Width [min]	Area [mAU*s]	Height [mAU]	Area %
1	7.525	MM	0.3983	543.55847	22.74740	20.0145
2	9.783	MF	0.6267	813.76630	21.64021	29.9639
3	11.341	FM	1.0155	545.92181	8.95960	20.1015
4	18.198	MM	1.7082	812.57977	7.92832	29.9202

Totals : 2715.82635 61.27553

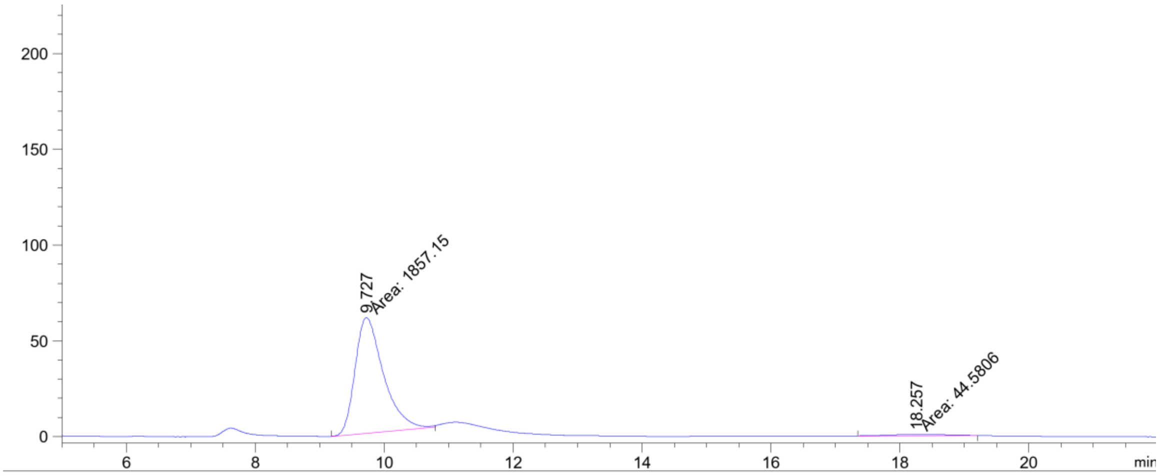

Signal 1: DAD1 A, Sig=220,4 Ref=off

Peak #	RetTime [min]	Type	Width [min]	Area [mAU*s]	Height [mAU]	Area %
1	9.727	MM	0.5120	1857.15332	60.45414	97.6558
2	18.257	MM	1.0836	44.58060	6.85710e-1	2.3442

Totals : 1901.73392 61.13985

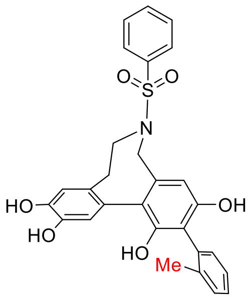

2b

According to the procedure, **2b** was obtained using **1b** (50.5 mg, 0.1 mmol) in 96% yield (48.3mg), light gray foam solid, 5/1 d r and 92% ee, (silica gel flash chromatography: DCM/ acetone = 20:1). R_f = 0.40 (DCM/acetone = 10:1).

^{1}H NMR (500 MHz, CD_3OD): δ 7.77 (d, *J* = 8.0 Hz, 2H), 7.62 – 7.47 (m, 4H), 7.32 – 7.07 (m, 5H), 6.89 (s, 1H), 6.68 (s, 1H), 6.63 (s, 1H), 4.64 (d, *J* = 13.5 Hz, 1H), 4.01 (dd, *J* = 12.0, 6.5 Hz, 1H), 2.98 (d, *J* = 14.0 Hz, 1H), 2.73 (dd, *J* = 14.5, 7.0 Hz, 1H), 2.49 (t, *J* = 11.5 Hz, 1H), 2.36 (dd, *J* = 20.0, 9.0 Hz, 1H), 2.19 (s, 3H).

^{13}C NMR (125 MHz, CD_3OD): δ 155.6, 152.5, 146.5, 144.5, 140.2, 139.3, 138.3, 135.2, 133.7, 132.3, 130.8, 130.3, 128.5, 128.2, 127.5, 126.6, 120.5, 118.5, 117.6, 117.3, 110.0, 51.1, 50.6, 34.1, 20.1.

HRMS (ESI) *m/z* calcd. for $C_{28}H_{25}NO_6S$ $(M+Na)^+$: 526.1295, found: 526.1287

Enantiomeric excess of **2b** is determined by HPLC (Chiralpak IB, Hexane/Isopropanol = 60/40, flow rate = 1.0 mL/min, 220 nm): major isomer: tr = 10.04min; minor isomer: tr = 19.01 min. $[\alpha]^{20}_D$ = -97.47 (c = 1.0, Acetone).

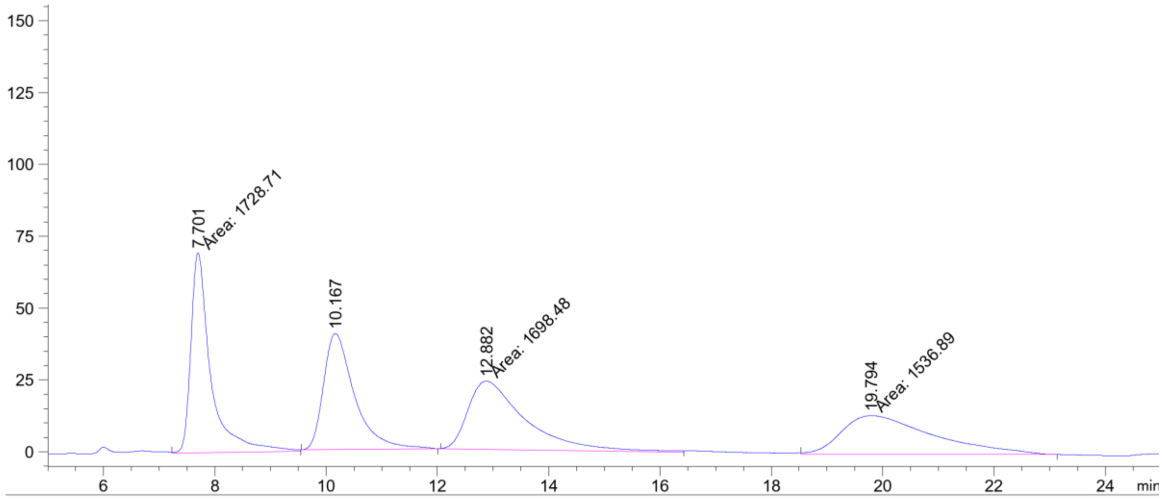

Signal 1: DAD1 A, Sig=220,4 Ref=off

Peak #	RetTime [min]	Type	Width [min]	Area [mAU*s]	Height [mAU]	Area %
1	7.701	MM	0.4141	1728.71411	69.58263	26.5014
2	10.167	BB	0.5740	1559.02161	40.42036	23.9000
3	12.882	MM	1.1906	1698.48145	23.77657	26.0379
4	19.794	MM	1.9031	1536.88904	13.45977	23.5607

Totals : 6523.10620 147.23933

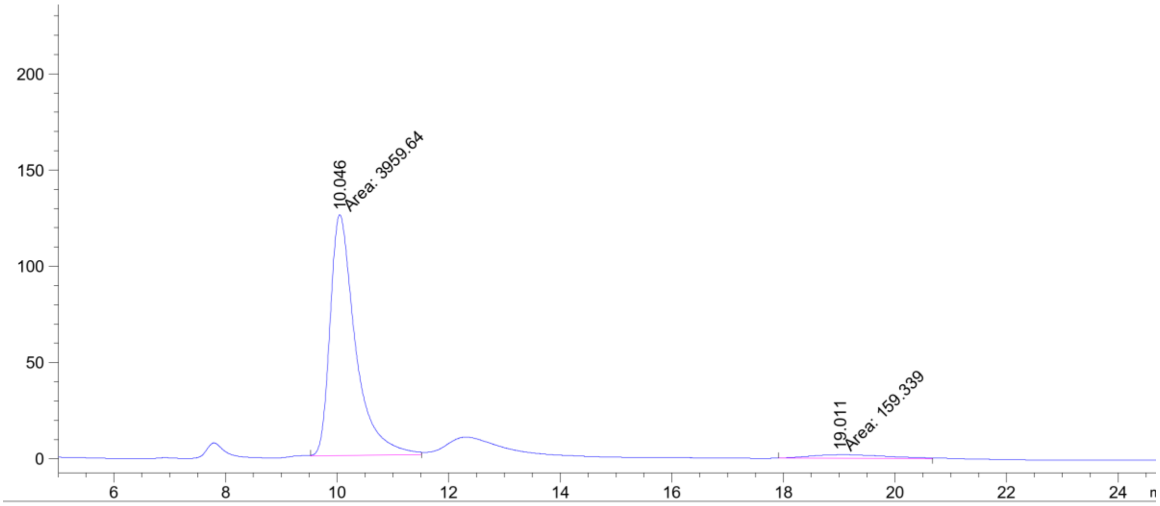

Signal 1: DAD1 A, Sig=220,4 Ref=off

Peak #	RetTime [min]	Type	Width [min]	Area [mAU*s]	Height [mAU]	Area %
1	10.046	MM	0.5272	3959.63599	125.18851	96.1316
2	19.011	MM	1.5015	159.33928	1.76863	3.8684

Totals : 4118.97527 126.95714

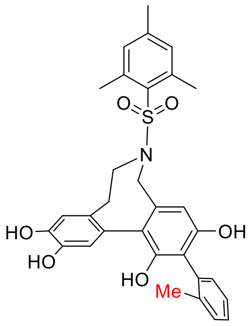

2c

According to the procedure, **2c** was obtained using **1c** (54.7 mg, 0.1 mmol) in 87% yield (47.4mg), light gray foam solid, 10/1 d r and 92% ee, (silica gel flash chromatography: DCM/ acetone = 20:1). R_f = 0.40 (DCM/acetone = 10:1).

^{1}H NMR (500 MHz, CD_3OD): δ 7.31 – 7.11 (m, 5H), 7.03 (s, 2H), 6.74 (s, 1H), 6.70 (s, 1H), 6.61 (s, 1H), 4.70 (d, *J* = 14.0 Hz, 1H), 3.57 (dd, *J* = 13.0, 7.0 Hz, 1H), 3.25 (d, *J* = 14.0 Hz, 1H), 2.75 – 2.62 (m, 2H), 2.55 (d, *J* = 6.0 Hz, 6H), 2.46 (dd, *J* = 14.0, 10.5 Hz, 1H), 2.29 (s, 3H), 2.20 (s, 3H).

^{13}C NMR (125 MHz, CD_3OD): δ 155.3, 152.4, 146.6, 144.6, 144.3, 141.5, 139.3, 138.5, 135.1, 134.1, 133.9, 133.2, 132.2, 130.9, 128.5, 127.5, 126.7, 120.7, 118.5, 117.5, 117.3, 110.3, 49.2, 34.1, 23.1, 21.00, 20.1.

HRMS (ESI) *m/z* calcd. for $C_{31}H_{31}NO_6S$ $(M+Na)^+$: 568.1764, found: 568.1758

Enantiomeric excess of **2c** is determined by UPC^2 (CHIRALPAK® OD-3, CO_2/MeOH = 70/30, flow rate = 1.0 mL/min, 220 nm): major isomer: tr = 4.63 min; minor isomer: tr = 7.52 min. $[\alpha]^{20}_D$ = -75.27 (c = 1.0, Acetone).

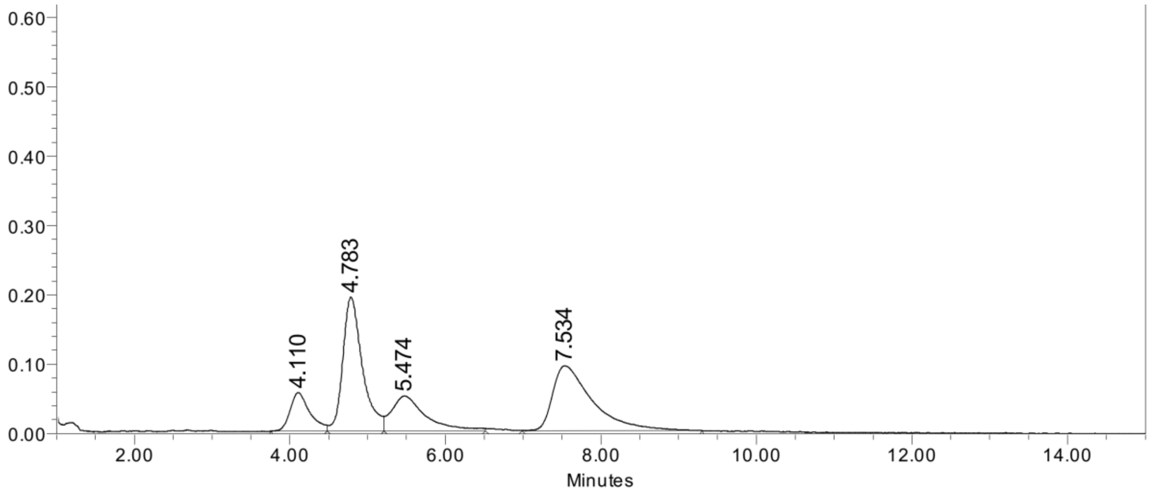

Signal: Sig = 220 nm

Peak	RetTime	Area	Height	Area%
1	4.110	987666	55663	13.40
2	4.783	3406730	193571	36.69
3	5.474	101666	50644	13.81
4	7.534	3352496	93817	36.10

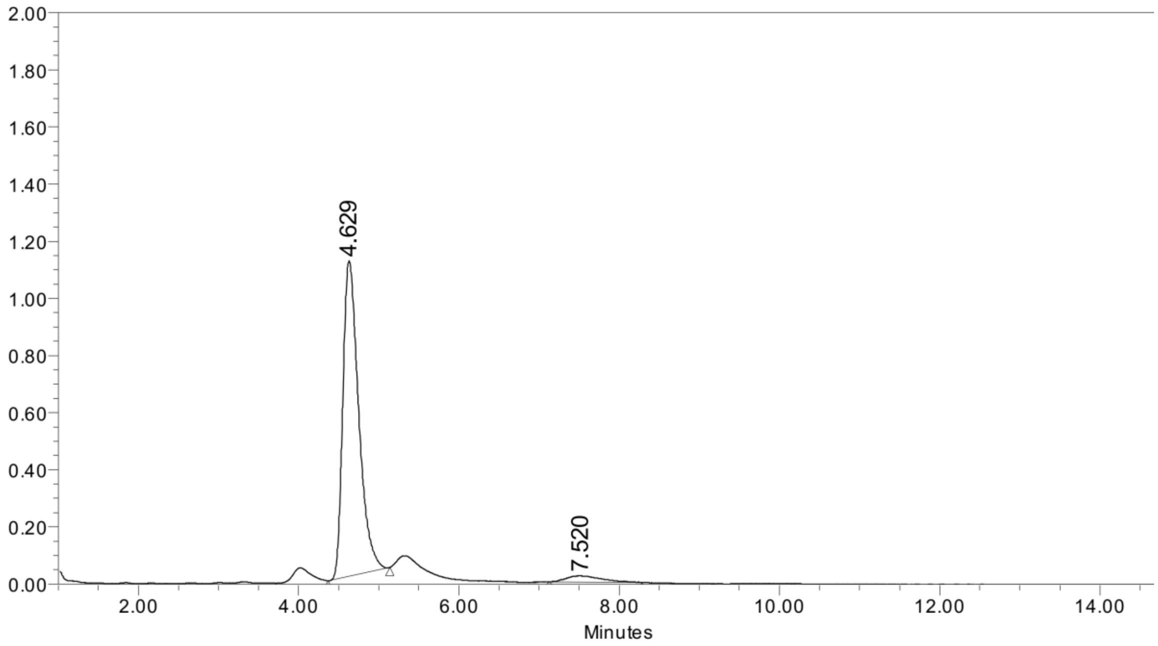

Peak	RetTime	Area	Height	Area%
1	4.629	16177828	1120438	96.14
2	7.520	649243	21479	3.86

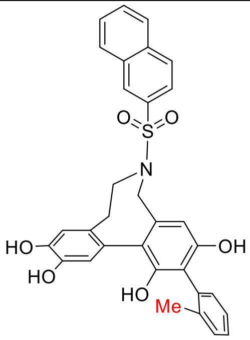

2f

According to the procedure, **2f** was obtained using **1f** (55.5 mg, 0.1 mmol) in 91% yield (50.32mg), light gray foam solid, 9/1 d r and 93% ee, (silica gel flash chromatography: DCM/ acetone = 20:1). R_f = 0.40 (DCM/acetone = 10:1).

^{1}H NMR (500 MHz, $CDCl_3$): δ 8.37 (s, 1H), 8.08 – 7.85 (m, 3H), 7.73 (d, *J* = 8.5 Hz, 1H), 7.60 (dd, *J* = 12.0, 7.5 Hz, 2H), 7.37 – 7.11 (m, 3H), 7.10 – 6.87 (m, 2H), 6.66 (d, *J* = 32.5 Hz, 2H), 4.75 (d, *J* = 14.0 Hz, 1H), 4.18 – 3.99 (m, 1H), 3.07 (d, *J* = 14.0 Hz, 1H), 2.71 (dd, *J* = 14.0, 6.5

Hz, 1H), 2.66 – 2.48 (m, 1H), 2.41 – 2.28 (m, 1H), 2.22 (s, 3H).

^{13}C NMR (125 MHz, $CDCl_3$): δ 155.5, 152.5, 146.5, 144.5, 139.2, 138.1, 137.5, 136.1, 135.2, 133.7, 133.5, 132.3, 130.8, 130.4, 130.3, 129.8, 129.5, 128.9, 128.6, 128.5, 127.5, 126.6, 123.5, 120.5, 118.6, 117.6, 117.3, 110.3, 51.1, 50.7, 34.1, 20.1.

HRMS (ESI) *m/z* calcd. for $C_{32}H_{27}NO_6S$ $(M+Na)^+$: 576.1451, found: 576.1442

Enantiomeric excess of **2f** is determined by HPLC (Chiralpak IB, Hexane/Isopropanol = 60/40, flow rate = 1.0 mL/min, 220 nm): major isomer: tr = 10.20 min; minor isomer: tr = 20.15 min. $[\alpha]^{20}_{D}$ = -278.06 (c = 1.0, Acetone).

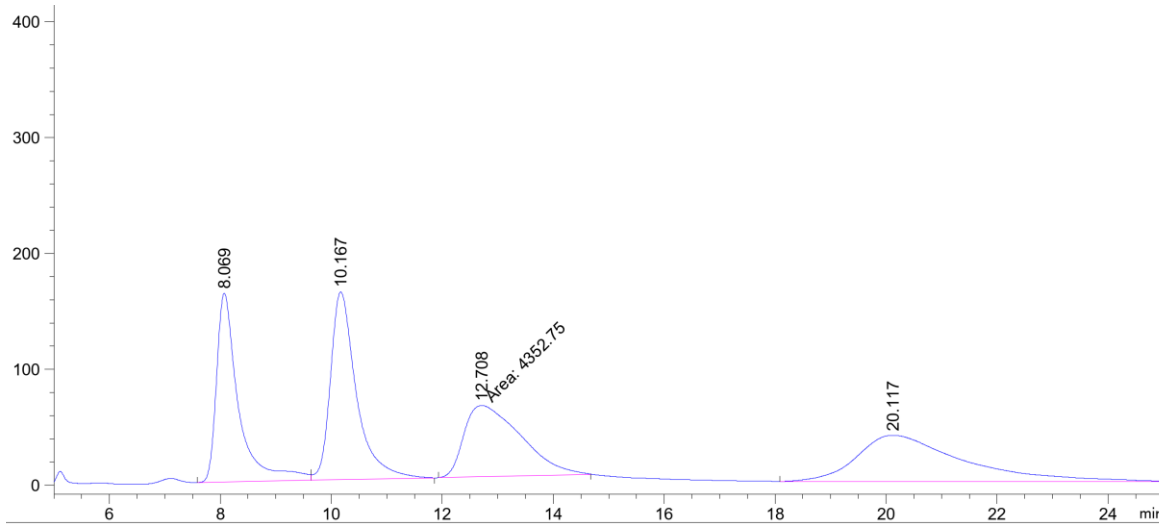

Signal 1: DAD1 A, Sig=220,4 Ref=off

Peak #	RetTime [min]	Type	Width [min]	Area [mAU*s]	Height [mAU]	Area %
1	8.069	BV	0.4003	4503.27246	162.98959	23.1363
2	10.167	VB	0.4852	5270.01367	162.12117	27.0756
3	12.708	MM	1.1803	4352.74805	61.46326	22.3630
4	20.117	BB	1.7354	5338.07031	39.88605	27.4252

Totals : 1.94641e4 426.46008

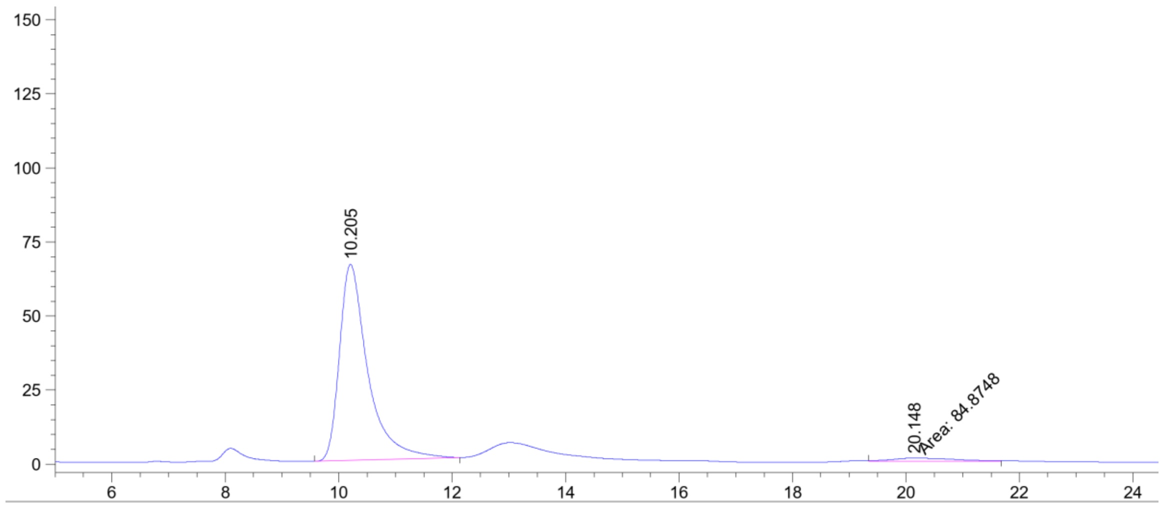

Signal 1: DAD1 A, Sig=220,4 Ref=off

Peak #	RetTime [min]	Type	Width [min]	Area [mAU*s]	Height [mAU]	Area %
1	10.205	BB	0.5141	2304.21826	66.19748	96.4474
2	20.148	MM	1.3350	84.87476	1.05958	3.5526

Totals :				2389.09302	67.25706	

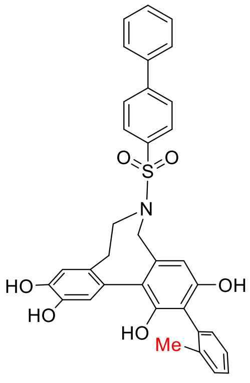

2e

According to the procedure, **2e** was obtained using **1e** (58.1mg, 0.1 mmol) in 97% yield (56.1mg), light gray foam solid, 10/1 dr and 92% ee, (silica gel flash chromatography: DCM/ acetone = 20:1). R_f = 0.40 (DCM/acetone = 10:1).

^{1}H NMR (500 MHz, CD_3OD): δ 7.80 (d, *J* = 8.5 Hz, 2H), 7.72 (d, *J* = 8.5 Hz, 2H), 7.60 (d, *J* = 7.5 Hz, 2H), 7.42 (t, *J* = 7.5 Hz, 2H), 7.36 (d, *J* = 7.5 Hz, 1H), 7.27 (d, *J* = 7.5 Hz, 1H), 7.19 (dd, *J* = 10.5, 4.0 Hz, 2H), 7.12 (dd, *J* = 15.5, 7.0 Hz, 2H), 6.88 (s, 1H), 6.72 (s, 1H), 6.67 (s, 1H), 4.61 (d, *J* = 14.0 Hz, 1H), 4.06 (dd, *J* = 12.0, 7.0 Hz, 1H), 2.98 (d, *J* = 14.0 Hz, 1H), 2.76 (dd, *J* =

14.0, 7.0 Hz, 1H), 2.53 (t, *J* = 11.5 Hz, 1H), 2.40 (dd, *J* = 14.0, 11.0 Hz, 1H), 2.21 (s, 3H).

^{13}C NMR (125 MHz, CD_3OD): δ 155.5, 152.4, 146.5, 146.5, 144.5, 140.4, 139.2, 138.8, 138.2, 135.2, 133.75, 132.3, 130.8, 130.1, 129.5, 128.8, 128.6, 128.5, 128.3, 127.5, 126.6, 120.4, 118.6, 117.5, 117.3, 110.1, 51.12, 50.7, 34.1, 20.1.

HRMS (ESI) *m/z* calcd. for $C_{34}H_{29}NO_6S$ $(M+Na)^+$: 602.1608, found: 602.1597

Enantiomeric excess of **2e** is determined by HPLC (Chiralpak IB, Hexane/Isopropanol = 70/30, flow rate = 1.0 mL/min, 220 nm)): major isomer: tr = 24.28 min; minor isomer: tr = 66.31 min. $[\alpha]^{20}_{D}$ = -313.33 (c = 1.0, Acetone).

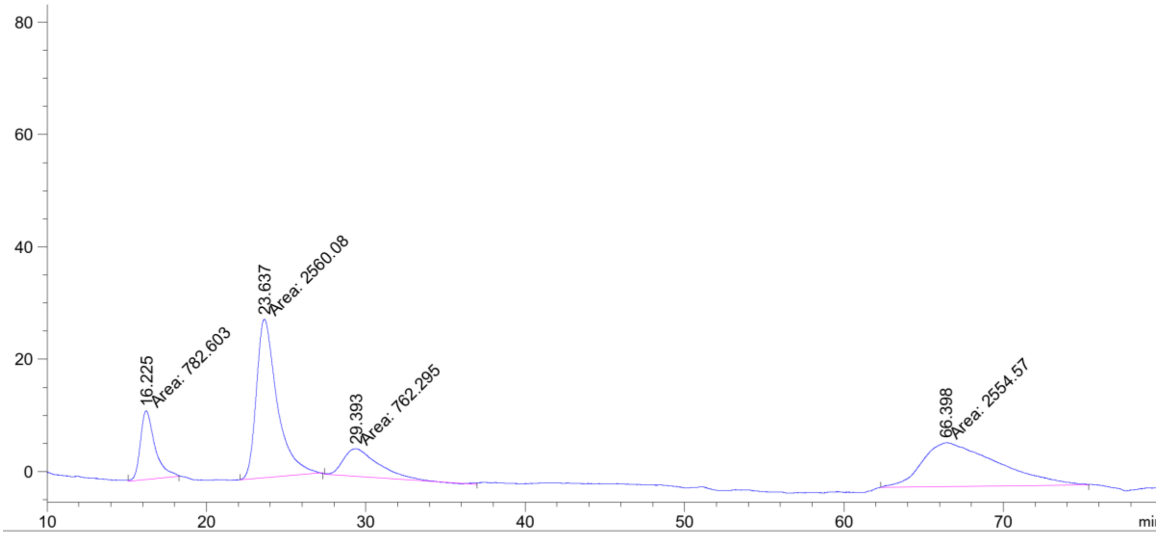

Signal 1: DAD1 A, Sig=220,4 Ref=off

Peak #	RetTime [min]	Type	Width [min]	Area [mAU*s]	Height [mAU]	Area %
1	16.225	MM	1.0655	782.60315	12.24106	11.7516
2	23.637	MM	1.5107	2560.08057	28.24458	38.4423
3	29.393	MM	2.5726	762.29480	4.93856	11.4466
4	66.398	MM	5.4537	2554.56836	7.80685	38.3595
Totals :				6659.54688	53.23106	

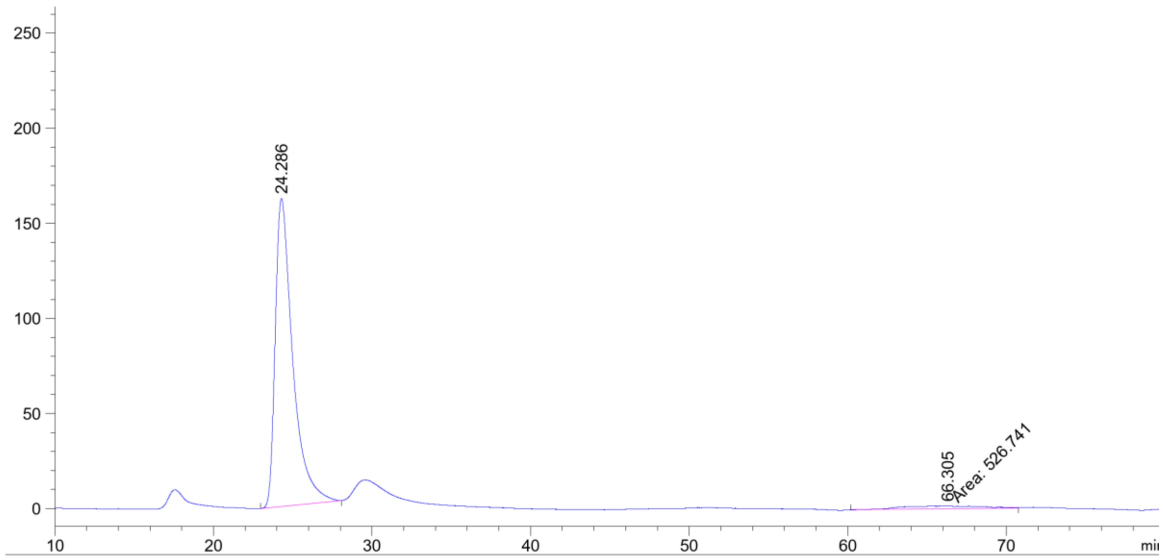

Signal 1: DAD1 A, Sig=220,4 Ref=off

Peak #	RetTime [min]	Type	Width [min]	Area [mAU*s]	Height [mAU]	Area %
1	24.286	BB	1.0916	1.24449e4	162.12091	95.9393
2	66.305	MM	5.8249	526.74109	1.50715	4.0607
Totals :				1.29717e4	163.62806	

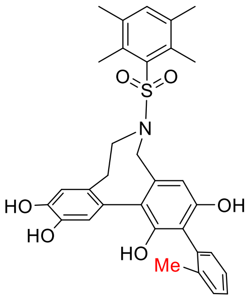

2d

According to the procedure, **2d** was obtained using **1d** (56.1 mg, 0.1 mmol) in 92% yield (51.4mg), light gray foam solid, 10/1 dr, and 94% ee, (silica gel flash chromatography: DCM/ acetone = 20:1). R_f = 0.40 (DCM/acetone = 10:1).

^{1}H NMR (500 MHz, CD_3OD)： δ 7.31 – 7.11 (m, 6H), 6.74 (d, *J* = 4.0 Hz, 1H), 6.69 (d, *J* = 8.5 Hz, 1H), 6.51 (s, 1H), 4.65 (dd, *J* = 15.0, 10.5 Hz, 1H), 3.63 (m, 1H), 3.25 (d, *J* = 14.0 Hz, 1H), 2.77 – 2.63 (m, 2H), 2.55 – 2.45 (m, 8H), 2.27 (s, 6H), 2.20 (s, 3H).

^{13}C NMR (125 MHz, CD_3OD)： δ 155.2, 152.4, 146.6, 144.6, 139.3, 138.4, 137.6, 137.6, 137.5,

137.3, 135.2, 134.1, 132.2, 130.9, 128.6, 127.4, 126.7, 120.7, 118.5, 117.5, 117.3, 110.3, 49.3, 34.0, 21.1, 20.1, 18.0, 17.9.

HRMS (ESI) *m/z* calcd. for $C_{32}H_{33}NO_6S$ $(M+Na)^+$: 582.1921, found: 582.1911

Enantiomeric excess of **2d** is determined by HPLC (Chiralpak IB, Hexane/Isopropanol = 55/45, flow rate = 1.0 mL/min, 220 nm)): major isomer: tr = 6.83 min; minor isomer: tr = 21.02 min. $[\alpha]^{20}_{D}$ = -70.27 (c = 1.0, acetone)

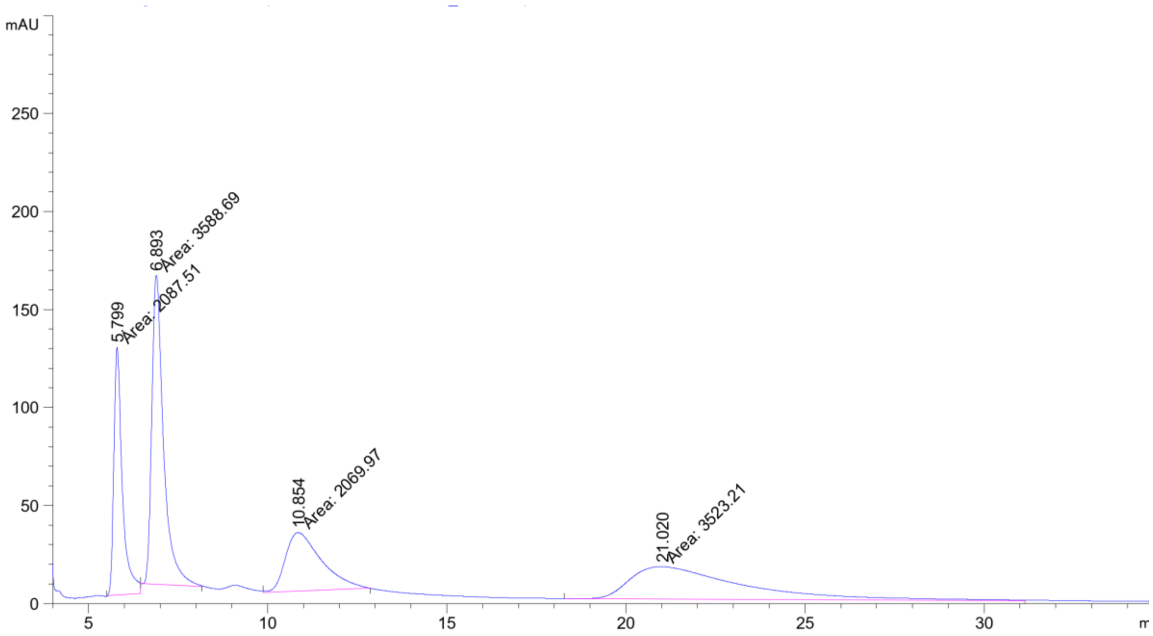

Signal 1: DAD1 A, Sig=220,4 Ref=off

Peak #	RetTime [min]	Type	Width [min]	Area [mAU*s]	Height [mAU]	Area %
1	5.799	MM	0.2756	2087.51245	126.25871	18.5237
2	6.893	MM	0.3791	3588.69067	157.79172	31.8446
3	10.854	MM	1.1590	2069.97412	29.76699	18.3681
4	21.020	MM	3.5686	3523.21362	16.45489	31.2636

Totals :				1.12694e4	330.27231	

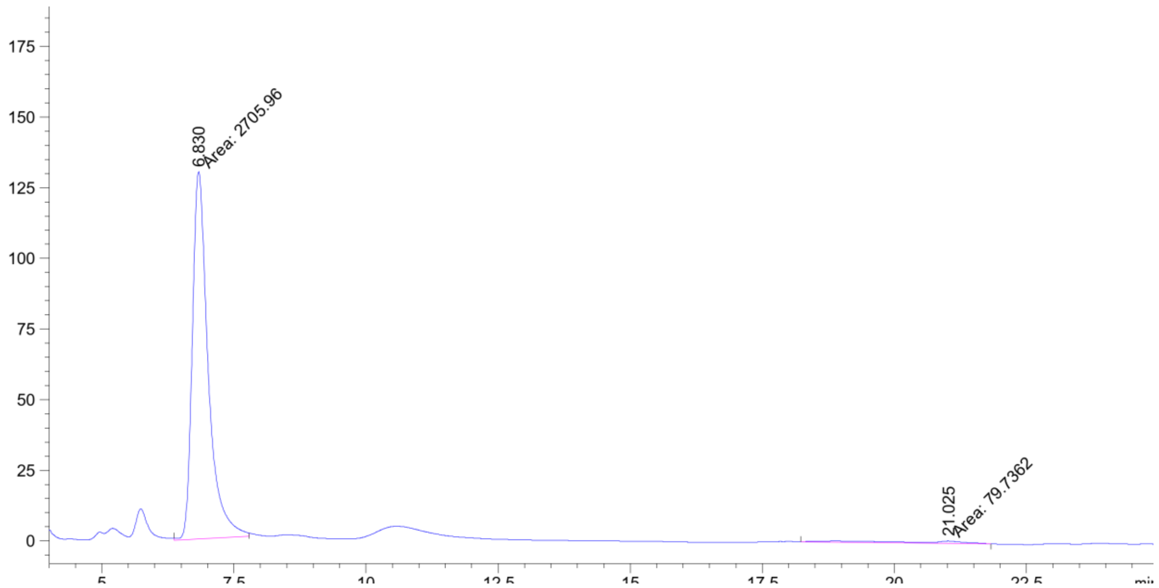

Signal 1: DAD1 A, Sig=220,4 Ref=off

Peak #	RetTime [min]	Type	Width [min]	Area [mAU*s]	Height [mAU]	Area %
1	6.830	MM	0.3471	2705.95972	129.93652	97.1377
2	21.025	MM	1.5336	79.73615	8.66532e-1	2.8623

Totals : 2785.69587 130.80306

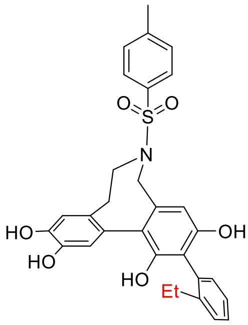

2g

According to the procedure, **2g** was obtained using **1g** (53.1, 0.1 mmol) in 96% yield (51mg) , light gray foam solid, 98% ee, 13/1 dr, (silica gel flash chromatography: DCM/ acetone = 10:1). R_f = 0.40 (DCM/acetone = 10:1).

^{1}H NMR (500 MHz, Acetone-d_6): δ 7.92 (s, 2H), 7.70 (d, *J* = 8.0 Hz, 2H), 7.38 (d, *J* = 8.0 Hz, 2H), 7.33 (d, *J* = 7.5 Hz, 1H), 7.27 (td, *J* = 7.5, 1.5 Hz, 1H), 7.20 (td, *J* = 7.5, 1.0 Hz, 1H), 7.15 (dd, *J* = 7.5, 1.0 Hz, 1H), 6.92 (d, *J* = 4.0 Hz, 1H), 6.75 (d, *J* = 15.0 Hz, 2H), 6.59 (s, 1H), 4.63 (d, *J* = 14.0 Hz, 1H), 4.04 (dd, *J* = 2.0, 7.0 Hz, 1H), 3.00 (s, 2H), 2.94 (d, *J* = 14.0 Hz, 1H), 2.82 (dd,

J = 14.0, 7.0 Hz, 1H), 2.60 (q, J = 7.5 Hz, 2H), 2.51 – 2.40 (m, 2H), 2.39 (s, 3H), 1.14 (t, J = 7.5 Hz, 3H).

^{13}C NMR (125 MHz, Acetone-d_6): δ 155.1, 152.1, 145.7, 144.5, 143.7, 137.7, 136.7, 133.8, 133.2, 132.0, 130.2, 128.8, 128.2, 127.6, 126.7, 126.2, 119.3, 117.8, 116.8, 116.3, 109.3, 50.4, 49.9, 33.3, 27.1, 21.0, 15.1.

HRMS (ESI) *m/z* calcd. for $C_{30}H_{29}NO_6S$ $(M+Na)^+$: 554.1608, found: 554.1604

Enantiomeric excess of **2g** is determined by HPLC (Chiralpak IB, Hexane/Isopropanol = 60/40, flow rate = 1.0 mL/min, 220 nm): major isomer: tr = 9.74 min; minor isomer: tr = 25.25 min. $[\alpha]^{20}_D$ = -730.67 (c = 1.0, $CHCl_3$).

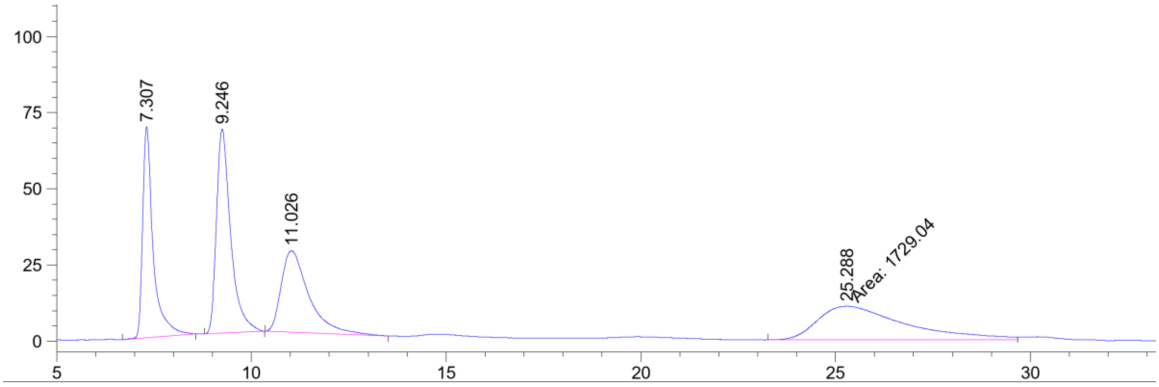

Signal 1: DAD1 A, Sig=220,4 Ref=off

Peak #	RetTime [min]	Type	Width [min]	Area [mAU*s]	Height [mAU]	Area %
1	7.307	BB	0.2727	1297.87842	69.24872	21.4648
2	9.246	BB	0.3804	1701.16150	66.95714	28.1345
3	11.026	BB	0.7315	1318.46033	26.72722	21.8052
4	25.288	MF	2.6107	1729.03687	11.03826	28.5955
Totals :				6046.53711	173.97134	

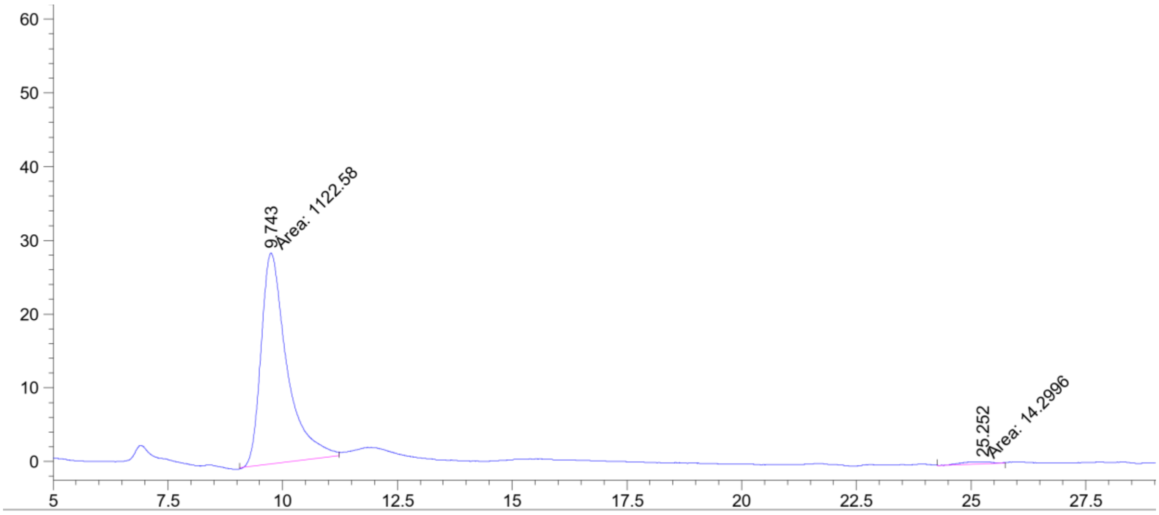

Signal 1: DAD1 A, Sig=220,4 Ref=off

Peak #	RetTime [min]	Type	Width [min]	Area [mAU*s]	Height [mAU]	Area %
1	9.743	MM	0.6543	1122.58240	28.59587	98.7422
2	25.252	MM	0.8455	14.29958	2.81882e-1	1.2578
Totals :				1136.88198	28.87776	

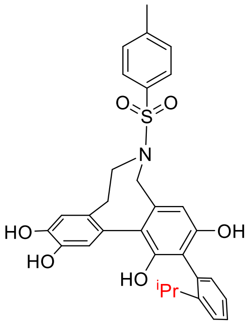

2h

According to the procedure, **2h** was obtained using **1h** (54.5 mg, 0.1 mmol) in 96% yield (52mg) , light gray foam solid, 91% ee, 17/1 dr, (silica gel flash chromatography: DCM/acetone = 10:1). R_f = 0.40 (DCM/acetone = 10:1).

^{1}H NMR (500 MHz, Acetone-d_6): δ 7.92 (s, 3H), 7.70 (d, J = 8.0 Hz, 2H), 7.40 (dd, J = 17.0, 8.0 Hz, 3H), 7.32 (td, J = 7.5, 1.5 Hz, 1H), 7.19 (td, J = 7.5, 1.0 Hz, 1H), 7.12 (dd, J = 7.5, 1.0 Hz, 1H), 6.92 (s, 1H), 6.76 (s, 1H), 6.73 (s, 1H), 6.50 (s, 1H), 4.64 (d, J = 14.0 Hz, 1H), 4.05 (dd, J =

12.0, 7.0 Hz, 1H), 3.04 – 2.97 (m, 3H), 2.94 (d, J = 14.0 Hz, 1H), 2.82 (dd, J = 14.0, 7.0 Hz, 1H), 2.53 – 2.45 (m, 1H), 2.45 – 2.36 (m, 4H), 1.21 (dd, J = 12.5, 7.0 Hz, 6H).

^{13}C NMR (125 MHz, Acetone-d_6): δ 155.2, 152.3, 149.1, 145.7, 143.7, 137.7, 136.7, 133.2, 131.8, 130.2, 128.4, 127.6, 126.7, 126.1, 125.7, 119.2, 117.8, 116.8, 116.3, 109.2, 50.4, 49.9, 33.3, 30.9, 23.9, 23.8, 21.0.

HRMS (ESI) *m/z* calcd. for $C_{31}H_{31}NO_6S$ $(M+Na)^+$: 568.1764, found: 568.1760

Enantiomeric excess of **2h** is determined by UPC^2 (CHIRALPAK® OD-3, CO_2/MeOH = 80/20,, flow rate = 1.0 mL/min, 220 nm): major isomer: tr = 9.99 min; minor isomer: tr = 14.06 min. $[\alpha]^{20}_{D}$ = -505.13 (c = 1.0, $CHCl_3$).

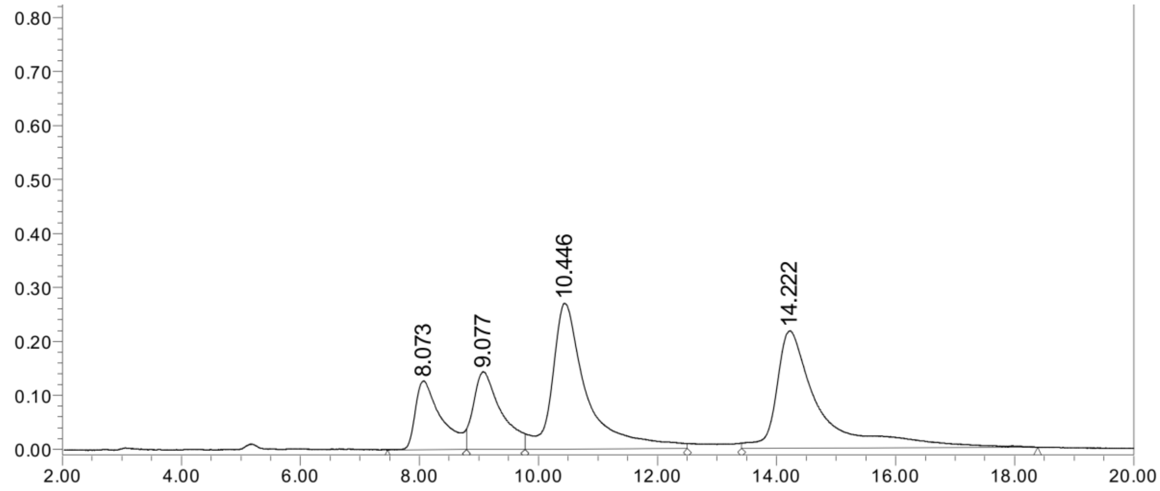

Signal: Sig = 220nm

Peak	RetTime	Area	Height	Area%
1	8.073	4541049	1271134	13.81
2	9.077	4641049	143902	14.15
3	10.446	10907268	270126	35.98
4	14.222	10933033	216970	36.06

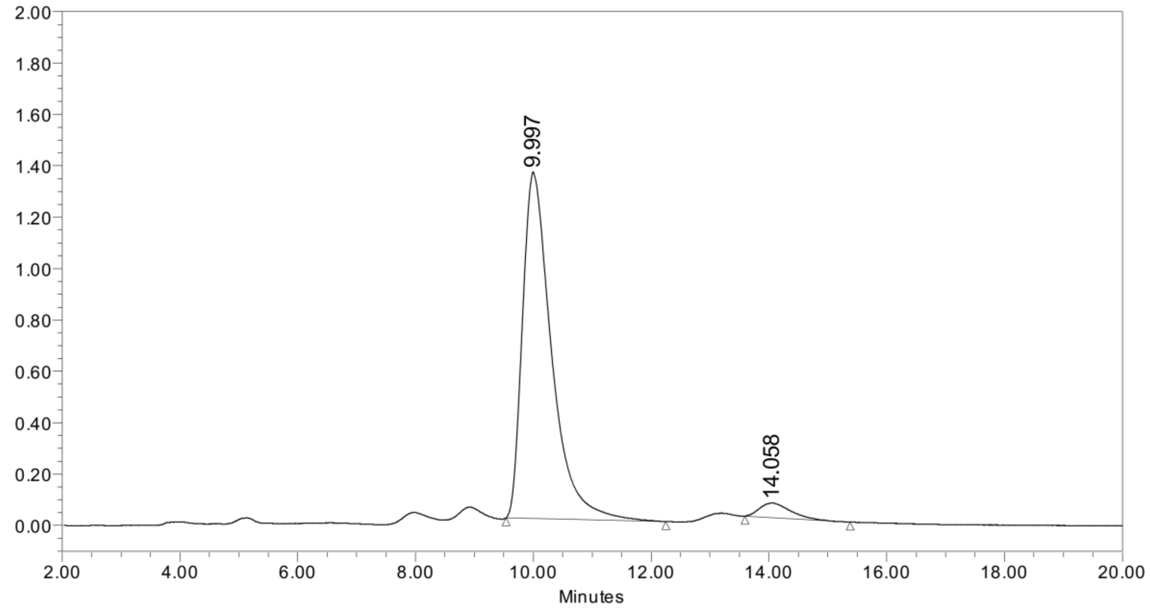

Peak	RetTime	Area	Height	Area%
1	9.997	46129932	1349910	95.69
2	14.058	2076700	56833	4.31

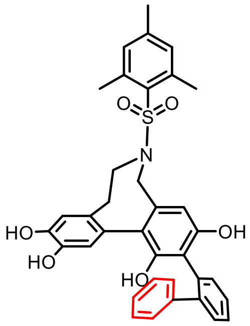

2i

According to the procedure, **2i** was obtained using **1i** (60.9 mg, 0.1 mmol) in 85% yield (51.6mg) , light gray foam solid, 92% ee, 10/1 dr, (silica gel flash chromatography: DCM/ acetone = 20:1). R_f = 0.40 (DCM/acetone = 10:1).

^{1}H NMR (500 MHz, CD_3OD): δ 7.42 – 7.33 (m, 4H), 7.30 (dd, *J* = 12.0, 7.0 Hz, 2H), 7.24 – 7.14 (m, 3H), 7.13 – 7.08 (m, 1H), 7.01 (d, *J* = 7.0 Hz, 2H), 6.65 (d, *J* = 6.0 Hz, 1H), 6.36 (d, *J* = 14.0 Hz, 1H), 4.56 (dd, *J* = 14.0, 8.0 Hz, 1H), 3.61 – 3.47 (m, 1H), 3.19 – 3.05 (m, 1H), 2.72 – 2.57 (m, 2H), 2.57 – 2.46 (m, 7H), 2.46 – 2.37 (m, 1H), 2.33 – 2.25 (m, 3H).

^{13}C NMR (125 MHz, CD_3OD): δ 155.4, 152.5, 146.6, 144.5, 144.3, 143.4, 141.8, 141.5, 138.3, 134.3, 134.1, 133.9, 133.1, 133.1, 133.0, 130.8, 130.7, 129.9, 128.6, 128.4, 128.3, 128.1, 127.5, 127.3, 120.2, 118.5, 117.9, 117.2, 109.9, 49.3, 34.1, 23.2, 23.1, 20.9.

HRMS (ESI) *m/z* calcd. for $C_{36}H_{33}NO_6S$ $(M+Na)^+$: 630.1921, found: 630.1913

Enantiomeric excess of **2i** is determined by UPC^2 (CHIRALPAK® IC-3, CO_2/MeOH = 70/30, flow rate = 1.0 mL/min, 220 nm): major isomer: tr = 4.83 min; minor isomer: tr = 6.92 min. $[\alpha]^{20}_D$ = -113.13 (c = 1.0, MeOH)

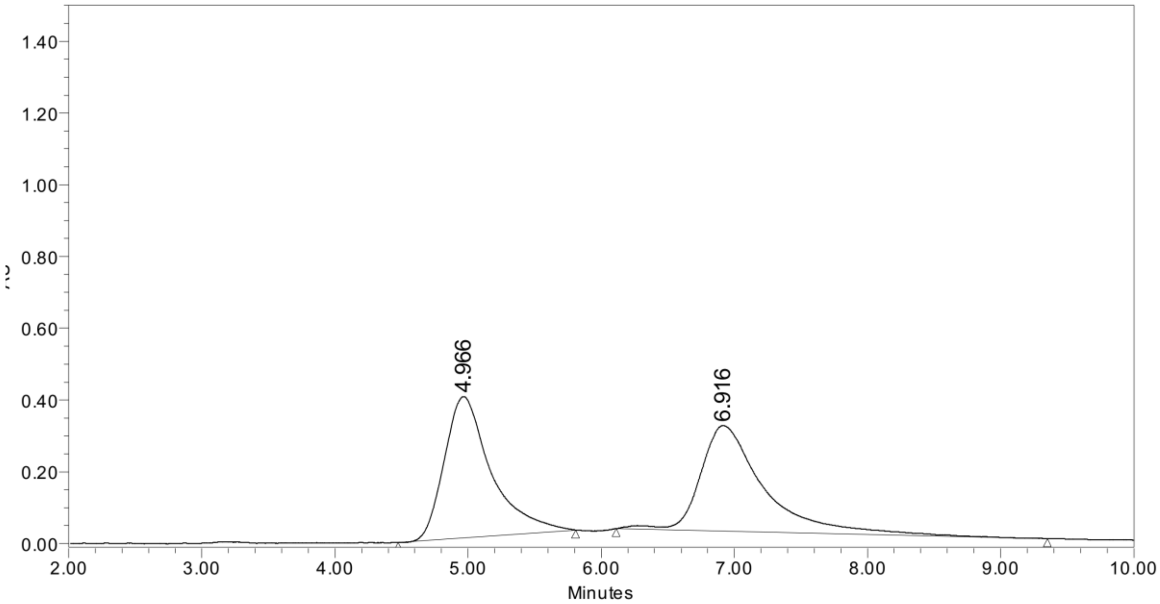

Signal: Sig = 220nm

Peak	RetTime	Area	Height	Area%
1	4.966	9592583	393643	49.52
2	6.916	9777743	294046	50.48

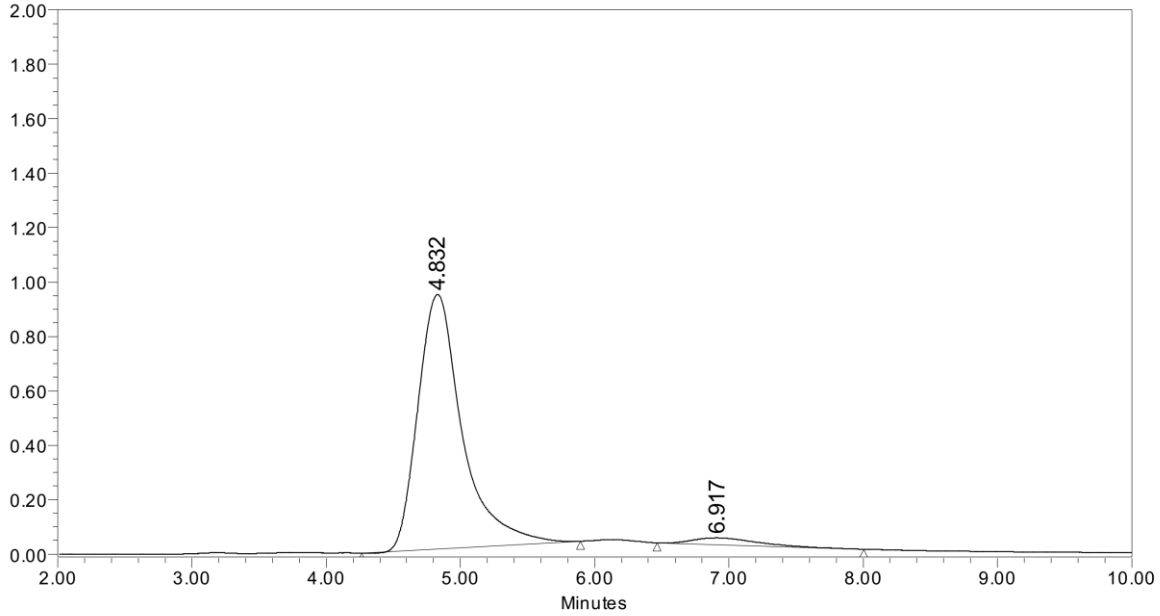

Peak	RetTime	Area	Height	Area%

1	4.832	21467192	935115	96.25
2	6.917	836200	25659	3.75

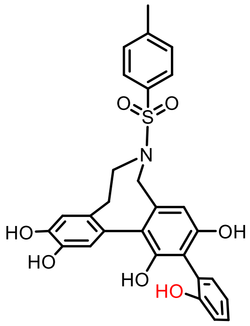

2j

According to the procedure, **2j** was obtained using **1j** (52.1 mg, 0.1 mmol) in 92% yield (47.7mg) , light gray foam solid, 72% ee, >20/1 dr, (silica gel flash chromatography: DCM/ acetone = 20:1). R_f = 0.40 (DCM/acetone = 10:1).

^{1}H NMR (500 MHz, CD_3OD): δ 7.63 (dd, *J* = 8.0, 2.5 Hz, 2H), 7.32 (d, *J* = 8.0 Hz, 2H), 7.28 – 7.15 (m, 1H), 7.02 – 6.84 (m, 3H), 6.75 (d, *J* = 24.5 Hz, 1H), 6.62 (s, 1H), 4.61 (d, *J* = 14.0 Hz, 1H), 4.05 – 3.85 (m, 1H), 2.94 (d, *J* = 14.0 Hz, 1H), 2.81 – 2.65 (m, 1H), 2.53 – 2.42 (m, 1H), 2.37 (d, *J* = 6.0 Hz, 4H).

^{13}C NMR (126 MHz, CD_3OD): δ 154.6, 154.5, 151.6, 151.5, 145.0, 143.4, 143.1, 137.2, 135.8, 135.7, 132.7, 132.7, 132.4, 132.3, 129.5, 129.4, 128.5, 126.8, 126.3, 120.9, 119.7, 119.5, 117.3, 115.8, 115.69, 115.6, 113.4, 113.3, 109.1, 108.9, 49.7, 49.2, 49.1, 32.6, 32.5, 20.1.

HRMS (ESI) *m/z* calcd. for $C_{28}H_{25}NO_7S$ $(M+Na)^+$: 542.1244, found: 542.1236

Enantiomeric excess of **2j** is determined by UPC^2 (CHIRALPAK® IG-3, CO_2/MeOH = 70/30, flow rate = 1.0 mL/min, 220 nm): major isomer: tr = 10.78 min; minor isomer: tr = 5.77 min. $[\alpha]^{20}_D$ = -109.27 (c = 1.0, $CHCl_3$)

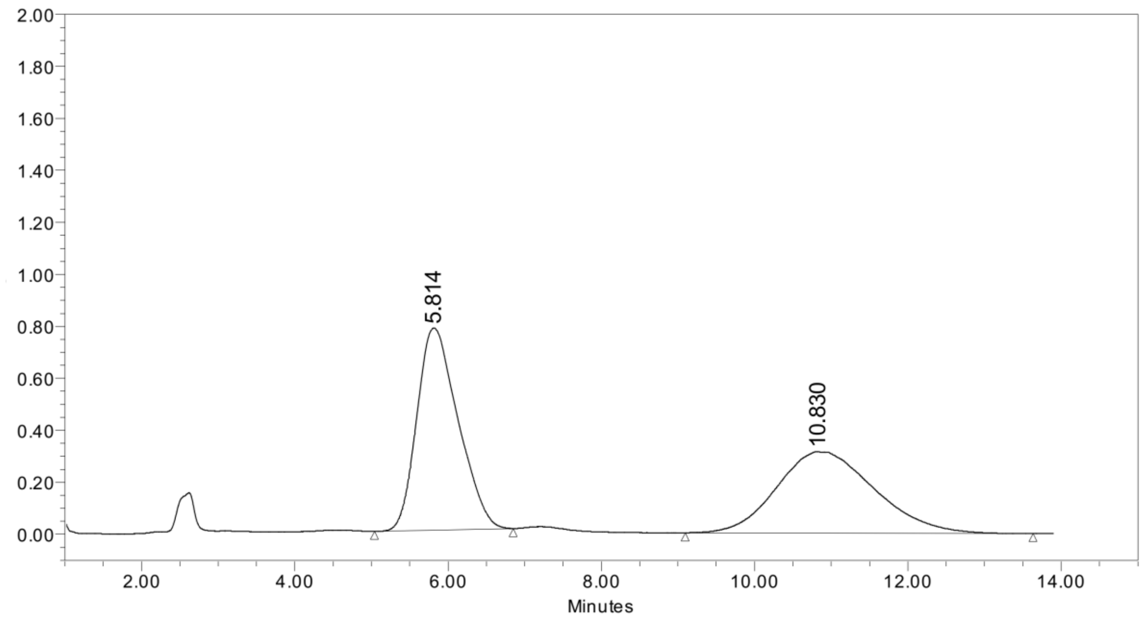

Signal: Sig = 220nm

Peak	RetTime	Area	Height	Area%
1	5.814	28932705	777772	50.94
2	10.830	27869817	312989	49.06

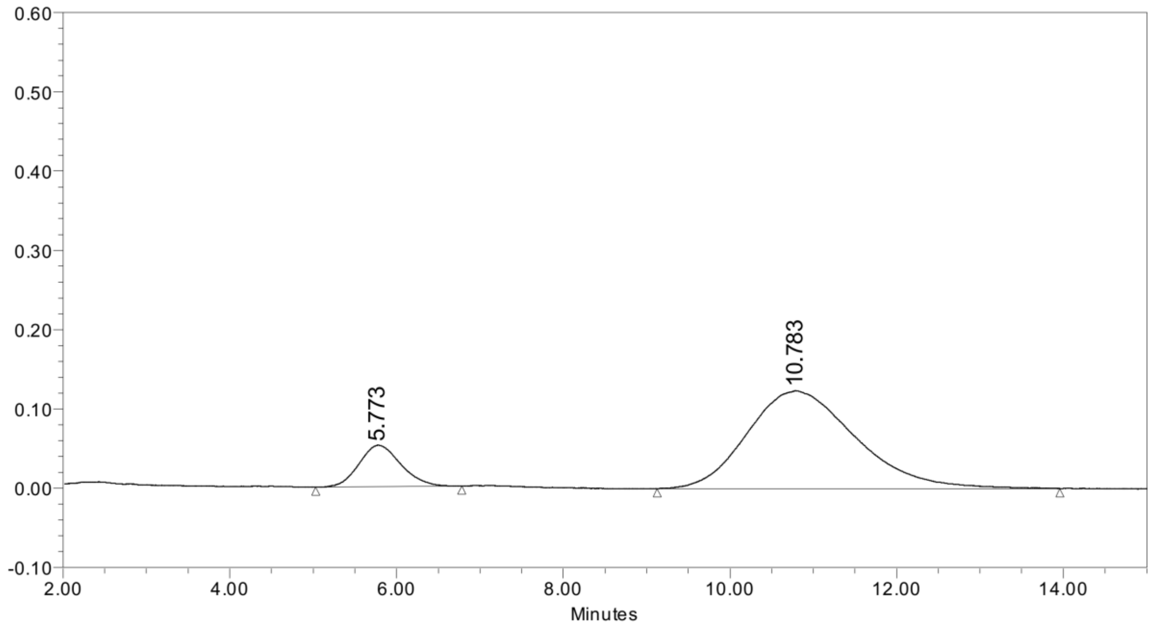

Peak	RetTime	Area	Height	Area%
1	5.773	1815602	52223	14.03
2	10.783	11128586	123491	85.97

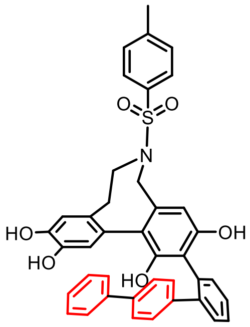

2k

According to the procedure, **2k** was obtained using **1k** (65.7 mg, 0.1 mmol) in 93% yield (61.6mg), light gray foam solid, 90% ee, >20/1 dr, (silica gel flash chromatography: DCM/ acetone = 20:1). R_f = 0.40 (DCM/acetone = 10:1).

^{1}H NMR (500 MHz, CD_3OD): δ 7.61 – 7.54 (m, 4H), 7.50 – 7.20 (m, 14H), 6.72 (s, 1H), 6.62 (s, 1H), 6.44 (s, 1H), 4.47 (d, *J* = 14.0 Hz, 1H), 3.94 (dd, *J* = 11.5, 7.0 Hz, 1H), 2.81 (d, *J* = 14.0 Hz, 1H), 2.70 (dd, *J* = 14.0, 7.0 Hz, 1H), 2.46 – 2.30 (m, 5H).

^{13}C NMR (125 MHz, CD_3OD): δ 155.7, 152.6, 146.5, 144.7, 144.6, 144.1, 142.5, 142.2, 140.5, 138.3, 137.2, 134.4, 133.8, 133.1, 130.8, 130.7, 130.4, 129.7, 128.7, 128.2, 128.2, 128.1, 127.9, 127.4, 127.0, 120.1, 118.6, 118.0, 117.3, 109.9, 51.0, 50.7, 34.1, 21.4.

HRMS (ESI) *m/z* calcd. for $C_{40}H_{33}NO_6S$ $(M+Na)^+$: 678.1921, found: 678.1907

Enantiomeric excess of **2k** is determined by HPLC (Chiralpak AD-H, Hexane/Isopropanol = 70/30, flow rate = 1.0 mL/min, 220 nm): major isomer: tr = 24.36 min; minor isomer: tr = 11.54 min. $[\alpha]^{20}_D$ = -440.60 (c = 1.0, MeOH).

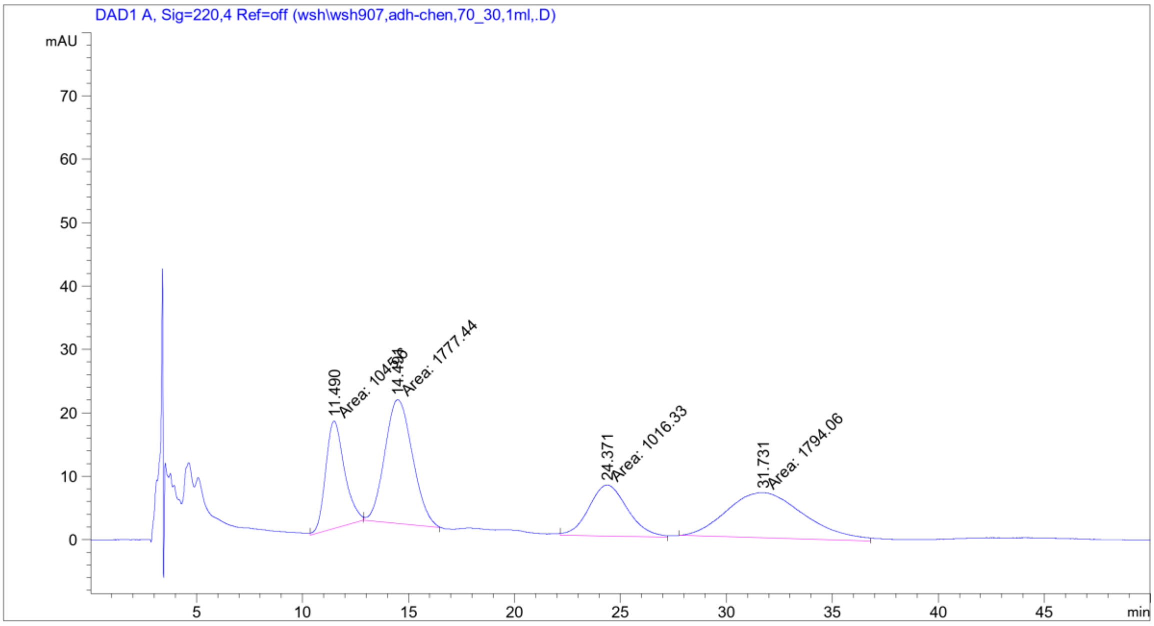

Signal 1: DAD1 A, Sig=220,4 Ref=off

Peak #	RetTime [min]	Type	Width [min]	Area [mAU*s]	Height [mAU]	Area %
1	11.490	MM	1.0298	1045.59900	16.92318	18.5606
2	14.491	MM	1.5183	1777.44312	19.51125	31.5517
3	24.371	MM	2.1067	1016.32770	8.04052	18.0410
4	31.731	MM	4.2239	1794.05786	7.07901	31.8466
Totals :				5633.42767	51.55396	

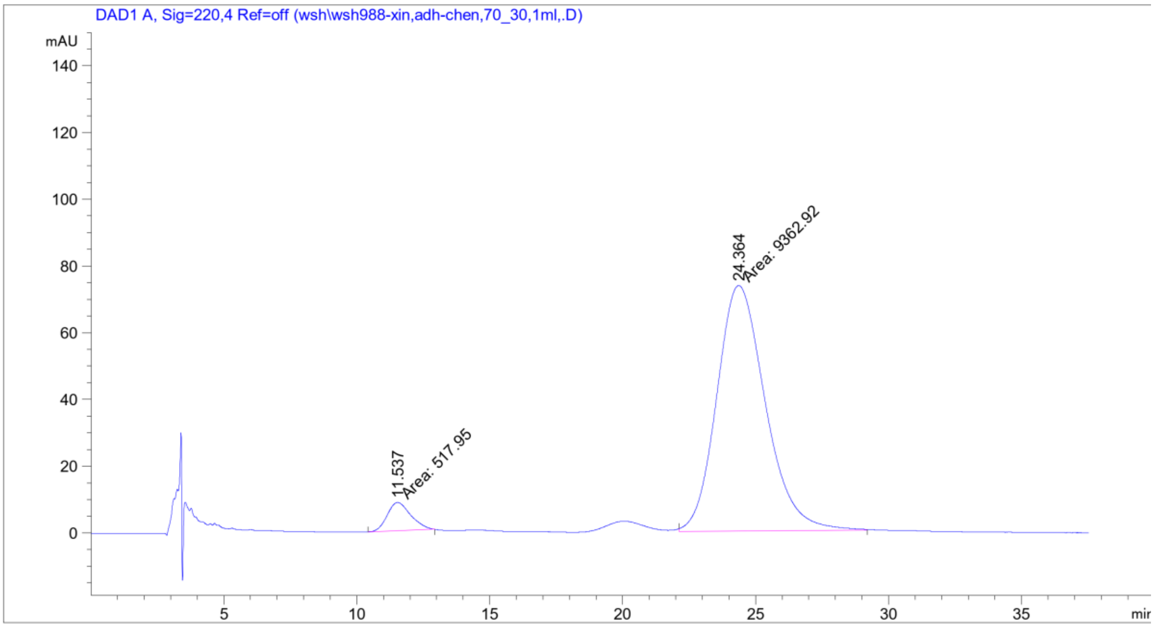

Signal 1: DAD1 A, Sig=220,4 Ref=off

Peak #	RetTime [min]	Type	Width [min]	Area [mAU*s]	Height [mAU]	Area %
1	11.537	MM	1.0226	517.95038	8.44209	5.2420
2	24.364	MM	2.1203	9362.91504	73.59884	94.7580

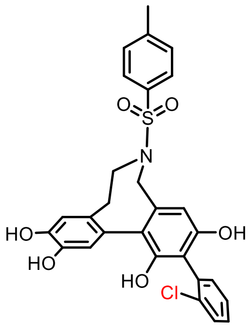

2l

According to the procedure, **2l** was obtained using **1l** (53.9 mg, 0.1 mmol) in 89% yield (48mg), light gray foam solid, 92% ee, >20/1 dr, (silica gel flash chromatography: DCM/acetone = 20:1). R_f = 0.40 (DCM/acetone = 10:1).

^{1}H NMR (500 MHz, CD_3OD): δ 7.63 (d, *J* = 8.0 Hz, 2H), 7.51 – 7.42 (m, 1H), 7.29 (dd, *J* = 21.5, 6.5 Hz, 5H), 6.88 (s, 1H), 6.73 (s, 1H), 6.65 (s, 1H), 4.60 (d, *J* = 14.0 Hz, 1H), 3.98 (dd, *J* = 12.0, 7.1 Hz, 1H), 2.92 (d, *J* = 14.0 Hz, 1H), 2.74 (dd, *J* = 14.0, 7.0 Hz, 1H), 2.57 – 2.22 (m, 5H).

^{13}**C NMR** (126 MHz, CD_3OD): δ 155.8, 152.8, 146.6, 144.8, 144.6, 138.9, 137.1, 136.3, 135.4, 134.1, 133.9, 130.8, 130.2, 129.6, 128.2, 127.5, 127.2, 120.3, 118.4, 117.5, 116.0, 109.9, 51.1, 50.6, 34.1, 21.5.

HRMS (ESI) *m/z* calcd. for $C_{28}H_{24}NO_6SCl$ $(M+Na)^+$: 560.0905, found: 560.0901

Enantiomeric excess of **2l** is determined by HPLC (Chiralpak IB, Hexane/Isopropanol = 70/30, flow rate = 1.0 mL/min, 220 nm): major isomer: tr = 19.67 min; minor isomer: tr = 29.20 min. $[\alpha]^{20}_{D}$ = -231.67 (c = 1.0, MeOH).

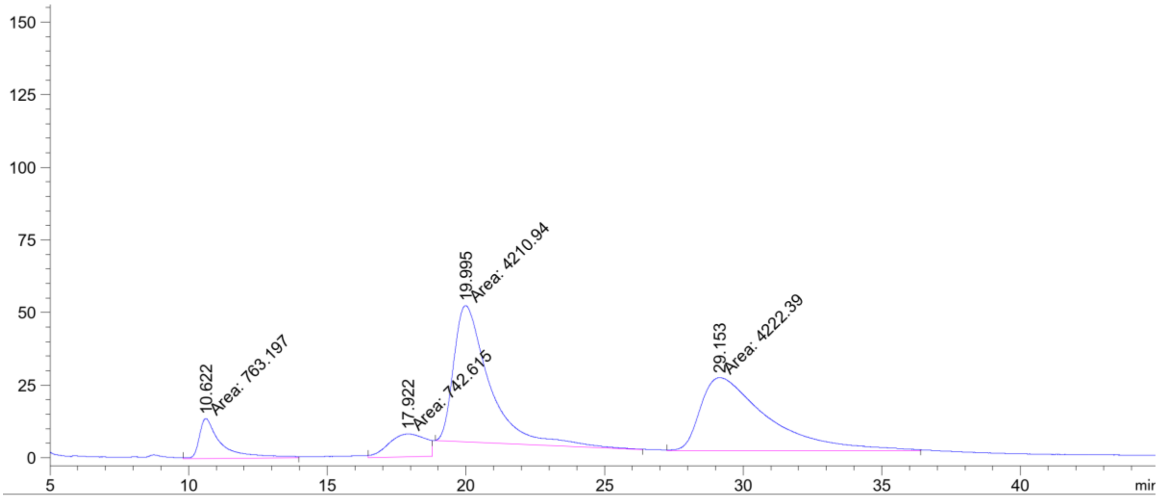

Signal 1: DAD1 A, Sig=220,4 Ref=off

Peak #	RetTime [min]	Type	Width [min]	Area [mAU*s]	Height [mAU]	Area %
1	10.622	MM	0.9262	763.19714	13.73325	7.6787
2	17.922	MF	1.5656	742.61523	7.90569	7.4716
3	19.995	MM	1.4973	4210.93604	46.87273	42.3672
4	29.153	MM	2.8028	4222.39014	25.10829	42.4825
Totals :				9939.13855	93.61996	

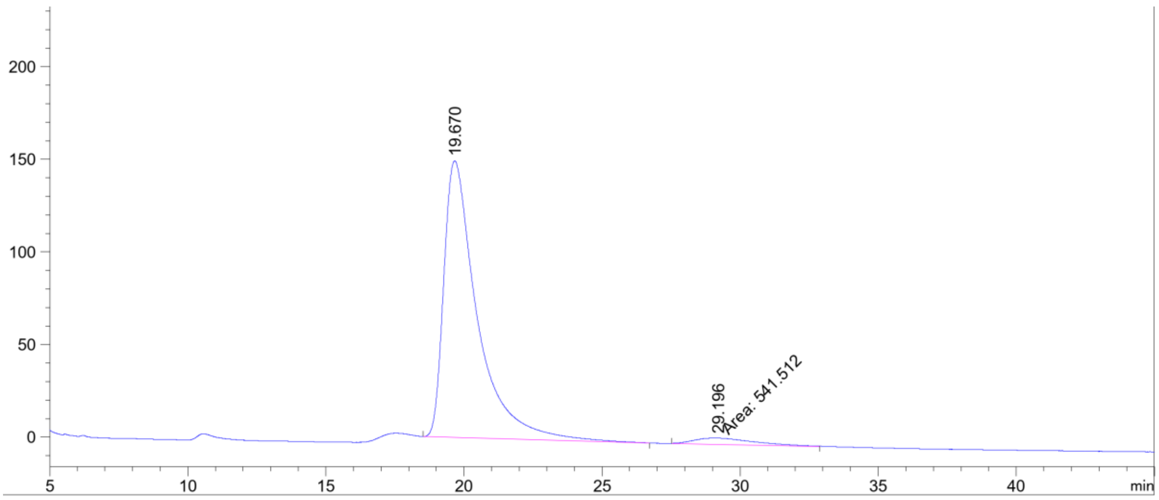

Signal 1: DAD1 A, Sig=220,4 Ref=off

Peak #	RetTime [min]	Type	Width [min]	Area [mAU*s]	Height [mAU]	Area %
1	19.670	BB	1.2055	1.25914e4	149.36957	95.8767
2	29.196	MM	2.5621	541.51190	3.52264	4.1233

Totals : 1.31329e4 152.89221

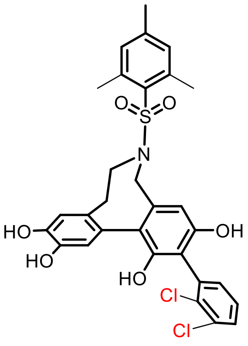

2m

According to the procedure, **2m** was obtained using **1m** (60.1 mg, 0.1 mmol) in 89% yield (54mg) , light gray foam solid, 92% ee, > 20/1 dr, (silica gel flash chromatography: DCM/acetone = 20:1). R_f = 0.40 (DCM/acetone = 10:1).

^{1}H NMR (500 MHz, CD_3OD): δ 7.46 (dd, *J* = 8.0, 1.0 Hz, 1H), 7.24 (dt, *J* = 7.5, 7.0 Hz, 2H), 7.03 (s, 2H), 6.76 (s, 1H), 6.71 (s, 1H), 6.67 (s, 1H), 4.73 (d, *J* = 14.0 Hz, 1H), 3.56 (dd, *J* = 13.0, 7.0 Hz, 1H), 3.25 (d, *J* = 14.0 Hz, 1H), 2.68 (ddd, *J* = 27.5, 15.5, 5.5 Hz, 2H), 2.56 (s, 6H), 2.46 (dd, *J* = 14.0, 10.5 Hz, 1H), 2.30 (s, 3H).

^{13}C NMR (125 MHz, CD_3OD): δ 155.5, 152.7, 146.8, 144.8, 144.3, 141.4, 139.4, 138.2, 134.6, 134.3, 133.9, 133.7, 133.2, 132.4, 130.2, 128.2, 127.0, 120.6, 118.3, 117.5, 115.9, 110.3, 49.4, 34.2, 29.5, 23.1, 21.0.

HRMS (ESI) *m/z* calcd. for $C_{30}H_{27}NO_6SCl_2$ $(M+Na)^+$: 622.0828, found: 622.0818

Enantiomeric excess of **2m** is determined by UPC^2 (CHIRALPAK® IG-3, CO_2/MeOH = 80/20, flow rate = 1.0 mL/min, 220 nm): major isomer: tr = 31.37min; minor isomer: tr = 14.24 min. $[\alpha]^{20}_D$ = -17.00 (c = 1.0, MeOH)

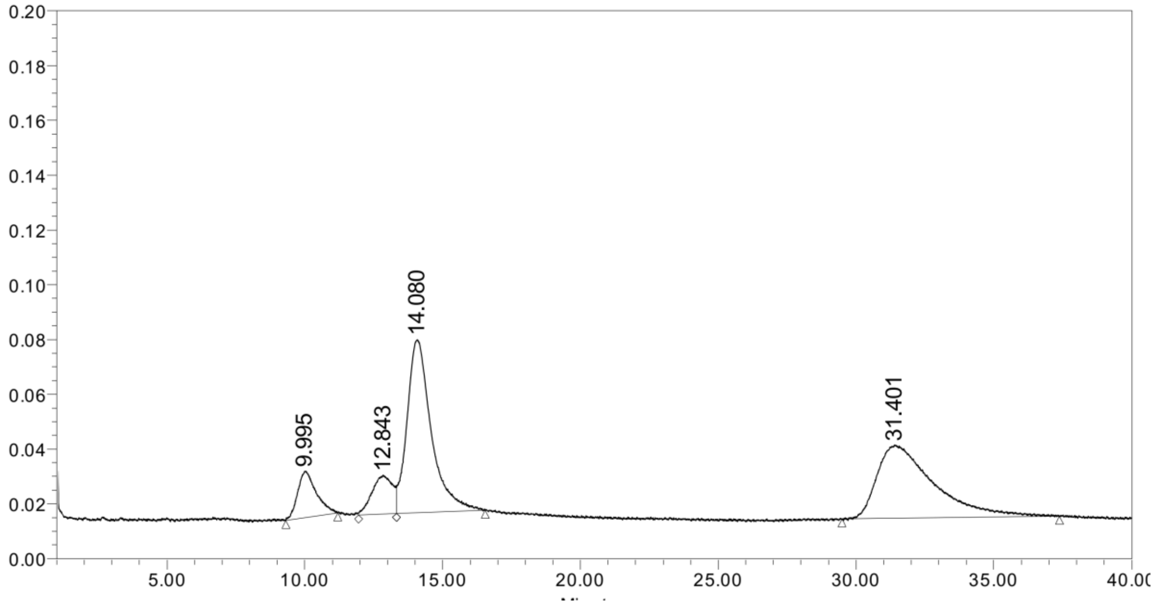

Signal: Sig = 220 nm

Peak	RetTime	Area	Height	Area%
1	9.995	798644	16925	8.69
2	12.843	733247	14144	7.98
3	14.080	3845061	63187	41.86
4	31.401	3809036	26723	41.47

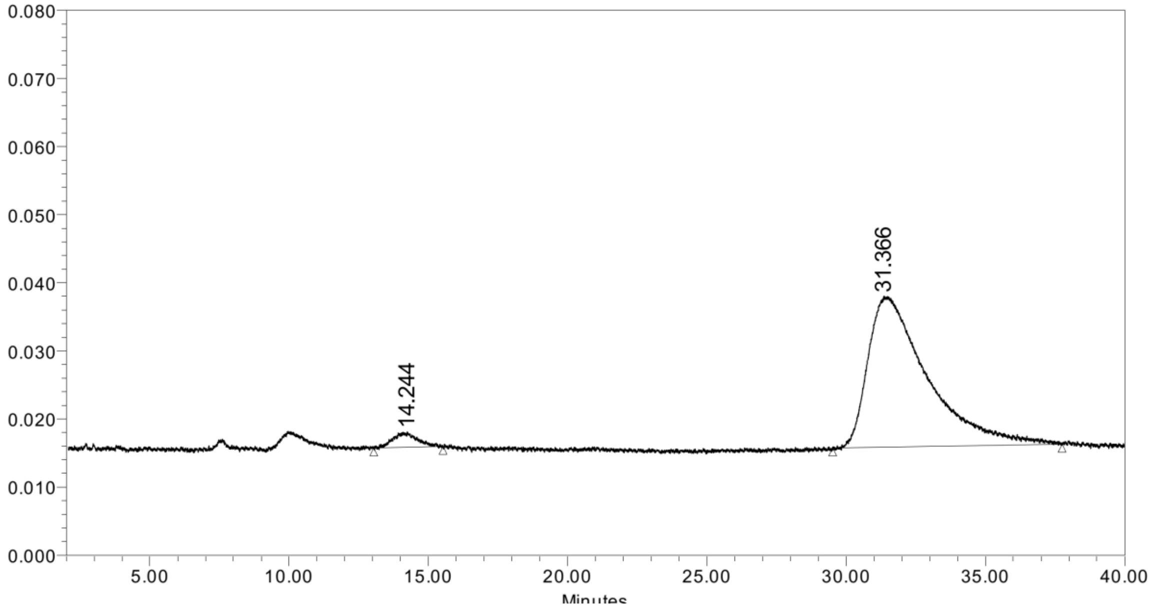

Peak	RetTime	Area	Height	Area%
1	14.244	130161	2255	3.82
2	31.366	3273229	22171	96.18

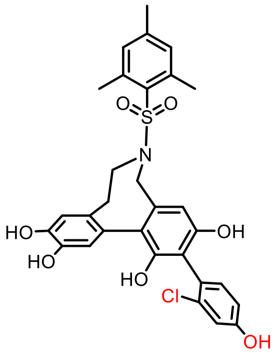

2n

According to the procedure, **2n** was obtained using **1n** (58.3 mg, 0.1 mmol) in 85% yield (50mg), light gray foam solid, 98% ee, >20/1 dr, (silica gel flash chromatography: DCM/acetone = 10:1). R_f = 0.40 (DCM/acetone = 4:1).

^{1}H NMR (500 MHz, Acetone-d_6): δ 7.92 (s, 1H), 7.15 (d, *J* = 8.5 Hz, 1H), 7.05 (s, 2H), 6.98 (s, 1H), 6.85 – 6.79 (m, 2H), 6.77 (s, 1H), 6.71 (s, 1H), 6.65 (s, 1H), 4.71 (d, *J* = 14.5 Hz, 1H), 3.63 (dd, *J* = 13.0, 7.0 Hz, 1H), 3.26 (d, *J* = 14.0 Hz, 1H), 3.06 (s, 4H), 2.76 (dd, *J* = 14.0, 7.0 Hz, 1H), 2.67 (t, *J* = 12.0 Hz, 1H), 2.58 (s, 6H), 2.48 – 2.41 (m, 1H), 2.30 (s, 3H).

^{13}C NMR (125 MHz, Acetone- d_6): δ 158.4, 155.6, 153.0, 146.2, 144.1, 143.3, 140.8, 138.8, 136.4, 134.6, 134.5, 134.0, 132.8, 127.0, 125.2, 119.7, 118.1, 117.2, 116.9, 115.0, 114.8, 109.8, 55.4, 49.0 , 33.9, 30.6, 23.0, 20.9.

HRMS (ESI) *m/z* calcd. for $C_{30}H_{28}NO_7SCl$ $(M+Na)^+$: 604.1167, found: 604.1163

Enantiomeric excess of **2n** is determined by HPLC (Chiralpak IB, Hexane/Isopropanol = 60/40, flow rate = 1.0 mL/min, 254 nm): major isomer: tr = 8.41 min; minor isomer: tr = 12.92 min. $[\alpha]^{20}_D$ = -138.46 (c = 1.0, MeOH)

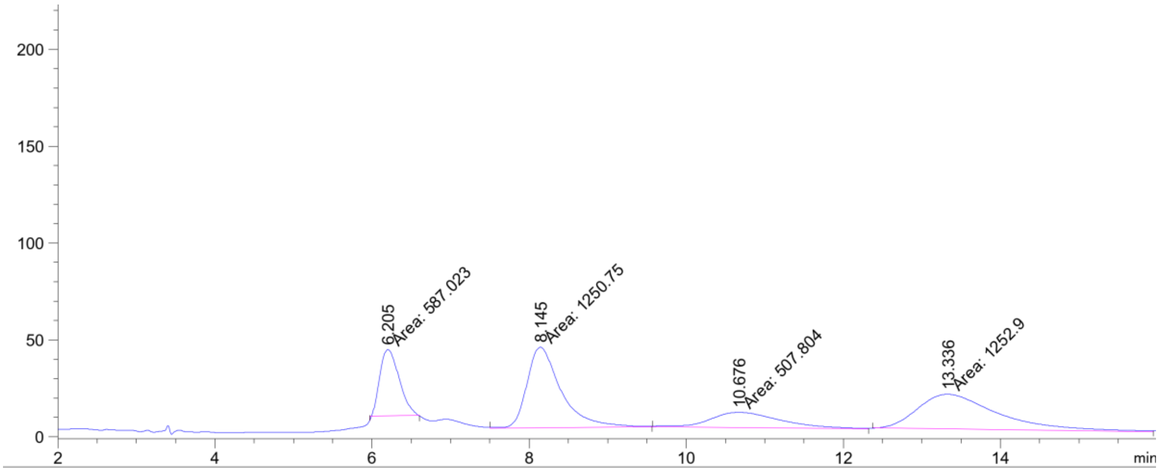

Signal 1: DAD1 A, Sig=220,4 Ref=off

Peak #	RetTime [min]	Type	Width [min]	Area [mAU*s]	Height [mAU]	Area %
1	6.205	MM	0.2852	587.02289	34.30146	16.3131
2	8.145	MM	0.5011	1250.74780	41.59957	34.7577
3	10.676	MM	1.0594	507.80389	7.98923	14.1116
4	13.336	MM	1.1705	1252.89929	17.84013	34.8175
Totals :				3598.47388	101.73039	

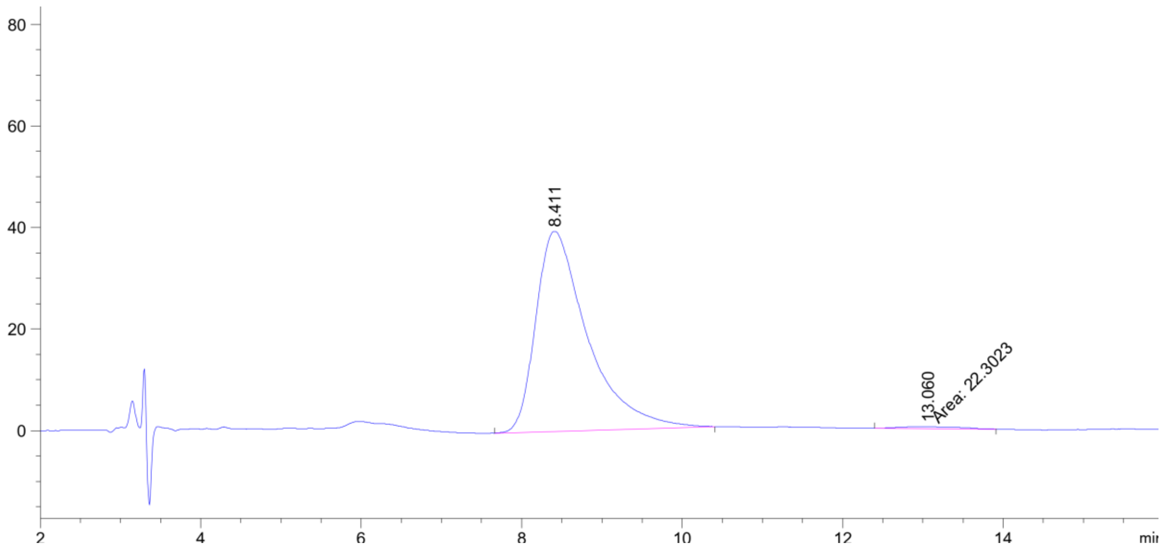

Signal 1: DAD1 A, Sig=220,4 Ref=off

Peak #	RetTime [min]	Type	Width [min]	Area [mAU*s]	Height [mAU]	Area %
1	8.411	BB	0.6563	1763.88293	39.39827	98.7514
2	13.060	MM	0.9155	22.30227	4.06025e-1	1.2486

Totals : 1786.18521 39.80430

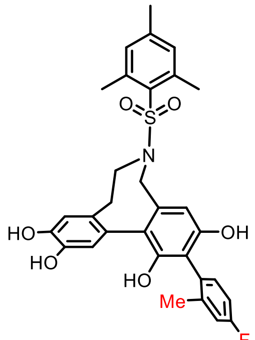

2o

According to the procedure, **2o** was obtained using **1o** (56.5 mg, 0.1 mmol) in 85% yield (47.8mg), light gray foam solid, 93% ee, 9/1 dr, (silica gel flash chromatography: DCM/ acetone = 10:1). R_f = 0.40 (DCM/acetone = 10:1).

^{1}H NMR (500 MHz, Acetone-d_6): δ 7.97 (s, 3H), 7.25 – 7.13 (m, 1H), 7.06 (d, J = 7.0 Hz, 3H), 6.95 (td, J = 8.5, 2.4 Hz, 1H), 6.79 (s, 1H), 6.78 (s, 1H), 6.74 (s, 1H), 6.70 (s, 1H), 4.73 (d, J = 14.0 Hz, 1H), 3.62 (dd, J = 13.0, 7.0 Hz, 1H), 3.28 (d, J = 14.0 Hz, 1H), 2.77 (dd, J = 14.0, 7.0 Hz, 1H), 2.72 – 2.62 (m, 1H), 2.58 (s, 6H), 2.46 (dd, J = 14.0, 10.5 Hz, 1H), 2.31 (s, 3H), 2.25 (s, 3H).

^{13}C NMR (125 MHz, Acetone-d_6): δ 163.0 (d, J = 242.6 Hz), 154.9, 152.3, 145.9, 143.9, 143.1, 141.5, 140.6, 138.5, 134.25, 133.81 (d, J = 8.4 Hz), 132.6, 130.9, 126.7, 119.7, 117.9, 116.9 (d, J = 21.1 Hz), 115.4, 112.9 (d, J = 21.1 Hz), 109.8, 48.7, 33.6, 22.8, 20.6, 19.9.

^{19}F NMR (470 MHz, CD_3OD): δ -118.5.

HRMS (ESI) *m/z* calcd. for $C_{31}H_{30}NO_6SF$ $(M+Na)^+$: 586.1670, found: 586.1666.

Enantiomeric excess of **2o** is determined by UPC^2 (CHIRALPAK® IG-3, CO_2/MeOH = 70/30, flow rate = 1.0 mL/min, 220 nm): major isomer: tr = 14.52min; minor isomer: tr = 4.68 min. $[\alpha]^{20}_D$ = -74.87 (c = 1.0, $CHCl_3$).

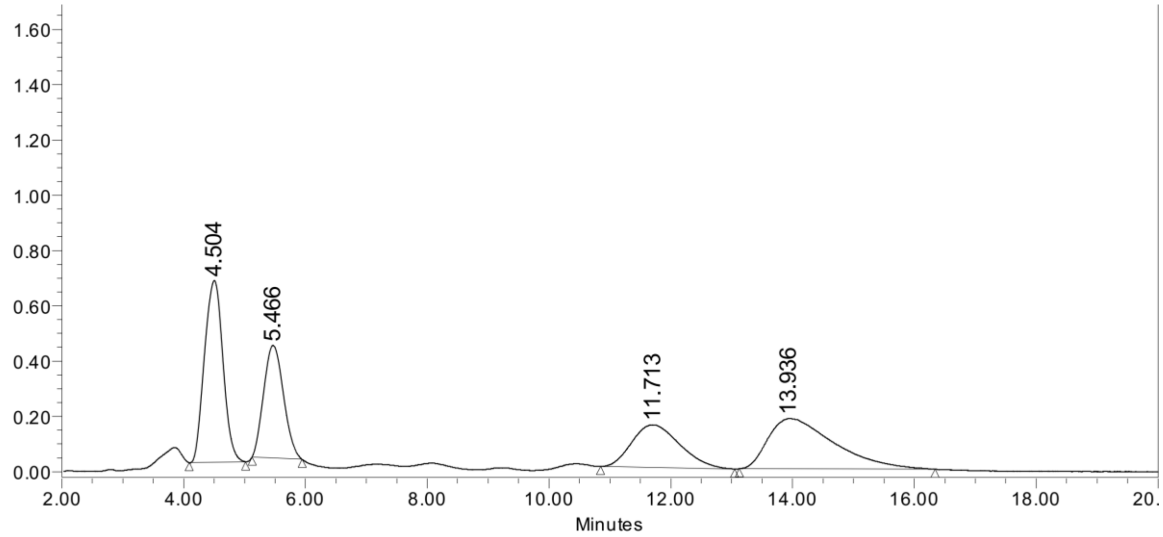

Signal: Sig= 220nm

Peak	RetTime	Area	Height	Area%
1	4.504	13868641	657415	30.67
2	5.466	9187667	406974	20.32
3	11.713	8630954	154106	19.00
4	13.936	13726862	182206	30.36

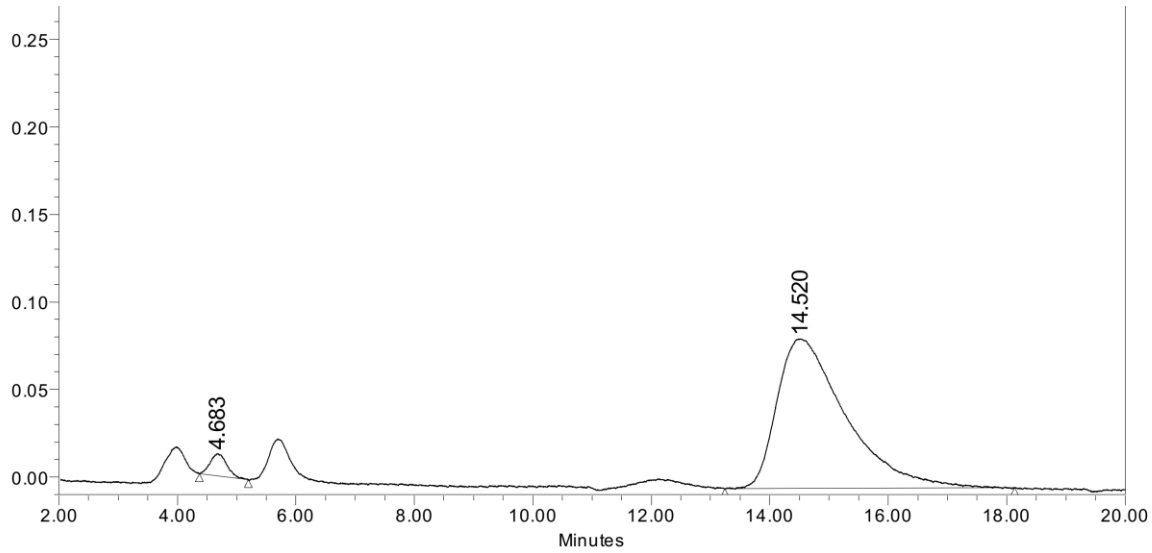

Peak	RetTime	Area	Height	Area%
1	4.683	247764	12601	3.47
2	14.520	6890996	85397	96.53

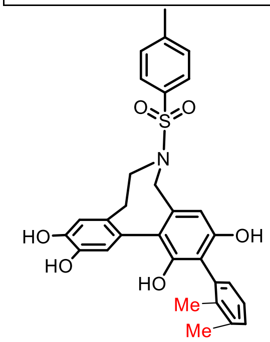

2p

According to the procedure, **2p** was obtained using **1p** (53.3mg, 0.1 mmol) in 95% yield (50.45mg), light gray foam solid, 89% ee, 8/1 dr, (silica gel flash chromatography: DCM/ acetone = 20:1). R_f = 0.40 (DCM/acetone = 10:1).

^{1}H NMR (500 MHz, Acetone-d_6): δ 7.70 (d, J = 8.0 Hz, 2H), 7.38 (d, J = 8.0 Hz, 2H), 7.18 – 7.07 (m, 2H), 7.03 (d, J = 7.0 Hz, 1H), 6.92 (s, 1H), 6.78 (s, 1H), 6.73 (s, 1H), 4.64 (d, J = 14.0 Hz, 1H), 4.04 (dd, J = 11.5, 7.5 Hz, 1H), 2.95 (d, J = 13.5 Hz, 1H), 2.81 (dd, J = 14.0, 7.0 Hz, 1H), 2.48 (t, J = 11.5 Hz, 1H), 2.44 – 2.34 (m, 4H), 2.31 (s, 3H), 2.15 (s, 3H).

^{13}C NMR (125 MHz, Acetone-d_6): δ 154.9, 152.0, 145.6, 143.7, 137.6, 137.0, 136.7, 134.2, 133.1, 130.2, 129.6, 129.5, 127.6, 126.8, 125.8, 119.3, 117.9, 116.8, 116.8, 109.3 50.4, 49.9, 33.3, 21.0, 20.4, 16.3.

HRMS (ESI) m/z calcd. for $C_{30}H_{29}NO_6S$ $(M+Na)^+$: 554.1608, found: 554.1605.

Enantiomeric excess of **2p** is determined by HPLC (Chiralpak IG, Hexane/Isopropanol = 70/30,

flow rate = 1.0 mL/min, 220 nm): major isomer: tr = 24.13min; minor isomer: tr = 30.06 min. $[\alpha]^{20}_{D}$ = -205.93 (c = 1.0, $CHCl_3$).

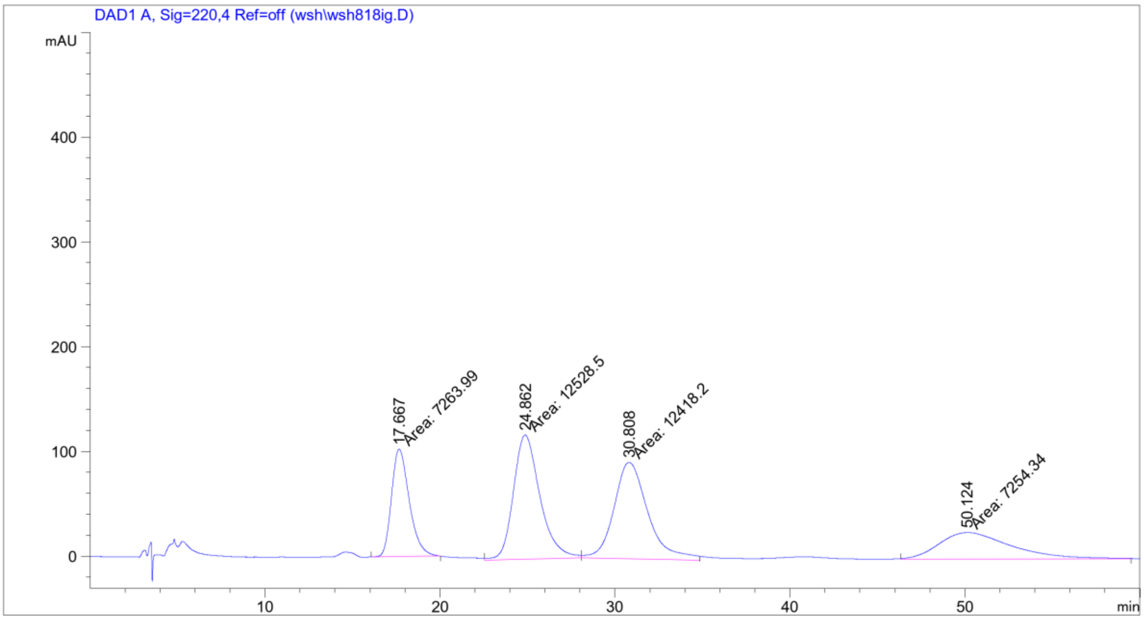

Signal 1: DAD1 A, Sig=220,4 Ref=off

Peak #	RetTime [min]	Type	Width [min]	Area [mAU*s]	Height [mAU]	Area %
1	17.667	MM	1.1800	7263.99365	102.59946	18.4062
2	24.862	MM	1.7604	1.25285e4	118.61603	31.7458
3	30.808	MM	2.2495	1.24182e4	92.00761	31.4663
4	50.124	MM	4.7478	7254.33740	25.46548	18.3817

Totals : 3.94650e4 338.68858

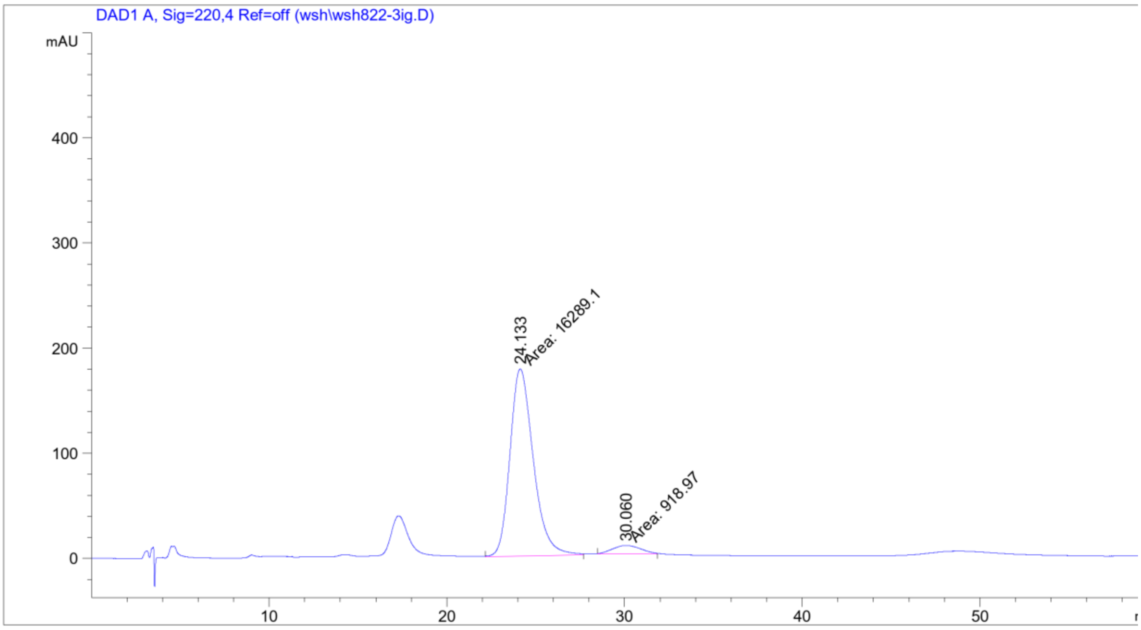

Signal 1: DAD1 A, Sig=220,4 Ref=off

Peak #	RetTime [min]	Type	Width [min]	Area [mAU*s]	Height [mAU]	Area %
1	24.133	MM	1.5245	1.62891e4	178.07860	94.6596
2	30.060	MM	1.8621	918.97009	8.22515	5.3404

Totals : 1.72080e4 186.30375

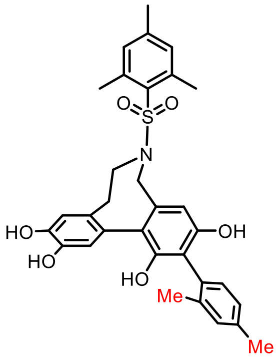

2q

According to the procedure, **2q** was obtained using **1q** (56.1 mg, 0.1 mmol) in 90% yield (52mg), light gray foam solid, 92% ee, 11/1 dr, (silica gel flash chromatography: DCM/acetone = 20:1). R_f = 0.40 (DCM/acetone = 10:1).

^{1}H NMR (500 MHz, Acetone-d_6): δ 7.90 (s, 2H), 7.70 (s, 1H), 7.16 – 6.98 (m, 5H), 6.83 (d, J = 7.5 Hz, 1H), 6.80 (d, J = 6.5 Hz, 1H), 6.67 (s, 1H), 6.52 (d, J = 15.5 Hz, 1H), 4.74 (d, J = 14.0

Hz, 1H), 3.65 (dd, J = 13.0, 7.0 Hz, 1H), 3.29 (d, J = 14.0 Hz, 1H), 2.79 (dd, J = 14.0, 7.0 Hz, 1H), 2.69 (dd, J = 12.5, 11.0 Hz, 1H), 2.60 (s, 6H), 2.53 – 2.42 (m, 1H), 2.33 (d, J = 5.5 Hz, 6H), 2.11 (d, J = 3.0 Hz, 3H).

^{13}C NMR (125 MHz, Acetone-d_6): δ 154.9, 152.2, 145.9, 143.8, 143.1, 140.6, 138.6, 138.1, 137.4, 134.3, 133.7, 132.6, 131.8, 131.3, 126.9, 119.7, 118.0, 116.9, 116.3, 109.7, 55.2, 48.7, 33.6, 30.4, 22.8, 20.9, 20.7, 19.9.

HRMS (ESI) *m/z* calcd. for $C_{32}H_{33}NO_6S$ $(M+Na)^+$: 582.1921, found: 582.1910.

Enantiomeric excess of **2q** is determined by UPC^2 (CHIRALPAK® IG-3, CO_2/MeOH = 70/30, flow rate = 1.0 mL/min, 220 nm): major isomer: tr = 29.89 min; minor isomer: tr = 7.01 min. $[\alpha]^{20}_D$ = -77.13 (c = 1.0, MeOH).

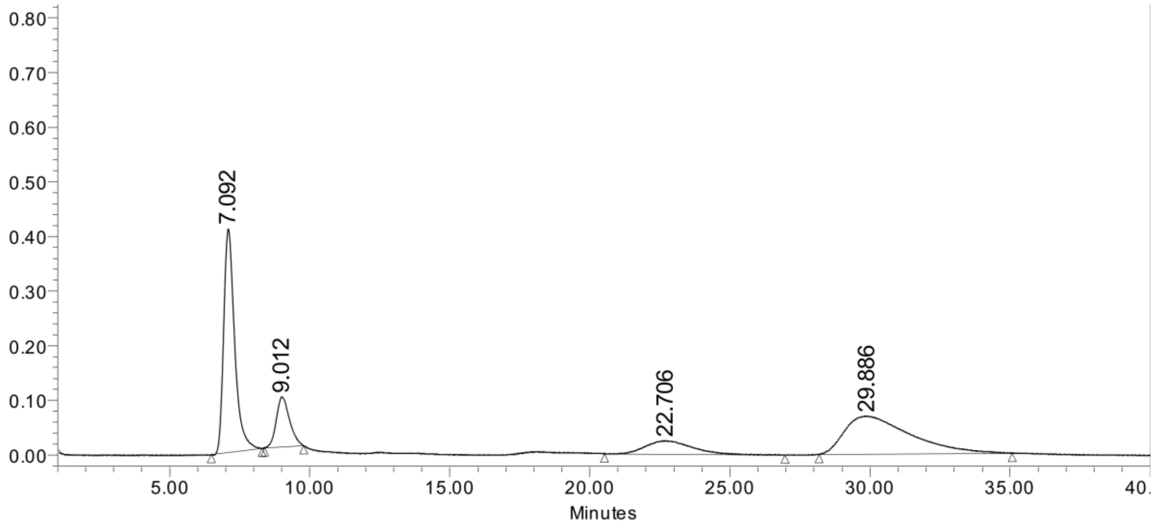

Signal: Sig = 220nm

Peak	RetTime	Area	Height	Area%
1	7.092	11101987	409200	39.32
2	9.012	3019488	91625	10.69
3	22.706	2822477	24404	10.00
4	29.886	11289133	69340	39.99

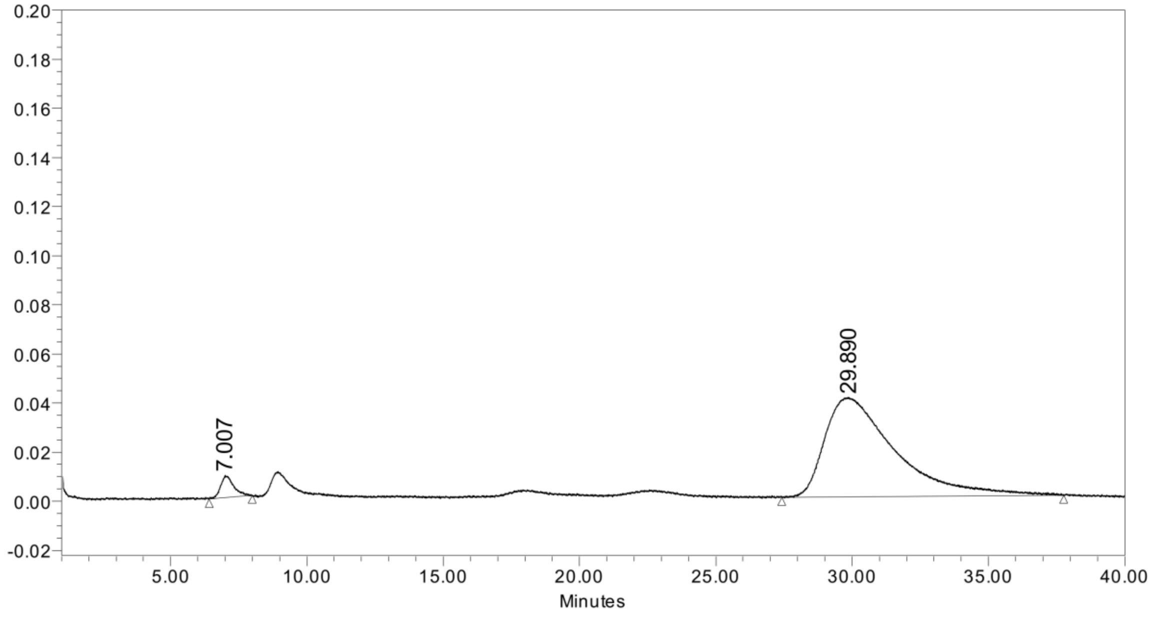

Peak	RetTime	Area	Height	Area%
1	7.007	299505	9029	3.98
2	29.890	7218623	40384	96.02

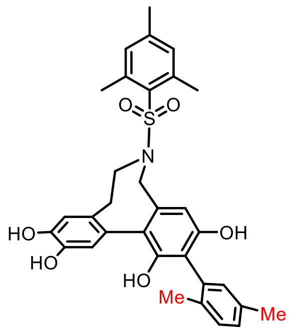

2r

According to the procedure, **2r** was obtained using **1r** (56.1 mg, 0.1 mmol) in 91% yield (51mg) , light gray foam solid, 80% ee, 8/1 dr, (silica gel flash chromatography: DCM/ acetone = 20:1). R_f = 0.40 (DCM/acetone = 10:1).

^{1}H NMR (500 MHz, Acetone-d_6): δ 7.93 (d, *J* = 13.0 Hz, 1H), 7.72 (s, 1H), 7.15 (t, *J* = 9.5 Hz, 1H), 7.10 – 7.02 (m, 3H), 6.99 (s, 1H), 6.85 – 6.71 (m, 2H), 6.65 (s, 1H), 6.51 (s, 1H), 4.72 (d, *J* = 14.0 Hz, 1H), 3.72 – 3.53 (m, 1H), 3.28 (d, *J* = 14.0 Hz, 1H), 3.01 (d, *J* = 13.0 Hz, 3H), 2.85 – 2.75 (m, 1H), 2.68 (t, *J* = 12.0 Hz, 1H), 2.58 (d, *J* = 7.0 Hz, 6H), 2.52 – 2.41 (m, 1H), 2.29 (d, *J* = 10.0 Hz, 6H), 2.18 (s, 3H).

^{13}C NMR (125 MHz, Acetone-d_6): δ 154.6, 154.5, 151.9, 151.8, 145.7, 143.6, 142.9, 140.5, 138.0, 135.4, 135.2, 134.1, 133.5, 132.4, 132.2, 130.2, 128.6, 126.7, 119.5, 119.4, 117.8, 116.7, 116.3,

116.2, 109.6, 109.5, 48.6, 33.4, 22.6, 20.7, 20., 20.5, 19.3, 19.1.

HRMS (ESI) *m/z* calcd. for $C_{32}H_{33}NO_6S$ $(M+Na)^+$: 582.1921, found: 582.1923.

Enantiomeric excess of **2r** is determined by HPLC (Chiralpak IA-AD-H, Hexane/Isopropanol = 50/50, flow rate = 1.0 mL/min, 220 nm): major isomer: tr = 27.99 min; minor isomer: tr = 30.54min. $[\alpha]^{20}_D$ = -69.53 (c = 1.0, MeOH).

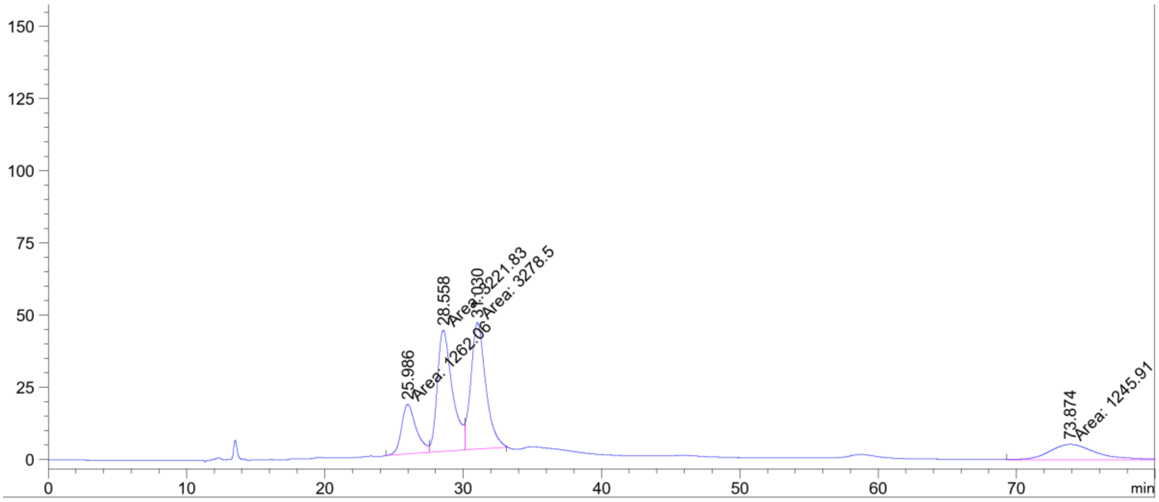

Signal 1: DAD1 B, Sig=254,4 Ref=off

Peak #	RetTime [min]	Type	Width [min]	Area [mAU*s]	Height [mAU]	Area %
1	25.986	MF	1.2245	1262.06042	17.17794	14.0100
2	28.558	MF	1.2769	3221.83350	42.05408	35.7652
3	31.030	FM	1.2489	3278.50146	43.75026	36.3942
4	73.874	MM	3.9075	1245.90735	5.31421	13.8307
Totals :				9008.30273	108.29649	

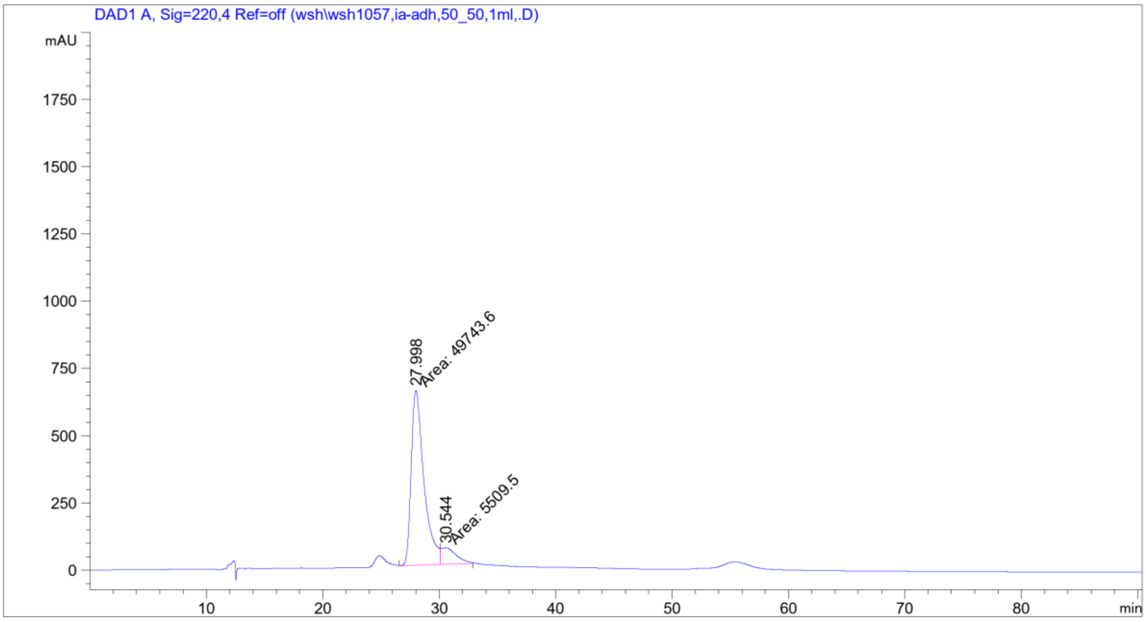

Signal 1: DAD1 A, Sig=220,4 Ref=off

Peak #	RetTime [min]	Type	Width [min]	Area [mAU*s]	Height [mAU]	Area %
1	27.998	MF	1.2753	4.97436e4	650.09485	90.0286
2	30.544	FM	1.5004	5509.50098	61.20063	9.9714
Totals :				5.52531e4	711.29548	

（We attempted the following chiral columns such as OD-H, AD-H, AS-H, AY-H, IA, IB, IC, ID, IE, IF, IG, IJ, OJ-H, AD but were unable to obtain satisfactory result）.

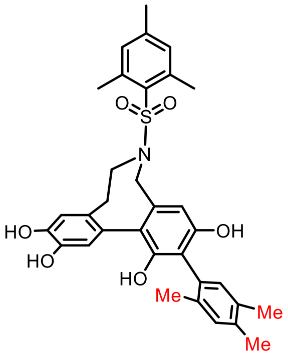

2s

According to the procedure, **2s** was obtained using **1s** (57.5 mg, 0.1 mmol) in 91% yield (52.4mg) , light gray foam solid, 95% ee, 10/1 dr, (silica gel flash chromatography: DCM/ acetone = 20:1). R_f = 0.40 (DCM/acetone = 10:1).

^{1}H NMR (500 MHz, Acetone-d_6): δ 7.91 (s, 1H), 7.61 (s, 1H), 7.05 (d, *J* = 7.0 Hz, 3H), 6.92 (s, 1H), 6.79 (d, *J* = 18.5 Hz, 2H), 6.63 (s, 1H), 6.46 (s, 1H), 4.71 (d, *J* = 14.0 Hz, 1H), 3.65 (dd, *J* = 12.5, 7.0 Hz, 1H), 3.27 (d, *J* = 14.0 Hz, 1H), 3.02 (d, *J* = 13.0 Hz, 3H), 2.77 (dd, *J* = 14.0, 7.0 Hz, 1H), 2.68 (t, *J* = 11.5 Hz, 1H), 2.58 (d, *J* = 7.5 Hz, 6H), 2.51 – 2.43 (m, 1H), 2.30 (s, 3H), 2.23 (s, 3H), 2.19 (s, 3H), 2.15 (s, 3H).

^{13}C NMR (125 MHz, Acetone-d_6): δ 154.7, 151.9, 151.8, 145.6, 143.5, 142.9, 140.5, 137.9, 135.9, 135.8, 134.0, 133.8, 133.5, 132.8, 132.4, 131.8, 131.2, 126.9, 119.5, 119.4, 117.9, 116.6, 109.5, 48.5, 33.4, 22.7, 20.5, 19.2, 19.1, 18.9.

HRMS (ESI) *m/z* calcd. for $C_{33}H_{35}NO_6S$ $(M+Na)^+$: 596.2077, found: 596.2067.

Enantiomeric excess of **2s** is determined by UPC^2 (CHIRALPAK® IG-3, CO_2/MeOH = 65/35, flow rate = 0.5 mL/min, 220 nm): major isomer: tr =14.62 min; minor isomer: tr = 7.49 min. $[\alpha]^{20}_D$ = -103.33(c = 1.0, MeOH)

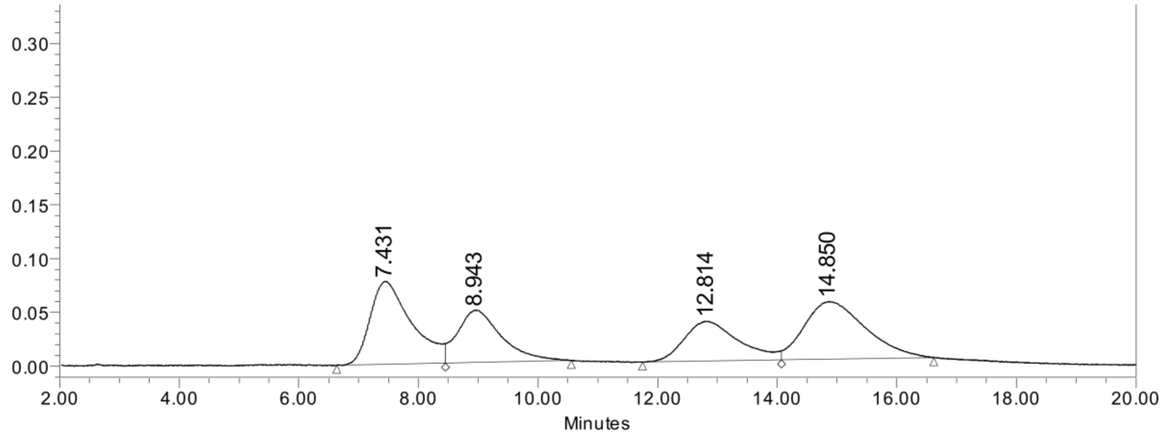

Signal: Sig = 220nm

Peak	RetTime	Area	Height	Area%
1	7.431	3710393	76931	30.50
2	8.943	2477577	48530	19.50
3	12.814	2424174	37598	19.08
4	14.850	3761487	54858	30.92

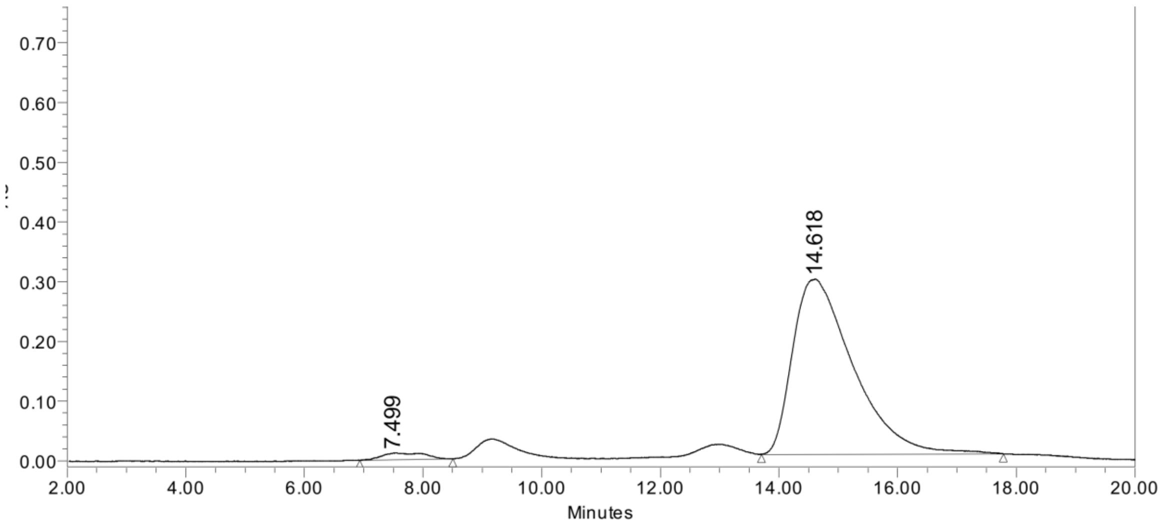

Peak	RetTime	Area	Height	Area%
1	7.499	541979	11013	2.54
2	14.618	20801753	293066	97.46

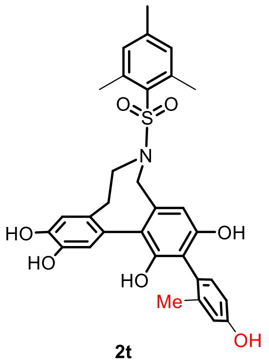

2t

According to the procedure, **2t** was obtained using **1t** (56.3 mg, 0.1 mmol) in 85% yield (47.7mg), light gray foam solid, 94 ee, 10/1 dr, (silica gel flash chromatography: DCM/ acetone = 20:1). R_f = 0.40 (DCM/acetone = 10:1).

^{1}H NMR (500 MHz, CD_3OD): δ 7.03 (s, 2H), 6.97 (d, *J* = 8.0 Hz, 1H), 6.77 (d, *J* = 2.0 Hz, 1H), 6.74 (d, *J* = 5.0 Hz, 1H), 6.72 – 6.65 (m, 2H), 6.55 (s, 1H), 4.68 (d, *J* = 14.0 Hz, 1H), 3.57 (dd, *J* = 12.5, 7.0 Hz, 1H), 3.24 (d, *J* = 14.0 Hz, 1H), 2.66 (dd, *J* = 14.0, 9.5 Hz, 2H), 2.55 (d, *J* = 6.5 Hz, 6H), 2.49 – 2.37 (m, 2H), 2.30 (s, 3H), 2.13 (s, 3H).

^{13}C NMR (125 MHz, CD_3OD): δ 157.9, 155.5, 152.7, 146.5, 144.4, 144.3, 141.5, 140.8, 138.3, 133.9, 133.9, 133.3, 133.2, 127.7, 125.5, 120.6, 118.7, 117.8, 117.1, 113.9, 110.1, 49.3, 34.1, 23.1, 21.0, 20.2.

HRMS (ESI) *m/z* calcd. for $C_{31}H_{31}NO_7S$ $(M+Na)^+$: 584.1713, found: 584.1714.

Enantiomeric excess of **2t** is determined by HPLC (Chiralpak IE, Hexane/Isopropanol = 50/50, flow rate = 1.0 mL/min, 300 nm): major isomer: tr = 10.25; minor isomer: tr = 6.39n. $[\alpha]^{20}{}_{D}$= - 33.40 (c = 1.0, $CHCl_3$)

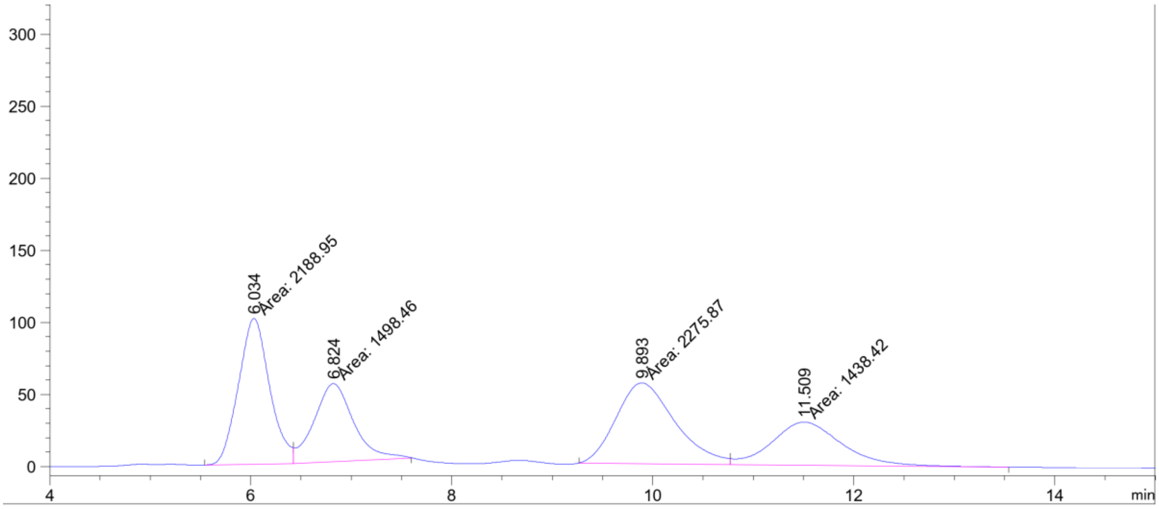

Signal 1: DAD1 E, Sig=300,4 Ref=off

Peak #	RetTime [min]	Type	Width [min]	Area [mAU*s]	Height [mAU]	Area %
1	6.034	MM	0.3601	2188.94702	101.30457	29.5736
2	6.824	MM	0.4600	1498.45618	54.29202	20.2448
3	9.893	MF	0.6781	2275.86621	55.93673	30.7479
4	11.509	FM	0.8028	1438.41907	29.86384	19.4337

Totals : 7401.68848 241.39716

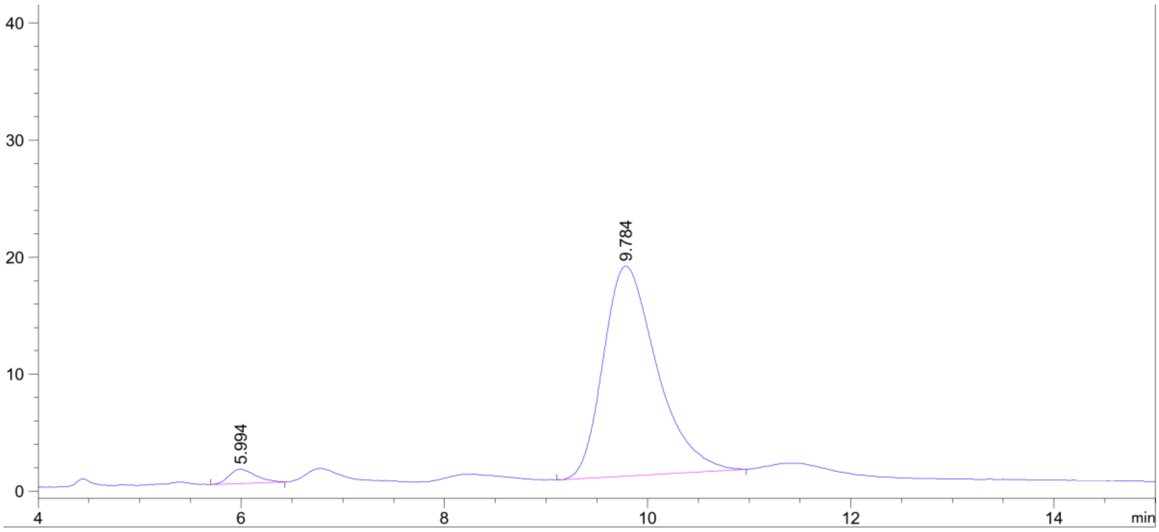

Signal 1: DAD1 E, Sig=300,4 Ref=off

Peak #	RetTime [min]	Type	Width [min]	Area [mAU*s]	Height [mAU]	Area %
1	5.994	BB	0.2364	21.89911	1.22619	3.2225
2	9.784	BB	0.5359	657.65942	17.93208	96.7775
Totals :				679.55854	19.15827	

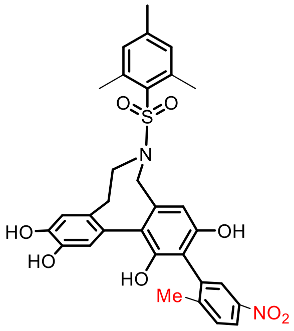

2u

According to the procedure, **2u** was obtained using **1u** (59.2 mg, 0.1 mmol) in 88% yield (52mg) , light gray foam solid, 94% ee, 99% ee, 2/1 dr, (silica gel flash chromatography: DCM/ acetone = 20:1). R_f = 0.40 (DCM/acetone = 10:1).

^{1}H NMR (500 MHz, Acetone-d_6): δ 8.16 – 8.04 (m, 2H), 7.58 – 7.52 (m, 1H), 7.06 (d, J = 5.0 Hz, 2H), 6.85 – 6.71 (m, 2H), 4.76 (d, J = 14.0 Hz, 1H), 3.68 – 3.56 (m, 1H), 3.35 – 3.23 (m, J = 14.0, 6.0 Hz, 1H), 2.78 (dd, J = 14.0, 7.0 Hz, 1H), 2.70 (s, 1H), 2.58 (d, J = 6.0 Hz, 4H), 2.51 – 2.41 (m, 1H), 2.39 (s, 1H), 2.32 – 2.28 (m, 3H).

^{13}C NMR (125MHz, Acetone- d_6): δ 154.9, 152.3, 147.5, 147.3, 146.9, 146.2, 146.2, 144.1, 144.1, 143.2, 140.7, 140.7, 139.4, 137.4, 137.3, 134.3, 134.2, 134.2, 134.1, 132.7, 131.3, 126.9, 126.9, 126.2, 126.1, 122.6, 122.6, 120.2, 117.8, 117.3, 117.2, 114.6, 110.1, 109.9, 48.9, 48.8, 33.8, 30.5, 22.9, 22.9, 20.8, 20.2, 20.1.

HRMS (ESI) m/z calcd. for $C_{31}H_{30}N_2O_8S$ $(M+Na)^+$: 613.1615, found: 613.1608.

Enantiomeric excess of **2u** is determined by UPC^2 (CHIRALPAK® AD-3, CO_2/MeOH = 80/20, flow rate = 1.0 mL/min, 220 nm): major isomer: tr = 14.91 min; minor isomer: tr = 6.49 min. major isomer: tr = 5.14 min; minor isomer: tr = 24.71 min. $[\alpha]^{20}_D$ = 33.13 (c = 1.0, MeOH).

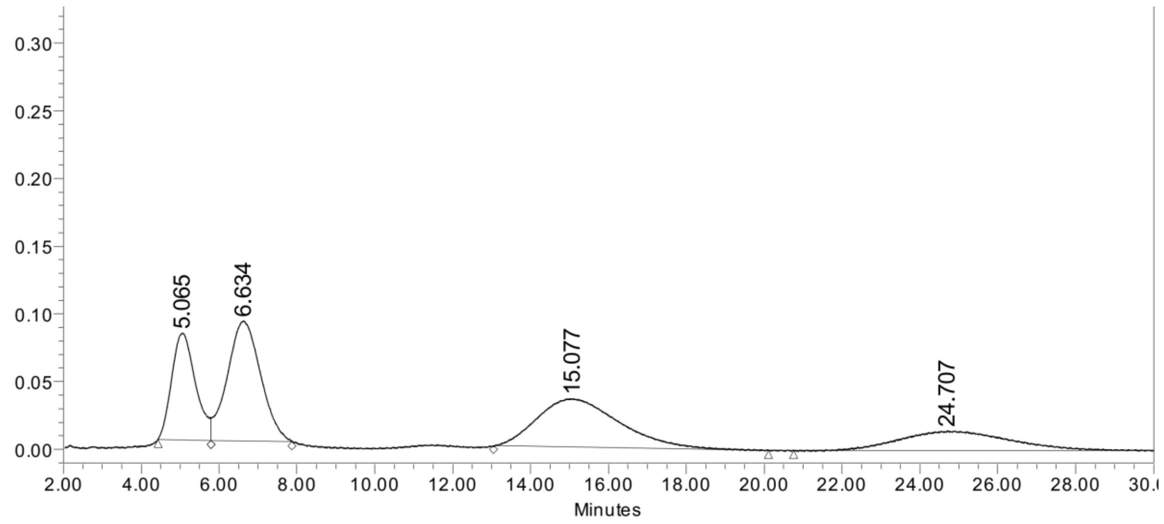

Signal: Sig = 220nm

Peak	RetTime	Area	Height	Area%
1	5.065	2910370	73319	19.24
2	6.634	4695411	83864	31.04
3	15.077	4689939	33499	31.00
4	24.707	2831403	14482	18.72

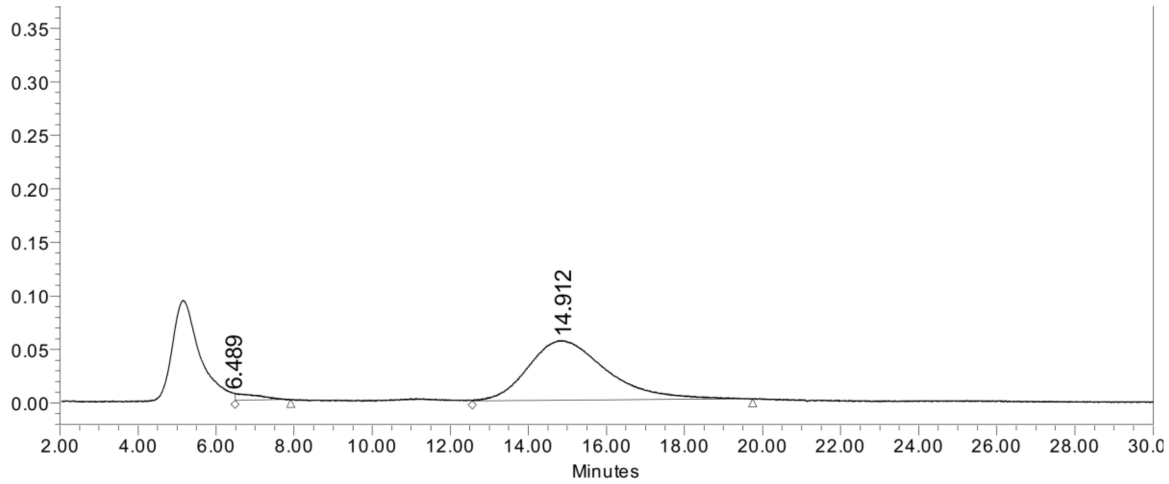

Peak	RetTime	Area	Height	Area%
1	6.489	252861	6237	3.24
2	14.912	7550638	55141	96.76

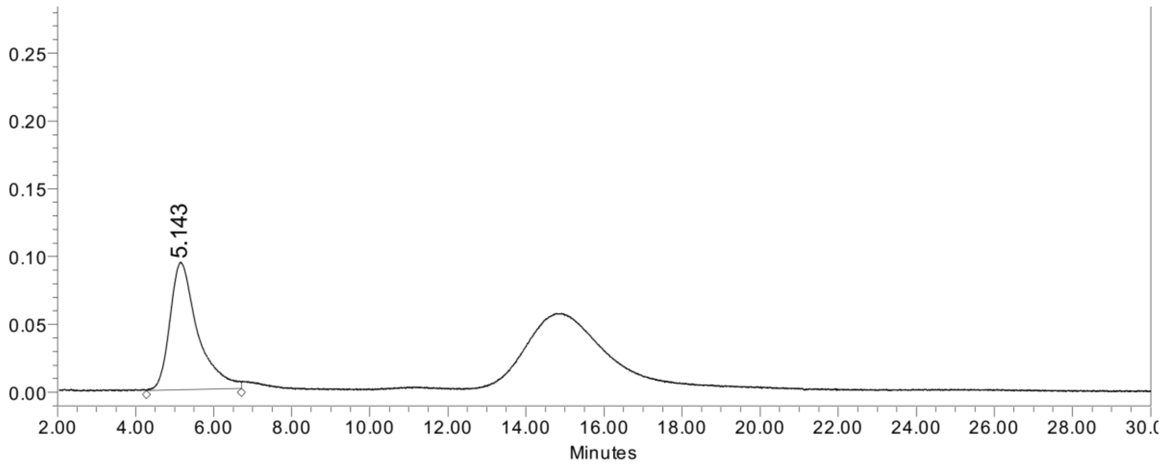

Peak	RetTime	Area	Height	Area%
1	5.143	4628139	93767	100.00

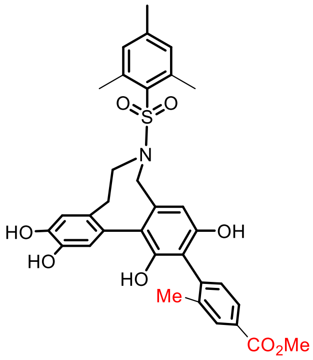

2v

According to the procedure, **2v** was obtained using **1v** (60.5 mg, 0.1 mmol) in 88% yield (53mg) , light gray foam solid, 99% ee, >20/1 dr, (silica gel flash chromatography: DCM/ acetone = 20:1). R_f = 0.40 (DCM/acetone = 10:1).

^{1}H NMR (500 MHz, Acetone-d_6): δ 7.92 (s, 1H), 7.83 (dd, J = 8.0, 1.5 Hz, 1H), 7.32 (d, J = 8.0 Hz, 1H), 7.06 (s, 2H), 6.79 (d, J = 6.0 Hz, 2H), 6.74 (s, 1H), 4.75 (d, J = 14.0 Hz, 1H), 3.88 (s, 3H), 3.62 (dd, J = 13.0, 7.0 Hz, 1H), 3.28 (d, J = 14.0 Hz, 1H), 3.05 (s, 2H), 2.77 (dd, J = 14.0, 6.9 Hz, 1H), 2.72 – 2.63 (m, 1H), 2.58 (s, 6H), 2.46 (dd, J = 14.0, 10.5 Hz, 1H), 2.32 (s, 3H), 2.30 (s, 3H).

^{13}C NMR (125 MHz, Acetone-d_6): δ 167.1, 154.5, 151.8, 145.9, 143.8, 142.9, 140.5, 140.4, 139.0, 138.6, 134.0, 133.8, 132.4, 132.1, 130.9, 129.5, 126.8, 126.2, 119.7, 117.6, 116.9, 115.5, 109.7, 51.8, 48.6, 48.6, 33.5, 30.2, 22.6, 20.5, 19.7.

HRMS (ESI) *m/z* calcd. for $C_{33}H_{33}NO_8S$ $(M+Na)^+$: 626.1819, found: 626.1812.

Enantiomeric excess of **2v** is determined by HPLC (Chiralpak IE-IE-3, Hexane/Isopropanol =

50/50, flow rate = 0.5 mL/min, 220 nm): major isomer: tr = 58.01 min; minor isomer: tr = 75.96 min. $[\alpha]^{20}_{D}$ = -77.33 (c = 1.0, MeOH).

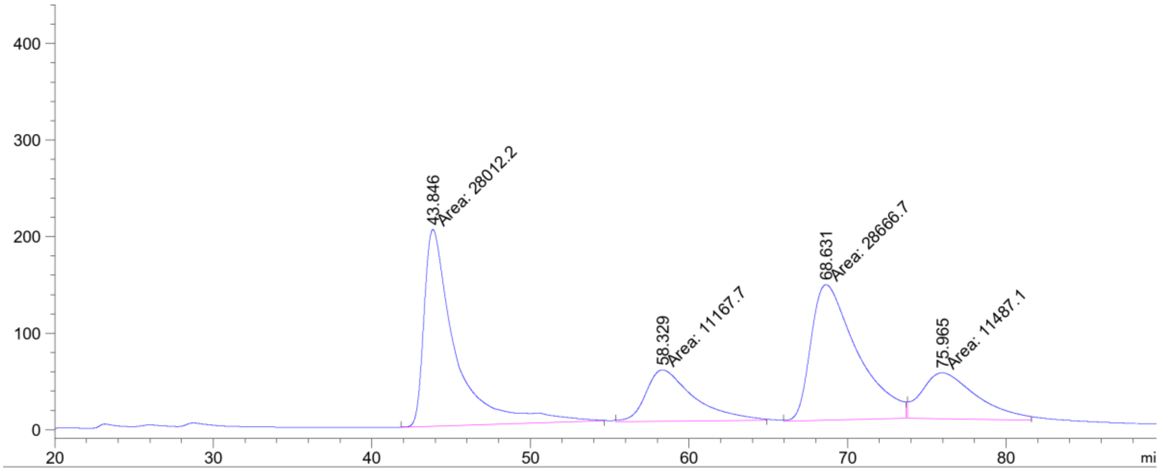

Signal 1: DAD1 A, Sig=220,4 Ref=off

Peak #	RetTime [min]	Type	Width [min]	Area [mAU*s]	Height [mAU]	Area %
1	43.846	MM	2.2895	2.80122e4	203.91524	35.3093
2	58.329	MM	3.4935	1.11677e4	53.27911	14.0769
3	68.631	MM	3.4040	2.86667e4	140.35733	36.1343
4	75.965	MM	4.0305	1.14871e4	47.50103	14.4795

Totals : 7.93338e4 445.05271

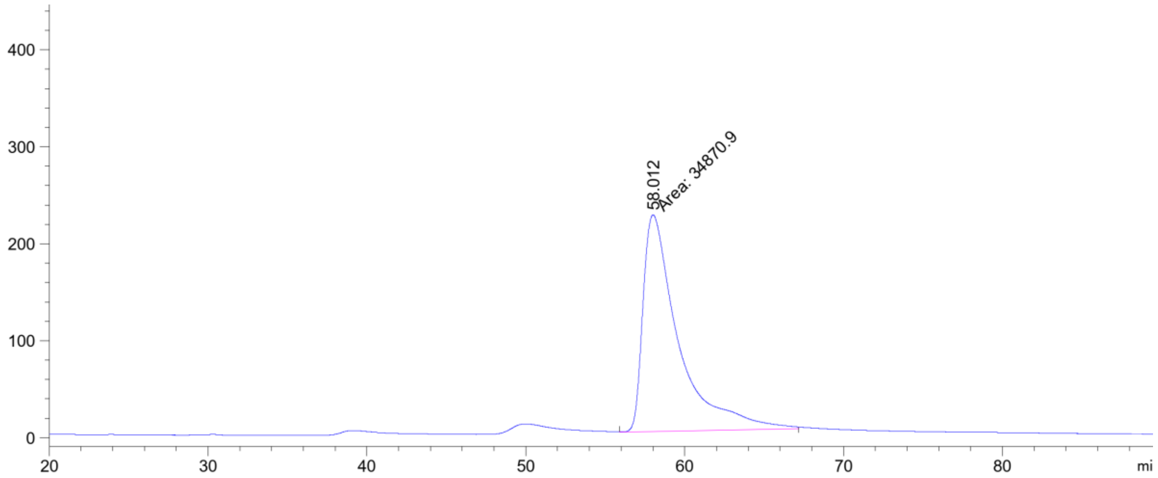

Signal 1: DAD1 A, Sig=220,4 Ref=off

Peak #	RetTime [min]	Type	Width [min]	Area [mAU*s]	Height [mAU]	Area %
1	58.012	MM	2.5994	3.48709e4	223.58121	100.0000

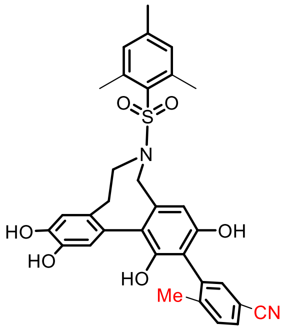

2w

According to the procedure, **2w** was obtained using **1w** (57.2 mg, 0.1 mmol) in 87% yield (50mg), light gray foam solid, 99% ee, 2/1 dr, (silica gel flash chromatography: DCM/ acetone = 20:1). R_f = 0.40 (DCM/acetone = 10:1).

^{1}H NMR (500 MHz, Acetone-d_6): δ 7.64 – 7.59 (m, 1H), 7.56 (s, 1H), 7.53 – 7.45 (m, 1H), 7.06 (d, J = 5.5 Hz, 2H), 6.83 – 6.71 (m, 3H), 4.75 (d, J = 14.5 Hz, 1H), 3.61 (dd, J = 13.0, 6.5 Hz, 1H), 3.28 (d, J = 14.5 Hz, 1H), 3.00 (s, 3H), 2.84 – 2.74 (m, 1H), 2.72 – 2.62 (m, 1H), 2.57 (d, J = 6.5 Hz, 6H), 2.46 (dd, J = 13.5, 10.5 Hz, 1H), 2.30 (s, 3H), 2.09 (s, 3H).

^{13}C NMR (125 MHz, Acetone-d_6): δ 206.0, 154.7, 152.0, 145.9, 144.9, 144.7, 143.9, 143.0, 140.5, 139.0, 136.9, 135.4, 135.4, 134.1, 133.9, 133.9, 132.5, 131.2, 131.1, 126.1, 119.9, 119.4, 117.6, 117.0, 114.4, 109.8, 109.7, 109.69, 48.7, 48.6, 33.5, 30.3, 22.7, 22.7, 20.5, 20.2.

HRMS (ESI) *m/z* calcd. for $C_{32}H_{30}N_2O_6S$ $(M+Na)^+$: 593.1717, found: 593.1704.

Enantiomeric excess of **2w** is determined by UPC^2 (CHIRALPAK® IG-3, Hexane/Isopropanol = 75/25, flow rate = 1.0 mL/min, 220 nm): major isomer: tr = 29.73 min; minor isomer: tr = 33.77 min. $[\alpha]^{20}_{D}$ = 17.8 (c = 1.0, MeOH).

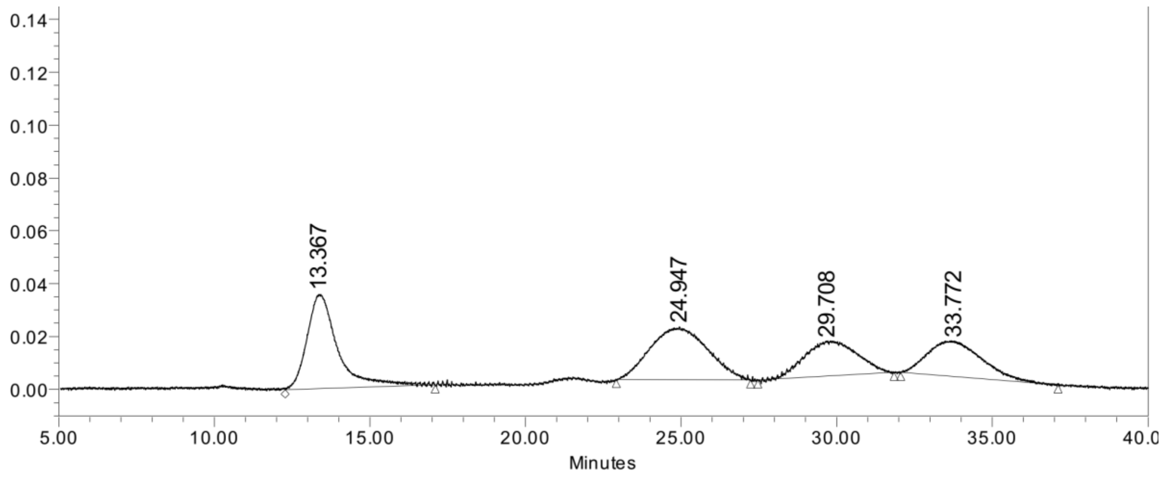

Signal: Sig = 220nm

Peak	RetTime	Area	Height	Area%
1	13.367	2460936	35682	30.32
2	24.947	2509582	19967	30.92
3	29.708	1542409	13188	19.01
4	33.772	1602386	13316	19.75

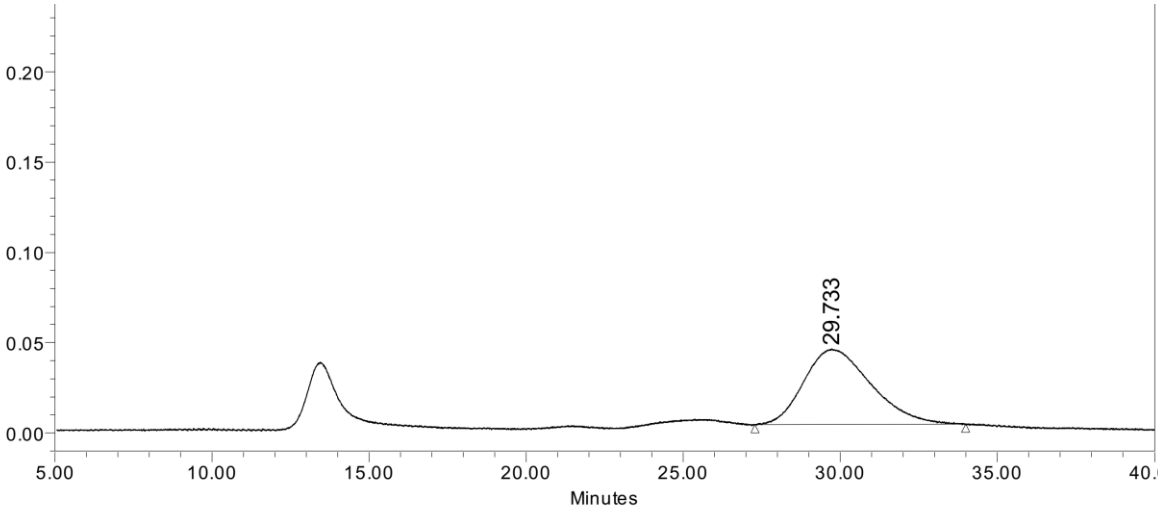

Peak	RetTime	Area	Height	Area%
1	29.733	5956712	41186	100.00

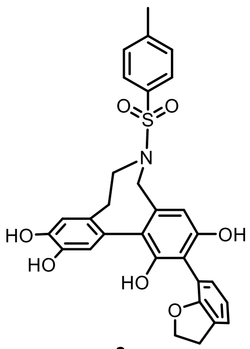

2x

According to the procedure, **2x** was obtained using **1x** (54.7mg, 0.1 mmol) in 89% yield (48.5mg) , light gray foam solid, 80% ee, >20/1 dr, (silica gel flash chromatography: DCM/ acetone = 20:1). R_f = 0.40 (DCM/acetone = 10:1).

^{1}H NMR (500 MHz, CD_3OD): δ 7.64 (d, *J* = 8.0 Hz, 2H), 7.33 (d, *J* = 8.0 Hz, 2H), 7.18 (d, *J* = 7.0 Hz, 1H), 7.16 – 7.01 (m, 1H), 6.88 (s, 2H), 6.73 (d, *J* = 14.0 Hz, 1H), 6.64 (d, *J* = 10.0 Hz, 1H), 4.61 (d, *J* = 14.0 Hz, 1H), 4.53 (s, 2H), 3.97 (s, 1H), 3.24 (d, *J* = 8.0 Hz, 2H), 2.95 (d, *J* = 14.0 Hz, 1H), 2.72 (d, *J* = 7.0 Hz, 1H), 2.47 (t, *J* = 11.0 Hz, 1H), 2.43 – 2.28 (m, 4H).

^{13}C NMR (125 MHz, CD_3OD): δ 159.3, 155.9, 152.8, 146.4, 144.8, 144.5, 138.6, 137.1, 133.7, 131.9, 130.8, 128.5, 128.3, 127.7, 125.2, 121.4, 118.6, 117.2, 117.1, 110.3, 71.9, 51.1, 50.5, 33.9, 30.8, 21.4.

HRMS (ESI) *m/z* calcd. for $C_{30}H_{27}NO_7S$ $(M+Na)^+$: 568.1400, found: 568.1401.

Enantiomeric excess of **2x** is determined by HPLC (Chiralpak AD-H, Hexane/Isopropanol = 60/40, flow rate = 1.0 mL/min, 220 nm): major isomer: tr = 23.61 min; minor isomer: tr = 12.33 min. $[\alpha]^{20}_D$ = -40.67 (c = 1.0, $CHCl_3$).

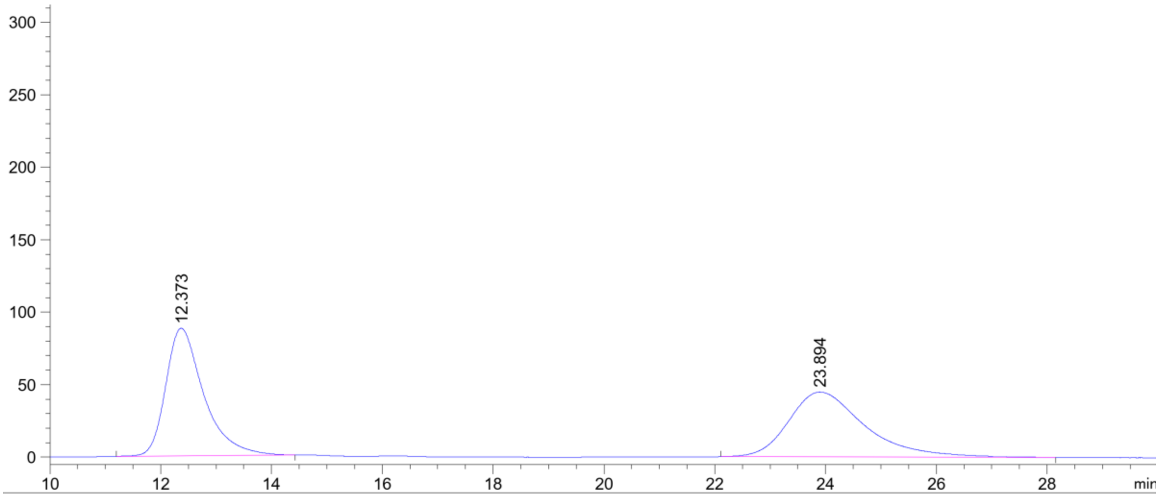

Signal 1: DAD1 A, Sig=220,4 Ref=off

Peak #	RetTime [min]	Type	Width [min]	Area [mAU*s]	Height [mAU]	Area %
1	12.373	BB	0.7099	4193.85547	88.01886	49.5090
2	23.894	BB	1.1612	4277.03271	44.74532	50.4910

Totals : 8470.88818 132.76418

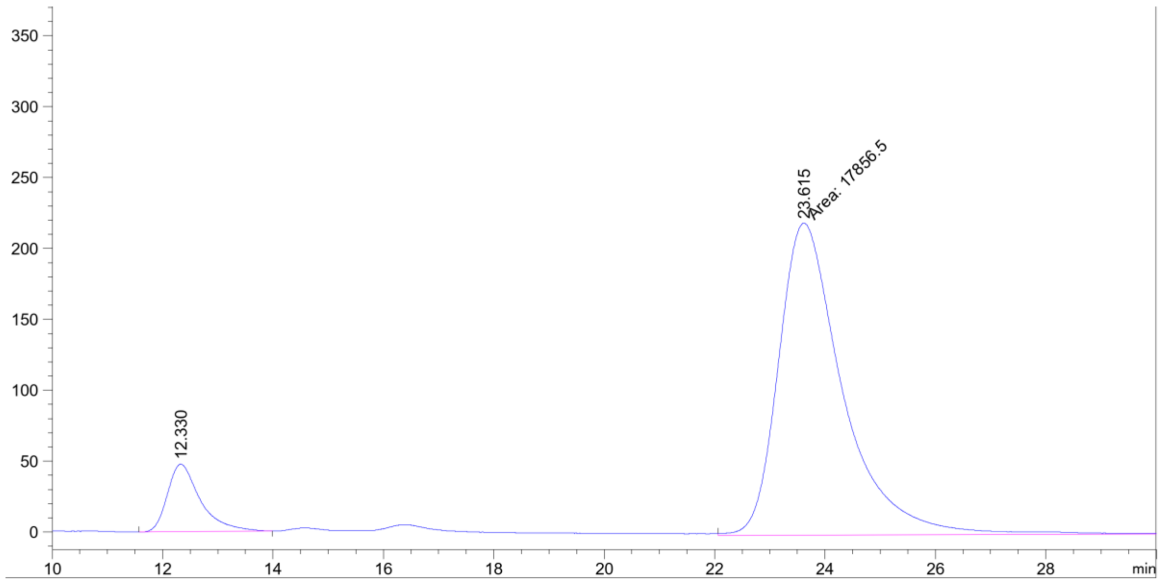

Signal 1: DAD1 E, Sig=220,4 Ref=off

Peak #	RetTime [min]	Type	Width [min]	Area [mAU*s]	Height [mAU]	Area %
1	12.330	BB	0.5996	1977.98279	47.71574	9.9724
2	23.615	MM	1.3523	1.78565e4	220.07388	90.0276
Totals :				1.98345e4	267.78962	

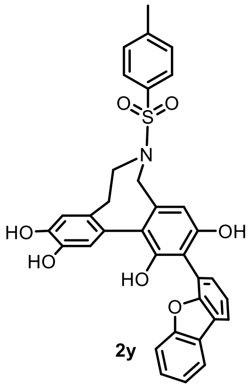

2y

According to the procedure, **2y** was obtained using **1y** (59.5mg, 0.1 mmol) in 99% yield (58.7mg) , light gray foam solid, 81% ee, >20/1 dr, (silica gel flash chromatography: DCM/ acetone = 20:1). R_f = 0.40 (DCM/acetone = 10:1).

^{1}H NMR (500 MHz, CD_3OD): δ 8.02 (dd, *J* = 16.0, 7.5 Hz, 2H), 7.67 (d, *J* = 8.0 Hz, 2H), 7.58 – 7.48 (m, 2H), 7.48 – 7.38 (m, 3H), 7.34 (t, *J* = 8.0 Hz, 3H), 7.01 (d, *J* = 4.0 Hz, 1H), 6.83 (d, *J* = 28.0 Hz, 1H), 6.70 (s, 1H), 4.69 (d, *J* = 14.0 Hz, 1H), 4.03 (dd, *J* = 11.0, 7.0 Hz, 1H), 3.03 (dd, *J* = 14.0, 3.0 Hz, 1H), 2.78 (dd, *J* = 13.5, 7.0 Hz, 1H), 2.60 – 2.43 (m, 2H), 2.38 (d, *J* = 3.0 Hz, 3H).

^{13}C NMR (125 MHz, CD_3OD): δ 157.4, 156.4, 156.2, 156.0, 153.2, 146.6, 144.8, 144.6, 139.2, 137.1, 137.1, 134.1, 133.9, 131.3, 131.3, 130.8, 128.2, 128.0, 127.2, 125.8, 125.4, 123.7, 121.6, 120.7, 120.4, 118.4, 117.5, 113.0, 112.9, 112.5, 112.5, 110.3, 51.1, 50.7, 34.1, 21.4.

HRMS (ESI) *m/z* calcd. for $C_{34}H_{27}NO_7S$ $(M+Na)^+$: 616.1400, found: 616.1386.

Enantiomeric excess of **2y** is determined by HPLC (Chiralpak AD-H, Hexane/Isopropanol = 40/60, flow rate = 1.0 mL/min, 220 nm): major isomer: tr = 17.02 min; minor isomer: tr = 8.23 min. $[\alpha]^{20}_D$ = -240.53 (c = 1.0, MeOH)

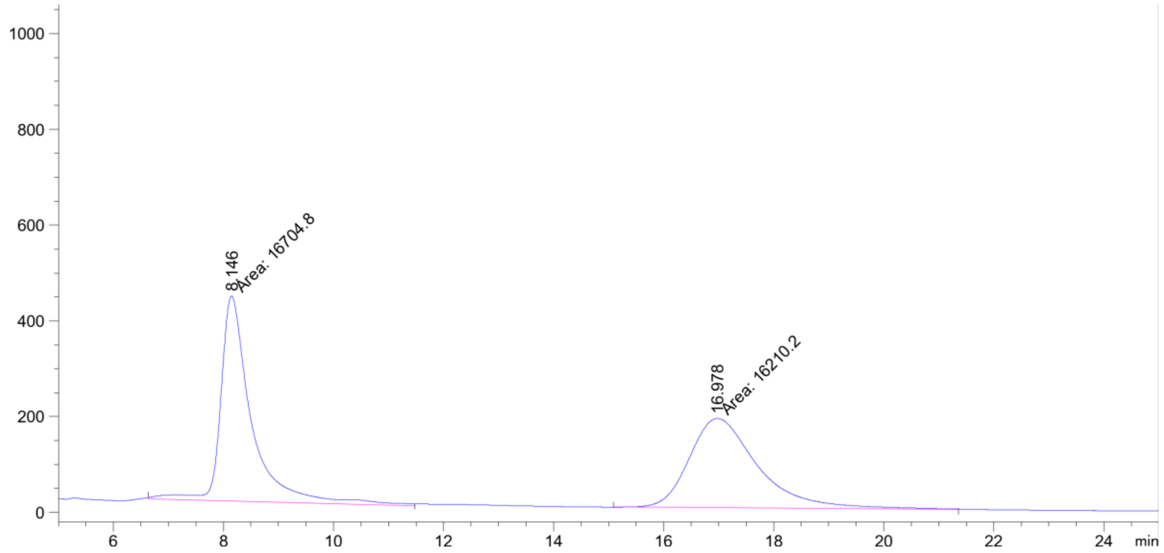

Signal 1: DAD1 A, Sig=220,4 Ref=off

Peak #	RetTime [min]	Type	Width [min]	Area [mAU*s]	Height [mAU]	Area %
1	8.146	MM	0.6502	1.67048e4	428.20221	50.7513
2	16.978	MM	1.4476	1.62102e4	186.63850	49.2487
Totals :				3.29149e4	614.84071	

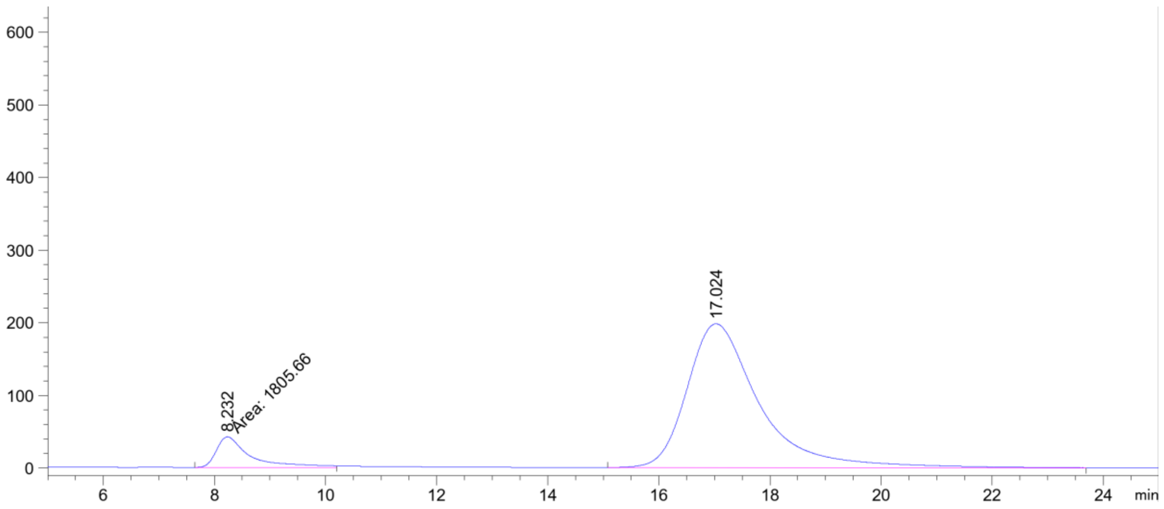

Signal 1: DAD1 A, Sig=220,4 Ref=off

Peak #	RetTime [min]	Type	Width [min]	Area [mAU*s]	Height [mAU]	Area %
1	8.232	MM	0.7143	1805.65784	42.13018	9.1415
2	17.024	BB	1.2806	1.79467e4	198.16801	90.8585
Totals :				1.97524e4	240.29819	

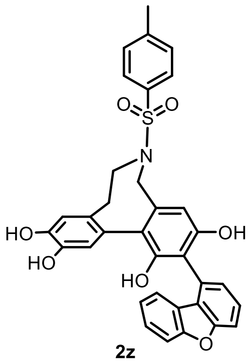

2z

According to the procedure, **2z** was obtained using **1z** (59.5mg, 0.1 mmol) in 93% yield (55mg) , light gray foam solid, 95% ee, >20/1 dr, (silica gel flash chromatography: DCM/ acetone = 20:1). R_f = 0.40 (DCM/acetone = 10:1).

^{1}H NMR (500 MHz, CD_3OD): δ 7.68 (d, *J* = 8.0 Hz, 2H), 7.60 – 7.48 (m, 3H), 7.41 (d, *J* = 8.0 Hz, 2H), 7.31 (dd, *J* = 10.5, 8.0 Hz, 3H), 7.19 (s, 1H), 7.08 (s, 1H), 6.80 (s, 1H), 6.71 (s, 1H), 4.74 (d, *J* = 13.5 Hz, 1H), 4.05 (dd, *J* = 11.5, 7.0 Hz, 1H), 3.08 (d, *J* = 14.0 Hz, 1H), 2.80 (dd, *J* = 14.0, 7.0 Hz, 1H), 2.50 (dd, *J* = 21.0, 12.5 Hz, 2H), 2.38 (s, 3H).

^{13}C NMR (125 MHz, CD_3OD): δ 157.7, 157.5, 156.0, 152.9, 146.7, 144.8, 144.6, 139.3, 137.1,

133.9, 130.8, 128.2, 127.9, 127.8, 127.2, 126.8, 125.8, 125.1, 123.6, 123.0, 120.7, 118.4, 117.5, 115.4, 112.1, 111.2, 110.3, 51.1, 50.7, 34.1, 21.4.

HRMS (ESI) *m/z* calcd. for $C_{34}H_{27}NO_7S$ $(M+Na)^+$: 616.1400, found: 616.1391.

Enantiomeric excess of **2z** is determined by UPC^2 (CHIRALPAK® IG-3, CO_2/MeOH = 60/40, flow rate = 1.0 mL/min, 220 nm): major isomer: tr = 5.22 min; minor isomer: tr = 4.55 min. $[\alpha]^{20}_D$ = -548.33 (c = 1.0, MeOH)

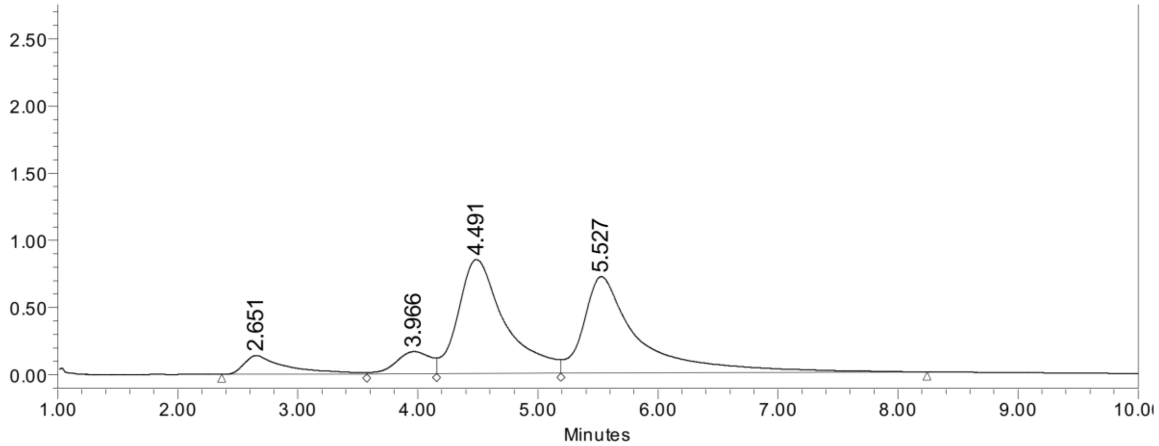

Signal: Sig = 220nm

Peak	RetTime	Area	Height	Area%
1	2.651	3441497	140060	6.51
2	3.966	3284702	165156	6.22
3	4.491	23036507	848290	43.59
4	5.527	23080414	717533	43.68

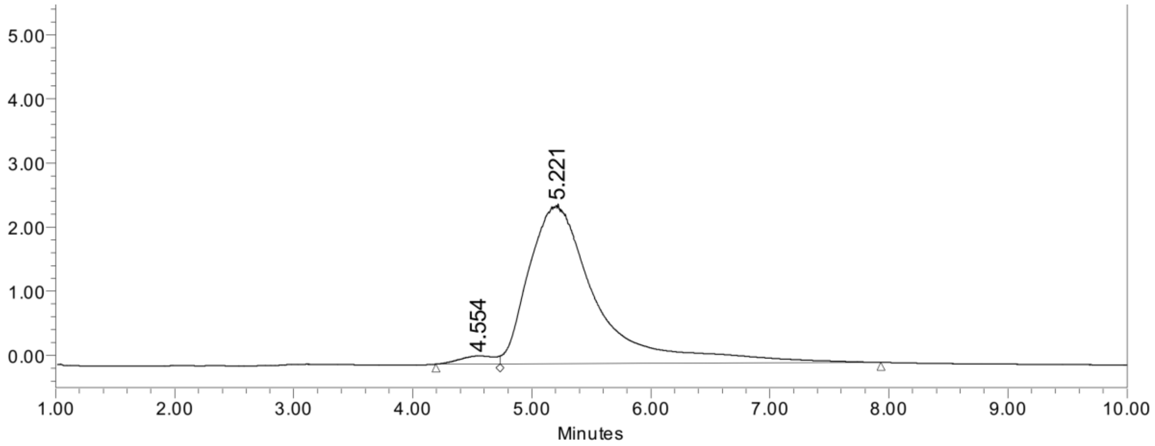

Peak	RetTime	Area	Height	Area%
1	4.554	2624207	124358	2.61
2	5.221	98101888	2488176	97.39

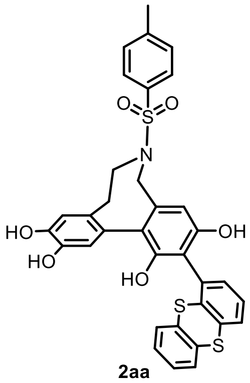

2aa

According to the procedure, **2aa** was obtained using **1aa** (64.3 mg, 0.1 mmol) in 82% yield (52.5mg), light gray foam solid, 96% ee, 4/1 dr, (silica gel flash chromatography: DCM/ acetone = 20:1). R_f = 0.40 (DCM/acetone = 10:1).

^{1}H NMR (500 MHz, CD_3OD): δ 7.71 – 7.61 (m, 2H), 7.49 – 7.43 (m, 2H), 7.35 (dd, *J* = 15.0, 7.0 Hz, 1H), 7.32 – 7.14 (m, 7H), 6.93 (d, *J* = 3.5 Hz, 1H), 6.83 (s, 1H), 6.68 (s, 1H), 4.67 – 4.57 (m, 1H), 4.05 – 3.92 (m, 1H), 2.99 (t, *J* = 12.5 Hz, 1H), 2.75 (dd, *J* = 14.0, 7.0 Hz, 1H), 2.53 – 2.44 (m, 1H), 2.44 – 2.28 (m, 4H).

^{13}C NMR (125 MHz, CD_3OD): δ 155.8, 152.8, 146.7, 144.8, 144.7, 139.1, 138.4, 137.9, 137.6, 137.1, 136.5, 136.3, 133.9, 132.1, 130.9, 130.8, 129.8, 129.2, 128.6, 128.6, 128.2, 128.0, 127.1, 120.3, 118.4, 117.5, 116.4, 110.01, 51.1, 50.7, 49.5, 34.1, 21.5.

HRMS (ESI) *m/z* calcd. for $C_{34}H_{27}NO_6S_3$ $(M+Na)^+$: 664.0893, found: 664.0880.

Enantiomeric excess of **2aa** is determined by HPLC (Chiralpak IG, Hexane/Isopropanol = 60/40, flow rate = 1.0 mL/min, 220 nm): major isomer: tr = 50.37min; minor isomer: tr = 20.16 min. $[\alpha]^{20}_{D}$= -93.07 (c = 1.0, acetone)

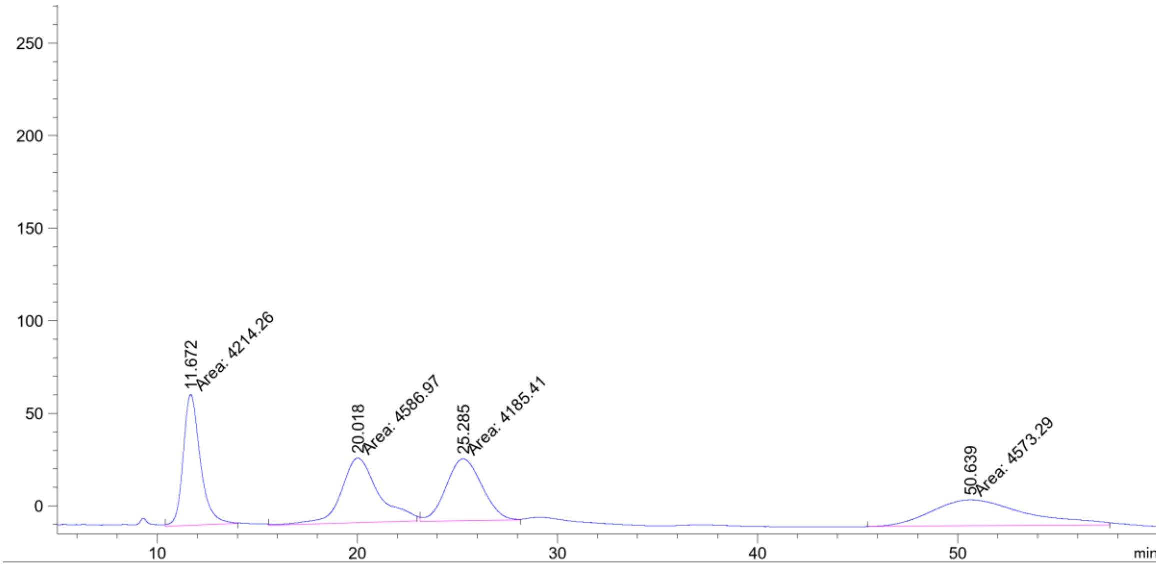

Signal 1: DAD1 A, Sig=220,4 Ref=off

Peak #	RetTime [min]	Type	Width [min]	Area [mAU*s]	Height [mAU]	Area %
1	11.672	MM	0.9919	4214.25684	70.81431	23.9993
2	20.018	MM	2.1851	4586.97461	34.98613	26.1218
3	25.285	MM	2.0796	4185.41211	33.54390	23.8350
4	50.639	MM	5.4416	4573.28857	14.00715	26.0439

Totals : 1.75599e4 153.35149

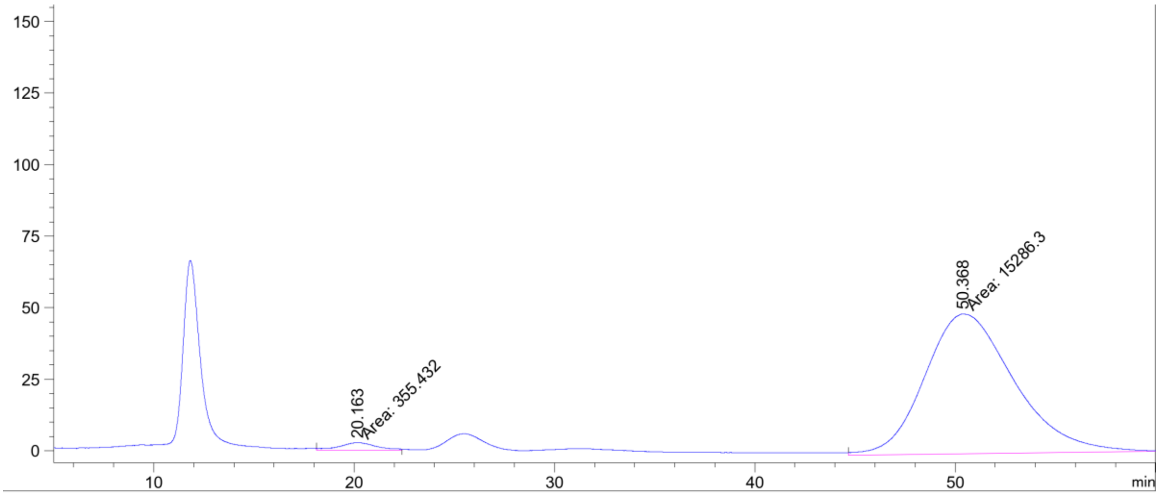

Signal 1: DAD1 A, Sig=220,4 Ref=off

Peak #	RetTime [min]	Type	Width [min]	Area [mAU*s]	Height [mAU]	Area %
1	20.163	MM	2.1655	355.43225	2.73557	2.2723
2	50.368	MM	5.2008	1.52863e4	48.98715	97.7277
Totals :				1.56418e4	51.72272	

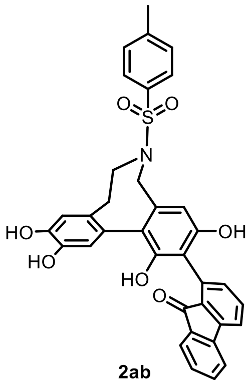

2ab

According to the procedure, **2ab** was obtained using **1ab** (60.7 mg, 0.1 mmol) in 90% yield (54.4mg), yellow foam solid, 86% ee, 4/1 dr, (silica gel flash chromatography: DCM/ acetone = 20:1). R_f = 0.40 (DCM/acetone = 10:1).

^{1}H NMR (500 MHz, CD_3OD): δ 7.68 – 7.59 (m, 5H), 7.56 (d, *J* = 7.0 Hz, 1H), 7.54 – 7.42 (m, 4H), 7.34 (dd, *J* = 16.0, 8.0 Hz, 3H), 7.29 – 7.18 (m, 3H), 6.91 (d, *J* = 7.5 Hz, 1H), 6.85 (s, 1H), 6.67 (s, 1H), 4.62 (d, *J* = 14.0 Hz, 1H), 4.00 (dd, *J* = 12.0, 7.0 Hz, 1H), 2.97 (dd, *J* = 17.0, 10.5 Hz, 2H), 2.75 (dd, *J* = 14.0, 7.0 Hz, 1H), 2.54 (t, *J* = 7.0 Hz, 1H), 2.51 – 2.40 (m, 1H), 2.37 (s, 3H).

^{13}C NMR (125MHz, CD_3OD): δ 194.8, 155.6, 152.8, 146.7, 145.9, 145.5, 144.8, 144.7, 144.6, 138.7, 137.1, 135.8, 135.7, 135.5, 135.5, 135.1, 134.5, 134.5, 134.3, 133.9, 133.3, 130.9, 130.8, 130.6, 129.9, 128.3, 128.2, 127.9, 127.2, 124.4, 121.3, 120.3, 120.3, 118.4, 118.2, 117.5, 114.5, 110.1, 51.1, 50.7, 34.1, 21.5, 21.5.

HRMS (ESI) *m/z* calcd. for $C_{35}H_{27}NO_7S$ $(M+Na)^+$: 628.1400, found: 628.1391

Enantiomeric excess of **2ab** is determined by HPLC (Chiralpak AD-H, Hexane/Isopropanol = 50/50, flow rate = 1.0 mL/min, 220 nm): major isomer: tr = 12.47 min; minor isomer: tr = 25.04

min. $[\alpha]^{20}_{D}$ = -5.44 (c = 1.0, MeOH)

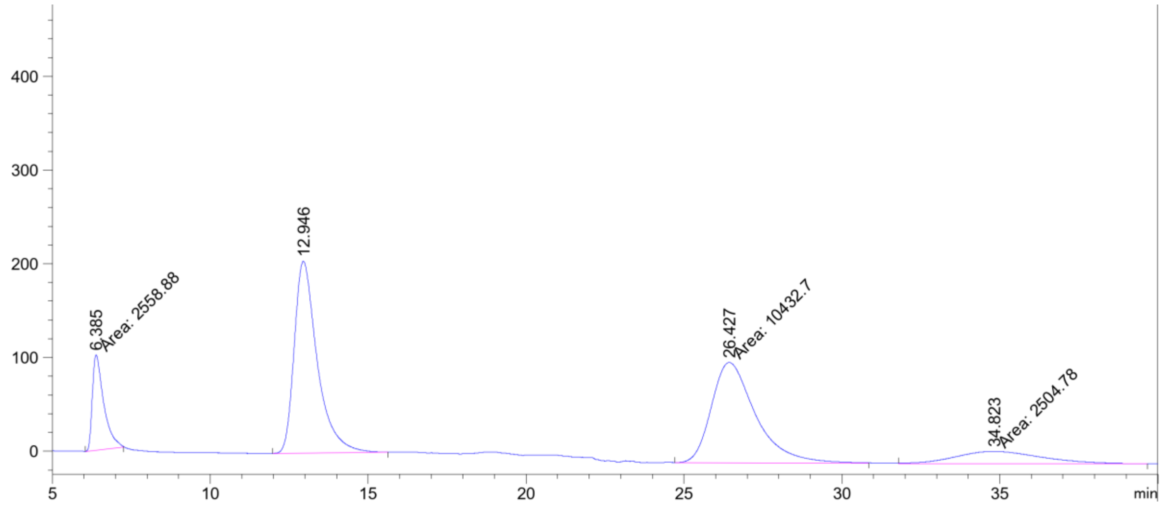

Signal 1: DAD1 A, Sig=220,4 Ref=off

Peak #	RetTime [min]	Type	Width [min]	Area [mAU*s]	Height [mAU]	Area %
1	6.385	MM	0.4192	2558.87866	101.74812	9.8734
2	12.946	BB	0.7598	1.04205e4	204.74948	40.2076
3	26.427	MM	1.6222	1.04327e4	107.18343	40.2543
4	34.823	MM	3.1594	2504.78491	13.21329	9.6647

Totals : 2.59169e4 426.89432

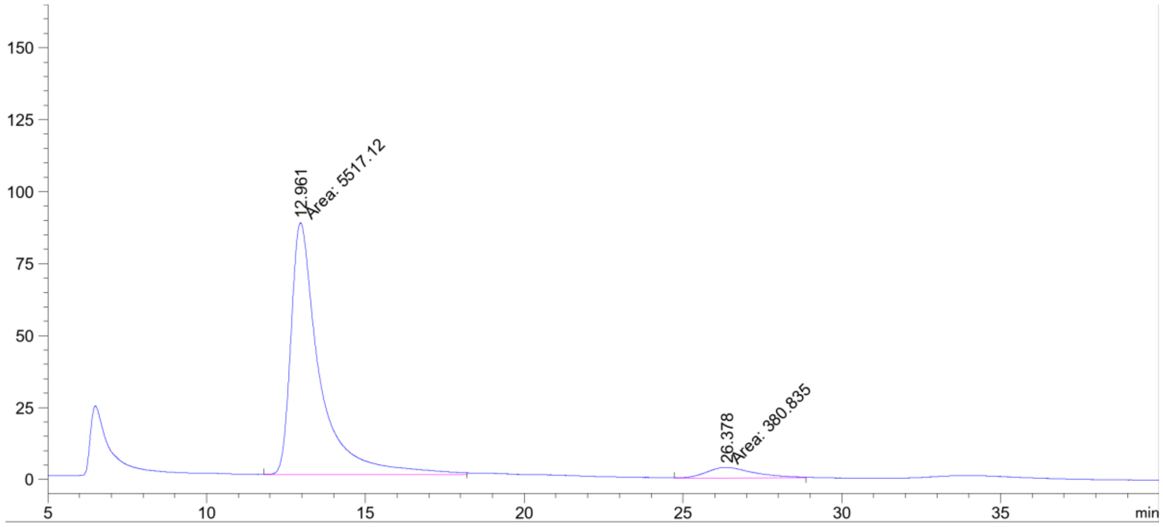

Signal 1: DAD1 A, Sig=220,4 Ref=off

Peak #	RetTime [min]	Type	Width [min]	Area [mAU*s]	Height [mAU]	Area %
1	12.961	MM	1.0509	5517.12061	87.49773	93.5429
2	26.378	MM	1.7259	380.83527	3.67762	6.4571

Totals : 5897.95587 91.17535

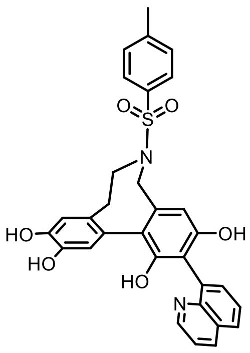

2ac

According to the procedure, **2ac** was obtained using **1ac** (55.6 mg, 0.1 mmol) in 85% yield (47.1mg), light gray foam solid, 78% ee, >20/1 dr, (silica gel flash chromatography: DCM/ acetone = 20:1). R_f = 0.40 (DCM/acetone = 4:1).

^{1}H NMR (500 MHz, CD_3OD): δ 8.74 (d, *J* = 24.0 Hz, 1H), 8.35 (d, *J* = 6.5 Hz, 1H), 7.91 (d, *J* = 8.0 Hz, 1H), 7.83 – 7.58 (m, 4H), 7.47 (s, 1H), 7.34 (s, 2H), 6.98 (s, 1H), 6.83 (d, *J* = 61.0 Hz, 1H), 6.65 (d, *J* = 3.5 Hz, 1H), 4.66 (d, *J* = 13.0 Hz, 1H), 4.02 (dd, *J* = 11.0, 6.5 Hz, 1H), 3.07 – 2.91 (m, 1H), 2.75 (s, 1H), 2.46 (s, 2H), 2.37 (s, 3H).

^{13}C NMR (125 MHz, CD_3OD): δ 156.3, 153.3, 150.7, 147.9, 146.5, 144.8, 144.6, 138.8, 138.7, 137.2, 135.1, 134.5, 134.2, 130.8, 130.3, 128.9, 128.2, 127.7, 122.1, 120.8, 118.5, 117.3, 116.1, 110.4, 51.1, 50.7, 34.1, 33.8, 21.5.

HRMS (ESI) *m/z* calcd. for $C_{31}H_{26}N_2O_6S$ $(M+Na)^+$: 577.1404, found: 577.1389.

Enantiomeric excess of **2ac** is determined by HPLC (Chiralpak AD-H, Hexane/Isopropanol = 40/60, flow rate = 1.0 mL/min, 220 nm): major isomer: tr = 12.86min; minor isomer: tr = 6.40 min. $[\alpha]^{20}_D$ = -194.73 (c = 1.0, MeOH)

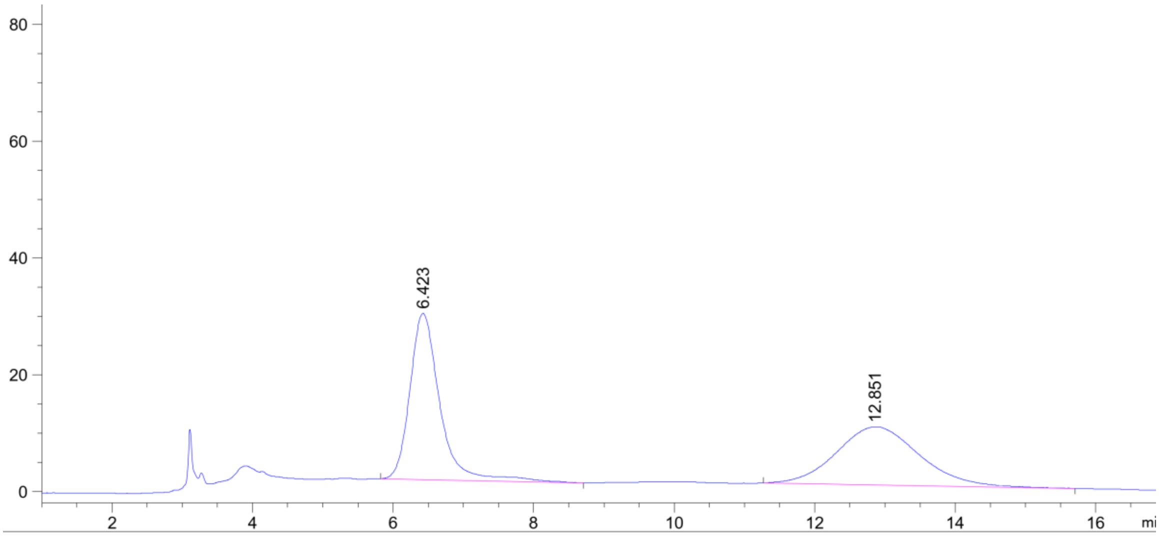

Signal 1: DAD1 A, Sig=220,4 Ref=off

Peak #	RetTime [min]	Type	Width [min]	Area [mAU*s]	Height [mAU]	Area %
1	6.423	BB	0.4433	846.70203	28.44582	50.8144
2	12.851	BB	0.9805	819.56042	9.95450	49.1856

Totals : 1666.26245 38.40032

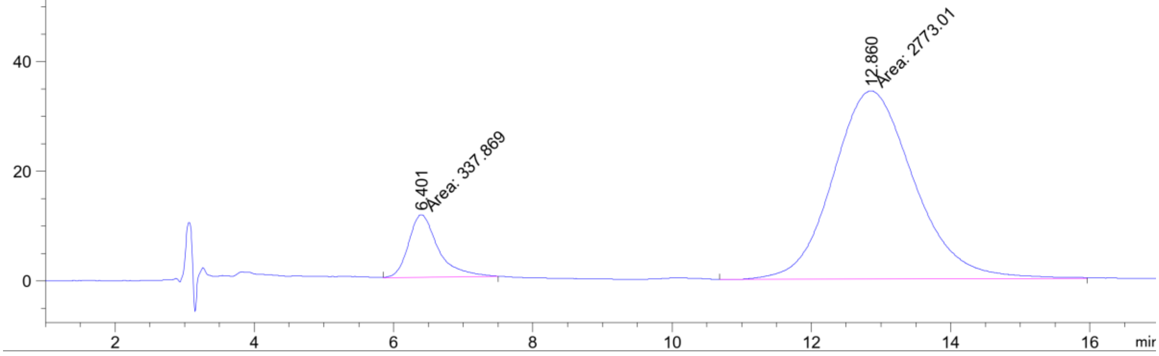

Signal 1: DAD1 A, Sig=220,4 Ref=off

Peak #	RetTime [min]	Type	Width [min]	Area [mAU*s]	Height [mAU]	Area %
1	6.401	MM	0.4934	337.86942	11.41364	10.8609
2	12.860	MM	1.3476	2773.01050	34.29499	89.1391
Totals :				3110.87991	45.70863	

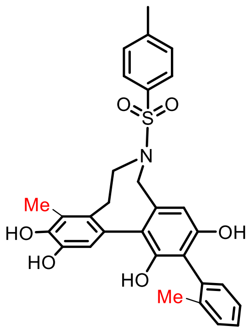

2ad

According to the procedure, **2ad** was obtained using **1ad** (53.3 mg, 0.1 mmol) in 96% yield (51mg), light gray foam solid, 60% ee, >20/1 dr, (silica gel flash chromatography: DCM/ acetone = 20:1). R_f = 0.40 (DCM/acetone = 10:1).

^{1}H NMR (500 MHz, Acetone-d_6): δ 8.40 – 8.27 (m, 1H), 7.99 – 7.92 (s, 1H), 7.71 (d, *J* = 8.0 Hz, 2H), 7.43 – 7.32 (m, 2H), 7.25 – 7.14 (m, 4H), 6.90 (d, *J* = 2.5 Hz, 1H), 6.66 (d, *J* = 9.5 Hz, 1H), 6.62 (d, *J* = 8.0 Hz, 1H), 4.61 (d, *J* = 13.5 Hz, 1H), 4.10 (dd, *J* = 12.0, 8.0 Hz, 1H), 3.05 (dd, *J* = 15.0, 7.5 Hz, 1H), 2.55 – 2.44 (m, 1H), 2.41 – 2.32 (m, 4H), 2.23 (s, 3H), 2.19 – 2.10 (m, 3H).

^{13}C NMR (125 MHz, Acetone-d_6): δ 154.9, 152.0, 144.0, 143.8, 142.9, 138.5, 137.7, 136.8, 134.8, 131.9, 131.9, 130.3, 130.2, 127.9, 127.8, 126.3, 126.1, 123.1, 120.2, 116.4, 114.8, 109.4, 50.0, 48.8, 29.3, 21.1, 19.9, 12.1.

HRMS (ESI) *m/z* calcd. for $C_{30}H_{29}NO_6S$ $(M+Na)^+$: 554.1608, found: 554.1603.

Enantiomeric excess of **2ad** is determined by HPLC (Chiralpak AD-H, Hexane/Isopropanol = 50/50, flow rate = 1.0 mL/min, 254 nm): major isomer: tr = 10.49 min; minor isomer: tr = 30.30min.

$[\alpha]^{20}_D$ = -131.53 (c = 1.0, MeOH)

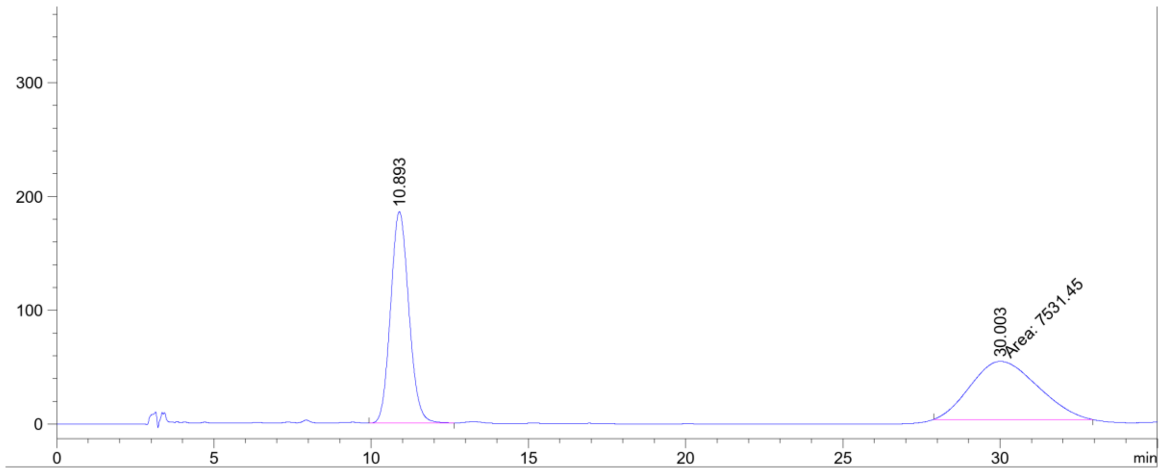

Signal 1: DAD1 B, Sig=254,4 Ref=off

Peak #	RetTime [min]	Type	Width [min]	Area [mAU*s]	Height [mAU]	Area %
1	10.893	BB	0.6277	7509.25049	185.73335	49.9262
2	30.003	MM	2.4619	7531.44775	50.98673	50.0738

Totals : 1.50407e4 236.72009

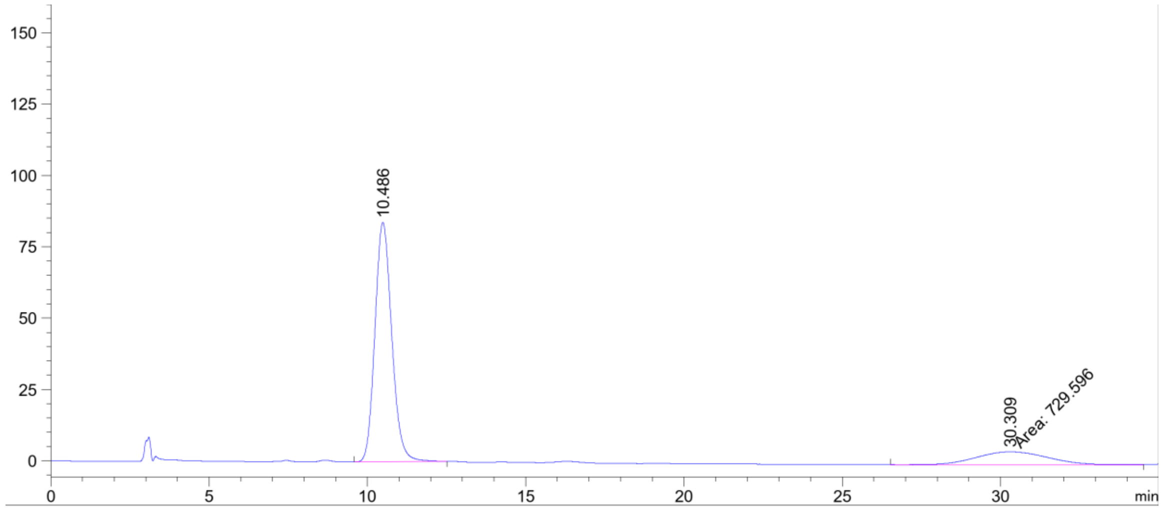

Signal 1: VWD1 A, Wavelength=254 nm

Peak #	RetTime [min]	Type	Width [min]	Area [mAU*s]	Height [mAU]	Area %
1	10.486	BB	0.5713	3101.50928	83.89839	80.9560
2	30.309	MM	2.6638	729.59613	4.56483	19.0440

Totals : 3831.10541 88.46322

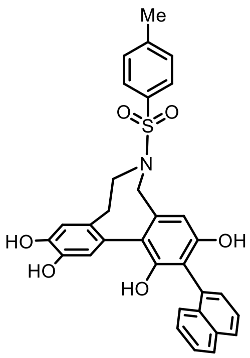

2ae

According to the procedure, **2ae** was obtained using **1ae** (55.5 mg, 0.1 mmol) in 95% yield (52.7mg), light gray foam solid, 88% ee, 8/1 dr, (silica gel flash chromatography: DCM/ acetone = 20:1). R_f = 0.40 (DCM/acetone = 10:1).

^{1}H NMR (500 MHz, Acetone-d_6): δ 8.02 (d, J = 7.5 Hz, 1H), 7.96 – 7.86 (m, 3H), 7.77 (d, J = 8.0 Hz, 1H), 7.74 – 7.70 (m, 2H), 7.58 – 7.53 (m, 1H), 7.52 – 7.43 (m, 3H), 7.40 – 7.36 (m, 2H), 7.01 (s, 1H), 6.83 (s, 1H), 6.77 – 6.68 (m, 2H), 4.70 (d, J = 13.5 Hz, 1H), 4.13 – 4.04 (m, 1H), 3.08 (s, 1H), 3.05 – 3.00 (m, 1H), 2.88 – 2.80 (m, 1H), 2.57 – 2.42 (m, 2H), 2.38 (s, 3H).

^{13}C NMR (125 MHz, Acetone-d_6): δ 155.6, 152.8, 152.7, 145.6, 143.7, 143.6, 138.3, 136.7, 136.6, 134.5, 133.5, 133.4, 133.2, 132.8, 130.2, 130.1, 129.5, 128.6, 128.5, 128.2, 127.6, 126.7, 126.6, 126.2, 126.1, 126.0, 119.4, 117.8, 116.8, 109.4, 50.4, 49.9, 33.3, 21.0, 21.0.

HRMS (ESI) m/z calcd. for $C_{32}H_{27}NO_6S$ $(M+Na)^+$: 576.1451, found: 576.1445.

Enantiomeric excess of **2ae** is determined by HPLC (Chiralpak AD-H, Hexane/Isopropanol = 40/60, flow rate = 1.0 mL/min, 220 nm): major isomer: tr = 12.79 min; minor isomer: tr = 6.48min. $[\alpha]^{20}_{D}$ = -459.87 (c = 1.0, $CHCl_3$).

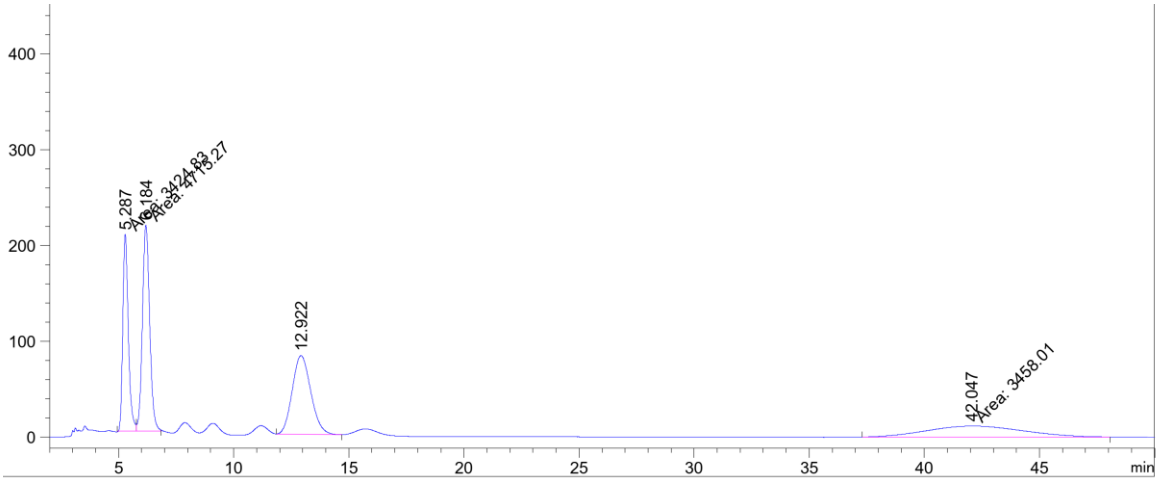

Signal 1: DAD1 A, Sig=220,4 Ref=off

Peak #	RetTime [min]	Type	Width [min]	Area [mAU*s]	Height [mAU]	Area %
1	5.287	MF	0.2782	3424.82935	205.16283	21.1547
2	6.184	FM	0.3658	4715.27148	214.86444	29.1256
3	12.922	BB	0.8647	4591.33643	82.06542	28.3601
4	42.047	MM	4.9620	3458.01294	11.61497	21.3597

Totals : 1.61895e4 513.70766

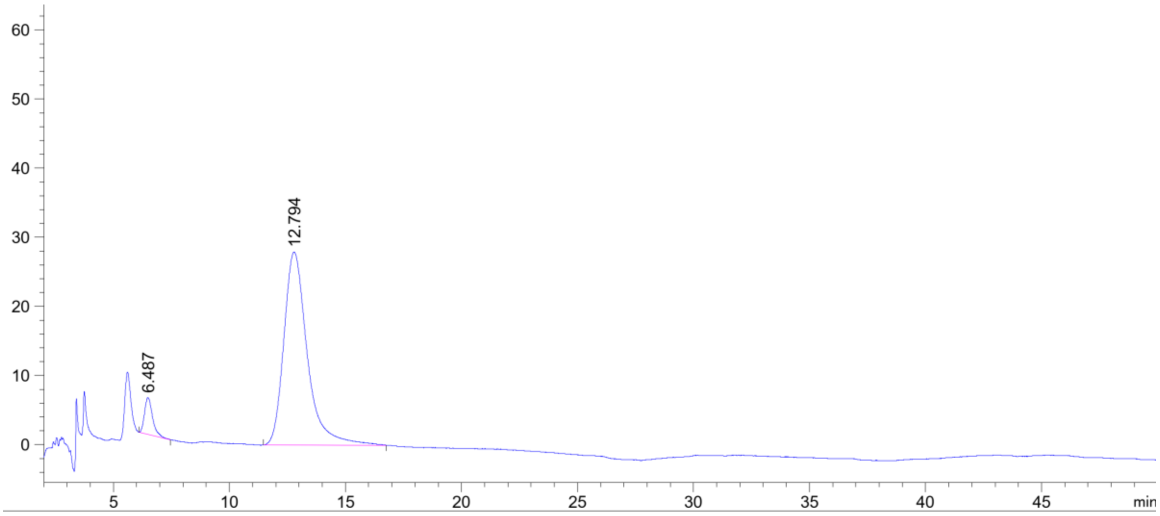

Signal 1: DAD1 A, Sig=220,4 Ref=off

Peak #	RetTime [min]	Type	Width [min]	Area [mAU*s]	Height [mAU]	Area %
1	6.487	BB	0.3499	127.60725	5.31978	6.0918
2	12.794	BB	0.9854	1967.12451	27.94067	93.9082

Totals : 2094.73177 33.26046

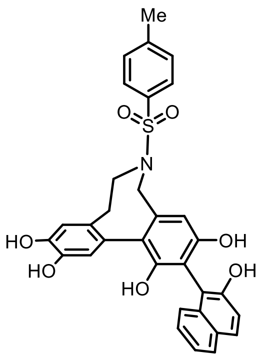

2af

According to the procedure, **2af** was obtained using **1af** (57.1 mg, 0.1 mmol) in 93% yield (53mg) , light gray foam solid, 96% ee, >20/1 dr, (silica gel flash chromatography: DCM/ acetone = 10:1). R_f = 0.40 (DCM/acetone = 4:1).

^{1}H NMR (500 MHz, Acetone-d_6): δ 7.96 (s, 1H), 7.88 (d, *J* = 4.5 Hz, 2H), 7.80 (dd, *J* = 20.0, 11.0 Hz, 3H), 7.72 (d, *J* = 8.0 Hz, 2H), 7.50 (d, *J* = 8.0 Hz, 1H), 7.44 – 7.34 (m, 3H), 7.30 (ddd, *J* = 8.0, 7.0, 1.0 Hz, 1H), 7.23 (d, *J* = 9.0 Hz, 1H), 6.97 (s, 1H), 6.81 (s, 1H), 6.74 (s, 1H), 6.72 (s, 1H), 4.66 (d, *J* = 13.5 Hz, 1H), 4.06 (dd, *J* = 12.0, 7.0 Hz, 1H), 3.02 – 2.94 (m, 4H), 2.81 (dd, *J* = 14.0, 7.0 Hz, 1H), 2.58 (dd, *J* = 14.0, 10.5 Hz, 1H), 2.48 (t, *J* = 11.0 Hz, 1H), 2.40 (s, 3H).

^{13}C NMR (125 MHz, Acetone-d_6): δ 159.4, 157.3, 156.7, 148.6, 146.8, 146.6, 141.8, 139.5, 138.1, 136.2, 133.3, 133.1, 132.7, 131.5, 130.7, 130.1, 129.6, 128.2, 126.2, 122.7, 122.3, 121.1, 119.7, 115.9, 112.5, 112.2, 53.6, 53.1, 36.1, 24.1.

HRMS (ESI) *m/z* calcd. for $C_{32}H_{27}NO_7S$ $(M+Na)^+$: 592.1400, found: 592.1399.

Enantiomeric excess of **2af** is determined by UPC^2 (CHIRALPAK® IG-3, CO_2/MeOH =62/38, flow rate = 1.0 mL/min, 220 nm): major isomer: tr = 34.34 min; minor isomer: tr = 14.97 min. $[\alpha]^{20}_{D}$ = -760.33(c = 1.0, $CHCl_3$).

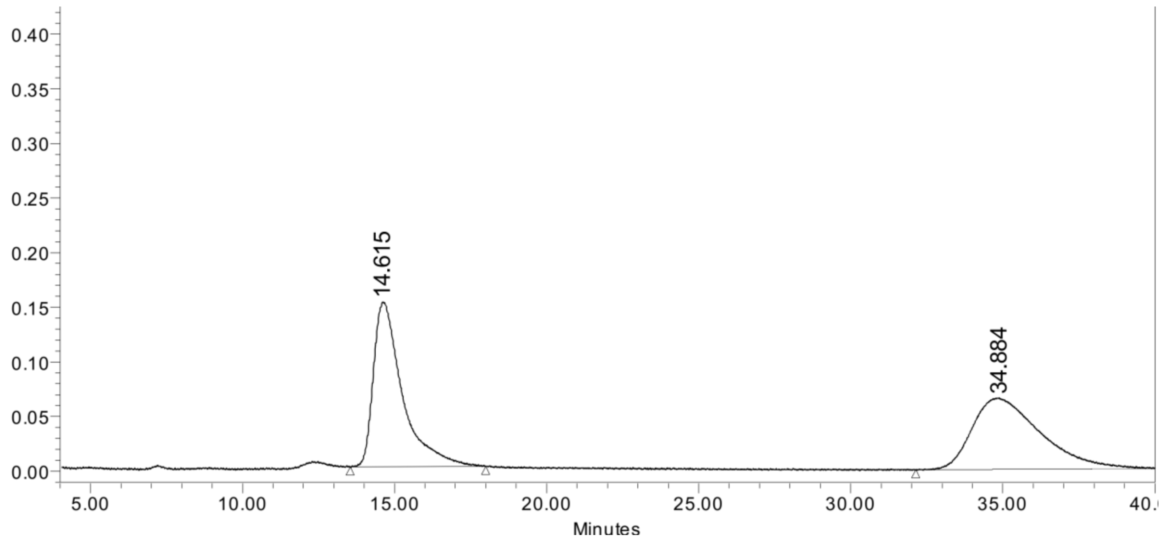

Signal: Sig = 220nm

Peak	RetTime	Area	Height	Area%
1	14.615	10002713	150748	49.67
2	34.884	10137126	65239	50.33

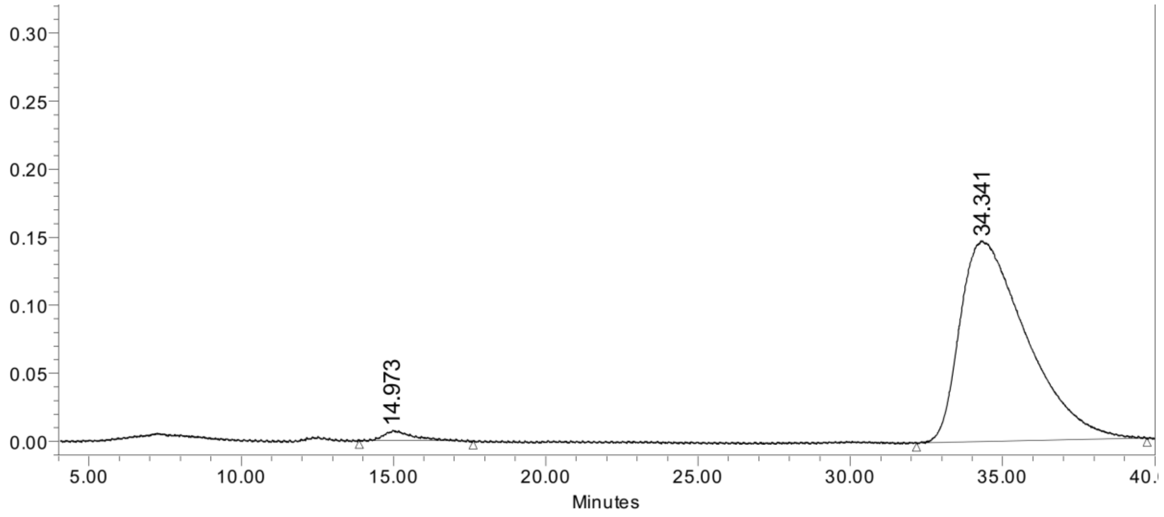

Peak	RetTime	Area	Height	Area%
1	14.973	481991	7419	2.09
2	34.341	22544377	147624	97.91

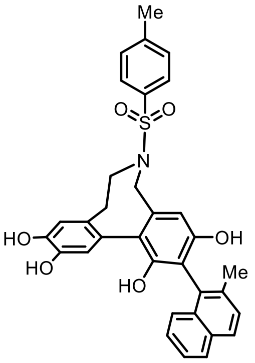

2ag

According to the procedure, **2ag** was obtained using **1ag** (56.9 mg, 0.1 mmol) in 95% yield (54mg), light gray foam solid, 94% ee, 5/1 dr, (silica gel flash chromatography: DCM/ acetone = 20:1). R_f = 0.40 (DCM/acetone = 10:1).

^{1}H NMR (500 MHz, CD_3OD): δ 7.79 (dd, *J* = 12.0, 8.5 Hz, 2H), 7.68 (d, *J* = 8.0 Hz, 2H), 7.50 – 7.39 (m, 2H), 7.33 (dd, *J* = 15.0, 7.0 Hz, 4H), 7.00 (d, *J* = 2.5 Hz, 1H), 6.75 (d, *J* = 5.5 Hz, 1H), 6.67 (s, 1H), 4.81 (s, 3H), 4.72 (d, *J* = 14.0 Hz, 1H), 4.12 – 3.95 (m, 1H), 3.07 (d, *J* = 14.0 Hz, 1H), 2.75 (d, *J* = 7.0 Hz, 1H), 2.56 (d, *J* = 12.0 Hz, 1H), 2.52 – 2.31 (m, 7H).

^{13}C NMR (126 MHz, CD_3OD): δ 155.9, 153.0, 146.5, 144.8, 144.5, 138.8, 137.5, 137.4, 136.8, 134.5, 134.4, 133.8, 131.3, 131.3, 130.8, 129.7, 128.9, 128.9, 128.7, 128.3, 128.2, 127.5, 126.8, 126.6, 125.6, 120.5, 118.6, 117.3, 114.7, 110.3, 110.2, 51.1, 50.6, 34.1, 34.1, 21.5, 21.5, 20.6, 20.5.

HRMS (ESI) *m/z* calcd. for $C_{33}H_{29}NO_6S$ $(M+Na)^+$: 590.1608, found: 590.1597.

Enantiomeric excess of **2ag** is determined by HPLC (Chiralpak AD-H, Hexane/Isopropanol = 60/40, flow rate = 1.0 mL/min, 210 nm): major isomer: tr = 13.65 min; minor isomer: tr = 6.69 min. $[\alpha]^{20}_D$ = -205.60 (c = 1.0, MeOH).

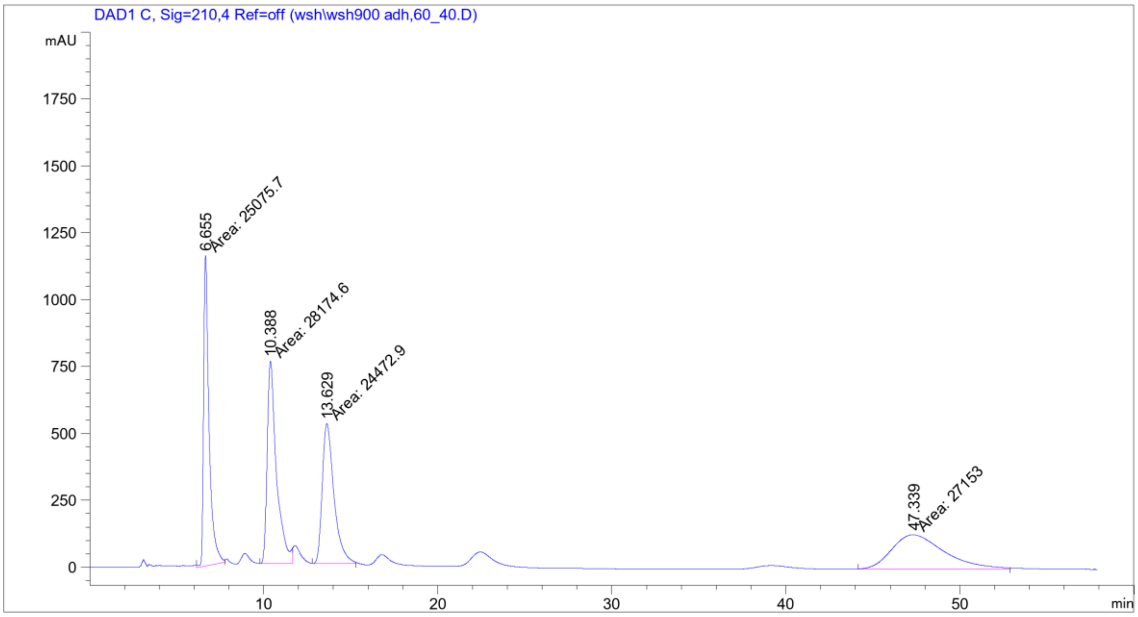

Signal 1: DAD1 C, Sig=210,4 Ref=off

Peak #	RetTime [min]	Type	Width [min]	Area [mAU*s]	Height [mAU]	Area %
1	6.655	MM	0.3603	2.50757e4	1160.03918	23.9098
2	10.388	MM	0.6220	2.81746e4	754.90479	26.8646
3	13.629	MM	0.7809	2.44729e4	522.35156	23.3351
4	47.339	MM	3.5302	2.71530e4	128.19193	25.8905

Totals : 1.04876e5 2565.48746

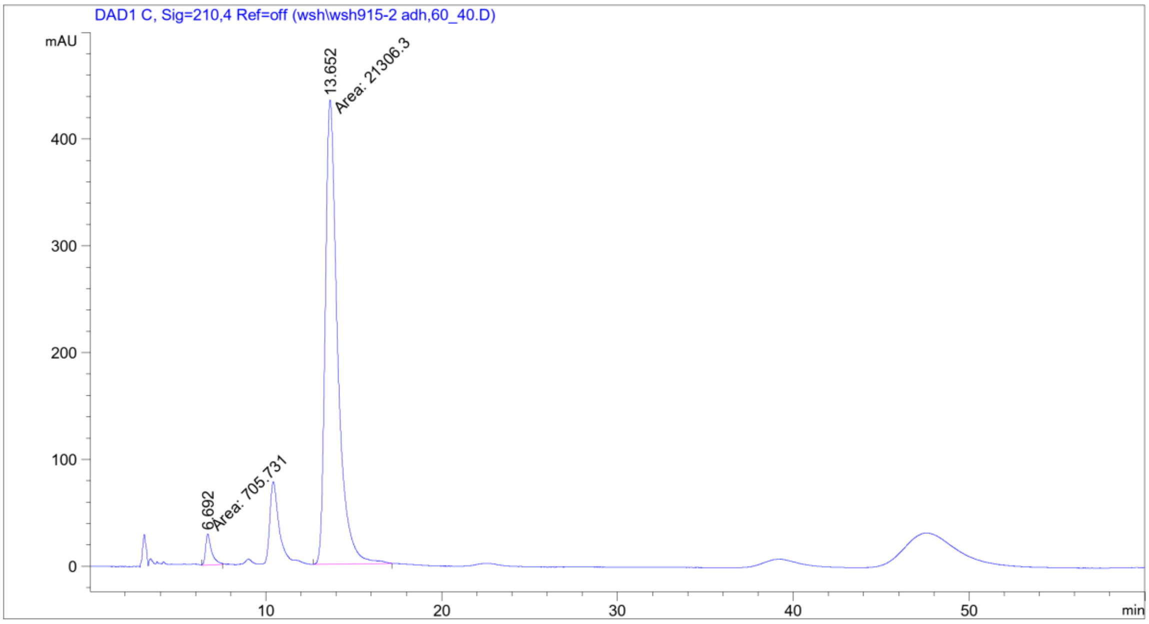

Signal 1: DAD1 C, Sig=210,4 Ref=off

Peak #	RetTime [min]	Type	Width [min]	Area [mAU*s]	Height [mAU]	Area %
1	6.692	MM	0.4002	705.73108	29.38794	3.2061
2	13.652	MM	0.8164	2.13063e4	434.97998	96.7939

Totals : 2.20120e4 464.36792

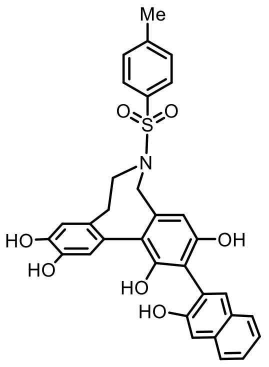

2ah

According to the procedure, **2ah** was obtained using **1ah** (57.1 mg, 0.1 mmol) in 84% yield (47.7mg), light gray foam solid, 86% ee, >20/1 dr, (silica gel flash chromatography: DCM/ acetone = 20:1). R_f = 0.40 (DCM/acetone = 10:1).

^{1}H NMR (500 MHz, CD_3OD): δ 7.72 (dd, *J* = 16.5, 7.5 Hz, 2H), 7.69 – 7.60 (m, 3H), 7.39 – 7.20 (m, 5H), 6.96 (d, *J* = 6.0 Hz, 1H), 6.79 (d, *J* = 17.0 Hz, 1H), 6.65 (s, 1H), 4.64 (d, *J* = 14.0 Hz, 1H), 4.07 – 3.91 (m, 1H), 2.98 (d, *J* = 14.0 Hz, 1H), 2.81 – 2.63 (m, 1H), 2.54 – 2.29 (m, 5H).

^{13}C NMR (125 MHz, CD_3OD): δ 156.2, 154.89, 153.2, 146.5, 144.8, 144.51, 138.8, 138.7, 137.2, 137.1, 136.1, 136.0, 133.8, 133.2, 130.8, 130.1, 128.7, 128.7, 128.2, 127.6, 127.0, 126.8, 125.2, 123.9, 120.9, 120.8, 118.5, 117.3, 117.2, 114.7, 114.6, 110.7, 110.6, 110.4, 110.3, 51.0, 50.6, 34.0, 33.9, 21.4.

HRMS (ESI) *m/z* calcd. for $C_{32}H_{27}NO_7S$ $(M+Na)^+$: 592.1400, found: 592.1396.

Enantiomeric excess of **2ah** is determined by HPLC (Chiralpak IG, Hexane/Isopropanol = 40/60, flow rate = 1.0 mL/min, 210 nm): major isomer: tr = 30.31 min; minor isomer: tr = 15.72 min. $[\alpha]^{20}_D$ = -12.53 (c = 1.0, $CHCl_3$).

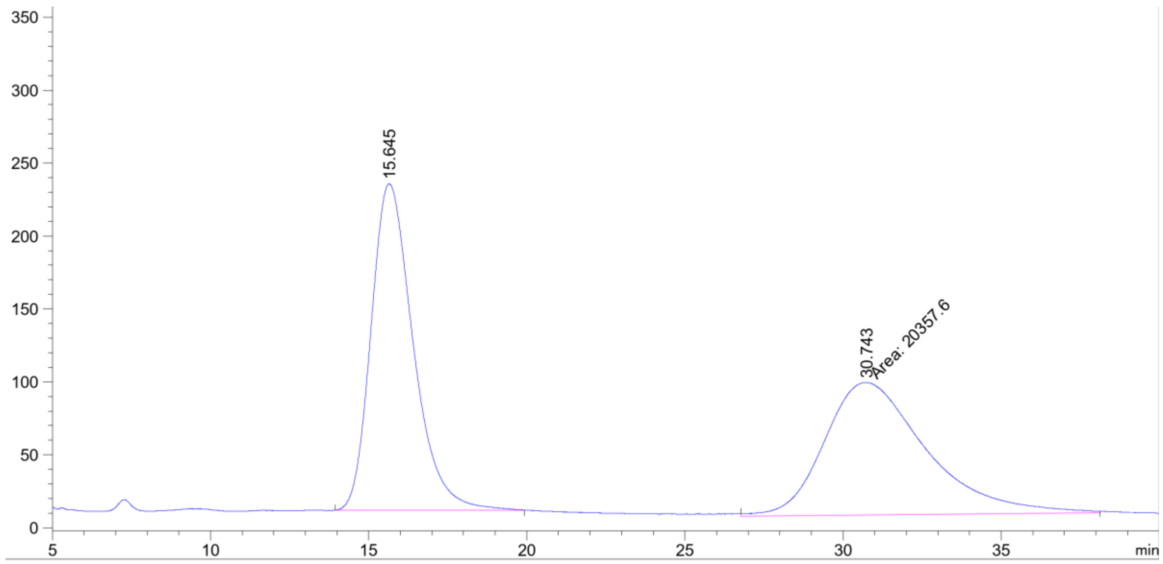

Signal 1: DAD1 C, Sig=210,4 Ref=off

Peak #	RetTime [min]	Type	Width [min]	Area [mAU*s]	Height [mAU]	Area %
1	15.645	BB	1.2108	2.06947e4	223.81134	50.4106
2	30.743	MM	3.7287	2.03576e4	90.99460	49.5894

Totals : 4.10522e4 314.80594

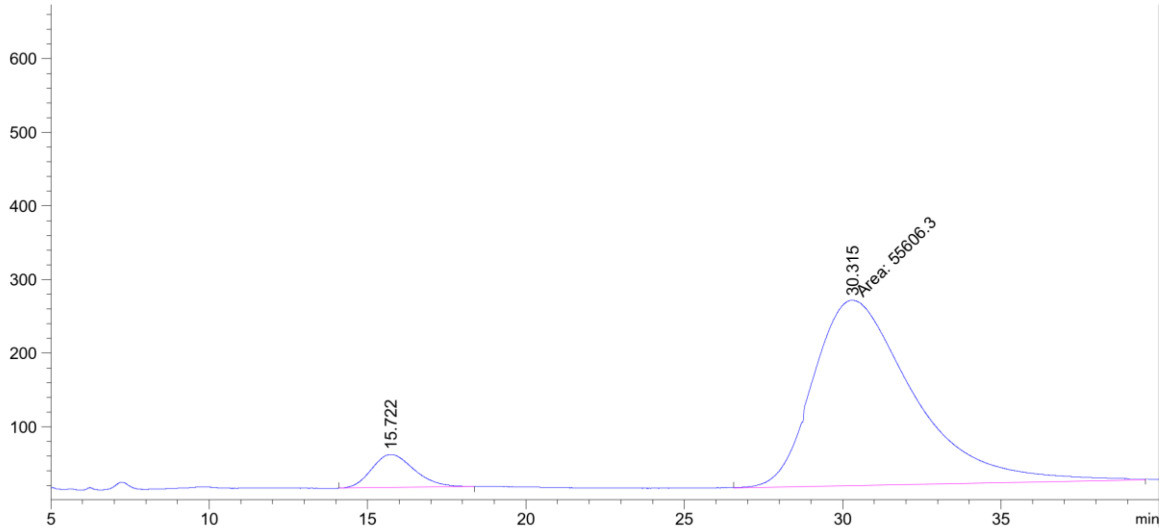

Signal 1: DAD1 C, Sig=210,4 Ref=off

Peak #	RetTime [min]	Type	Width [min]	Area [mAU*s]	Height [mAU]	Area %
1	15.722	BV R	1.0984	4195.33252	45.13731	7.0154
2	30.315	MM	3.6818	5.56063e4	251.71729	92.9846
Totals :				5.98016e4	296.85460	

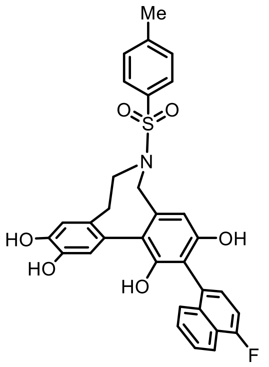

2ai

According to the procedure, **2ai** was obtained using **1ai** (57.3mg, 0.1 mmol) in 95% yield (54.2mg), light gray foam solid, 90% ee, 4/1 dr, (silica gel flash chromatography: DCM/ acetone = 20:1). R_f = 0.40 (DCM/acetone = 10:1).

^{1}H NMR (500 MHz, CD_3OD): δ 8.11 (t, *J* = 8.5 Hz, 1H), 7.75 – 7.62 (m, 3H), 7.37 (m, 6H), 6.99 (s, 1H), 6.76 (d, *J* = 8.0 Hz, 1H), 6.67 (d, *J* = 2.5 Hz, 1H), 4.68 (d, *J* = 14.0 Hz, 1H), 4.11 – 3.95 (m, 1H), 3.01 (dd, *J* = 14.0, 3.0 Hz, 1H), 2.77 (dd, *J* = 13.5, 7.0 Hz, 1H), 2.60 – 2.29 (m, 6H).

^{13}C NMR (125 MHz, CD_3OD): δ 159.7 (d, *J* = 249.9 Hz), 156.4, 153.4, 153.4, 146.5, 144.7 (d, *J* = 36.6 Hz), 139.0, 138.9, 137.0, 135.5, 135.5, 133.8, 130.8, 129.8 (d, *J* = 19.4 Hz), 128.2, 127.6, 127.4, 127.3, 126.9, 125.2 (d, *J* = 16.4 Hz), 121.2, 120.5, 118.5, 118.4, 117.4, 115.4, 115.3, 110.1, 110.0, 51.1, 50.6, 34.1, 21.5.

^{19}F NMR (376 MHz, CD3OD): δ -122.6.

HRMS (ESI) *m/z* calcd. for $C_{32}H_{26}NO_6SF$ $(M+Na)^+$: 594.1357, found: 594.1347.

Enantiomeric excess of **2ai** is determined by HPLC (Chiralpak IG, Hexane/Isopropanol = 65/35, flow rate = 1.0 mL/min, 220 nm): major isomer: tr = 55.38 min; minor isomer: tr = 17.92 min. $[\alpha]^{20}_D$ = -253.20 (c = 1.0, $CHCl_3$).

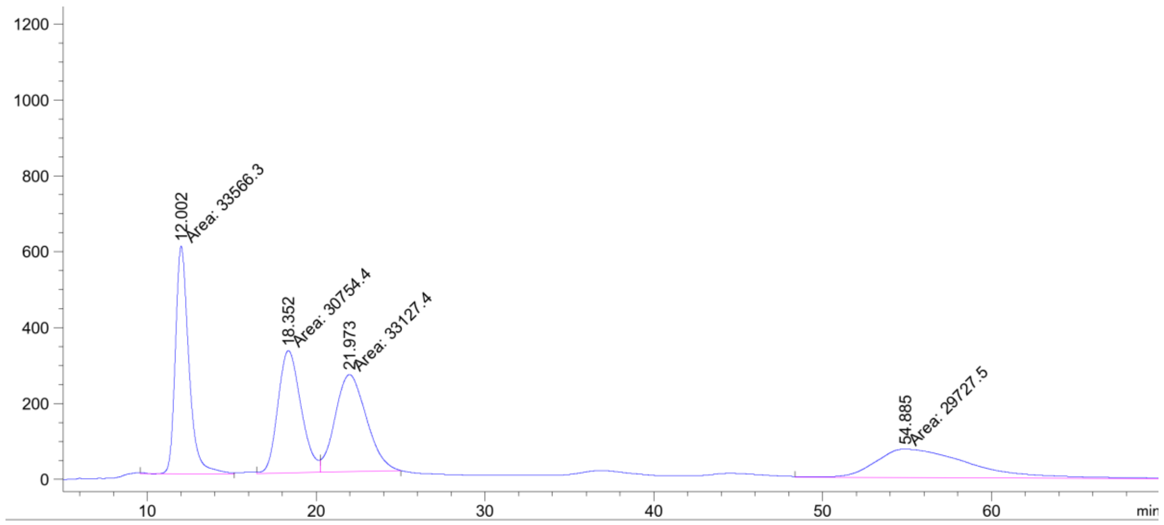

Signal 1: DAD1 A, Sig=220,4 Ref=off

Peak #	RetTime [min]	Type	Width [min]	Area [mAU*s]	Height [mAU]	Area %
1	12.002	MM	0.9318	3.35663e4	600.40051	26.3937
2	18.352	MF	1.5886	3.07544e4	322.64926	24.1826
3	21.973	FM	2.1540	3.31274e4	256.32809	26.0485
4	54.885	MM	6.6332	2.97275e4	74.69422	23.3751

Totals : 1.27176e5 1254.07209

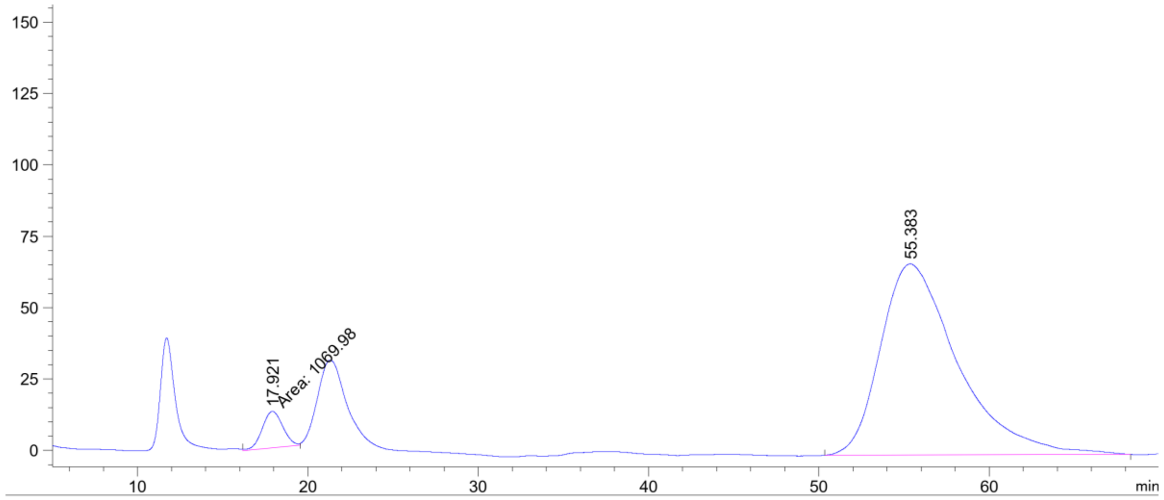

Signal 1: DAD1 A, Sig=220,4 Ref=off

Peak #	RetTime [min]	Type	Width [min]	Area [mAU*s]	Height [mAU]	Area %
1	17.921	MM	1.3976	1069.97693	12.75937	4.8211
2	55.383	BB	3.7000	2.11237e4	66.96835	95.1789
Totals :				2.21936e4	79.72771	

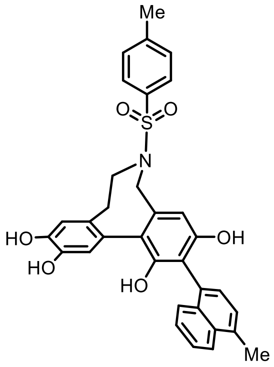

2aj

According to the procedure, **2aj** was obtained using **1aj** (56.9 mg, 0.1 mmol) in 90% yield (51.03mg), light gray foam solid, 90% ee, 5/1 dr, (silica gel flash chromatography: DCM/ acetone = 10:1). R_f = 0.40 (DCM/acetone = 10:1).

^{1}H NMR (500 MHz, Acetone-d_6): δ 8.07 (d, J = 8.4 Hz, 1H), 7.77 (d, J = 8.0 Hz, 1H), 7.72 (dd, J = 8.0, 3.5 Hz, 2H), 7.58 – 7.52 (m, 1H), 7.51 – 7.44 (m, 1H), 7.45 – 7.37 (m, 3H), 7.35 (d, J = 7.0 Hz, 1H), 6.99 (d, J = 2.0 Hz, 1H), 6.81 (s, 1H), 6.73 (s, 1H), 4.69 (d, J = 13.5 Hz, 1H), 4.07 (dd, J = 11.5, 7.5 Hz, 1H), 3.01 (d, J = 13.5 Hz, 1H), 2.90 – 2.78 (m, 1H), 2.72 (s, 3H), 2.55 – 2.41 (m, 2H), 2.39 (s, 3H).

^{13}C NMR (125 MHz, Acetone-d_6): δ 155.8, 152.9, 145.8, 143.8, 138.3, 136.7, 134.4, 133.7, 133.1, 130.9, 130.3, 129.4, 127.7, 127.3, 127.2, 126.8, 126.0, 125.9, 124.8, 119.5, 117.9, 116.8, 115.0, 109.4, 50.5, 50.0, 33.4, 21.1, 19.3.

HRMS (ESI) *m/z* calcd. for $C_{33}H_{29}NO_6S$ $(M+Na)^+$: 590.1608, found: 590.1600.

Enantiomeric excess of **2aj** is determined by UPC^2 (CHIRALPAK® IG-3, CO_2/MeOH = 60/40, flow rate = 0.6 mL/min, 220 nm): major isomer: tr = 29.00 min; minor isomer: tr = 13.03 min. $[\alpha]^{20}$

$_D$ = -366.80(c = 1.0, $CHCl_3$).

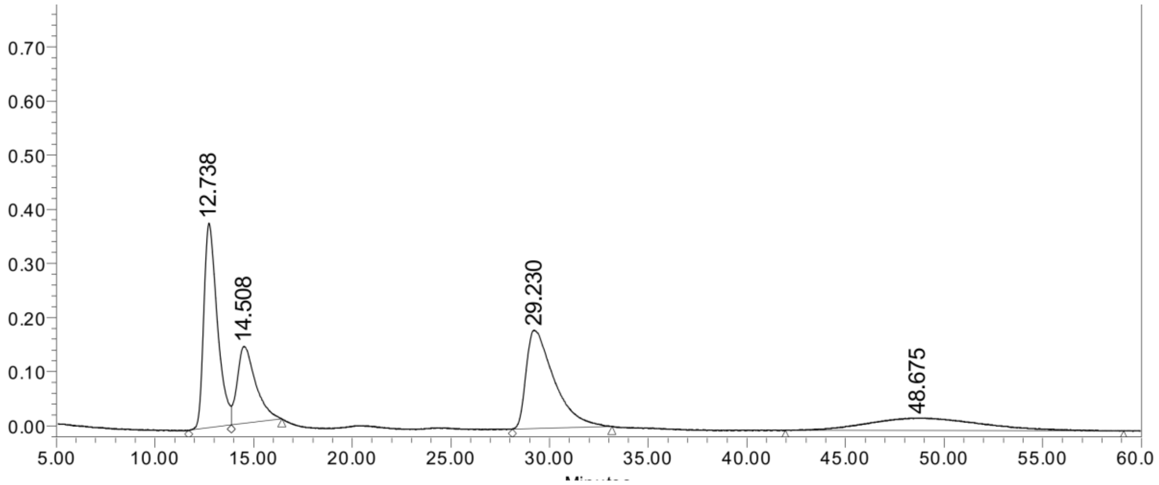

Signal: Sig = 220nm

Peak	RetTime	Area	Height	Area%
1	12.738	17976323	377988	33.14
2	14.508	9301735	142280	17.15
3	29.230	18002525	182062	33.19
4	48.675	8966426	22825	16.53

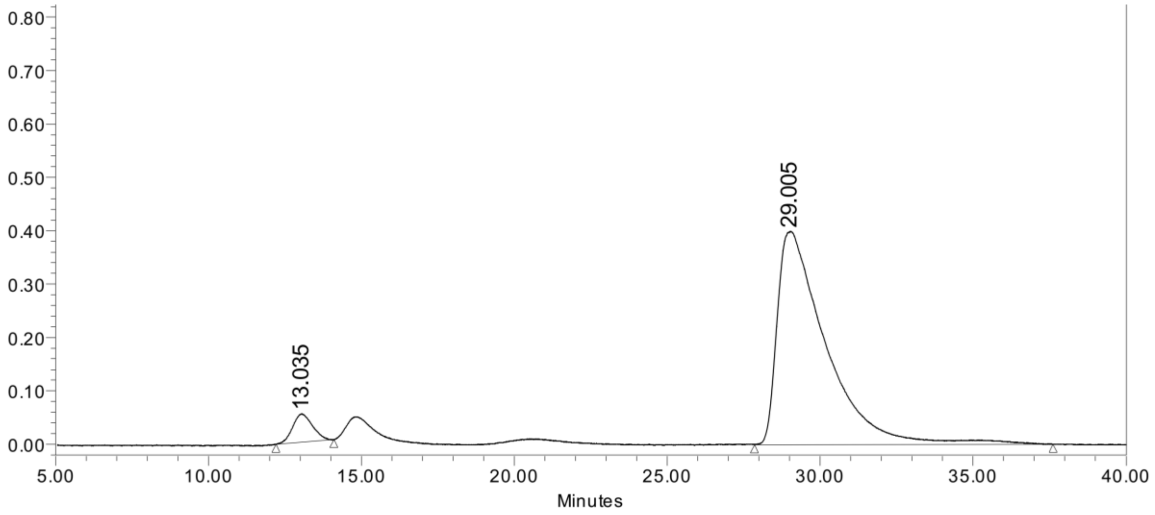

Peak	RetTime	Area	Height	Area%
1	13.035	2391728	52700	5.12
2	29.005	44285085	399215	94.88

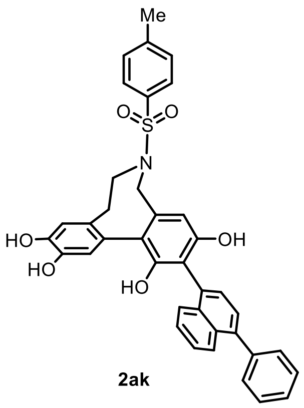

2ak

According to the procedure, **2ak** was obtained using **1ak** (63.1 mg, 0.1 mmol) in 90% yield (mg), light gray foam solid, 82% ee, 4/1 dr, (silica gel flash chromatography: DCM/acetone = 20:1). R_f = 0.40 (DCM/acetone = 10:1).

^{1}H NMR (500 MHz, Acetone-d_6): δ 8.13 (s, 1H), 7.93 (dd, J = 7.5, 2.0 Hz, 2H), 7.87 (dd, J = 7.5, 2.0 Hz, 2H), 7.78 – 7.66 (m, 2H), 7.58 – 7.33 (m, 11H), 7.03 (d, J = 2.0 Hz, 1H), 6.84 (d, J = 4.5 Hz, 2H), 6.75 (s, 1H), 4.71 (d, J = 13.5 Hz, 1H), 4.08 (dd, J = 11.0, 7.0 Hz, 1H), 3.02 (s, 2H), 2.85 (dd, J = 13.5, 7.0 Hz, 1H), 2.49 (dd, J = 17.0, 12.5 Hz, 2H), 2.39 (s, 3H).

^{13}C NMR (125 MHz, Acetone-d_6) δ 155.8, 152.9, 145.7, 143.8, 141.5, 140.2, 138.4, 136.7, 133.9, 132.8, 132.4, 130.5, 130.2, 129.1, 128.9, 127.8, 127.7, 127.2, 126.7, 126.4, 126.3, 126.2, 126.1, 119.5, 117.9, 116.9, 114.9, 109.5, 50.5, 50.0, 33.4, 21.1.

HRMS (ESI) *m/z* calcd. for $C_{38}H_{31}NO_6S$ $(M+Na)^+$: 652.1764, found: 652.1758.

Enantiomeric excess of **2ak** is determined by UPC^2 (CHIRALPAK® IG-3, CO_2/MeOH = 55/45, flow rate = 1.0 mL/min, 222 nm): major isomer: tr = 22.93 min; minor isomer: tr = 5.04 min. $[\alpha]^{20}_D$ = -516.73 (c = 1.0, $CHCl_3$).

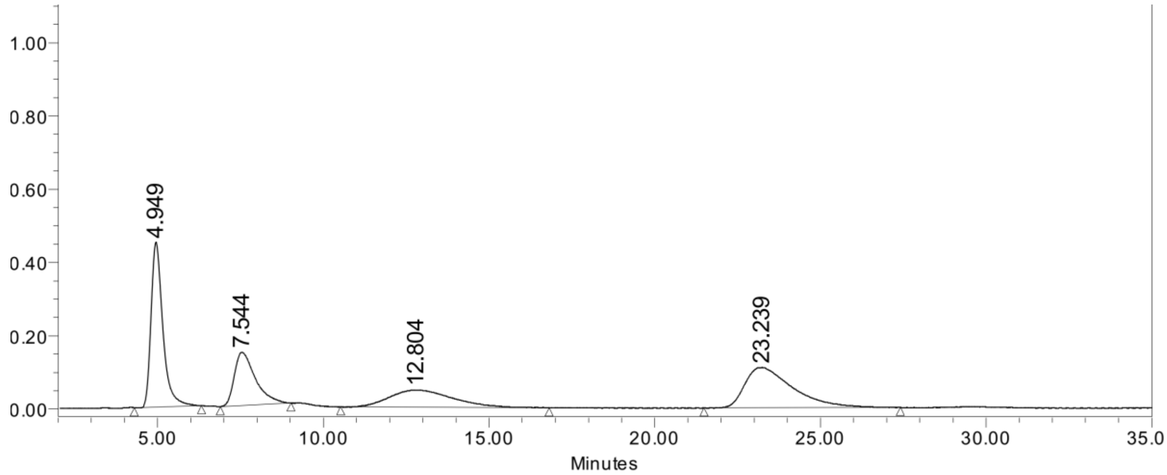

Signal: Sig = 222nm

Peak	RetTime	Area	Height	Area%
1	4.949	11108785	449815	31.91
2	7.544	6436685	145723	18.49
3	12.804	6183417	46909	17.76
4	23.239	11080542	110407	31.83

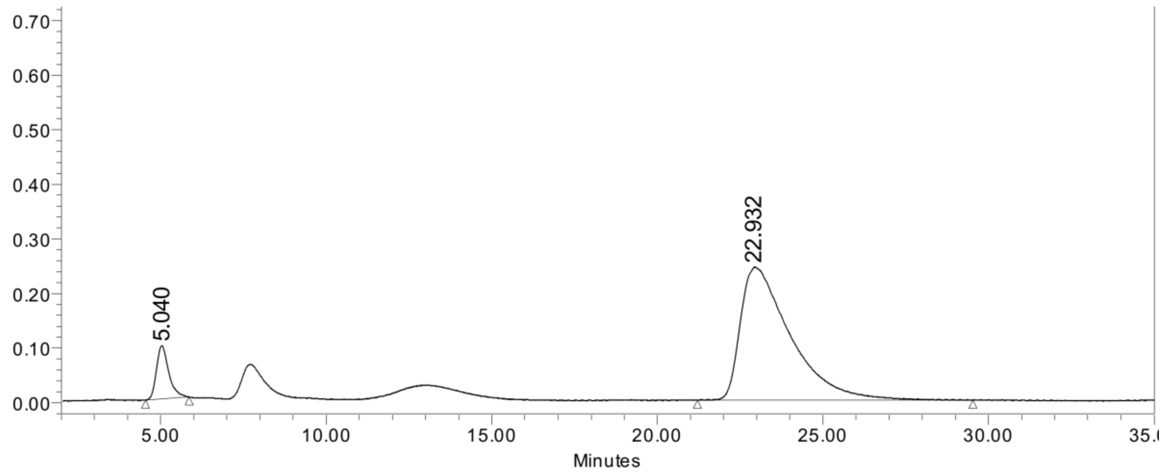

Peak	RetTime	Area	Height	Area%
1	5.040	2512514	97542	9.00
2	22.932	25411003	243702	91.00

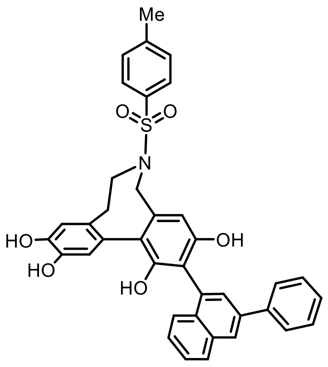

2al

According to the procedure, **2al** was obtained using **1al** (63.1 mg, 0.1 mmol) in 90% yield (56.6mg), light gray foam solid, 98% ee, >20/1 dr, (silica gel flash chromatography: DCM/ acetone = 20:1). R_f = 0.40 (DCM/acetone = 10:1).

^{1}H NMR (500 MHz, CD_3OD): δ 7.89 (dd, *J* = 17.5, 8.5 Hz, 3H), 7.57 (dt, *J* = 15.5, 6.0 Hz, 5H), 7.50 – 7.34 (m, 5H), 7.30 – 7.08 (m, 6H), 6.80 (s, 1H), 6.64 (s, 1H), 6.47 (s, 1H), 4.51 (d, *J* = 14.0 Hz, 1H), 3.99 (dd, *J* = 12.0, 7.0 Hz, 1H), 2.85 (d, *J* = 14.0 Hz, 1H), 2.72 (dd, *J* = 14.0, 7.0 Hz, 1H), 2.51 – 2.36 (m, 2H), 2.30 (s, 3H).

^{13}C NMR (125 MHz, CD_3OD): δ 156.3, 153.3, 146.5, 144.7, 144.5, 143.8, 141.8, 138.6, 137.3,

134.4, 134.4, 133.8, 131.1, 130.7, 130.1, 129.2, 128.9, 128.9, 128.4, 128.2, 127.6, 127.4, 127.3, 127.1, 126.5, 119.9, 118.5, 117.3, 114.9, 109.8, 50.9, 50.6, 34.1, 21.5.

HRMS (ESI) *m/z* calcd. for $C_{38}H_{31}NO_6S$ $(M+Na)^+$: 652.1764, found: 652.1760.

Enantiomeric excess of **2al** is determined by HPLC (Chiralpak AD-H, Hexane/Isopropanol = 70/30, flow rate = 1.0 mL/min, 220nm): major isomer: tr = 48.67 min; minor isomer: tr = 13.46min. $[\alpha]^{20}_{D}$ = -29.00 (c = 1.0, MeOH).

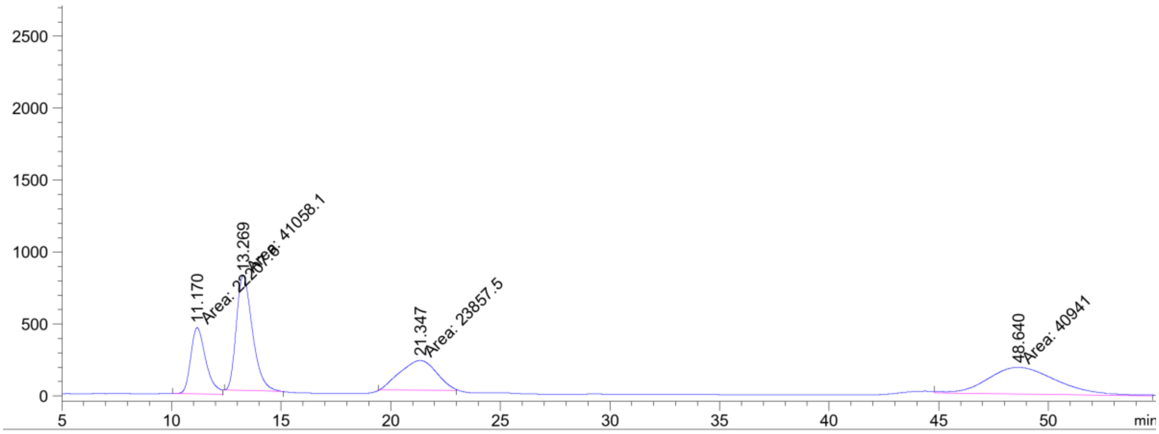

Signal 1: DAD1 A, Sig=220,4 Ref=off

Peak #	RetTime [min]	Type	Width [min]	Area [mAU*s]	Height [mAU]	Area %
1	11.170	MM	0.8025	2.22076e4	461.23526	17.3410
2	13.269	MM	0.8576	4.10581e4	797.88452	32.0605
3	21.347	MM	1.9288	2.38575e4	206.14755	18.6294
4	48.640	MM	3.6825	4.09410e4	185.29738	31.9691

Totals : 1.28064e5 1650.56471

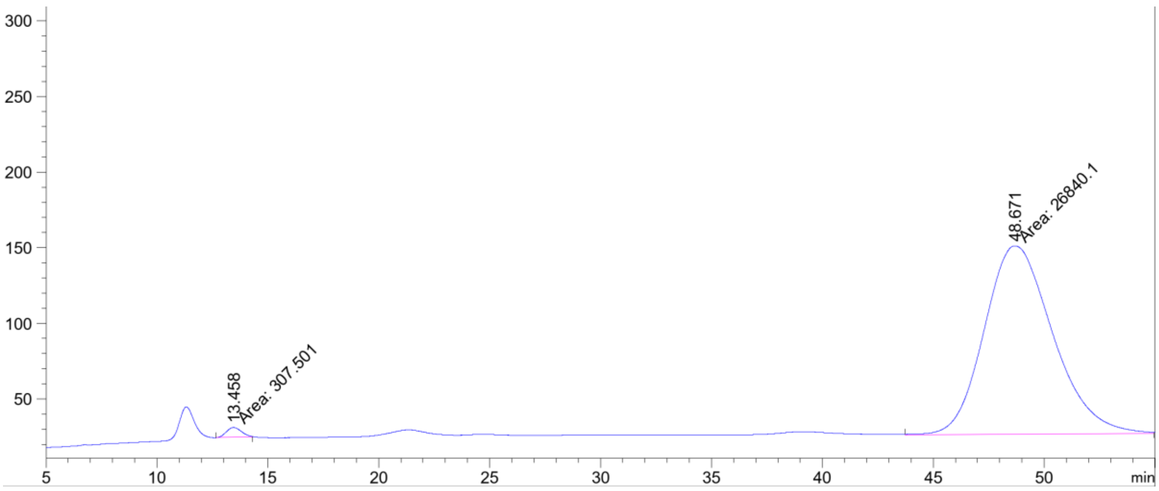

Signal 1: DAD1 A, Sig=220,4 Ref=off

Peak #	RetTime [min]	Type	Width [min]	Area [mAU*s]	Height [mAU]	Area %
1	13.458	MM	0.7898	307.50067	6.48868	1.1327
2	48.671	MM	3.5889	2.68401e4	124.64342	98.8673
Totals :				2.71476e4	131.13210	

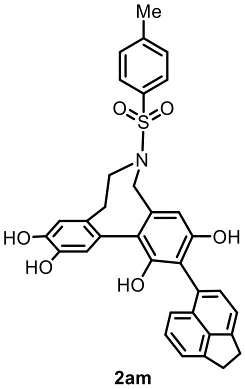

2am

According to the procedure, **2am** was obtained using **1am** (58.1 mg, 0.1 mmol) in 95% yield (55.0mg), light gray foam solid, 85% ee, 3/1 dr, (silica gel flash chromatography: DCM/ acetone = 20:1). R_f = 0.40 (DCM/acetone = 10:1).

^{1}H NMR (500 MHz, CD_3OD): δ 7.70 – 7.58 (m, 2H), 7.46 – 7.15 (m, 7H), 6.97 (s, 1H), 6.77 (d, *J* = 11.0 Hz, 1H), 6.65 (s, 1H), 4.66 (d, *J* = 13.5 Hz, 1H), 4.00 (dd, *J* = 11.5, 7.0 Hz, 1H), 3.38 (s, 4H), 3.00 (d, *J* = 14.0 Hz, 1H), 2.74 (dd, *J* = 14.0, 7.0 Hz, 1H), 2.45 (dd, *J* = 21.0, 12.5 Hz, 2H), 2.35 (s, 3H).

^{13}C NMR (125 MHz, CD_3OD): δ 156.3, 153.2, 153.2, 147.2, 147.1, 147.0, 146.4, 144.8, 144.4,

140.8, 138.6, 138.5, 137.3, 137.1, 133.8, 133.7, 132.5, 132.4, 131.7, 130.8, 128.6 128.2, 127.6, 122.3, 120.4, 120.2, 120.0, 118.6, 117.2, 115.5, 110.1, 51.1, 50.6, 34.0, 31.3, 30.9, 21.5, 21.4.

HRMS (ESI) *m/z* calcd. for $C_{34}H_{29}NO_6S$ $(M+Na)^+$: 602.1608, found: 602.1604.

Enantiomeric excess of **2am** is determined by UPC^2 (CHIRALPAK® IG-3, CO_2/MeOH = 60/40, flow rate = 1.0 mL/min, 220 nm): major isomer: tr = 24.83 min; minor isomer: tr = 13.25 min. $[\alpha]^{20}_{D}$ = -325.00(c = 1.0, acetone)

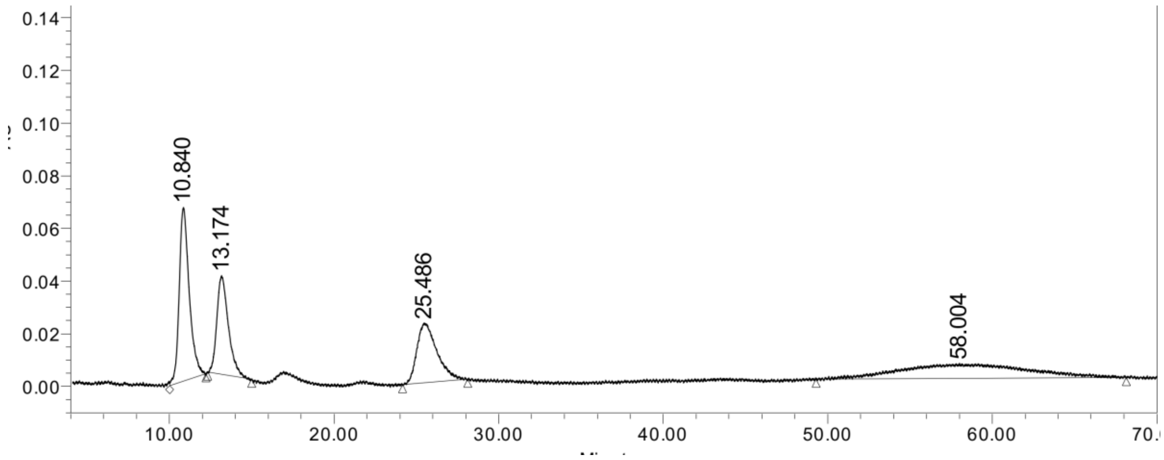

Signal: Sig = 220nm

Peak	RetTime	Area	Height	Area%
1	10.840	2782686	65943	29.79
2	13.174	1812160	37378	19.40
3	25.486	1900306	22659	20.34
4	58.004	2845511	5651	30.46

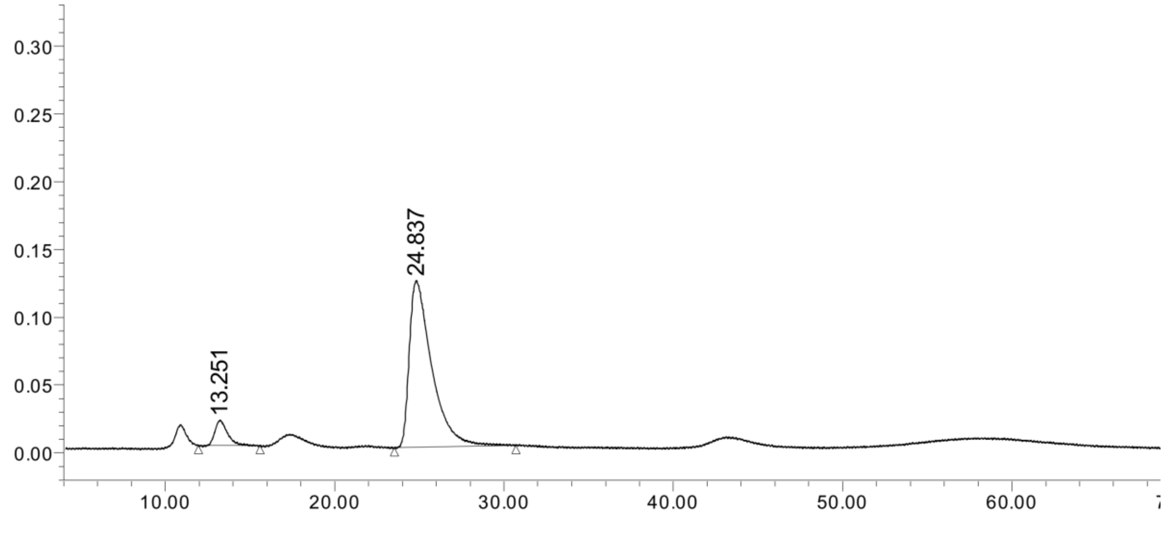

Peak	RetTime	Area	Height	Area%
1	13.251	946083	18409	7.72
2	24.837	11312531	123010	92.28

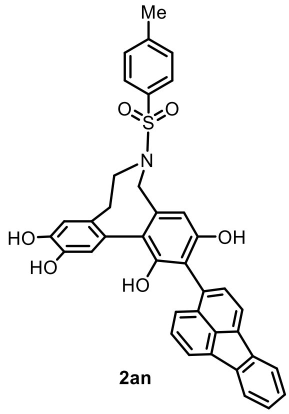

2an

According to the procedure, **2an** was obtained using **1an** (62.9 mg, 0.1 mmol) in 88% yield (55.1mg), light gray foam solid, 86% ee, 2/1 dr, (silica gel flash chromatography: DCM/ acetone = 20:1). R_f = 0.40 (DCM/acetone = 10:1).

^{1}H NMR (500 MHz, CD_3OD): δ 8.00 (d, *J* = 7.0 Hz, 1H), 7.97 – 7.85 (m, 3H), 7.70 – 7.49 (m, 6H), 7.33 (dd, *J* = 5.5, 3.0 Hz, 2H), 7.31 – 7.19 (m, 2H), 7.02 (d, *J* = 3.0 Hz, 1H), 6.81 (d, *J* = 19.0 Hz, 1H), 6.68 (s, 1H), 4.67 (d, *J* = 14.0 Hz, 1H), 4.01 (d, *J* = 11.0 Hz, 1H), 3.00 (d, *J* = 14.0 Hz, 1H), 2.76 (dd, *J* = 11.0, 5.5 Hz, 1H), 2.60 – 2.40 (m, 2H), 2.31 (d, *J* = 14.0 Hz, 3H).

^{13}C NMR (125 MHz, CD_3OD): δ 156.4, 156.3, 153.3, 153.2, 146.6, 144.8, 144.6, 140.9, 140.6, 139.1, 138.9, 138.2, 138.2, 137.6, 137.3, 136.9, 134.0, 133.9, 133.9, 131.9, 131.3, 131.2, 130.8, 128.7, 128.6, 128.5, 128.2, 127.3, 127.1, 122.4, 121.1, 120.8, 120.6, 118.6, 117.4, 115.3, 115.2, 110.2, 51.1, 50.7, 34.1, 21.5, 21.4.

HRMS (ESI) *m/z* calcd. for $C_{38}H_{29}NO_6S$ $(M+Na)^+$: 650.1608, found: 650.1596.

Enantiomeric excess of **2an** is determined by HPLC (Chiralpak IE, Hexane/Isopropanol = 40/60, flow rate = 1.0 mL/min, 254 nm): major isomer: tr = 28.52 min; minor isomer: tr = 17.43 min. $[\alpha]^{20}_D$ = -239.13 (c = 1.0, $CHCl_3$).

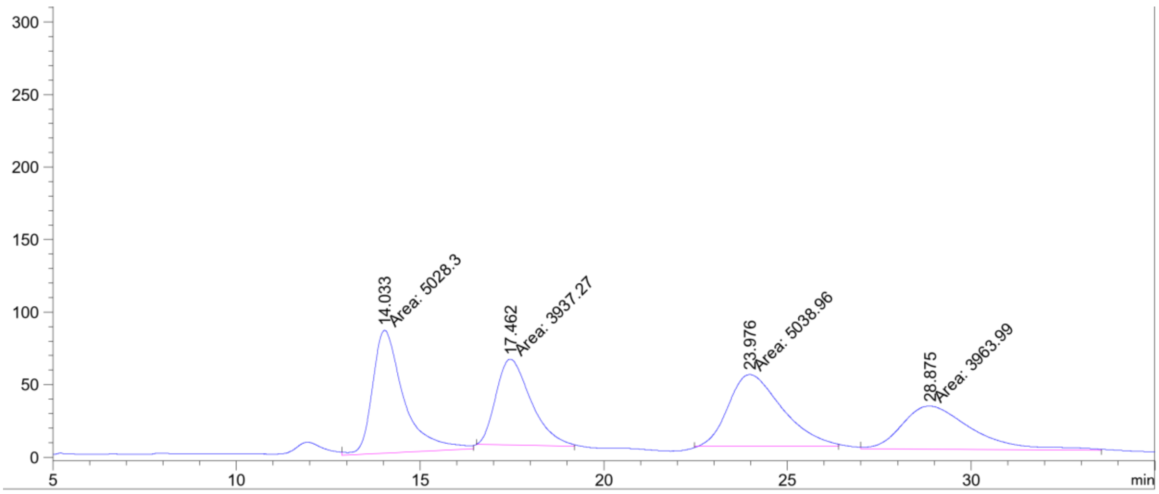

Signal 1: DAD1 H, Sig=290,4 Ref=off

Peak #	RetTime [min]	Type	Width [min]	Area [mAU*s]	Height [mAU]	Area %
1	14.033	MM	0.9903	5028.29541	84.62459	27.9839
2	17.462	MM	1.1081	3937.26685	59.22213	21.9120
3	23.976	MM	1.6973	5038.95801	49.48150	28.0433
4	28.875	MM	2.2150	3963.98633	29.82624	22.0607

Totals : 1.79685e4 223.15445

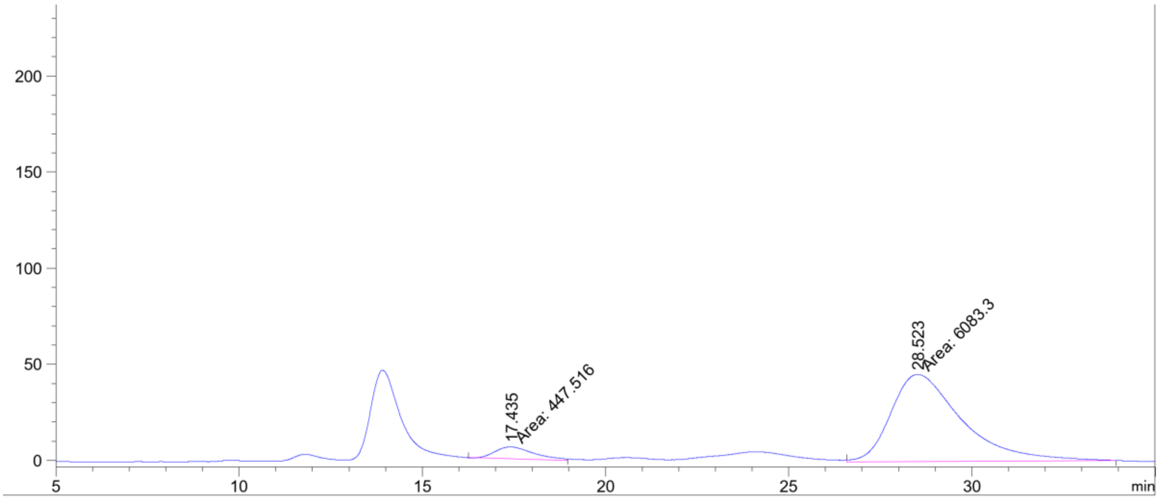

Signal 1: DAD1 B, Sig=254,4 Ref=off

Peak #	RetTime [min]	Type	Width [min]	Area [mAU*s]	Height [mAU]	Area %
1	17.435	MM	1.2217	447.51633	6.10496	6.8524
2	28.523	MM	2.2366	6083.29541	45.33130	93.1476
Totals :				6530.81174	51.43626	

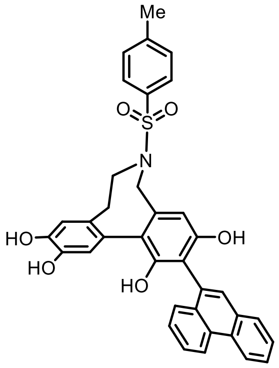

2ao

According to the procedure, **2ao** was obtained using **1ao** (60.5mg, 0.1 mmol) in 91% yield (54.8mg), light gray foam solid, 92% ee, 5/1 dr, (silica gel flash chromatography: DCM/ acetone = 10:1). R_f = 0.40 (DCM/acetone = 10:1).

^{1}H NMR (500 MHz, Acetone-d_6): δ 8.87 (dd, *J* = 15.0, 8.0 Hz, 2H), 7.98 (d, *J* = 7.0 Hz, 2H), 7.91 – 7.84 (m, 1H), 7.82 (s, 1H), 7.79 – 7.59 (m, 6H), 7.41 (d, *J* = 8.0 Hz, 2H), 7.08 (d, *J* = 3.5 Hz, 1H), 6.89 (s, 1H), 6.77 (d, *J* = 2.5 Hz, 1H), 4.76 (d, *J* = 13.5 Hz, 1H), 4.17 – 4.06 (m, 1H), 3.08 (d, *J* = 13.5 Hz, 1H), 2.95 – 2.84 (m, 1H), 2.63 – 2.46 (m, 2H), 2.41 (s, 3H).

^{13}C NMR (125 MHz, Acetone-d_6): δ 155.7, 152.9, 145.5, 143.6, 143.4, 138.4, 136.5, 132.9, 132.6, 132.5, 131.2, 131.2, 130.8, 130.4, 130.1, 129.1, 127.5, 127.1, 127.0, 126.9, 126.8, 123.2, 122.9, 119.5, 117.9, 116.6, 114.6, 109.3, 50.3, 49.9, 33.3, 20.9.

HRMS (ESI) *m/z* calcd. for $C_{36}H_{29}NO_6S$ $(M+Na)^+$: 626.1608, found: 626.1604

Enantiomeric excess of **2ap** is determined by UPC^2 (CHIRALPAK® IG-3, CO_2/MeOH = 45/55, flow rate = 1.0 mL/min, 220 nm): major isomer: tr = 26.65 min; minor isomer: tr = 7.14 min. $[\alpha]^{20}_D$ = -631.13 (c = 1.0, $CHCl_3$).

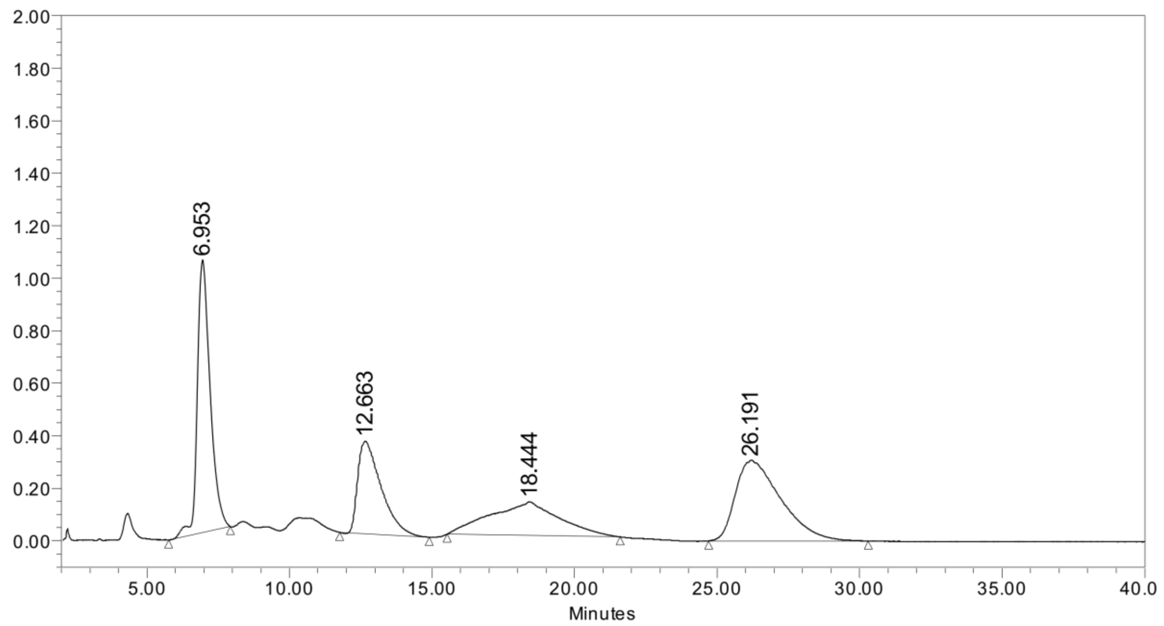

Signal: Sig = 220nm

Peak	RetTime	Area	Height	Area%
1	6.953	32466334	1037500	30.12
2	12.663	20157602	352917	18.70
3	18.444	21470207	127718	19.92
4	26.191	33702901	306815	31.27

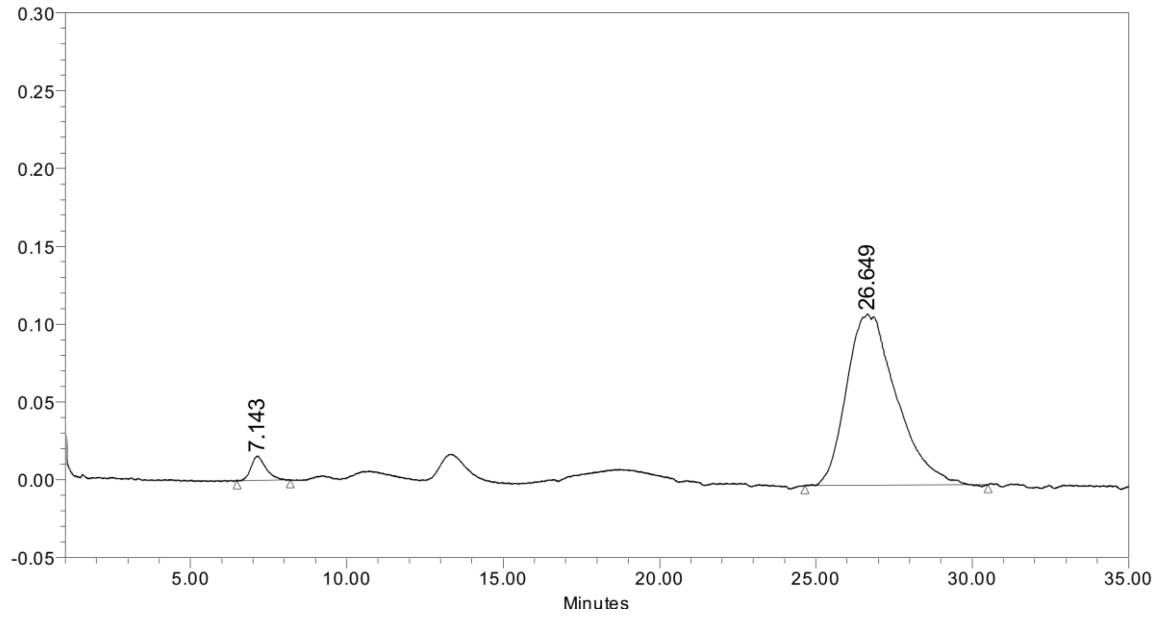

Peak	RetTime	Area	Height	Area%
1	7.143	510465	15485	4.00
2	26.649	12259050	110043	96.00

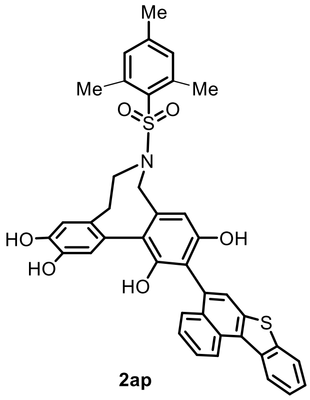

2ap

According to the procedure, **2ap** was obtained using **1ap** (68.9 mg, 0.1 mmol) in 92% yield (63mg), light gray foam solid, 92% ee, 9/1 dr, (silica gel flash chromatography: DCM/ acetone = 20:1). R_f = 0.40 (DCM/acetone = 10:1).

^{1}H NMR (500 MHz, DMSO-*d*$_6$) δ 9.30 (s, 1H), 9.15 (d, *J* = 8.5 Hz, 1H), 9.02 (d, *J* = 8.5 Hz, 1H), 8.89 (s, 1H), 8.83 (s, 1H), 8.20 (d, *J* = 7.5 Hz, 1H), 8.00 (d, *J* = 3.5 Hz, 1H), 7.80 (t, *J* = 8.0 Hz, 2H), 7.73 – 7.65 (m, 1H), 7.60 (dt, *J* = 15.5, 6.0 Hz, 3H), 7.10 (s, 2H), 6.85 (d, *J* = 4.5 Hz, 1H), 6.71 (s, 1H), 6.66 (s, 1H), 4.74 (d, *J* = 14.0 Hz, 1H), 3.48 (dd, *J* = 12.5, 7.0 Hz, 1H), 3.21 (d, *J* = 14.0 Hz, 1H), 2.84 – 2.72 (m, 1H), 2.55 (m, 7H), 2.39 – 2.24 (m, 4H).

^{13}C NMR (125 MHz, Acetone-*d*$_6$) δ 155.6, 153.0, 145.9, 143.7, 143.1, 140.6, 140.2, 139.1, 139.0, 137.1, 134.1, 133.7, 133.1, 132.5, 132.4, 131.6, 129.3, 128.2, 127.7, 126.7, 126.0, 125.7, 125.6, 125.3, 124.7, 123.9, 119.9, 118.1, 116.8, 114.6, 109.8, 48.7, 33.6, 30.3, 22.8, 20.6.

HRMS (ESI) *m/z* calcd. for $C_{40}H_{33}NO_6S_2$ $(M+Na)^+$: 710.1642, found: 710.1627.

Enantiomeric excess of **2ap** is determined by HPLC (Chiralpak IA, Hexane/Isopropanol = 60/40, flow rate = 1.0 mL/min, 220 nm): major isomer: tr = 43.96 min; minor isomer: tr = 16.06 min. $[\alpha]^{20}_{D}$ = -126.73 (c = 1.0, MeOH)

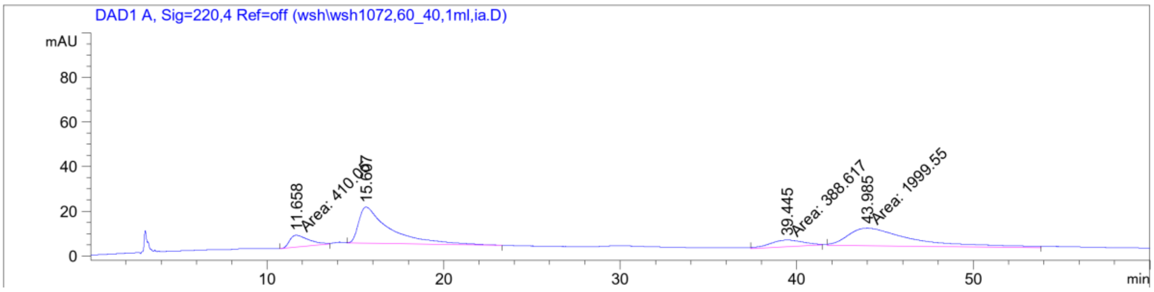

Signal 1: DAD1 A, Sig=220,4 Ref=off

Peak #	RetTime [min]	Type	Width [min]	Area [mAU*s]	Height [mAU]	Area %
1	11.658	MM	1.2643	410.04968	5.40543	8.5983
2	15.607	BB	1.4674	1970.74670	16.08465	41.3244
3	39.445	MM	1.5773	388.61716	2.94918	8.1489
4	43.985	MM	4.2328	1999.55359	7.87321	41.9284

Totals : 4768.96713 32.31248

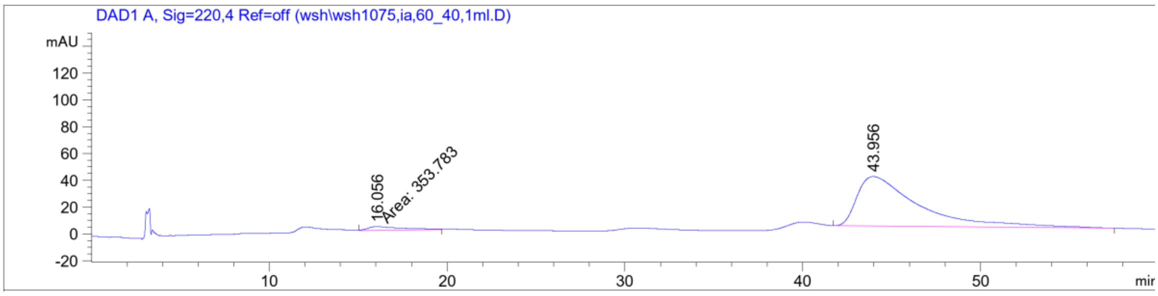

Signal 1: DAD1 A, Sig=220,4 Ref=off

Peak #	RetTime [min]	Type	Width [min]	Area [mAU*s]	Height [mAU]	Area %
1	16.056	MM	2.2169	353.78323	2.65979	3.8087
2	43.956	BB	2.8354	8934.95410	36.94647	96.1913

Totals : 9288.73734 39.60626

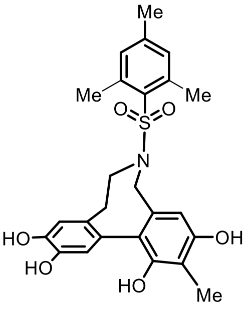

2aq

According to the procedure, **2aq** was obtained using **1aq** (47.1 mg, 0.1 mmol) in 82% yield (38.4mg), light gray foam solid, 94% ee, (silica gel flash chromatography: DCM/ acetone = 20:1).

R_f = 0.40 (DCM/acetone = 10:1).

^{1}H NMR (500 MHz, Acetone-d_6): δ 7.03 (s, 2H), 6.77 (s, 1H), 6.73 (s, 1H), 6.58 (s, 1H), 4.65 (d, *J* = 14.0 Hz, 1H), 3.54 (dd, *J* = 13.0, 7.0 Hz, 1H), 3.18 (d, *J* = 14.0 Hz, 1H), 2.71 (dd, *J* = 14.0, 7.0 Hz, 1H), 2.61 (dd, *J* = 13.0, 11.0 Hz, 1H), 2.54 (s, 6H), 2.35 (dd, *J* = 14.0, 10.5 Hz, 1H), 2.29 (s, 3H), 2.13 (s, 3H).

^{13}C NMR (125 MHz, Acetone-d_6): δ 155.5, 152.6, 145.7, 143.7, 142.7, 140.3, 135.5, 133.9, 133.8, 132.2, 126.2, 118.9, 117.1, 116.9, 110.9, 108.9, 48.5, 48.3, 33.2, 22.5, 20.3, 8.6.

HRMS (ESI) *m/z* calcd. for $C_{25}H_{27}NO_6S$ $(M+Na)^+$: 492.1451, found: 492.1446.

Enantiomeric excess of **2aq** is determined by UPC^2 (CHIRALPAK® OD-3, CO_2/MeOH = 70/30, flow rate = 1.0 mL/min, 220 nm): major isomer: tr = 3.60 min; minor isomer: tr = 4.67 min. $[\alpha]^{20}_D$ = -657.73 (c = 1.0, MeOH).

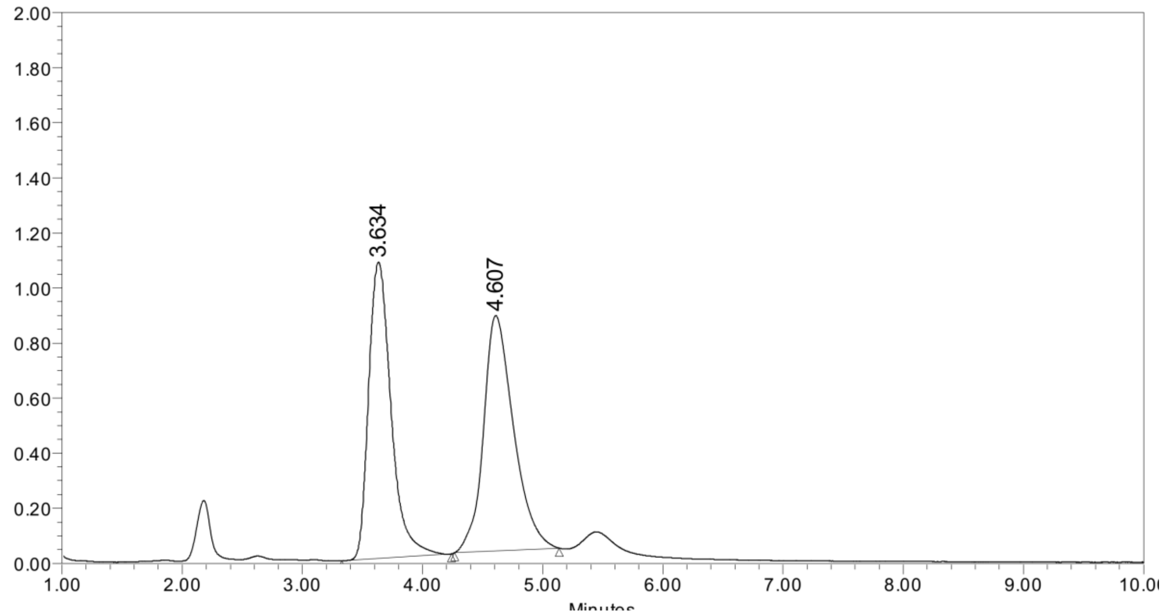

Signal: Sig = 220nm

Peak	RetTime	Area	Height	Area%
1	3.634	14971164	1076274	50.15
2	4.607	14883647	854895	49.85

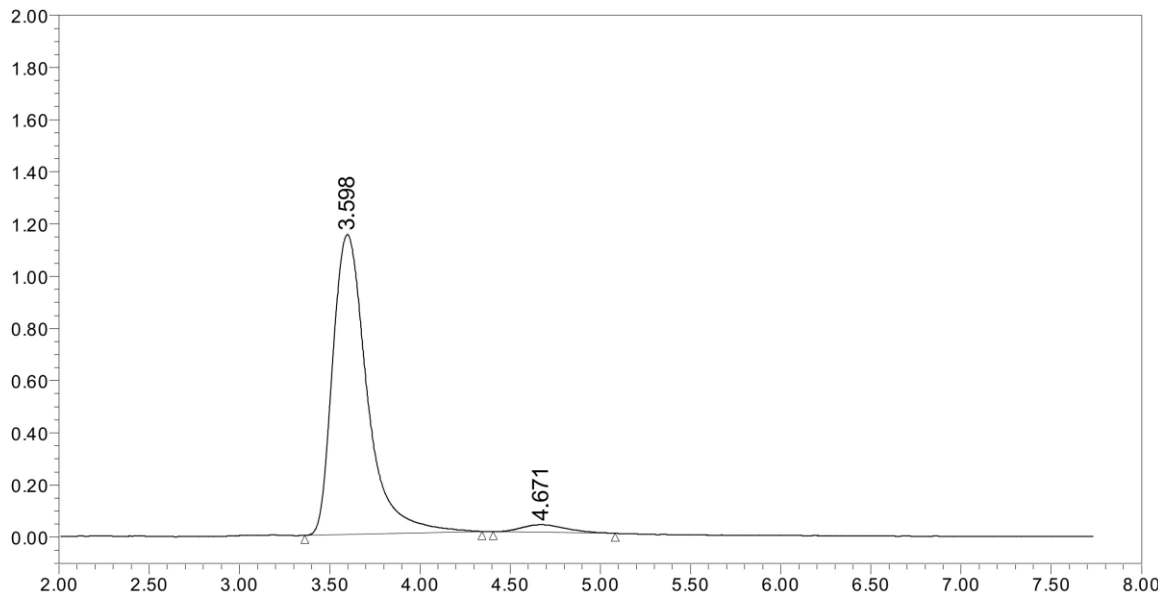

Peak	RetTime	Area	Height	Area%
1	3.598	15234892	1150275	96.78
2	4.671	507612	29363	3.22

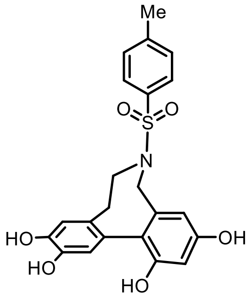

2ar

According to the procedure, **2ar** was obtained using **1ar** (42.9 mg, 0.1 mmol) in 88% yield (37.6mg), 95% ee, light gray foam solid, (silica gel flash chromatography: DCM/ acetone = 20:1). R_f = 0.40 (DCM/acetone = 10:1).

^{1}H NMR (500 MHz, Acetone-d_6): δ 7.98 (s, 2H), 7.75 – 7.64 (m, 2H), 7.38 (d, *J* = 7.0 Hz, 2H), 6.80 (s, 2H), 6.72 (d, *J* = 3.0 Hz, 1H), 6.49 (s, 1H), 4.59 (d, *J* = 14.0 Hz, 1H), 4.00 (d, *J* = 7.0 Hz, 1H), 3.57 – 3.06 (m, 2H), 2.89 (dd, *J* = 14.0, 3.0 Hz, 1H), 2.79 (d, *J* = 7.0 Hz, 1H), 2.44 (d, *J* = 11.0 Hz, 1H), 2.39 (s, *J* = 3.0 Hz, 3H), 2.33 (d, *J* = 14.0 Hz, 1H).

^{13}C NMR (125 MHz, Acetone-d_6): δ 157.7, 155.3, 145.2, 143.5, 143.2, 139.1, 136.3, 132.6, 130.0, 127.4, 126.7, 119.1, 117.8, 116.4, 108.9, 102.8, 50.2, 49.6, 32.9, 20.8.

HRMS (ESI) *m/z* calcd. for $C_{22}H_{21}NO_6S$ $(M+Na)^+$: 450.0982, found: 450.0987.

Enantiomeric excess of **2ar** is determined by HPLC (Chiralpak AD-H, Hexane/Isopropanol = 60/40, flow rate = 1.0 mL/min, 220 nm): major isomer: tr = 12.89min; minor isomer: tr = 5.30 min. $[\alpha]^{20}_{D}$ = -231.26 (c = 1.0, MeOH).

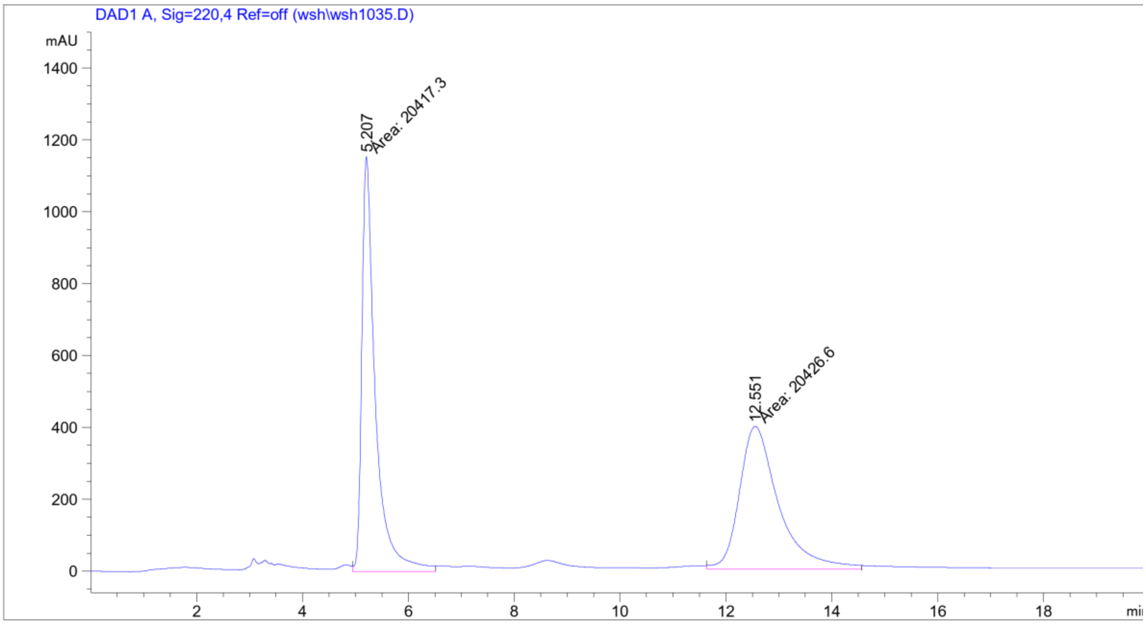

Signal 1: DAD1 A, Sig=220,4 Ref=off

Peak #	RetTime [min]	Type	Width [min]	Area [mAU*s]	Height [mAU]	Area %
1	5.207	MM	0.2950	2.04172e4	1153.35999	49.9885
2	12.551	MM	0.8576	2.04266e4	396.97253	50.0115
Totals :				4.08439e4	1550.33252	

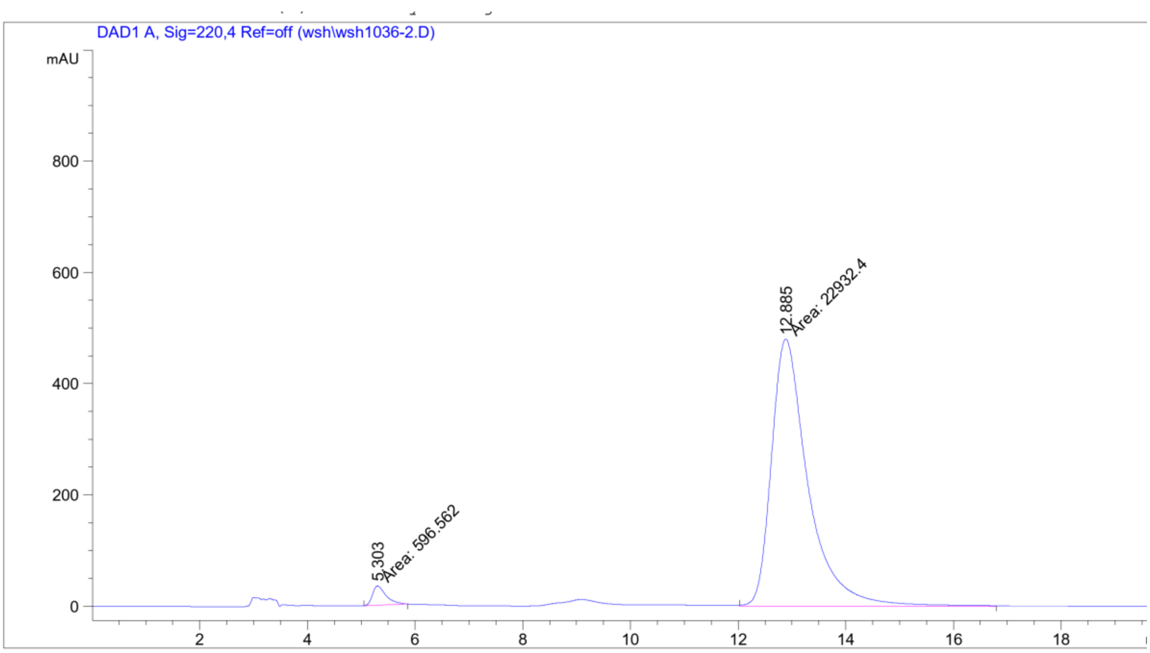

Signal 1: DAD1 A, Sig=220,4 Ref=off

Peak #	RetTime [min]	Type	Width [min]	Area [mAU*s]	Height [mAU]	Area %
1	5.303	MM	0.2878	596.56171	34.54997	2.5354
2	12.885	MM	0.7952	2.29324e4	480.61981	97.4646

Totals : 2.35290e4 515.16978

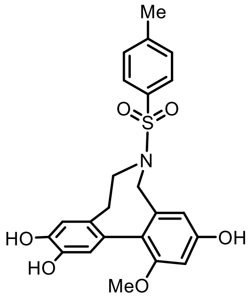

2as

According to the procedure, **2as** was obtained using **1as** (44.3 mg, 0.1 mmol) in 97% yield (43mg) and 91% ee, light gray foam solid, (silica gel flash chromatography: DCM/ acetone = 20:1). R_f = 0.40 (DCM/acetone = 10:1).

^{1}H NMR (500 MHz, Acetone-d_6): δ 7.68 (d, *J* = 8.0 Hz, 2H), 7.36 (d, *J* = 8.0 Hz, 2H), 6.84 (d, *J* = 2.0 Hz, 1H), 6.72 (s, 1H), 6.68 (s, 1H), 6.52 (d, *J* = 2.0 Hz, 1H), 4.57 (d, *J* = 14.0 Hz, 1H), 3.98

(dd, J = 12.0, 7.0 Hz, 1H), 3.68 (s, 3H), 3.07 (d, J = 42.0 Hz, 2H), 2.88 (d, J = 14.0 Hz, 1H), 2.76 (dd, J = 14.0, 7.0 Hz, 1H), 2.43 (t, J = 11.0 Hz, 1H), 2.37 (s, 3H), 2.25 (dd, J = 8.5, 6.0 Hz, 1H).

^{13}C NMR (125 MHz, Acetone-d_6): δ 157.7, 144.8, 143.2, 142.6, 138.7, 135.9, 131.8, 129.7, 127.1, 126.8, 120.5, 117.9, 115.5, 108.6, 99.0, 55.1, 49.8, 49.1, 32.6, 20.5.

HRMS (ESI) *m/z* calcd. for $C_{23}H_{23}NO_6S$ $(M+Na)^+$: 464.1138, found: 464.1137.

Enantiomeric excess of **2as** is determined by UPC^2 (CHIRALPAK® OD-3, CO_2/MeOH = 80/20, flow rate = 1.0 mL/min, 220 nm): major isomer: tr = 9.43 min; minor isomer: tr = 7.97 min. $[\alpha]^{20}_D$ = -292.60 (c = 1.0, MeOH)

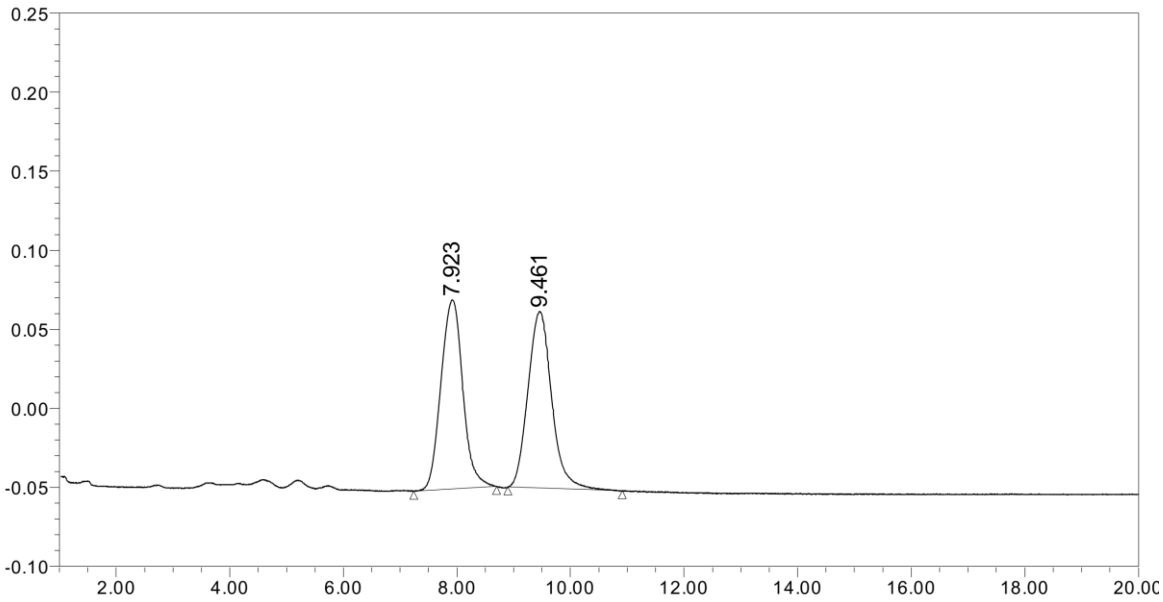

Signal: Sig = 220nm

Peak	RetTime	Area	Height	Area%
1	7.923	3181036	119526	49.27
2	9.461	3274659	111799	50.73

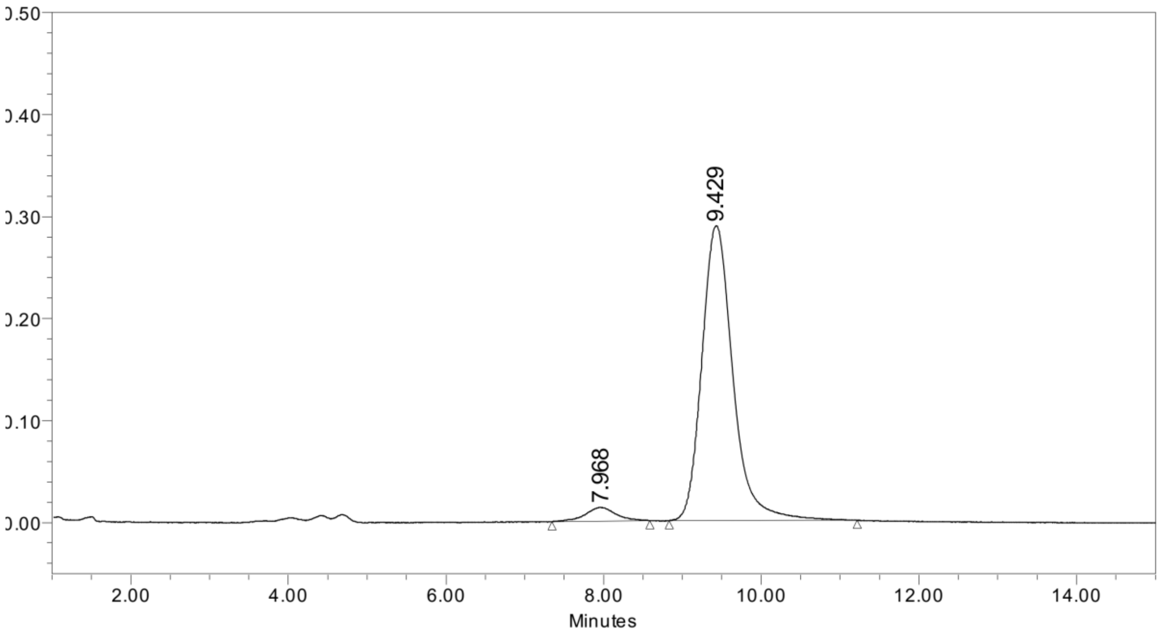

Peak	RetTime	Area	Height	Area%
1	7.968	365804	13593	4.41
2	9.429	7921152	288844	95.59

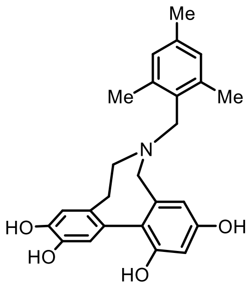

2at

According to the procedure, **2at** was obtained using **1at** (40.7 mg, 0.1 mmol) in 97% yield (39mg) and 93% ee, light gray foam solid, (silica gel flash chromatography: DCM/ acetone = 10:1). R_f = 0.40 (DCM/acetone = 10:1).

^{1}H NMR (500 MHz, Acetone-d_6): δ 6.84 (s, 2H), 6.79 (s, 1H), 6.76 (s, 1H), 6.35 (d, J = 2.0 Hz, 1H), 6.04 (d, J = 2.0 Hz, 1H), 3.63 (d, J = 12.5 Hz, 1H), 3.54 (d, J = 12.5 Hz, 1H), 3.39 (d, J = 13.0 Hz, 1H), 3.07 (d, J = 11.0 Hz, 1H), 2.87 (d, J = 13.0 Hz, 1H), 2.55 – 2.47 (m, 1H), 2.32 (d, J = 5.5 Hz, 8H), 2.24 (s, 3H).

^{13}C NMR (125 MHz, Acetone-d_6): δ 157.3, 155.0, 145.2, 142.9, 142.3, 138.4, 136.4, 135.0, 133.4, 129.3, 126.9, 119.1, 117.6, 116.6, 109.6, 101.9, 56.8, 55.7, 55.1, 33.2, 20.7, 20.0.

HRMS (ESI) *m/z* calcd. for $C_{25}H_{27}NO_4(M+H)^+$: 406.2013, found: 406.2009.

Enantiomeric excess of **2at** is determined by HPLC (Chiralpak AD-H, Hexane/Isopropanol = 60/40, flow rate = 1.0 mL/min, 220 nm): major isomer: tr = 4.27 min; minor isomer: tr = 5.29 min. $[\alpha]^{20}_{D}$ = 3.87 (c = 1.0, MeOH).

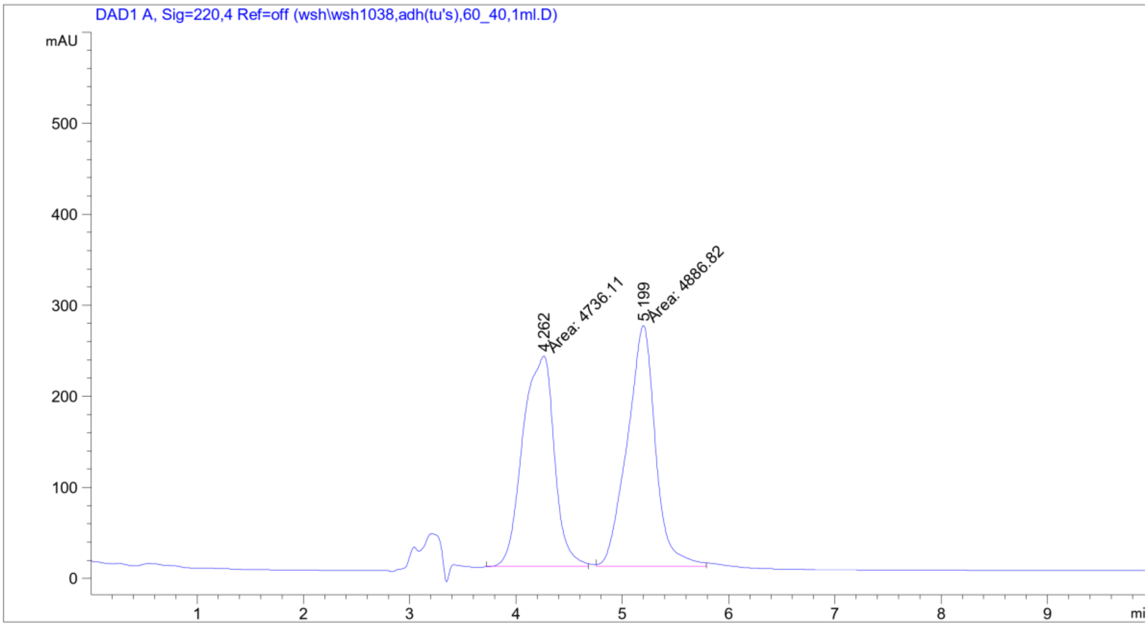

Signal 1: DAD1 A, Sig=220,4 Ref=off

Peak #	RetTime [min]	Type	Width [min]	Area [mAU*s]	Height [mAU]	Area %
1	4.262	MM	0.3420	4736.10986	230.78632	49.2169
2	5.199	MM	0.3081	4886.81689	264.33850	50.7831

Totals : 9622.92676 495.12482

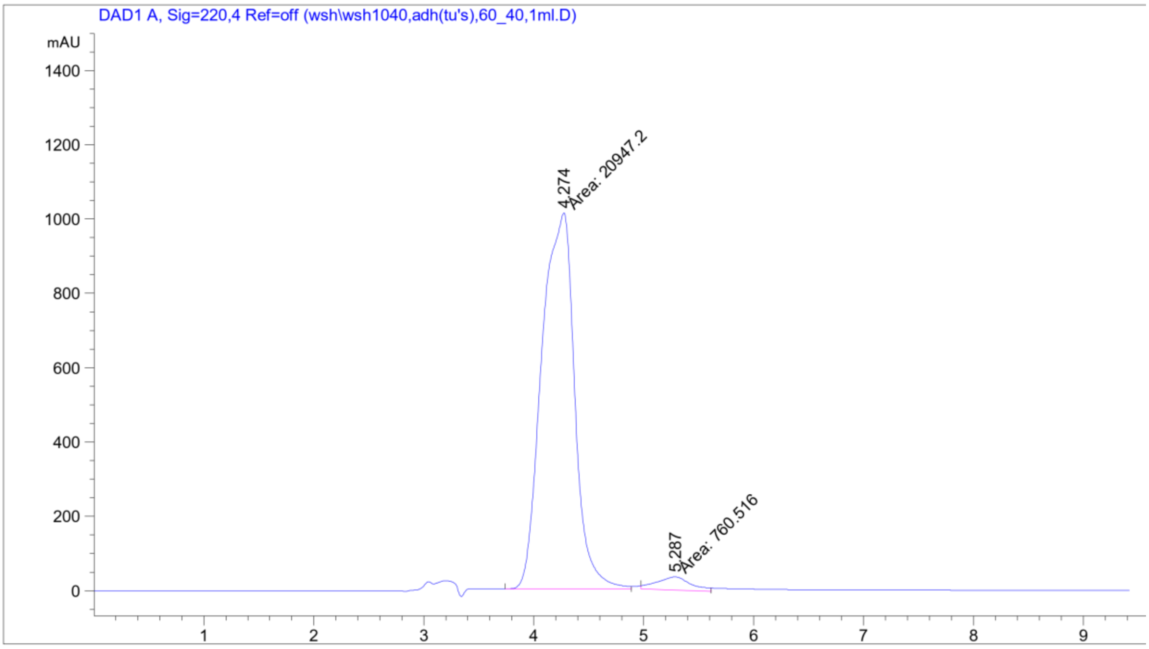

Signal 1: DAD1 A, Sig=220,4 Ref=off

Peak #	RetTime [min]	Type	Width [min]	Area [mAU*s]	Height [mAU]	Area %
1	4.274	MM	0.3451	2.09472e4	1011.67847	96.4966
2	5.287	MM	0.3585	760.51593	35.35355	3.5034

Totals : 2.17077e4 1047.03202

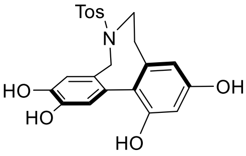

2az

According to the procedure, **2az** was obtained using **1az** (43.0 mg, 0.1 mmol) in 86% yield (37mg) and 92% ee, light gray foam solid, (silica gel flash chromatography: DCM/ MeOH = 10:1). R_f = 0.40 (DCM/MeOH = 10:1).

^{1}H NMR (400 MHz, CD_3OD) δ 7.62 (d, J = 8.2 Hz, 2H), 7.32 (d, J = 8.1 Hz, 2H), 7.14 (s, 1H), 6.74 (s, 1H), 6.24 (d, J = 2.1 Hz, 1H), 6.14 (d, J = 2.2 Hz, 1H), 4.91 (s, 4H), 4.56 (d, J = 14.0 Hz, 1H), 3.97 (dd, J = 11.8, 7.6 Hz, 1H), 2.88 (d, J = 14.0 Hz, 1H), 2.68 (dd, J = 13.9, 7.5 Hz, 1H), 2.43 (t, J = 5.2 Hz, 1H), 2.37 (s, 3H), 2.28 – 2.15 (m, 1H).

^{13}C NMR (100 MHz, CD_3OD) δ 159.5, 158.7, 156.3, 145.9, 145.5, 144.8, 143.7, 136.9, 130.9, 130.8, 129.9, 128.9, 128.2, 119.5, 118.5, 117.9, 108.4, 108.0, 102.1, 50.8, 50.2, 34.5, 21.4.

HRMS (ESI) *m/z* calcd. for $C_{22}H_{21}NO_6S$ $(M+Na)^+$: 450.0982, found: 450.0987.

Enantiomeric excess of **2az** is determined by UPC^2 (CHIRALPAK® IG-3, CO_2/MeOH = 60/40, flow rate = 1.0 mL/min, 220 nm): major isomer: tr = 1.61 min; minor isomer: tr = 2.37 min. $[\alpha]^{20}_D$ = -1.30 (c = 1.0, MeOH)

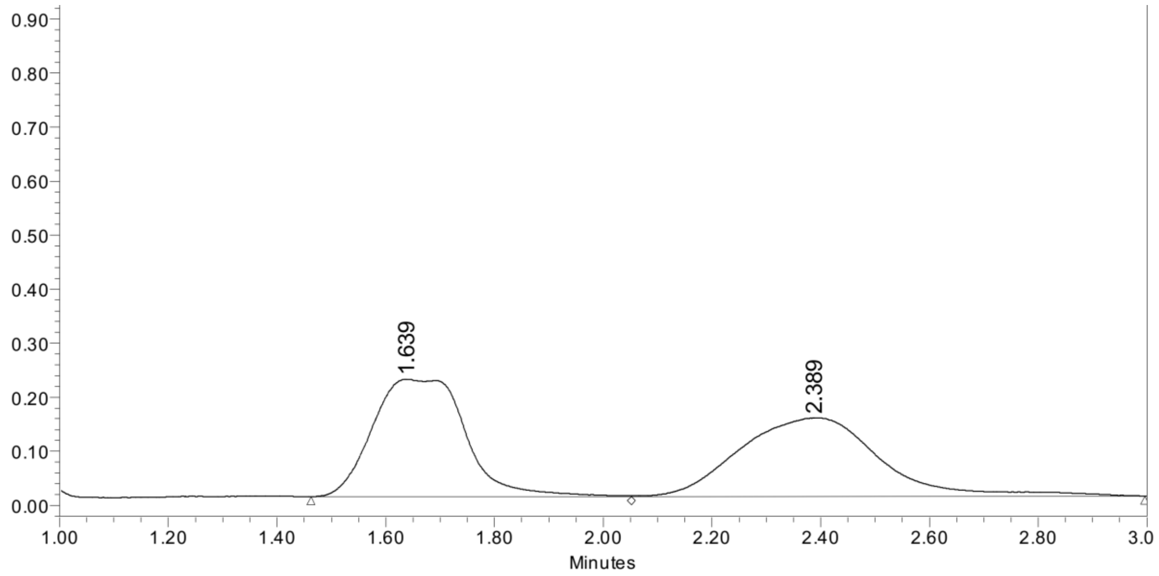

Signal: Sig = 220nm

Peak	RetTime	Area	Height	Area%
1	1.639	2573122	217089	49.87
2	2.389	2586691	145145	50.13

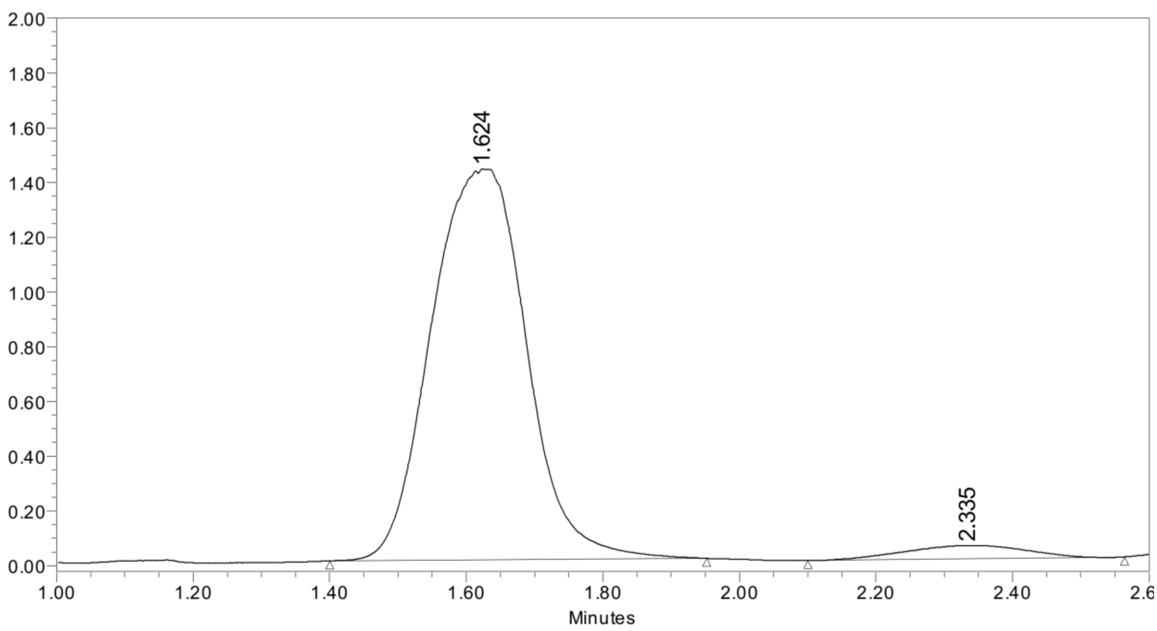

Peak	RetTime	Area	Height	Area%
1	1.624	13834754	1427832	95.92
2	2.335	588860	48076	4.08

9. Preparation of 3a:

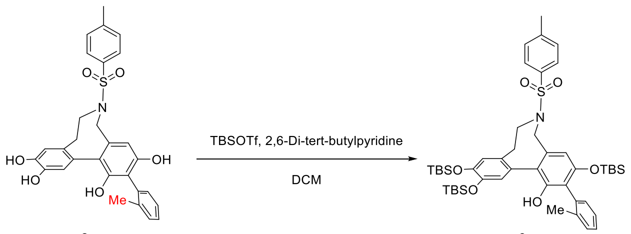

Supplementary Fig. 11: procedure for **3a** preparation

Procedure for **3a** preparation (**Supplementary Fig. 11**): Under argon atmosphere, **2a** (20 mg, 1.0 equiv) was dissolved in dry DCM at 0 ℃, 2,6-Di-tert-butylpyridine (0.1Ml, 12 equiv) and TBSOTf (53 μL, 6.0 equiv) was added subsequently. The resulted solution was stirred for 0.5 hours, then the reaction was quenched by addition of a saturated solution of $NaHCO_3$ (10 mL) then the system was extracted with DCM (3 × 20 mL). The combined organic layer was wash with brine, dried over Na_2SO_4 and concentrated under vacuum and chromatographied on silica gel (petroleum ether/ethyl acetate = 30:1 as eluent) to give **3a**(28mg, 85%).

The **3a** was obtained as colorless foam solid. R_f = 0.70 (petroleum ether/ethyl acetate = 8:1).

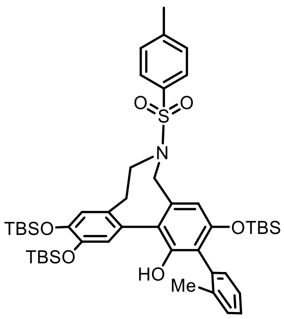

3a

^{1}H NMR (400 MHz, $CDCl_3$) δ 7.71 (d, *J* = 8.2 Hz, 2H), 7.40 – 7.18 (m, 6H), 7.06 (d, *J* = 3.8 Hz, 1H), 6.86 (s, 1H), 6.71 (s, 1H), 4.72 (d, J = 14.0 Hz, 1H), 4.10 (t, *J* = 9.0 Hz, 1H), 3.06 (d, *J* = 14.0 Hz, 1H), 2.86 – 2.72 (m, 1H), 2.64 – 2.51 (m, 2H), 2.45 (s, 3H), 2.27 (s, 3H), 1.08 – 0.92 (m, 18H), 0.72 (s, 9H), 0.29 – 0.11 (m, 18H).

^{13}C NMR (100 MHz, $CDCl_3$) δ 153.1, 150.2, 146.8, 145.0, 143.0, 138.2, 137.1, 136.1, 133.8, 133.0, 131.1, 130.1, 129.6, 128.0, 127.2, 127.1, 125.9, 122.6, 122.3, 119.4, 119.2, 112.9, 49.4, 49.2, 33.4, 25.8, 25.2, 21.4, 19.7, 18.4, 18.3, 17.7, -4.0, -4.1, -4.2, -4.2, -4.5, -4.5.

HRMS (ESI) *m/z* calcd. for $C_{47}H_{69}NO_6SSi_3$ $(M{-}H)^-$: 858.4081, found: 858.4044. $[\alpha]^{20}_D$ = -55.6 (c = 1.0, DCM).

Enantiomeric excess of **3a** is determined by HPLC (Chiralpak IE, Hexane/Isopropanol = 98/2, flow rate = 1.0 mL/min, 220 nm): major isomer: tr = 10.28 min; minor isomer: tr = 7.99 min.

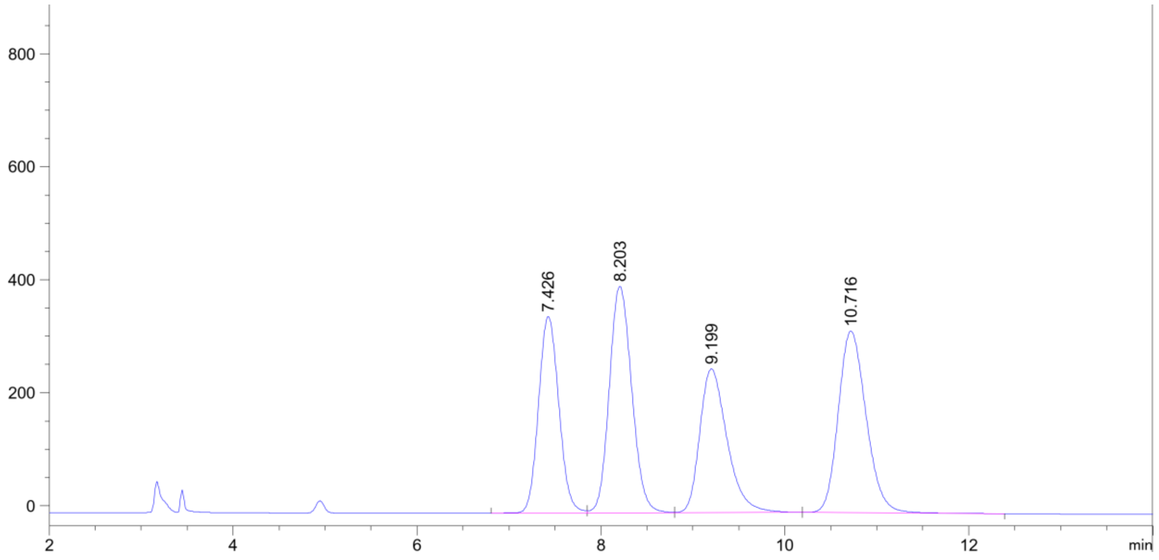

Signal 1: DAD1 A, Sig=220,4 Ref=off

Peak #	RetTime [min]	Type	Width [min]	Area [mAU*s]	Height [mAU]	Area %
1	7.426	BV	0.2415	5352.56201	347.96912	22.1301
2	8.203	VV	0.2661	6811.80518	401.07474	28.1633
3	9.199	VB	0.3185	5249.73828	254.64876	21.7050
4	10.716	BB	0.3277	6772.70947	321.56598	28.0017
Totals :				2.41868e4	1325.25859	

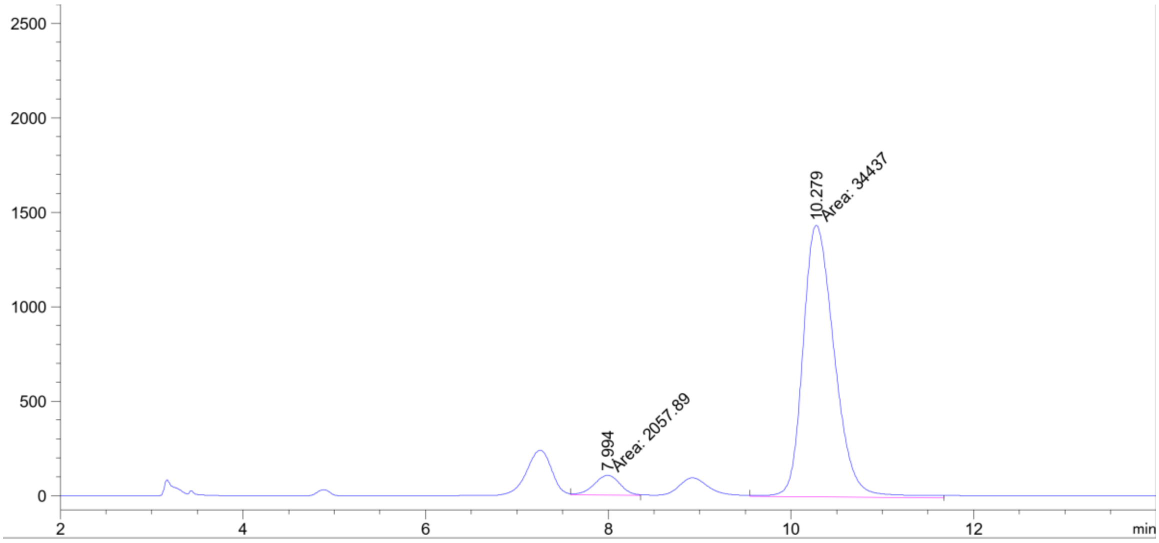

Signal 1: DAD1 A, Sig=220,4 Ref=off

Peak #	RetTime [min]	Type	Width [min]	Area [mAU*s]	Height [mAU]	Area %
1	7.994	MM	0.3297	2057.88647	104.02133	5.6388
2	10.279	MM	0.3994	3.44370e4	1437.09216	94.3612
Totals :				3.64949e4	1541.11349	

10. Mechanistic studies:

The oxidation process was first confirmed. Autoxidation of Co(II)/SPDO under aerobic conditions is evident by UV-visible spectroscopy. Bubbling O_2 through a Acetone/EtOH solution of Co(II)/SPDO resulted in an observed new absorption band, indicating oxidation of

Co(II)/SPDO (orange Line)(**Supplementary Fig. 12**). Additionally, the resulting EPR spectrum reveals a signal of organic free radicals (Co(III)-superoxide) (**Supplementary Fig. 13**). Next, abstraction of a hydrogen atom (HAT) and an ensuing proton-coupled-electron transfer (PCET) process from catechol by cobalt-superoxide complex **I** and generation of a highly active *o*-benzoquinone **II** was proposed based on known literatures.

a)

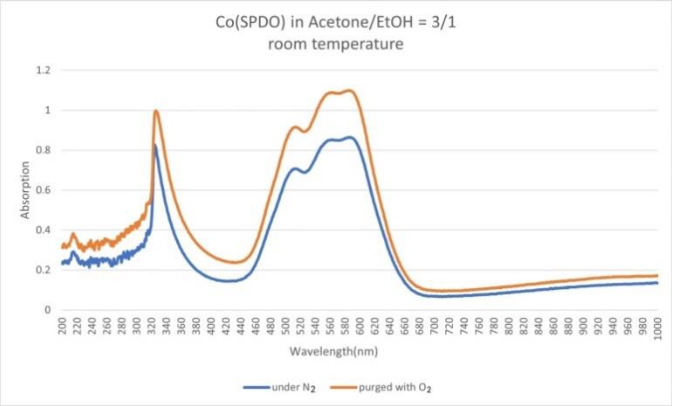

Supplementary Fig. 12. UV-visible spectra of Co/SPDO dissolved in Acetone/EtOH at room temperature under N_2 (blue line), after bubbling O_2 through the solution (orange Line).

b)

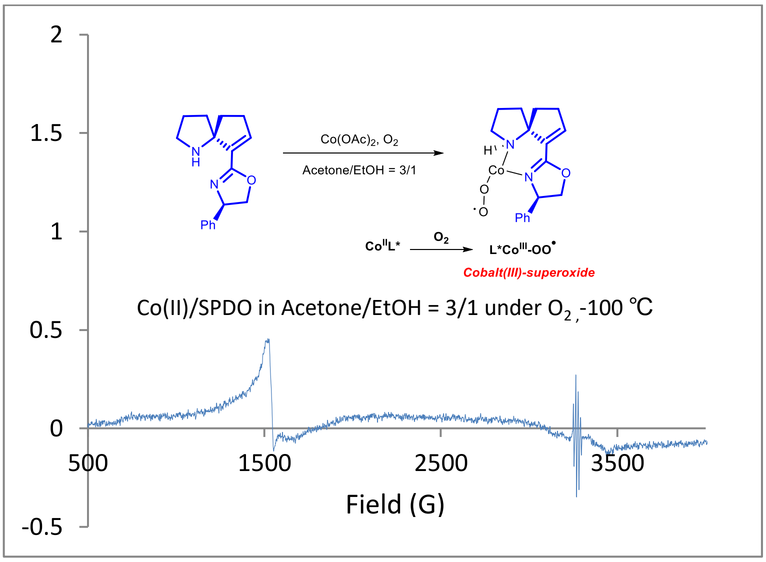

Supplementary Fig. 13 X-band EPR spectra of Co/SPDO (1 mM) in O_2-saturated Acetone/EtOH = 3/1 at -100 ^oC.

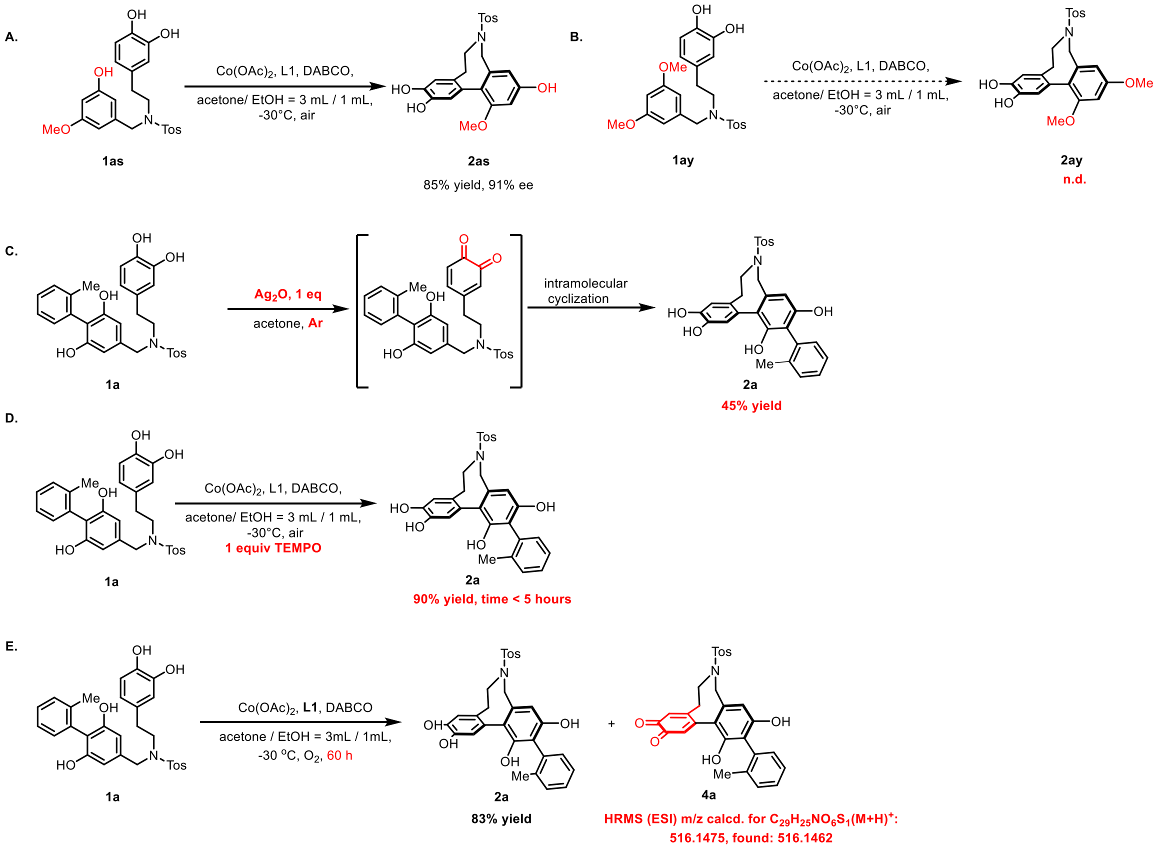

Supplementary Fig. 14. Control experiments

Second, to further validate the proposed reaction mechanism, some control experiments were conducted (**Supplementary Fig. 14**). Monomethyl-protected substrate **1as** on resorcinol could deliver the desired product with good yield and enantioselectivity **(Supplementary Fig. 14-A).** However, the dimethyl-protected substrate **1ay** couldn't undergo the cyclization process and offer the expected product due to the lower nucleophilicity **(Supplementary Fig. 14-B)**. On the basis of previous reports[3-5], silver oxide can be used to oxidize catechol instead of resorcinol to *o*-benzoquinone. In order to prove this process via the intermediate of *o*-benzoquinone, **1a** was first oxidized by silver oxide. As expected, the target product **2a** was obtained in 45% yield in the presence of 1 equivalent of silver oxide, which indicated that a highly active *o*-benzoquinone **II** was formed followed by 8-exo-trig cycloaddition to deliver intermediate **III** with both central and axial chiralities. Finally, the desired product **2** was produced after aromatization. It is worth noting that phenols cannot produce free radicals through silver oxide excluding the process involving radicals **(Supplementary Fig. 14-C)**. The process was also confirmed by free radical capture experiment. The addition of TEMPO only accelerated the reaction and could not prevent it from

proceeding **(Supplementary Fig. 14-D)**. In addition, to confirm that the catalytic cycle also involve *o*-benzoquinone intermediate **II**, we conducted an over oxidation experiment on compound **1a**, the product **2a** (83% yield) and the over oxidation product **4a** confirmed by **HRMS** (**Supplementary Fig. 15**), was obtained as expected, which is very unstable and cannot be isolated, that indirectly proved the process via the *o*-benzoquinone intermediate **II** **(Supplementary Fig. 14-E)**.

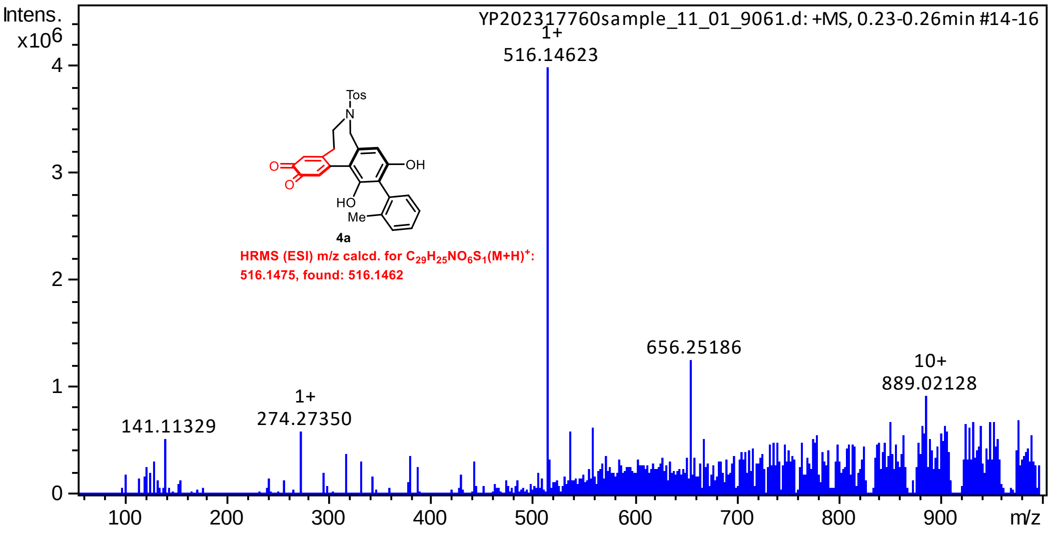

Supplementary Fig. 15. HRMS-ESI for the over oxidation product **4a**

11. X-ray crystallographic data for 2ad and 3a.

Crystal data and structure refinement for **2ad** (**Supplementary Table 1**) and **3a** (**Supplementary Table 2**): the crystals of **2ad** and **3a** were grown from a solvent mixture of dichloromethane to acetone in a ratio of 3:1 at 0°C. The single-crystal X-ray diffraction data were collected on a Bruker D8 VENTURE CMOS Photon II diffractometer with helios mx multilayer monochrmator Cu Kα radiation (λ = 1.54178 Å) at 173 K in the Instrumental Analysis Center of Shanghai Jiao Tong University. Data collection, unit cell refinement and data reduction were performed using APEX3 v2019.11-0. All the structures were solved using direct method, and refined by full-matrix least-squares on F 2 by the SHELXL-2018/3 software package[6]. The hydrogen atoms on carbon were calculated in ideal positions with isotropic displacement parameters set to 1.2xUeq of the attached atom (1.5xUeq for methyl hydrogen atoms). The contributions of highly disordered solvents were removed using

SQUEEZE routine PLATON software or the solvent mask[7] program of Olex2[8]. In the asymmetric Unit, 8.3 electrons have been masked. This is consistent with 1 H_2O.

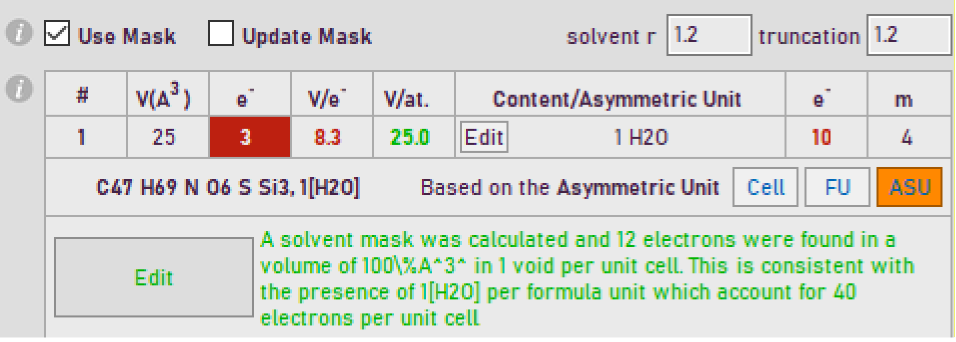

The absolute configuration of compound **2ad**, **3a** is determined by the absolute structure parameter, with values of 0.032(13) and 0.028(17), respectively. The value of the Flack parameter is close to zero within a region of three standard uncertainties i.e. u < 0.04 (or u < 0.10 for a chemically proven enantiomeric excess of 100%) and |x|/u < 3.0. So the absolute structure of the crystal has been determined satisfactorily[9].

There a B-level alert in CheckCif file of **2ad** and **3a**, respectively. We check again that there is no hydrogen bond acceptor around H6 of **3a** and H2 of **2ad** using the olex 2 software package. The acceptor here may be the pi systems that is flanking the phenolic groups or that sterics imposed on the phenolic donor impede any traditional acceptor.

Crystal data and structure refinement for 2ad

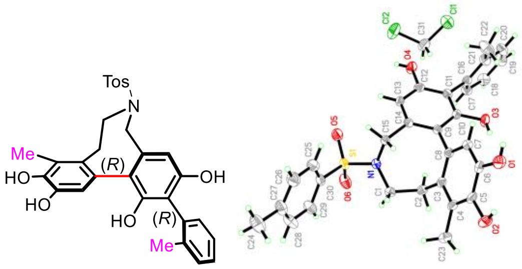

CCDC 2244054

Probability ellipsoids 50%

Supplementary Table 1. Crystal data and structure refinement for 2ad.

Identification code	**2ad**
Empirical formula	C31 H31 Cl2 N O6 S
Formula weight	616.53
Temperature	173(2) K

Wavelength	1.54178 Å
Crystal system	Orthorhombic
Space group	$P2_12_12_1$
Unit cell dimensions	a = 9.7589(2) Å α= 90 °.
	b = 11.2087(2) Å β= 90 °.
	c = 26.2484(4) Å γ = 90 °.
Volume	2871.17(9) $Å^3$
Z	4
Density (calculated)	1.426 Mg/m^3
Absorption coefficient	3.100 mm^{-1}
F(000)	1288
Crystal size	0.180 x 0.160 x 0.140 mm^3
Theta range for data collection	4.289 to 68.593 °.
Index ranges	-11<=h<=11, -13<=k<=13, -31<=l<=31
Reflections collected	32074
Independent reflections	5273 [R(int) = 0.0383]
Completeness to theta = 67.679 °	99.8 %
Absorption correction	Semi-empirical from equivalents
Max. and min. transmission	0.7531 and 0.5897
Refinement method	Full-matrix least-squares on F^2
Data / restraints / parameters	5273 / 0 / 386
Goodness-of-fit on F^2	1.031
Final R indices [I>2sigma(I)]	R1 = 0.0257, wR2 = 0.0666
R indices (all data)	R1 = 0.0269, wR2 = 0.0674
Absolute structure parameter	0.032(13)
Extinction coefficient	n/a
Largest diff. peak and hole	0.275 and -0.312 e.$Å^{-3}$

Crystal data and structure refinement for 3a

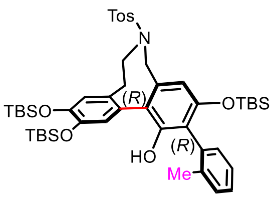

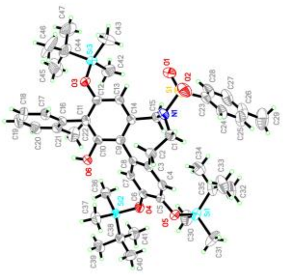

CCDC 2244056

Probability ellipsoids 50%

Supplementary Table 2. Crystal data and structure refinement for 3a.

Identification code	**3a**	
Empirical formula	C47 H71 N O7 S Si3	
Formula weight	878.37	
Temperature	173.00 K	
Wavelength	1.54178 Å	
Crystal system	Orthorhombic	
Space group	$P2_12_12_1$	
Unit cell dimensions	a = 6.1935(2) Å	a= 90 °.
	b = 17.5858(6) Å	b= 90 °.
	c = 45.0611(14) Å	g = 90 °.
Volume	4907.9(3) $Å^3$	
Z	4	
Density (calculated)	1.189 Mg/m^3	
Absorption coefficient	1.667 mm^{-1}	
F(000)	1896	
Crystal size	0.200 x 0.180 x 0.160 mm^3	
Theta range for data collection	1.961 to 68.663 °.	
Index ranges	-7<=h<=7, -21<=k<=21, -53<=l<=54	
Reflections collected	38406	
Independent reflections	9036 [R(int) = 0.0550]	
Completeness to theta = 67.679 °	100.0 %	

Absorption correction	Semi-empirical from equivalents
Max. and min. transmission	0.7531 and 0.5928
Refinement method	Full-matrix least-squares on F^2
Data / restraints / parameters	9036 / 0 / 542
Goodness-of-fit on F^2	1.041
Final R indices [I>2sigma(I)]	R1 = 0.0329, wR2 = 0.0813
R indices (all data)	R1 = 0.0377, wR2 = 0.0847
Absolute structure parameter	0.028(17)
Extinction coefficient	n/a
Largest diff. peak and hole	0.220 and -0.214 e.Å^{-3}

12. Unsuccessful substrates

Some unsuccessful substrates (**Supplementary Fig. 16**).

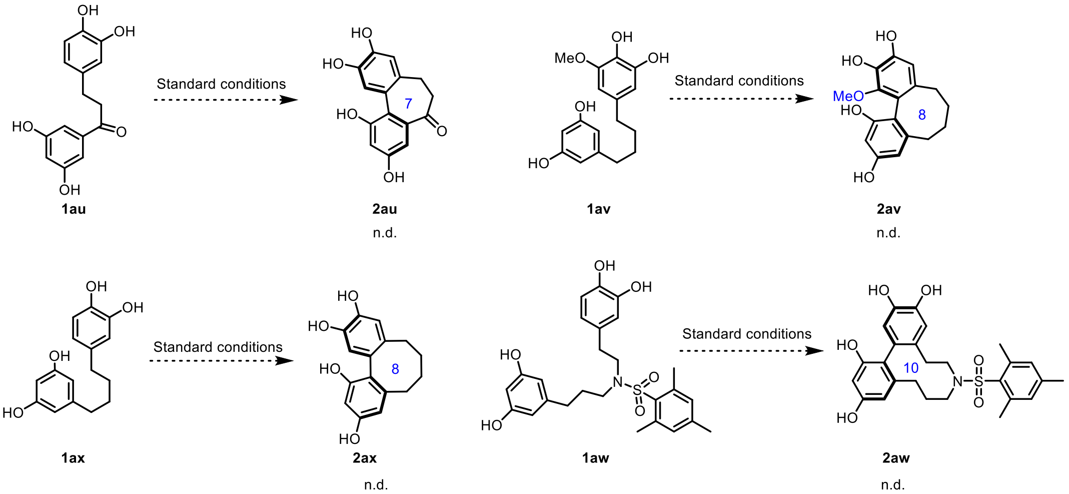

Supplementary Fig. 16: Exploration of oxidative coupling of **1au-1ax**

13. Gram scale reaction and transformation

As shown in the following **Supplementary Fig. 17**, we have conducted a gram-scale aerobic oxidative coupling of phenol **1a** using 5% Co(II)/SPDO catalyst and obtained compound **2a** with a similar yield and ee value. Next, we conducted some synthetic applications. First, we performed TBS protection of product **2a** and obtained compound **3a** with 85% yield and 89% ee. Second, we attempted to remove the protecting group on nitrogen of **2a** and **3a** but failed to obtain

the corresponding products.

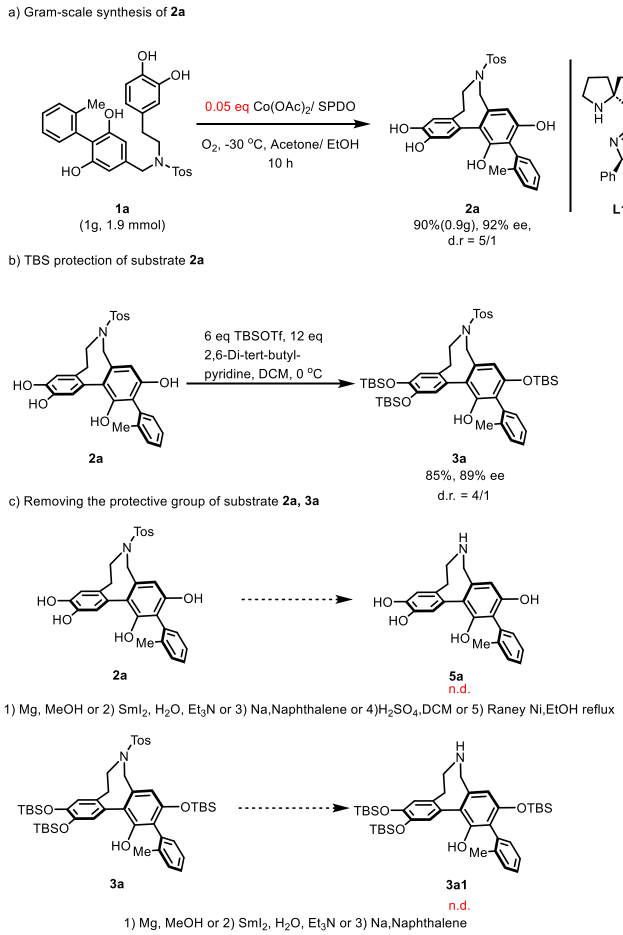

Supplementary Fig. 17: Gram scale reaction of **1a** and transformation of **2a**

14. Stability testing

Compounds **2al** and **2ar** are very stable at -18 °C. However, at room temperature, the diastereoselectivity of **2al** would change from 24/1 to 1.8/1 in methanol solution during a period of 96 hours. Meanwhile, the enantioselectivity of the major product remains the same and that of the minor product greatly enhances. These results indicate that one of the axials could rotate at ambient temperature, leading to an epimerization process. Next, we determined that the bridged axis was unstable by examining the stability of compound **2ar**. Finally, stability test of **2t** at different temperatures indicated that epimerization begins at around the 16 ^{o}C.

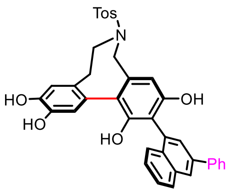

2al

entry	T(°C)	Time (h)	Major ee(%)[a]	Minor ee(%)[a]	dr[a]	solvent
1	25	0	98	-30	24/1	
2	25	24	98	51	13/1	
3	25	48	98	83	8/1	MeOH
4	25	72	98	91	3/1	
5	25	96	95	95	1.8/1	
6	-18	30 days	98	-30	24/1	

[a] ee and dr values were determined by HPLC analysis using a chiral stationary phase

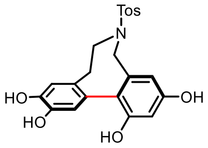

2ar

entry	T(°C)	Time (h)	ee(%)[a]	solvent
1	25	0	95	
2	25	24	88	
3	25	48	87	MeOH
4	25	120	78	
5	-18	30 days	95	

[a] ee values were determined by HPLC analysis using a chiral stationary phase

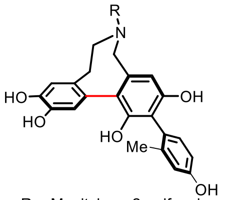

R = Mesitylene-2-sulfonyl

2t

entry	T(°C)	Time (h)	Major ee(%)[a]	Minor ee(%)[a]	dr[a]	solvent
1	-18	12	94	-12	10/1	
2	-13	12	94	-12	10/1	
3	-8	12	94	-12	10/1	
4	-6	12	94	-12	10/1	
5	-2	12	94	-12	10/1	
6	0	12	94	-12	10/1	
7	2	12	94	-12	10/1	MeOH
8	6	12	94	-12	10/1	
9	8	12	94	-12	10/1	
10	10	12	94	-12	10/1	
11	12	12	94	-12	10/1	
12	16	12	94	30	7/1	
13	25	60	92	70	3.3/1	

[a] ee and dr values were determined by HPLC analysis using a chiral stationary phase

15. Copies of NMR spectra

^{1}H NMR spectrum of compound 1a (500 MHz) in CDCl$_3$

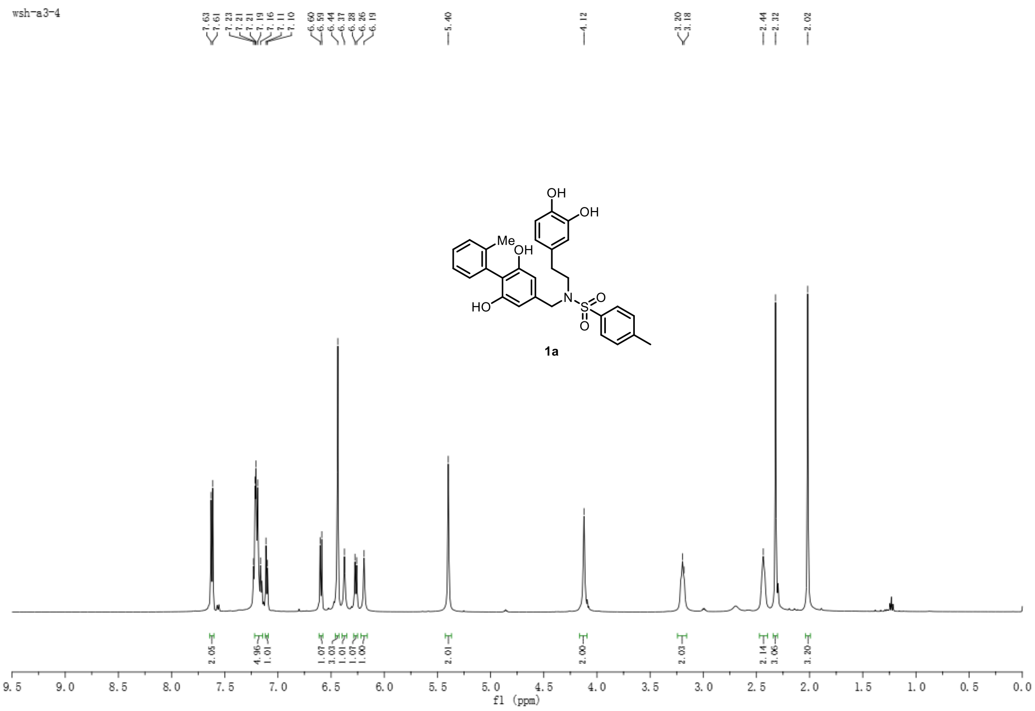

^{13}C NMR spectrum of compound 1a (125 MHz) in CDCl$_3$

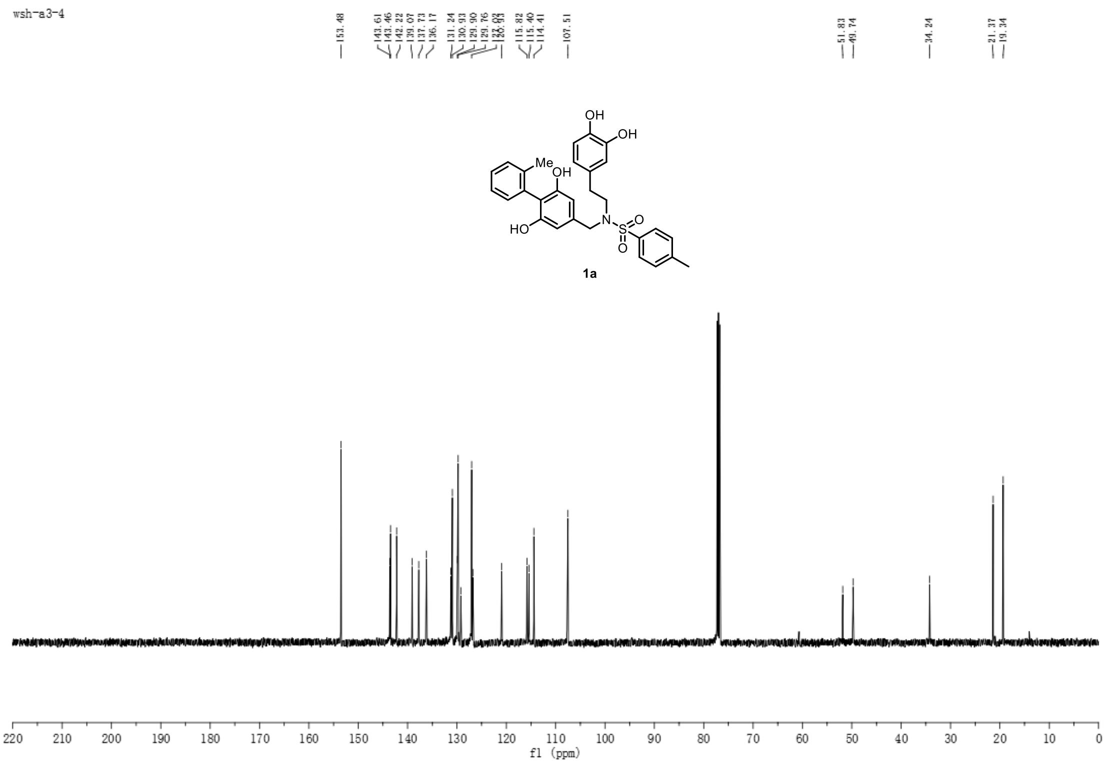

1H NMR spectrum of compound 1b (500 MHz) in CD_3OD

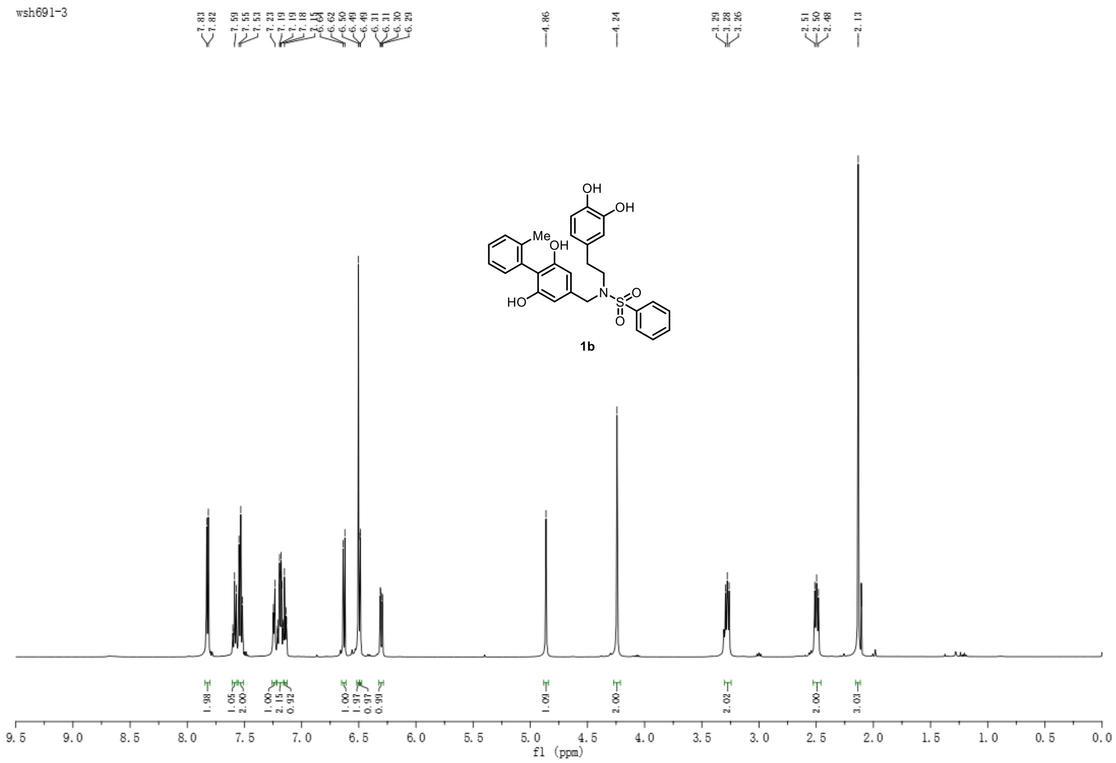

^{13}C NMR spectrum of compound 1b (125 MHz) in CD_3OD

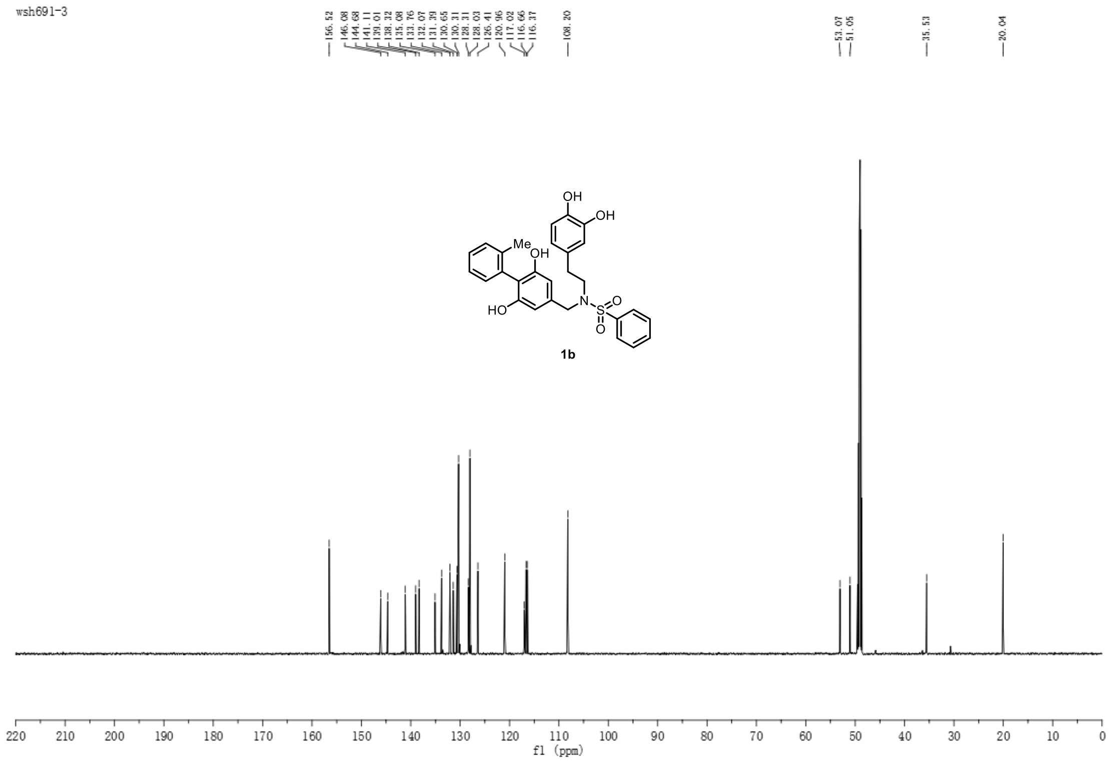

^{1}H NMR spectrum of compound 1c (500 MHz) in Acetone-d_6

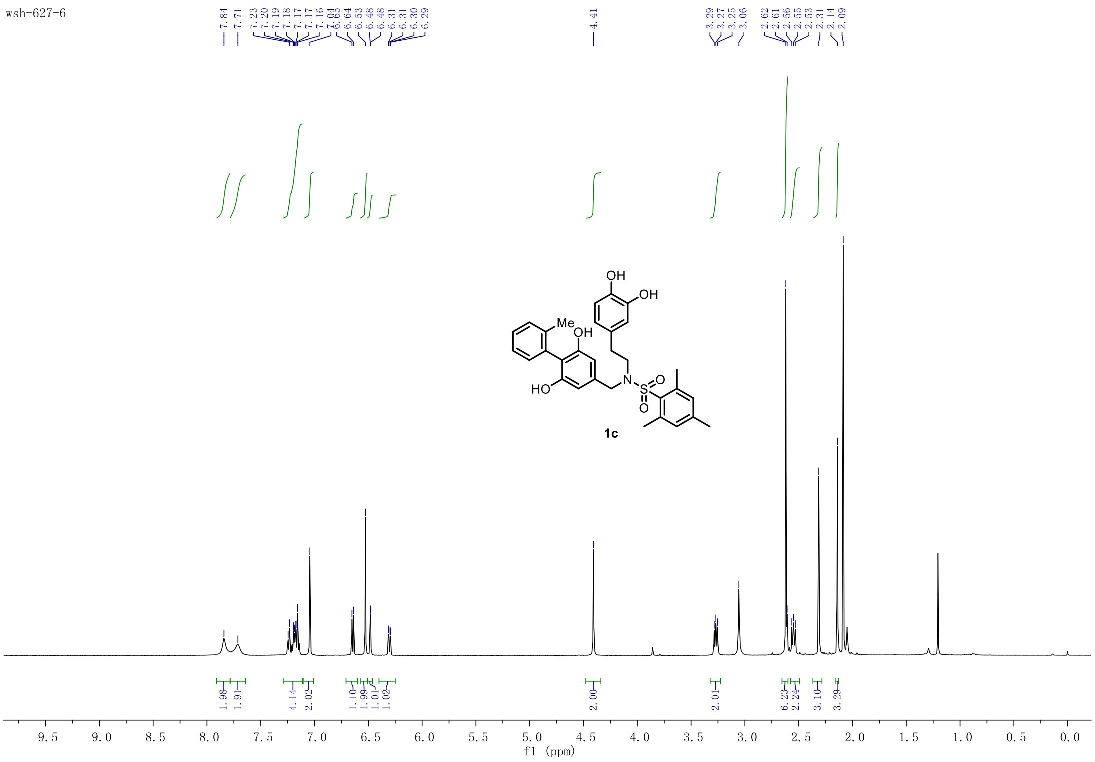

^{13}C NMR spectrum of compound 1c (125 MHz) in Acetone-d_6

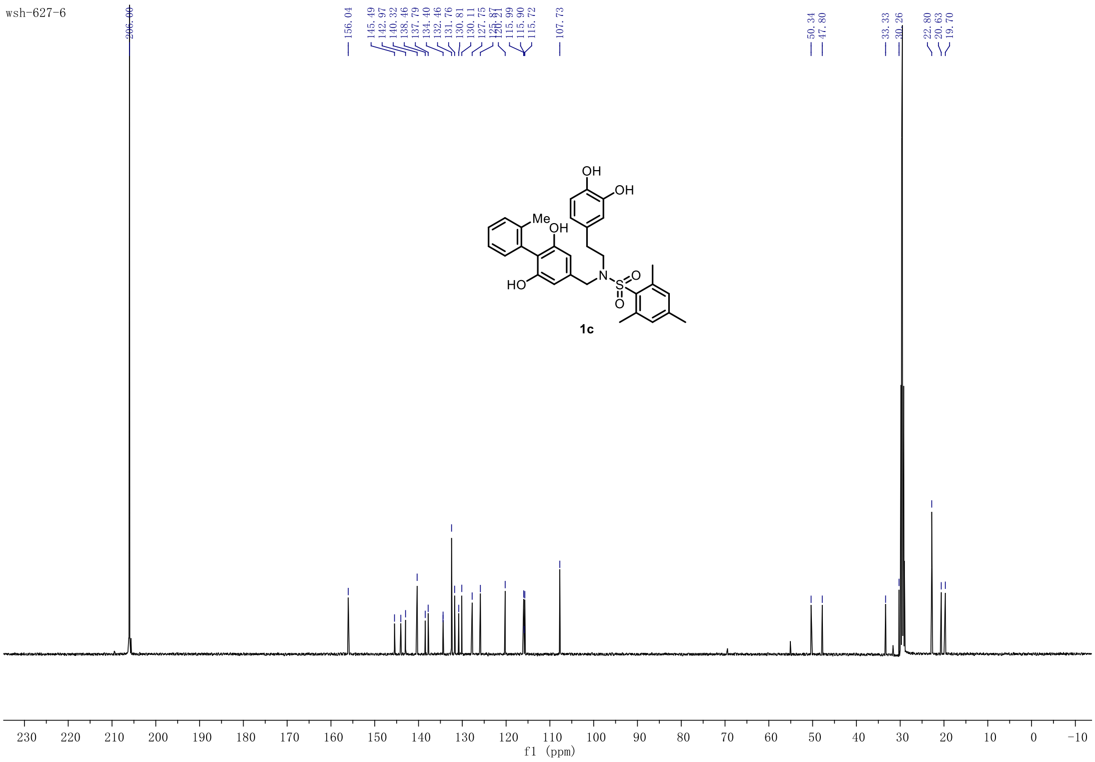

^{1}H NMR spectrum of compound 1d (500 MHz) in CD_3OD

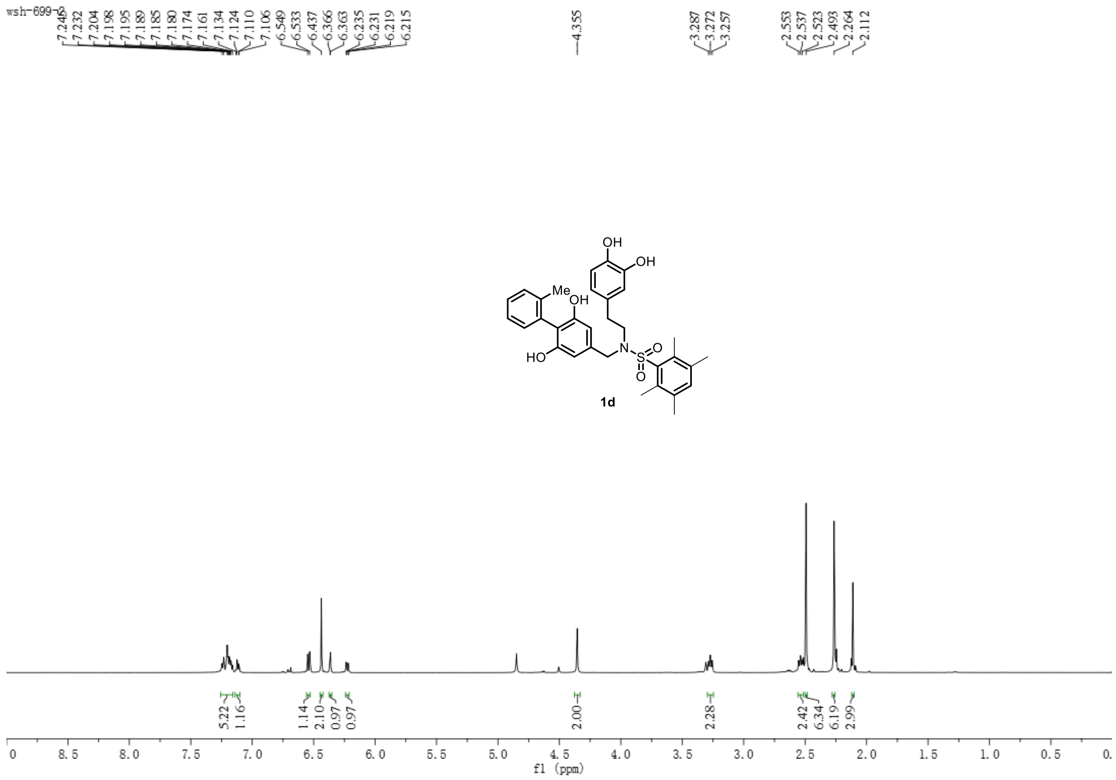

^{13}C NMR spectrum of compound 1d (125 MHz) in CD_3OD

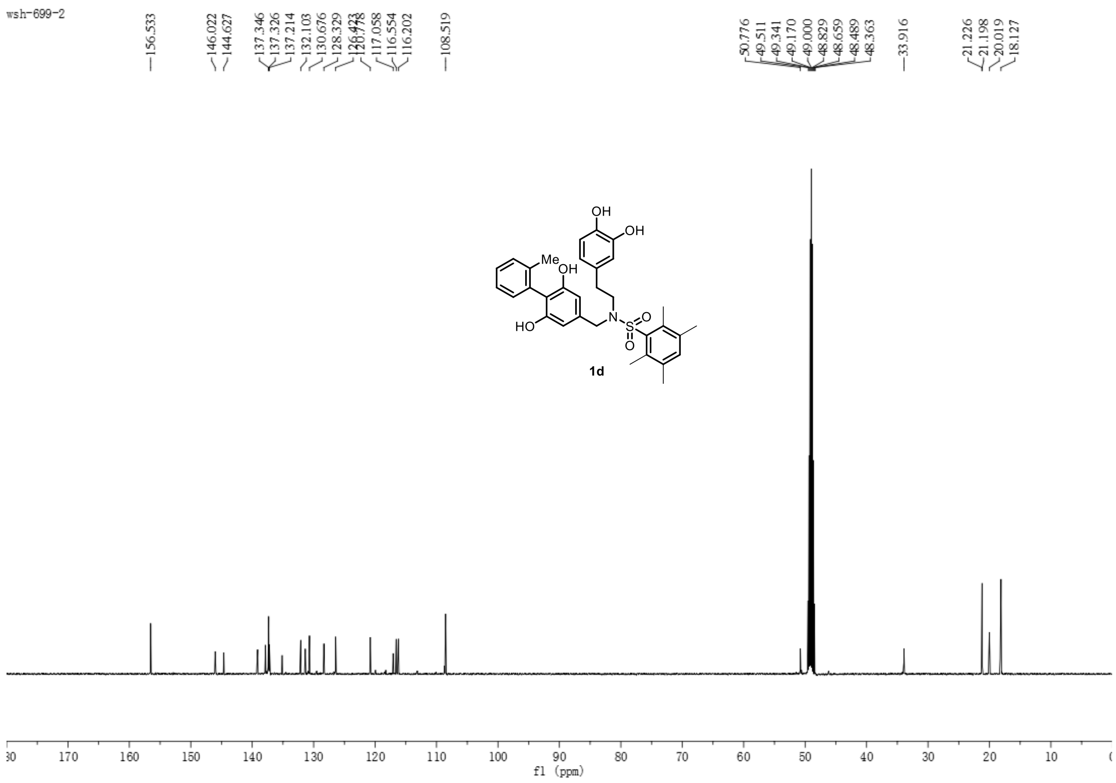

^{1}H NMR spectrum of compound 1e (500 MHz) in CD_3OD

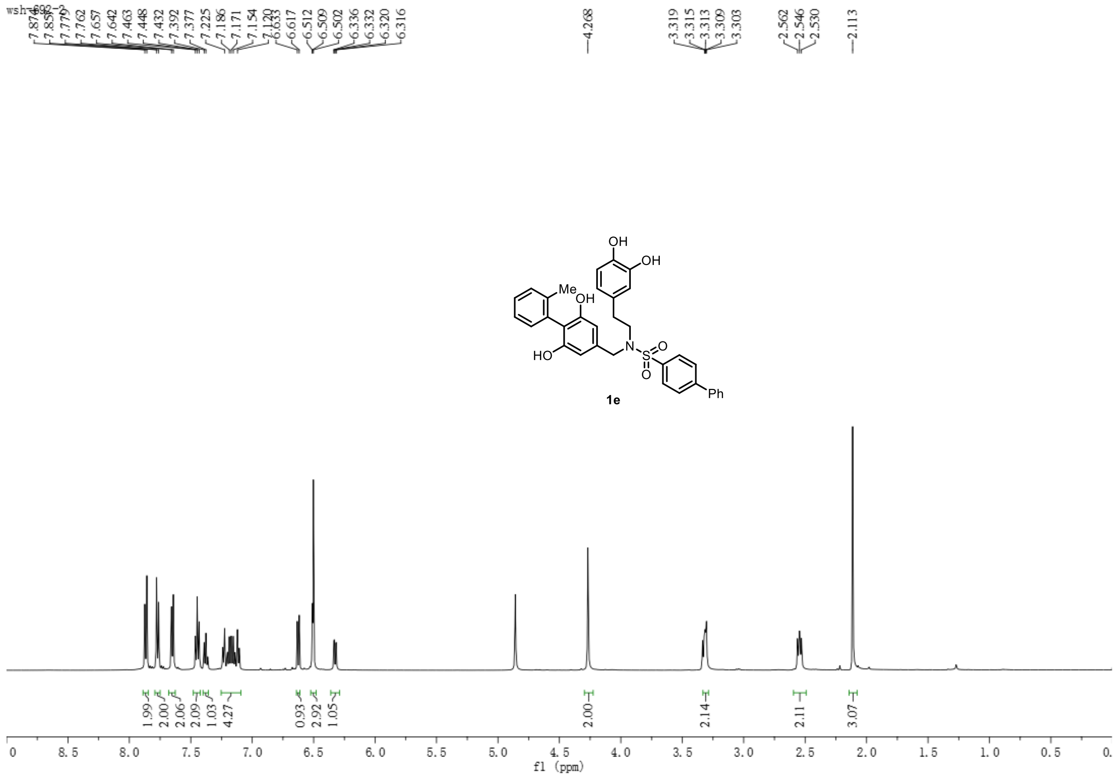

^{13}C NMR spectrum of compound 1e (125 MHz) in CD_3OD

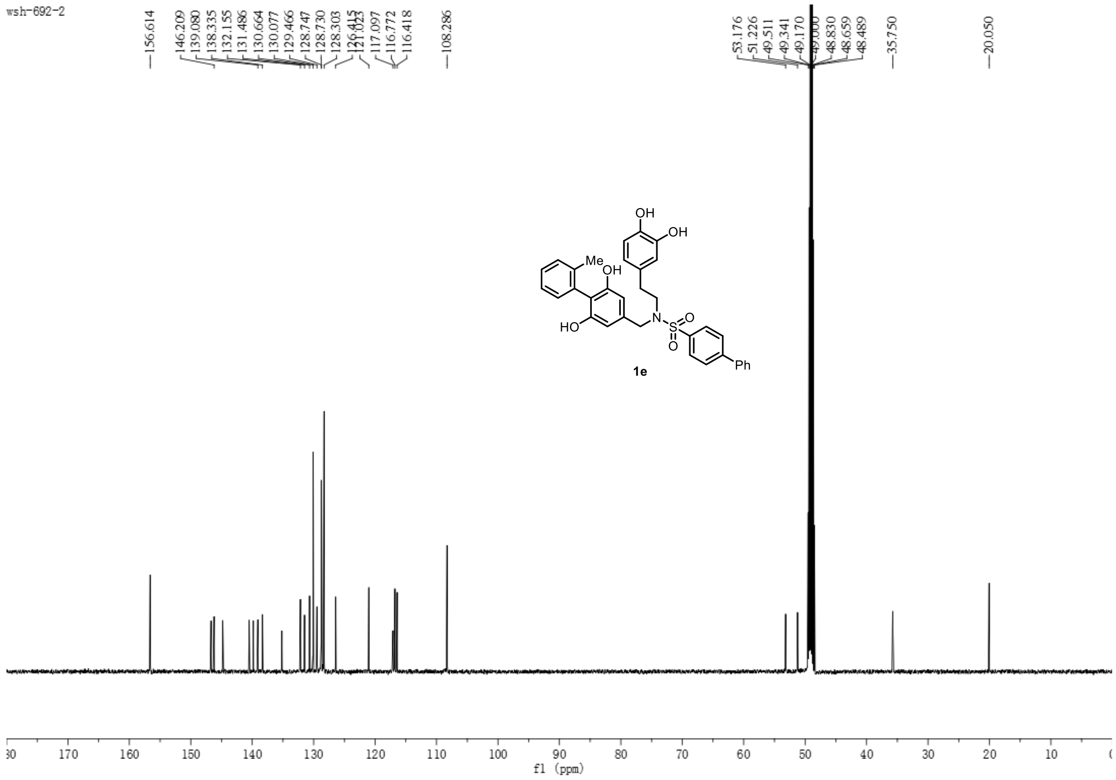

^{1}H NMR spectrum of compound 1f (500 MHz) in CD_3OD

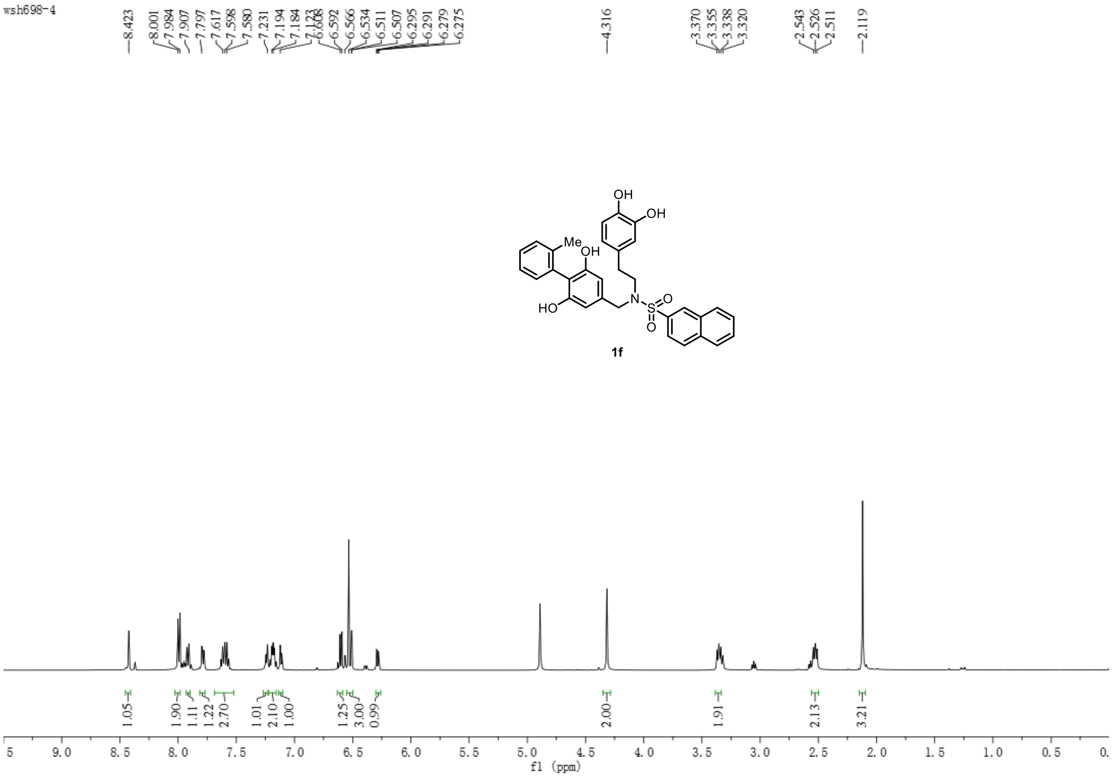

^{13}C NMR spectrum of compound 1f (125 MHz) in CD_3OD

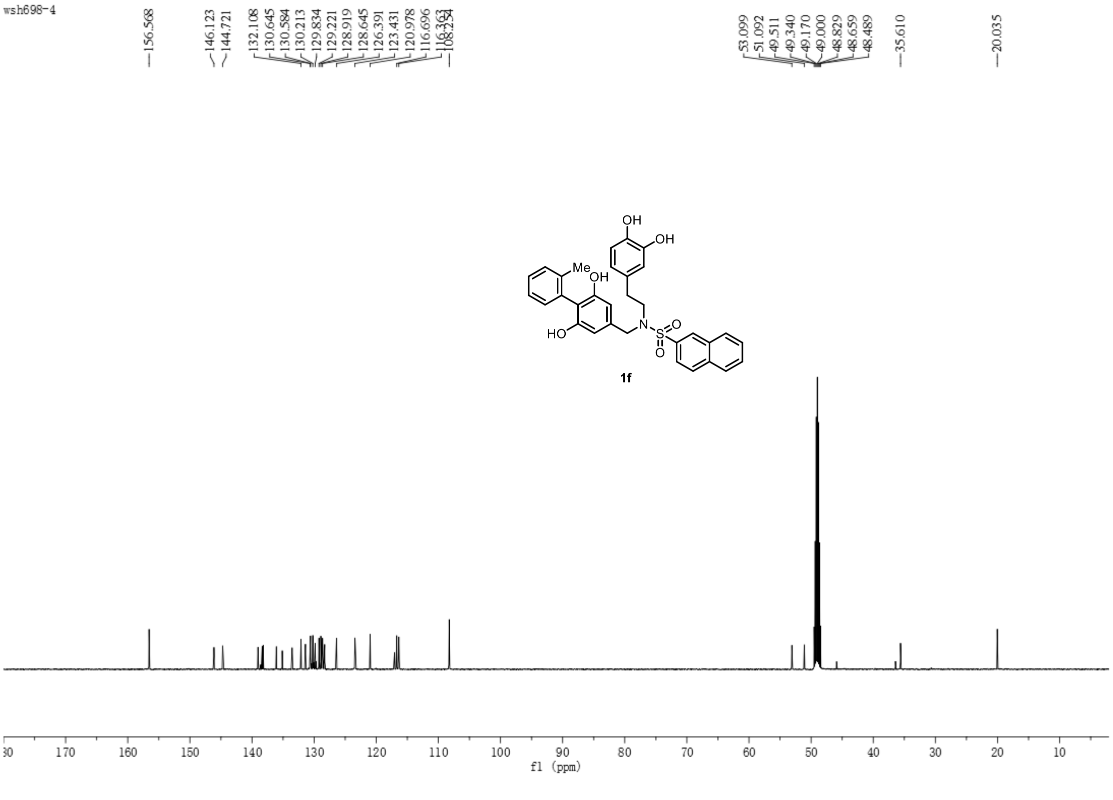

^{1}H NMR spectrum of compound 1g (500 MHz) in Acetone-d_6

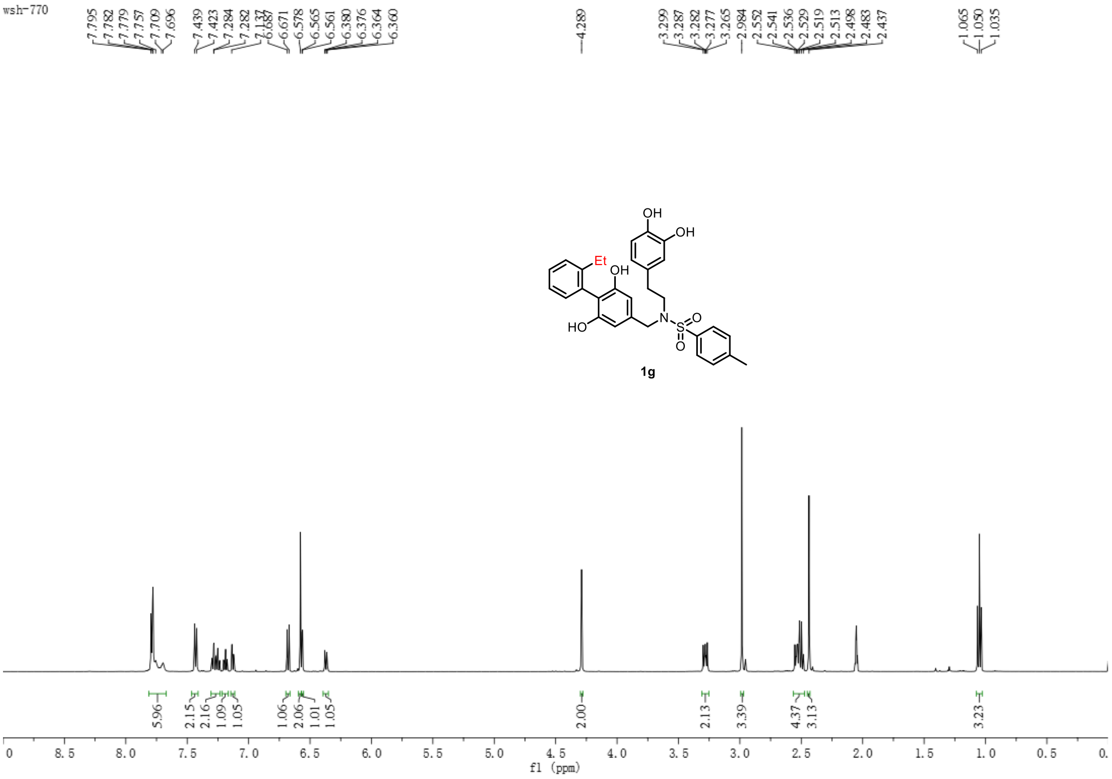

^{13}C NMR spectrum of compound 1g (125 MHz) in Acetone-d_6

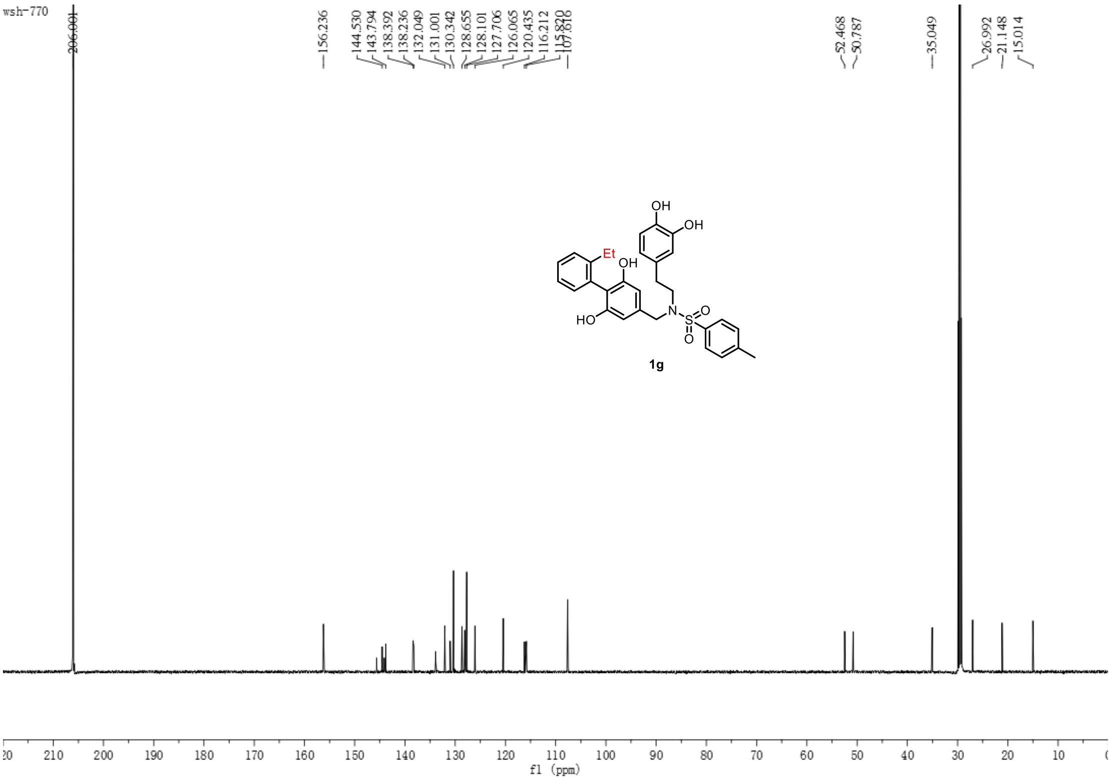

^{1}H NMR spectrum of compound 1h (500 MHz) in Acetone-d_6

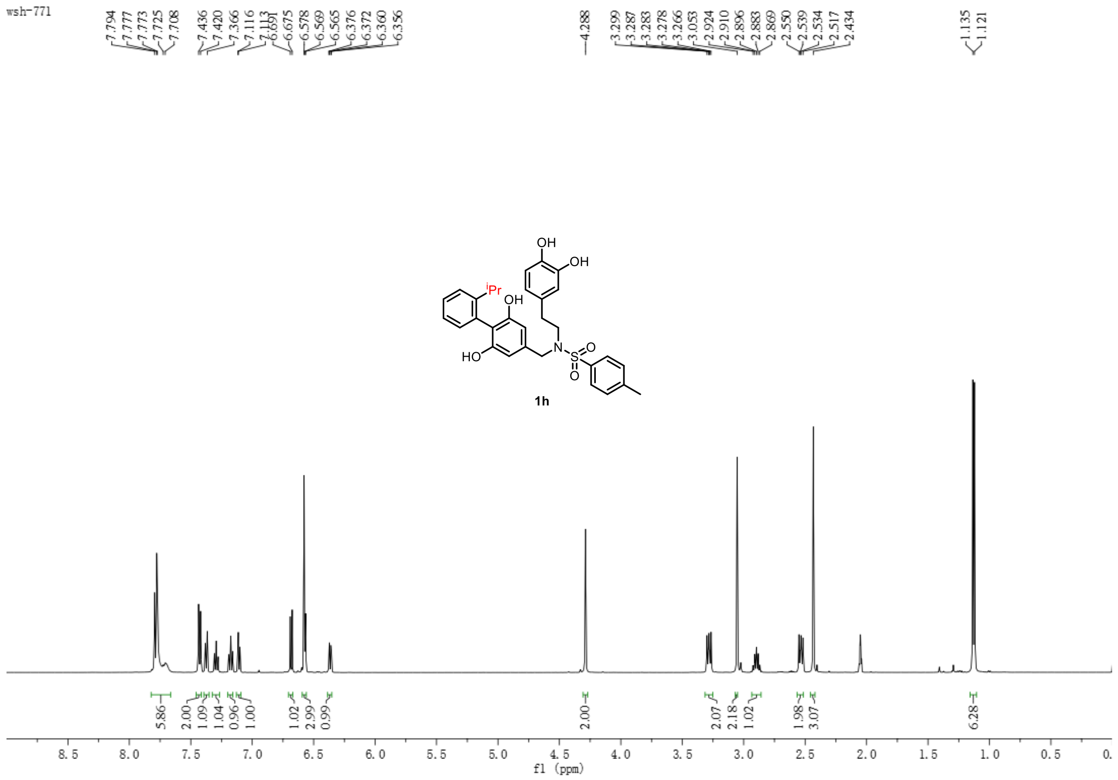

^{13}C NMR spectrum of compound 1h (125 MHz) in Acetone-d_6

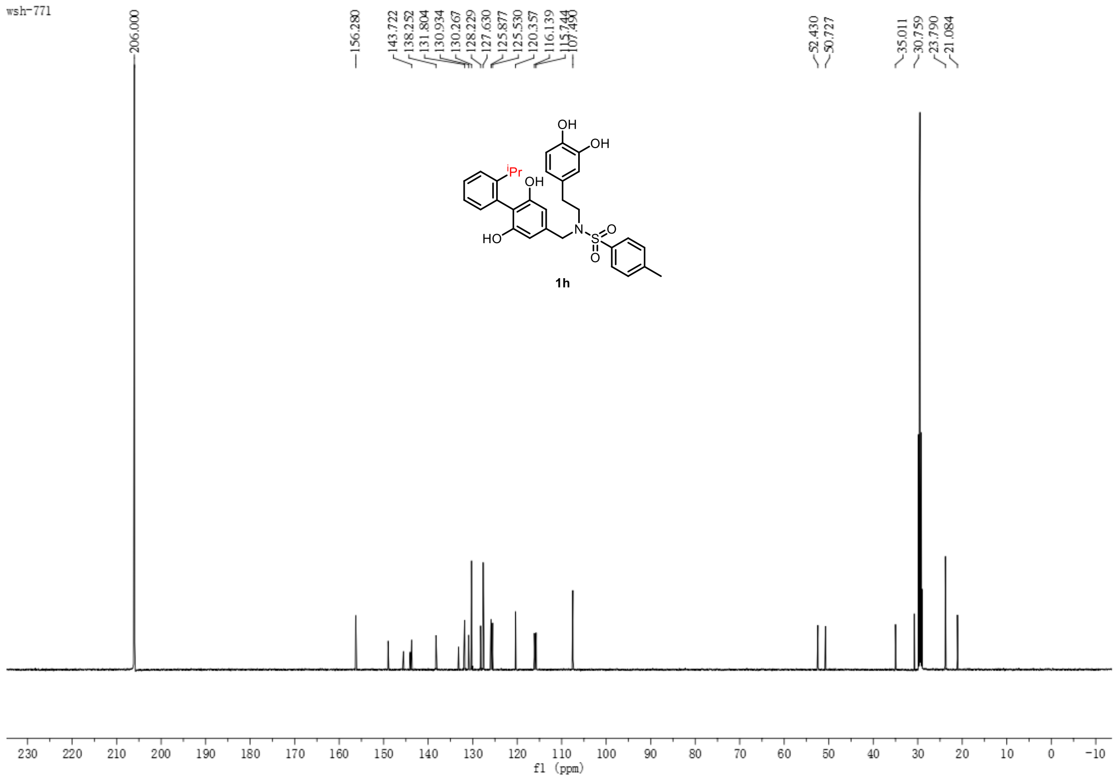

^{1}H NMR spectrum of compound 1i (500 MHz) in CD_3OD

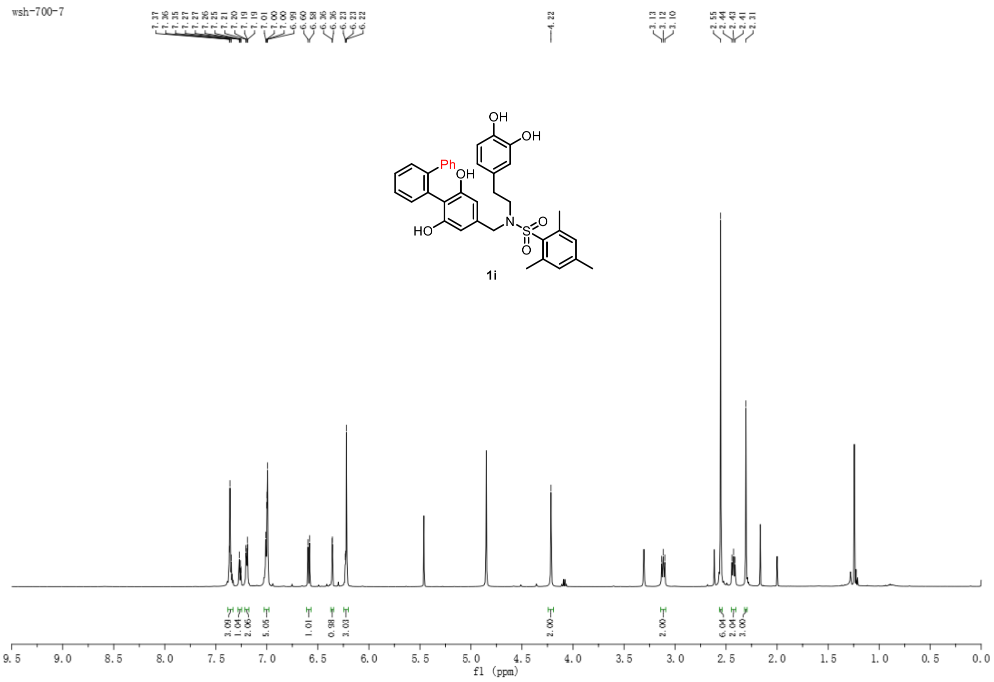

^{13}C NMR spectrum of compound 3aa (125 MHz) in CD_3OD

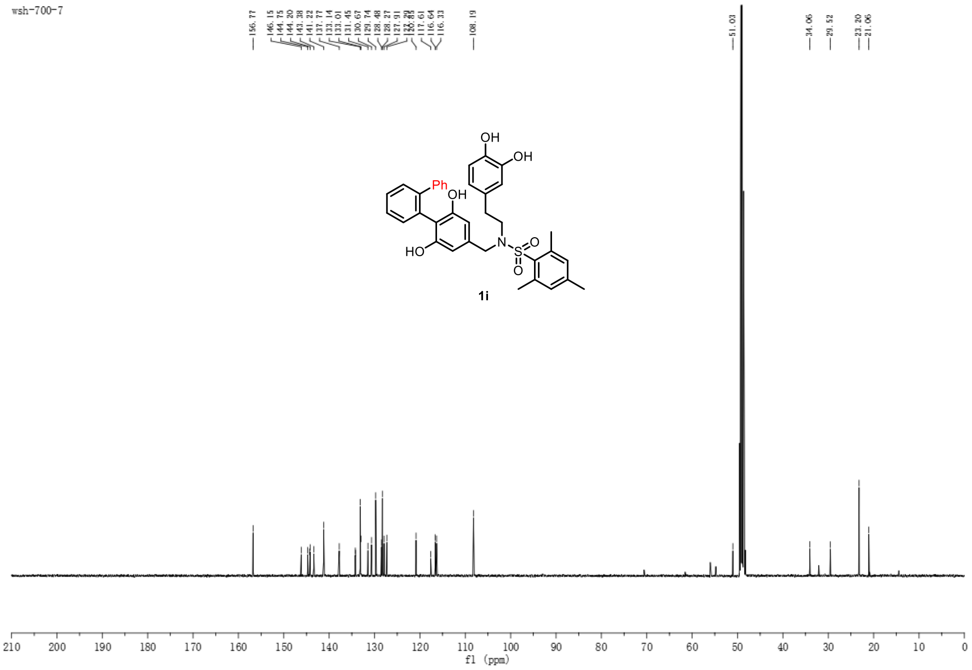

^{1}H NMR spectrum of compound 1j (500 MHz) in Acetone-d_6

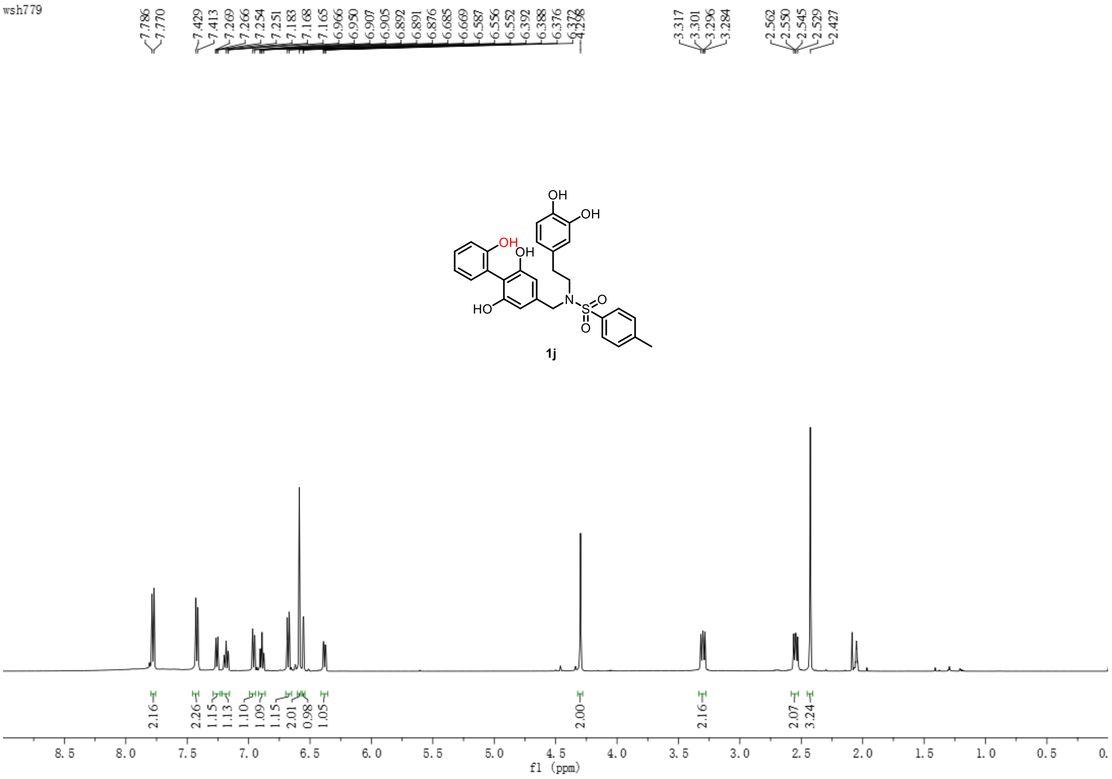

^{13}C NMR spectrum of compound 1j (125 MHz) inAcetone-d_6

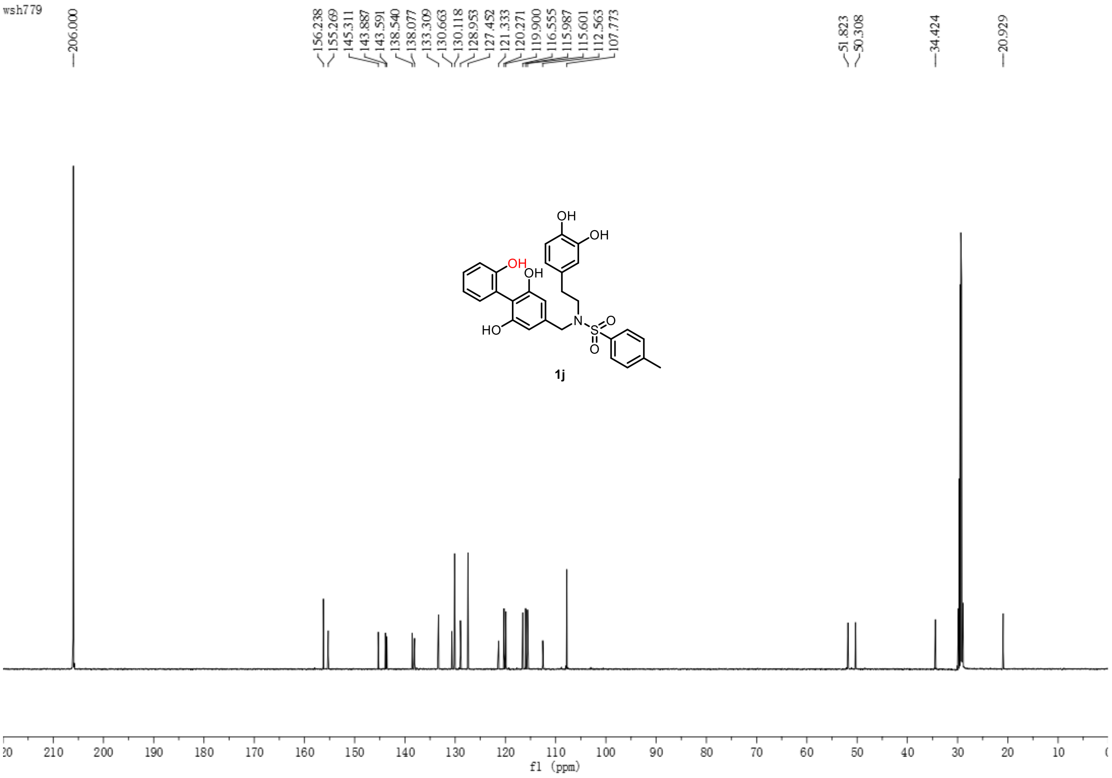

^{1}H NMR spectrum of compound 1k (500 MHz) in Acetone-d_6

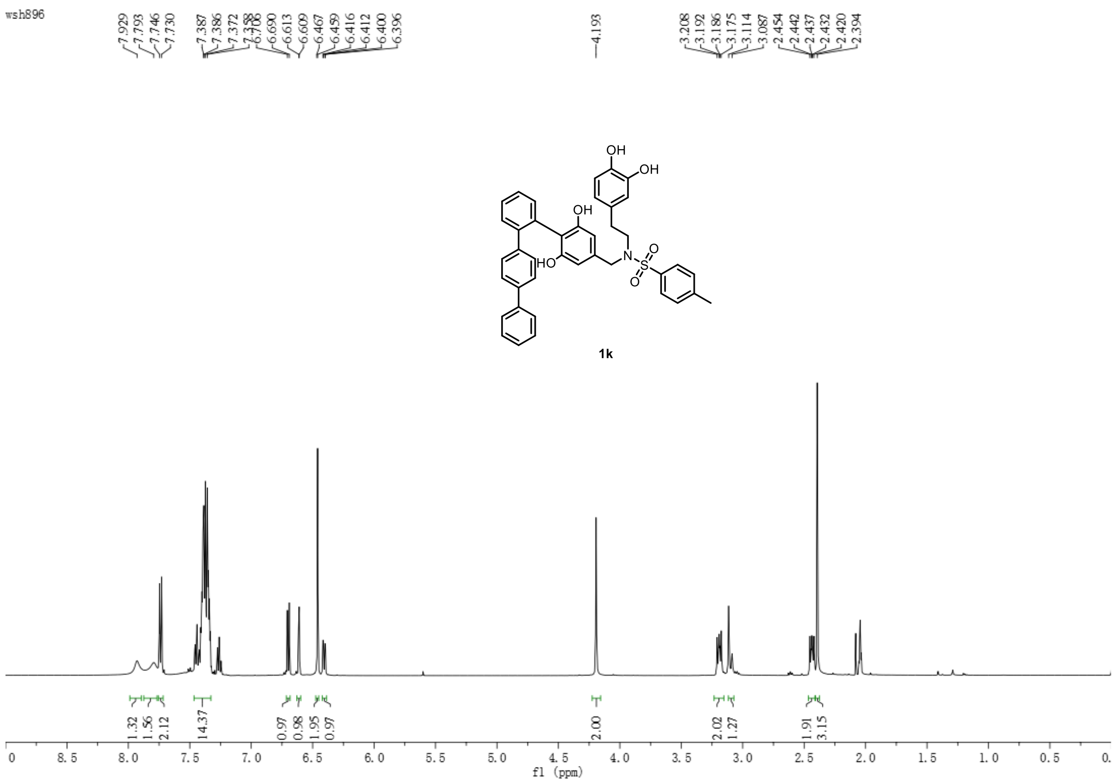

^{13}C NMR spectrum of compound 1k (125 MHz) in Acetone-d_6

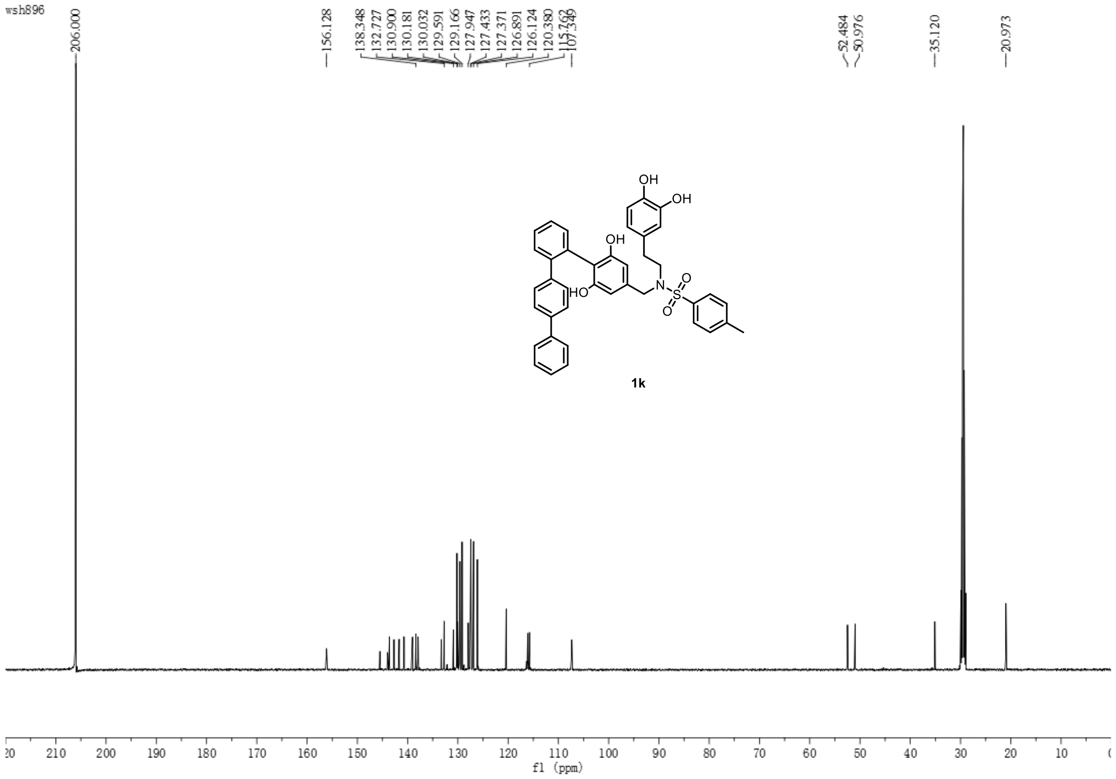

^{1}H NMR spectrum of compound 1l (500 MHz) in Acetone-d_6

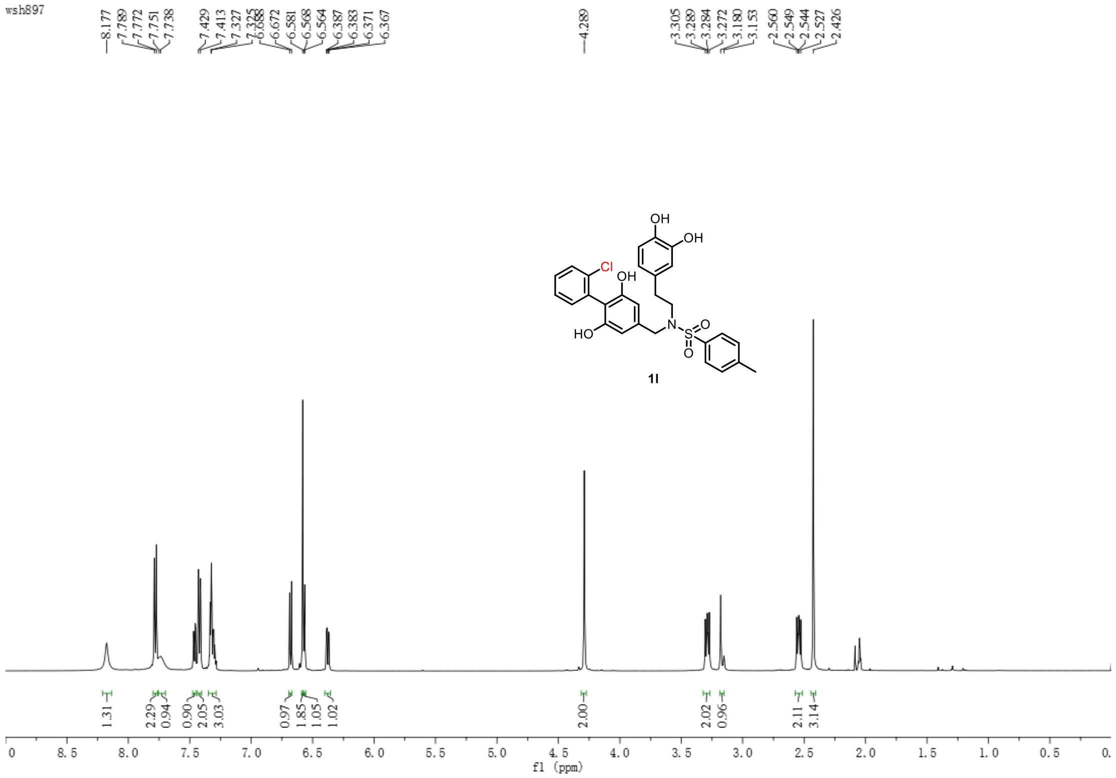

^{13}C NMR spectrum of compound 1l (125 MHz) in Acetone-d_6

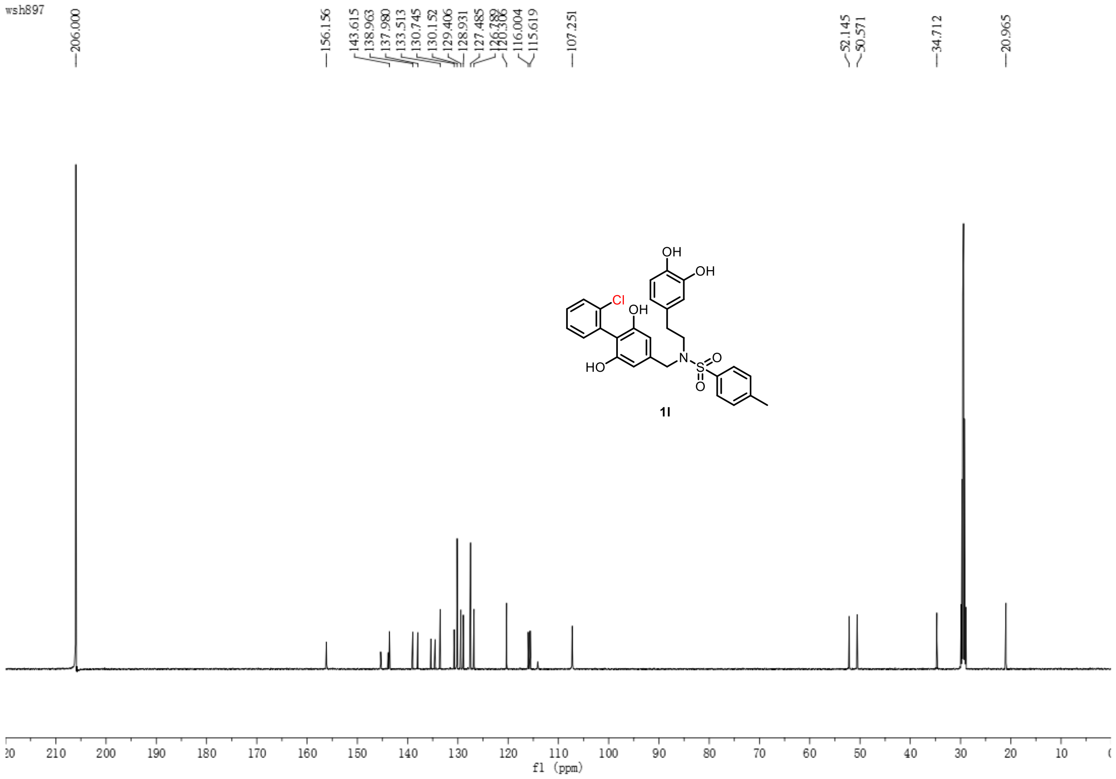

^{1}H NMR spectrum of compound 1m (500 MHz) in Acetone-d_6

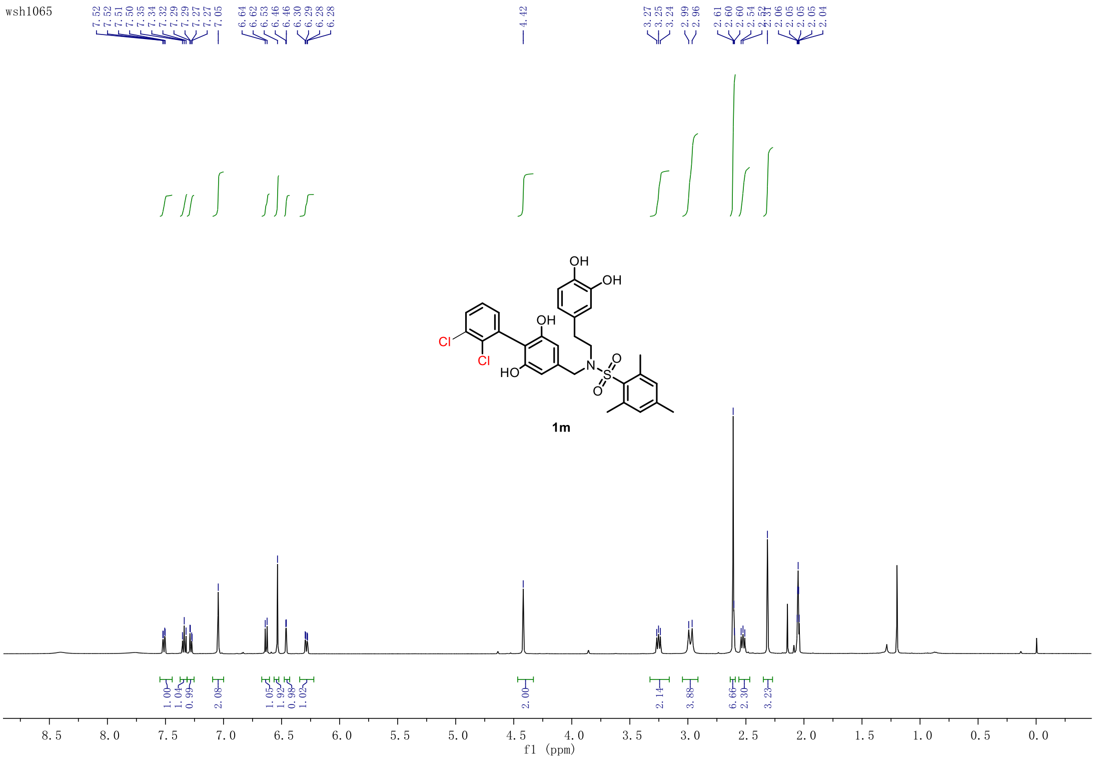

^{13}C NMR spectrum of compound 1m (125 MHz) in Acetone-d_6

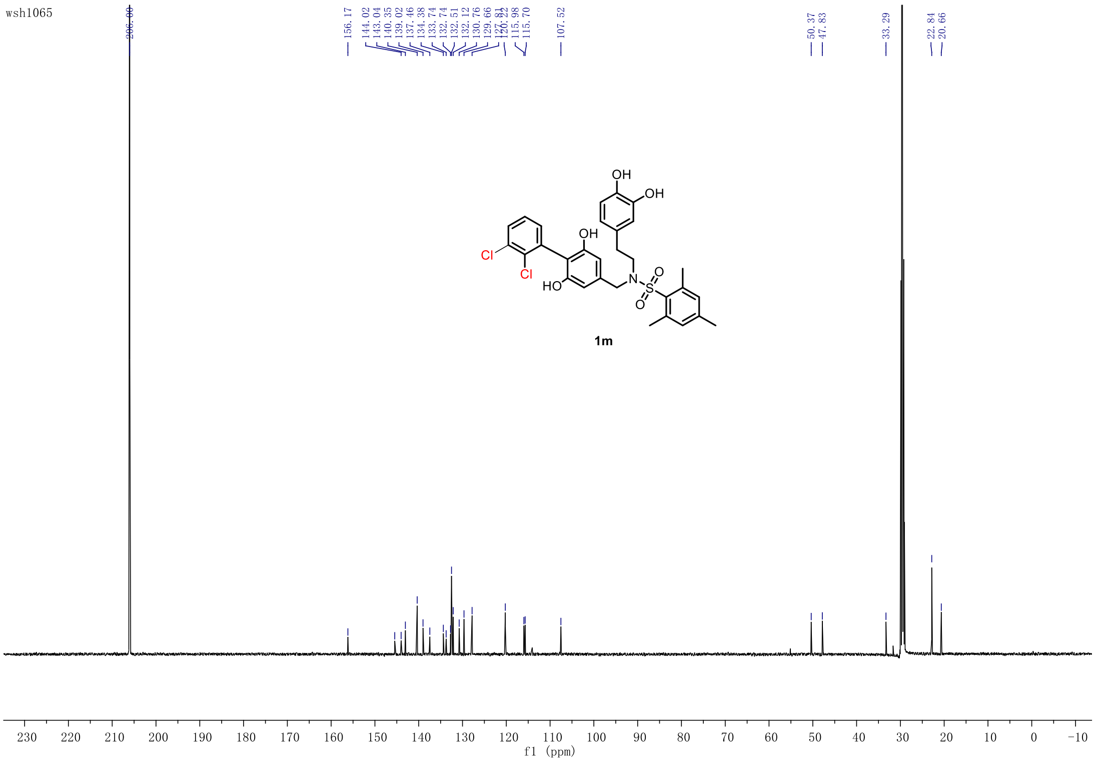

^{1}H NMR spectrum of compound 1n (500 MHz) in Acetone-d_6

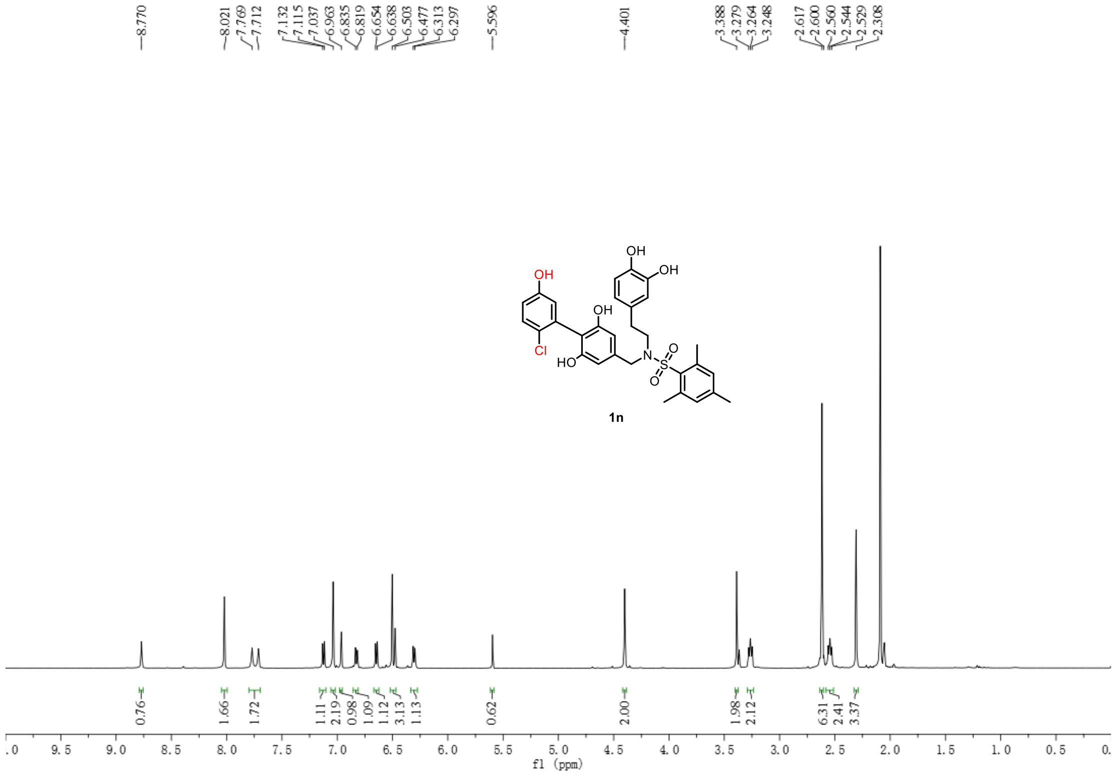

^{13}C NMR spectrum of compound 1n (125 MHz) in Acetone-d_6

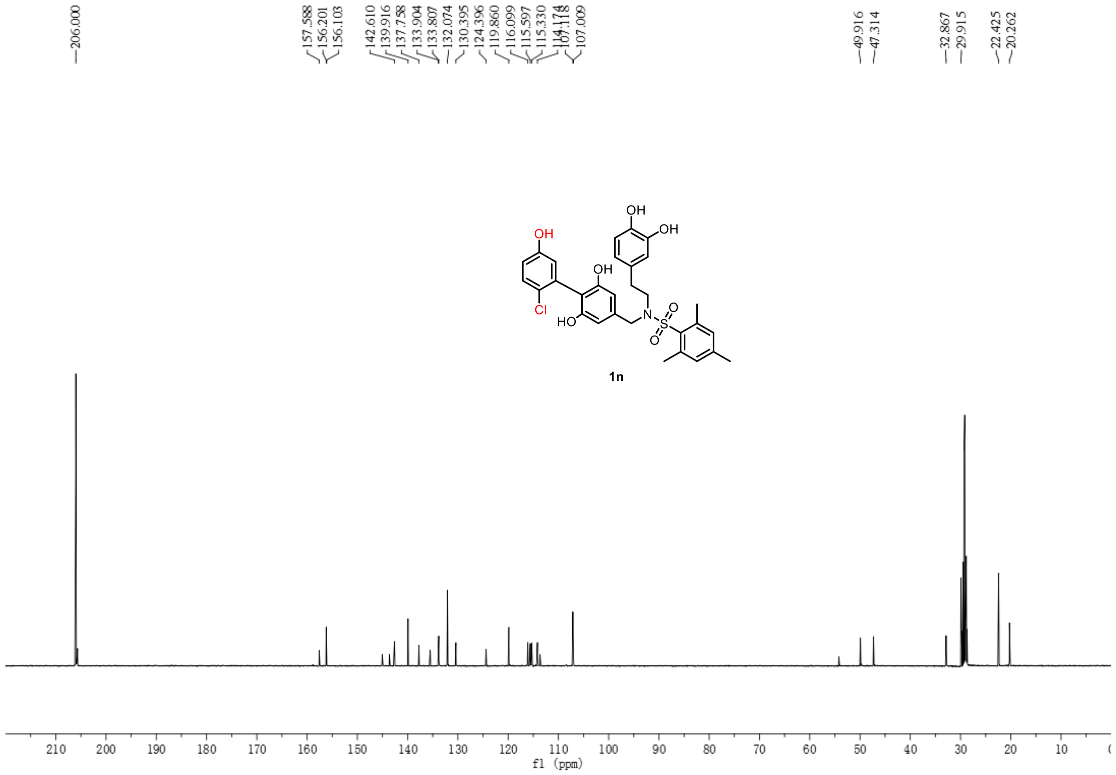

^{1}H NMR spectrum of compound 1o (500 MHz) in Acetone-d_6

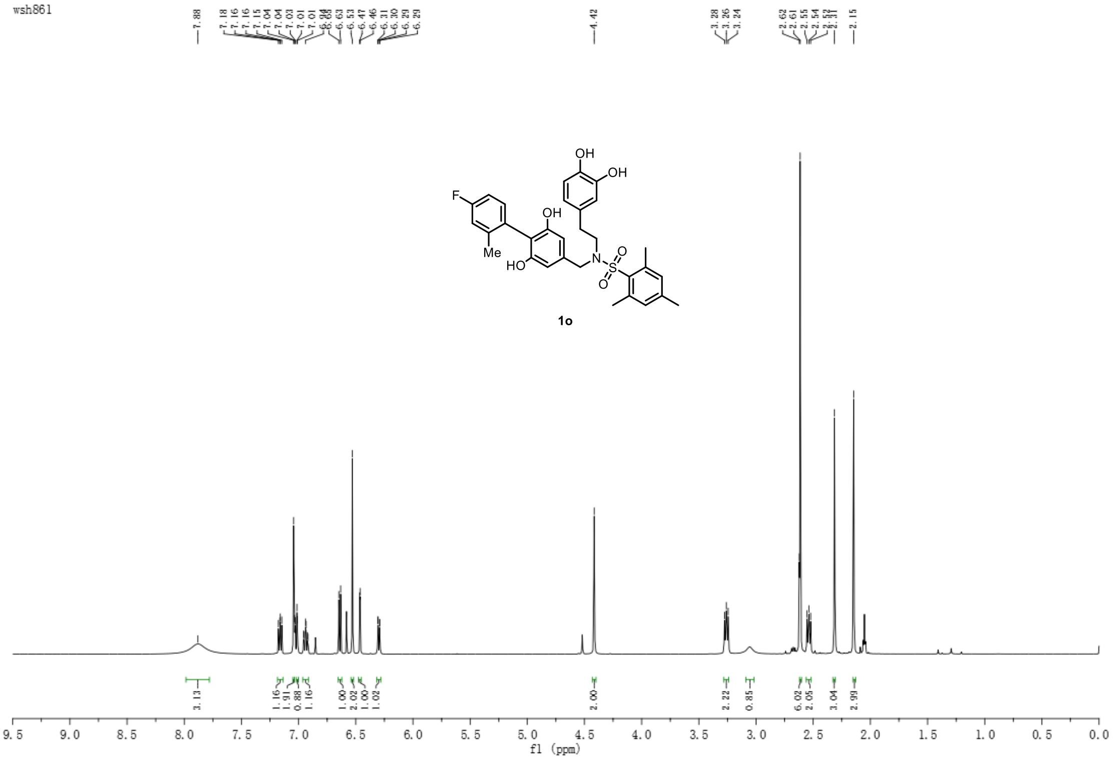

^{13}C NMR spectrum of compound 1o (125 MHz) in Acetone-d_6

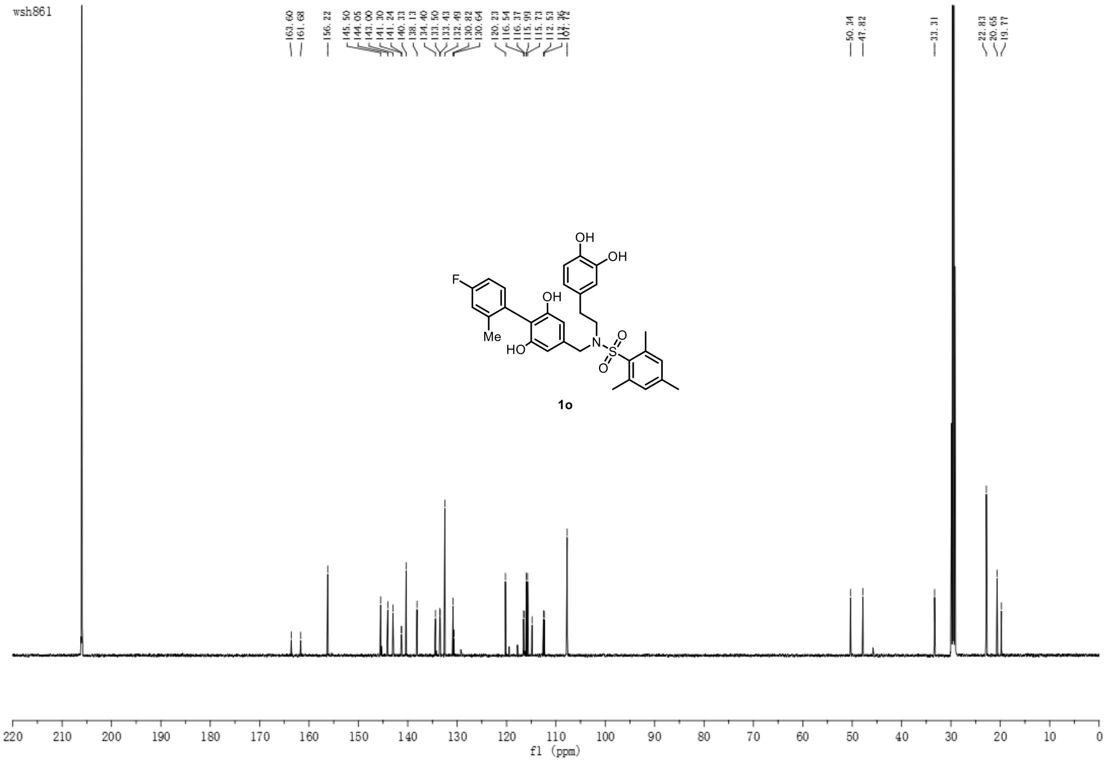

^{19}F NMR spectrum of compound 1o (470 MHz) in CD_3OD

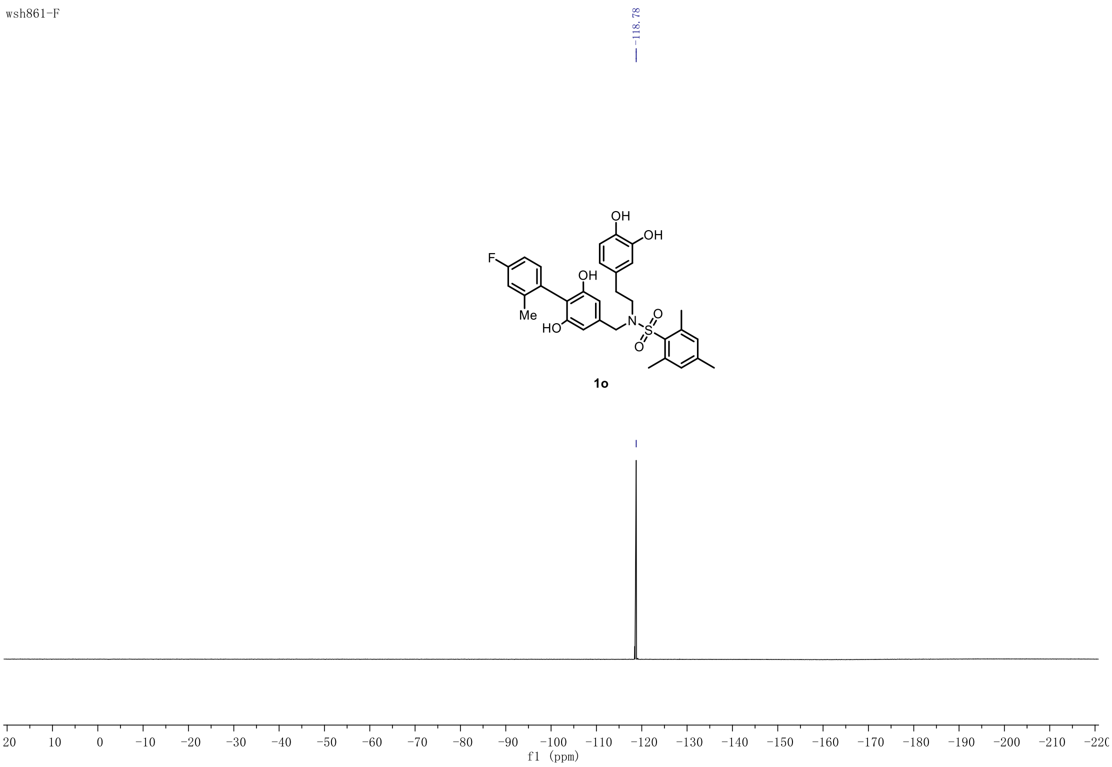

^{1}H NMR spectrum of compound 1p (500 MHz) in Acetone-d_6

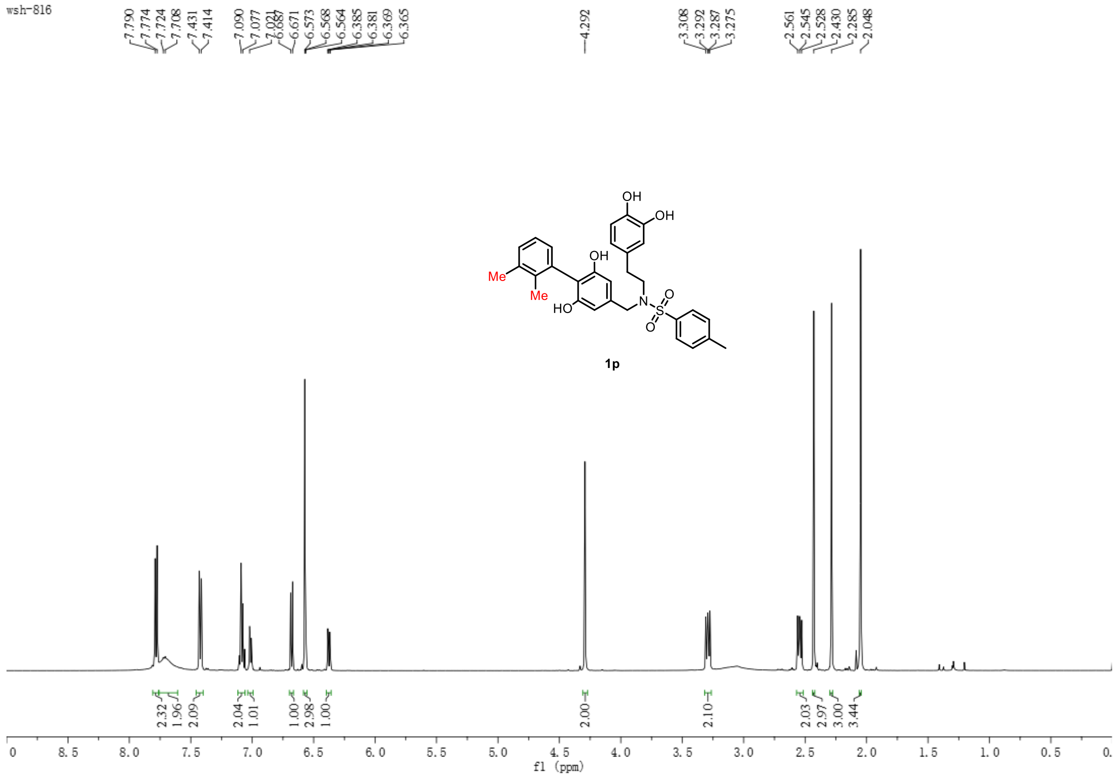

^{13}C NMR spectrum of compound 1p (125 MHz) in Acetone-d_6

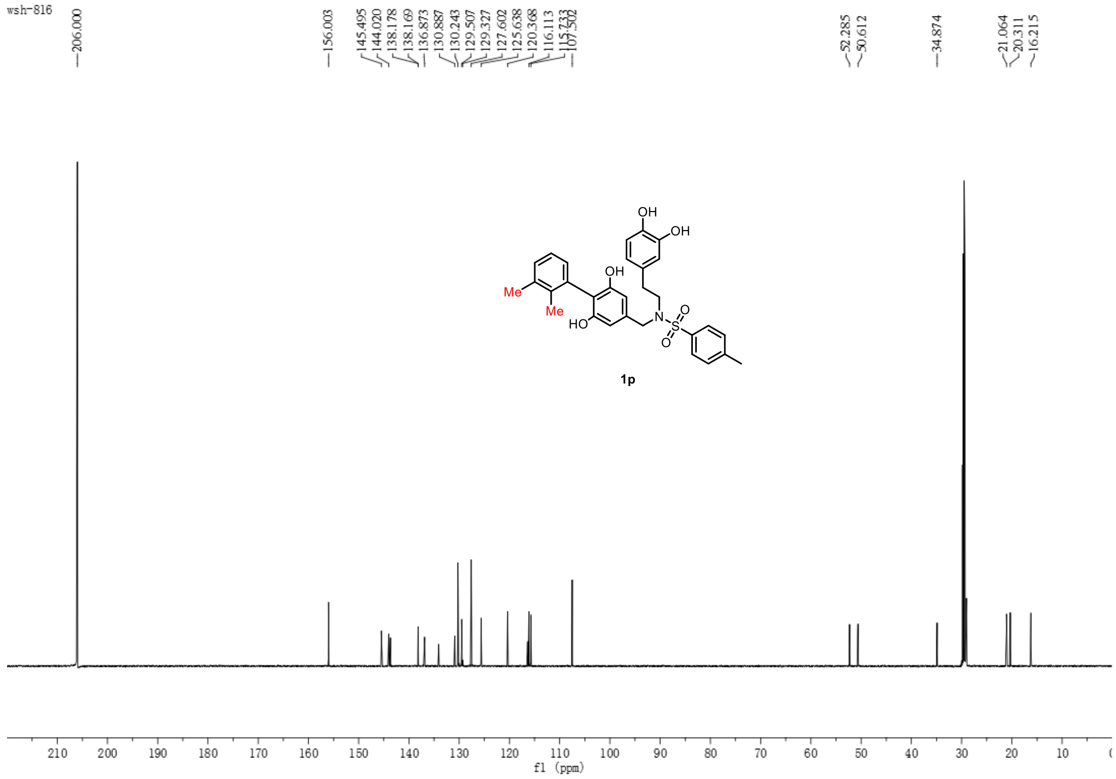

^{1}H NMR spectrum of compound 1q (500 MHz) in Acetone-d_6

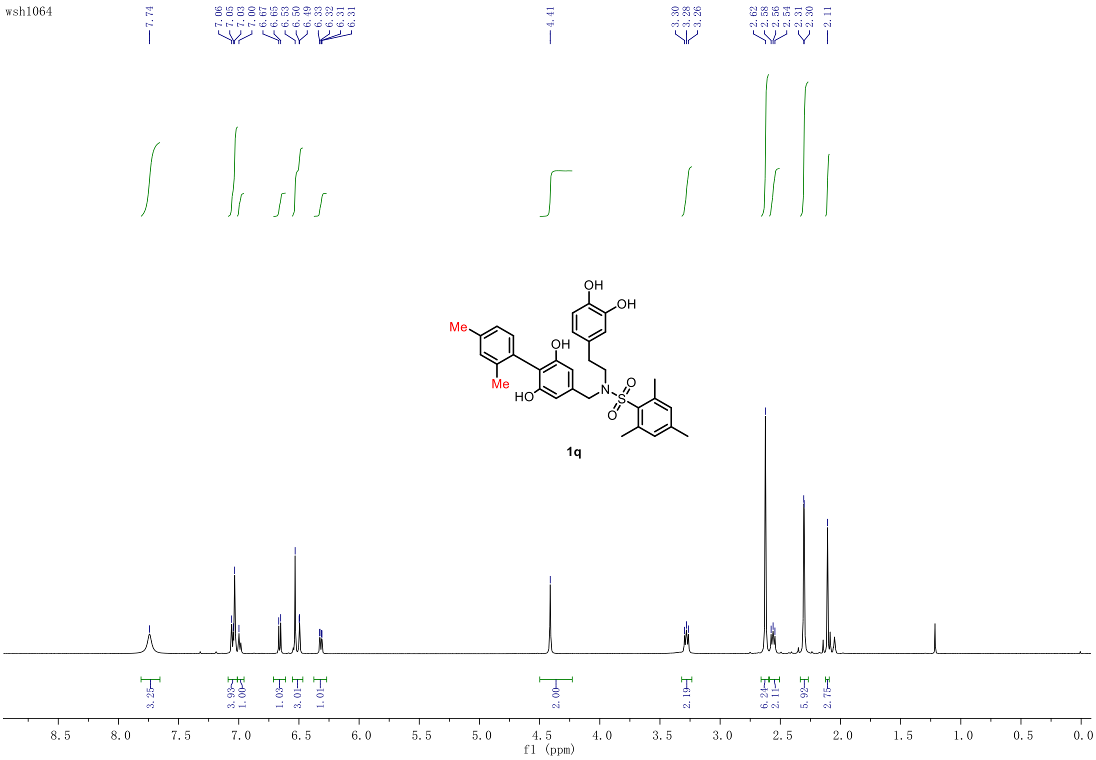

^{13}C NMR spectrum of compound 1q (125 MHz) in Acetone-d_6

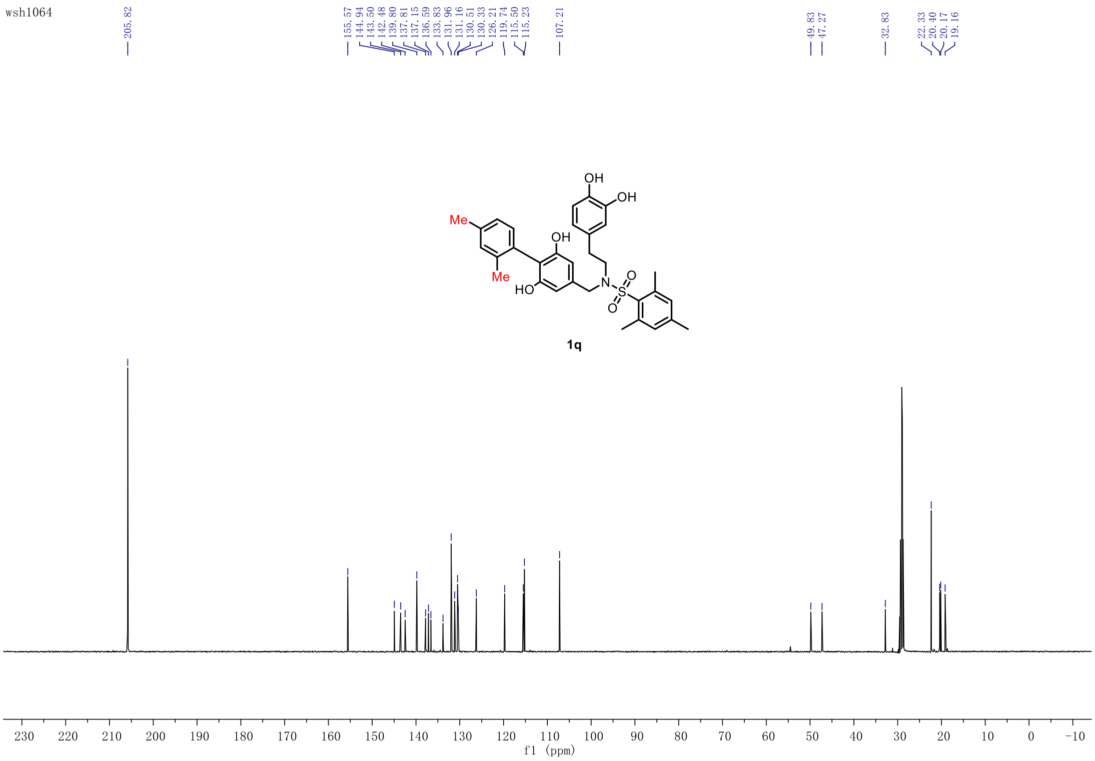

^{1}H NMR spectrum of compound 1r (500 MHz) in Acetone-d_6

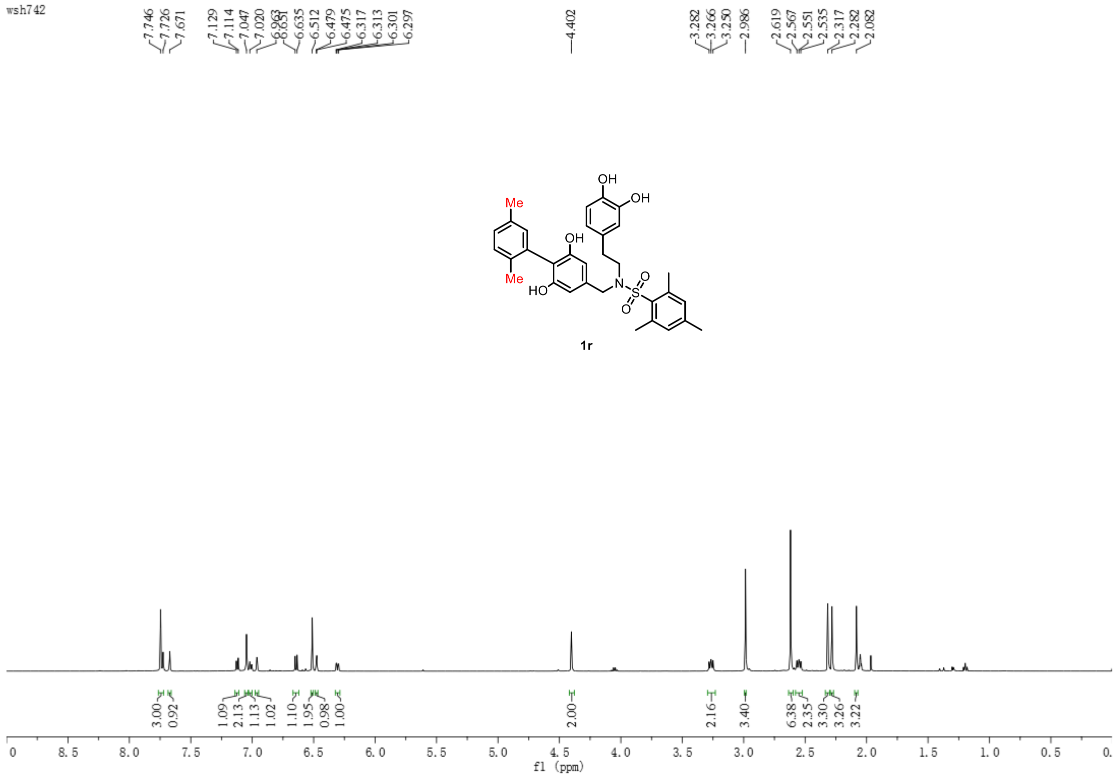

^{13}C NMR spectrum of compound 1r (125 MHz) in Acetone-d_6

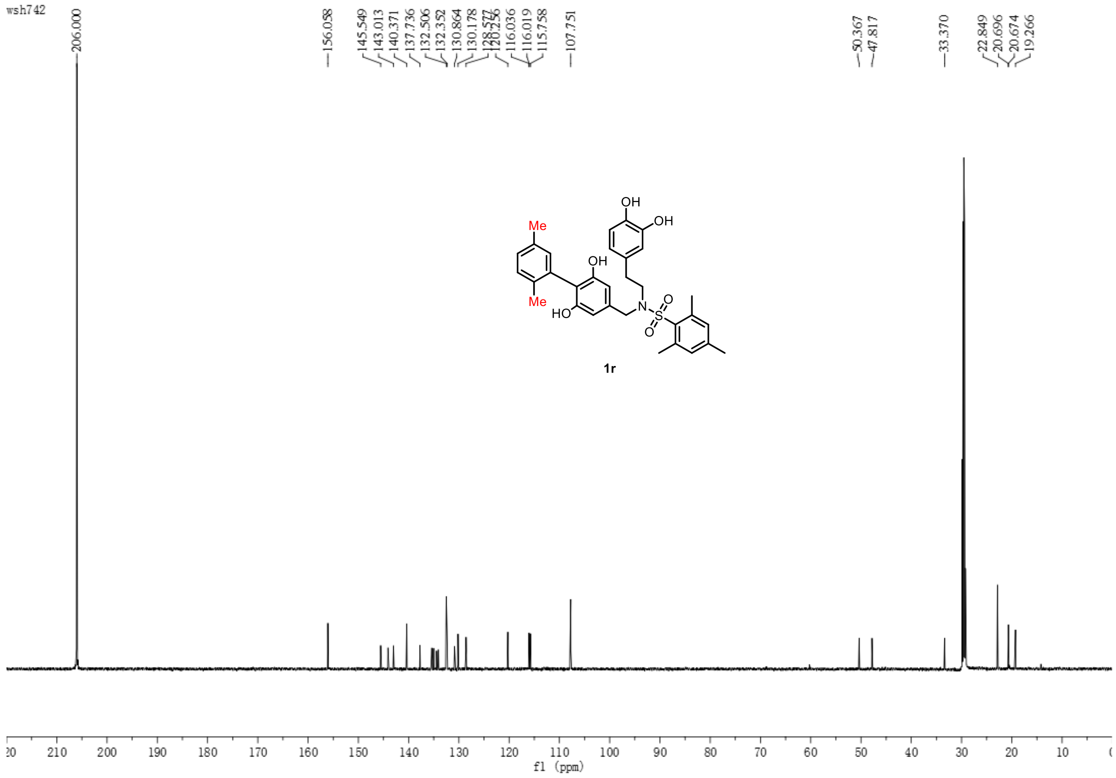

^{1}H NMR spectrum of compound 1s (500 MHz) in Acetone-d_6

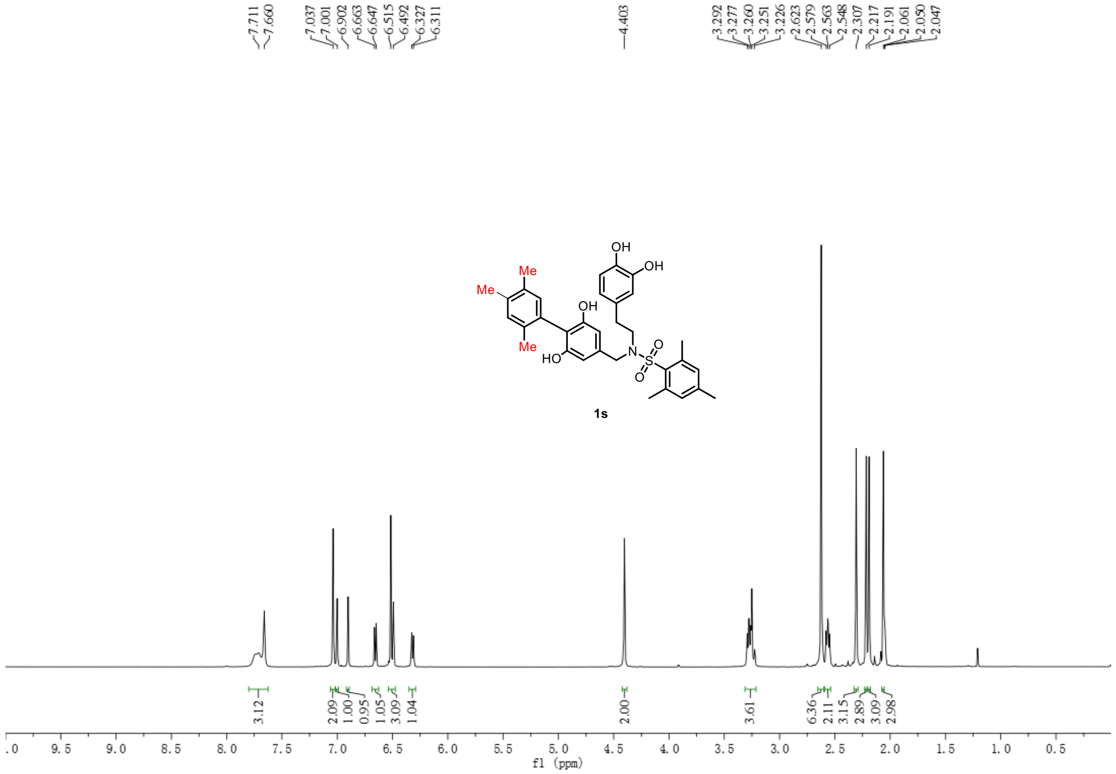

^{13}C NMR spectrum of compound 1s (125 MHz) in Acetone-d_6

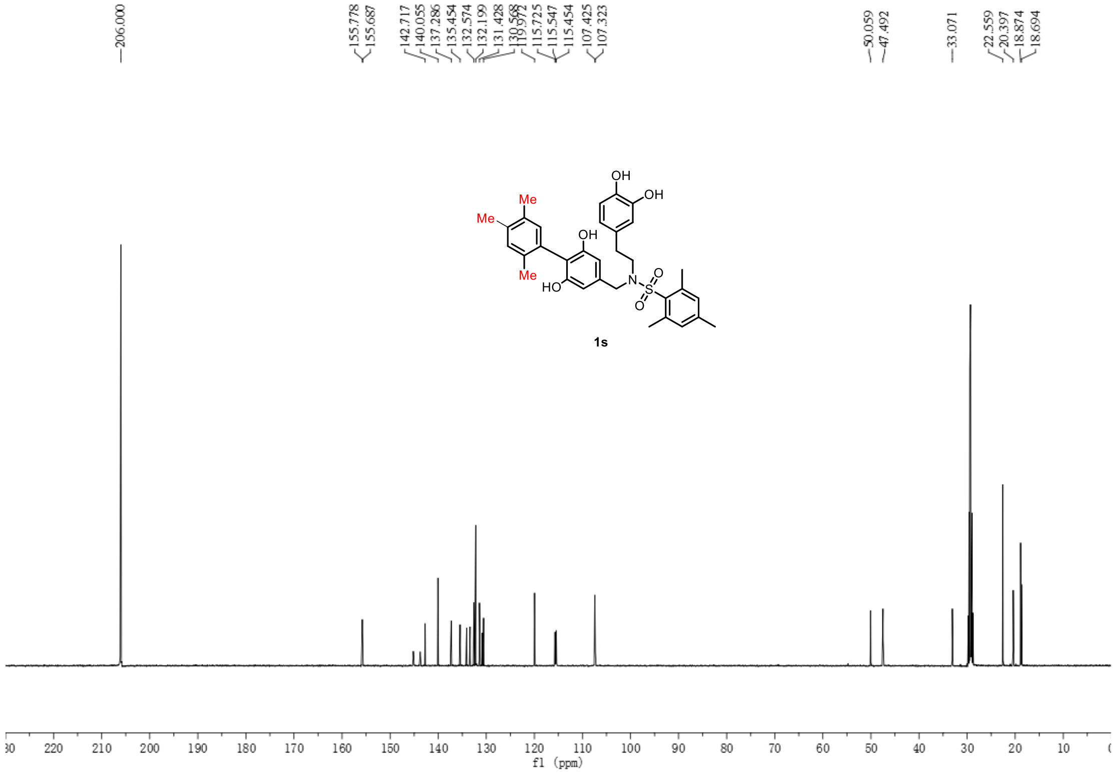

^{1}H NMR spectrum of compound 1t (500 MHz) in Acetone-d_6

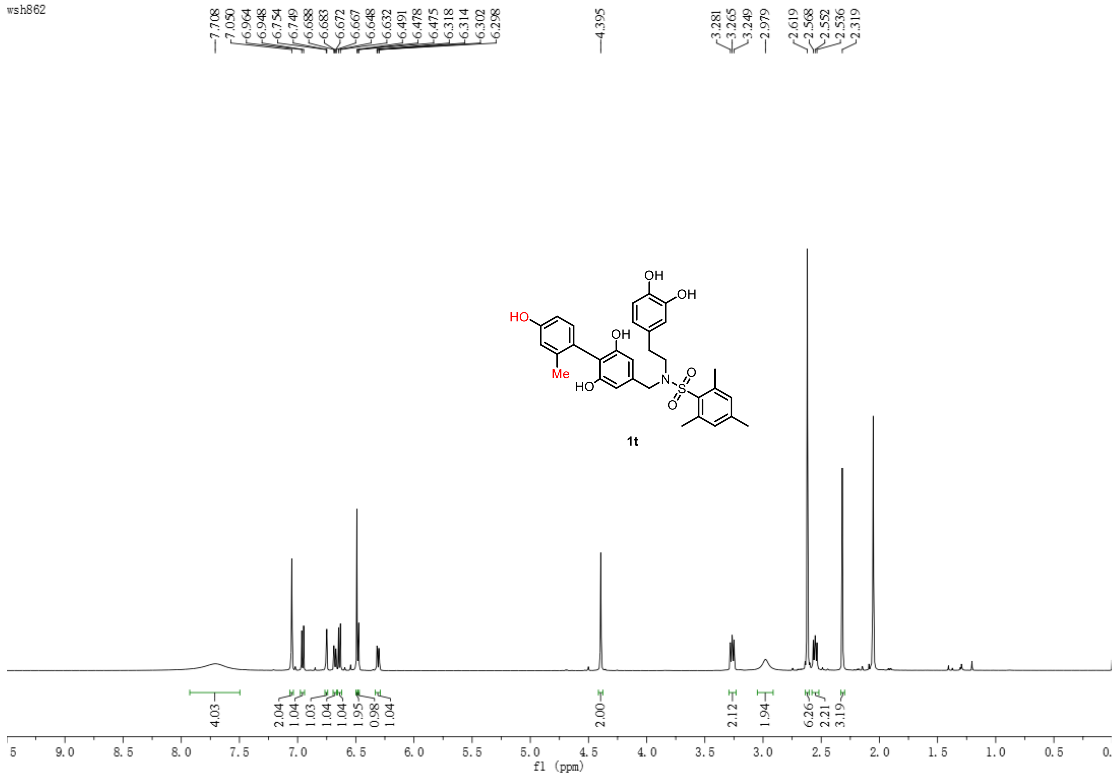

^{13}C NMR spectrum of compound 1t (125 MHz) in Acetone-d_6

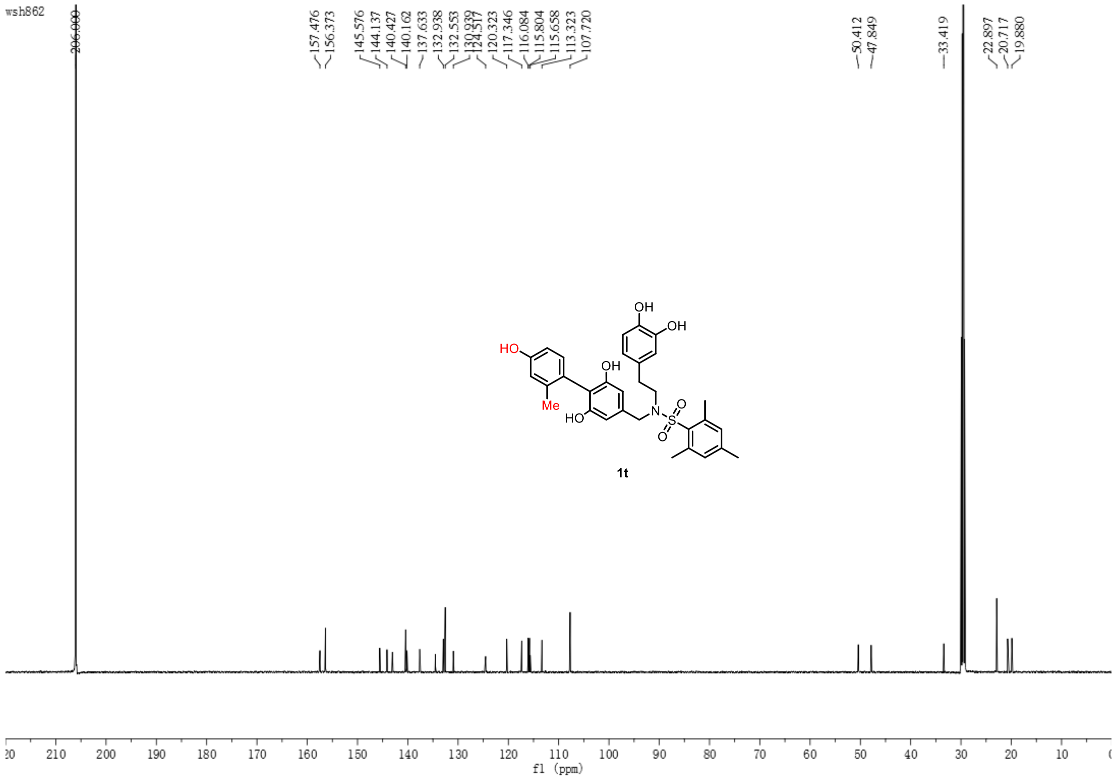

^{1}H NMR spectrum of compound 1u (500 MHz) in Acetone-d_6

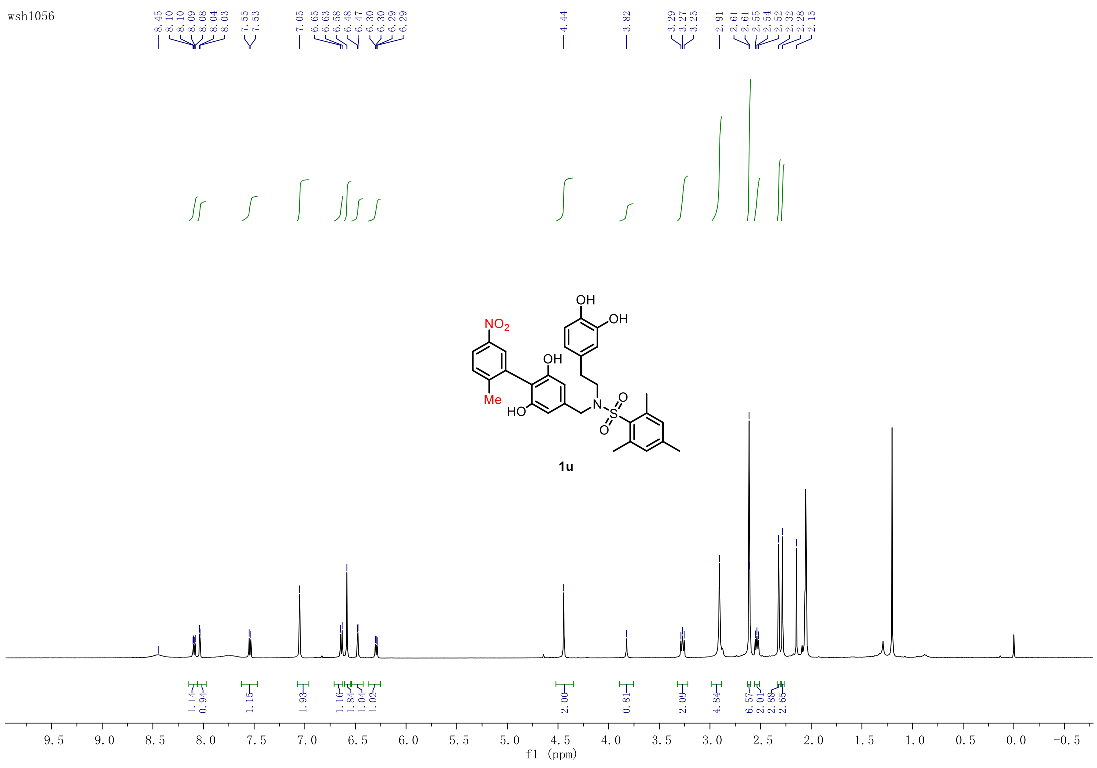

^{13}C NMR spectrum of compound 1u (125 MHz) in Acetone-d_6

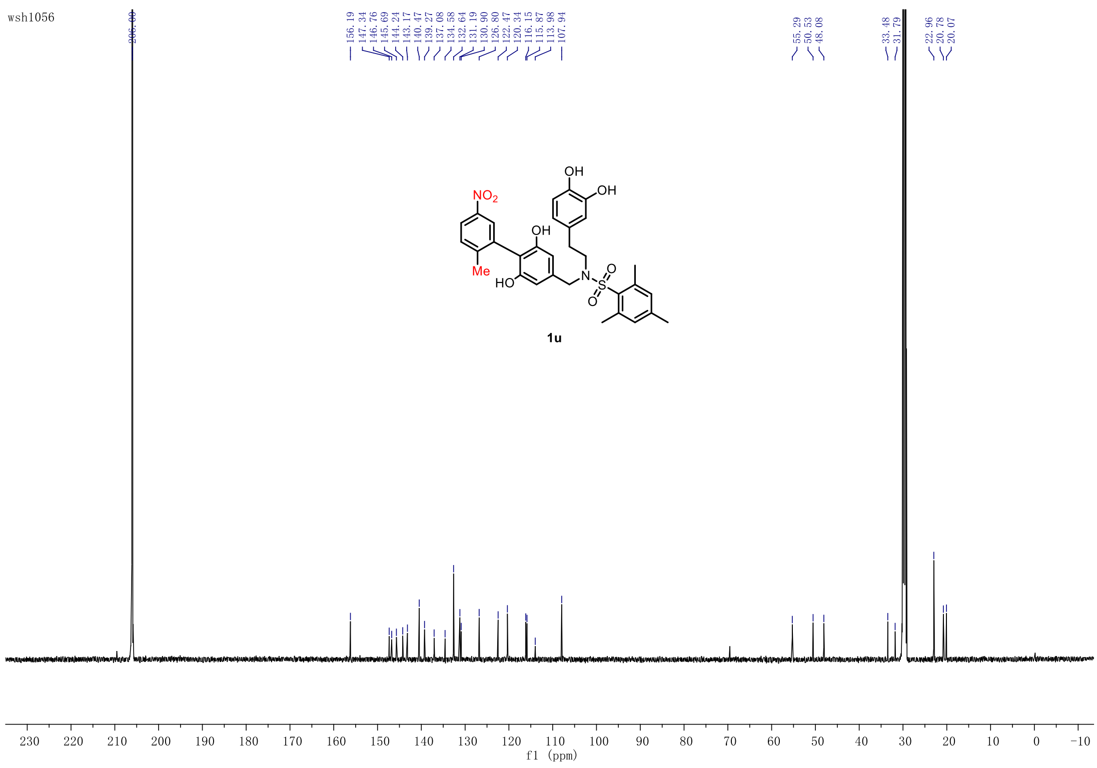

^{1}H NMR spectrum of compound 1v (500 MHz) in Acetone-d_6

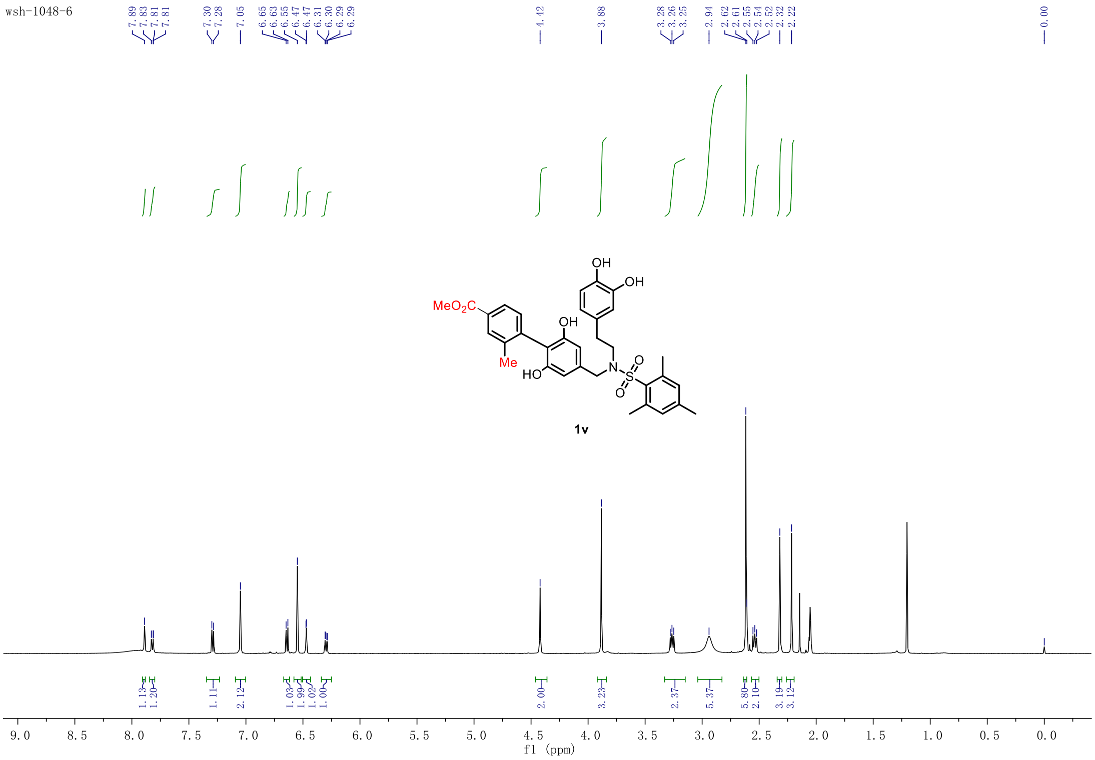

^{13}C NMR spectrum of compound 1v (125 MHz) in Acetone-d_6

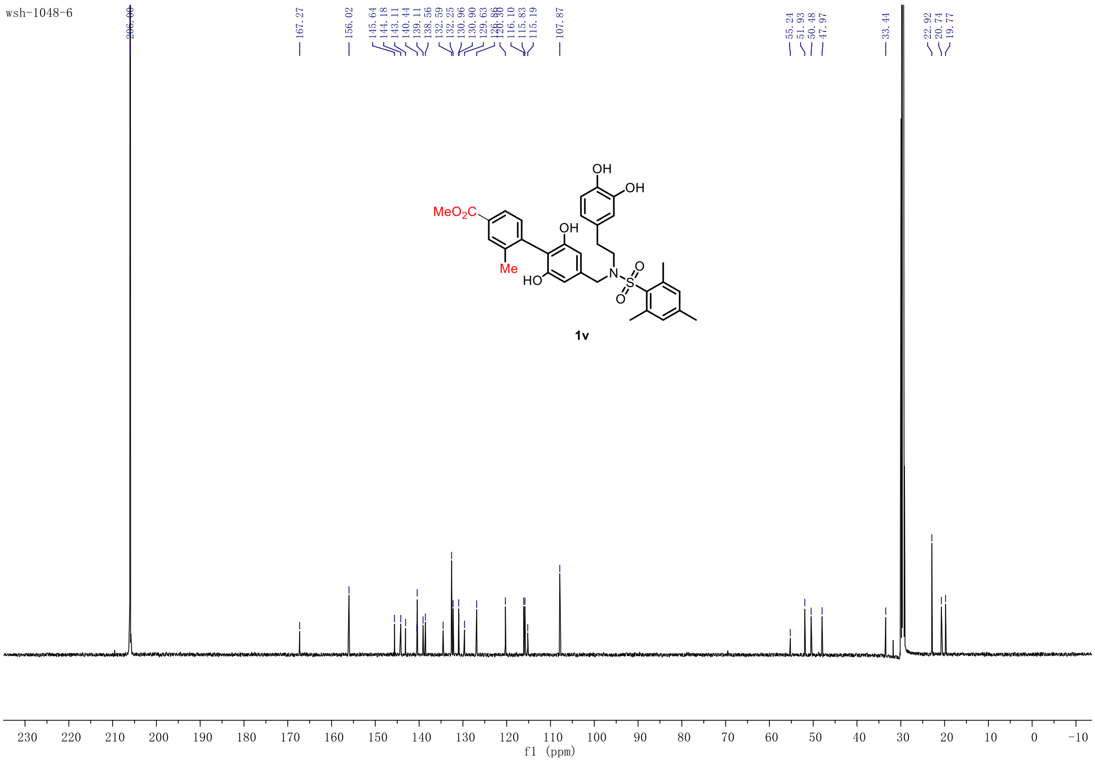

^{1}H NMR spectrum of compound 1w (500 MHz) in Acetone-d_6

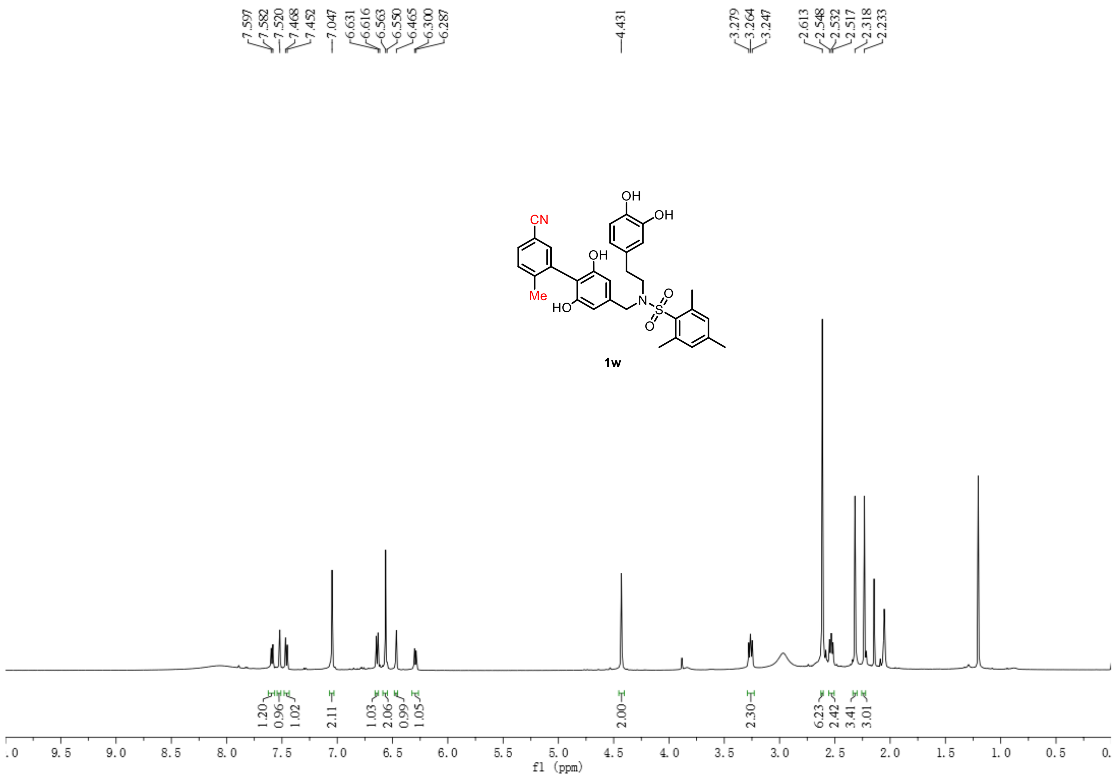

^{13}C NMR spectrum of compound 1w (125 MHz) in Acetone-d_6

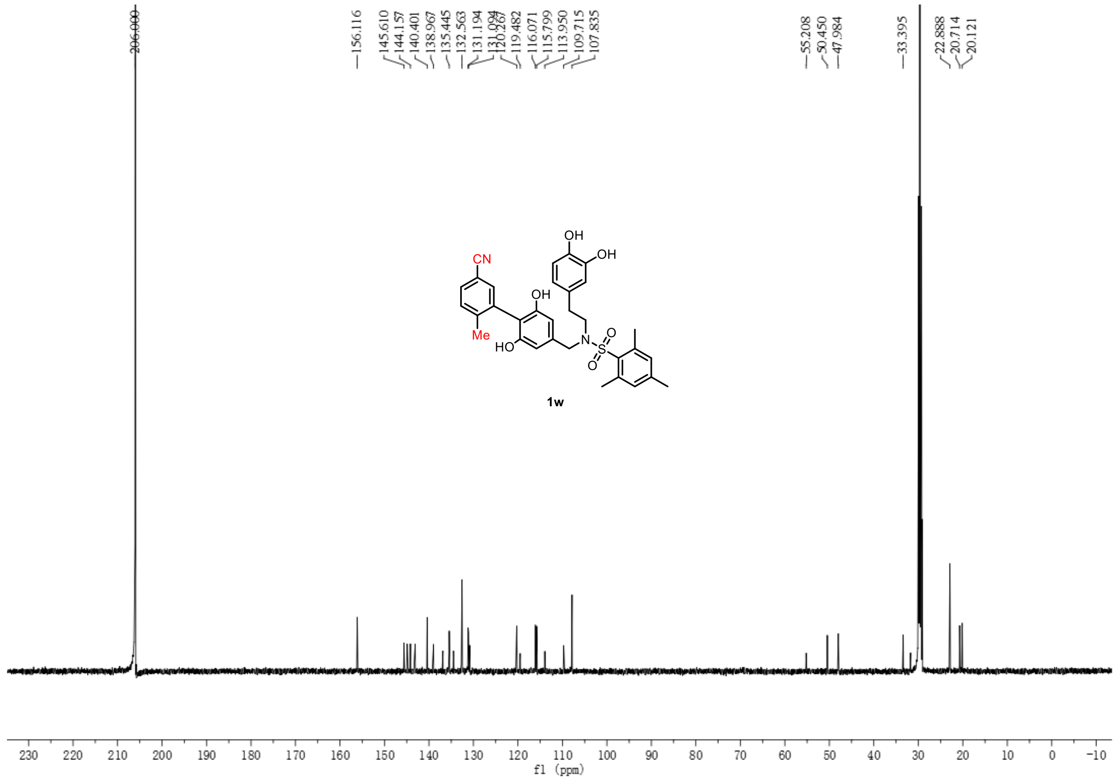

^{1}H NMR spectrum of compound 1x (500 MHz) in CD_3OD

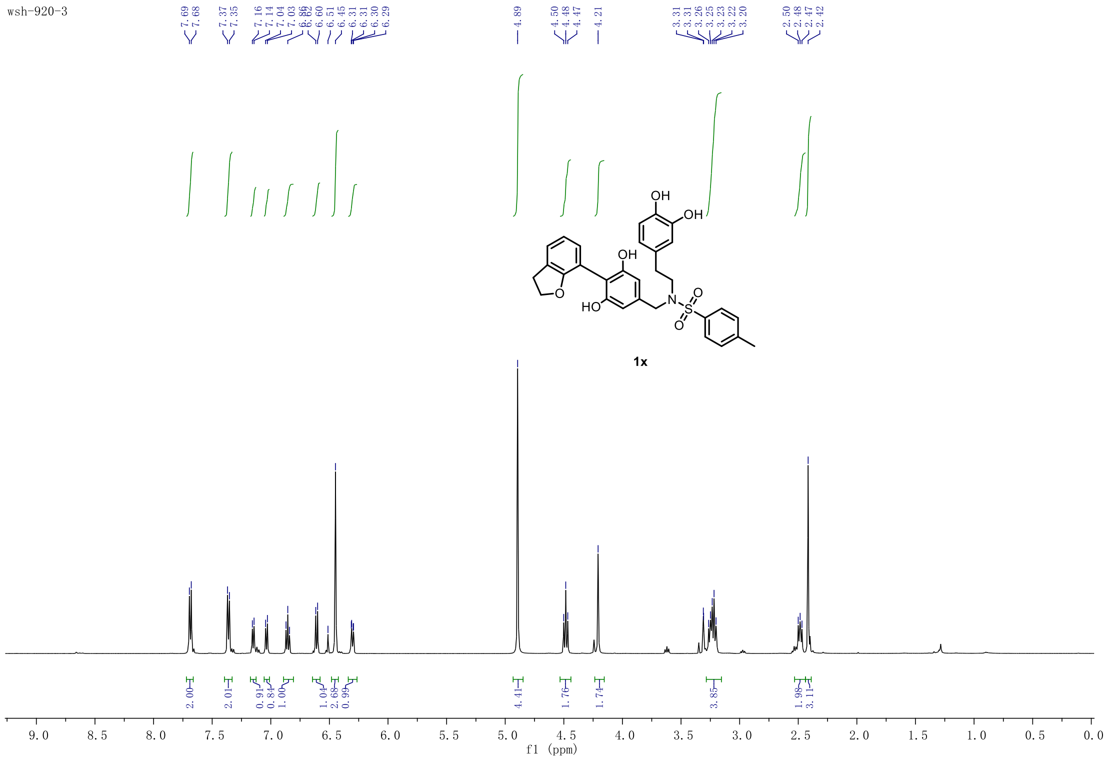

^{13}C NMR spectrum of compound 1x (125 MHz) in CD_3OD

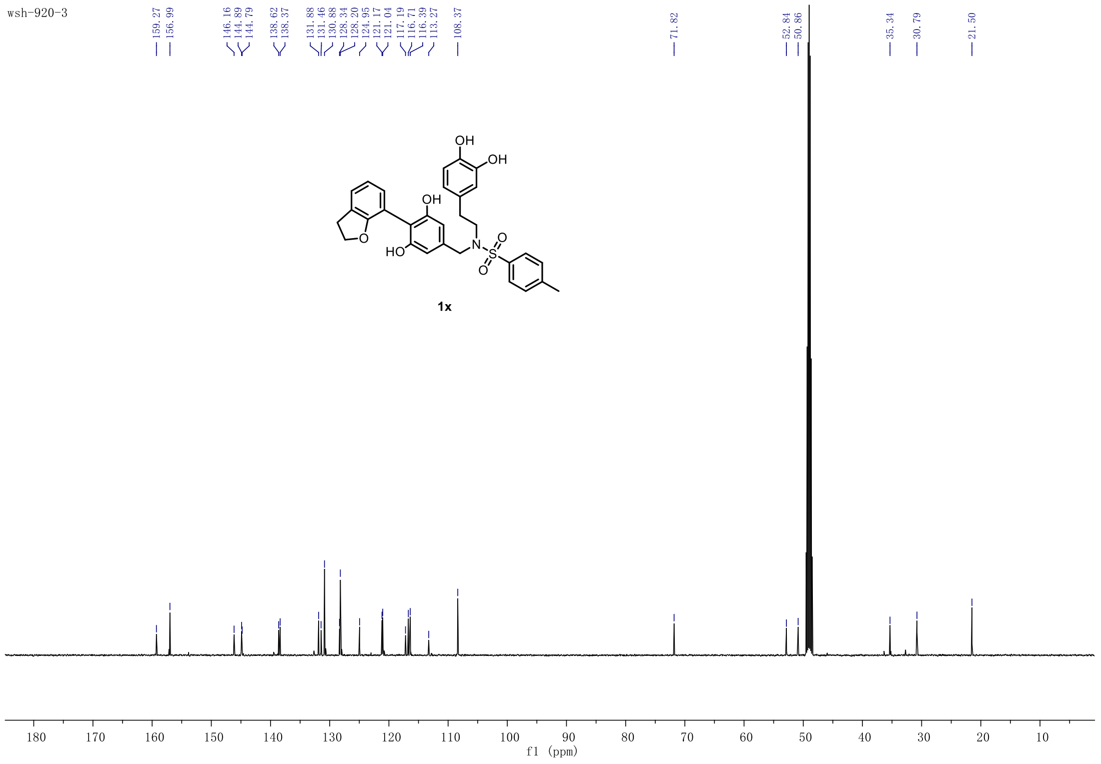

^{1}H NMR spectrum of compound 1y (500 MHz) in Acetone-d_6

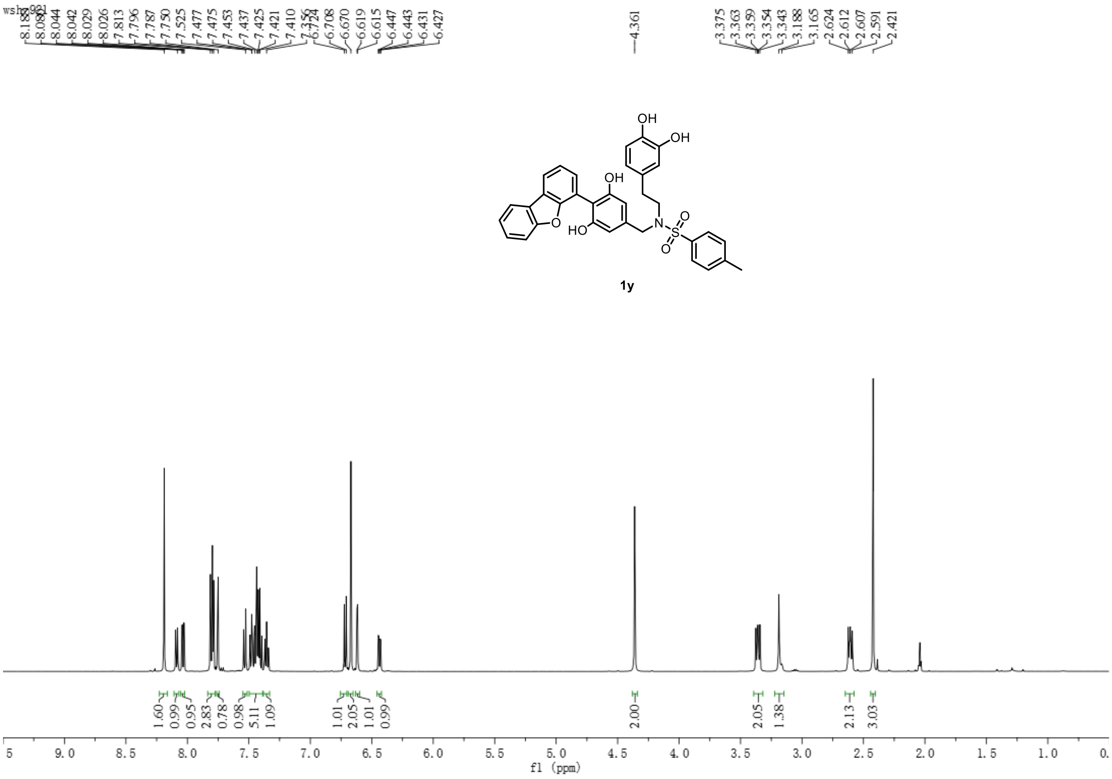

^{13}C NMR spectrum of compound 1y (125 MHz) in Acetone-d_6

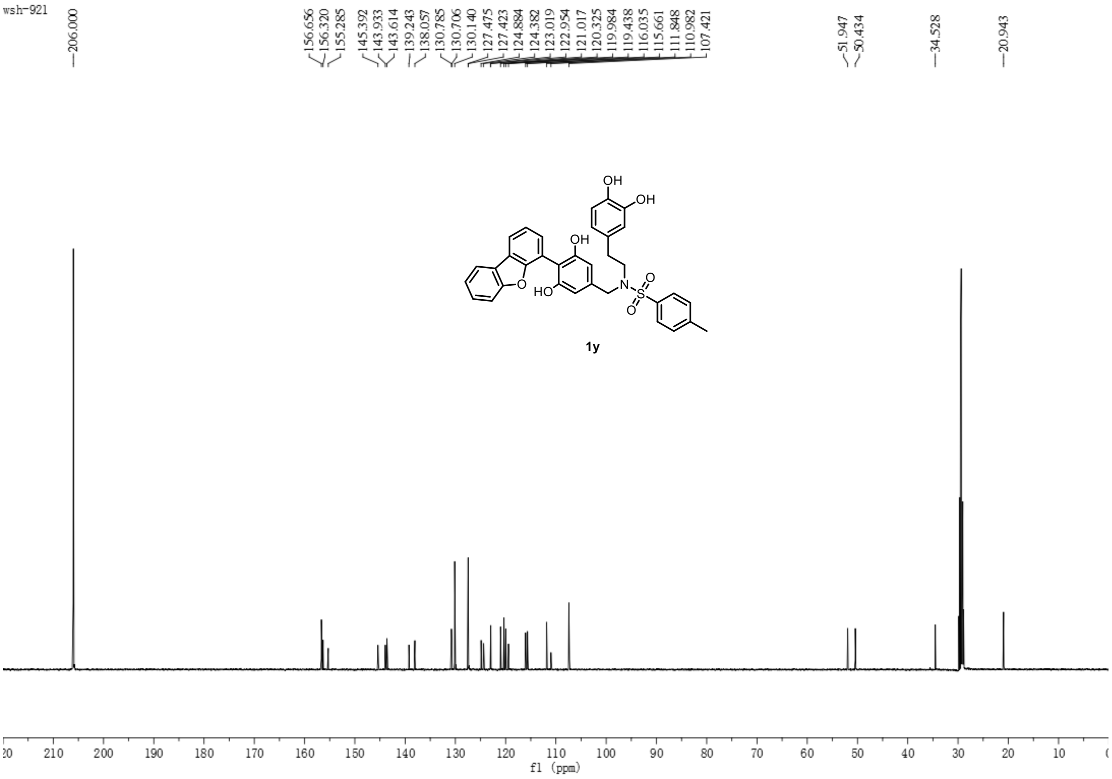

^{1}H NMR spectrum of compound 1z (500 MHz) in Acetone-*d*

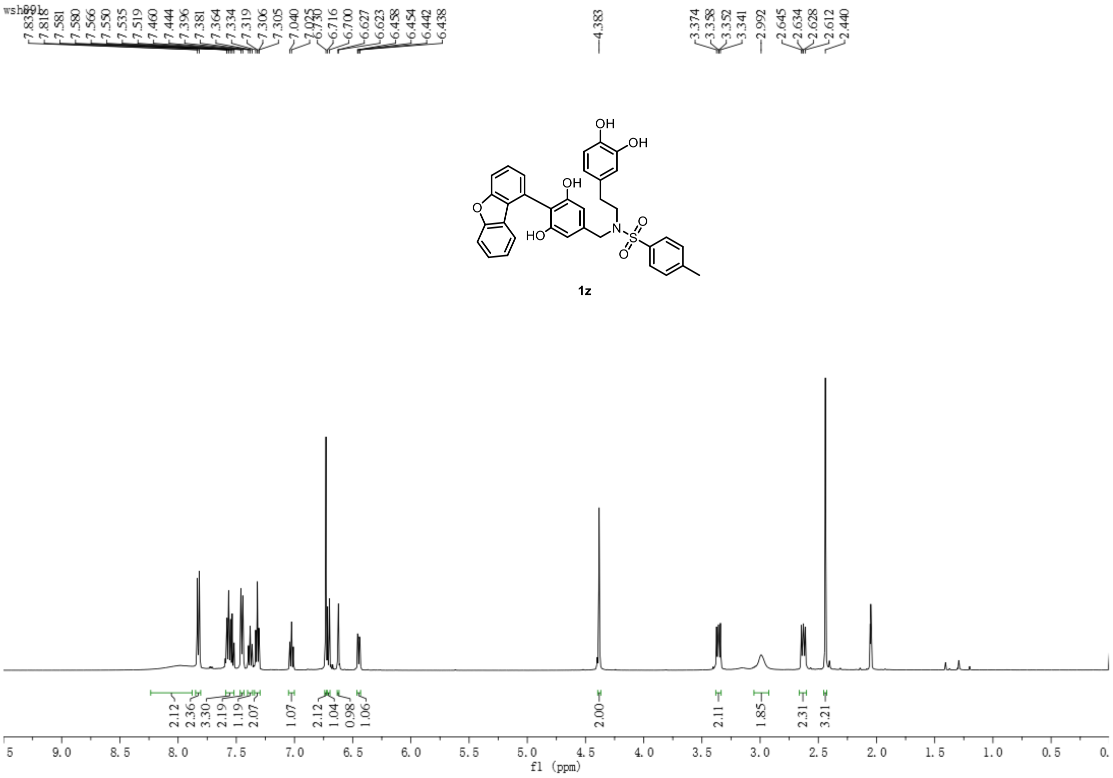

^{13}C NMR spectrum of compound 1z (125 MHz) in Acetone-d_6

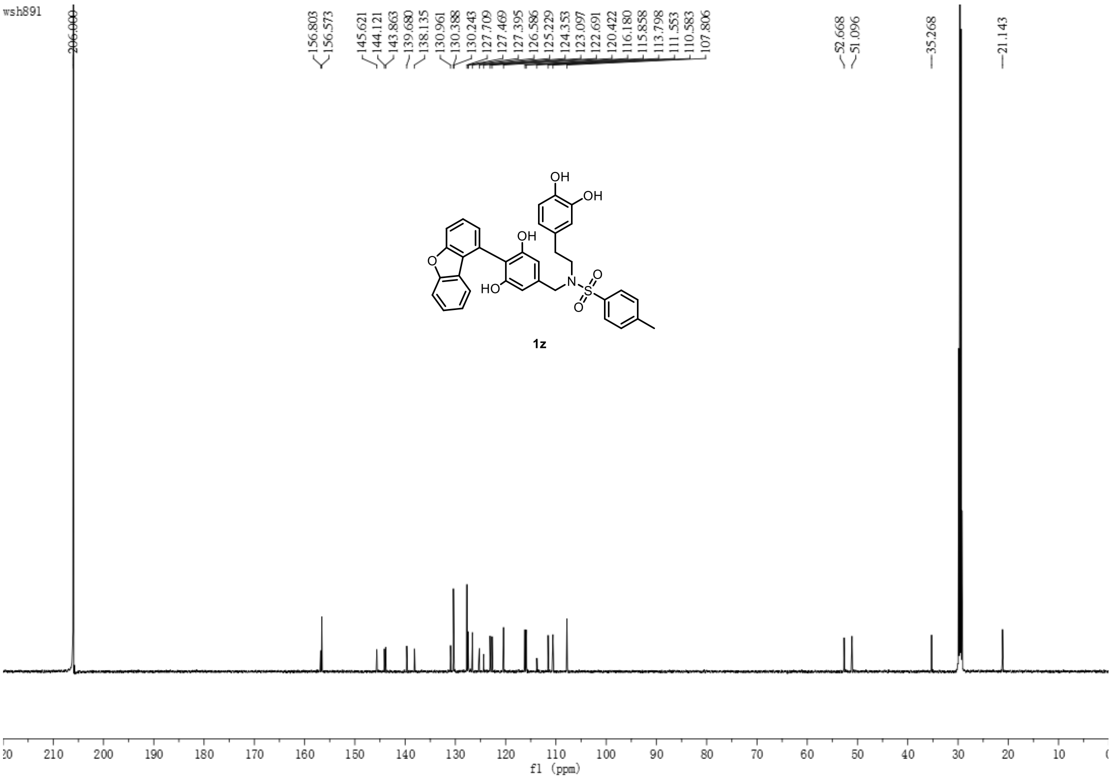

^{1}H NMR spectrum of compound 1aa (500 MHz) in CD_3OD

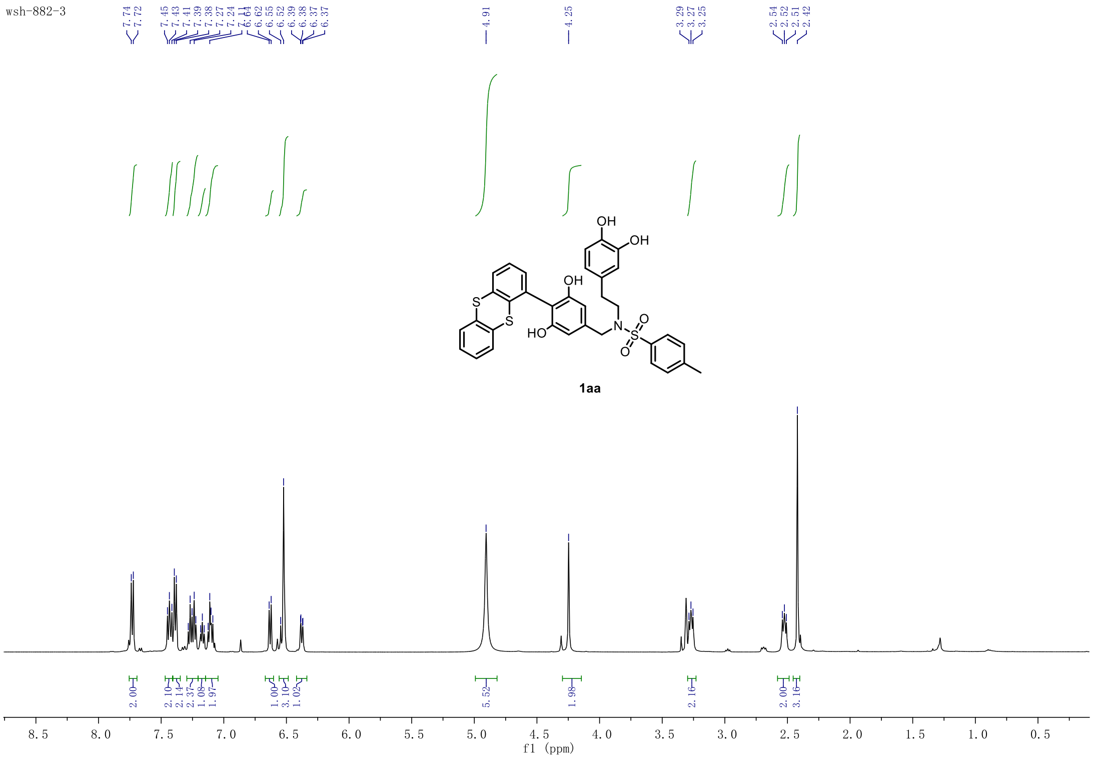

^{13}C NMR spectrum of compound 1aa (125 MHz) in CD_3OD

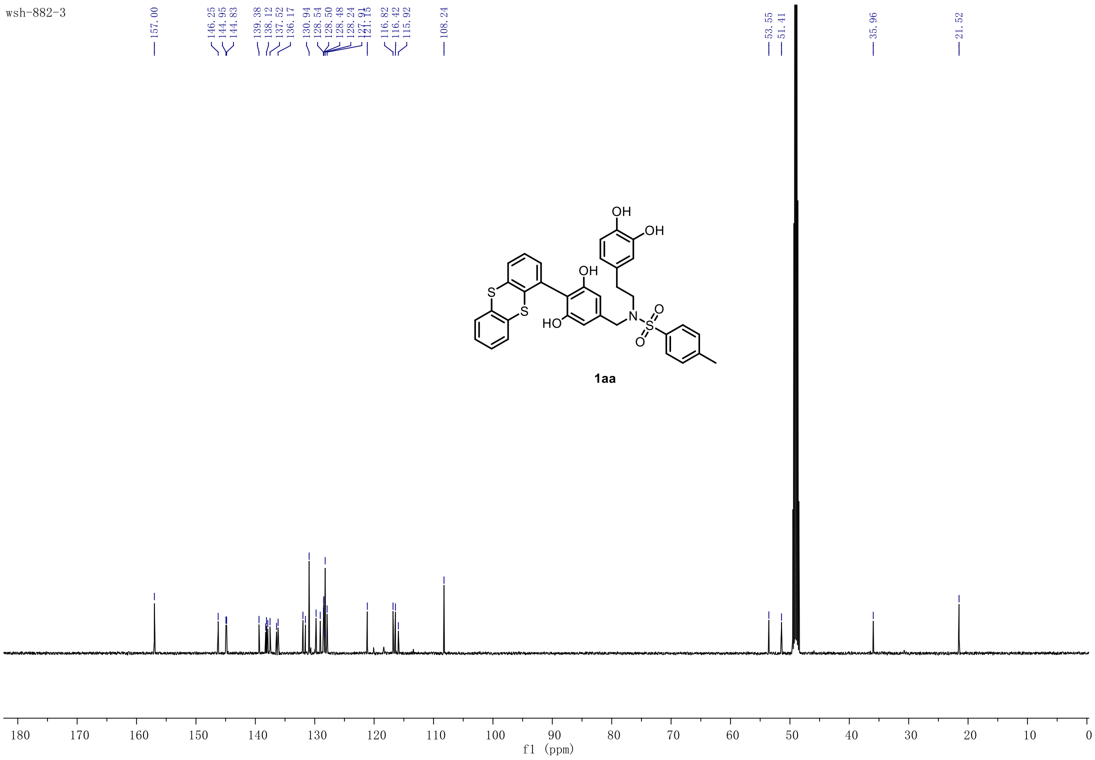

^{1}H NMR spectrum of compound 1ab (500 MHz) in Acetone-d_6

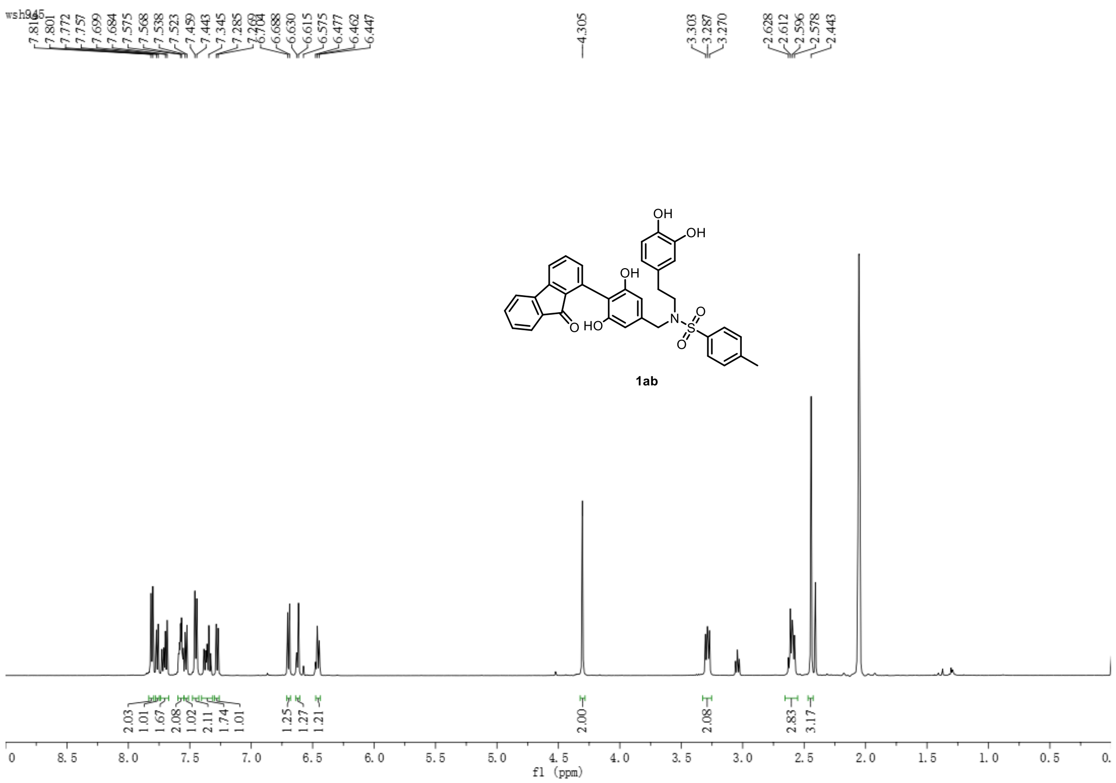

^{13}C NMR spectrum of compound 1ab (125 MHz) in Acetone-d_6

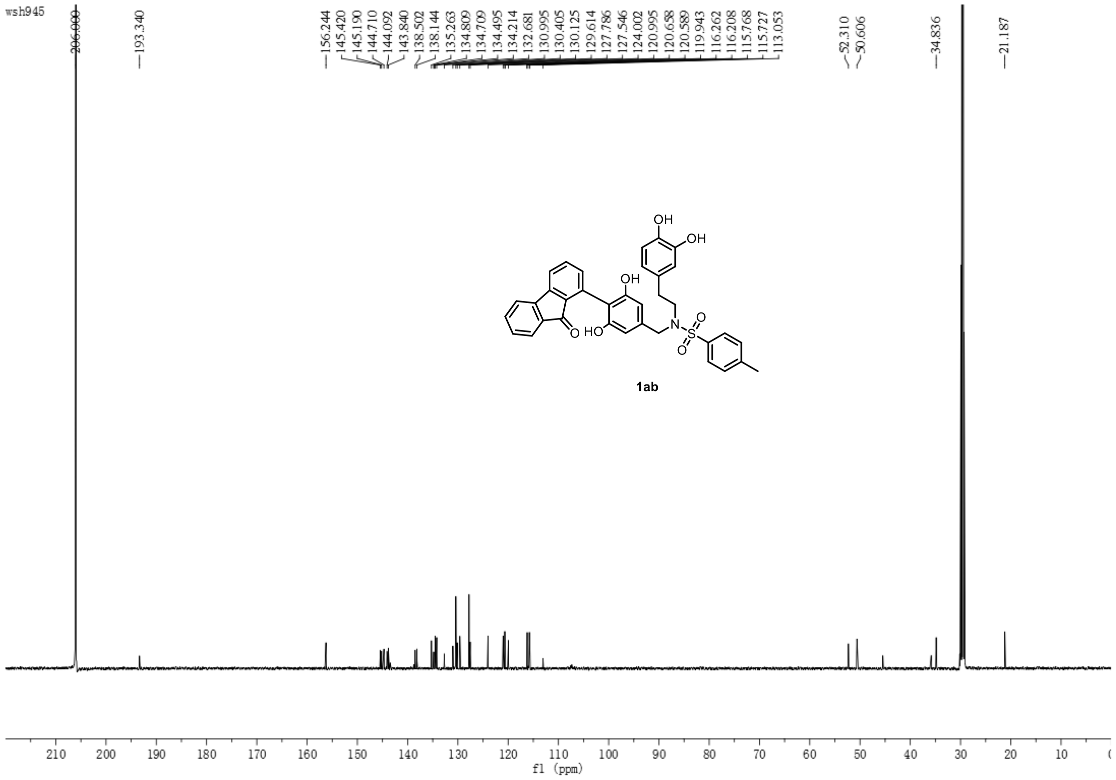

^{1}H NMR spectrum of compound 1ac (500 MHz) in Acetone-d_6

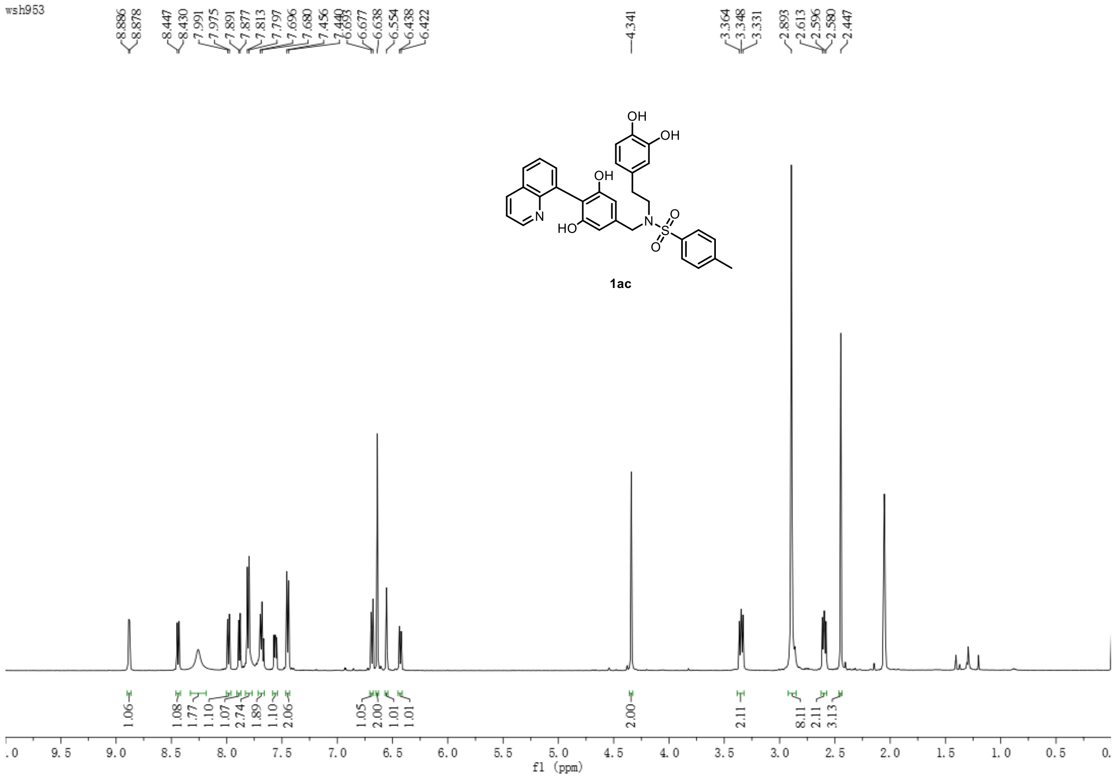

^{13}C NMR spectrum of compound 1ac (125 MHz) in Acetone-d_6

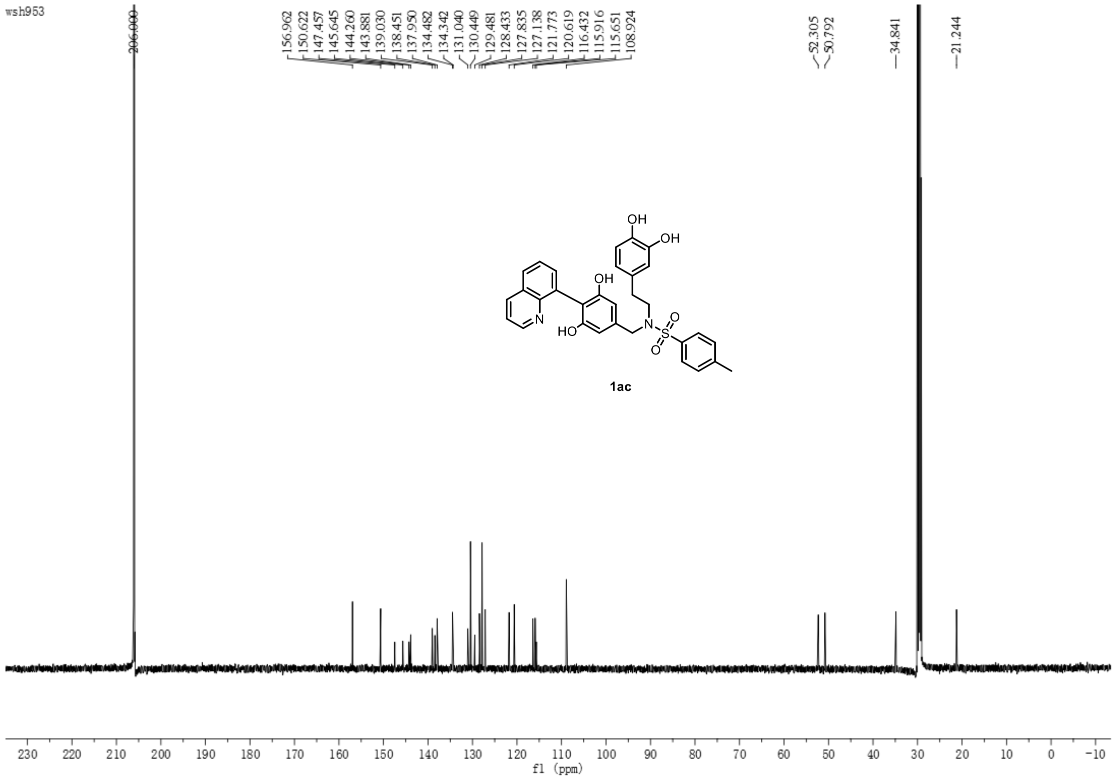

^{1}H NMR spectrum of compound 1ad (500 MHz) in Acetone-d_6

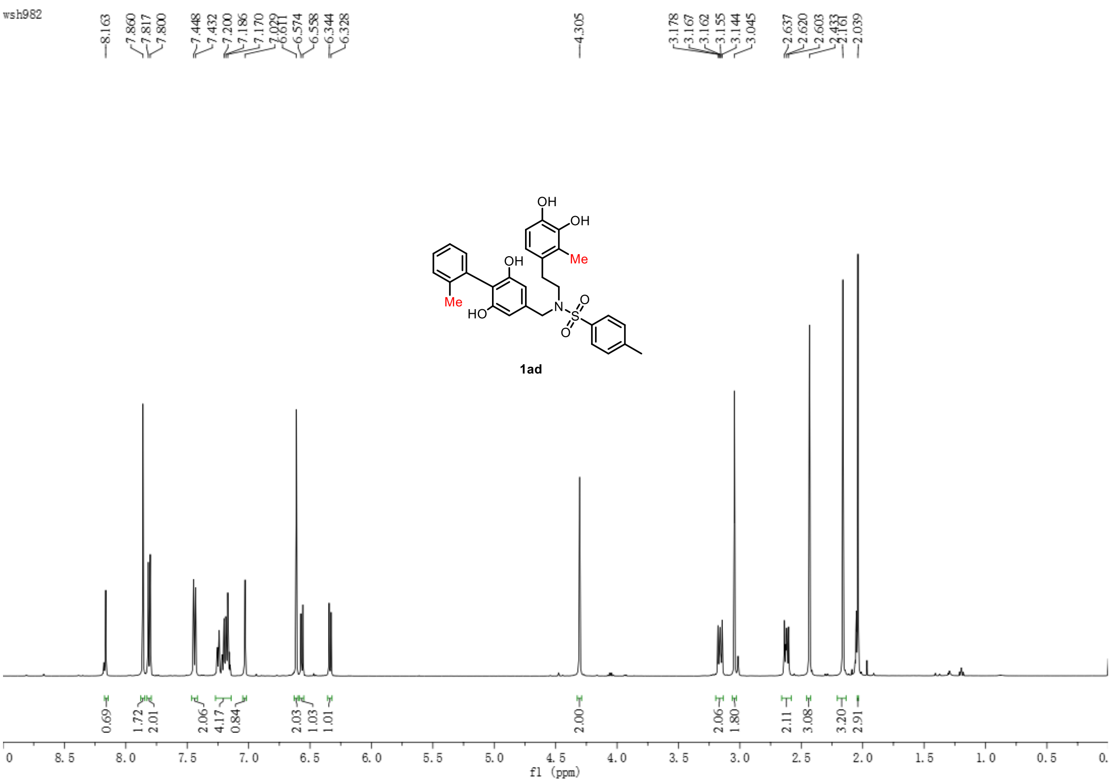

^{13}C NMR spectrum of compound 1ad (125 MHz) in Acetone-d_6

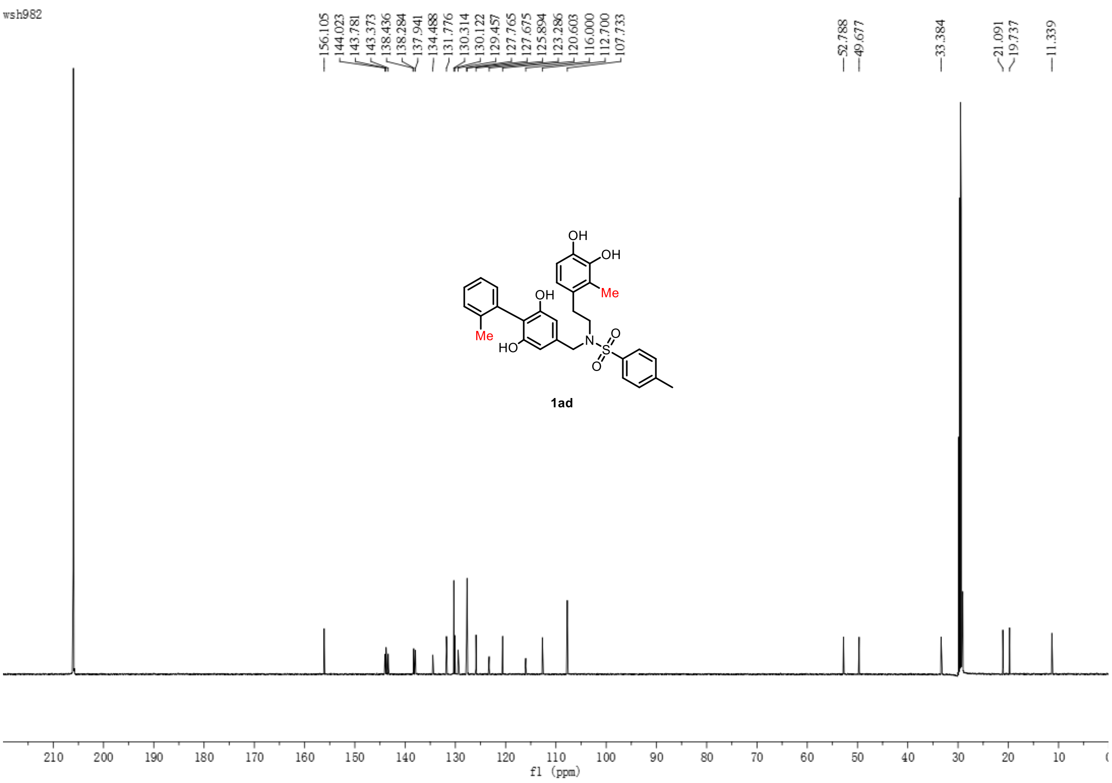

^{1}H NMR spectrum of compound 1ae (500 MHz) in Acetone-d_6

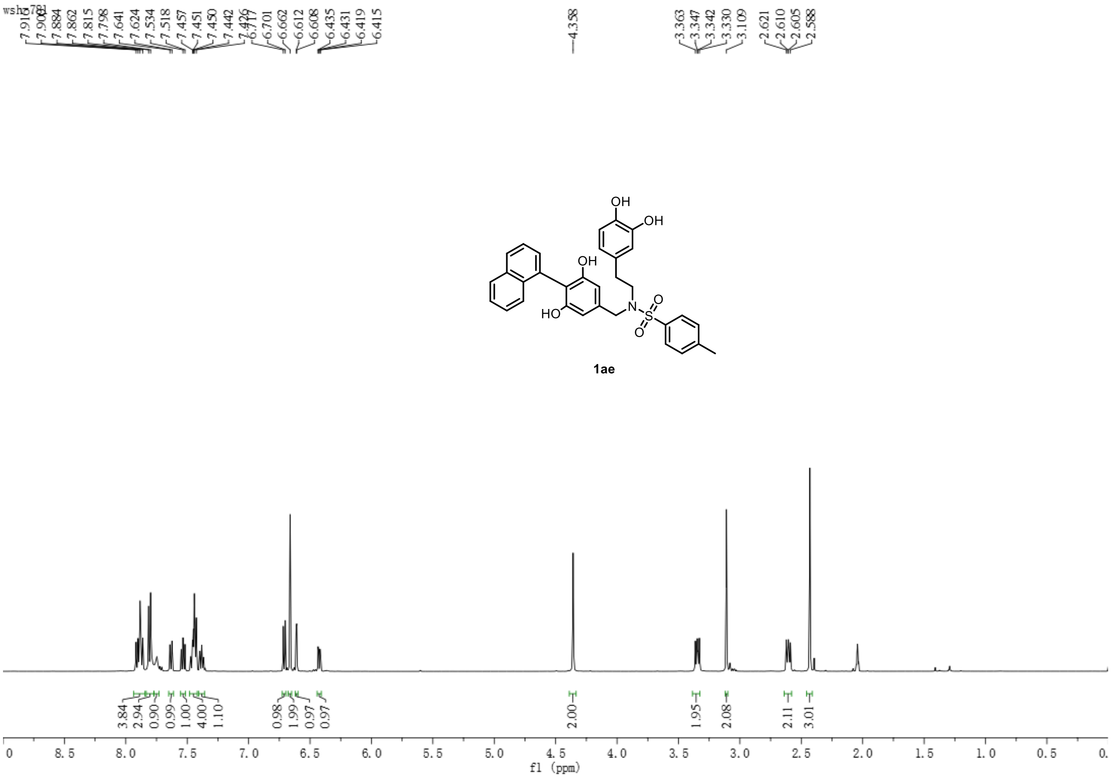

^{13}C NMR spectrum of compound 1ae (125 MHz) in Acetone-d_6

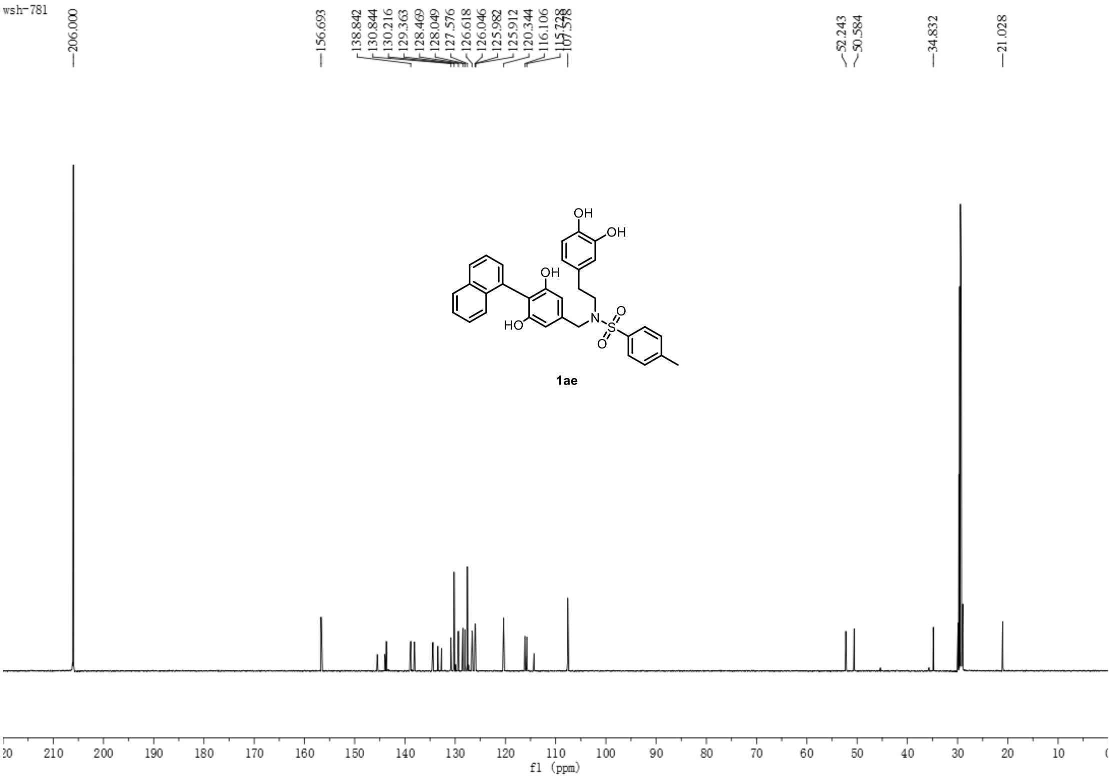

^{1}H NMR spectrum of compound 1af (500 MHz) in Acetone-d_6

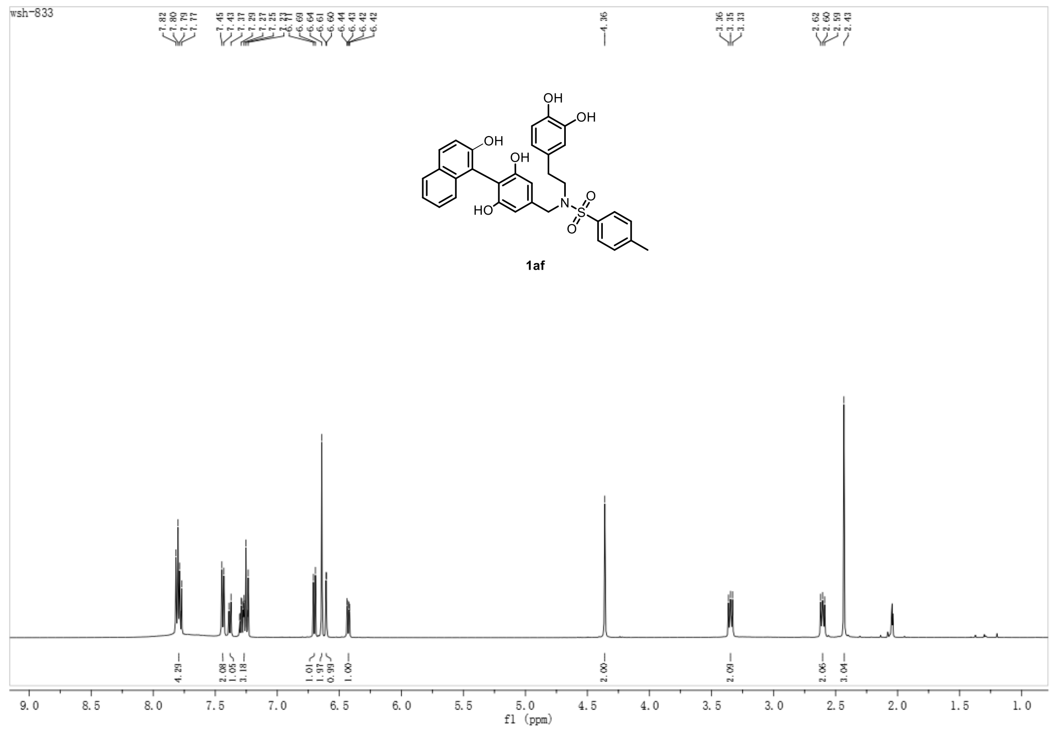

^{13}C NMR spectrum of compound 1af (125 MHz) in Acetone-d_6

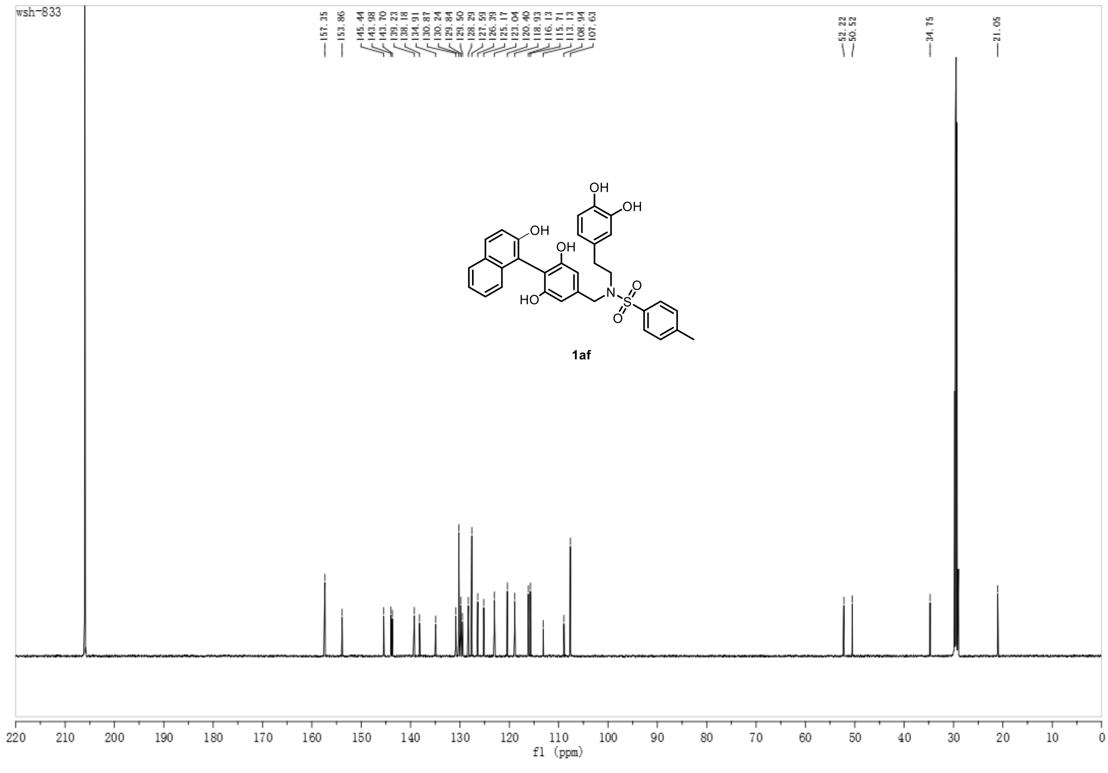

^{1}H NMR spectrum of compound 1ag (500 MHz) in Acetone-d_6

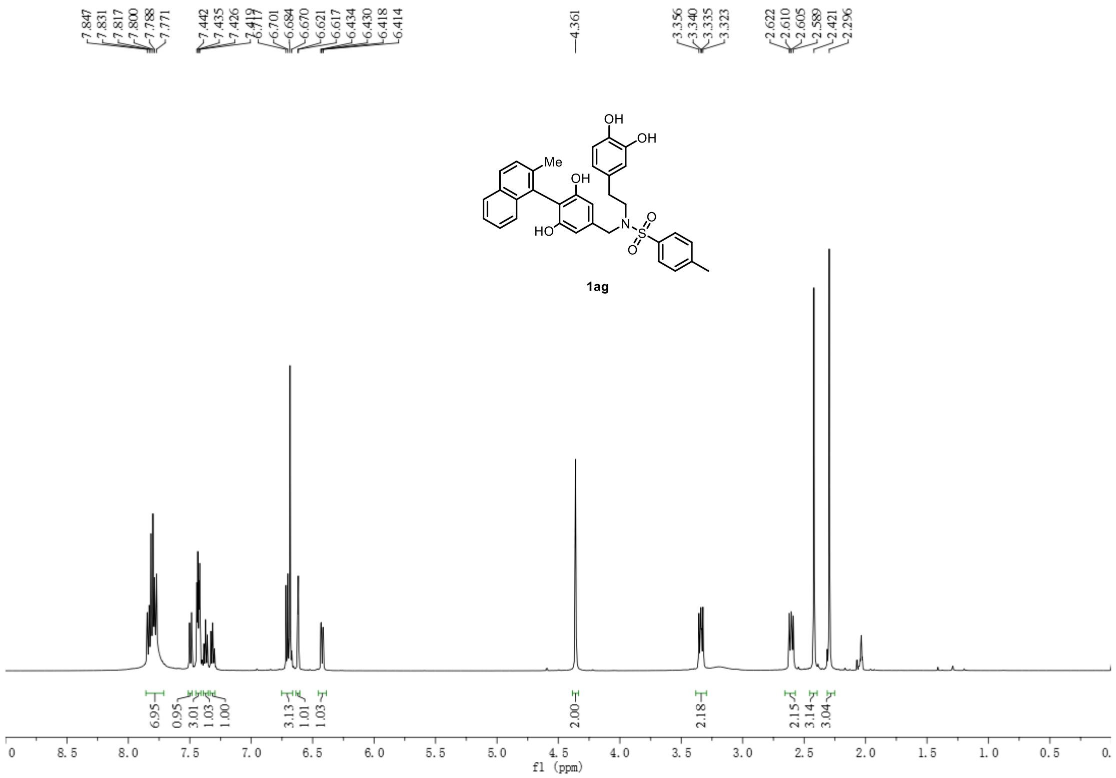

^{13}C NMR spectrum of compound 1ag (125 MHz) in Acetone-d_6

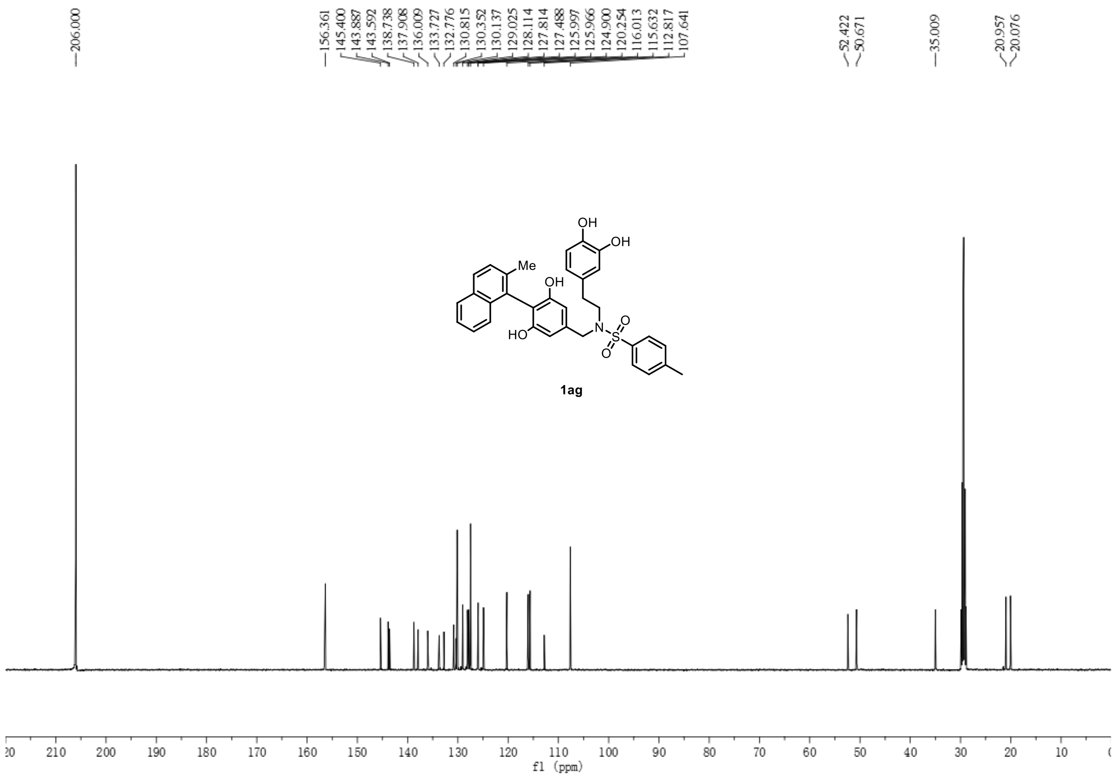

^{1}H NMR spectrum of compound 1ah (500 MHz) in Acetone-d_6

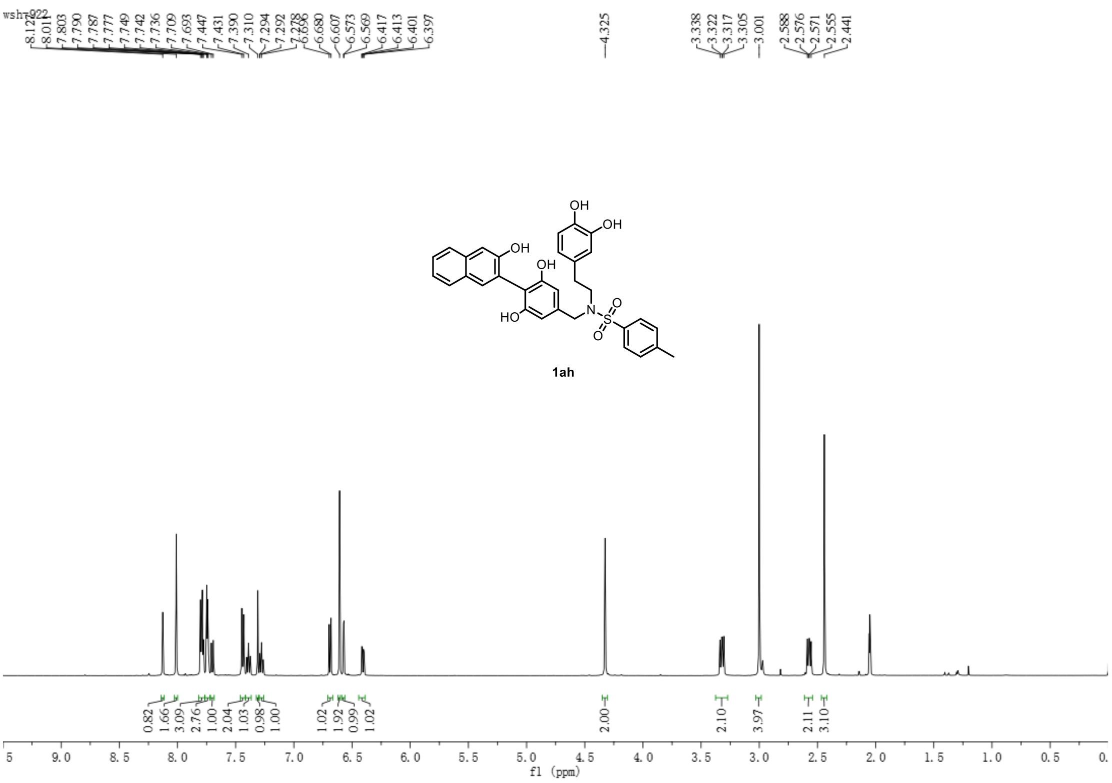

^{13}C NMR spectrum of compound 1ah (125 MHz) in Acetone-d_6

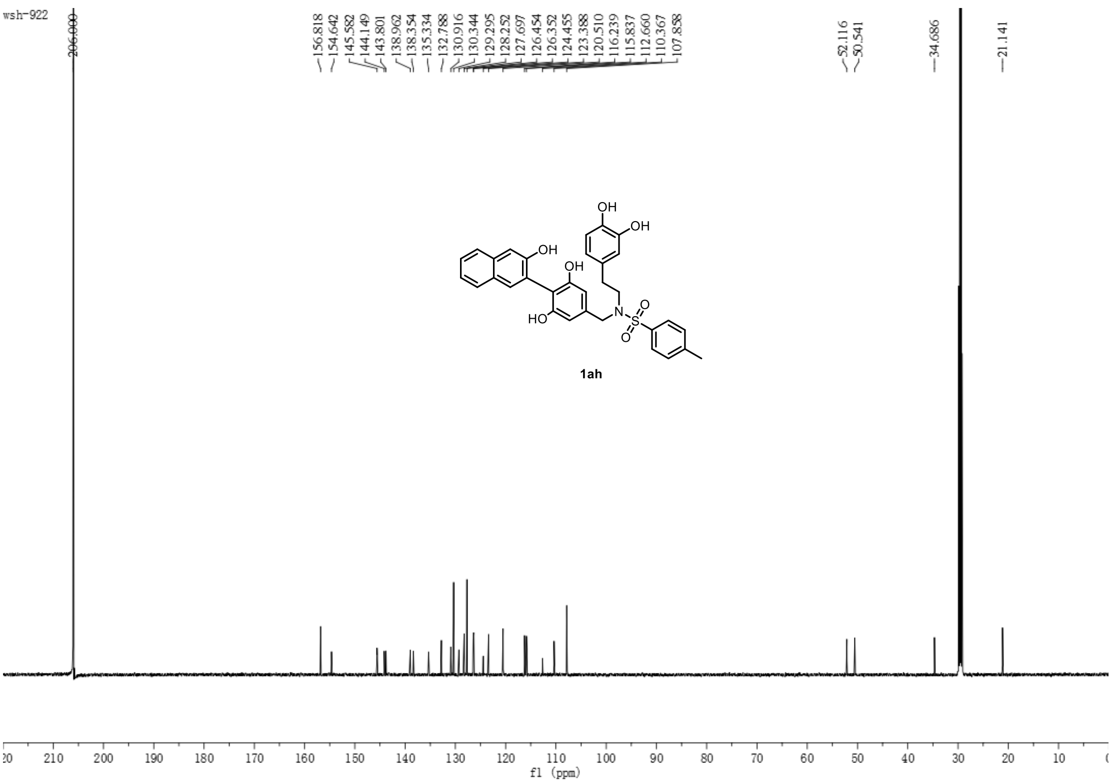

^{1}H NMR spectrum of compound 1ai (500 MHz) in Acetone-d_6

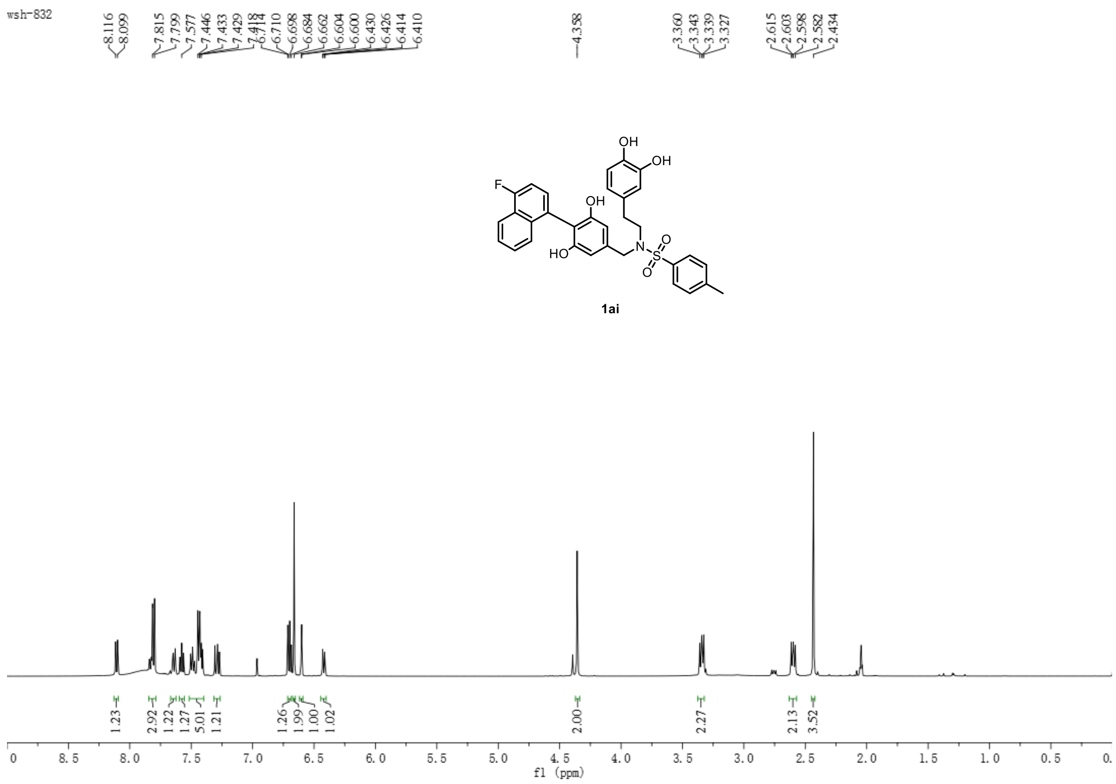

^{13}C NMR spectrum of compound 1ai (125 MHz) in Acetone-d_6

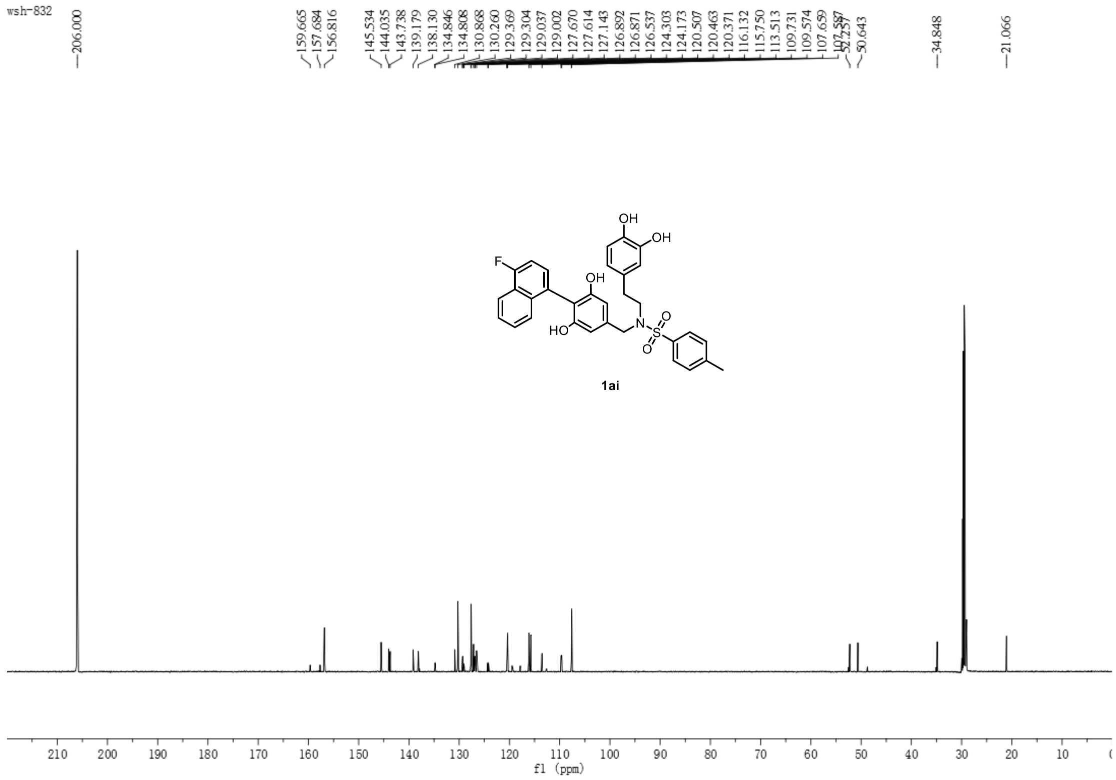

^{19}F NMR spectrum of compound 2ai (470 MHz) in CD_3OD

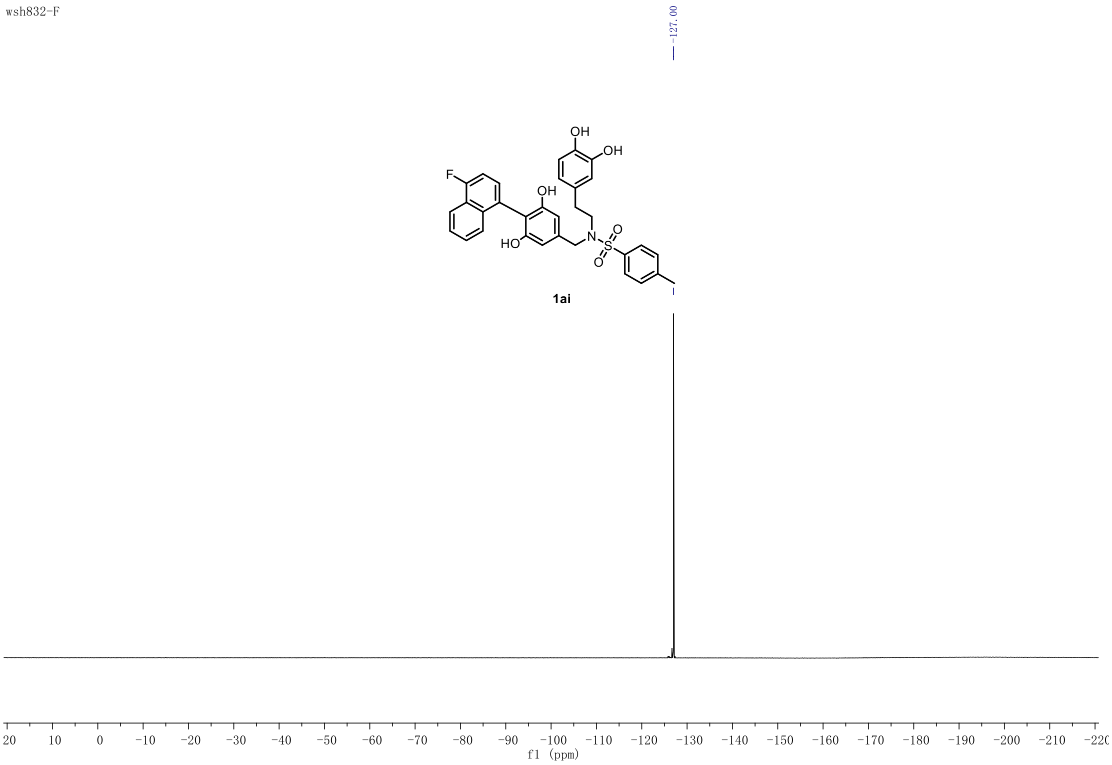

^{1}H NMR spectrum of compound 1aj (500 MHz) in Acetone-d_6

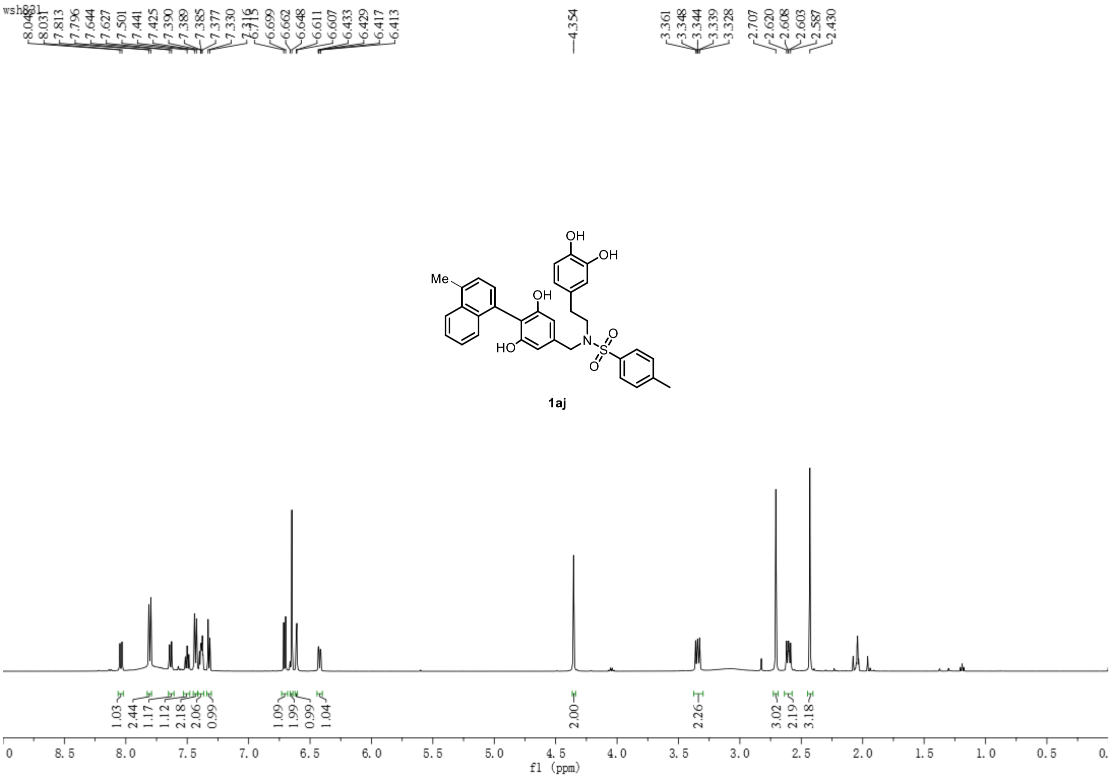

^{13}C NMR spectrum of compound 1aj (125 MHz) in Acetone-d_6

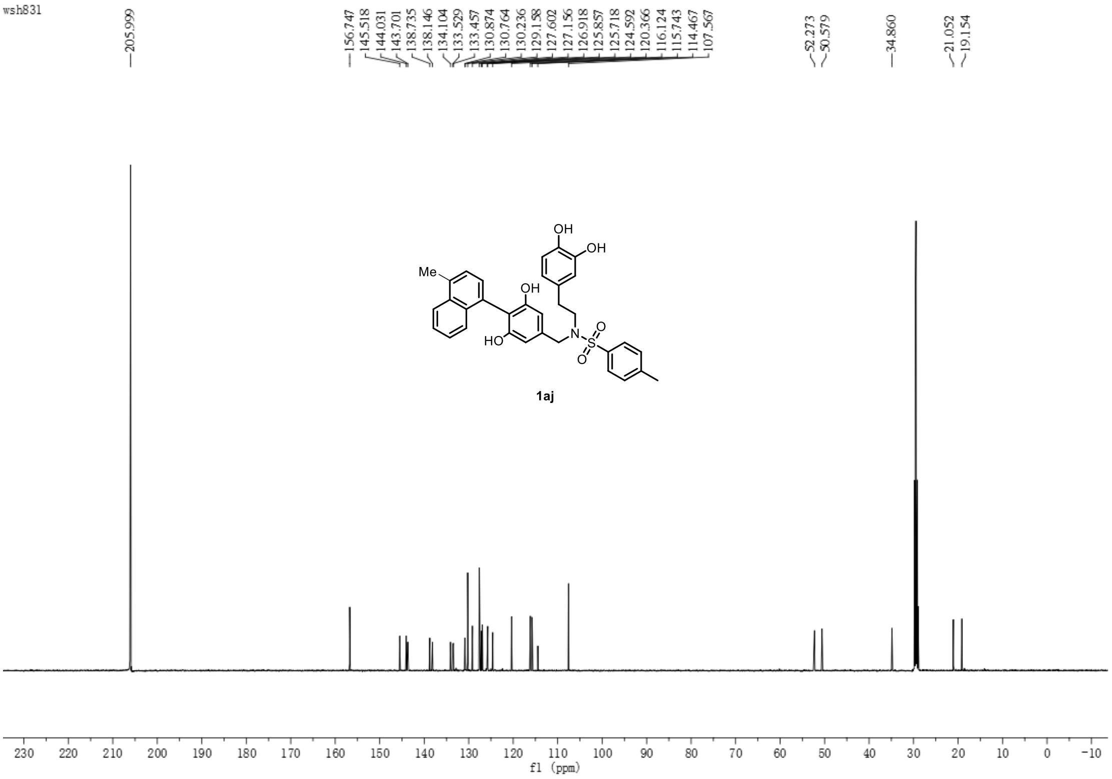

^{1}H NMR spectrum of compound 1ak (500 MHz) in Acetone-d_6

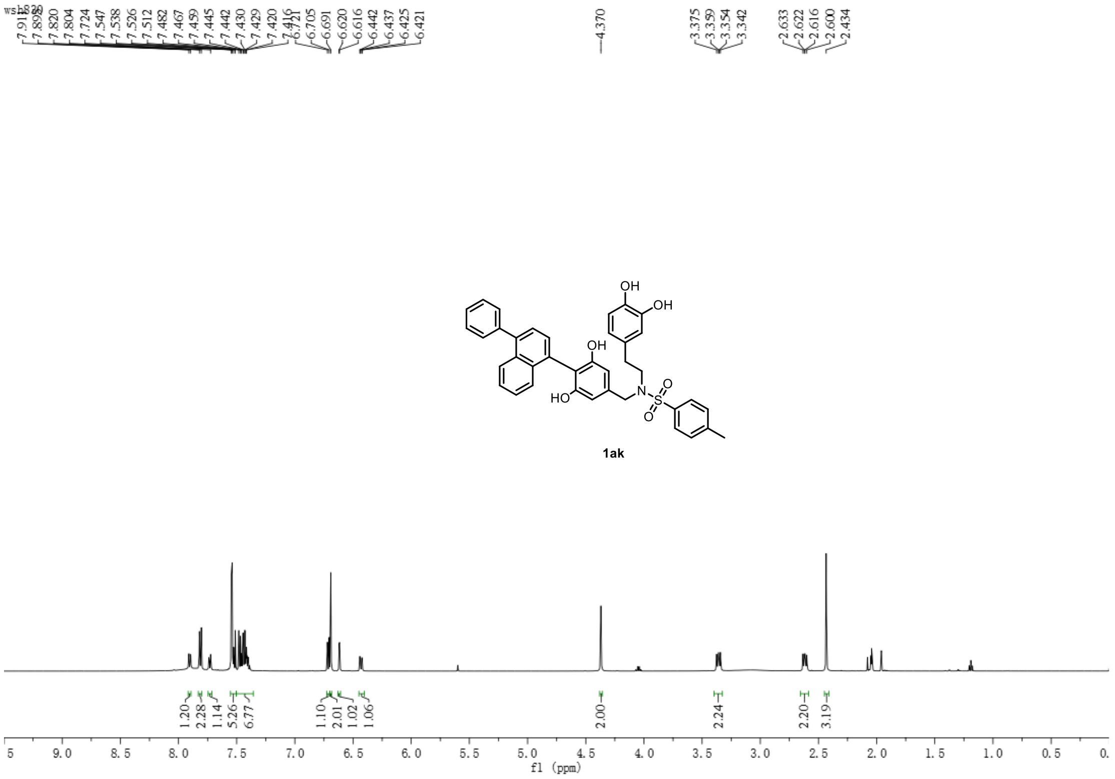

^{13}C NMR spectrum of compound 1ak (125 MHz) in Acetone-d_6

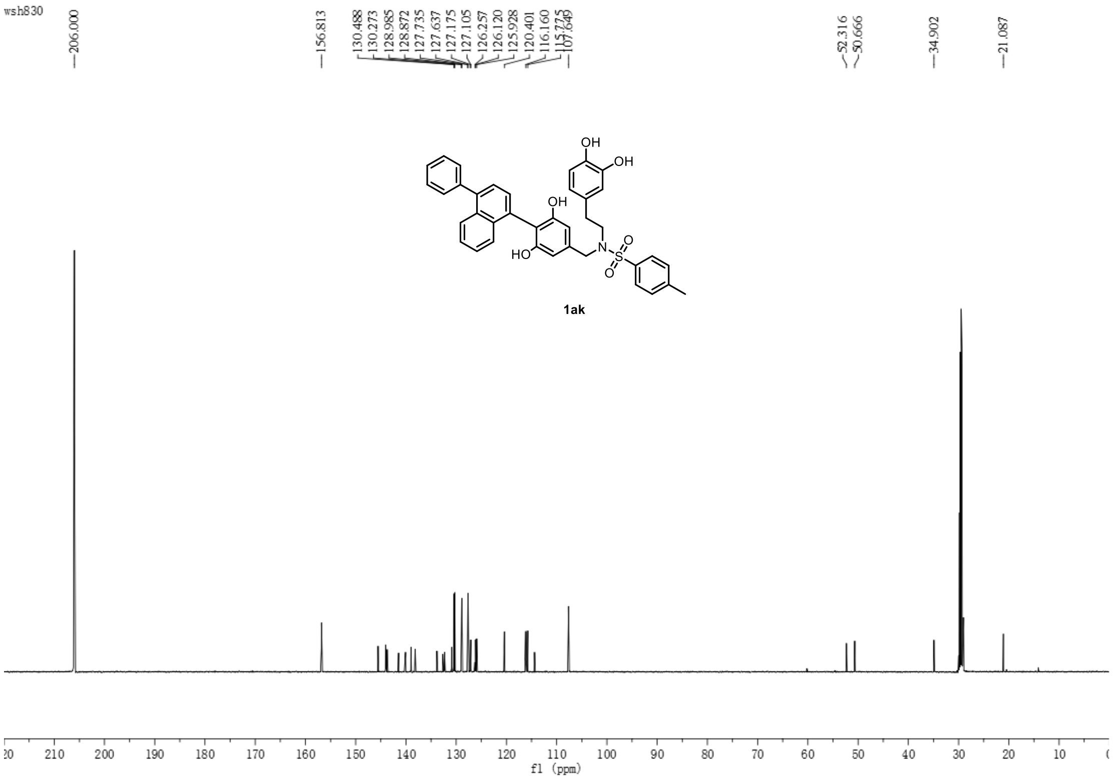

^{1}H NMR spectrum of compound 1al (500 MHz) in Acetone-d_6

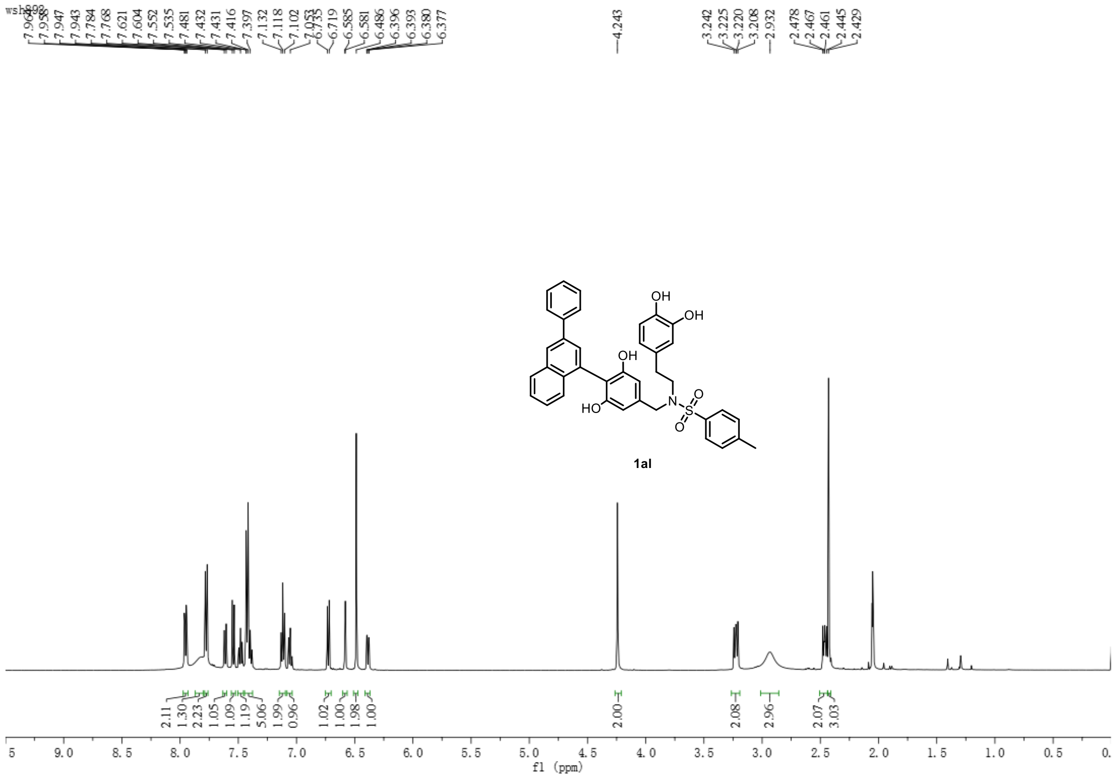

^{13}C NMR spectrum of compound 1al (125 MHz) in Acetone-d_6

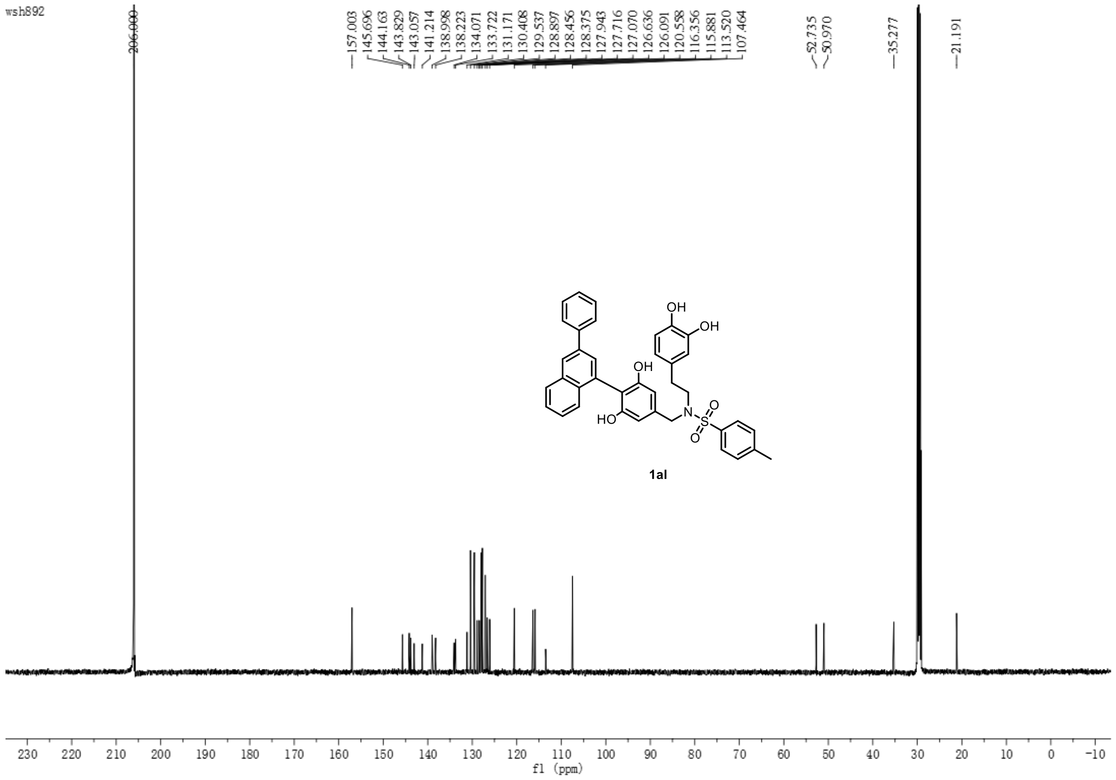

^{1}H NMR spectrum of compound 1am (500 MHz) in Acetone-d_6

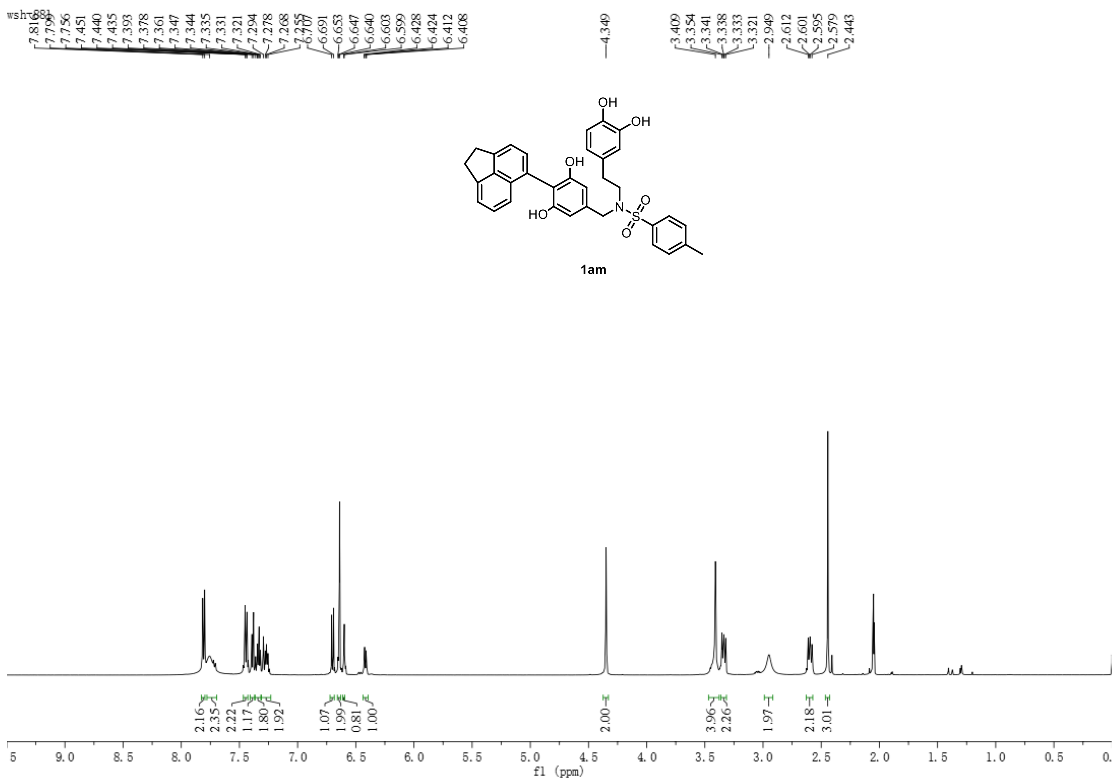

^{13}C NMR spectrum of compound 1am (125 MHz) in Acetone-d_6

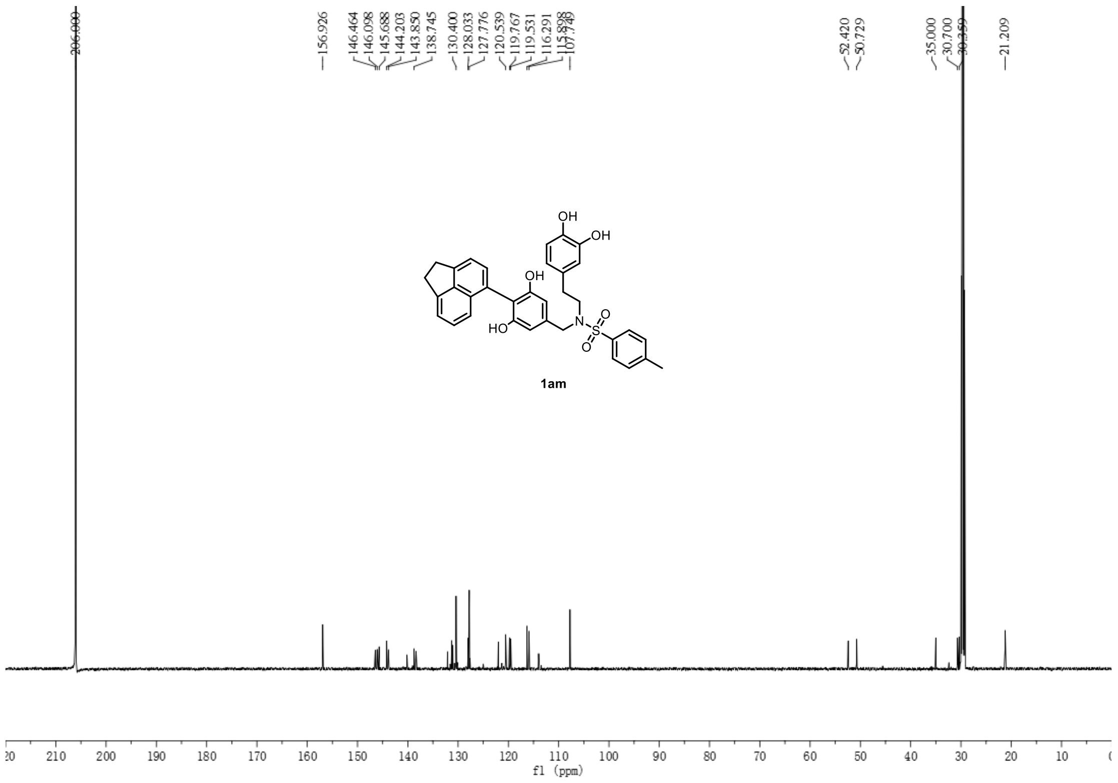

^{1}H NMR spectrum of compound 1an (500 MHz) in Acetone-d_6

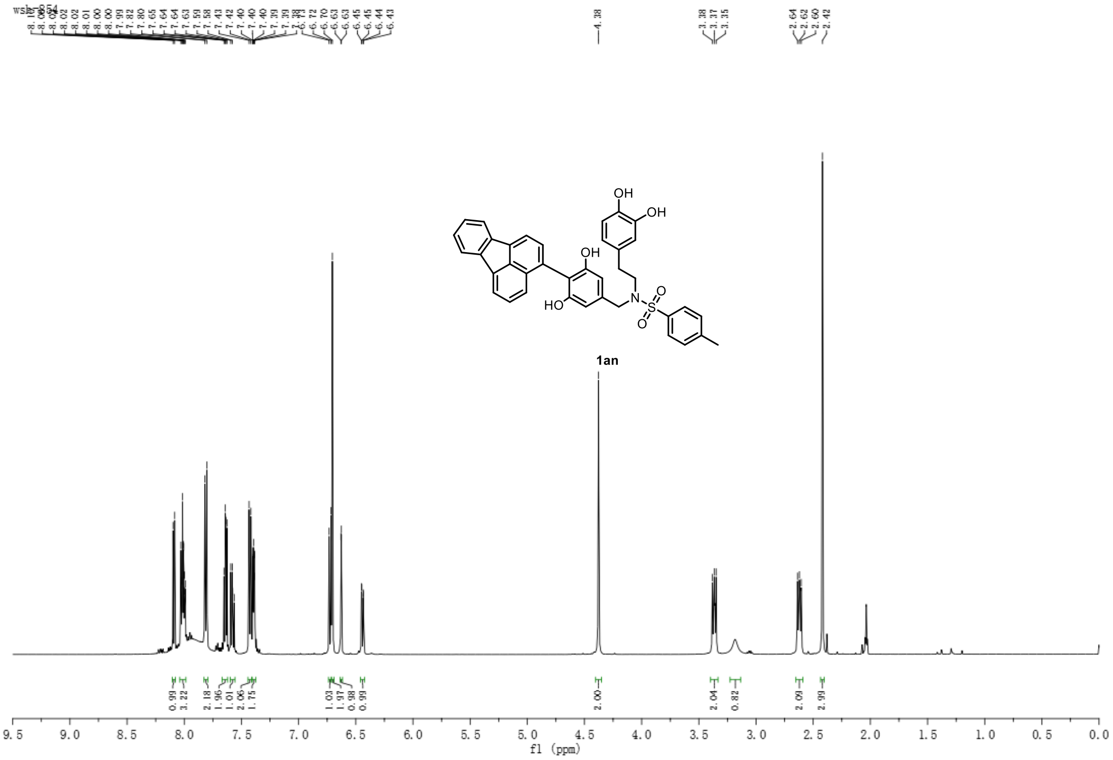

^{13}C NMR spectrum of compound 1an (125 MHz) in Acetone-d_6

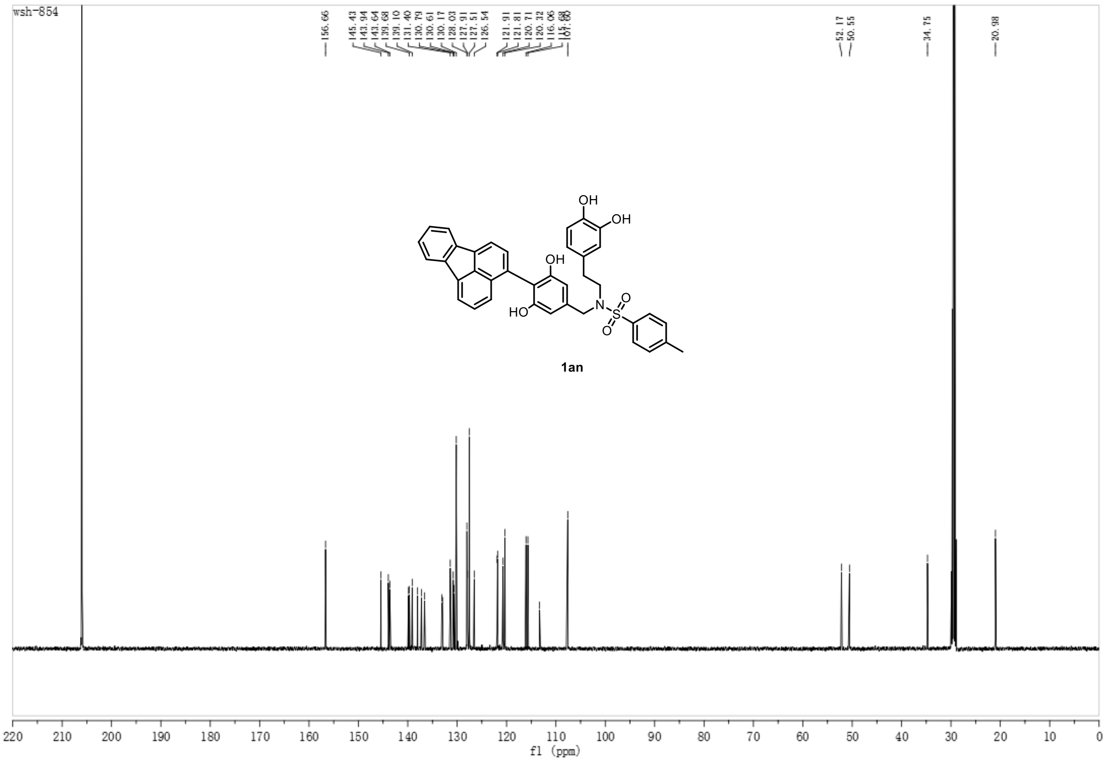

^{1}H NMR spectrum of compound 1ao (500 MHz) in Acetone-d_6

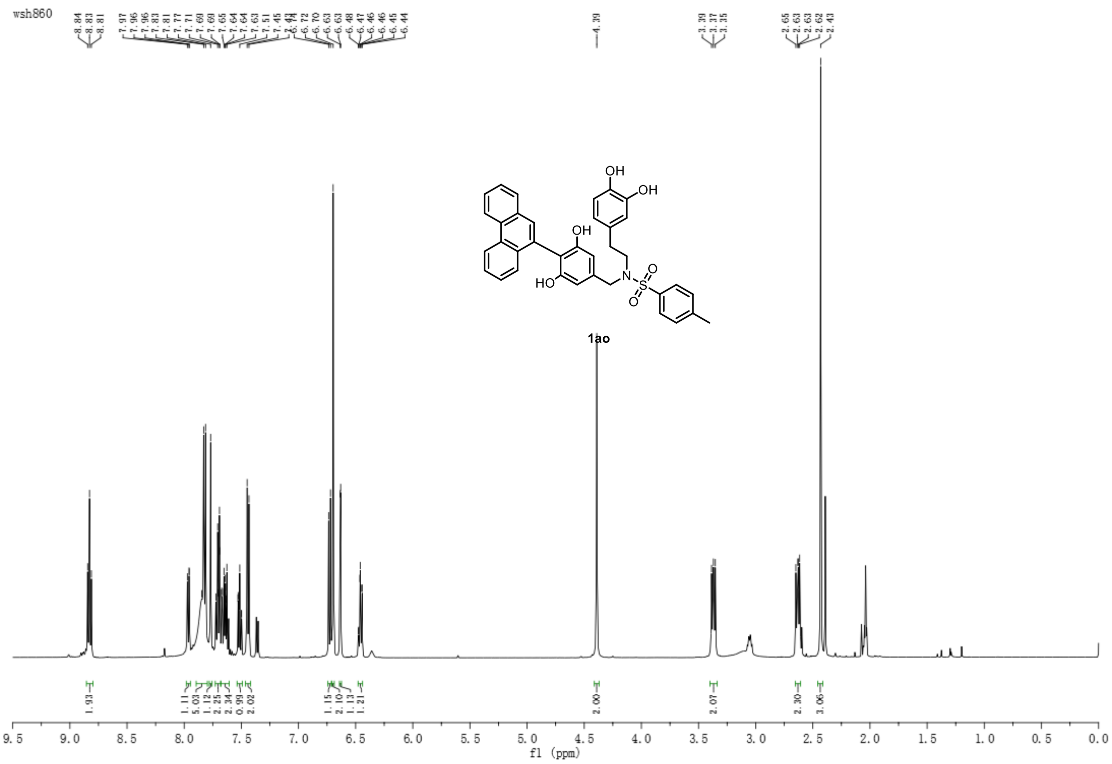

^{13}C NMR spectrum of compound 1ao (125 MHz) in Acetone-d_6

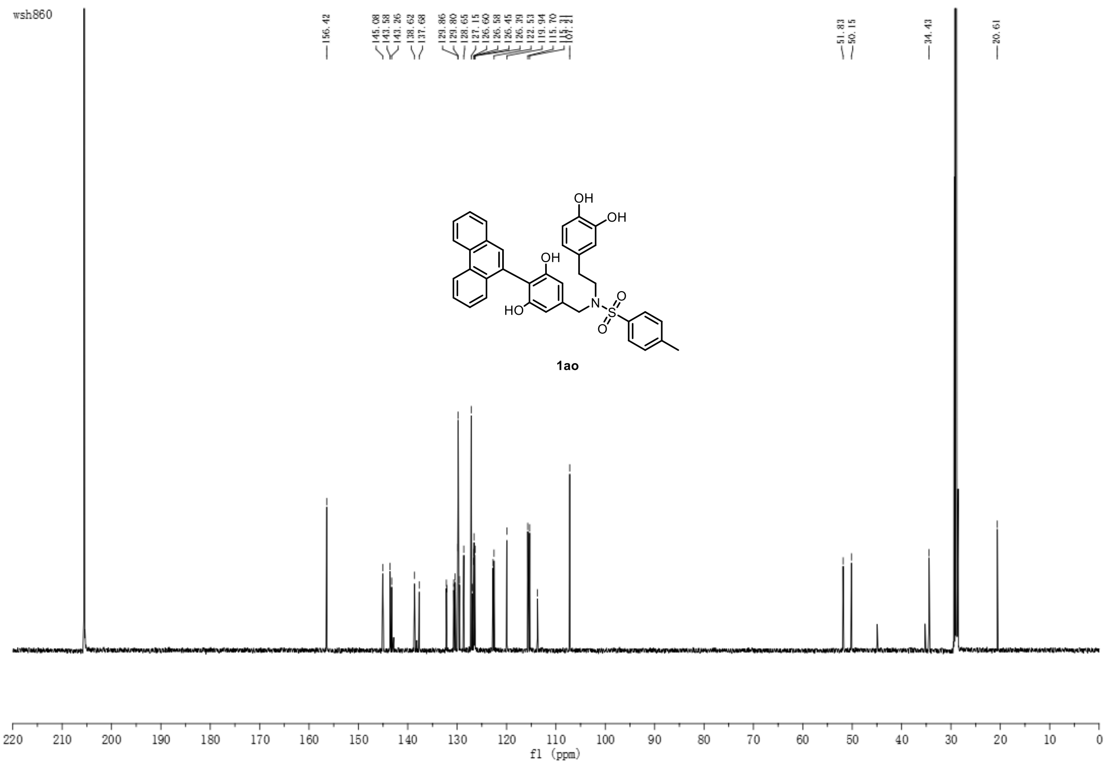

^{1}H NMR spectrum of compound 1ap (500 MHz) in Acetone-d_6

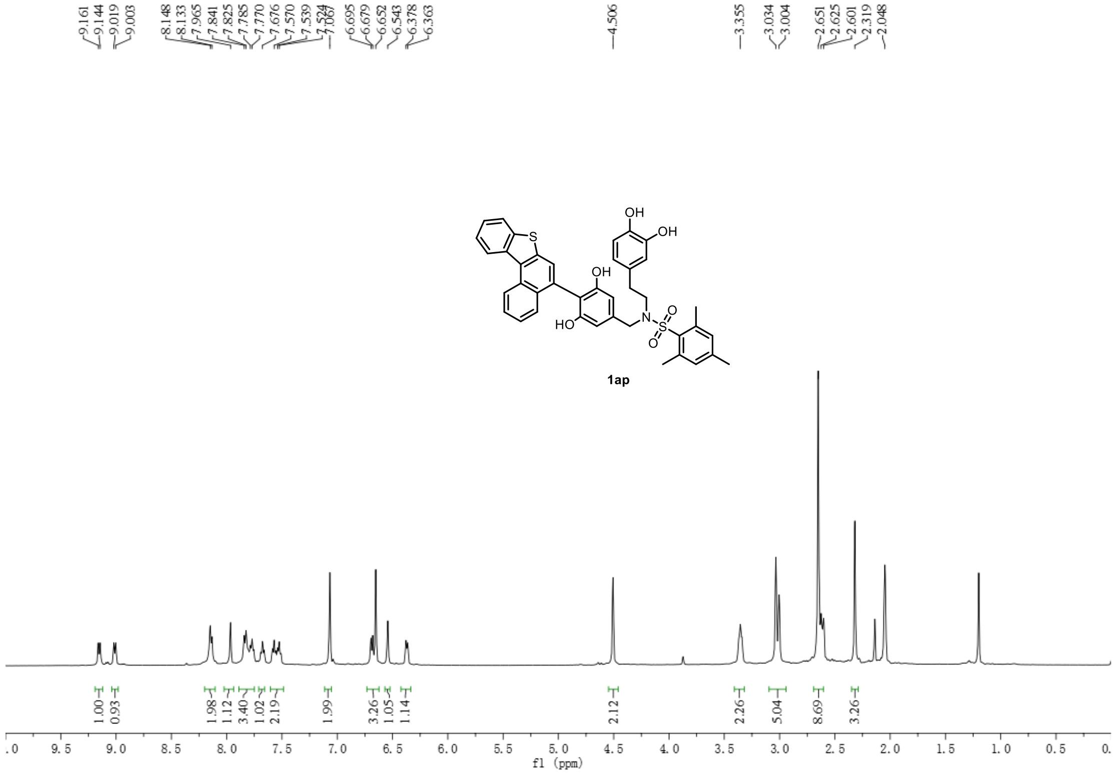

^{13}C NMR spectrum of compound 1ap (125 MHz) in Acetone-d_6

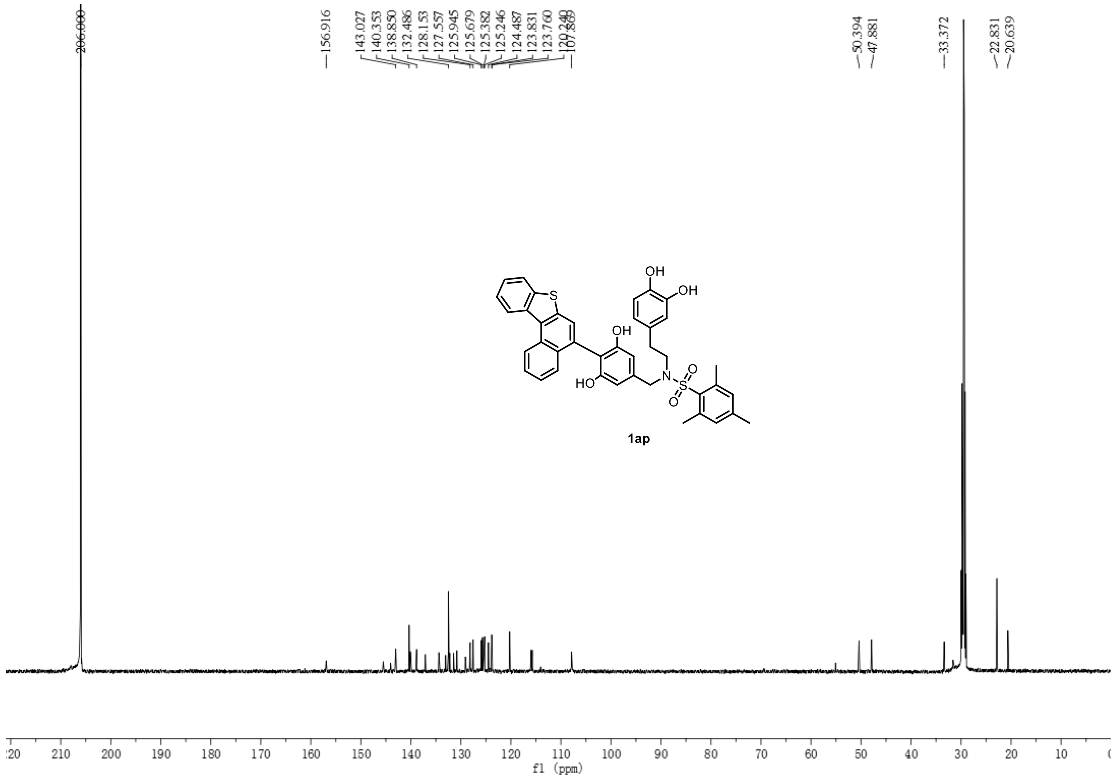

^{1}H NMR spectrum of compound 1aq (500 MHz) in CD_3OD

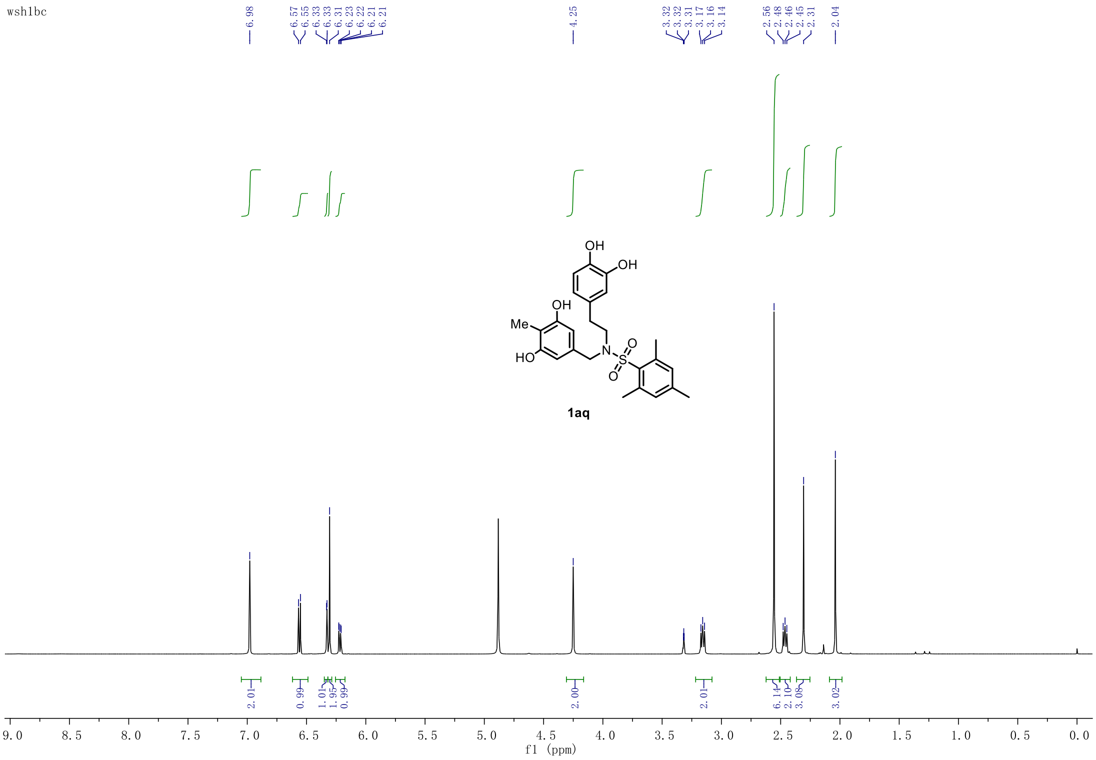

^{13}C NMR spectrum of compound 1aq (125 MHz) in CD_3OD

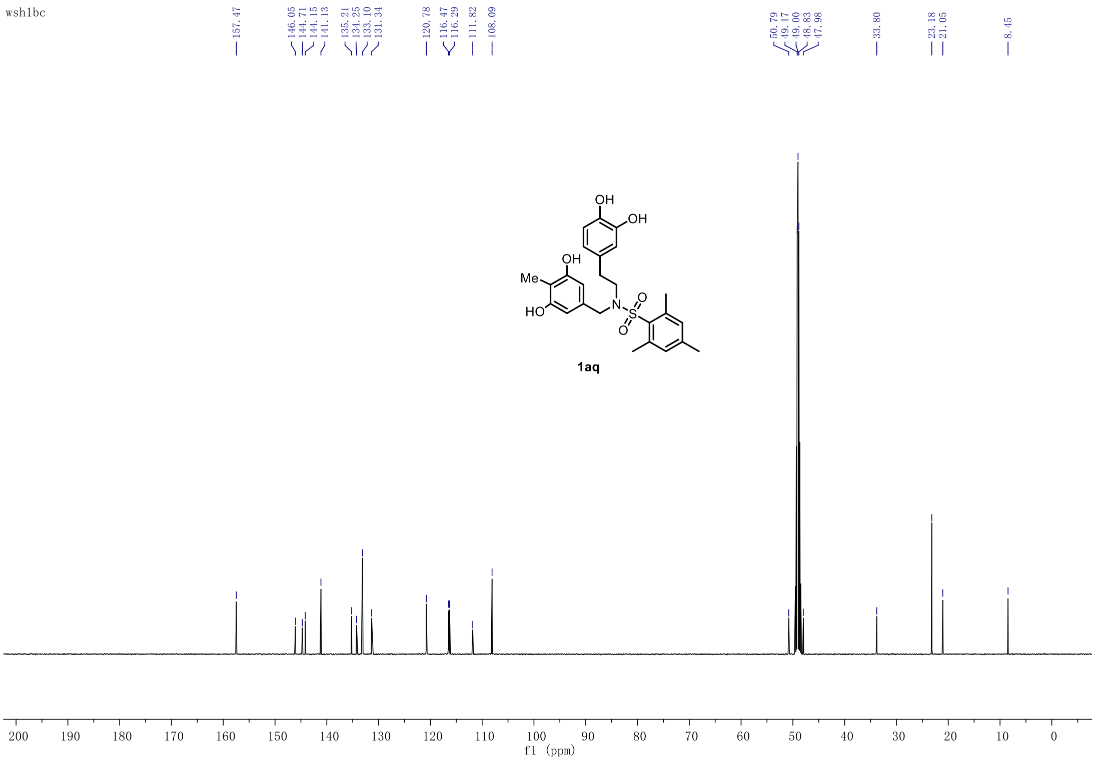

^{1}H NMR spectrum of compound 1ar (500 MHz) in CD_3OD

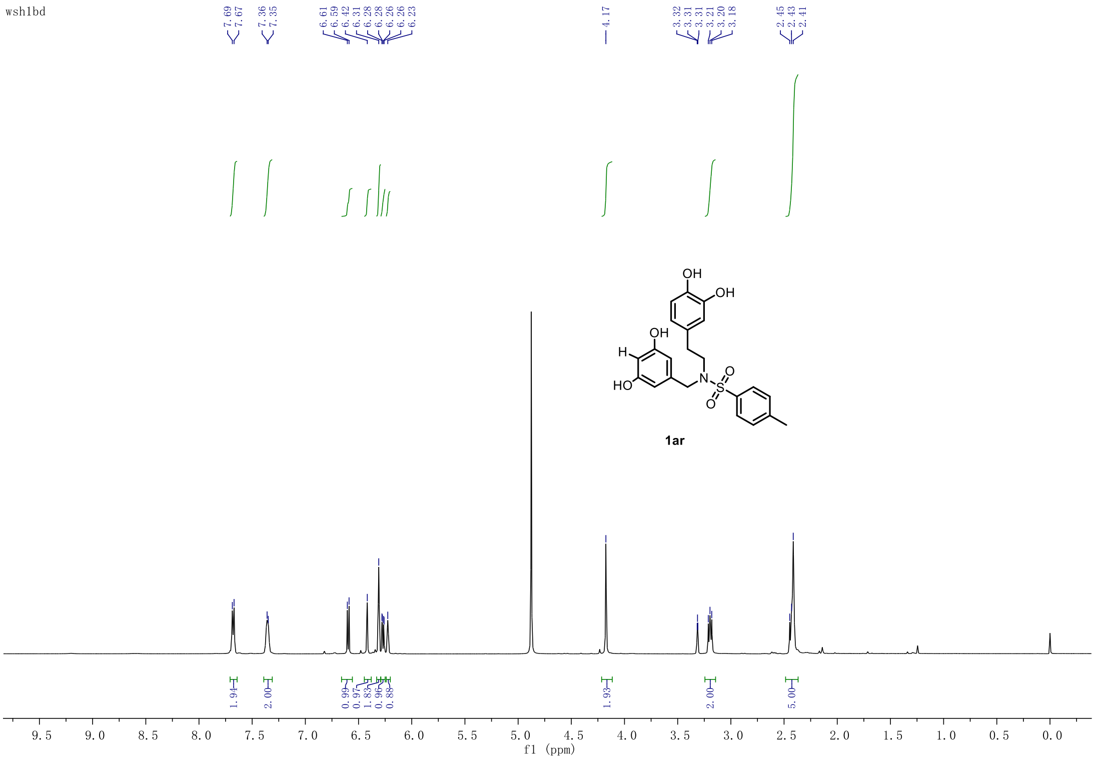

^{13}C NMR spectrum of compound 1ar (125 MHz) in CD_3OD

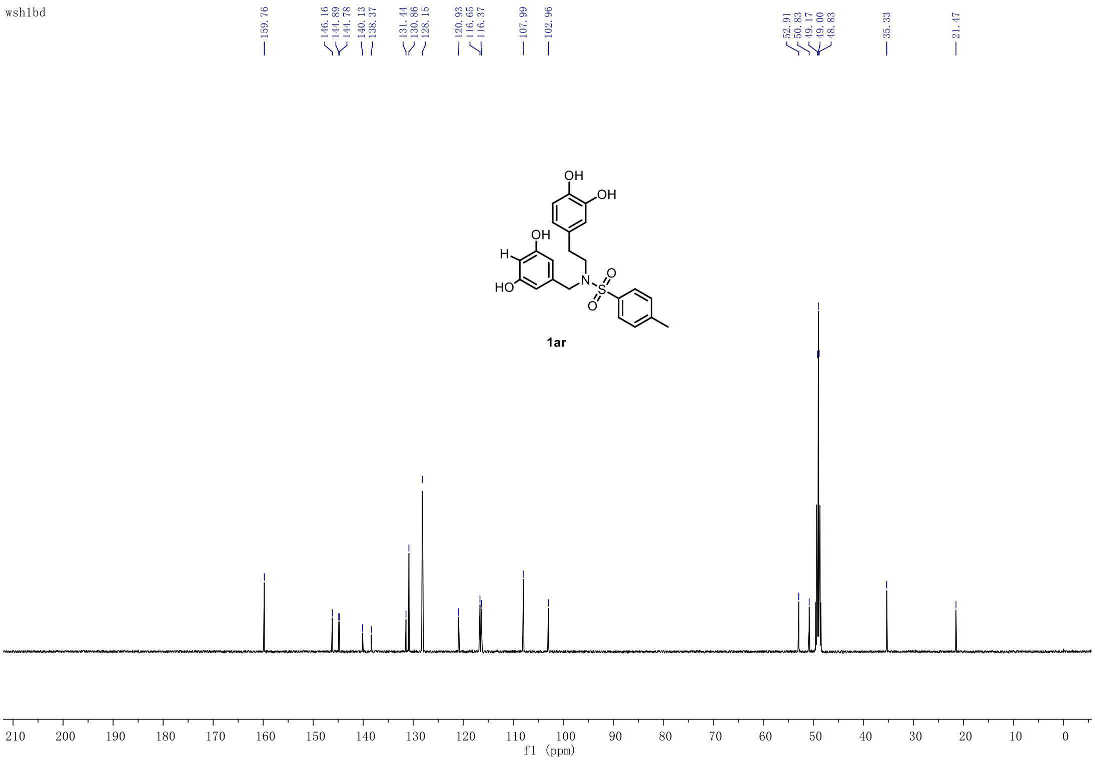

^{1}H NMR spectrum of compound 1as (500 MHz) in CD_3OD

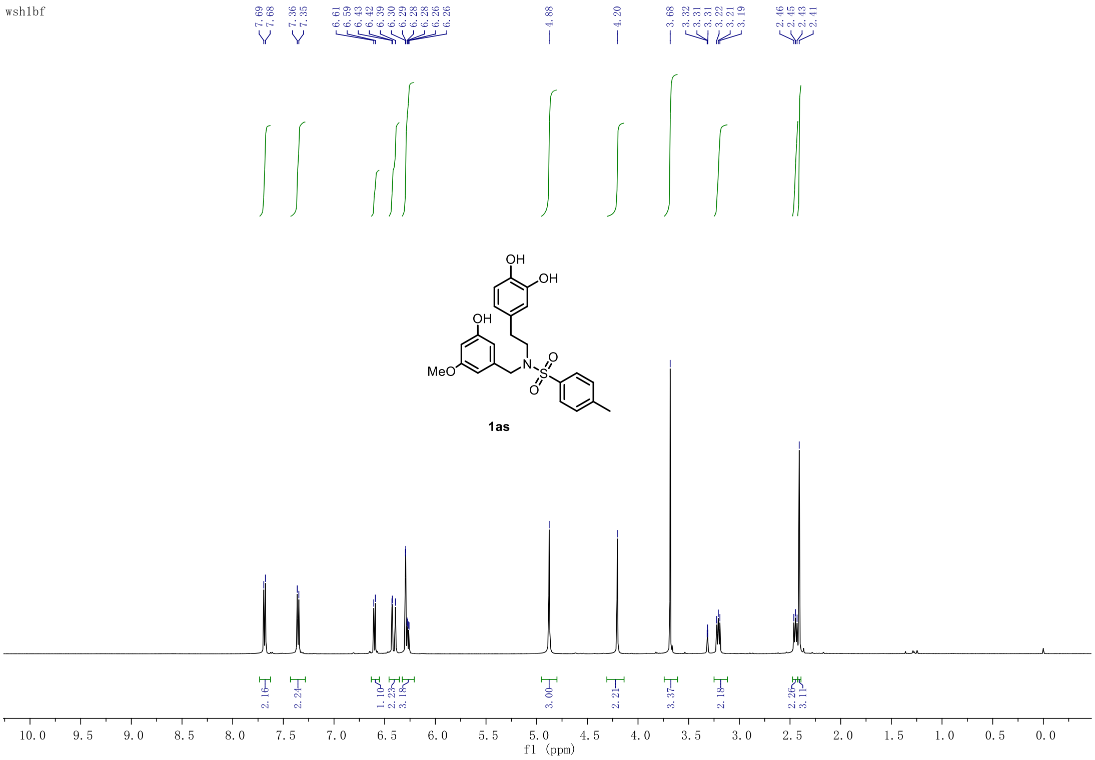

^{13}C NMR spectrum of compound 1as (125 MHz) in CD_3OD

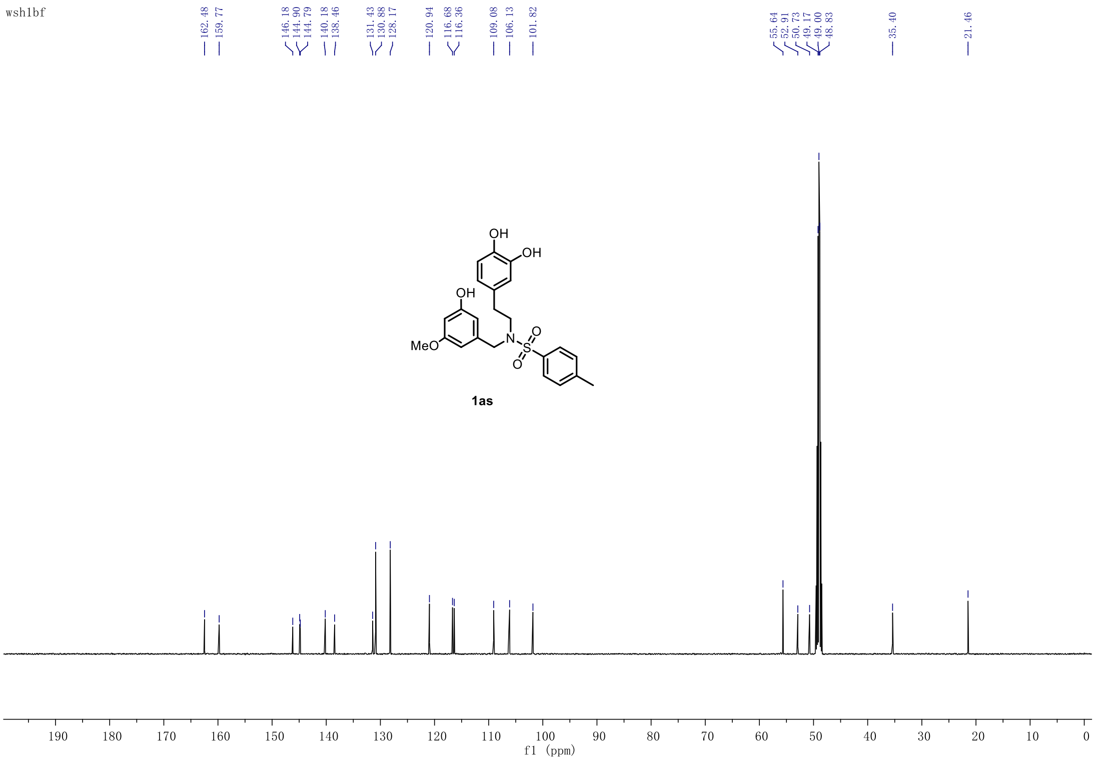

^{1}H NMR spectrum of compound 1at (500 MHz) in CD_3OD

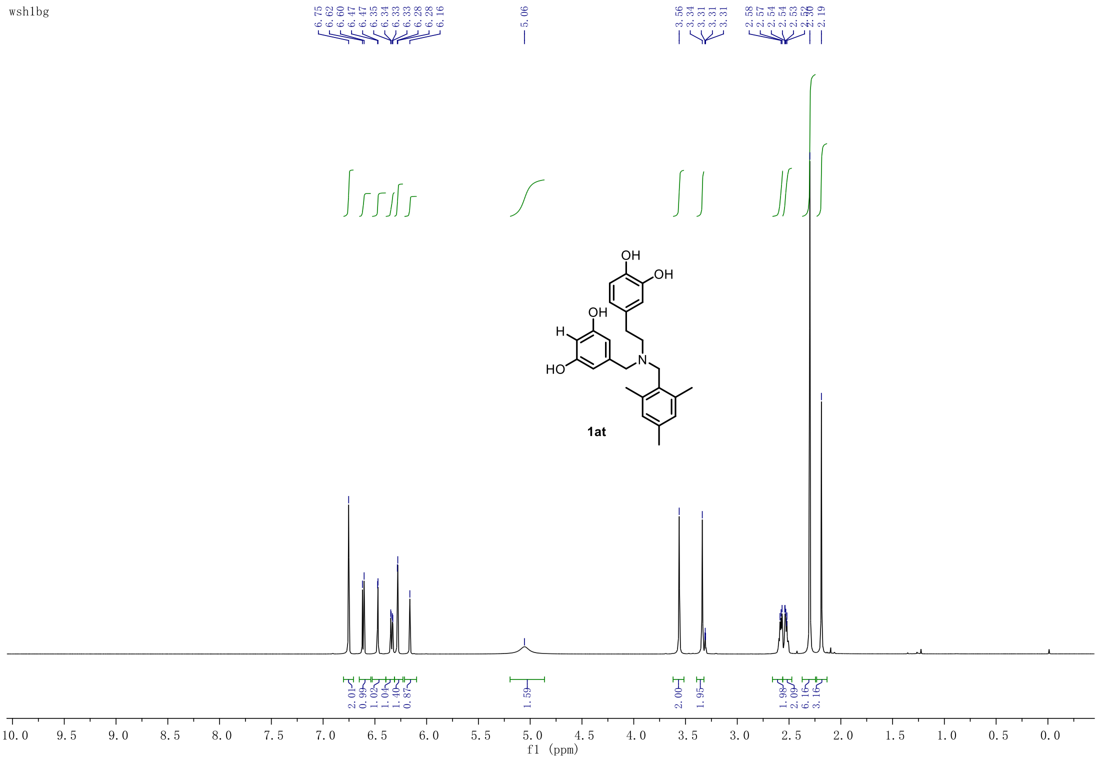

^{13}C NMR spectrum of compound 1at (125 MHz) in CD_3OD

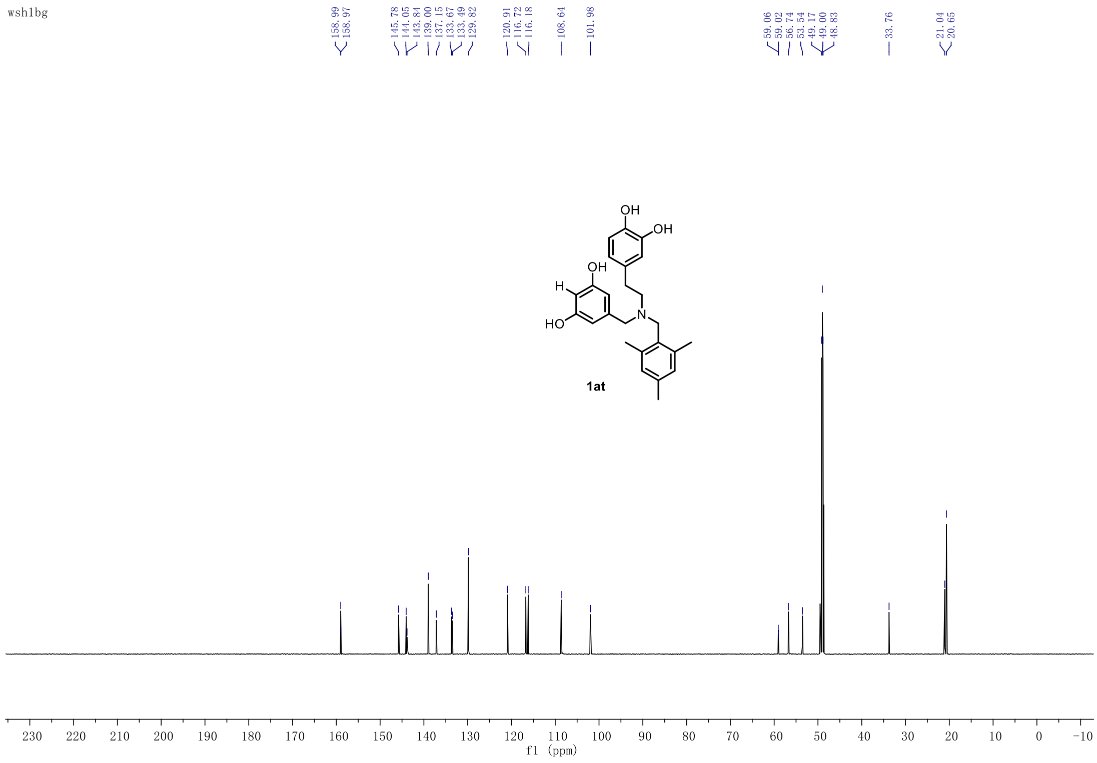

^{1}H NMR spectrum of compound 1au (400 MHz) in CD_3OD

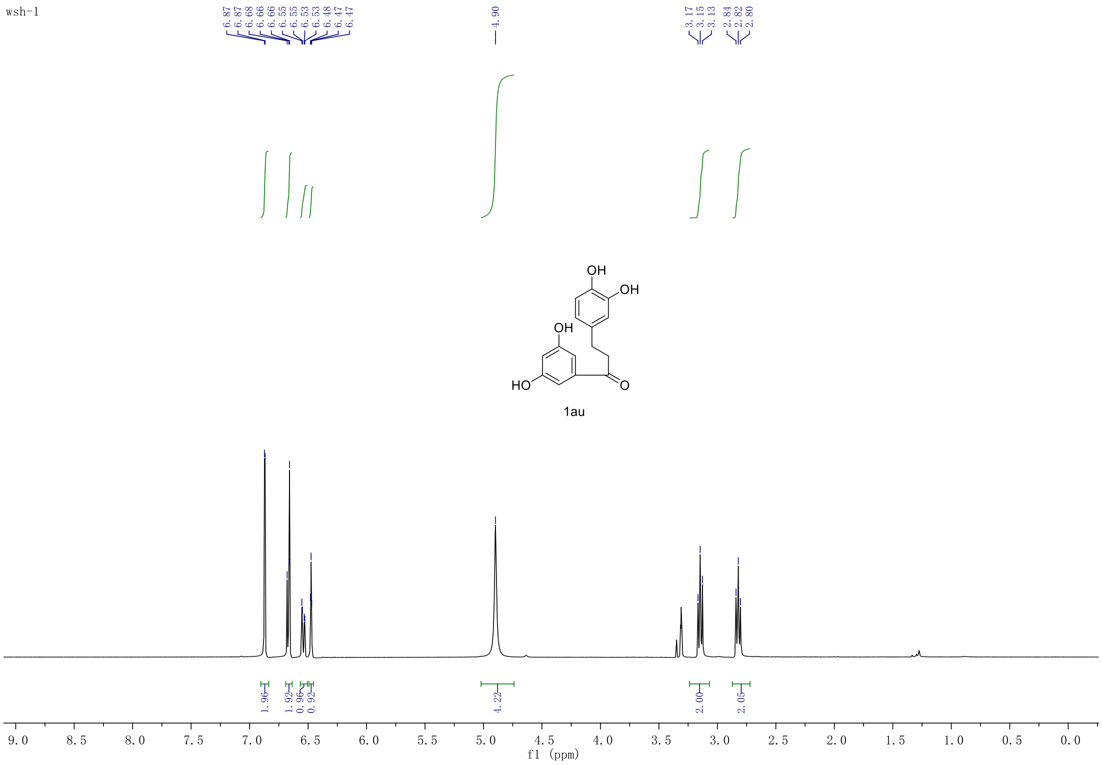

^{13}C NMR spectrum of compound 1au (100 MHz) in CD_3OD

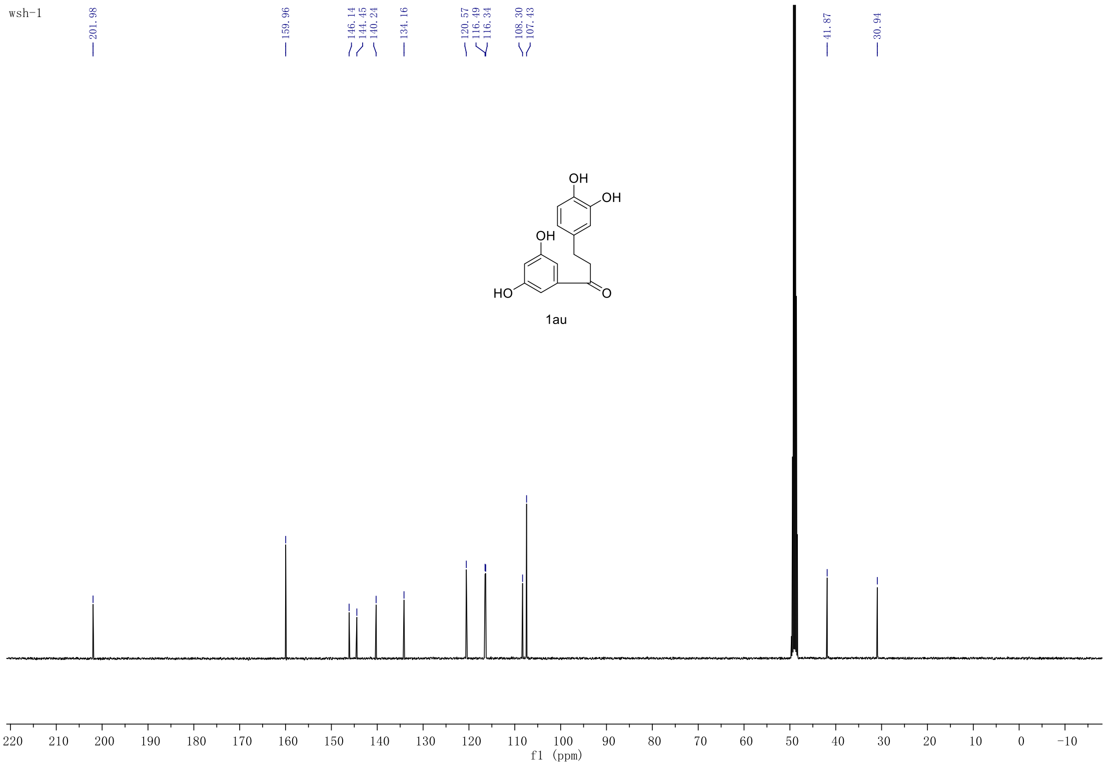

^{1}H NMR spectrum of compound 1av (400 MHz) in CD_3OD

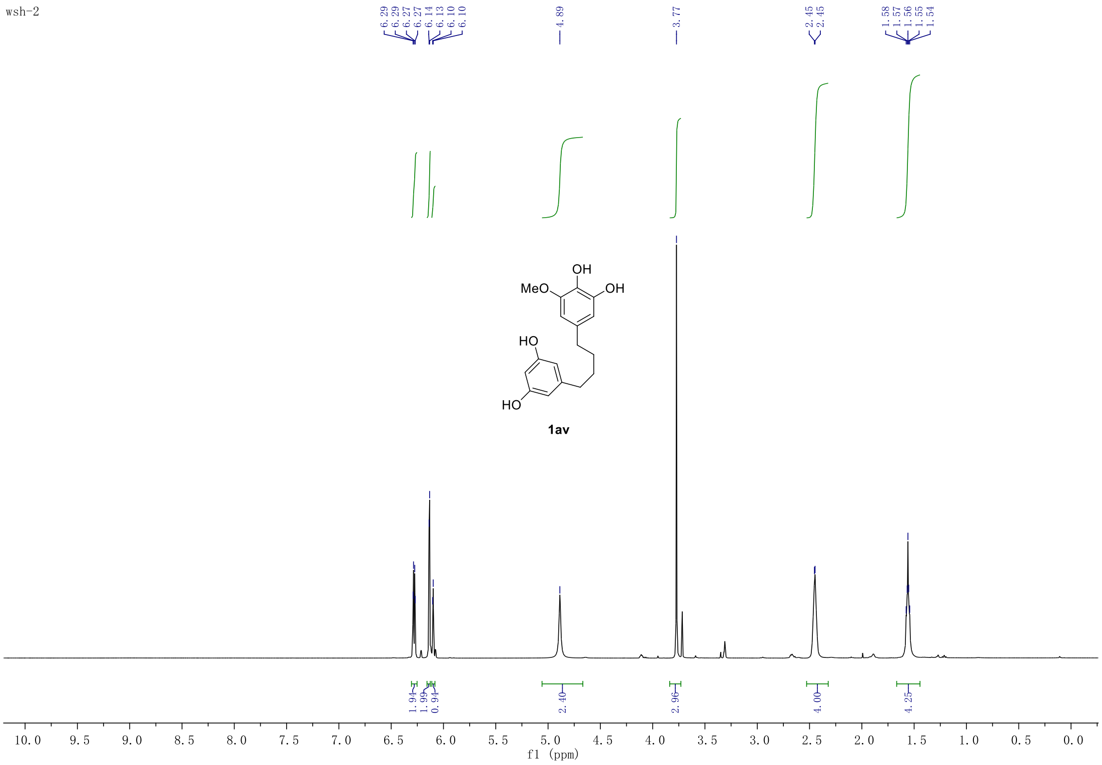

^{13}C NMR spectrum of compound 1av (100 MHz) in CD_3OD

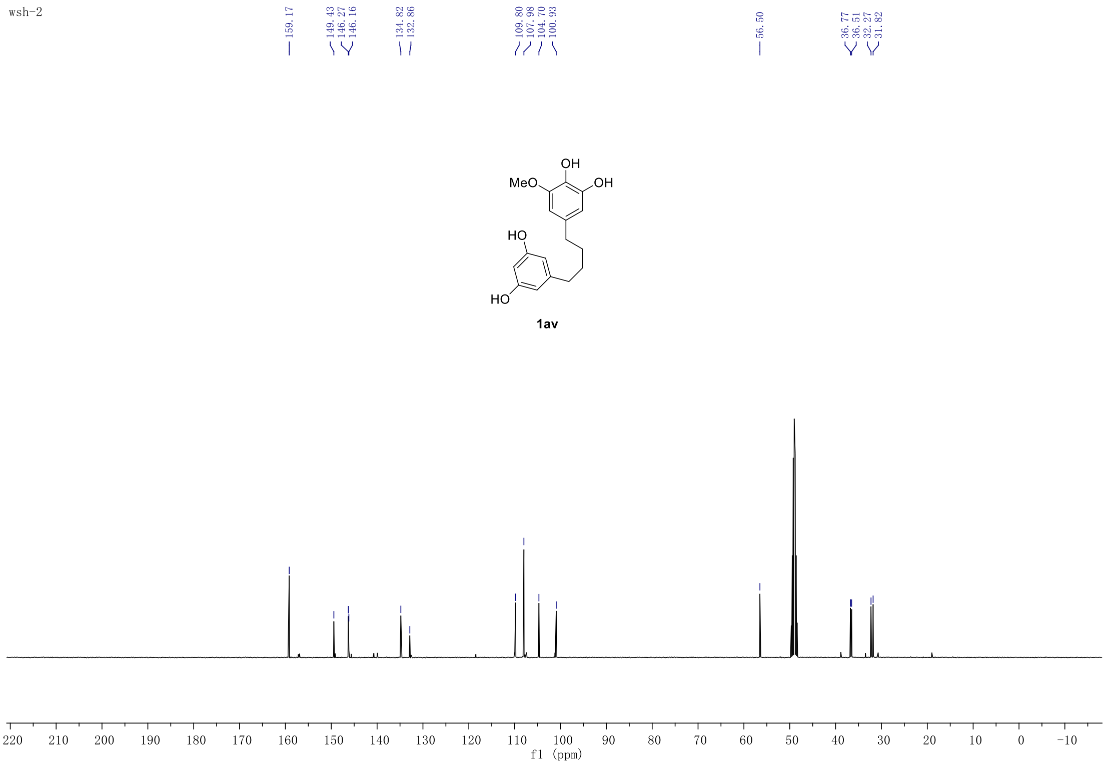

^{1}H NMR spectrum of compound 1aw (400 MHz) in CD_3OD

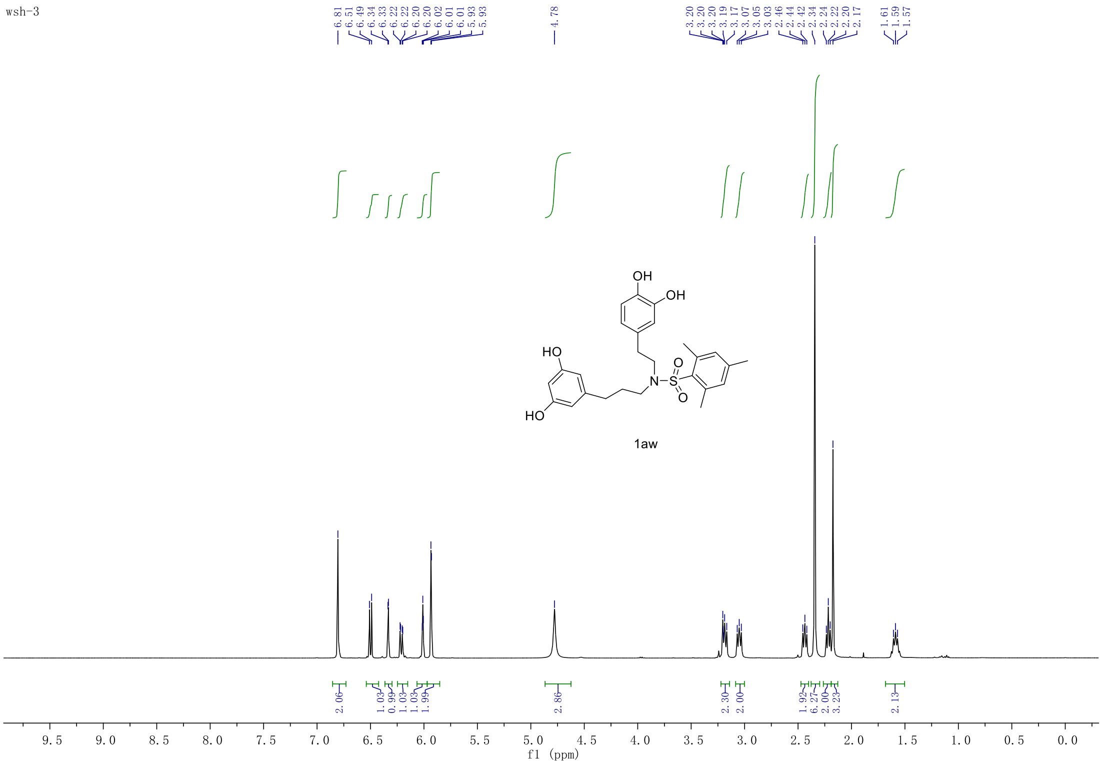

^{13}C NMR spectrum of compound 1aw (100 MHz) in CD_3OD

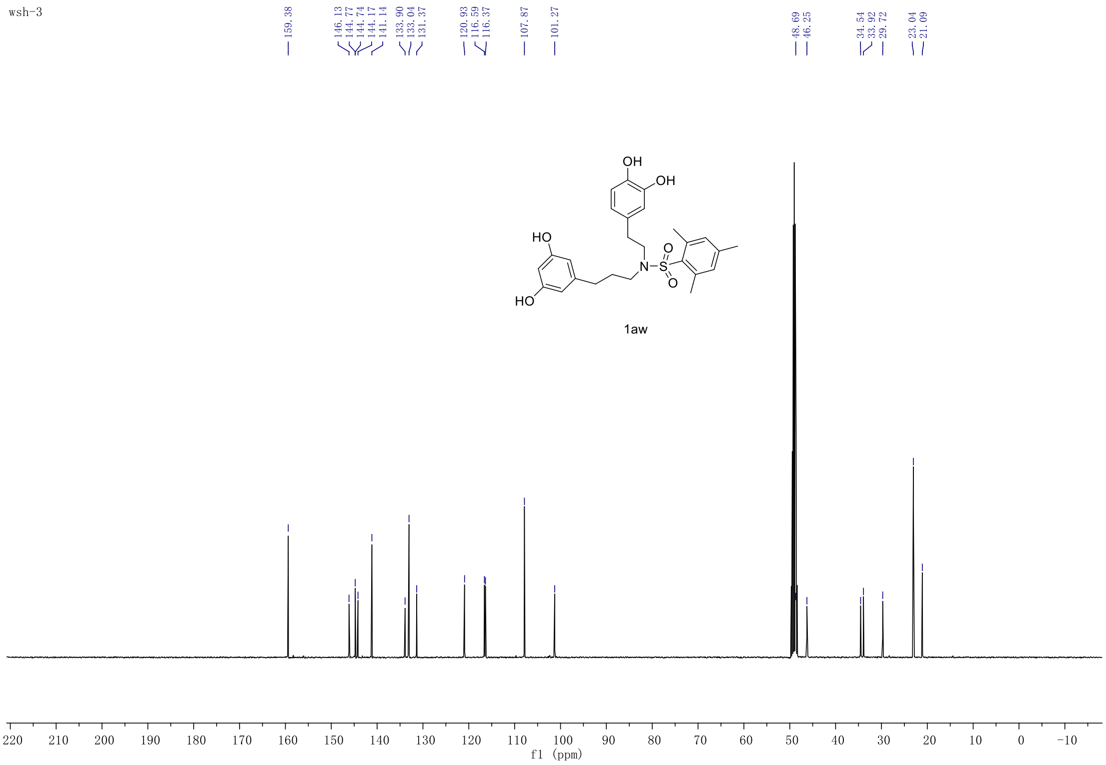

^{1}H NMR spectrum of compound 1ax (400 MHz) in CD_3OD

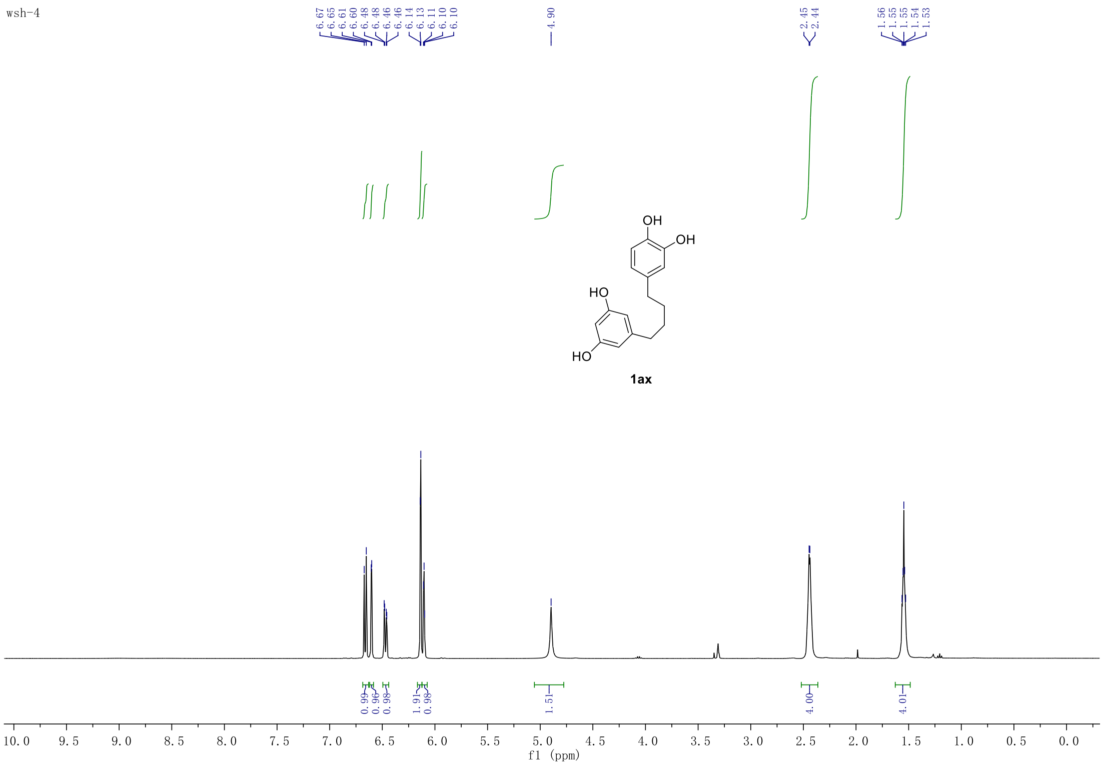

^{13}C NMR spectrum of compound 1ax (100 MHz) in CD_3OD

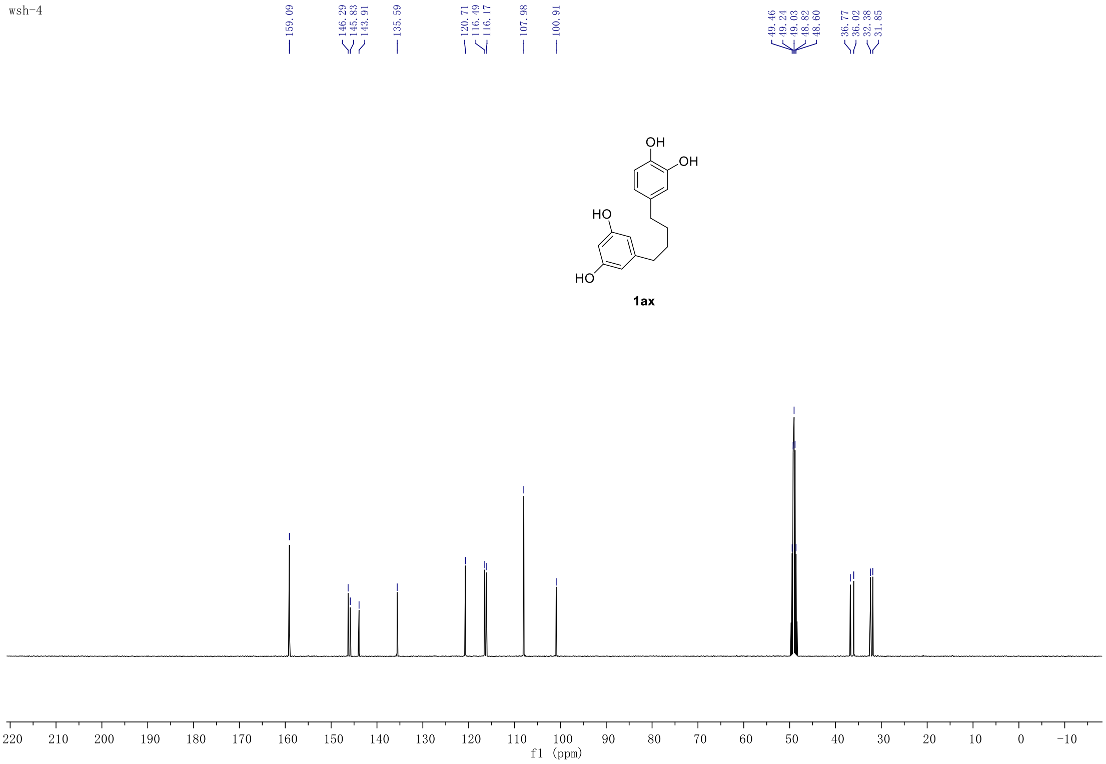

^{1}H NMR spectrum of compound 1az (400 MHz) in CD_3OD

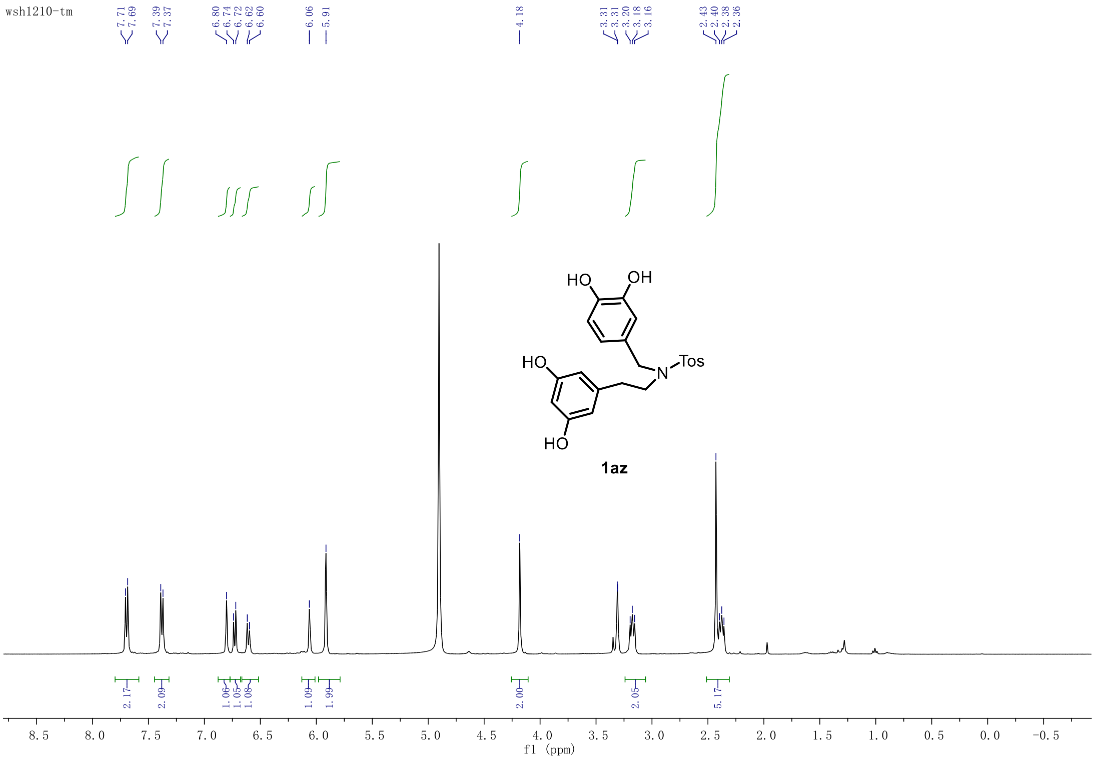

^{13}C NMR spectrum of compound 1az (100 MHz) in CD_3OD

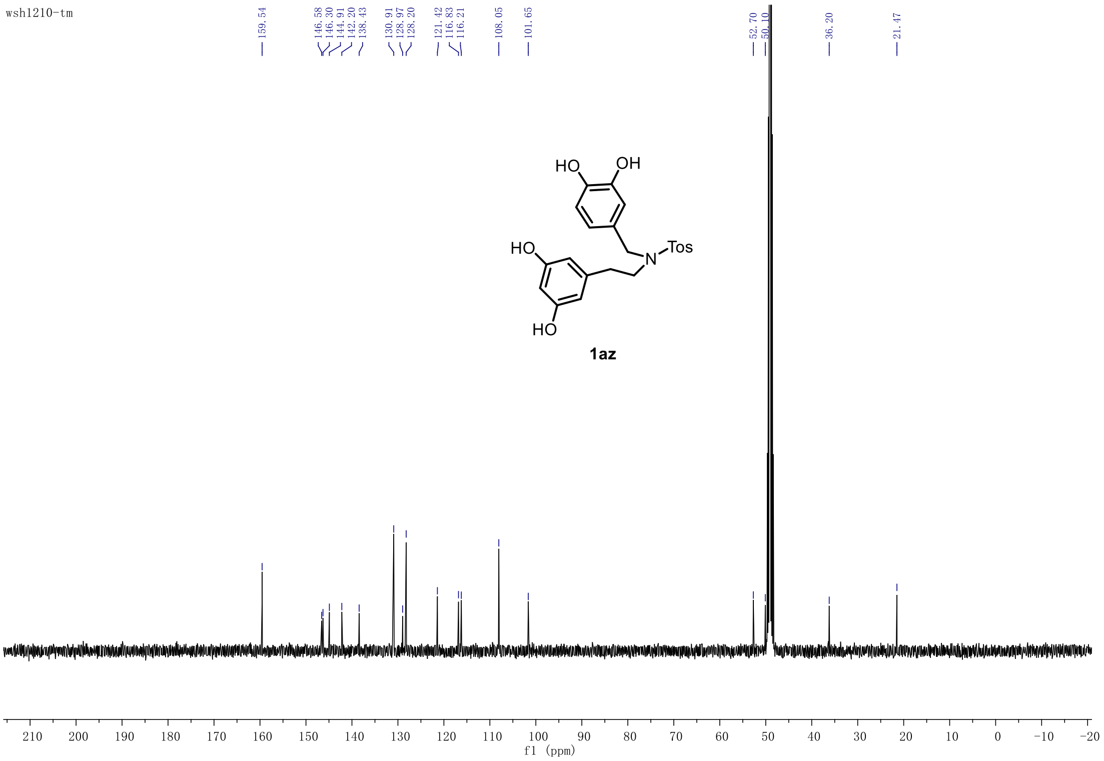

^{1}H NMR spectrum of compound 1ba (400 MHz) in CD_3Cl

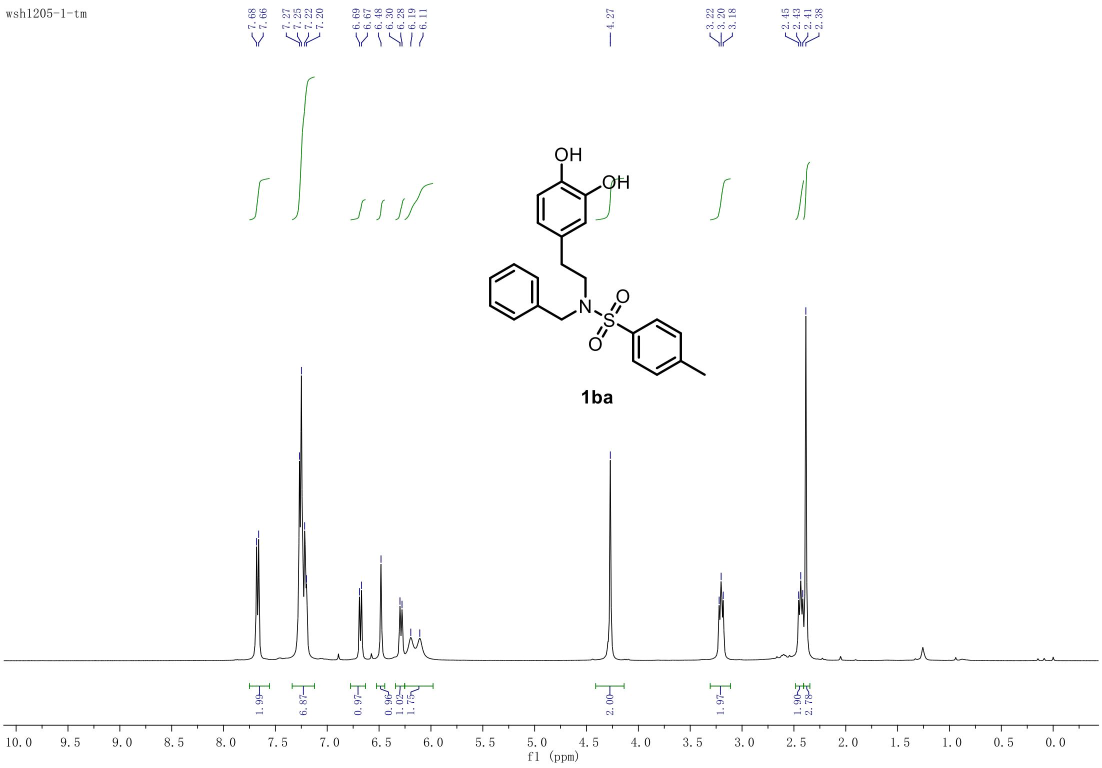

^{13}C NMR spectrum of compound 1ba (100 MHz) in CD_3Cl

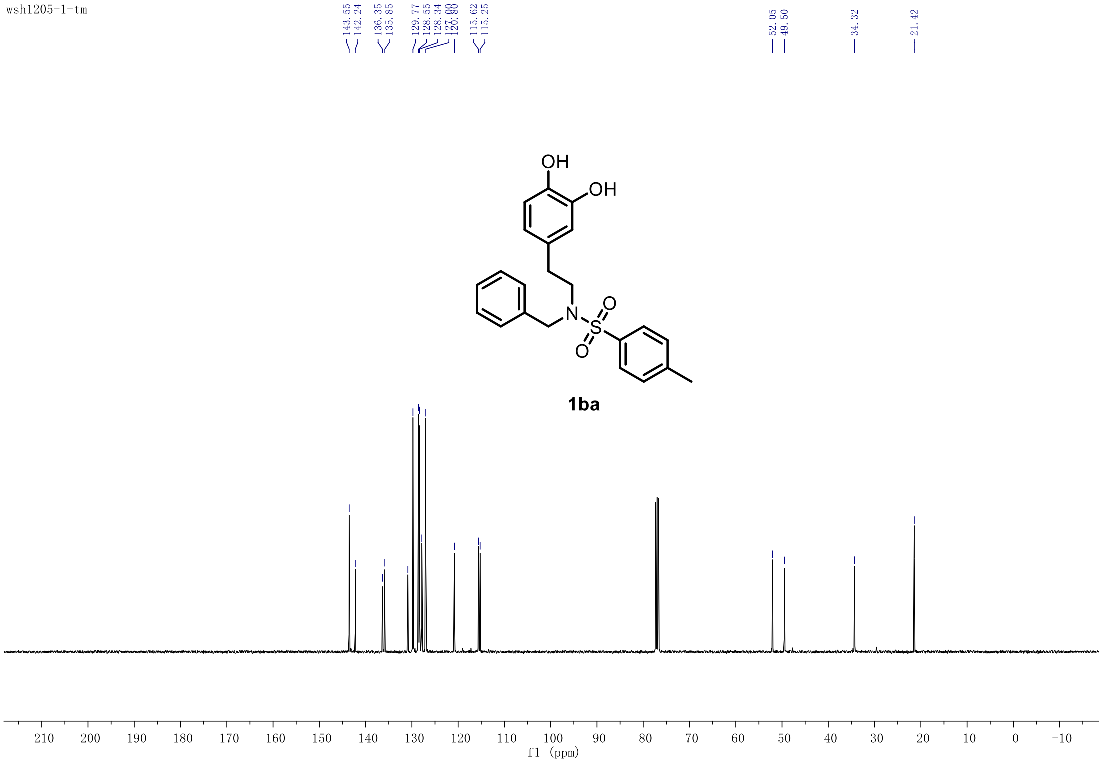

^{1}H NMR spectrum of compound 2a (500 MHz) in CD_3OD

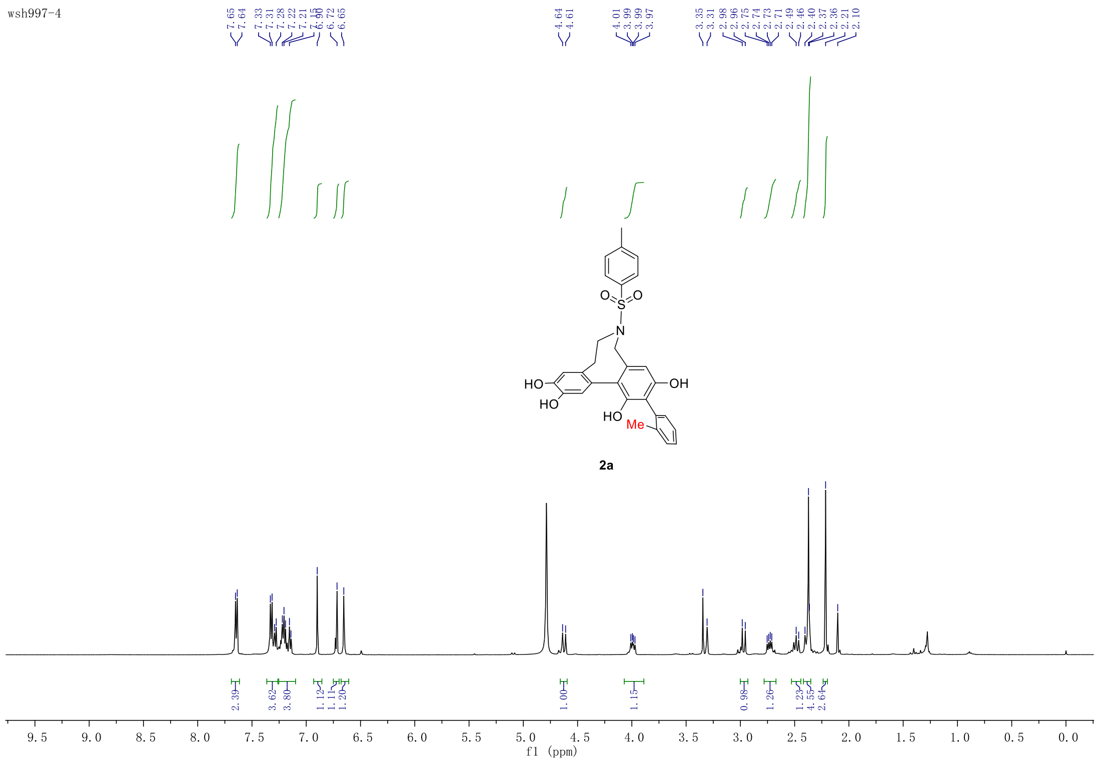

^{13}C NMR spectrum of compound 2a (125 MHz) in CD_3OD

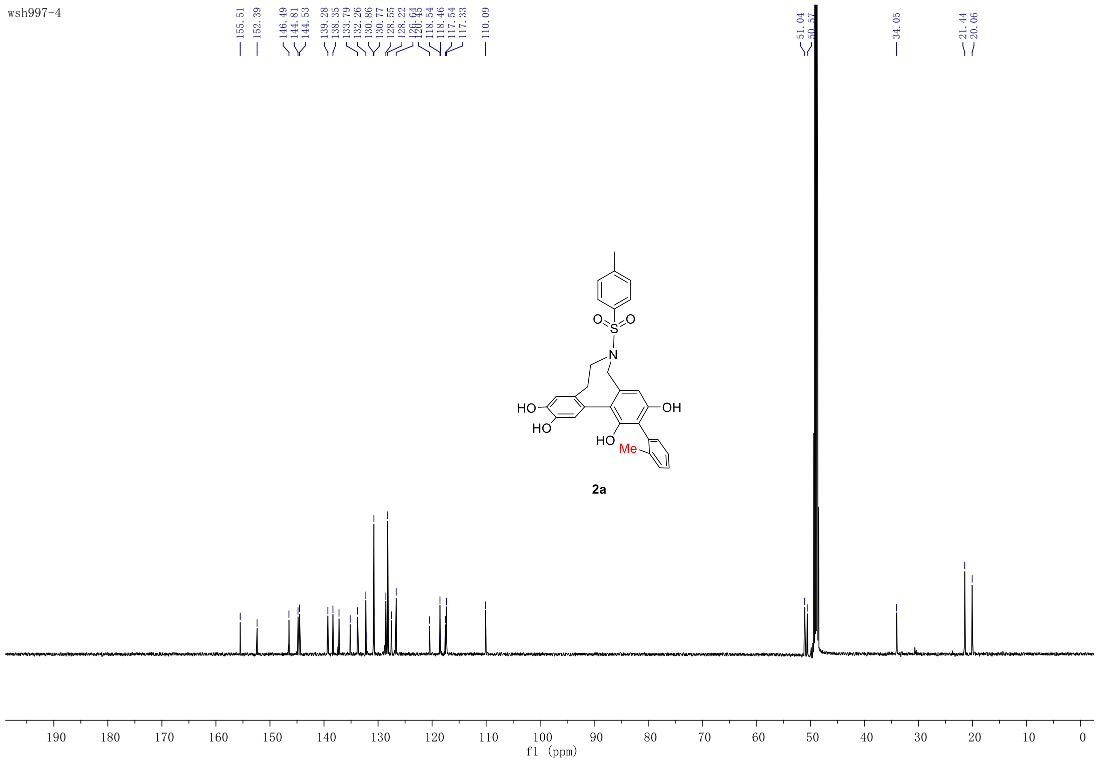

^{1}H NMR spectrum of compound 2b (500 MHz) in CD_3OD

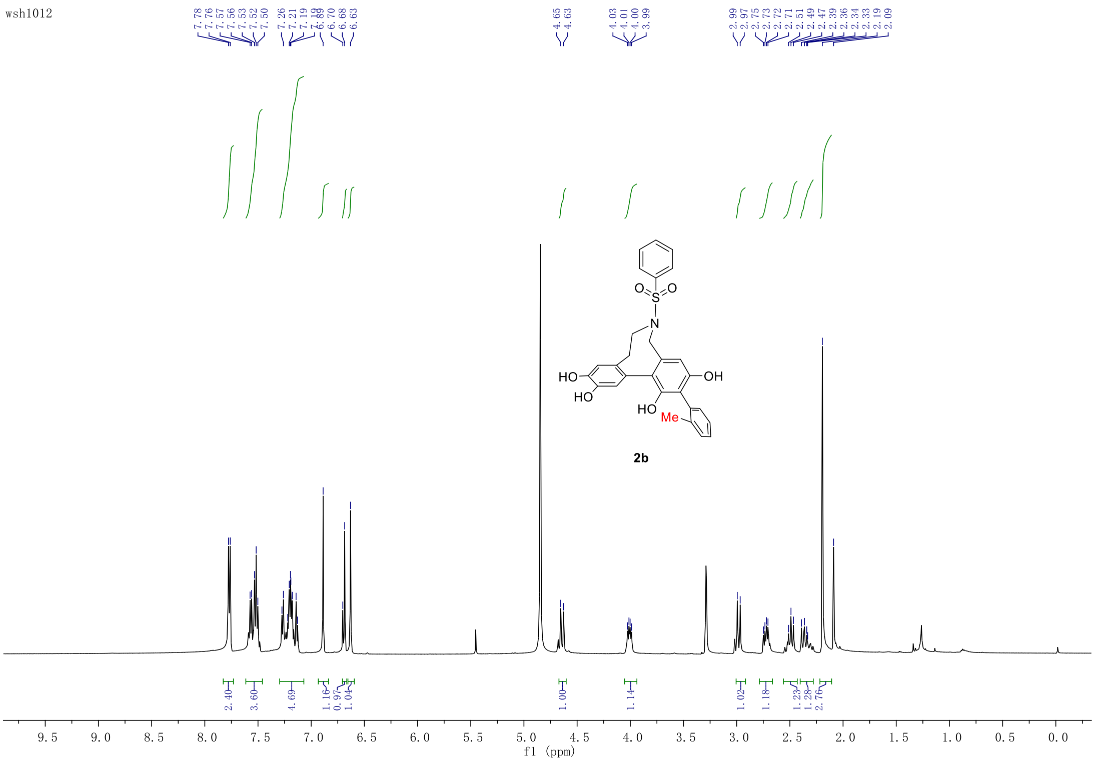

^{13}C NMR spectrum of compound 2b (125 MHz) in CD_3OD

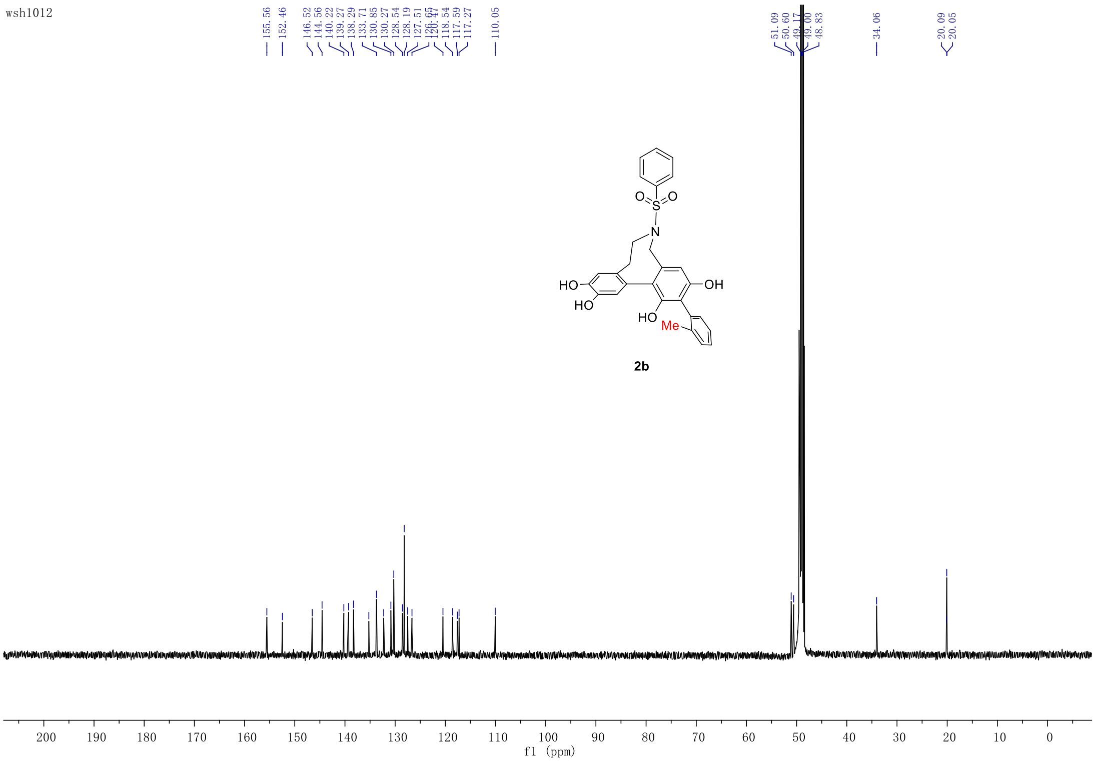

^{1}H NMR spectrum of compound 2c (500 MHz) in CD_3OD

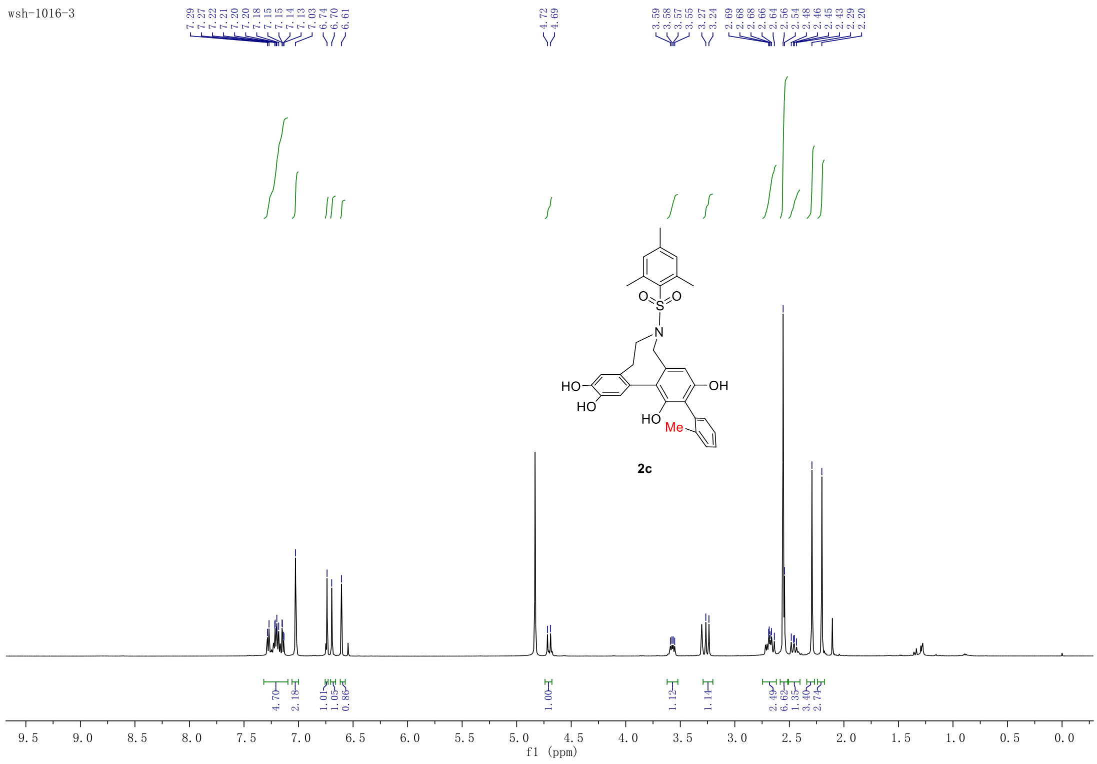

^{13}C NMR spectrum of compound 2c (125 MHz) in CD_3OD

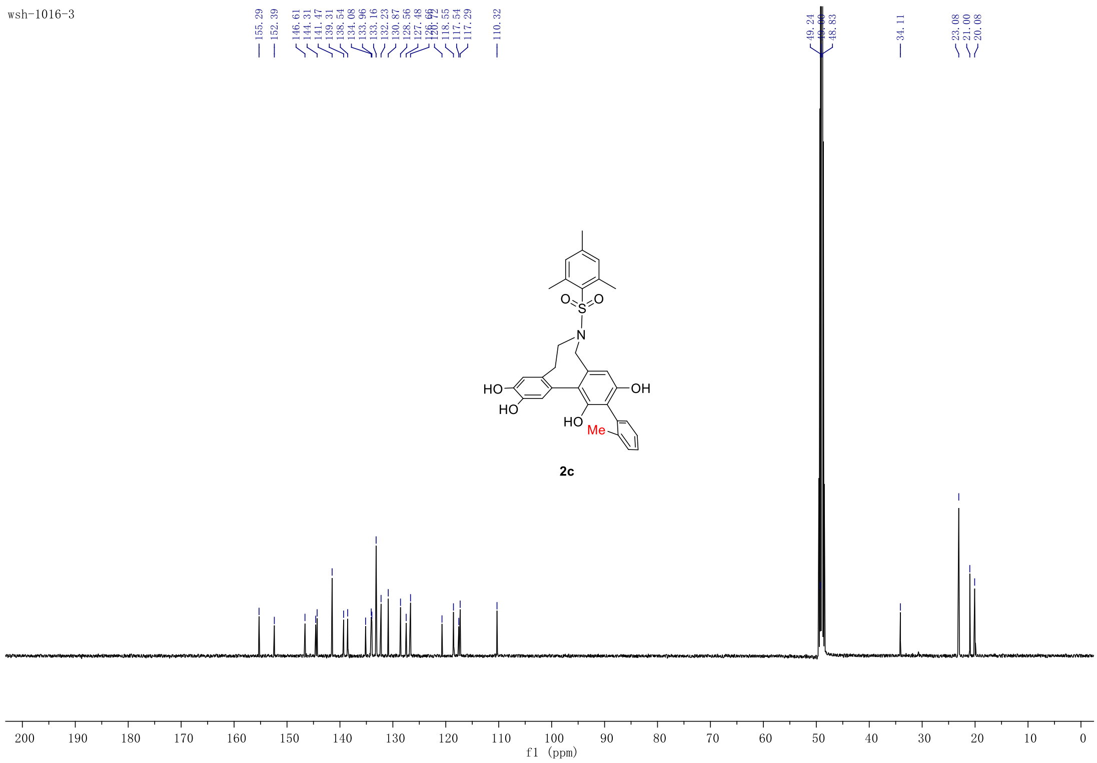

^{1}H NMR spectrum of compound 2d (500 MHz) in CD_3OD

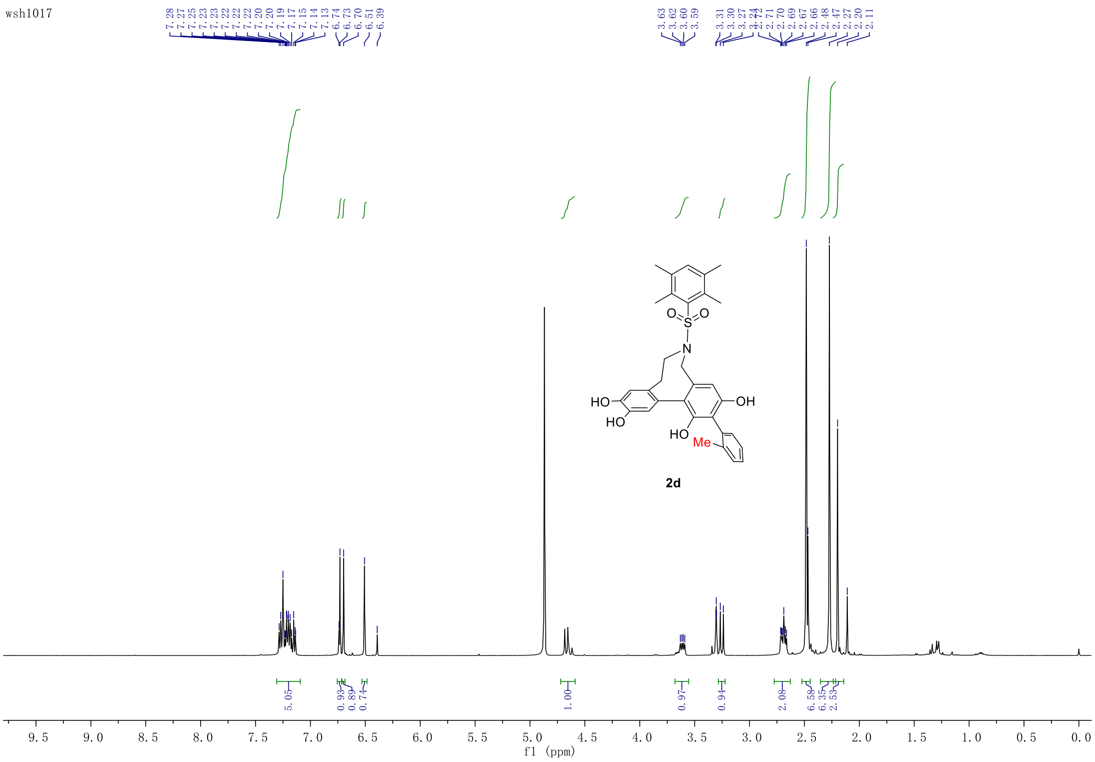

^{13}C NMR spectrum of compound 2d (125 MHz) in CD_3OD

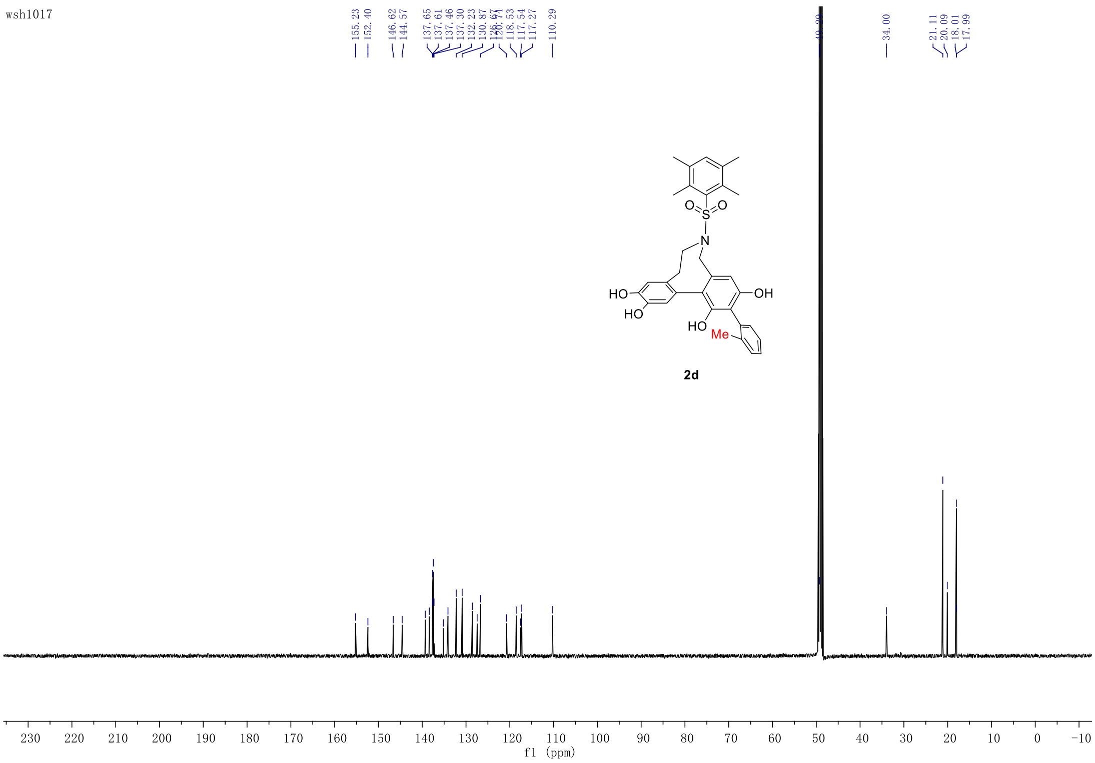

^{1}H NMR spectrum of compound 2e (500 MHz) in CD_3OD

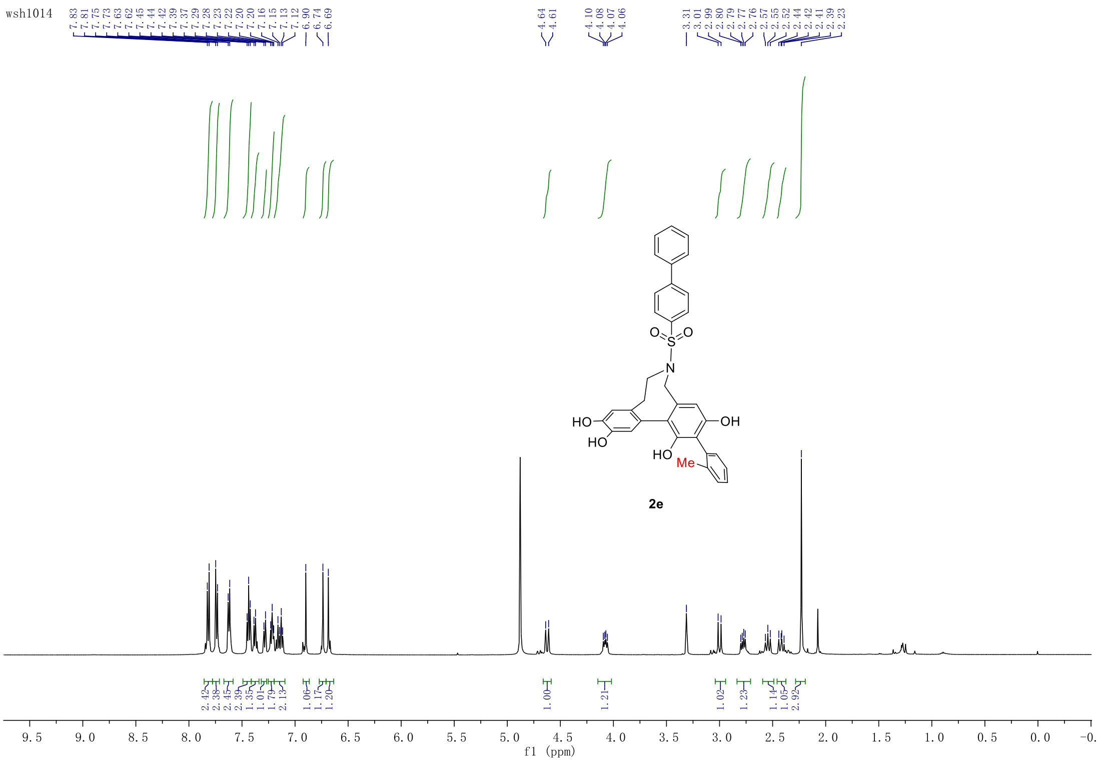

^{13}C NMR spectrum of compound 2e (125 MHz) in CD_3OD

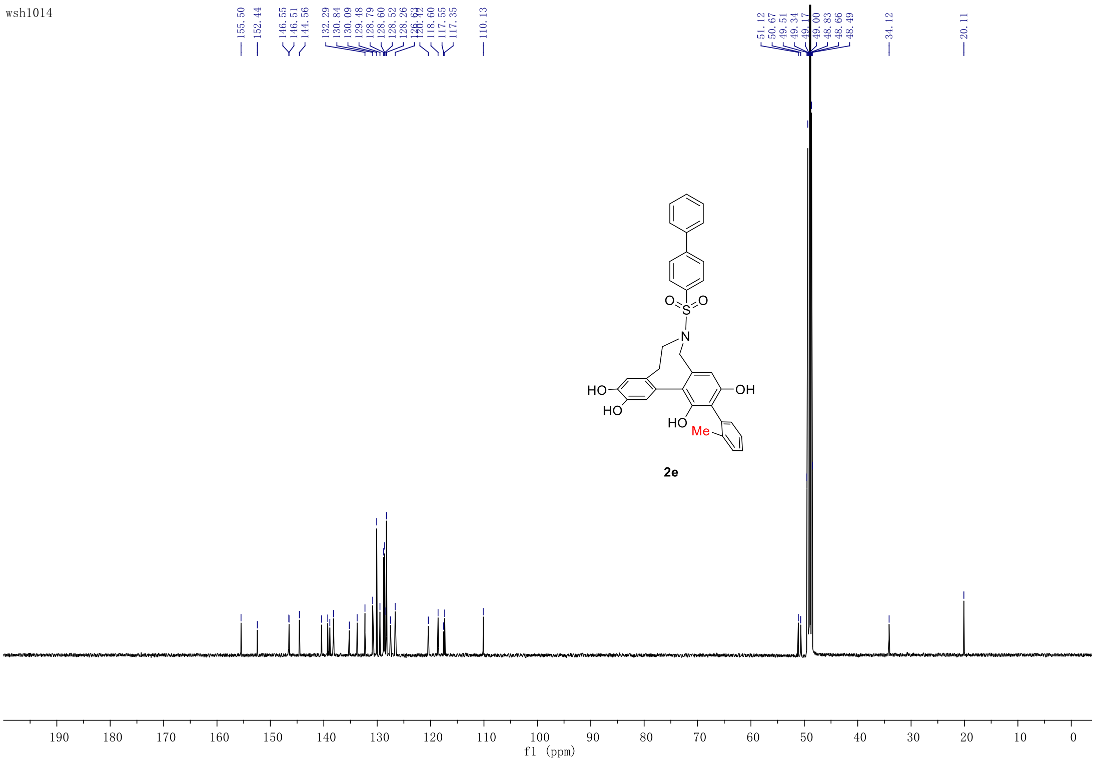

^{1}H NMR spectrum of compound 2f (500 MHz) in CD_3OD

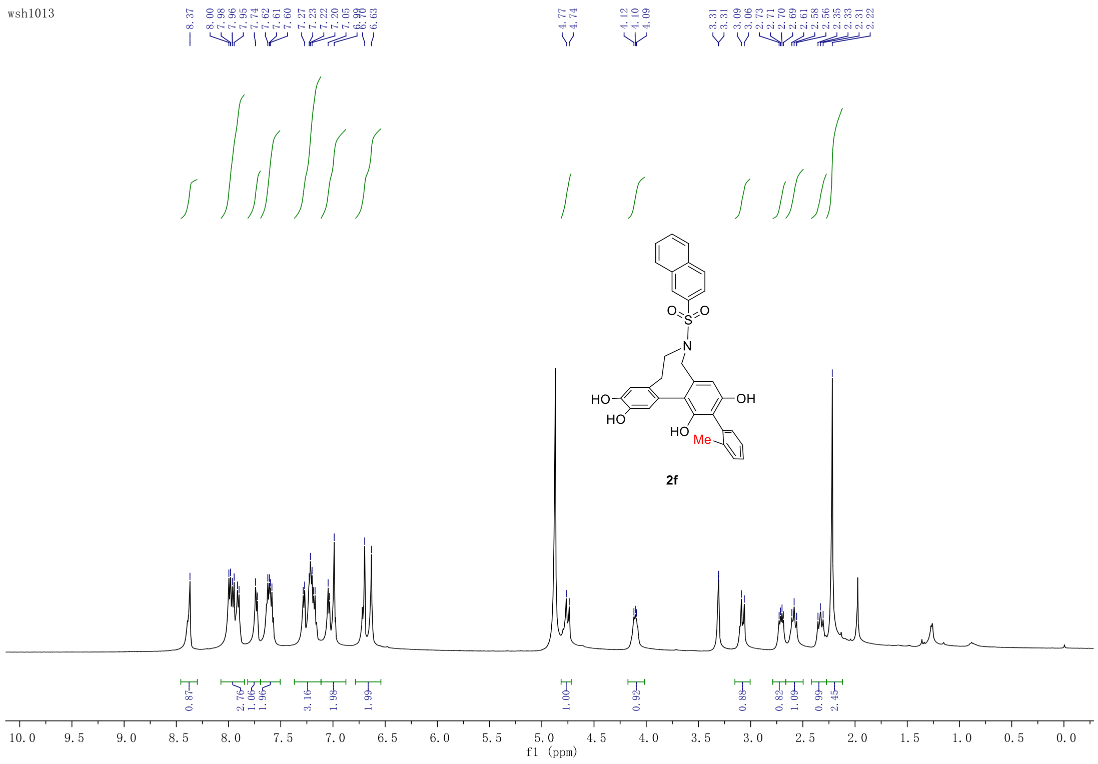

^{13}C NMR spectrum of compound 2f (125 MHz) in CD_3OD

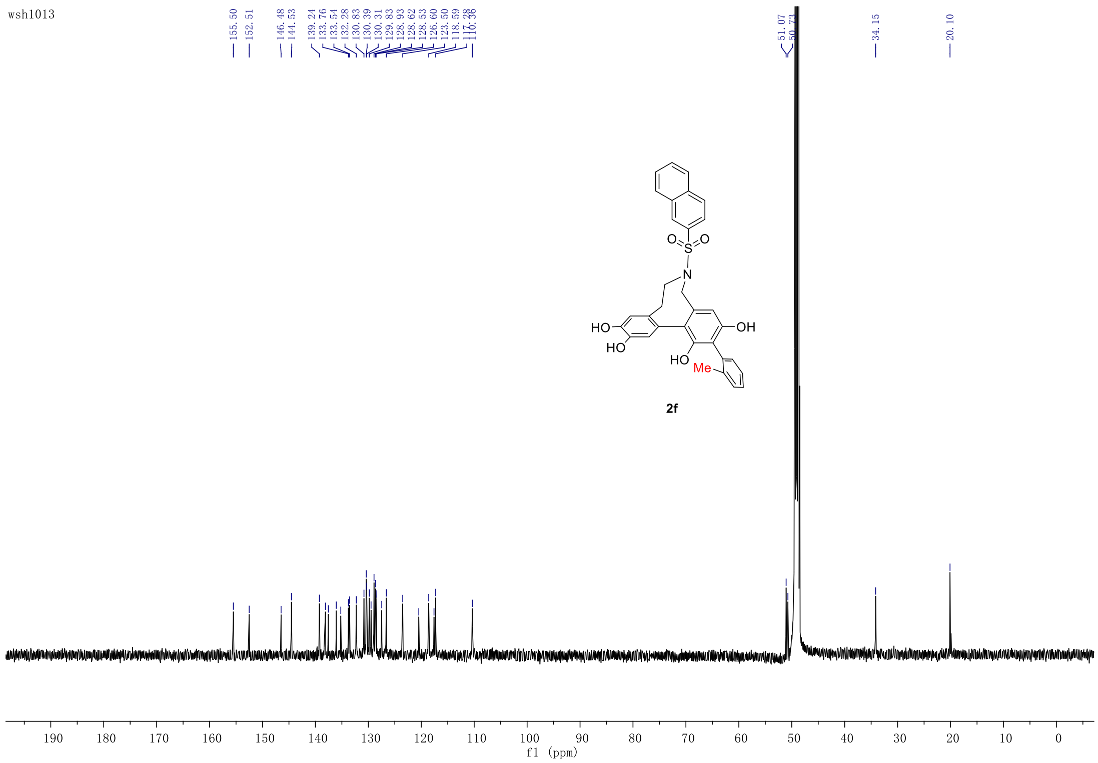

^{1}H NMR spectrum of compound 2g (500 MHz) in Acetone-d_6

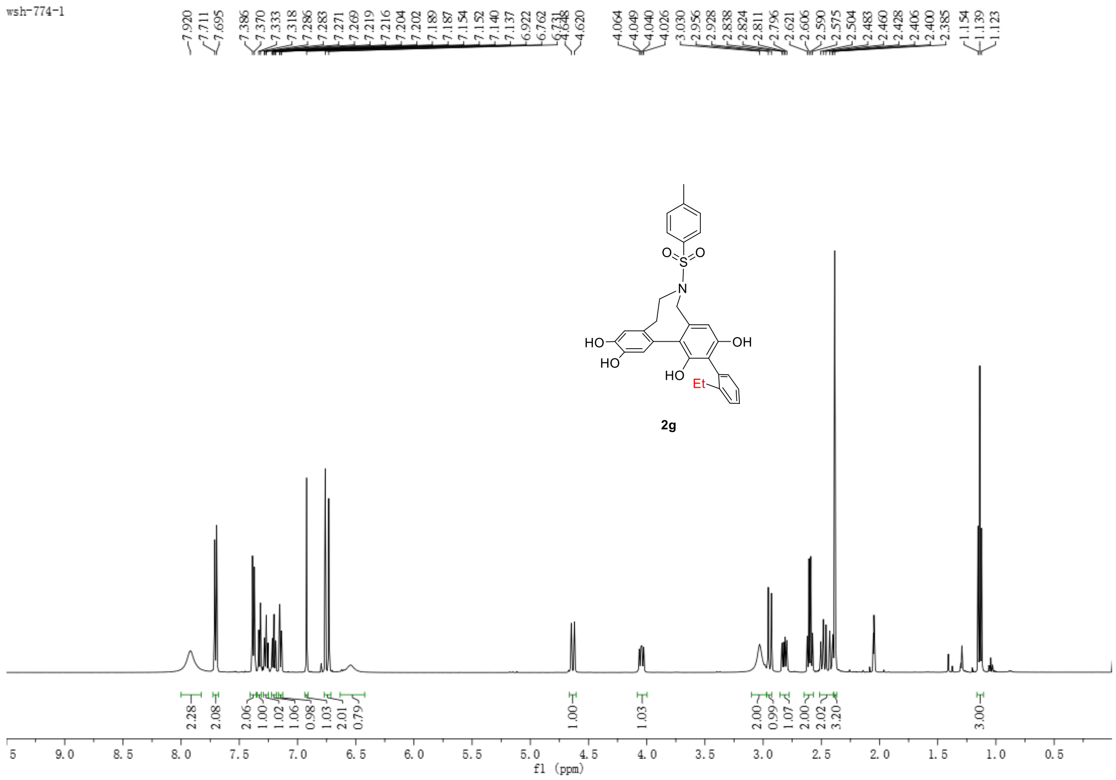

^{13}C NMR spectrum of compound 2g (125 MHz) in Acetone-d_6

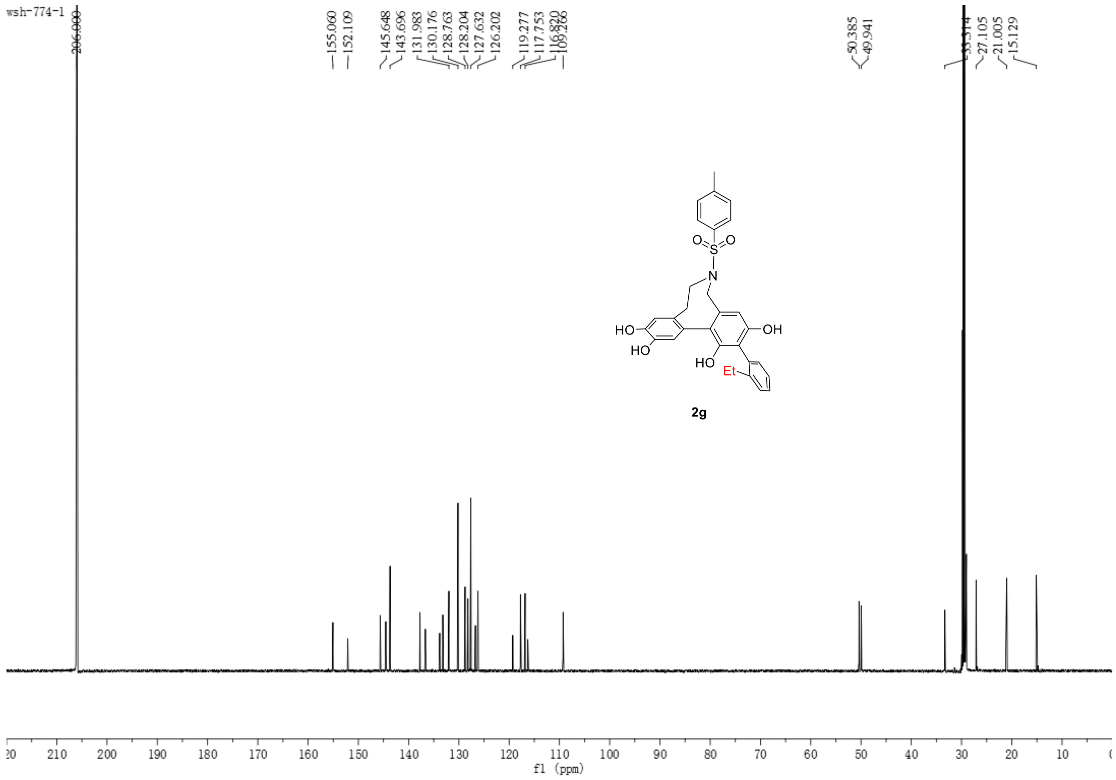

^{1}H NMR spectrum of compound 2h (500 MHz) in Acetone-d_6

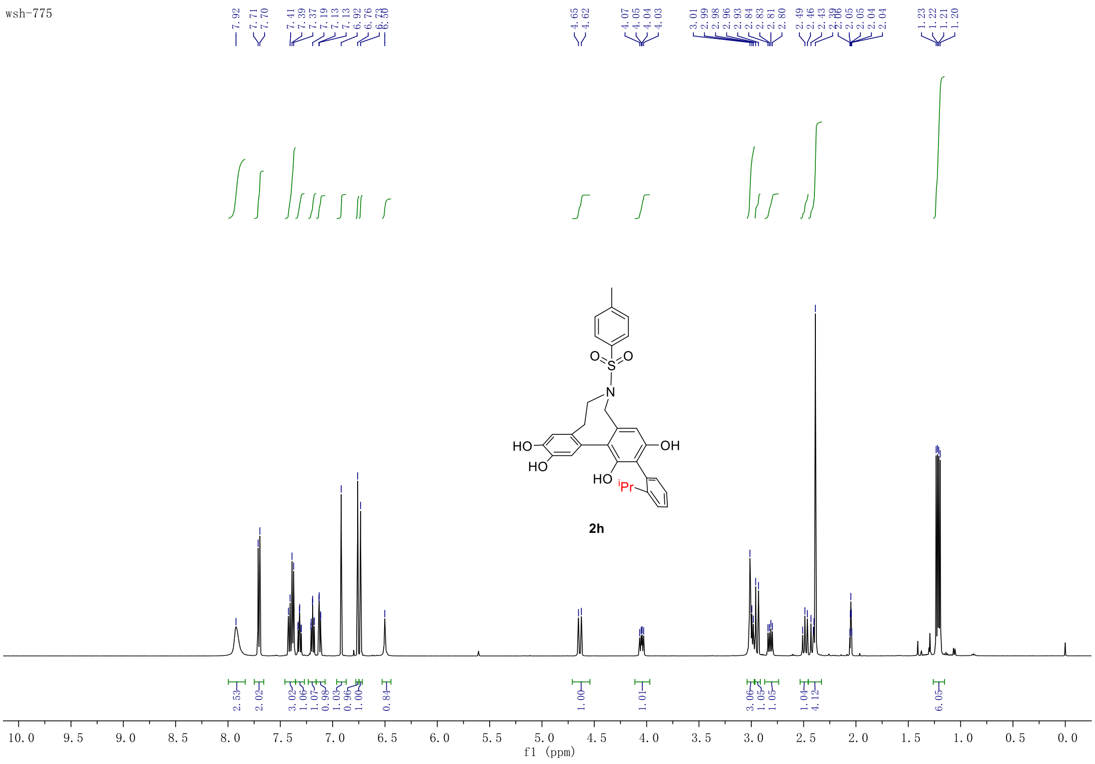

^{13}C NMR spectrum of compound 2h (125 MHz) in Acetone-d_6

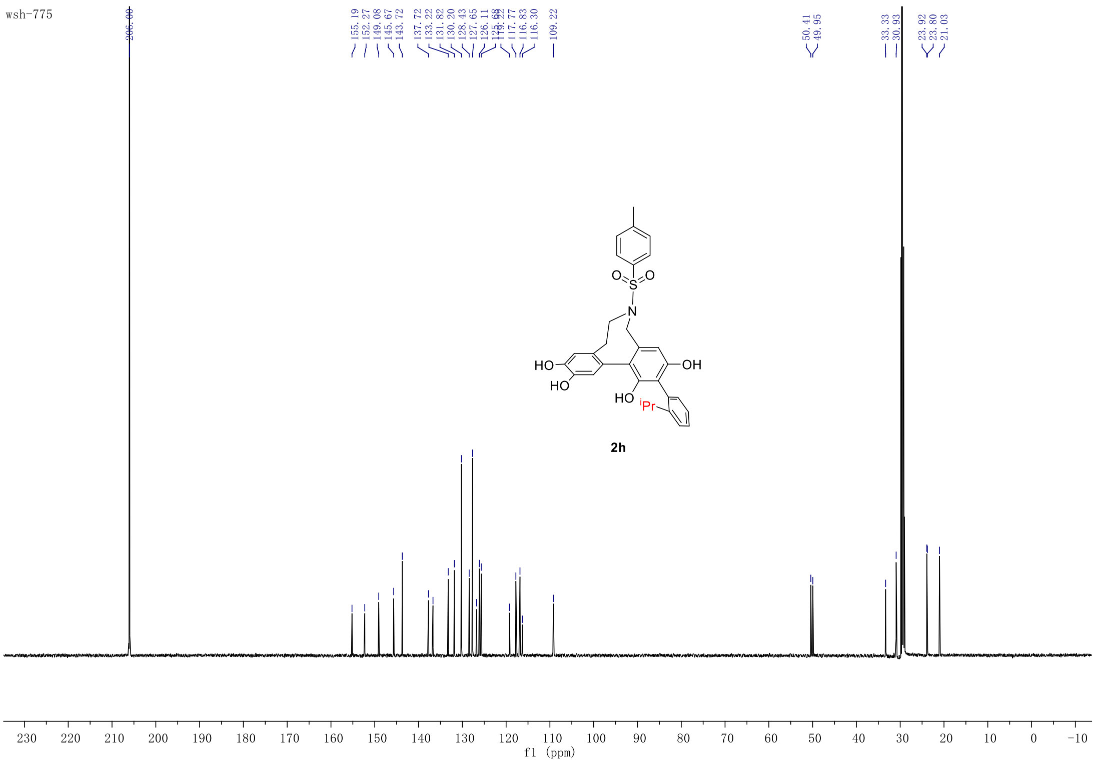

^{1}H NMR spectrum of compound 2i (500 MHz) in CD_3OD

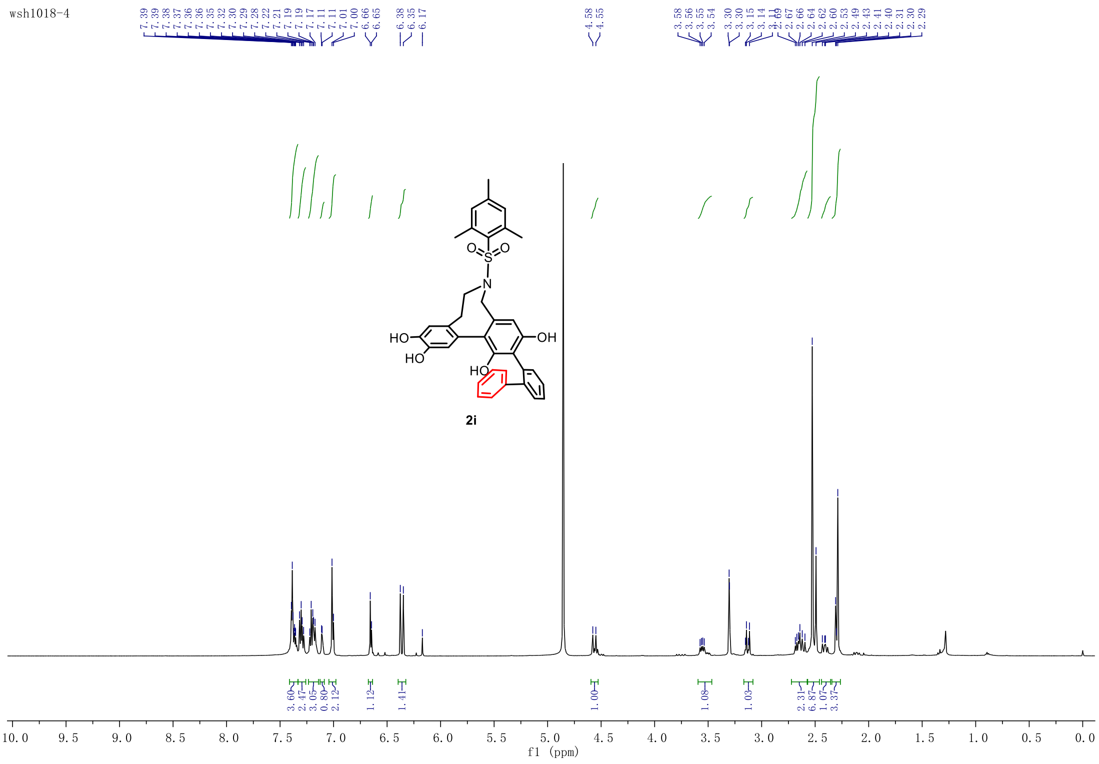

^{13}C NMR spectrum of compound 2i (125 MHz) in CD_3OD

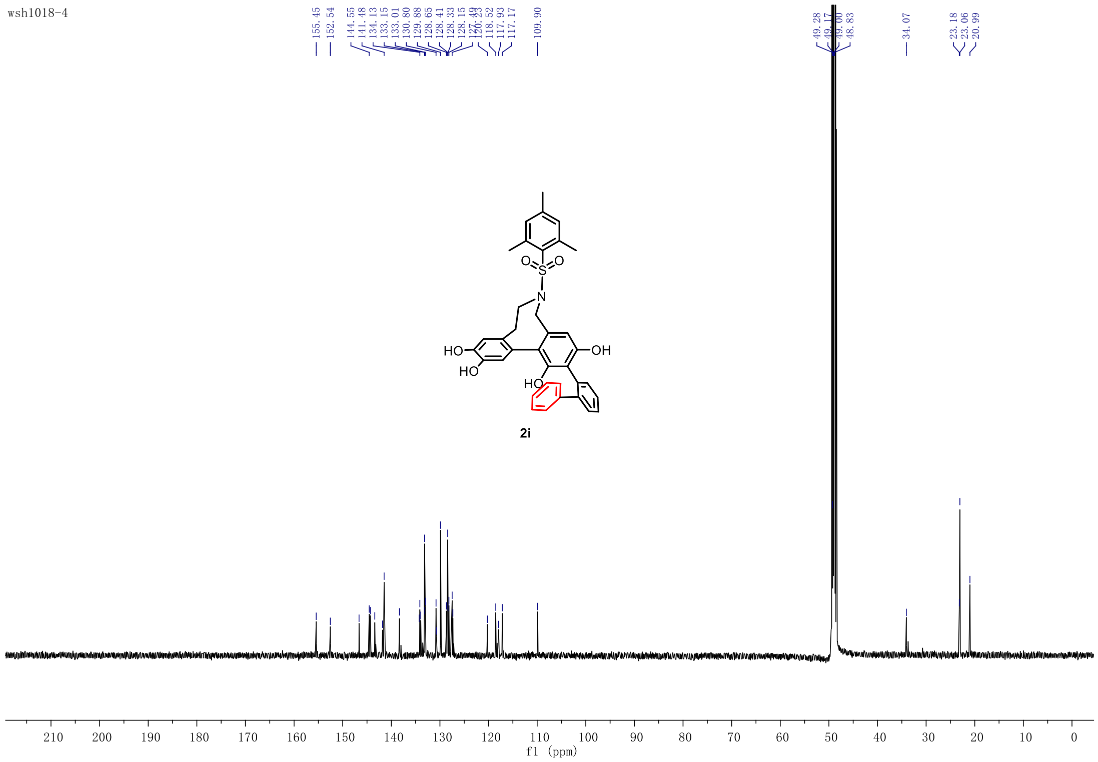

^{1}H NMR spectrum of compound 2j (500 MHz) in CD_3OD

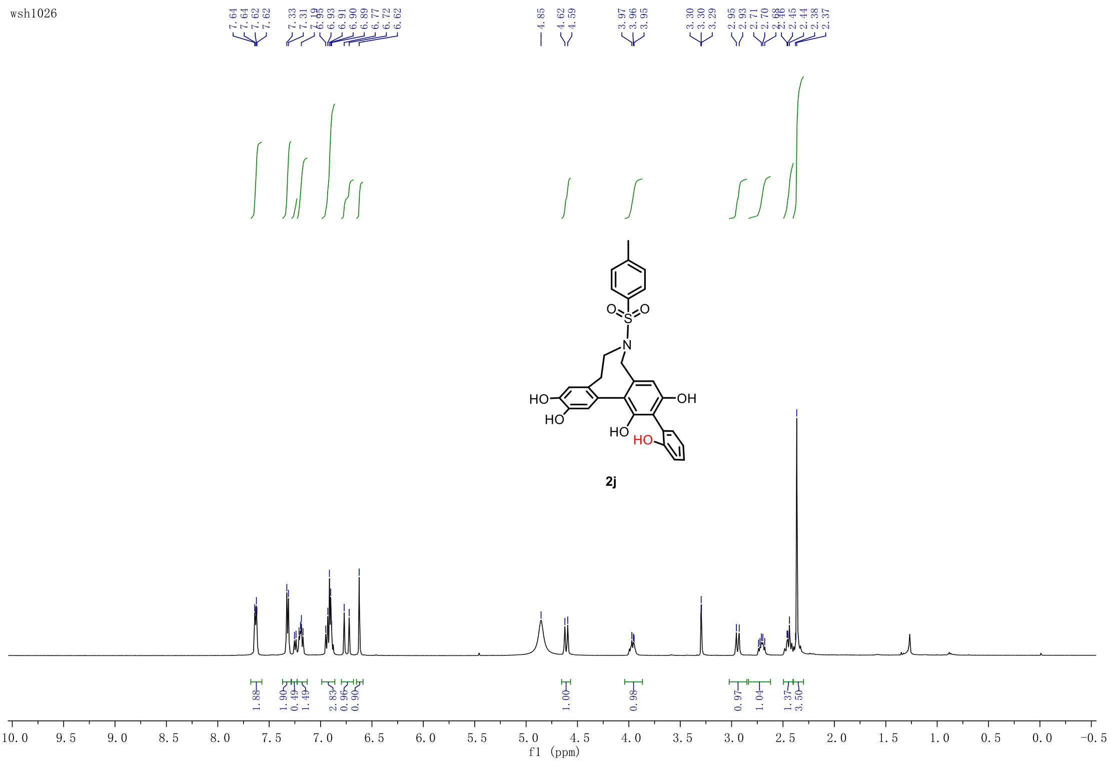

^{13}C NMR spectrum of compound 2j (125 MHz) in CD_3OD

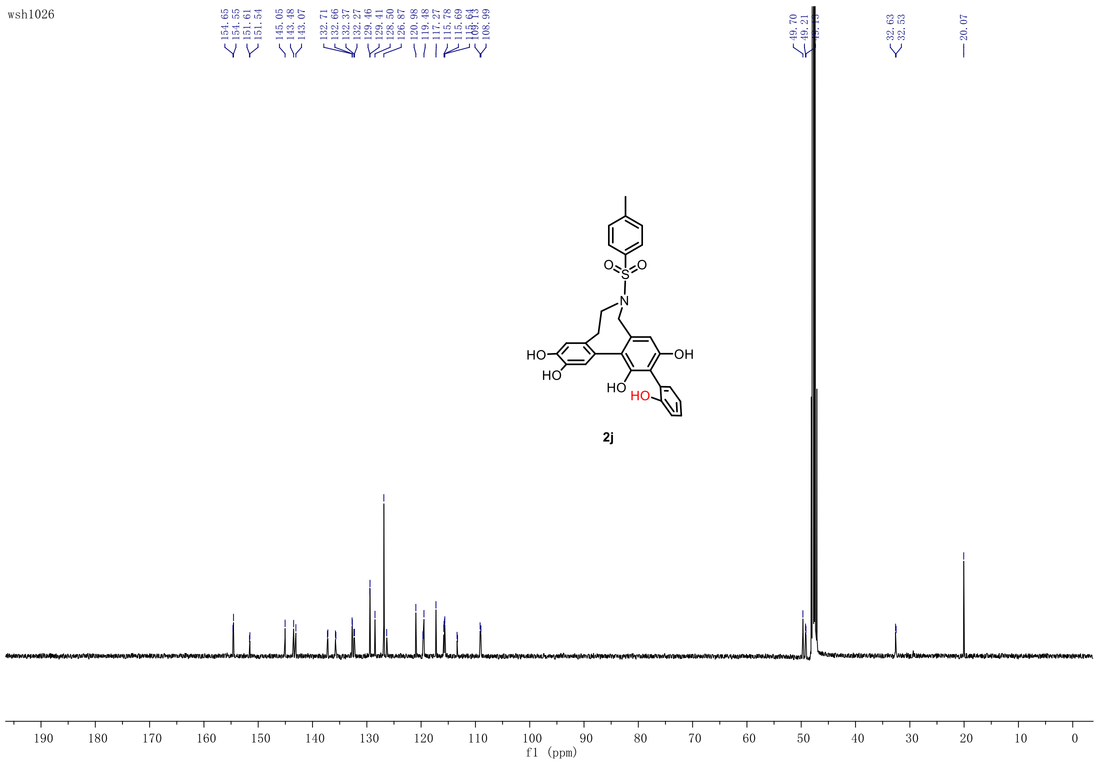

^{1}H NMR spectrum of compound 2k (500 MHz) in CD_3OD

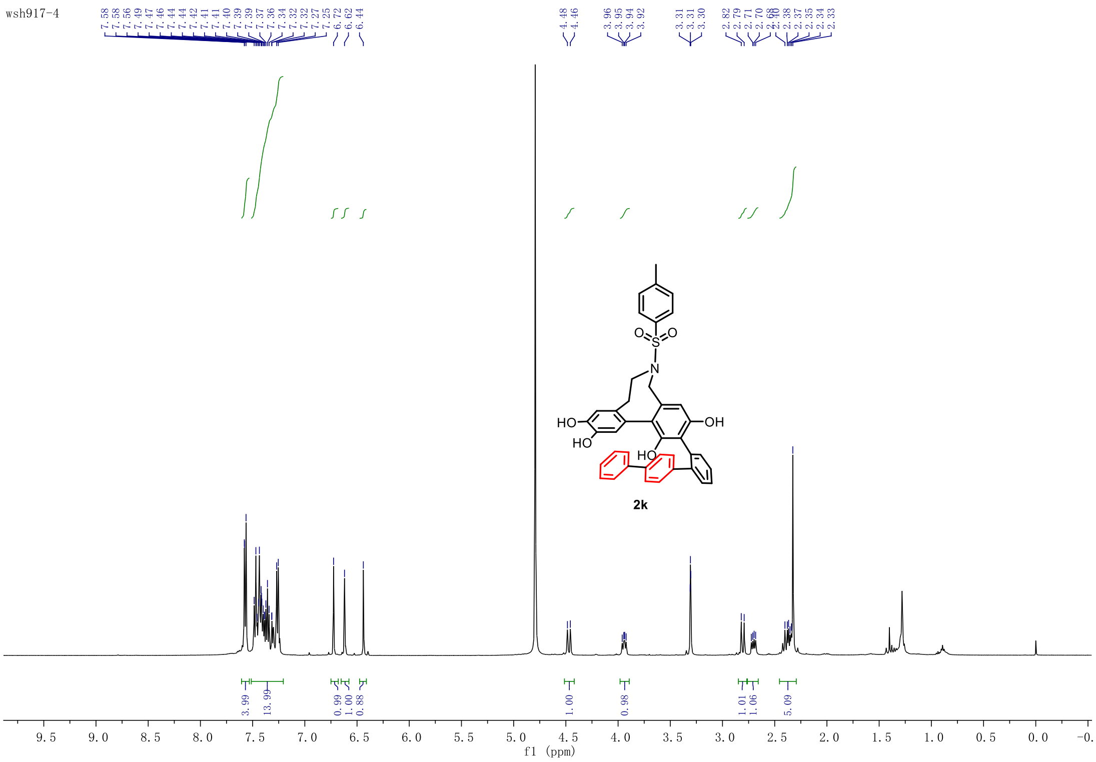

^{13}C NMR spectrum of compound 2k (125 MHz) in CD_3OD

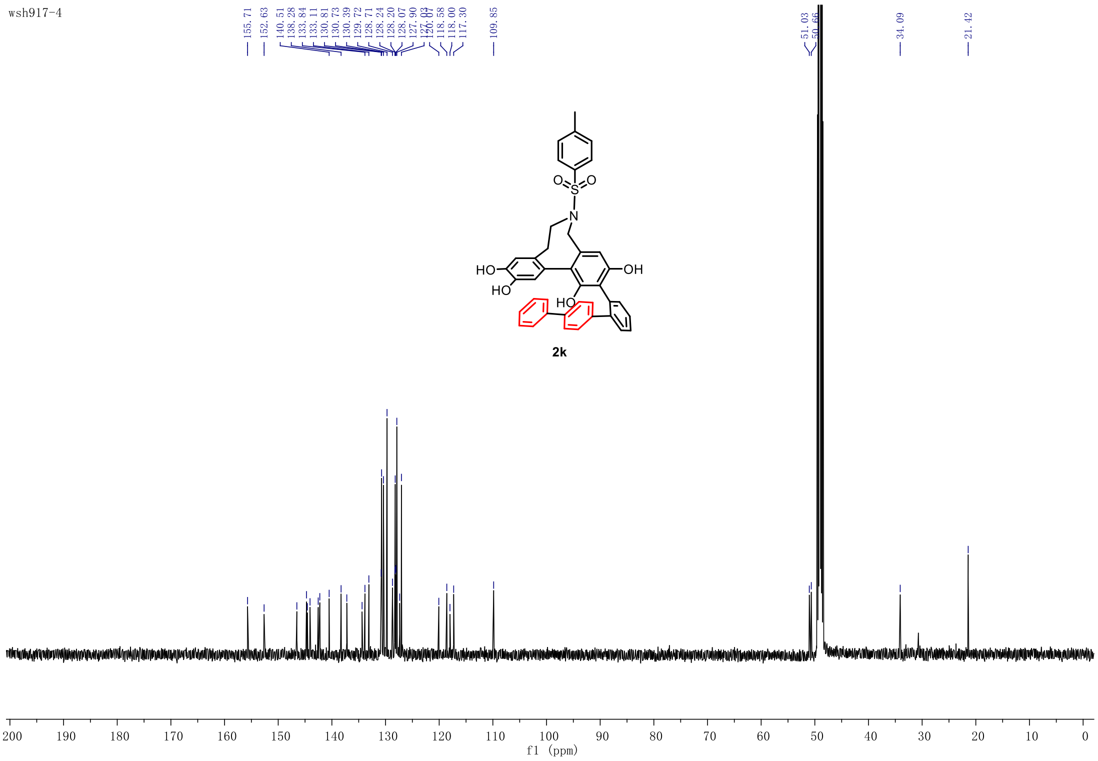

^{1}H NMR spectrum of compound 2l (500 MHz) in CD_3OD

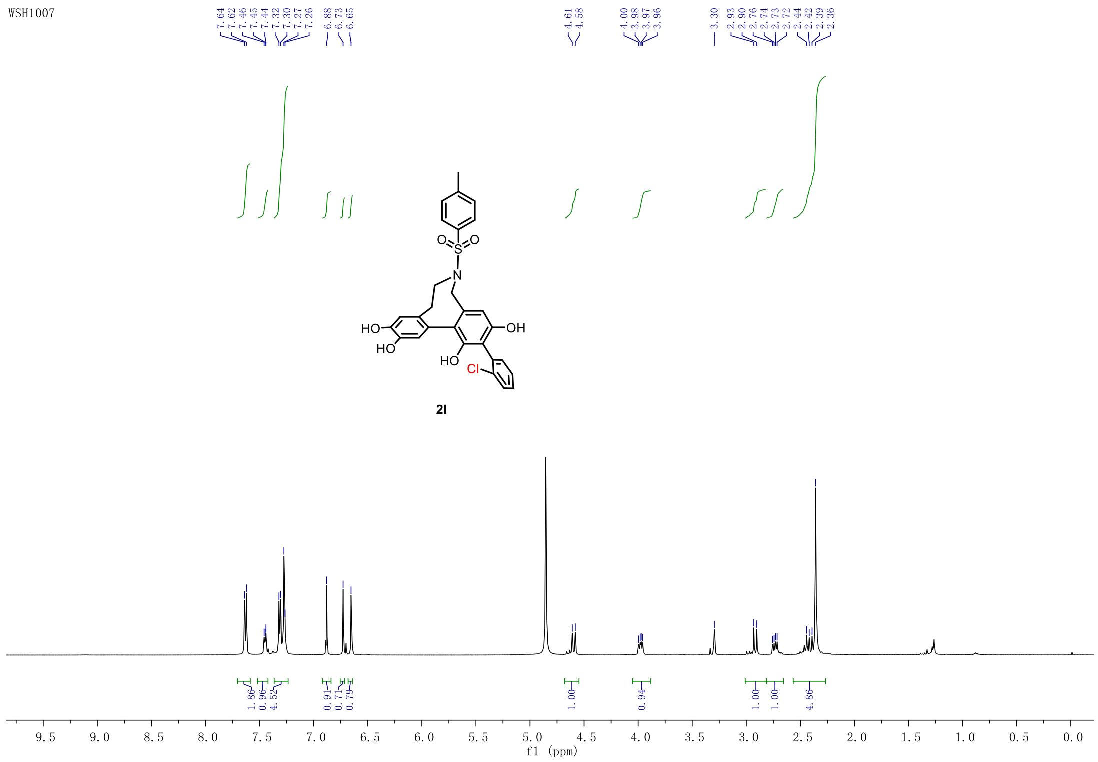

^{13}C NMR spectrum of compound 2l (125 MHz) in CD_3OD

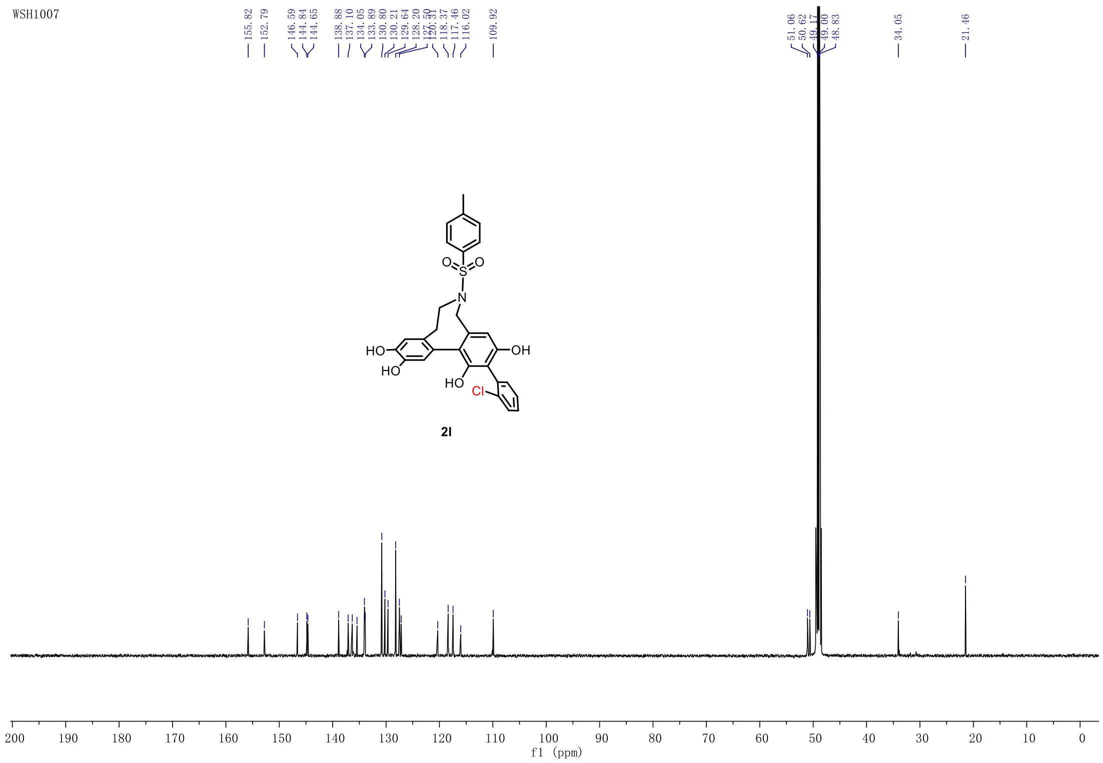

^{1}H NMR spectrum of compound 2m (500 MHz) in CD_3OD

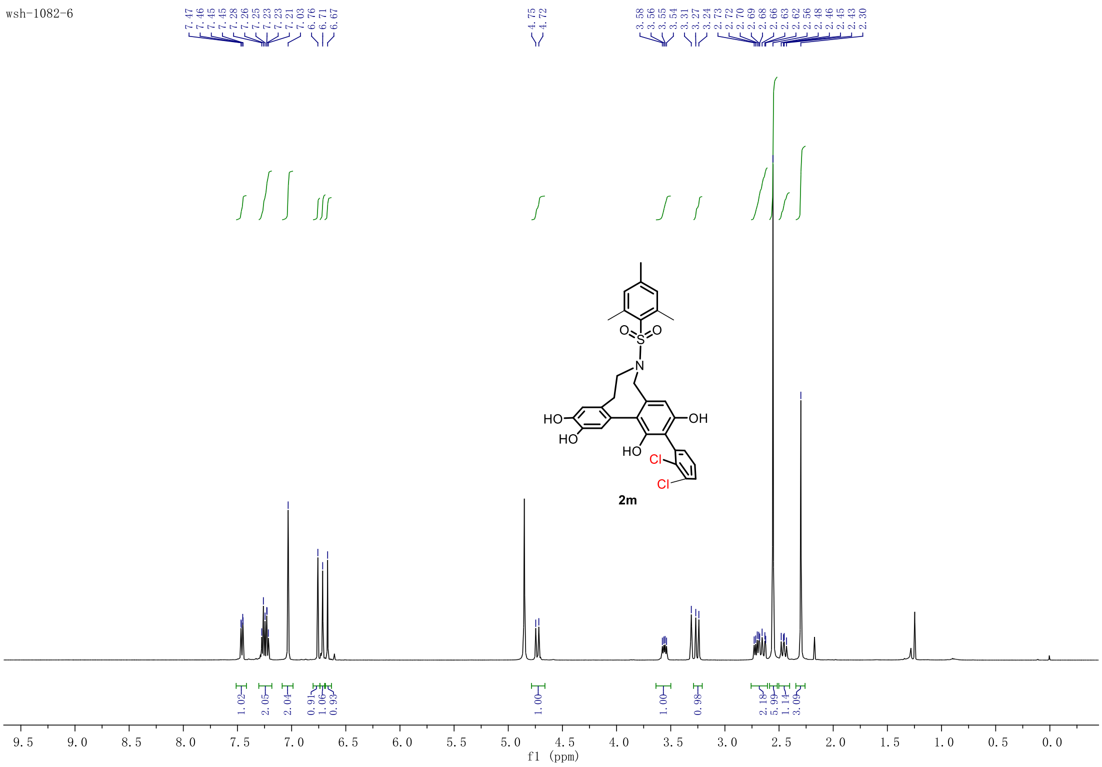

^{13}C NMR spectrum of compound 2m (125 MHz) in CD_3OD

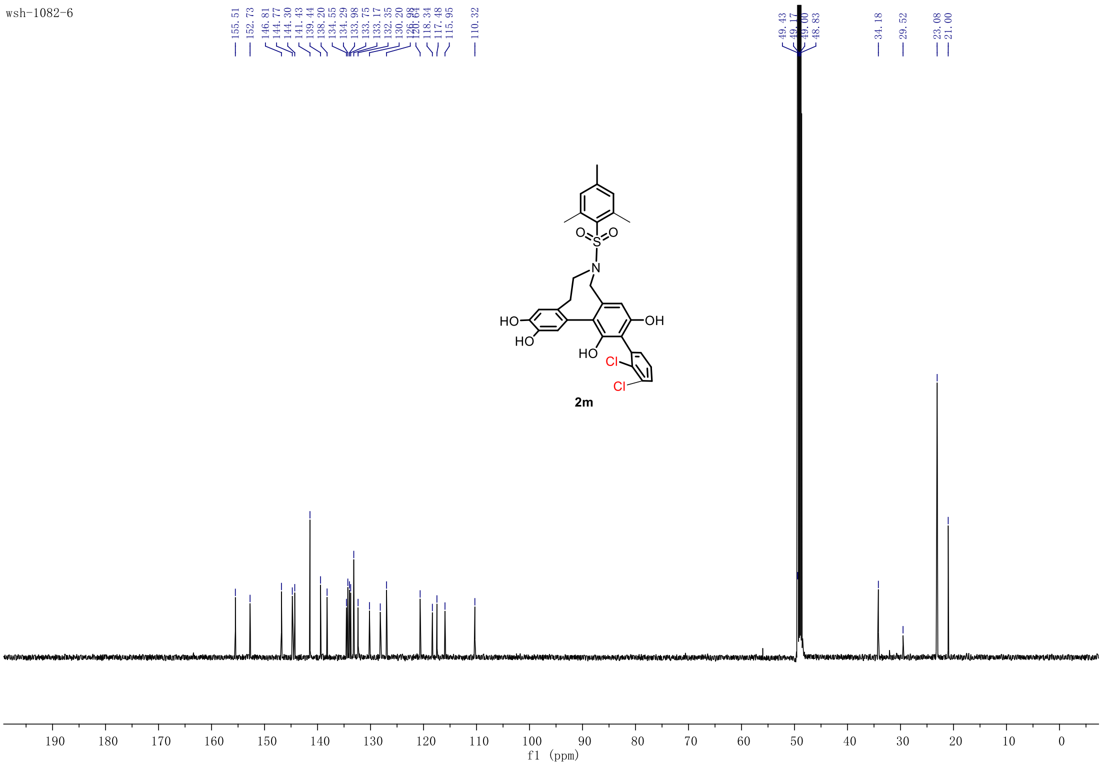

^{1}H NMR spectrum of compound 2n (500 MHz) in Acetone-d_6

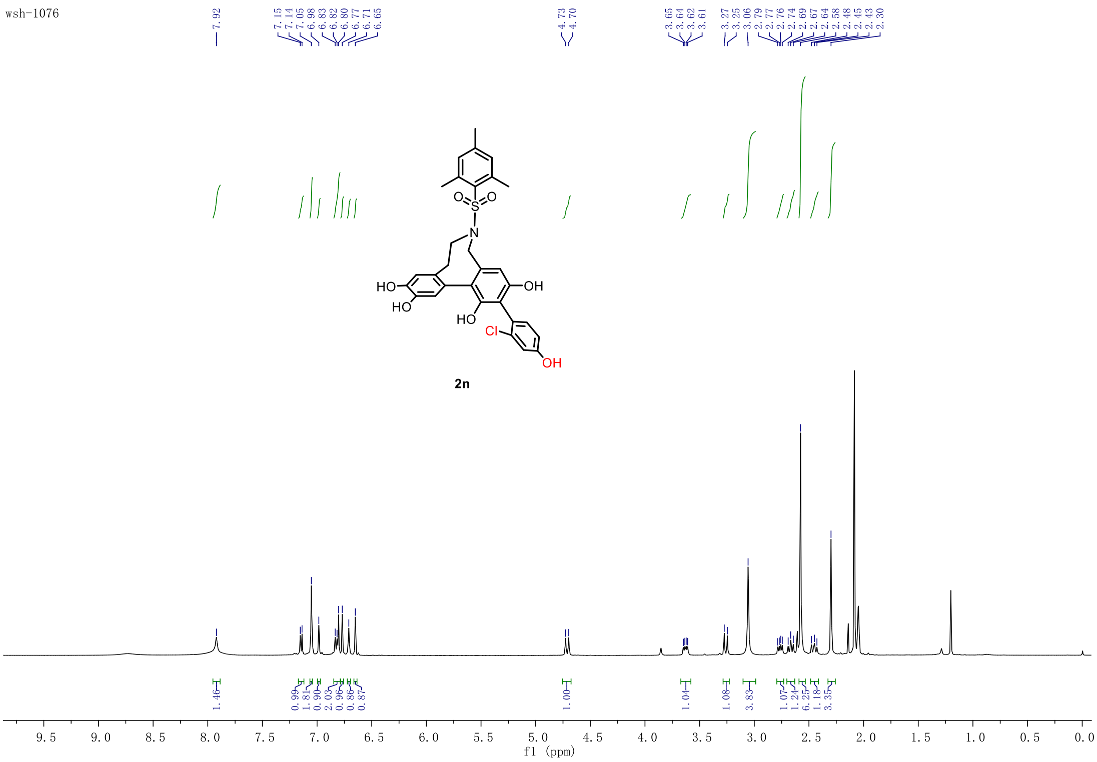

^{13}C NMR spectrum of compound 2n (125 MHz) in Acetone-d_6

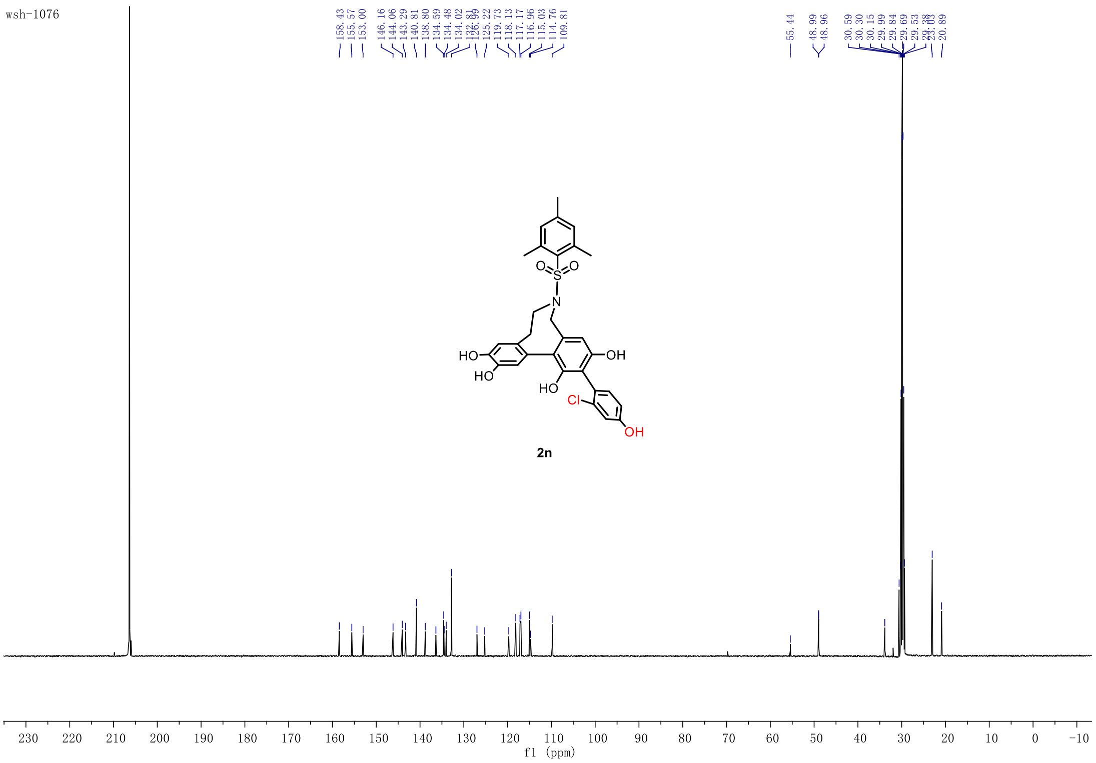

^{1}H NMR spectrum of compound 2o (500 MHz) in Acetone-d_6

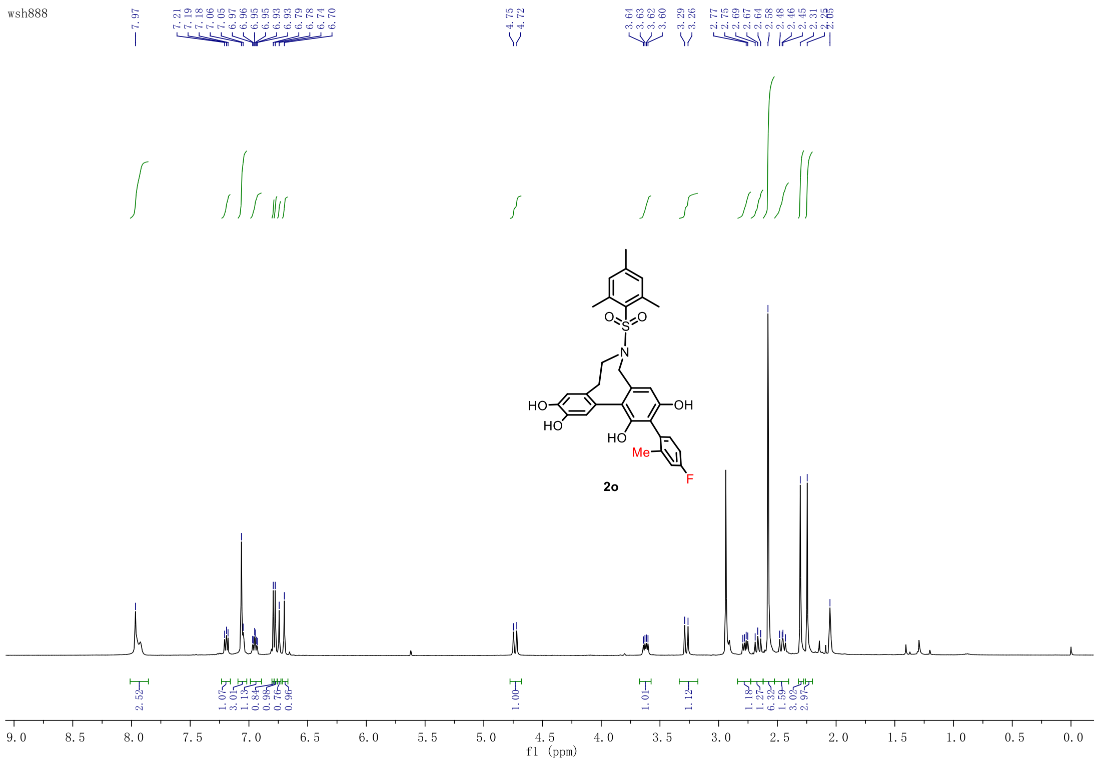

^{13}C NMR spectrum of compound 2o (125 MHz) in Acetone-d_6

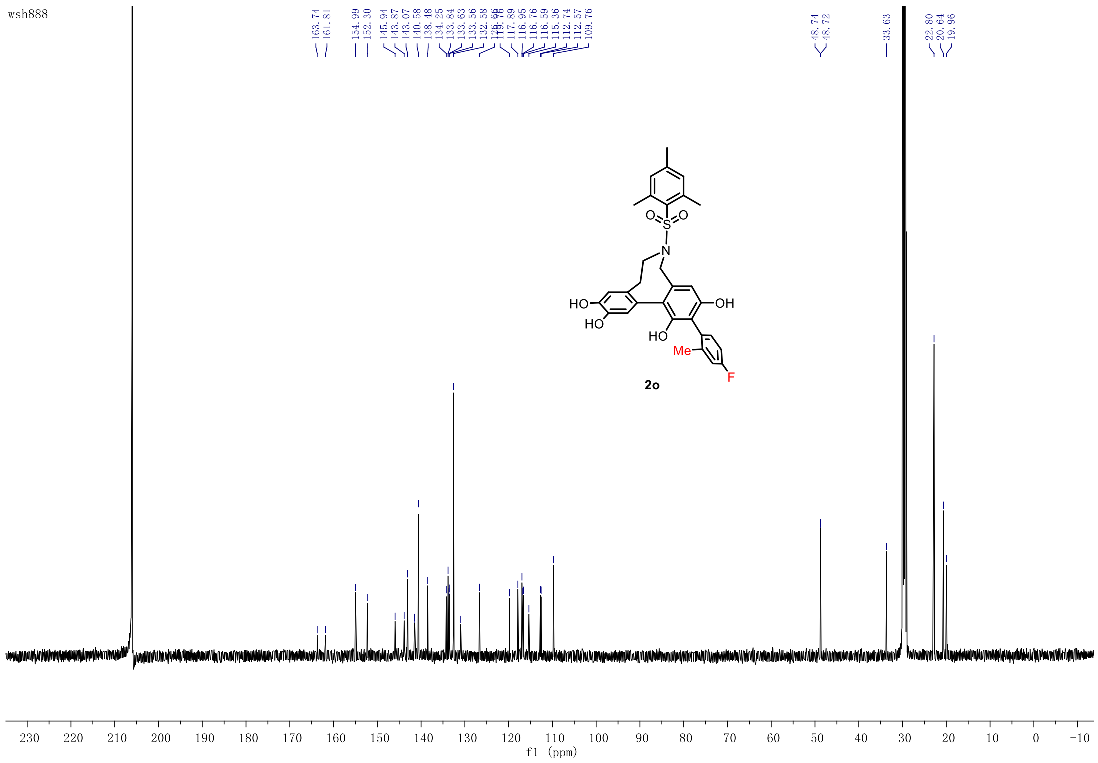

^{19}F NMR spectrum of compound 2o (376 MHz) in Methanol-d_4

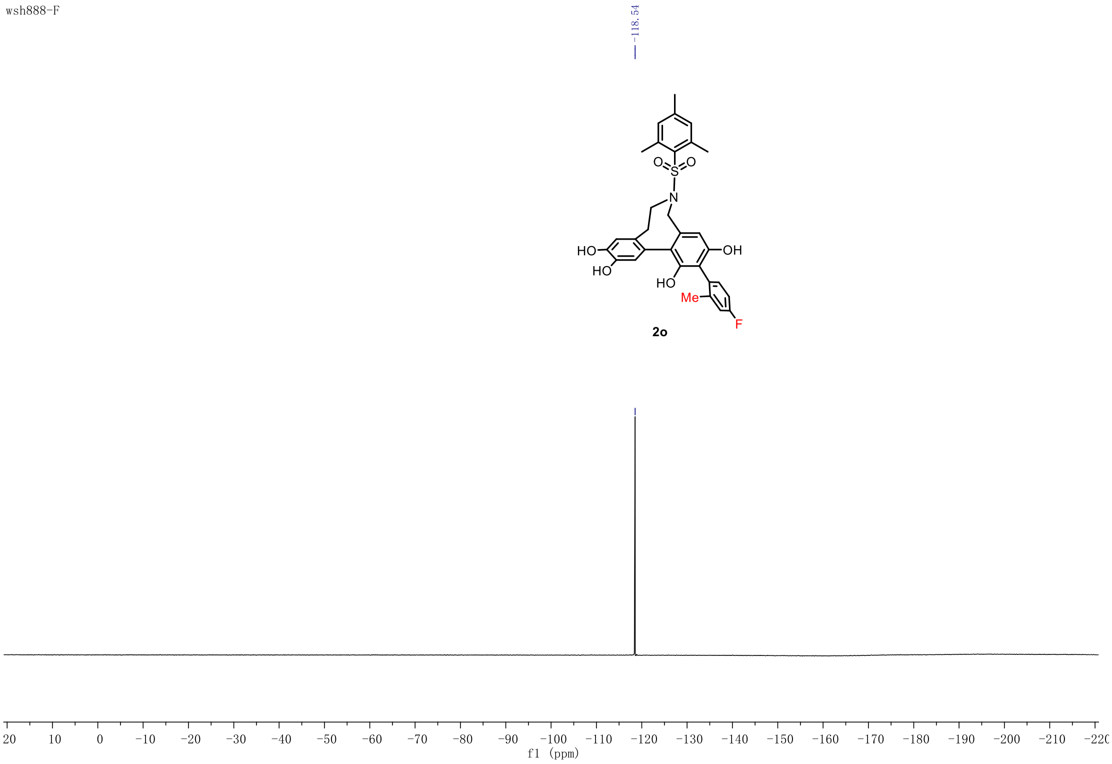

^{1}H NMR spectrum of compound 2p (500 MHz) in Acetone-d_6

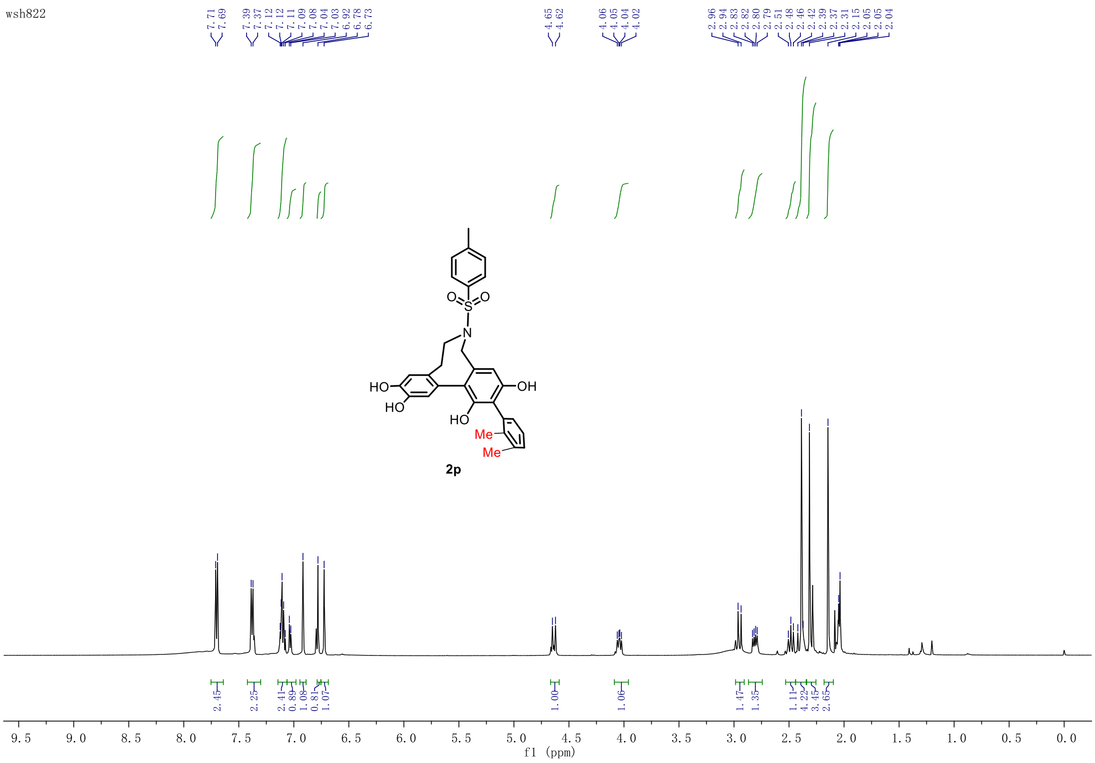

^{13}C NMR spectrum of compound 2p (125 MHz) in Acetone-d_6

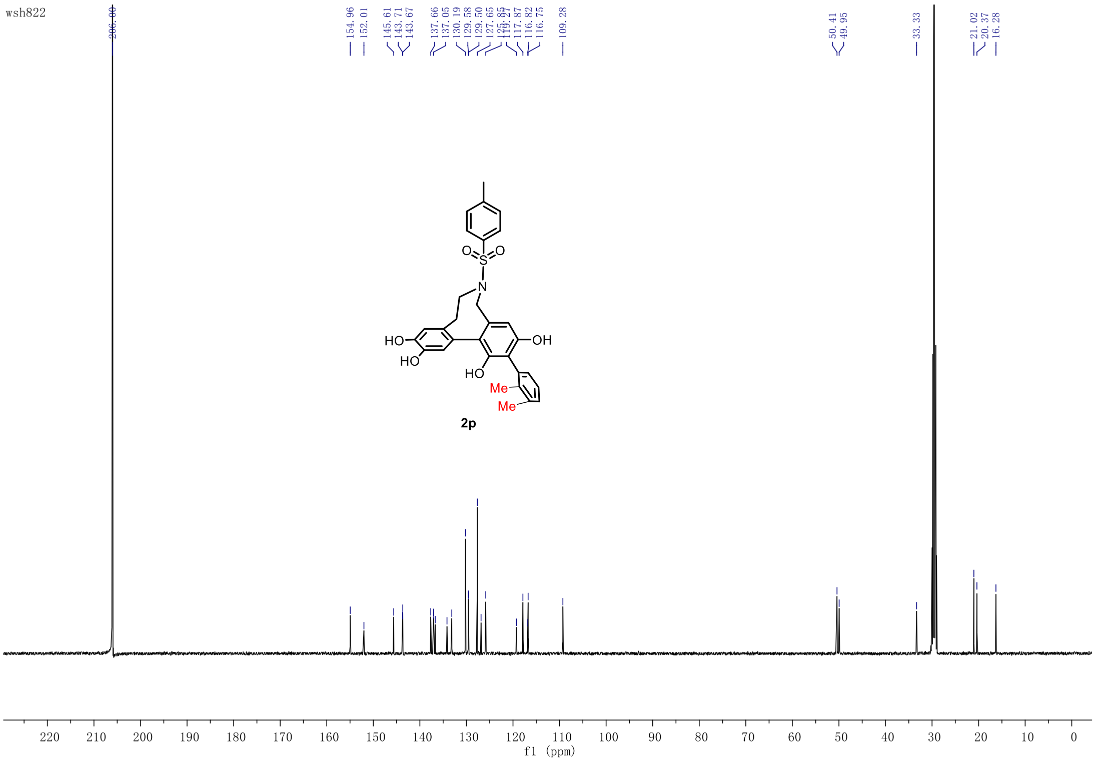

^{1}H NMR spectrum of compound 2q (500 MHz) in Acetone-d_6

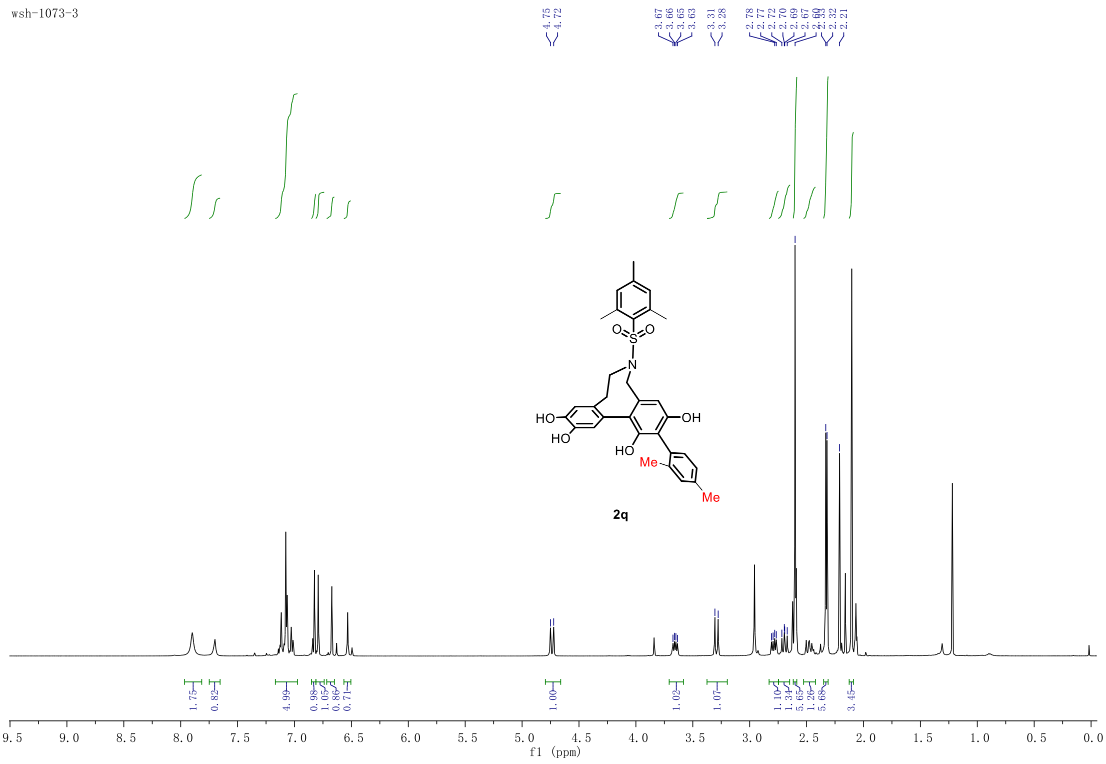

^{13}C NMR spectrum of compound 2q (125 MHz) in Acetone-d_6

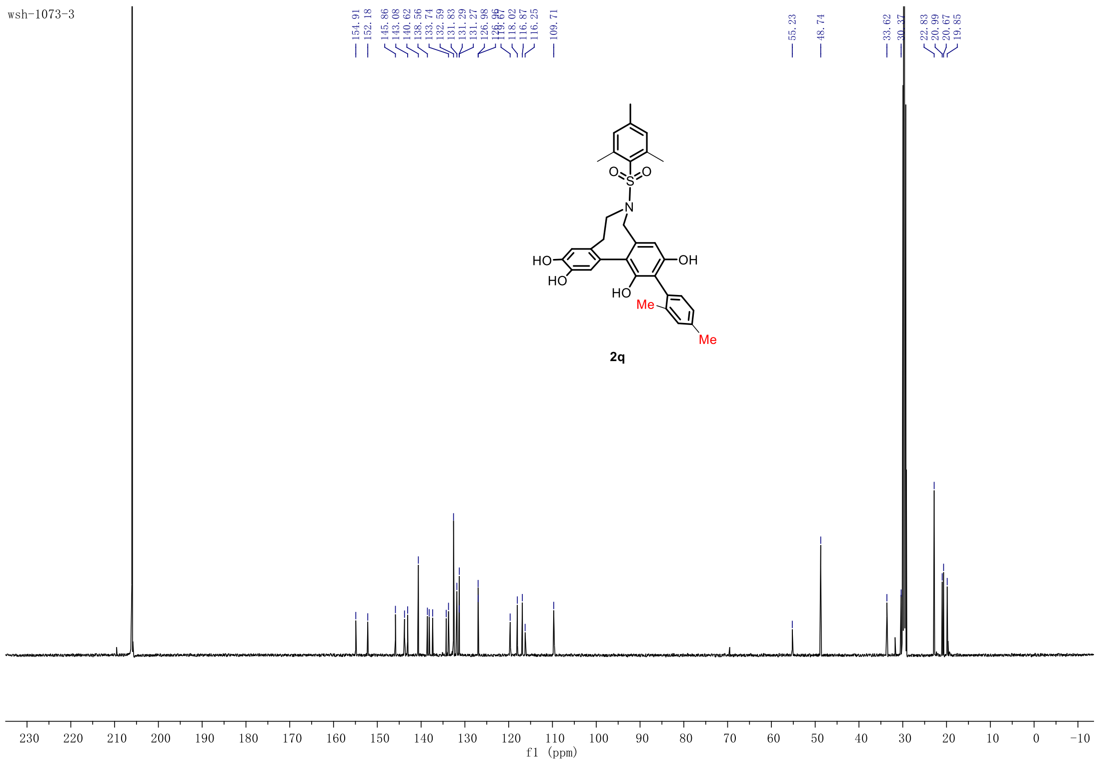

^{1}H NMR spectrum of compound 2r (500 MHz) in Acetone-d_6

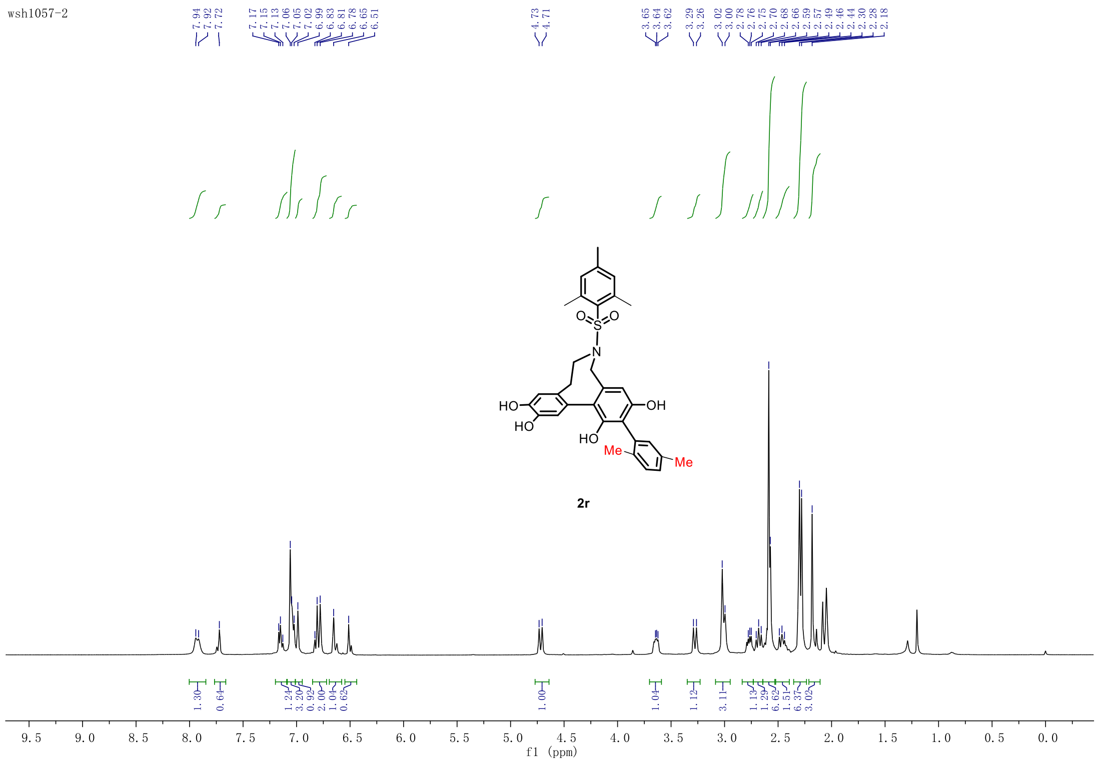

^{13}C NMR spectrum of compound 2r (125 MHz) in Acetone-d_6

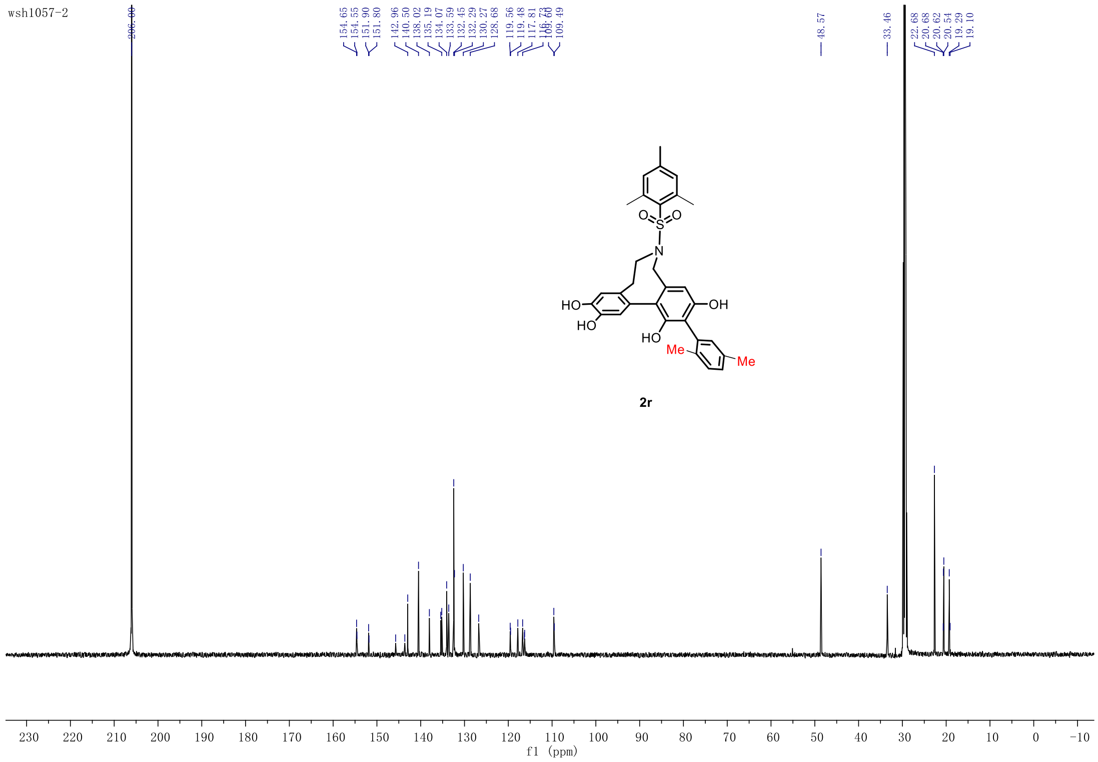

^{1}H NMR spectrum of compound 2s (500 MHz) in Acetone-d_6

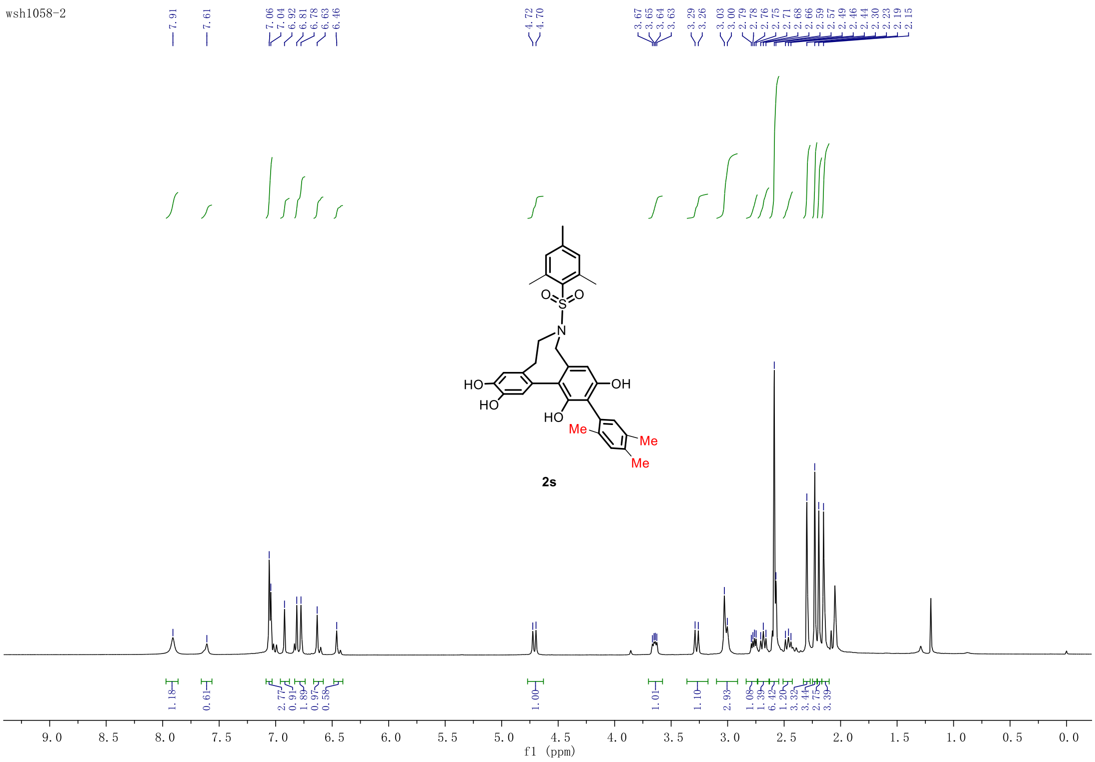

^{13}C NMR spectrum of compound 2s (125 MHz) in Acetone-d_6

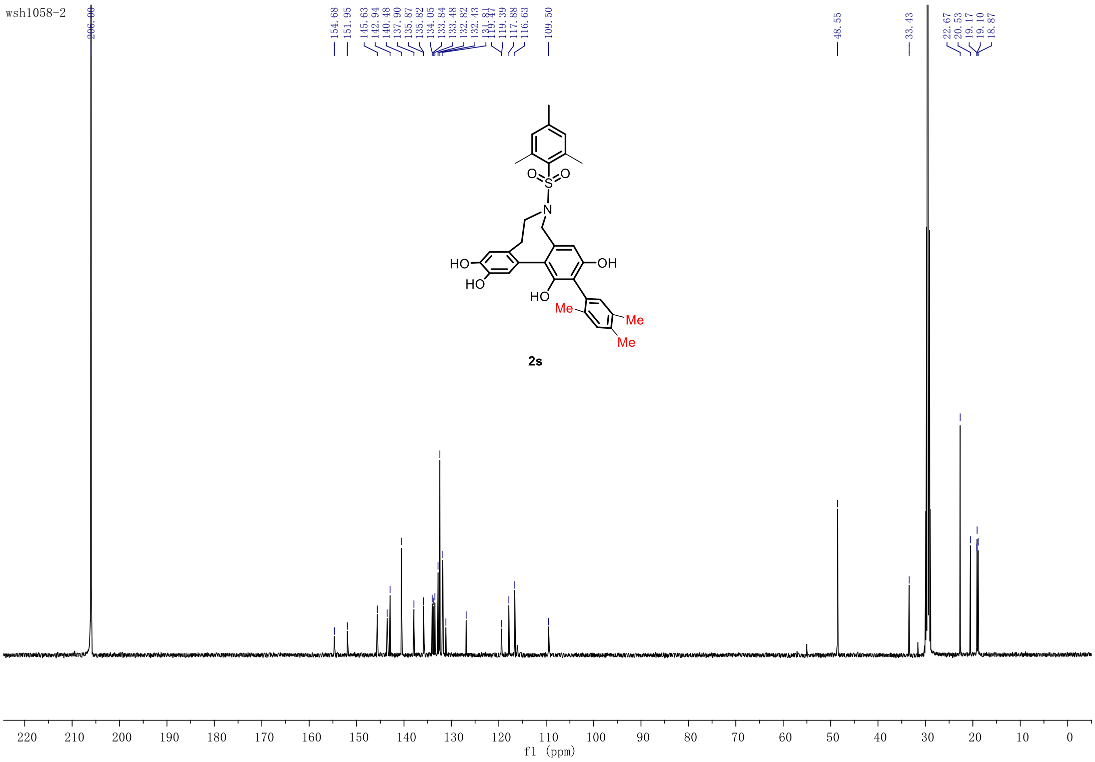

^{1}H NMR spectrum of compound 2t (500 MHz) in CD_3OD

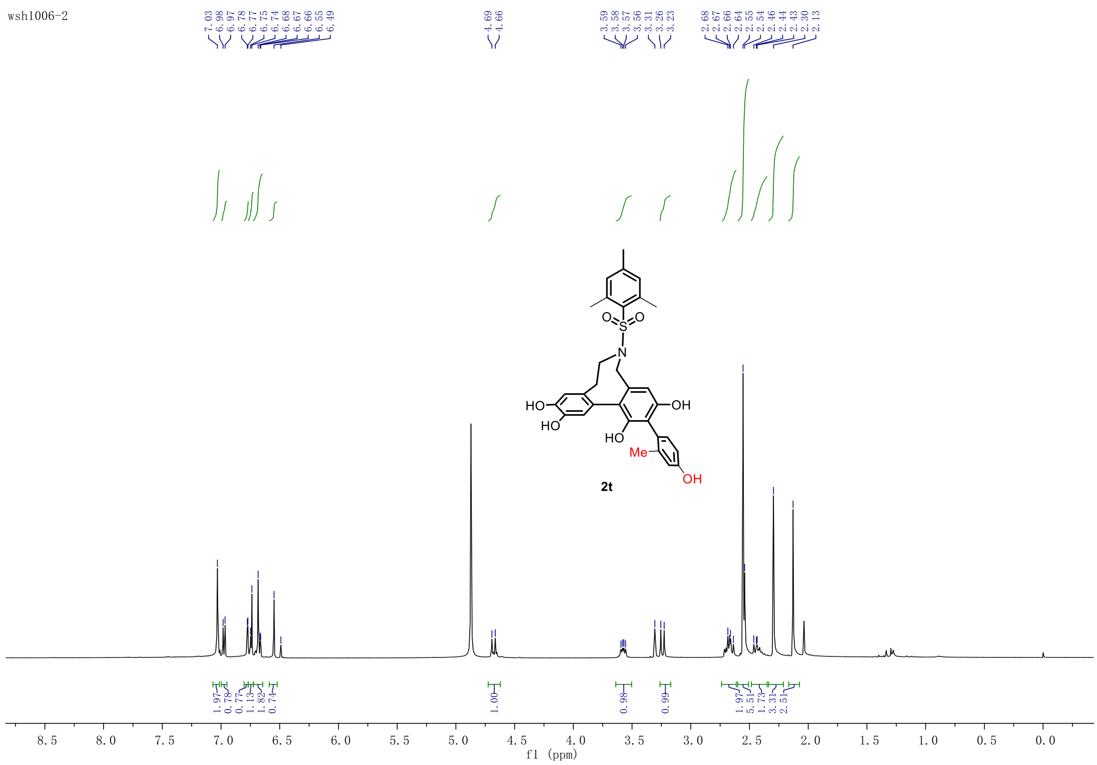

^{13}C NMR spectrum of compound 2t (125 MHz) in CD_3OD

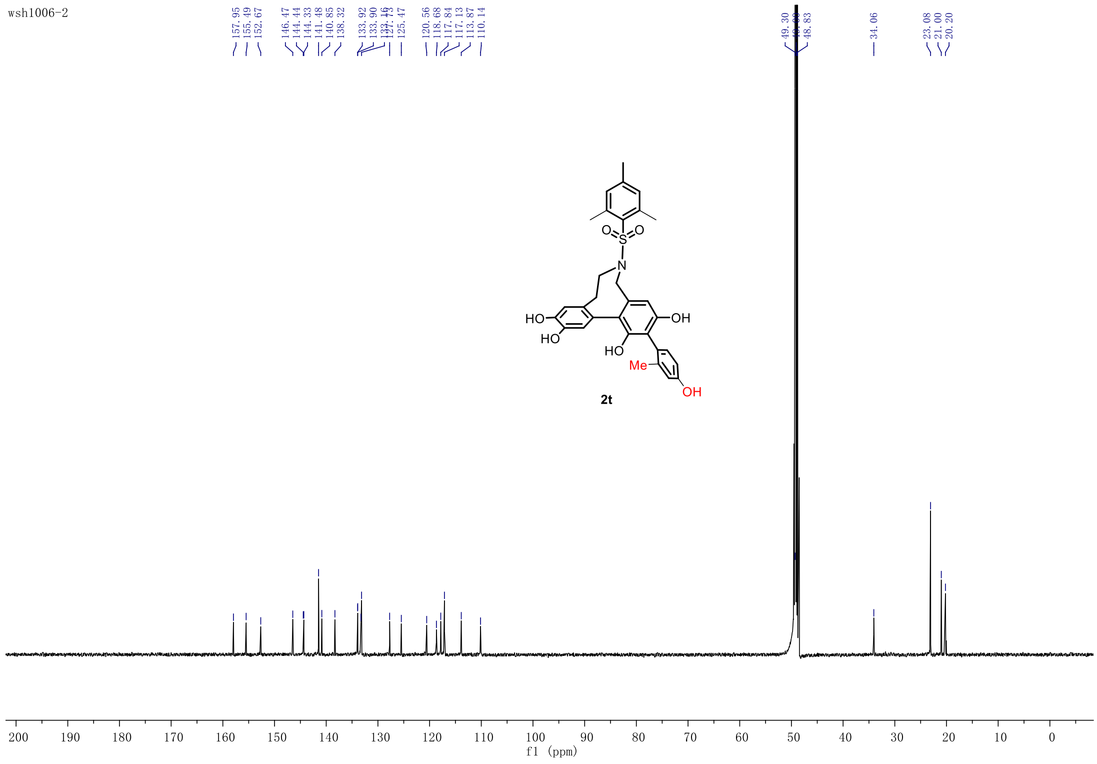

^{1}H NMR spectrum of compound 2u (500 MHz) in Acetone-d_6

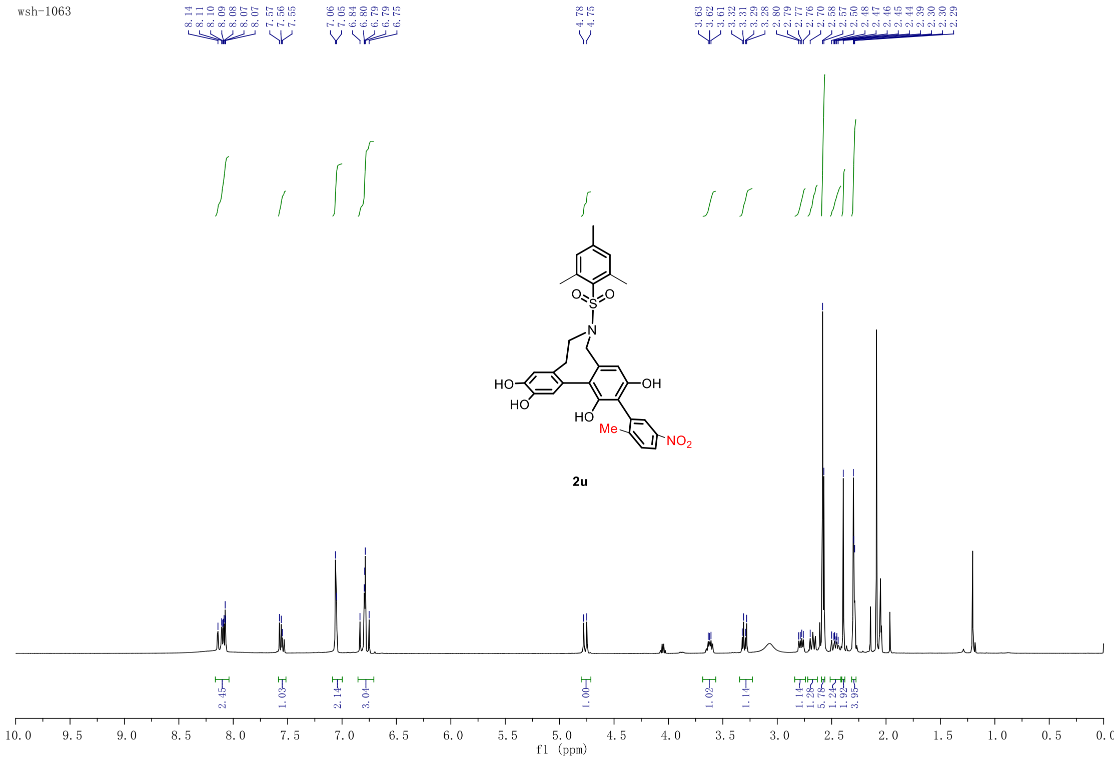

^{13}C NMR spectrum of compound 2u (125 MHz) in Acetone-d_6

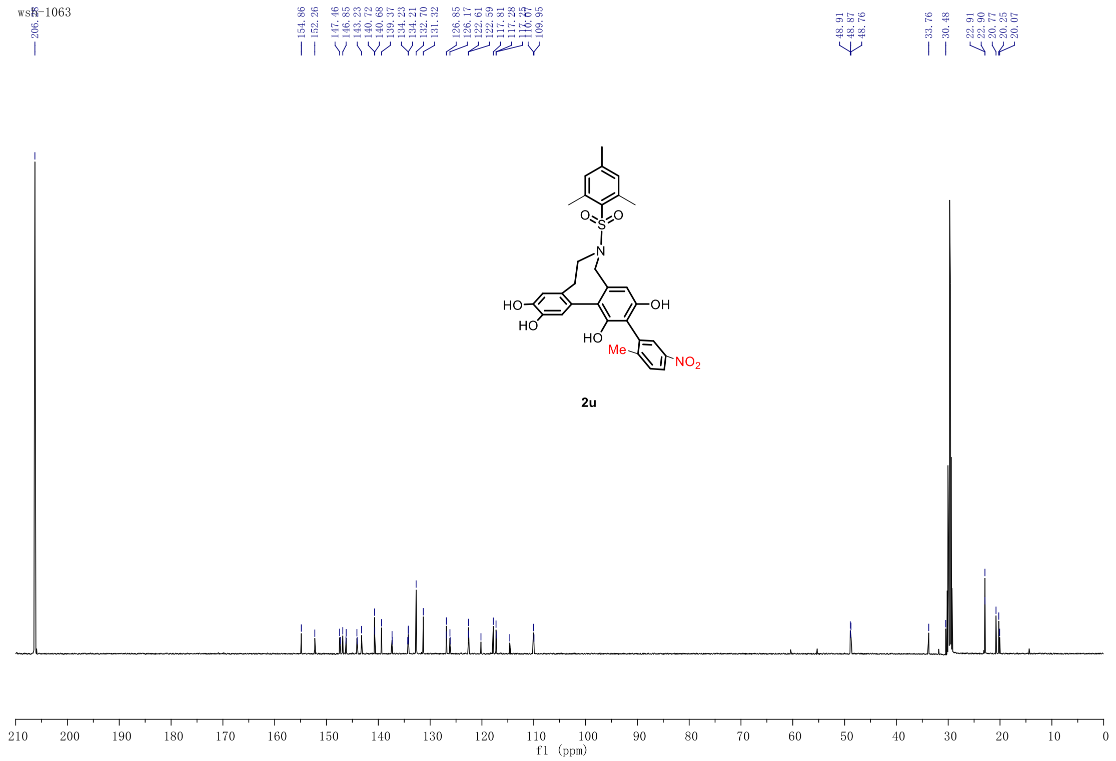

^{1}H NMR spectrum of compound 2v (500 MHz) in Acetone-d_6

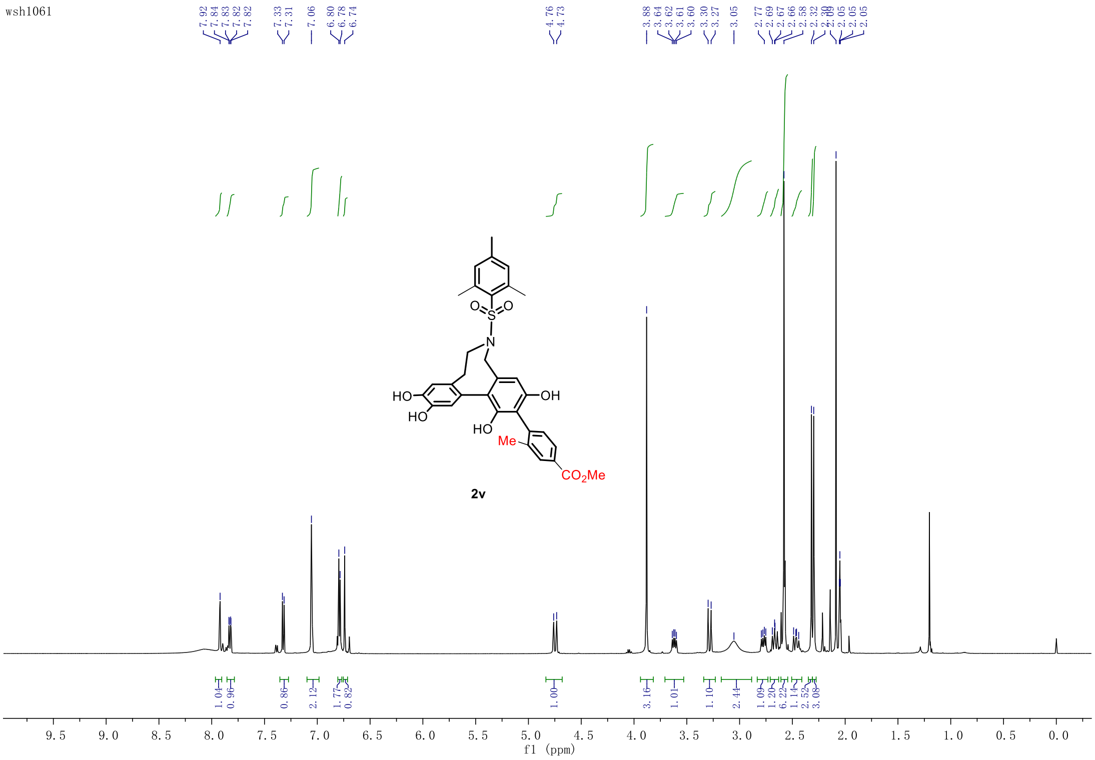

^{13}C NMR spectrum of compound 2v (125 MHz) in Acetone-d_6

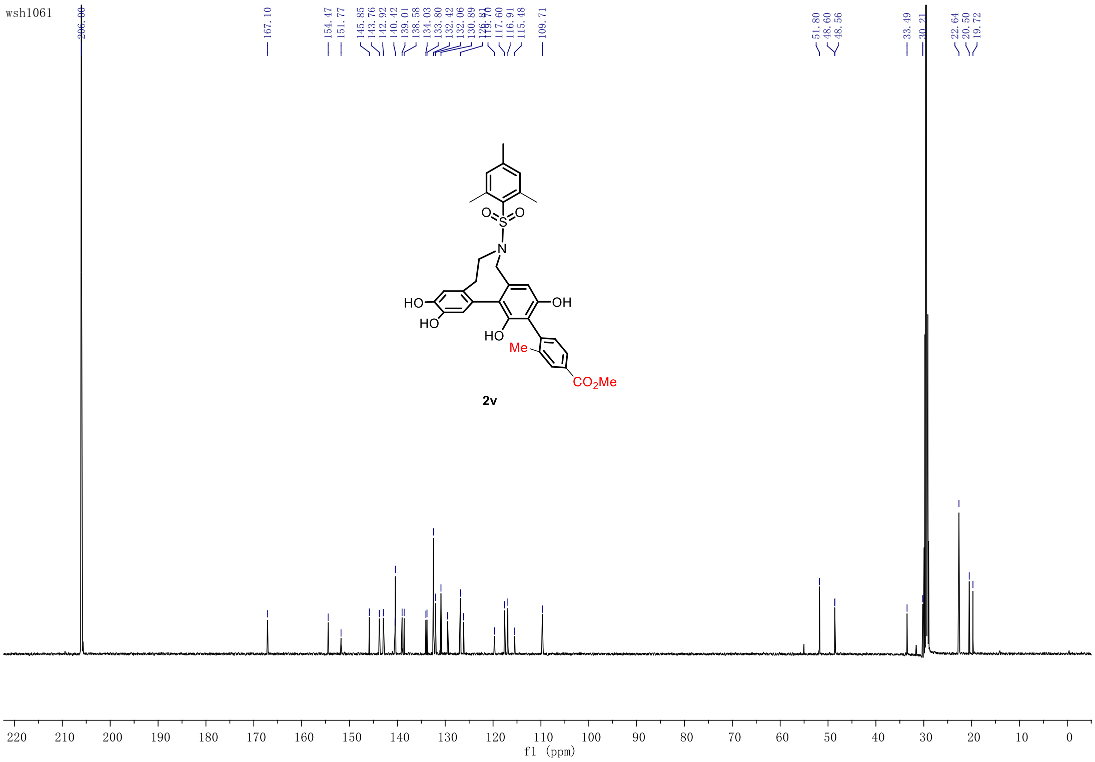

^{1}H NMR spectrum of compound 2w (500 MHz) in Acetone-d_6

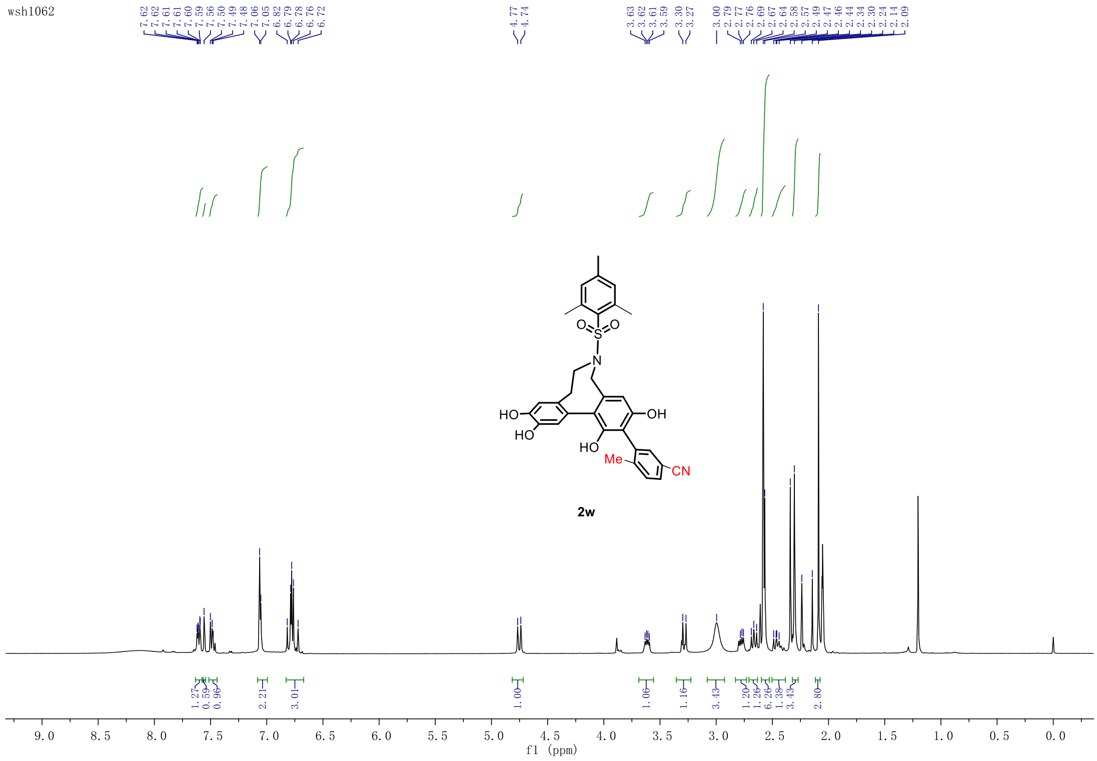

^{1}H NMR spectrum of compound 2w (500 MHz) in Acetone-d_6

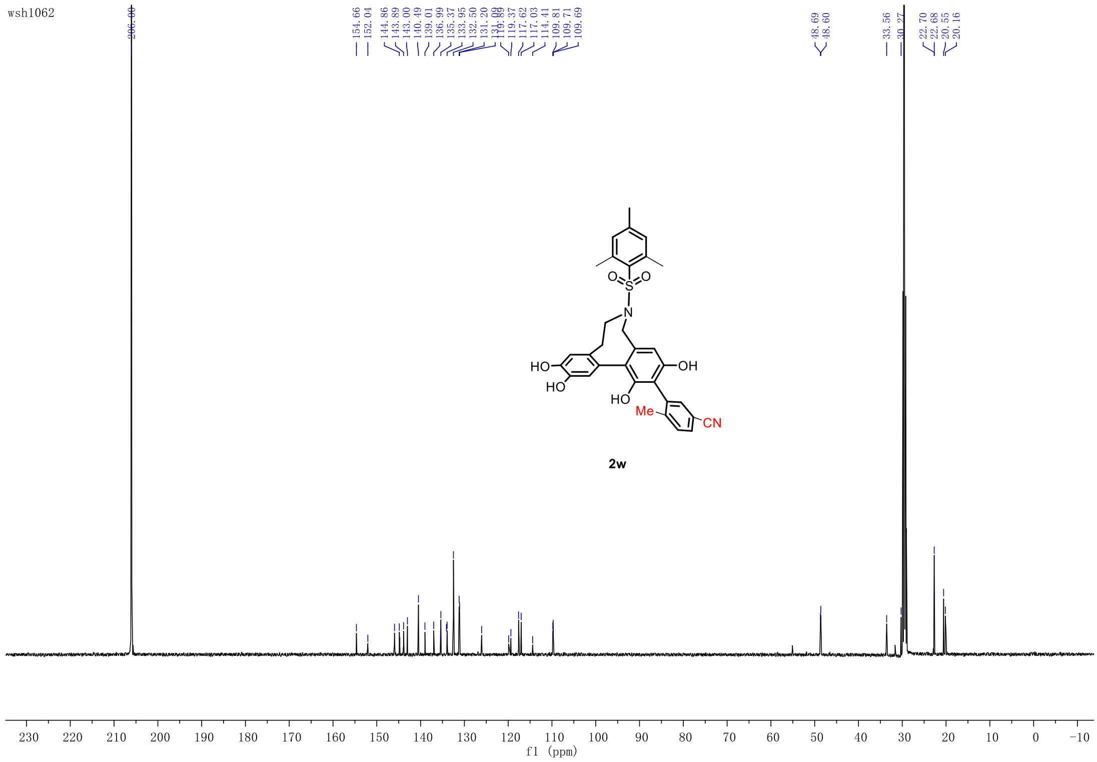

^{1}H NMR spectrum of compound 2x (500 MHz) in CD_3OD

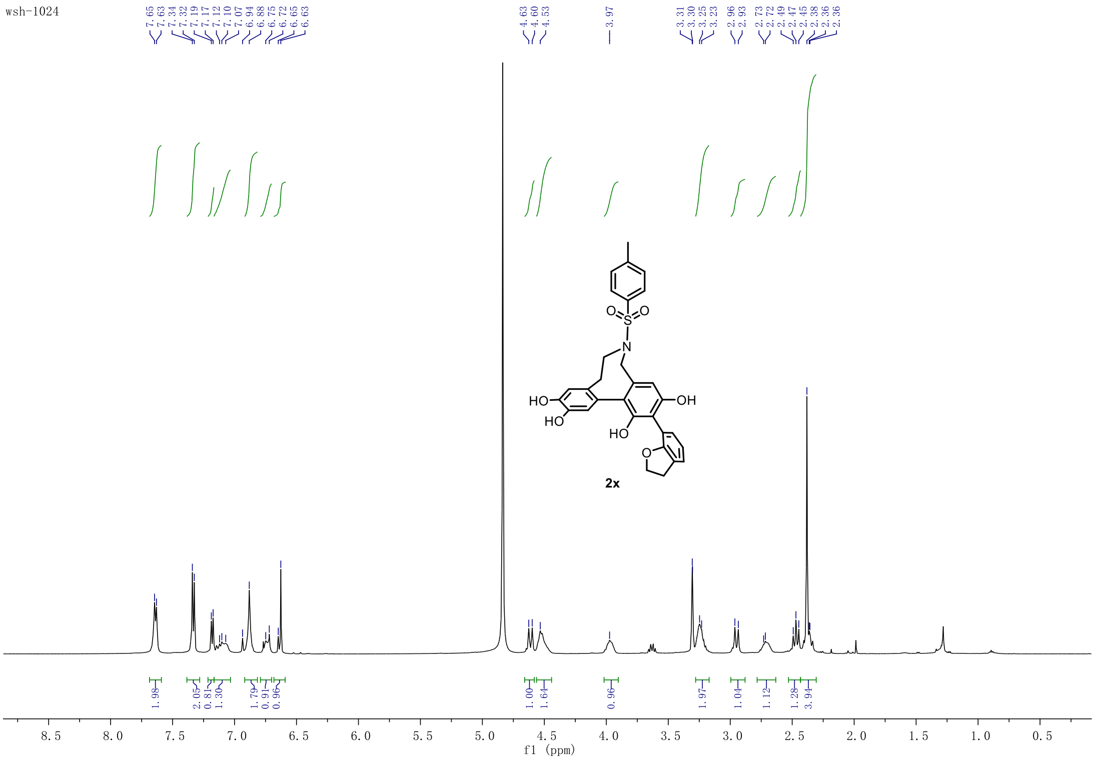

^{13}C NMR spectrum of compound 2x (125 MHz) in CD_3OD

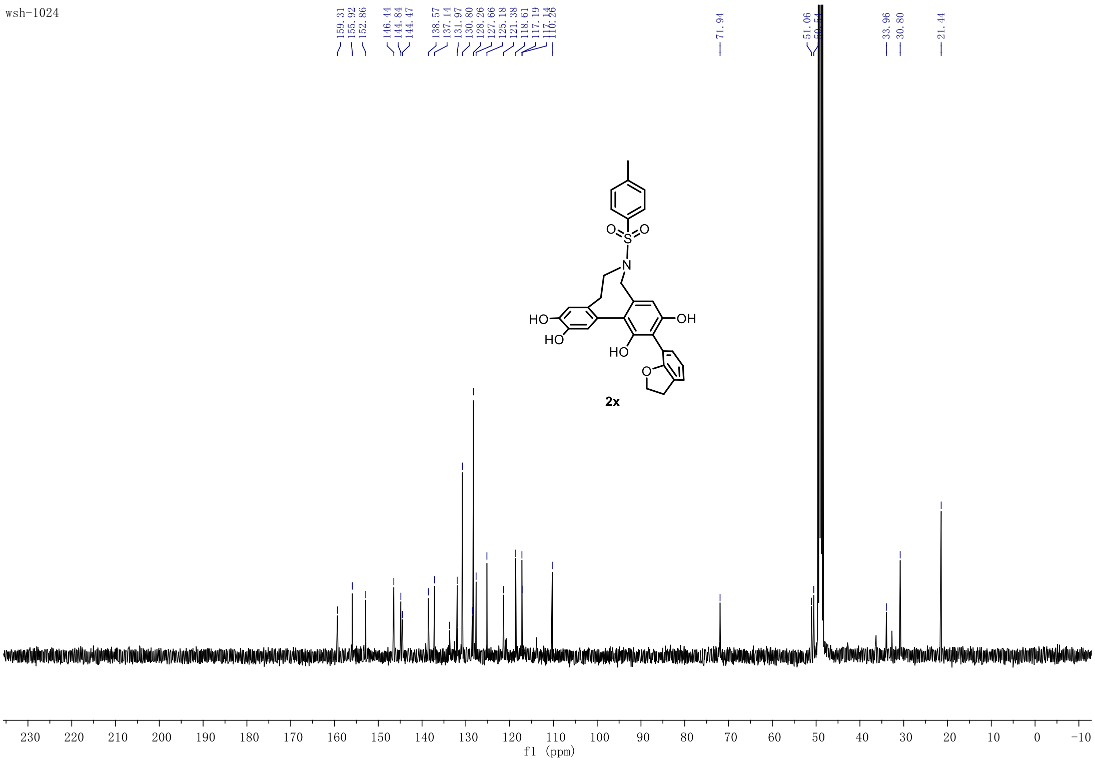

^{1}H NMR spectrum of compound 2y (500 MHz) in CD_3OD

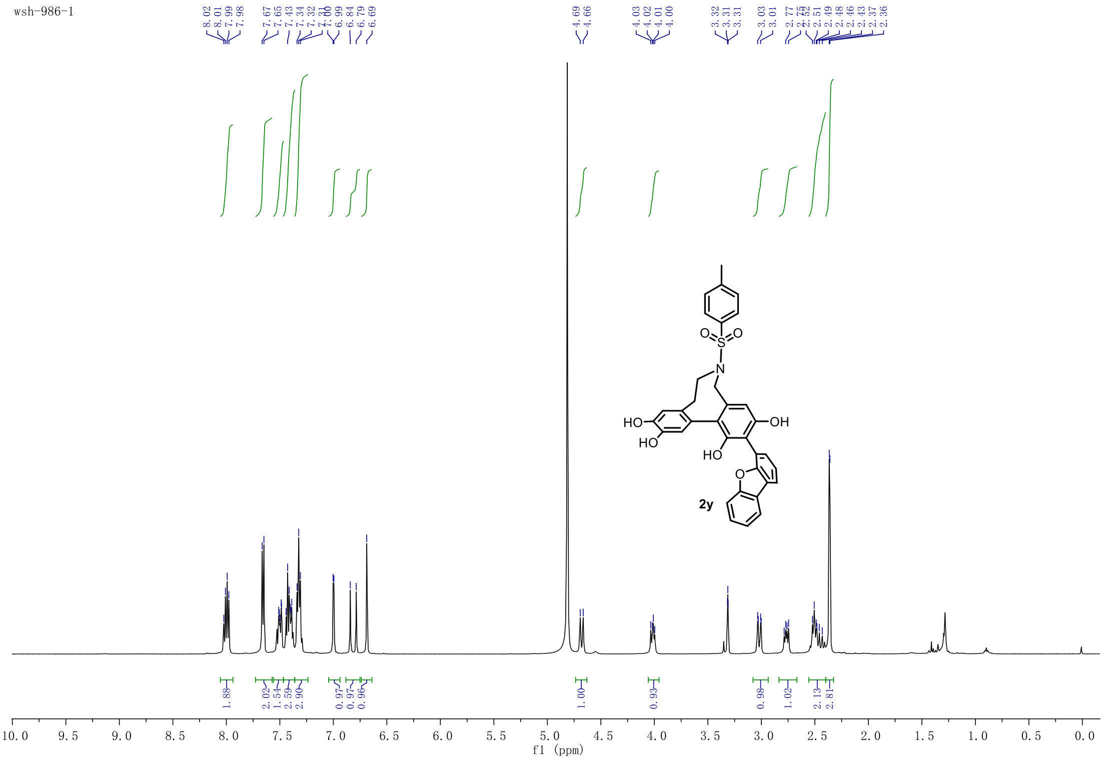

^{13}C NMR spectrum of compound 2y (125 MHz) in CD_3OD

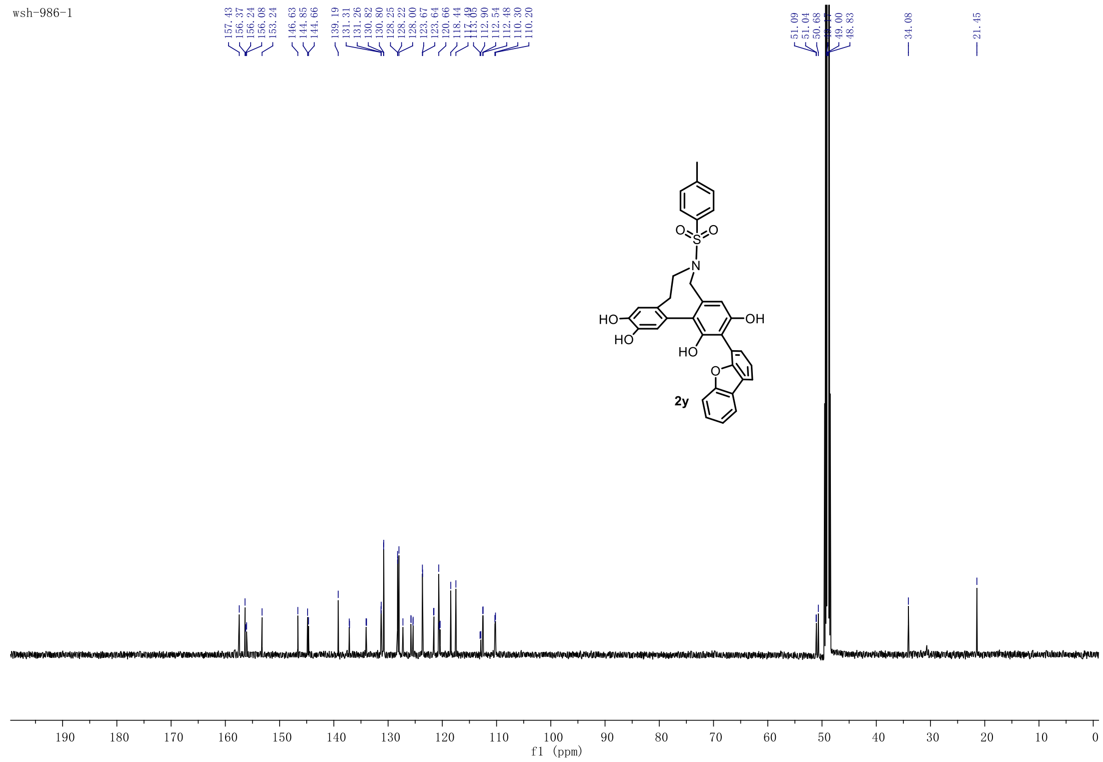

^{1}H NMR spectrum of compound 2z (500 MHz) in CD_3OD

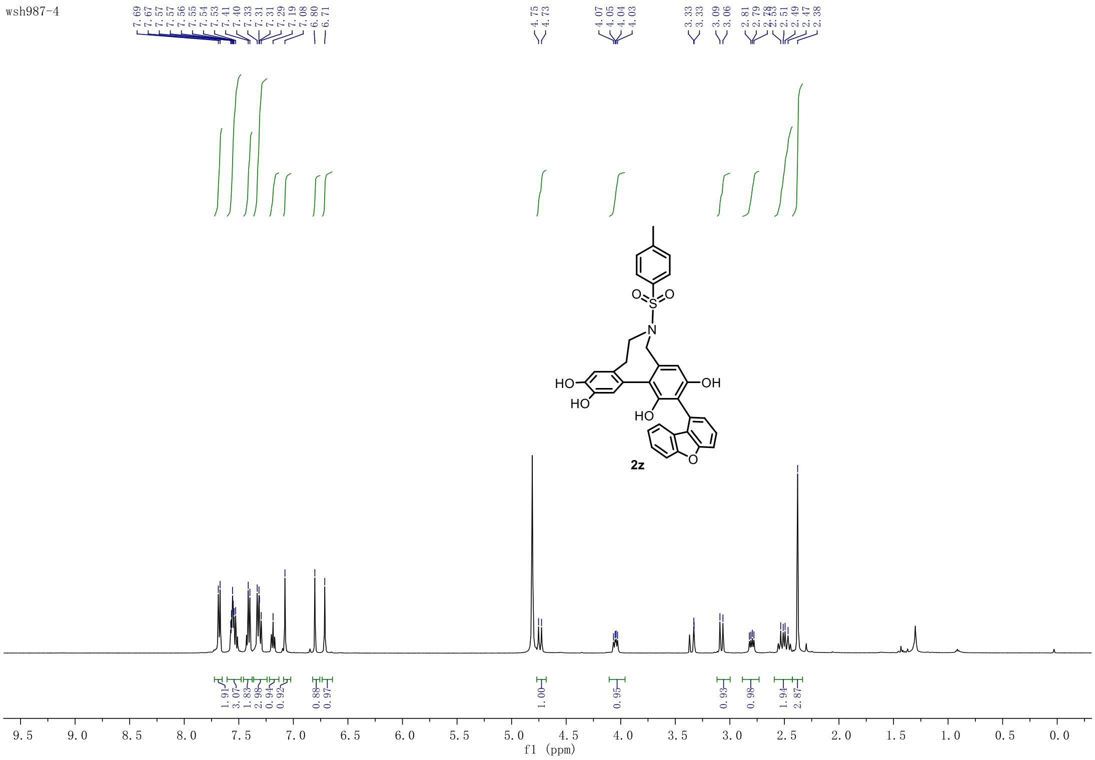

^{13}C NMR spectrum of compound 2z (125 MHz) in CD_3OD

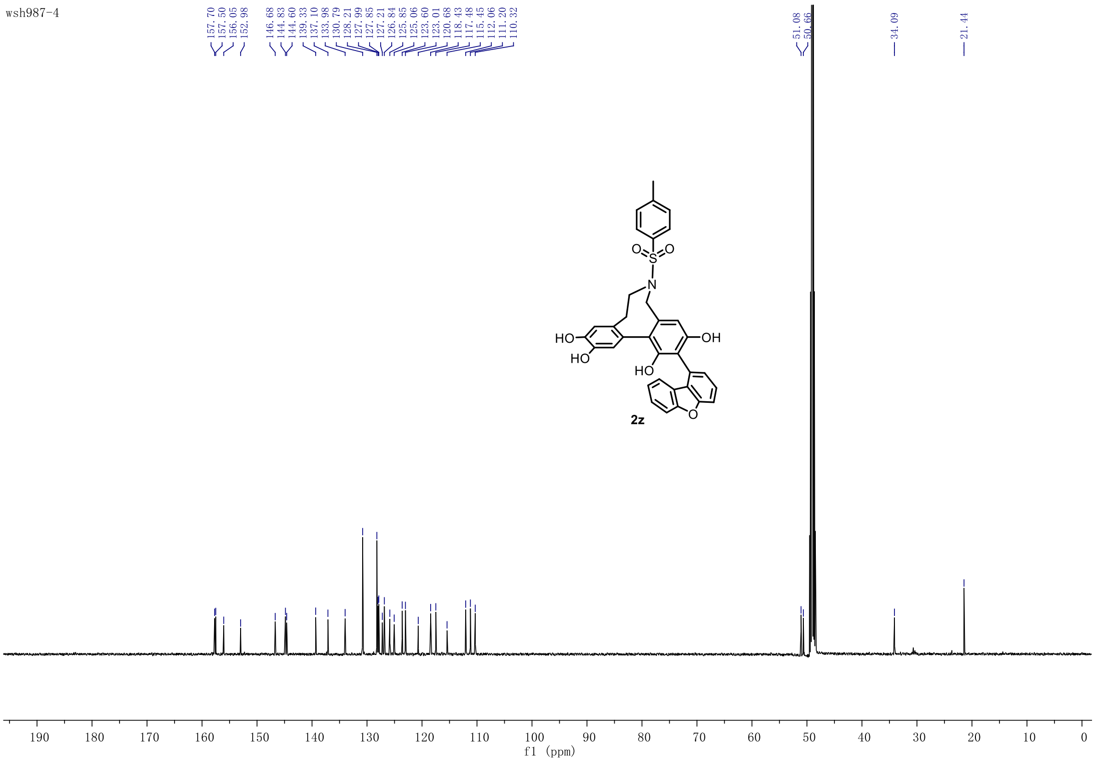

^{1}H NMR spectrum of compound 2aa (500 MHz) in CD_3OD

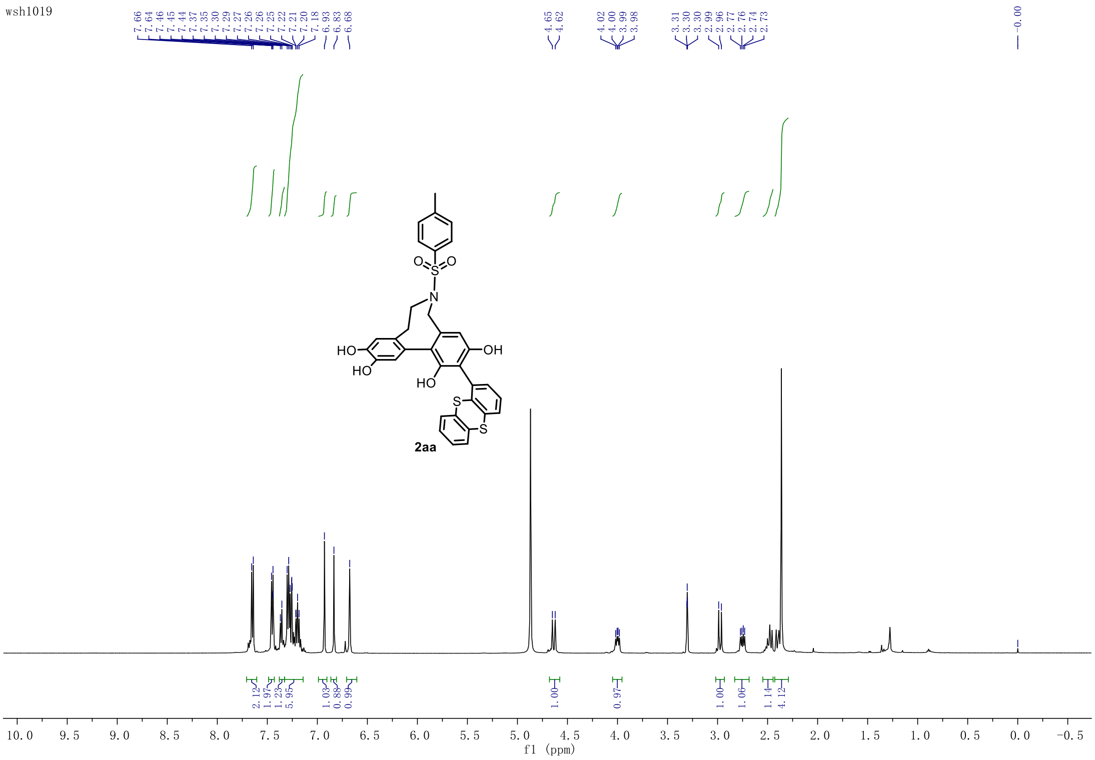

^{13}C NMR spectrum of compound 2aa (125 MHz) in CD_3OD

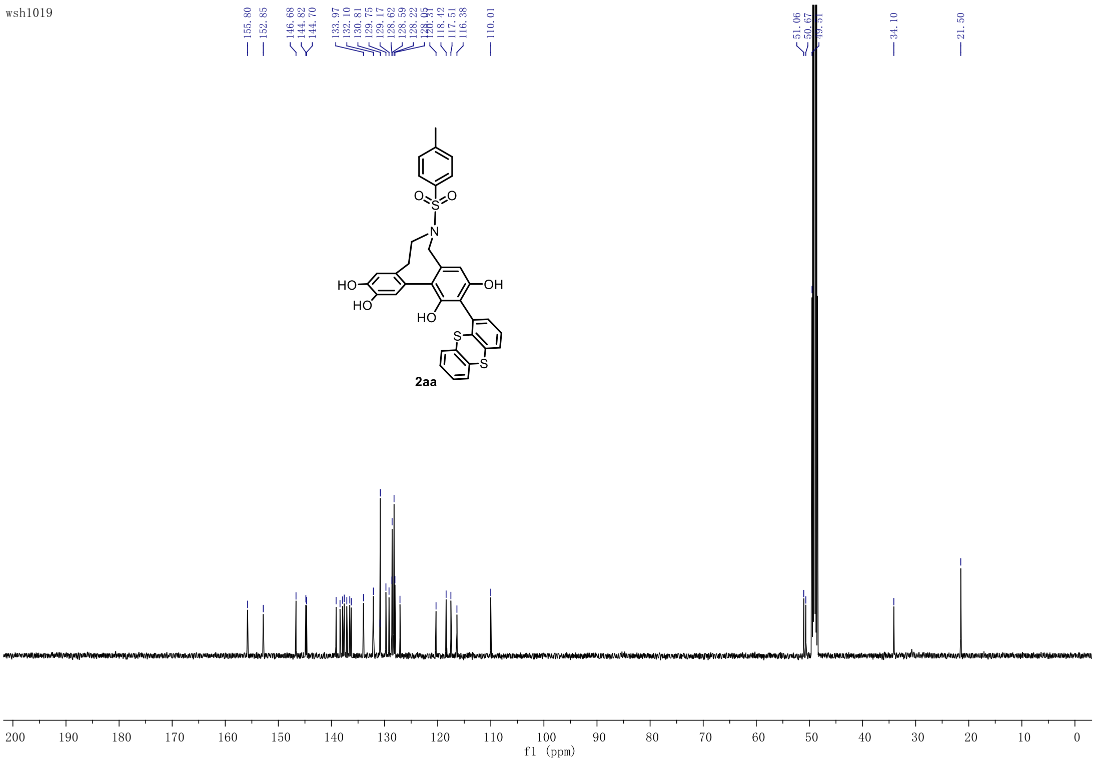

^{1}H NMR spectrum of compound 2ab (500 MHz) in CD_3OD

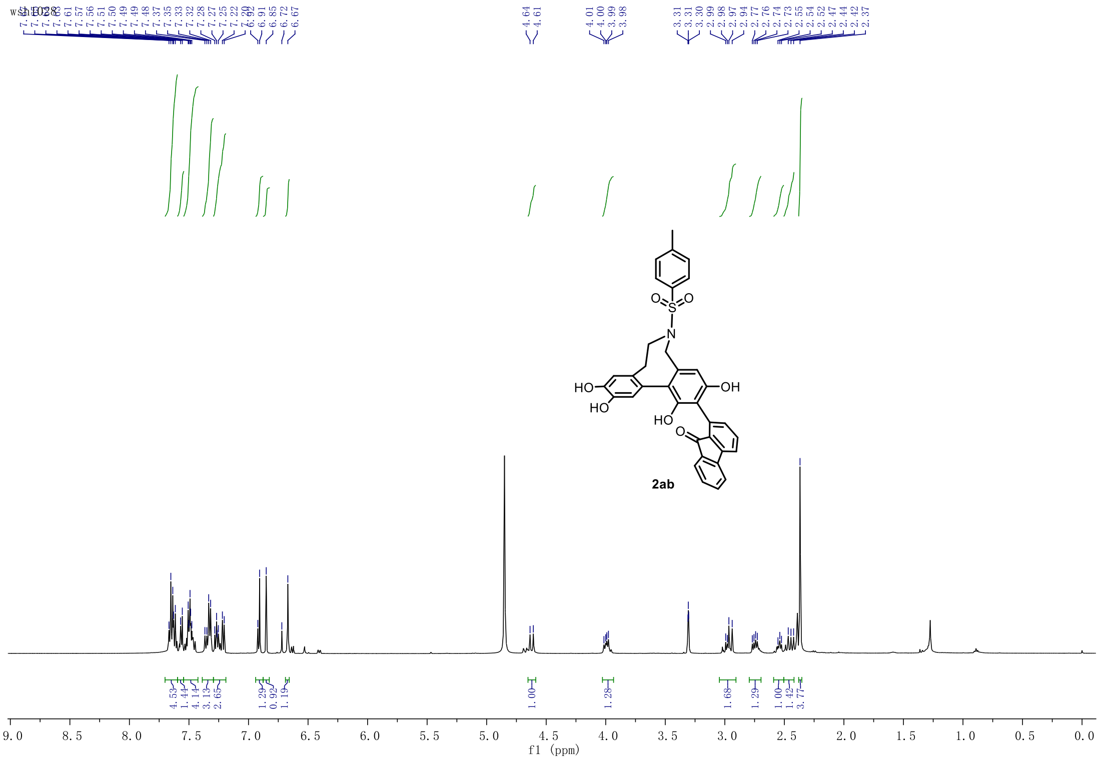

^{13}C NMR spectrum of compound 2ab (125 MHz) in CD_3OD

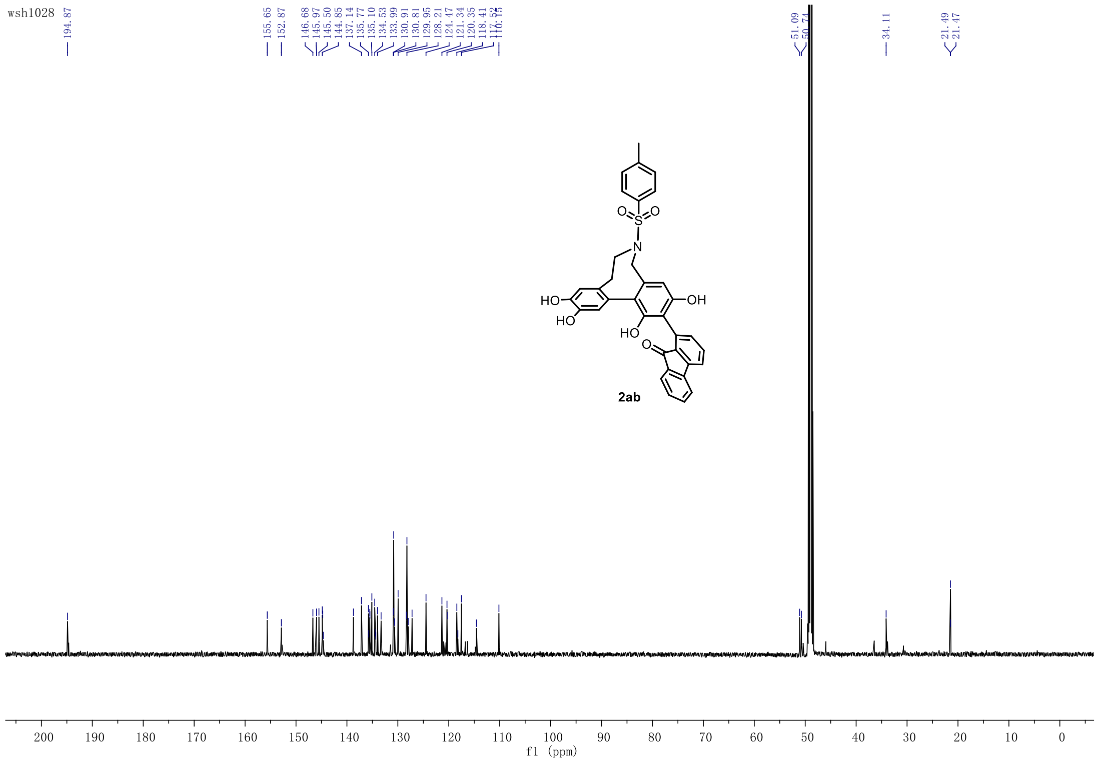

^{1}H NMR spectrum of compound 2ac (500 MHz) in CD_3OD

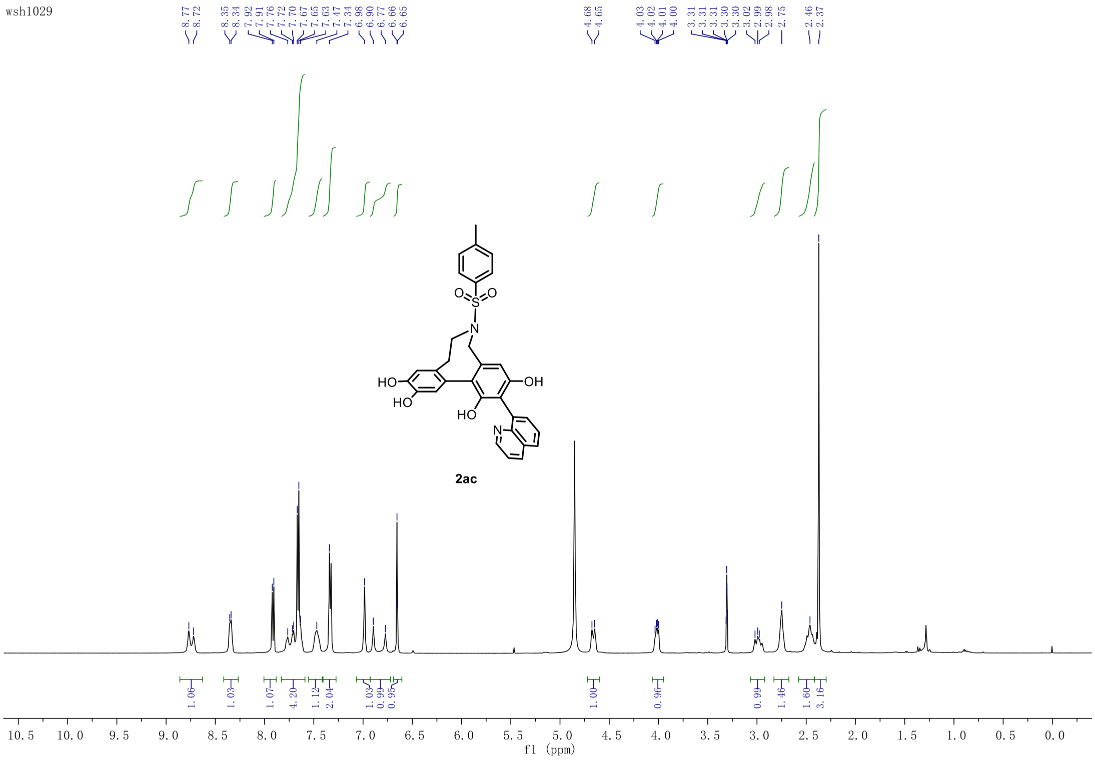

^{13}C NMR spectrum of compound 2ac (125 MHz) in CD_3OD

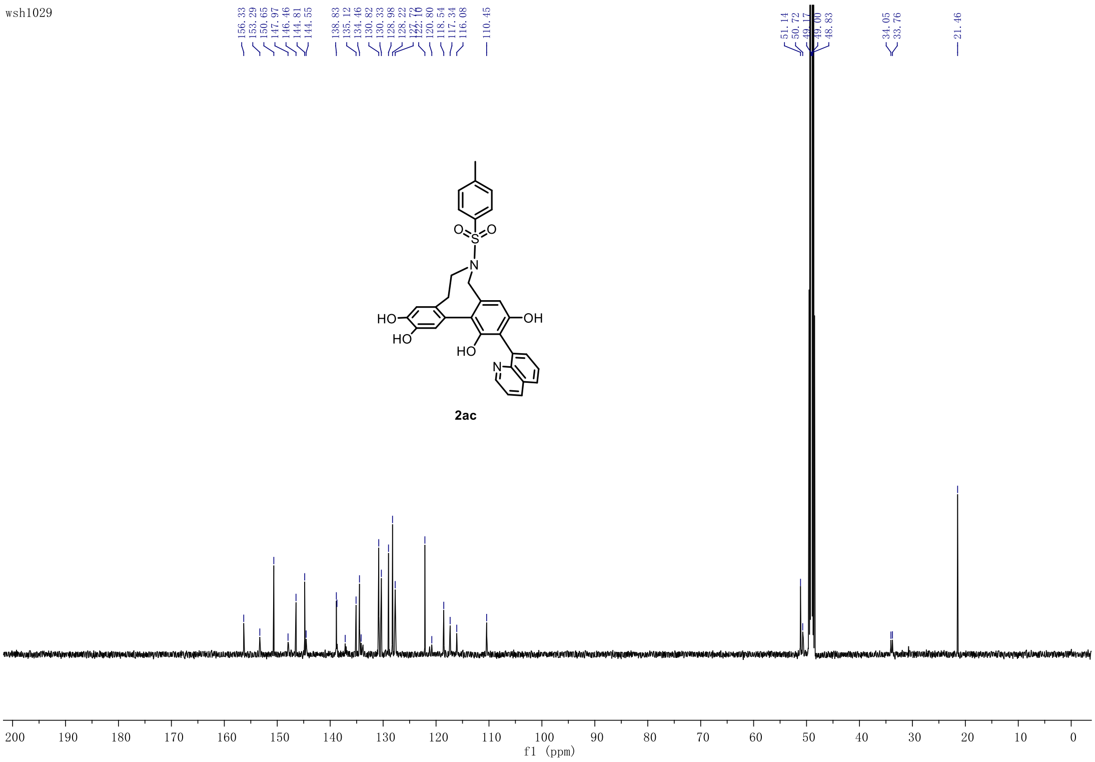

^{1}H NMR spectrum of compound 2ad (500 MHz) in Acetone-d_6

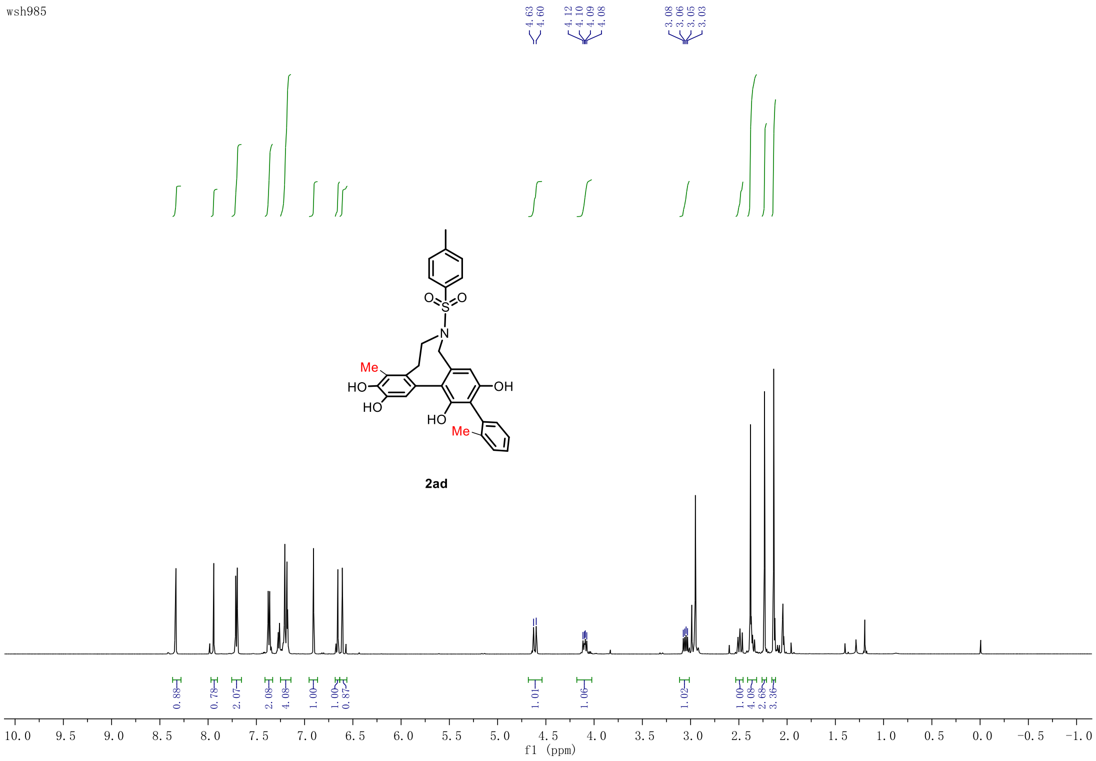

^{13}C NMR spectrum of compound 2ad (125 MHz) in Acetone-d_6

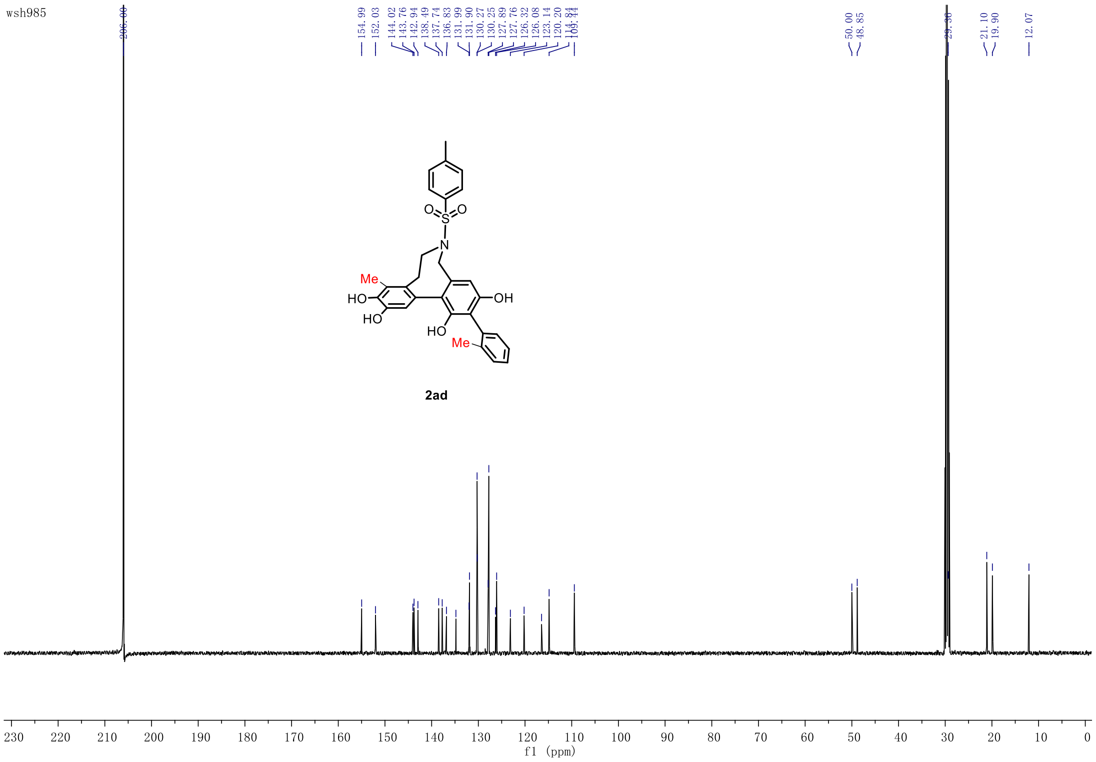

^{1}H NMR spectrum of compound 2ae (500 MHz) in Acetone-*d*$_6$

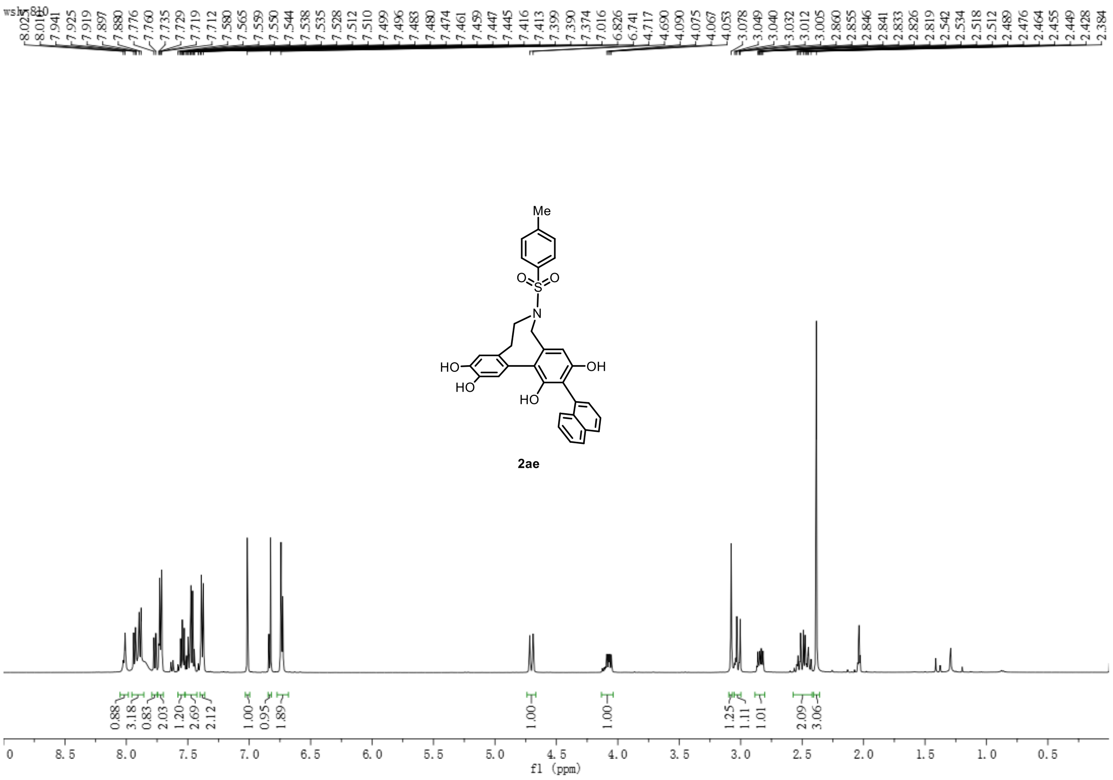

^{13}C NMR spectrum of compound 2ae (125 MHz) in Acetone-*d*$_6$

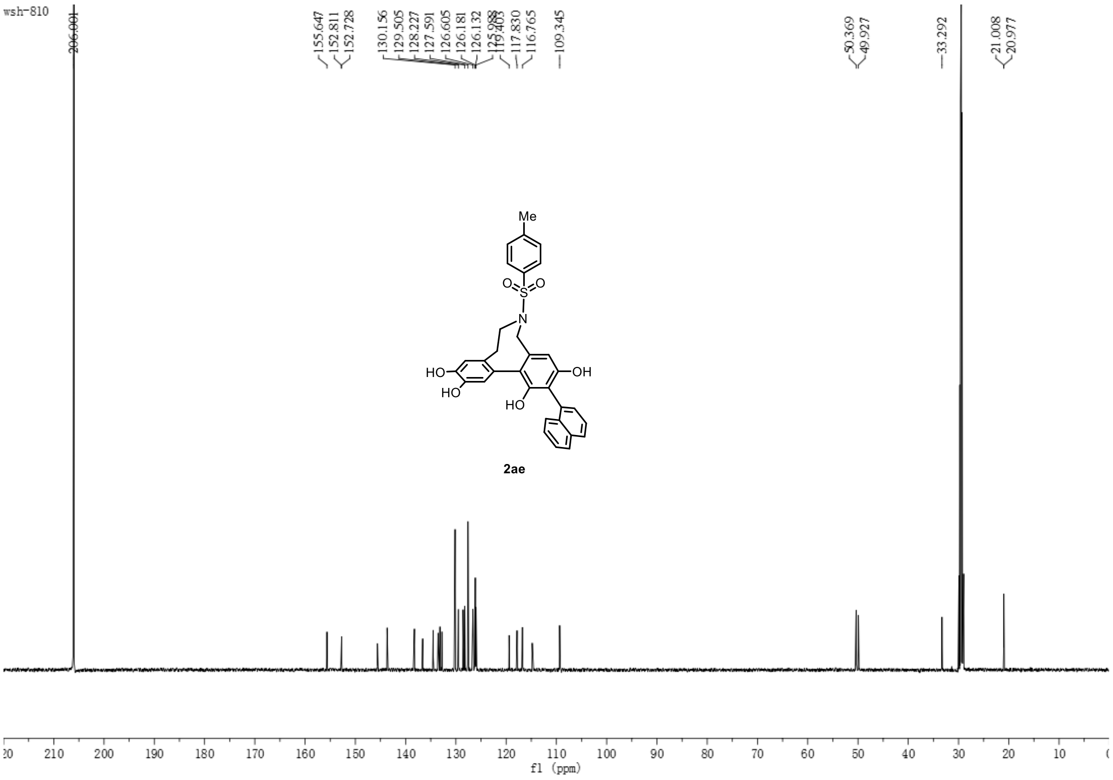

^{1}H NMR spectrum of compound 2af (500 MHz) in Acetone-d_6

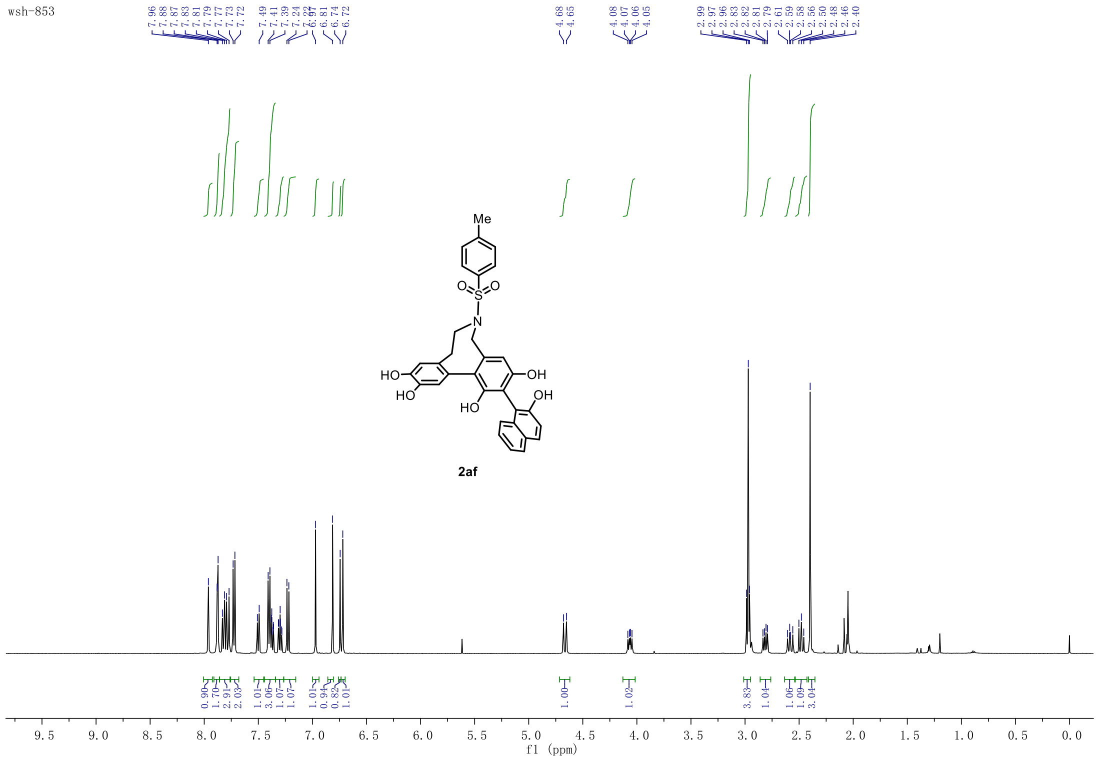

^{13}C NMR spectrum of compound 2af (125 MHz) in Acetone-d_6

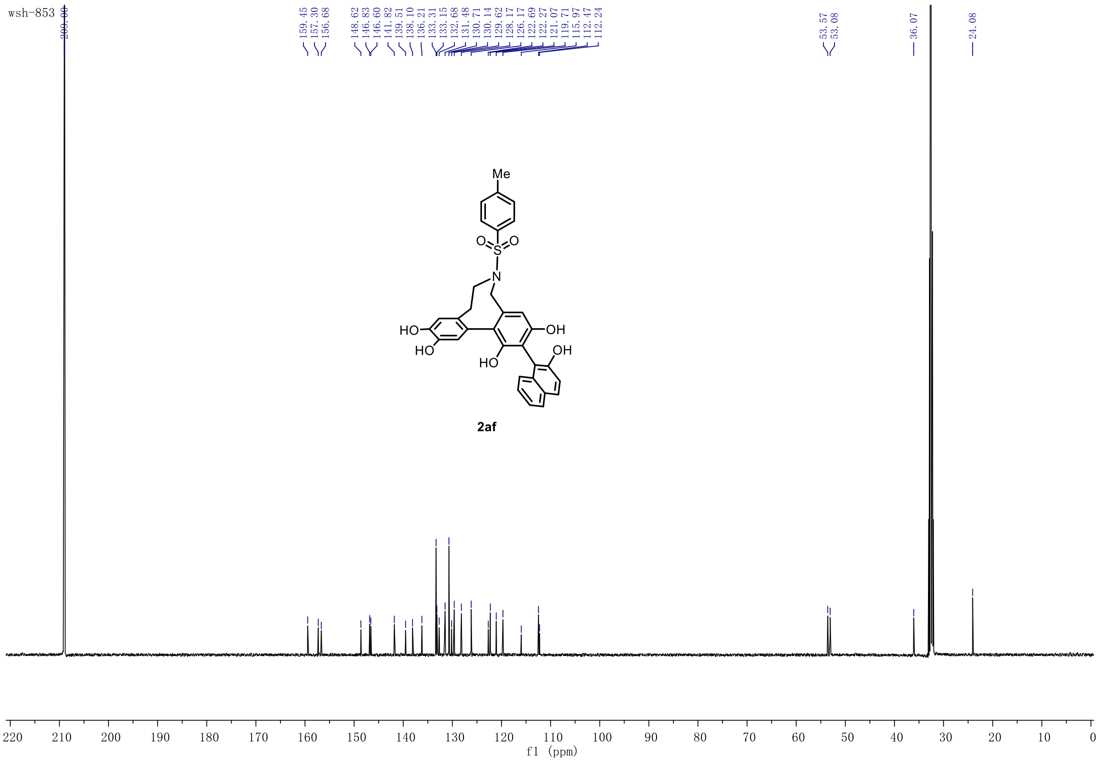

^{1}H NMR spectrum of compound 2ag (500 MHz) in CD_3OD

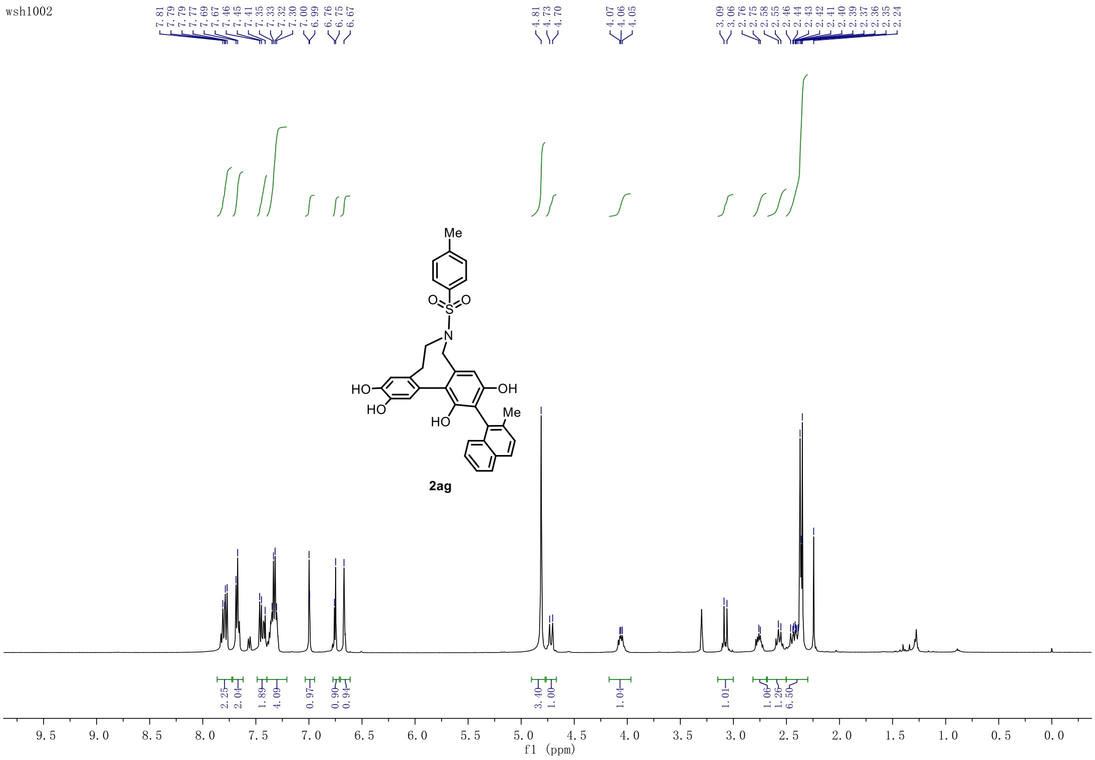

^{13}C NMR spectrum of compound 2ag (125 MHz) in CD_3OD

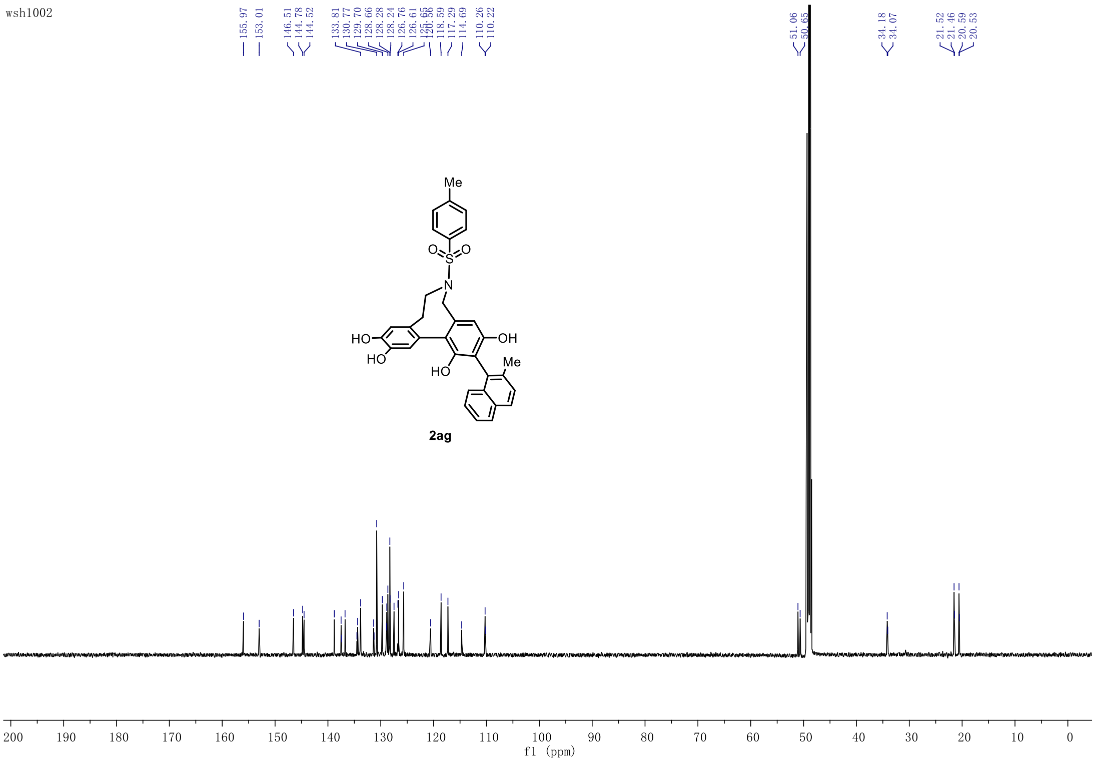

^{1}H NMR spectrum of compound 2ah (500 MHz) in CD_3OD

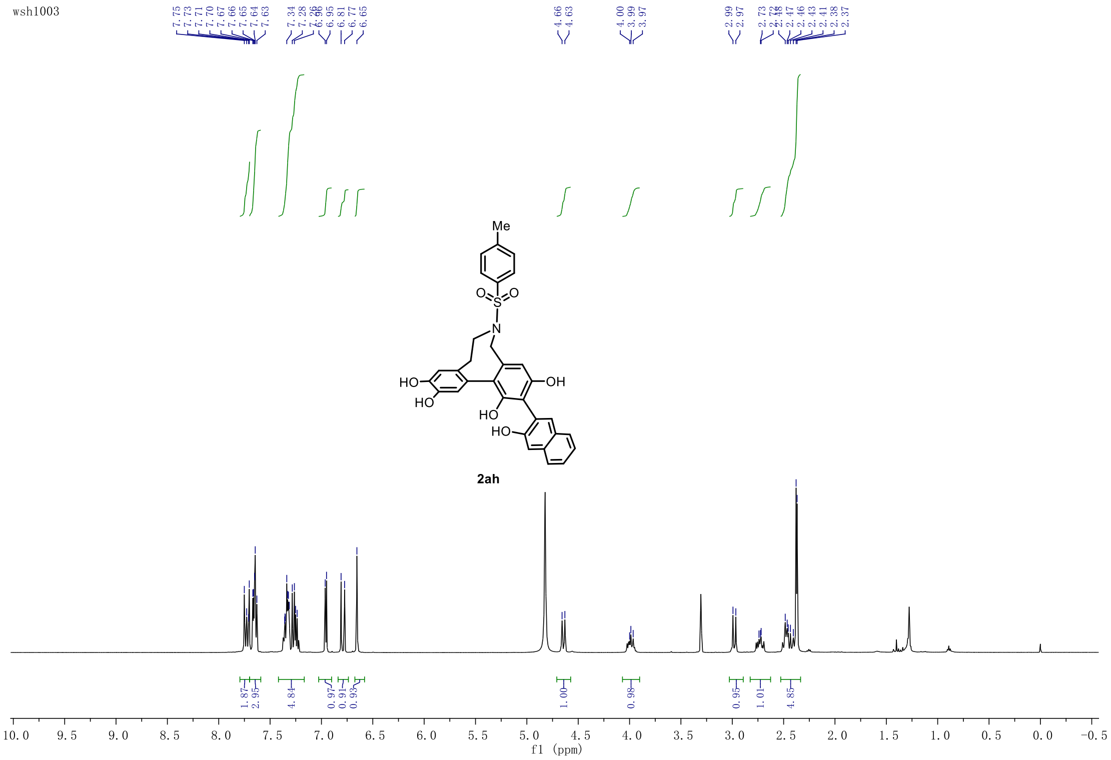

^{13}C NMR spectrum of compound 2ah (125 MHz) in CD_3OD

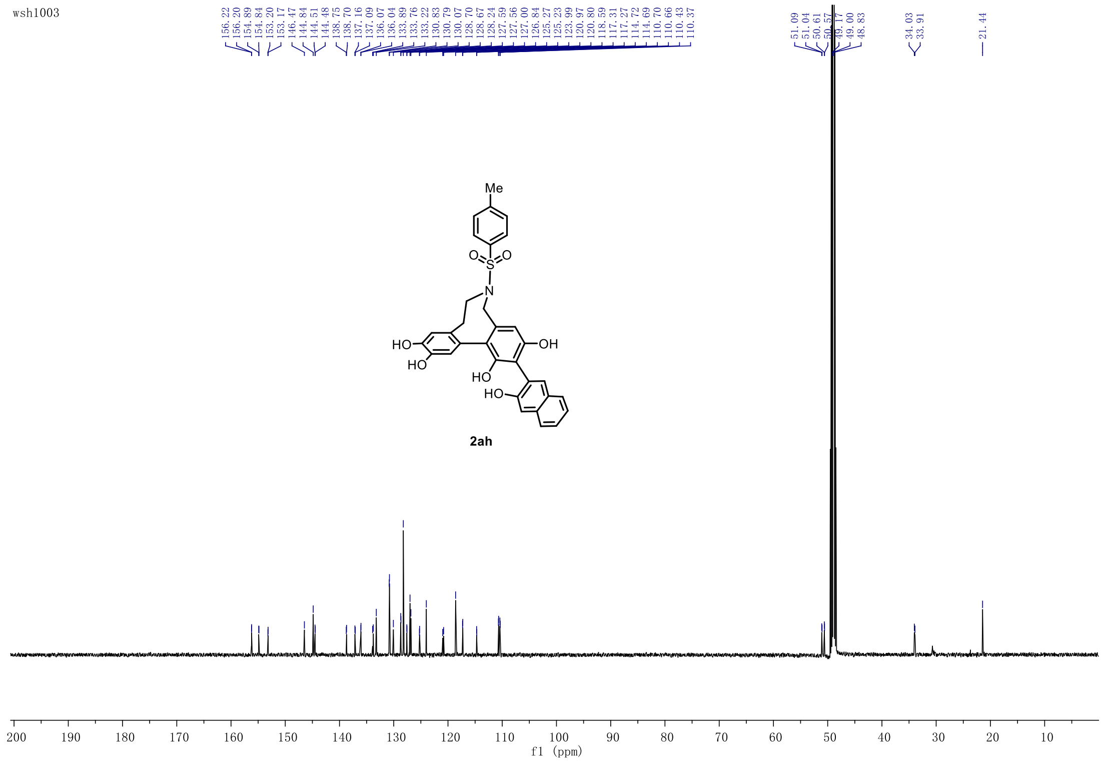

^{1}H NMR spectrum of compound 2ai (500 MHz) in CD_3OD

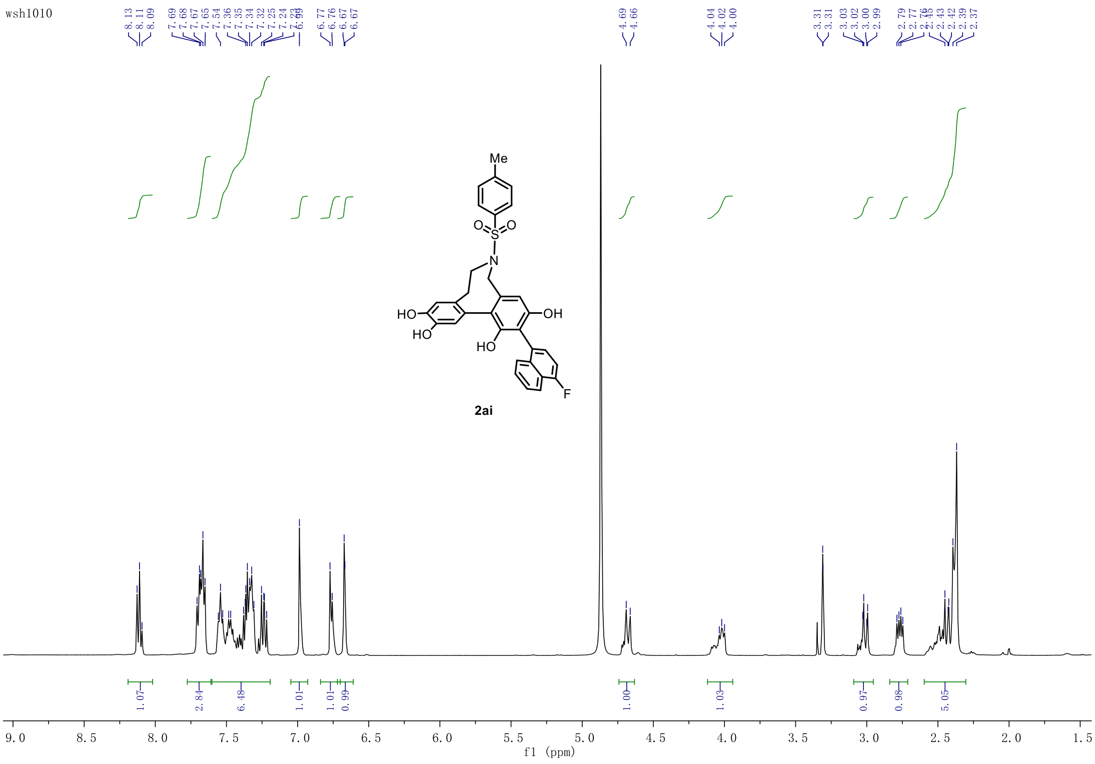

^{13}C NMR spectrum of compound 2ai (125 MHz) in CD_3OD

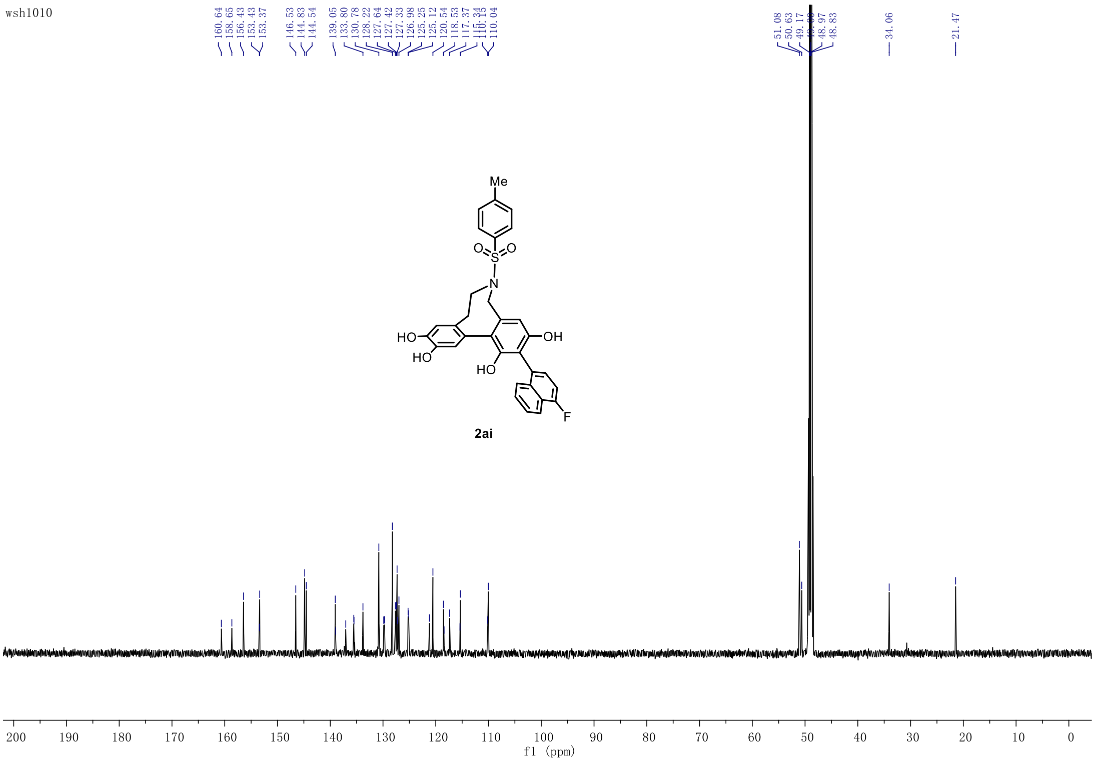

^{19}F NMR spectrum of compound 2ai (470 MHz) in CD_3OD

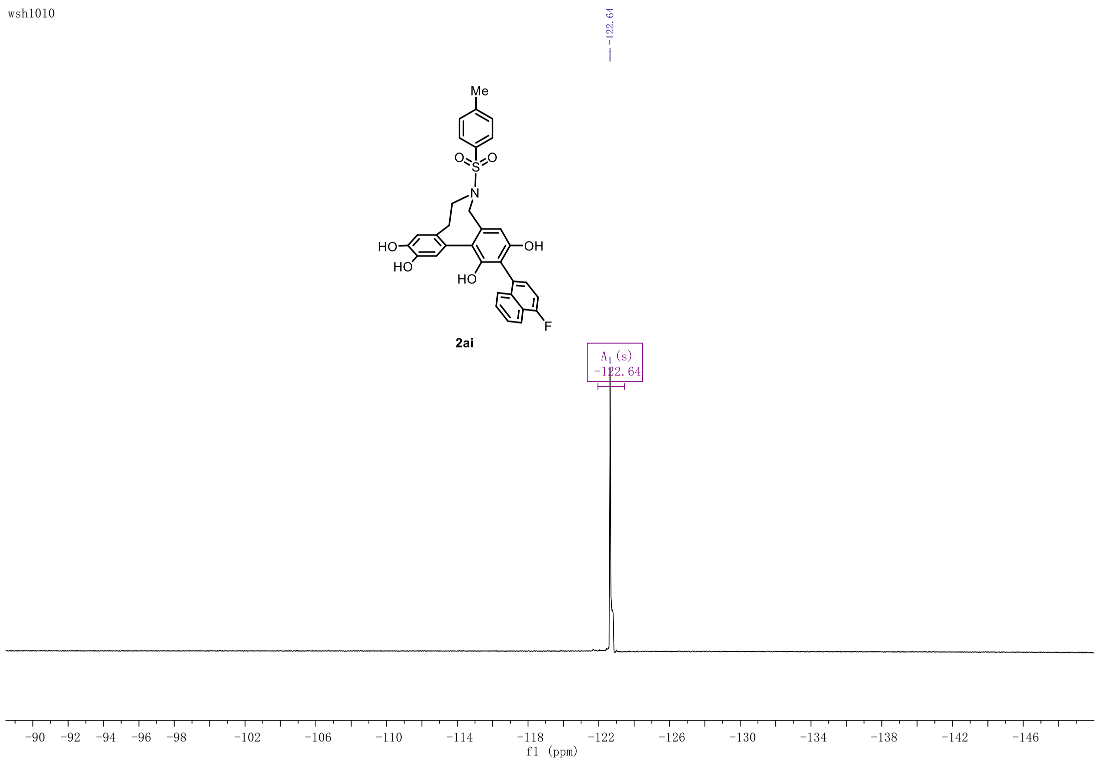

^{1}H NMR spectrum of compound 2aj (500 MHz) in Acetone-d_6

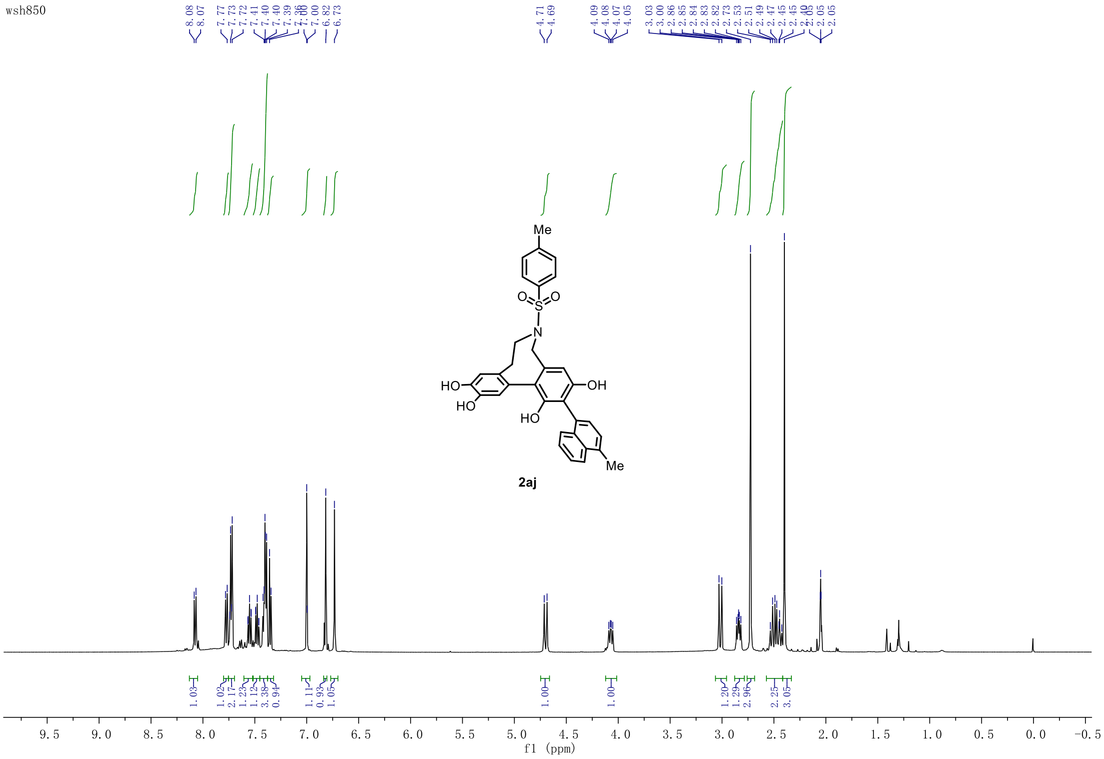

^{13}C NMR spectrum of compound 2aj (125 MHz) in Acetone-d_6

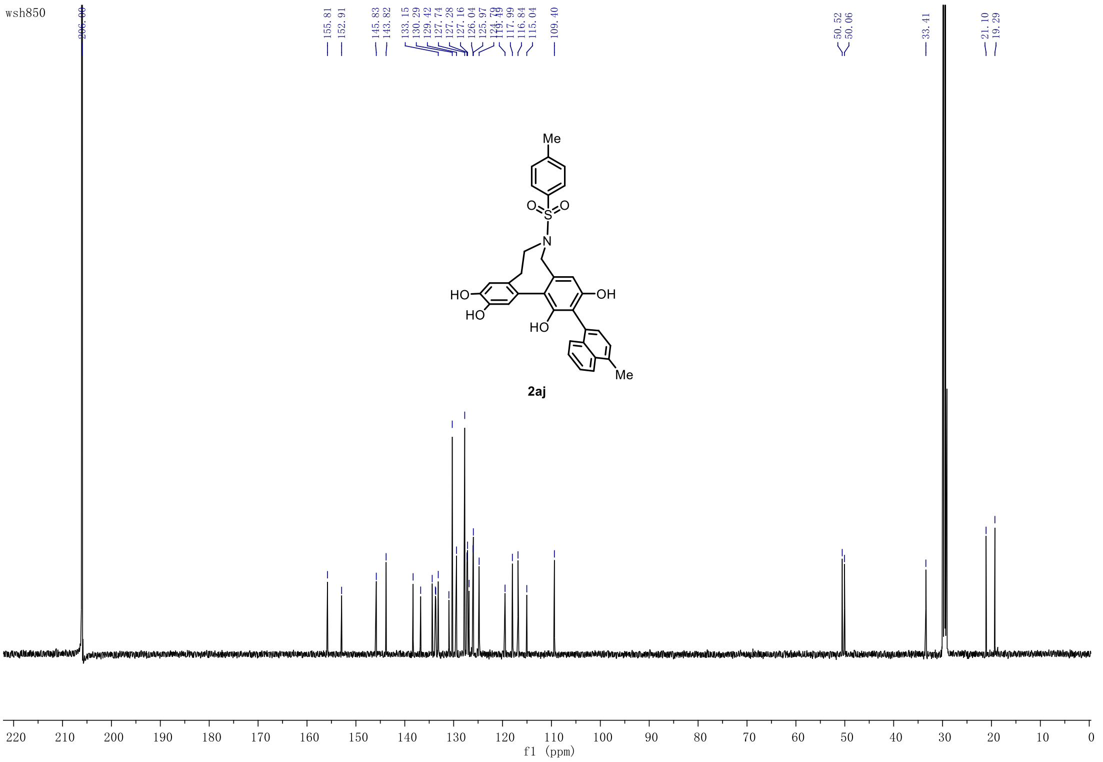

^{1}H NMR spectrum of compound 2ak (500 MHz) in Acetone-d_6

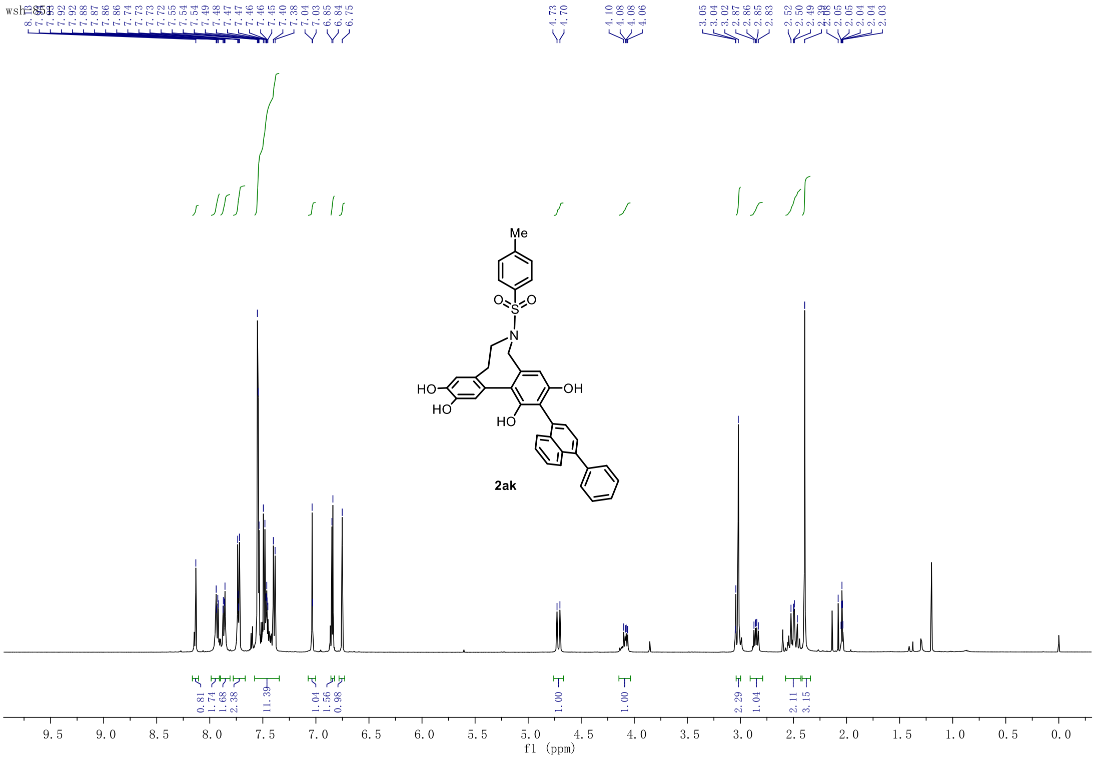

^{13}C NMR spectrum of compound 2ak (125 MHz) in Acetone-d_6

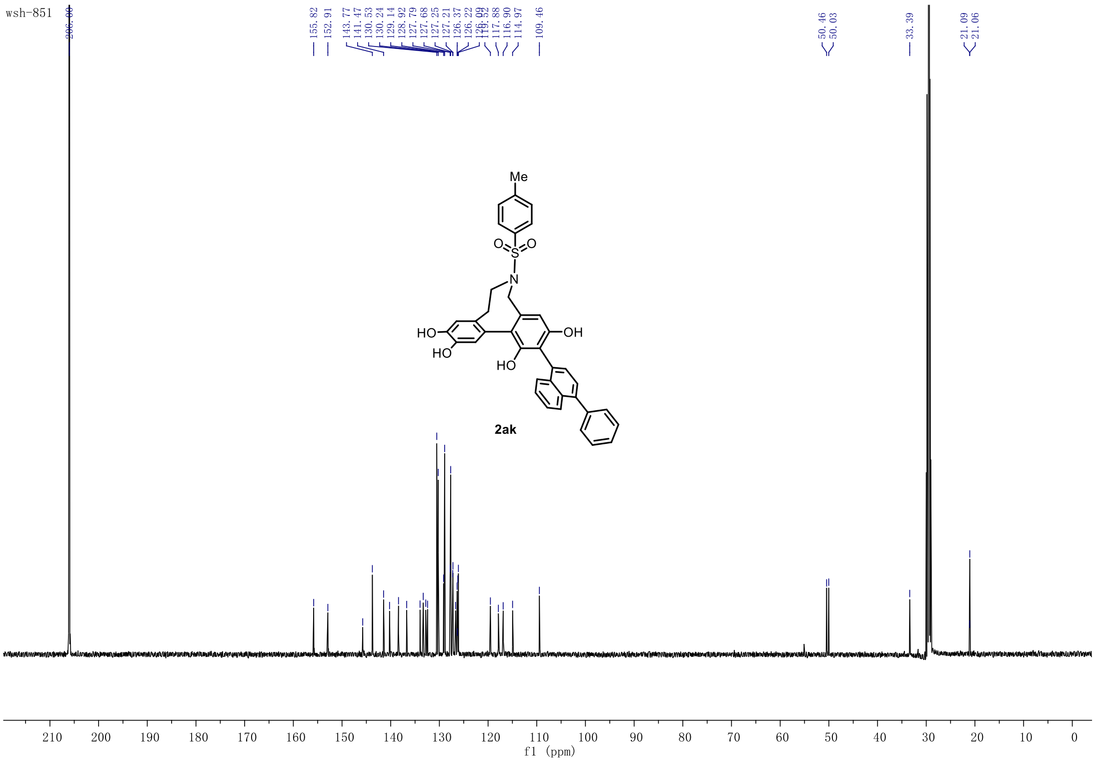

^{1}H NMR spectrum of compound 2al (500 MHz) in CD_3OD

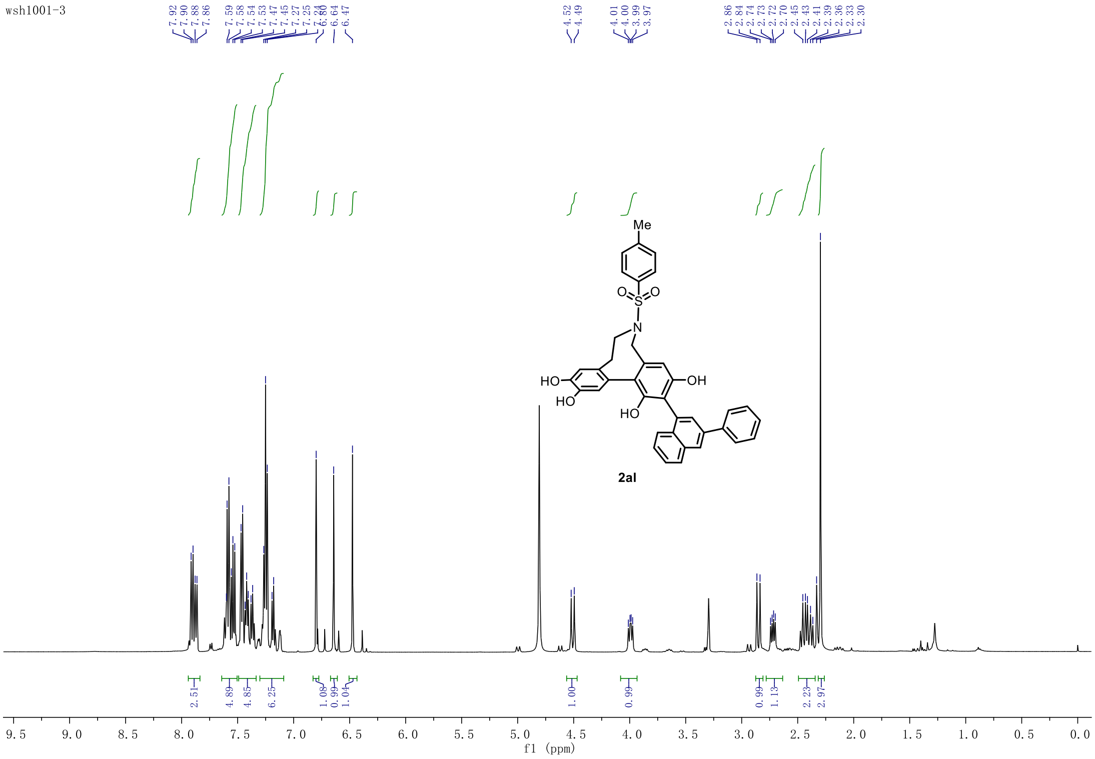

^{13}C NMR spectrum of compound 2al (125 MHz) in CD_3OD

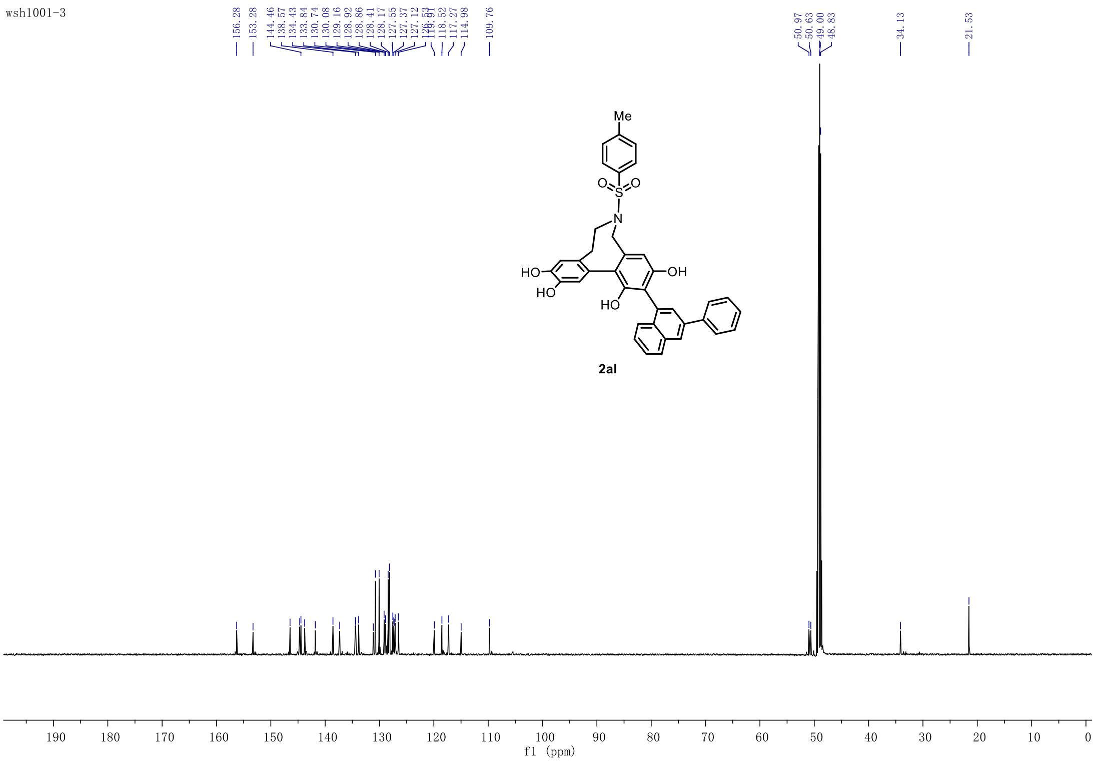

^{1}H NMR spectrum of compound 2am (500 MHz) in CD_3OD

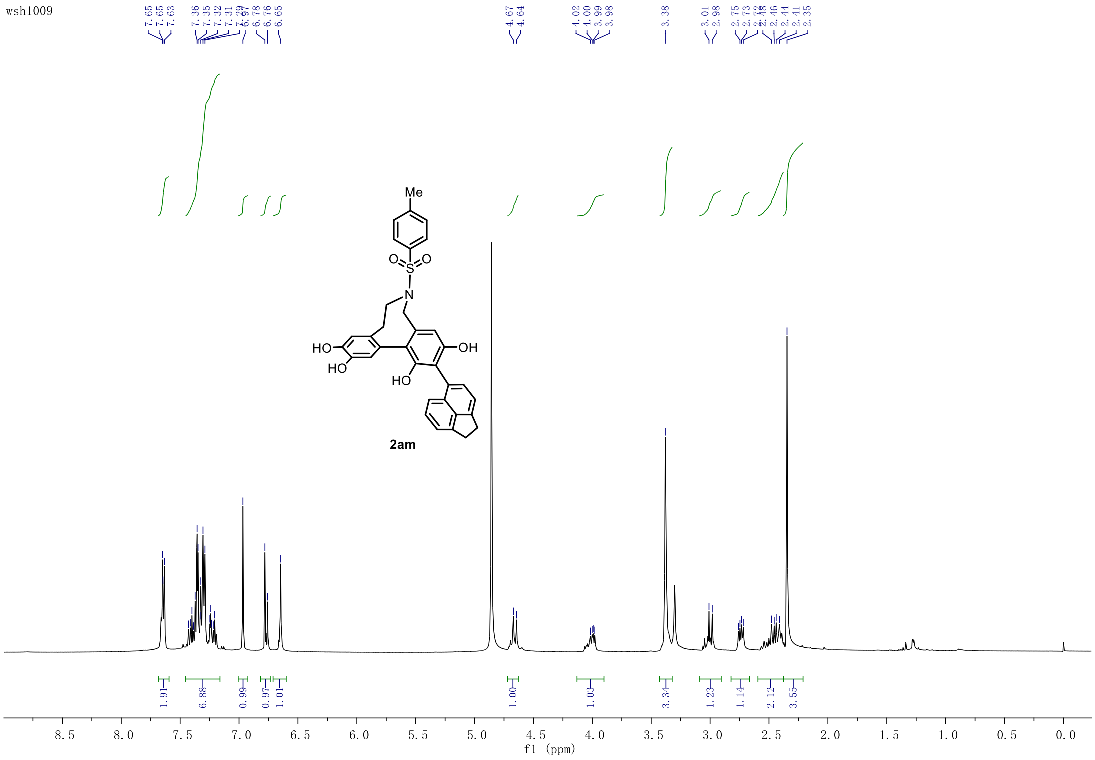

^{13}C NMR spectrum of compound 2am (125 MHz) in CD_3OD

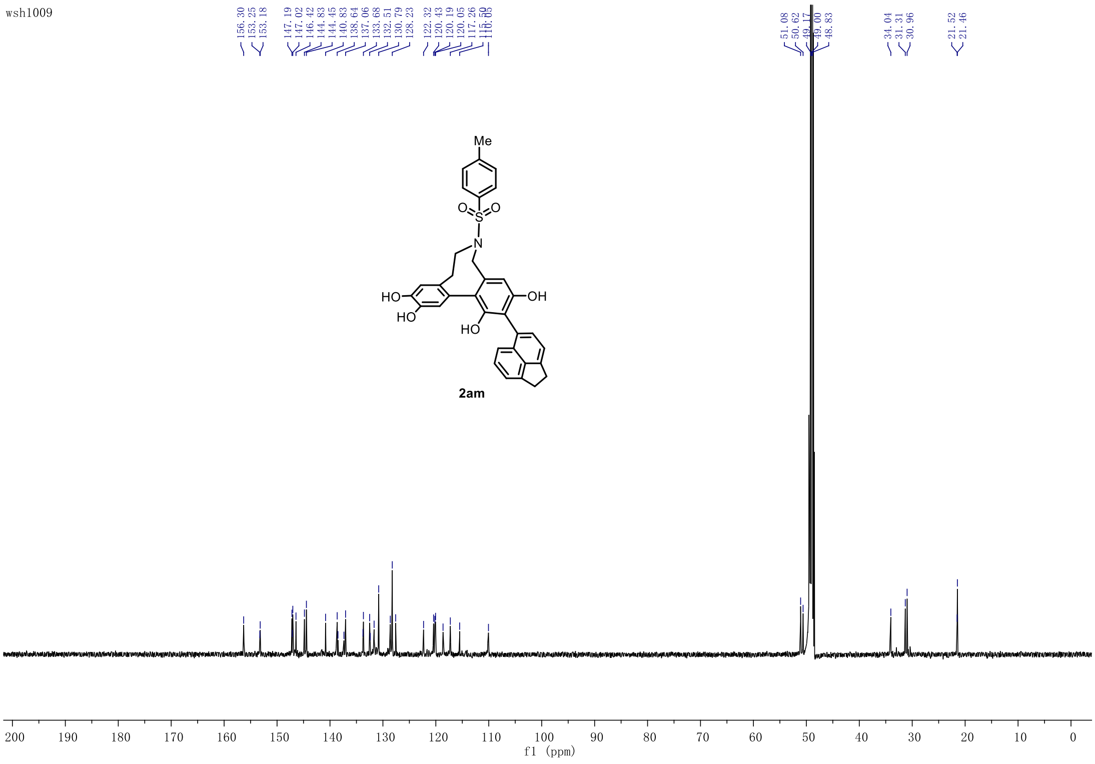

^{1}H NMR spectrum of compound 2an (500 MHz) in CD_3OD

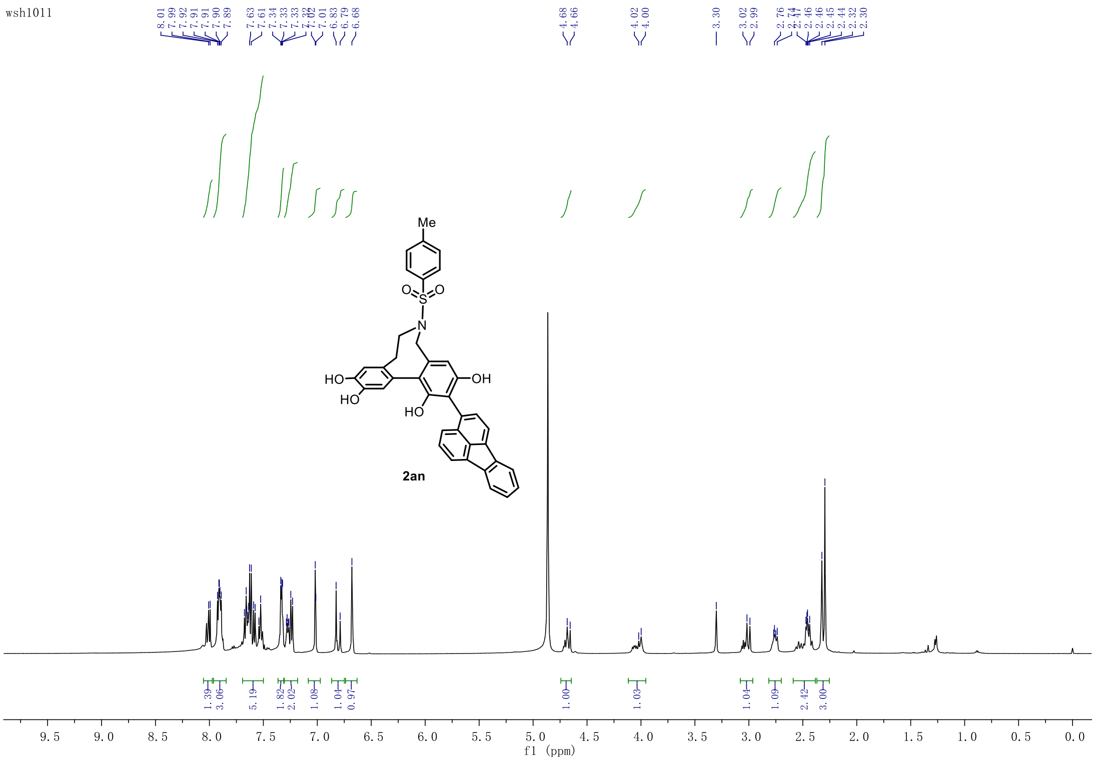

^{13}C NMR spectrum of compound 2an (125 MHz) in CD_3OD

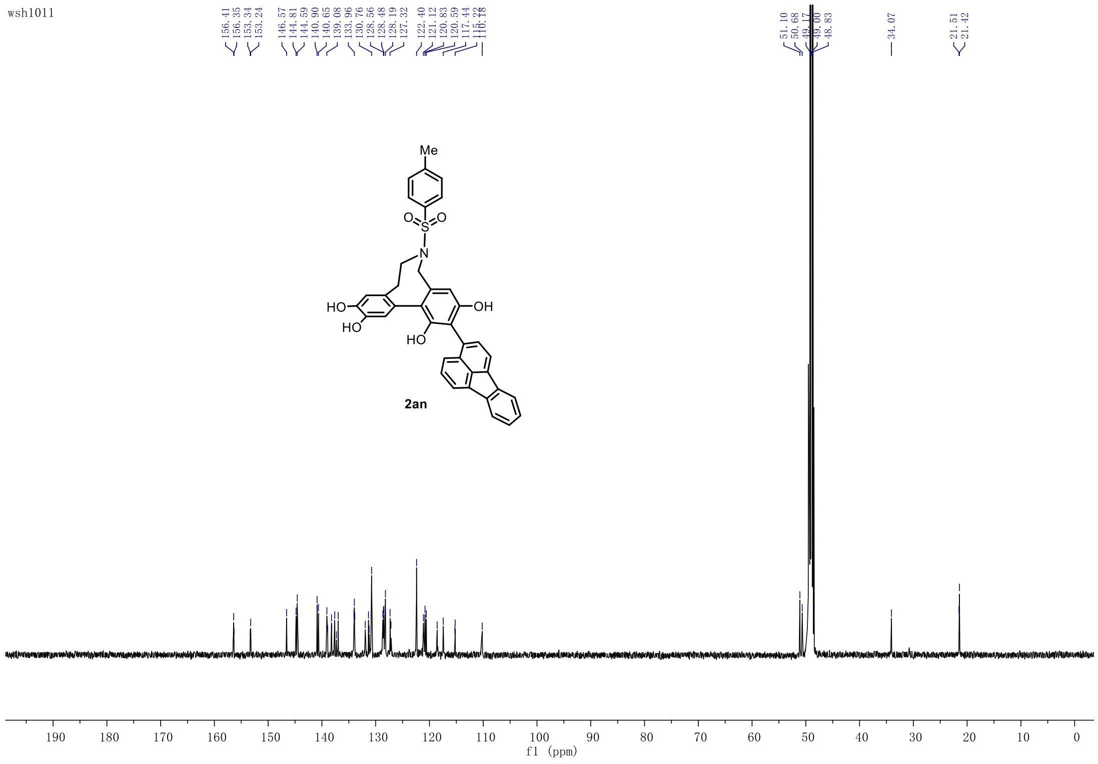

^{1}H NMR spectrum of compound 2ao (500 MHz) in Acetone-d_6

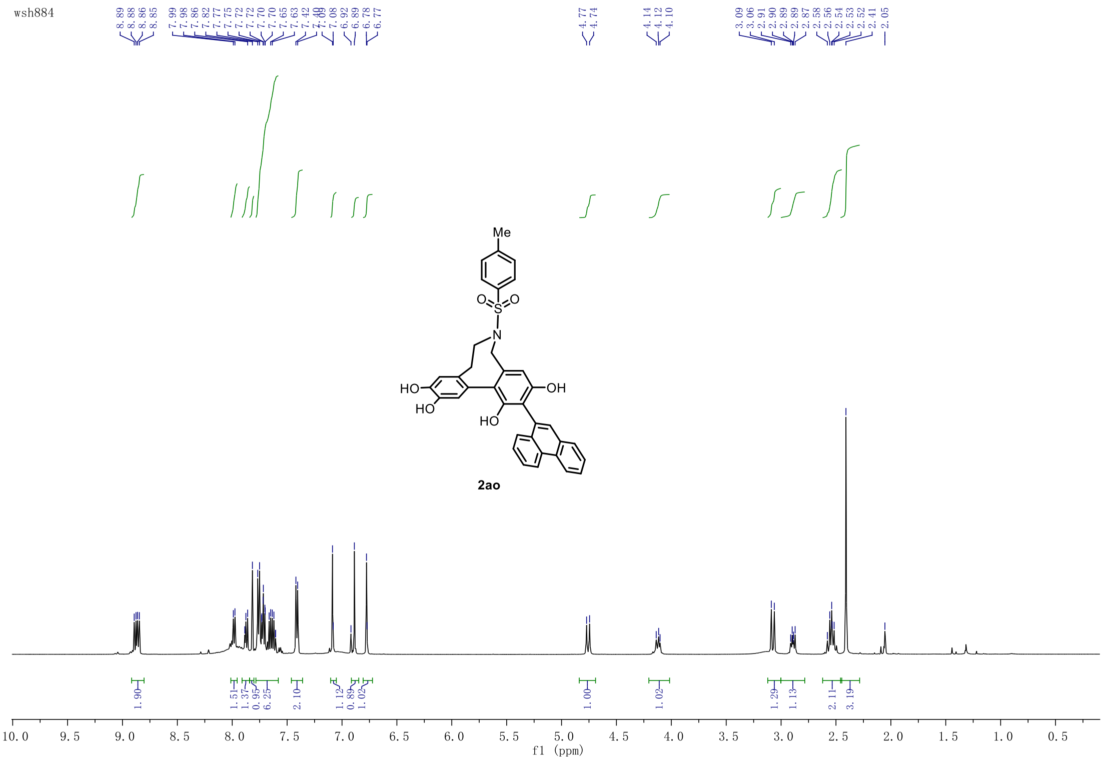

^{13}C NMR spectrum of compound 2ao (125 MHz) in Acetone-d_6

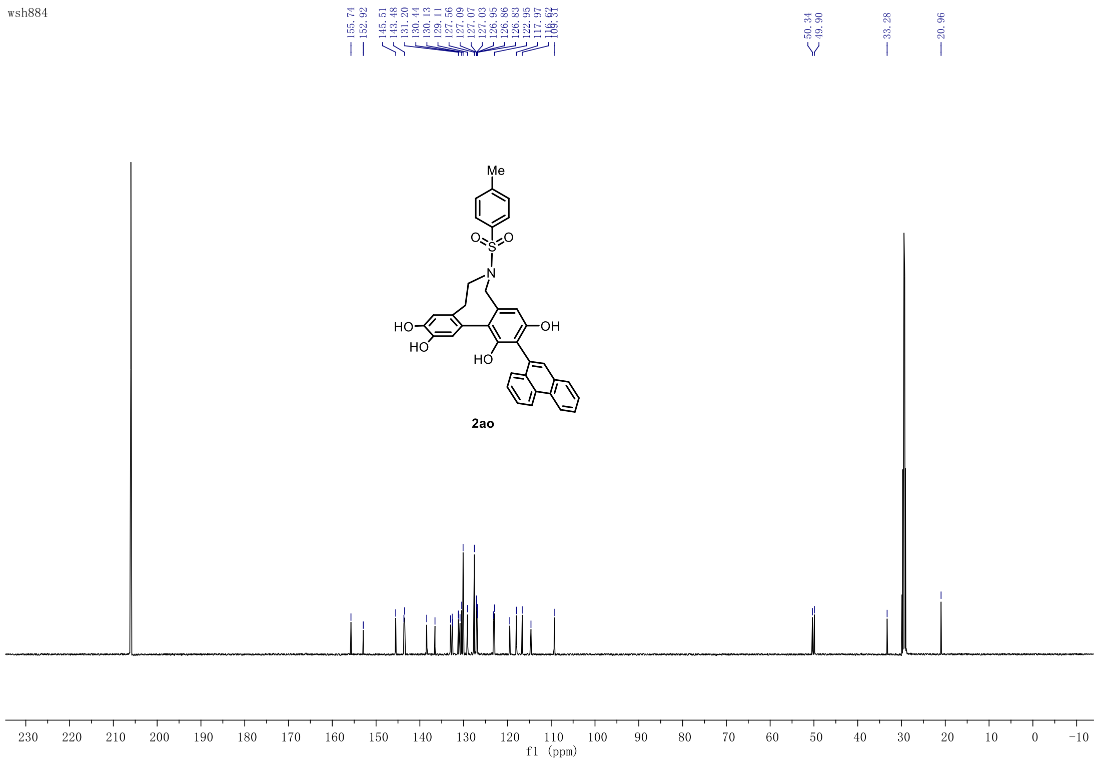

^{1}H NMR spectrum of compound 2ap (500 MHz) in DMSO-*d*$_6$

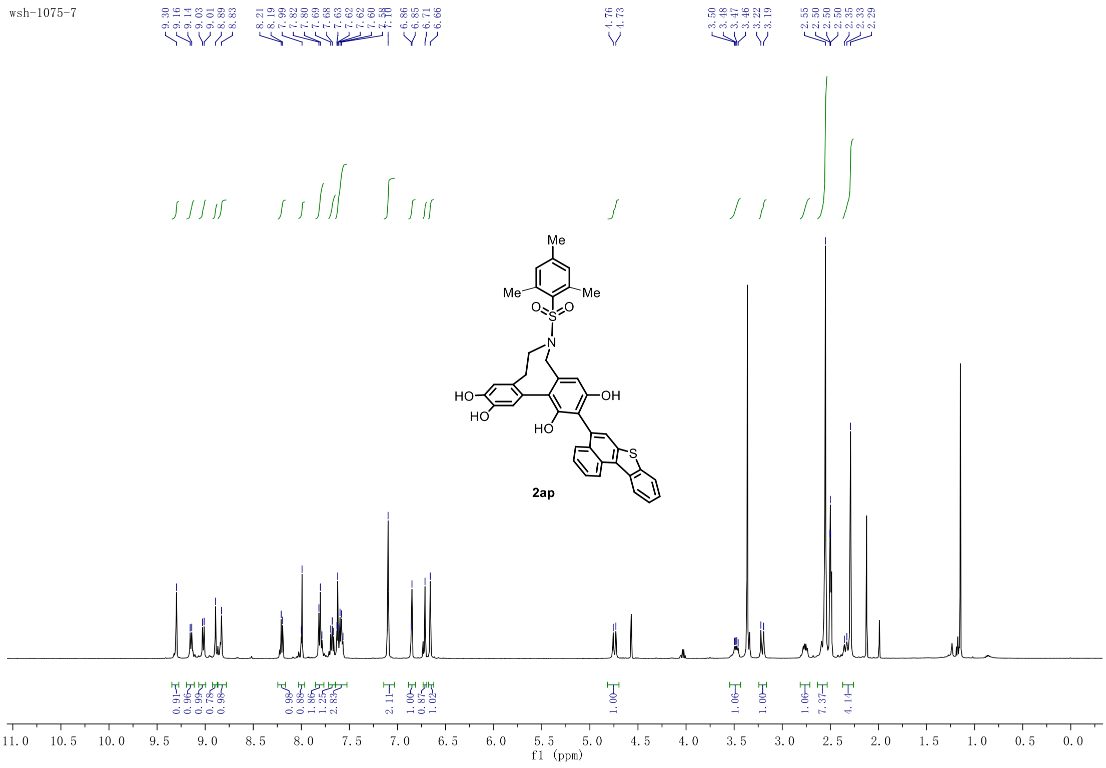

^{13}C NMR spectrum of compound 2ap (125 MHz) in Acetone-*d*$_6$

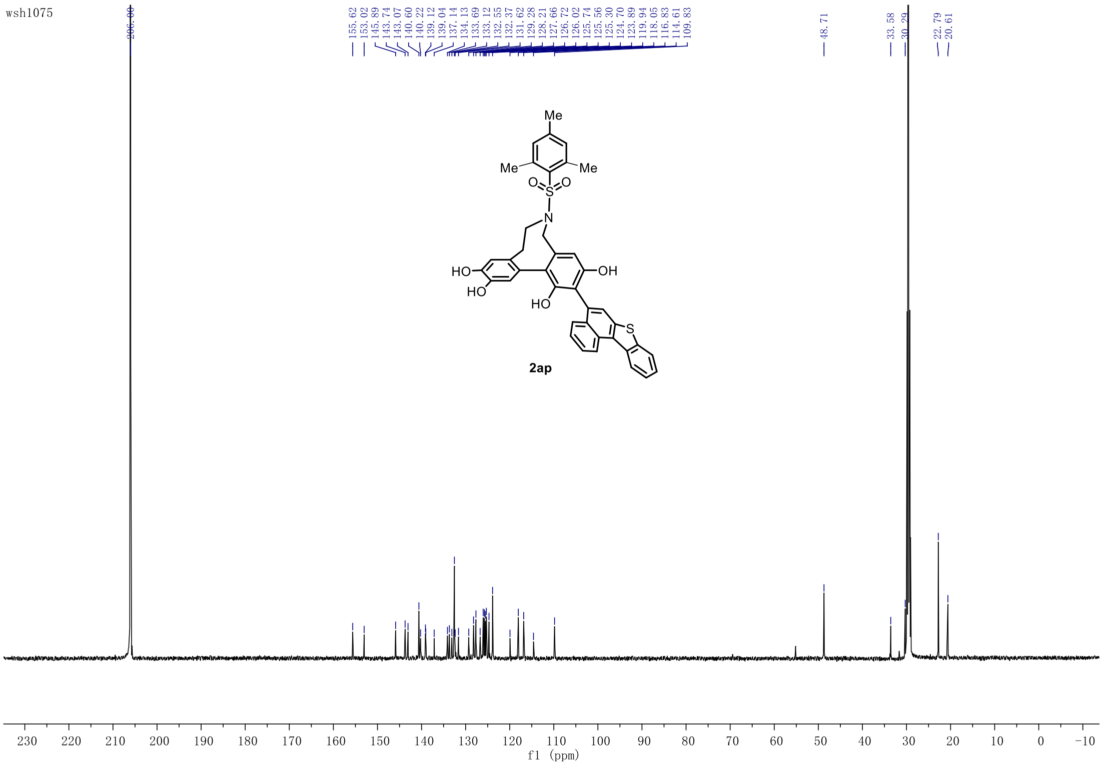

^{1}H NMR spectrum of compound 2aq (500 MHz) in Acetone-*d*$_6$

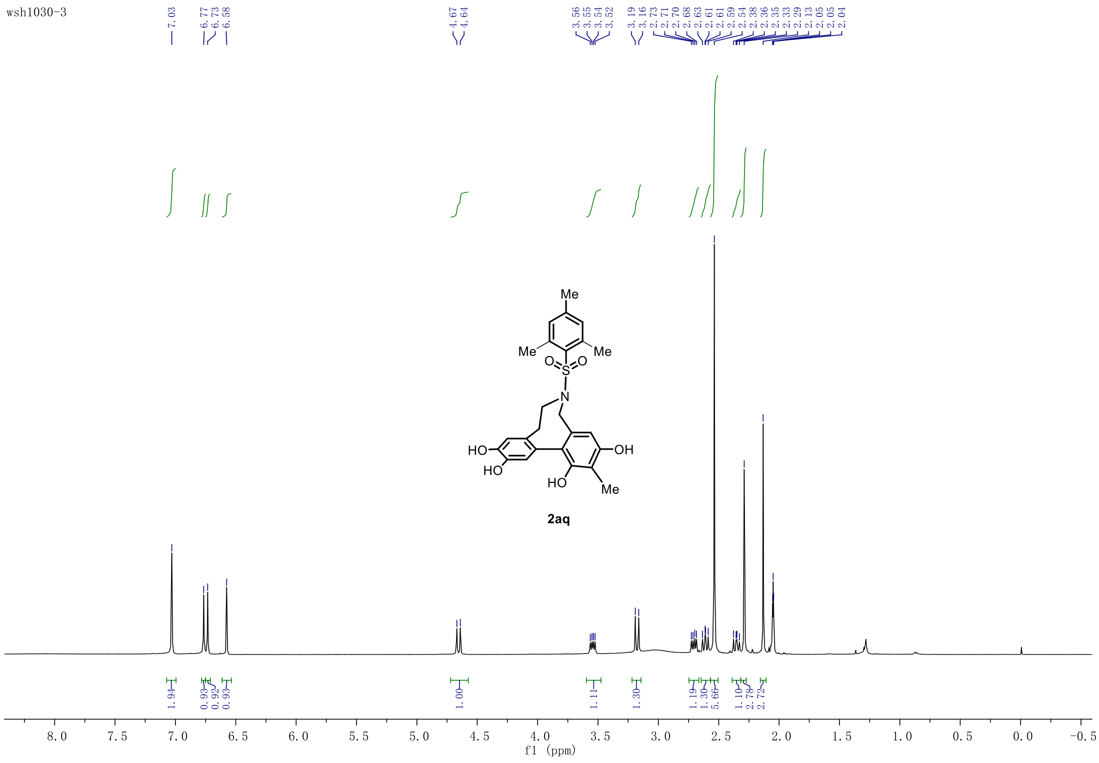

^{13}C NMR spectrum of compound 2aq (125 MHz) in Acetone-*d*$_6$

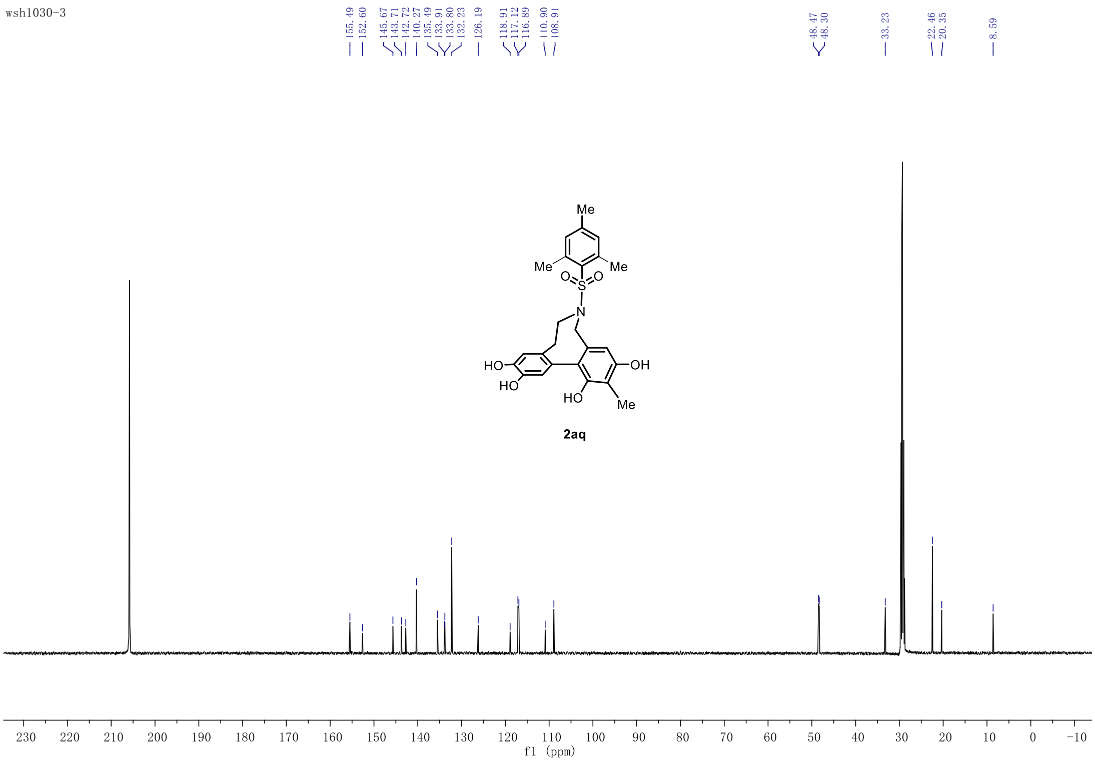

^{1}H NMR spectrum of compound 2ar (500 MHz) in Acetone-d_6

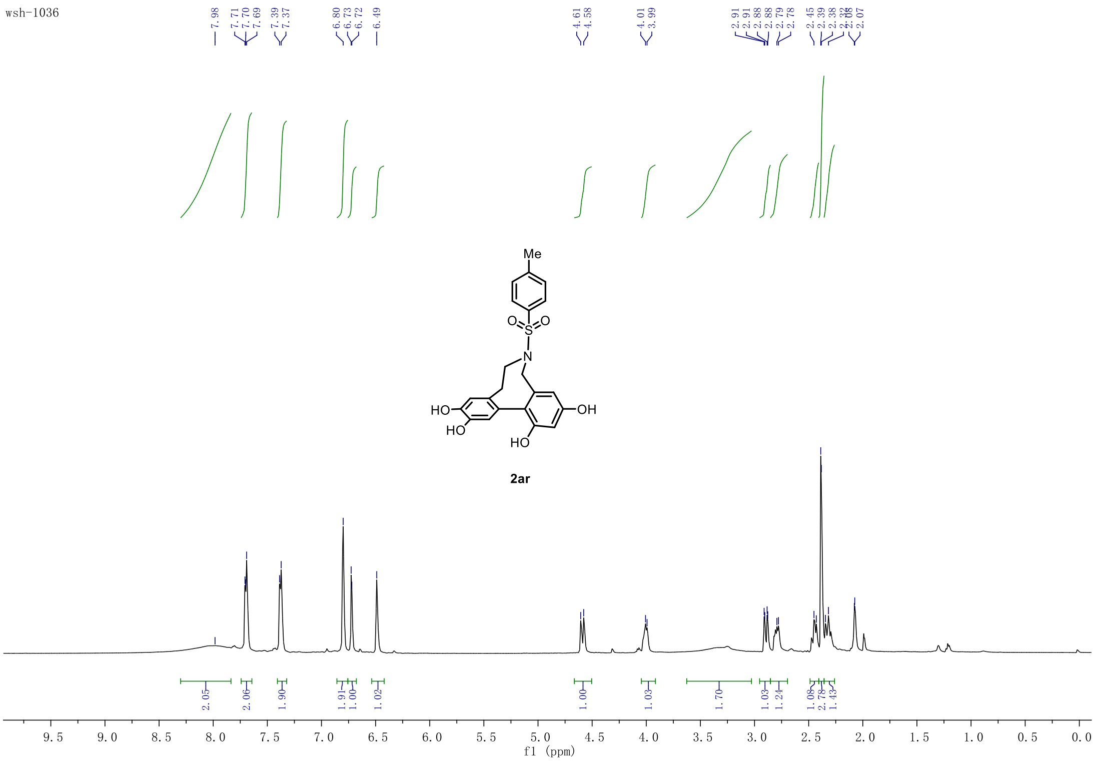

^{13}C NMR spectrum of compound 2ar (125 MHz) in Acetone-d_6

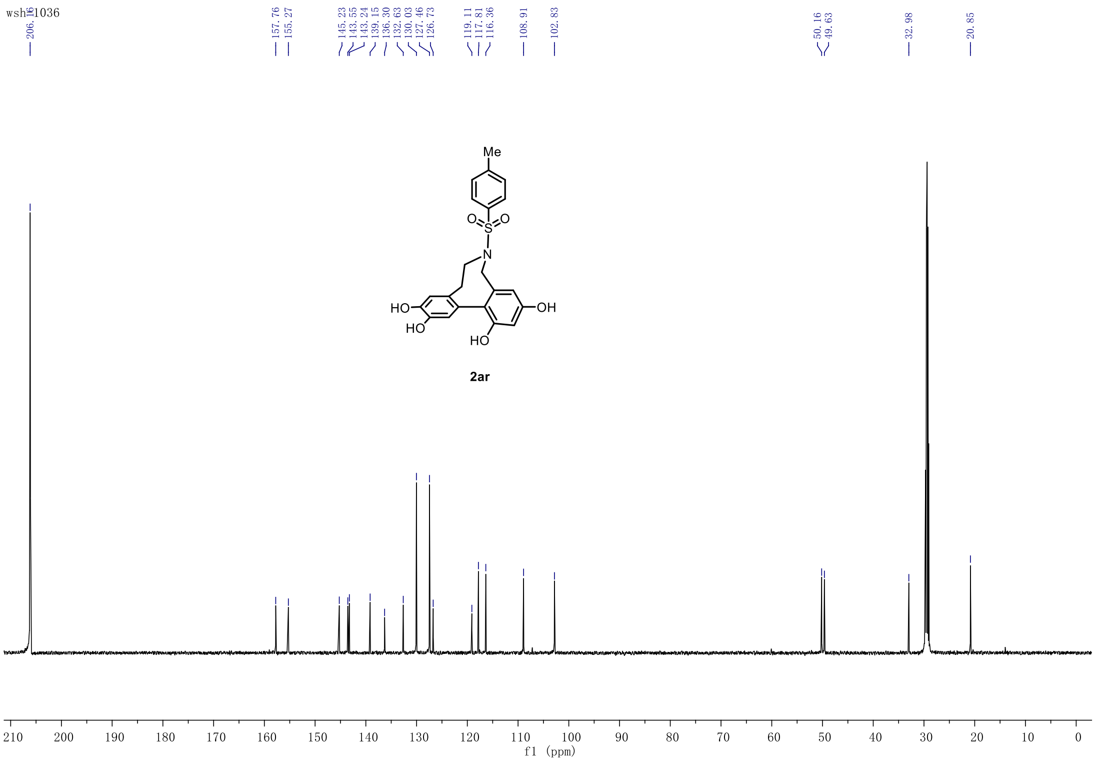

^{1}H NMR spectrum of compound 2as (500 MHz) in Acetone-d_6

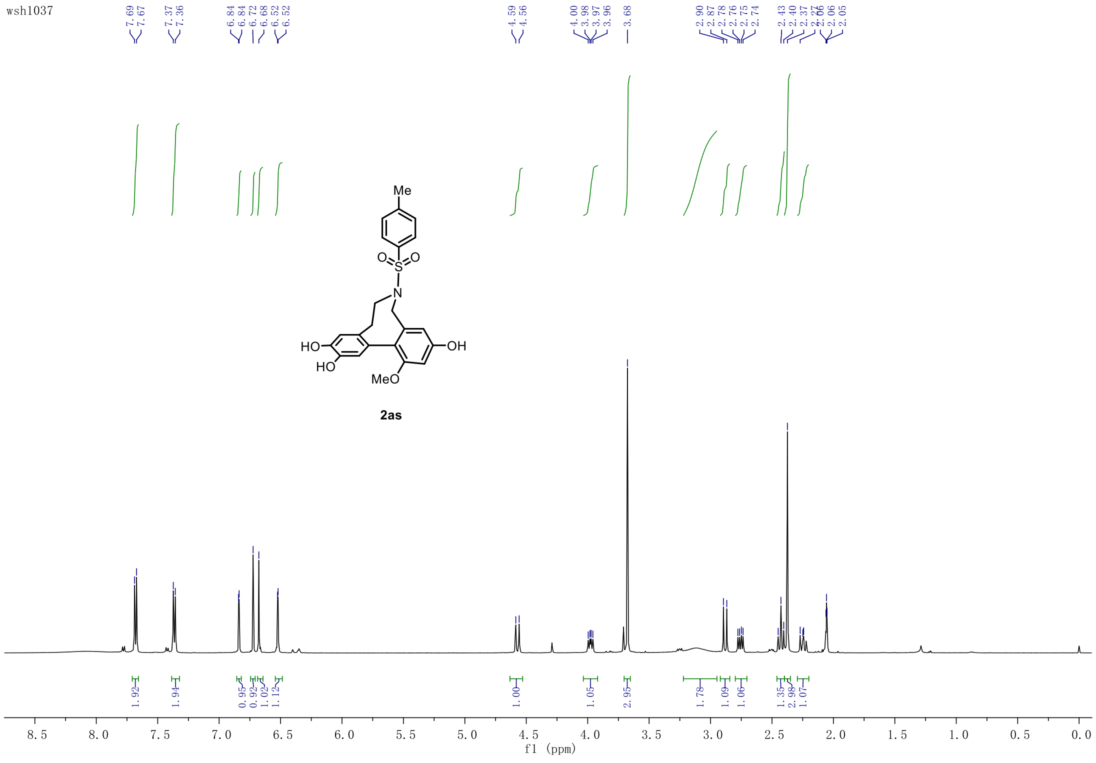

^{13}C NMR spectrum of compound 2as (125 MHz) in Acetone-d_6

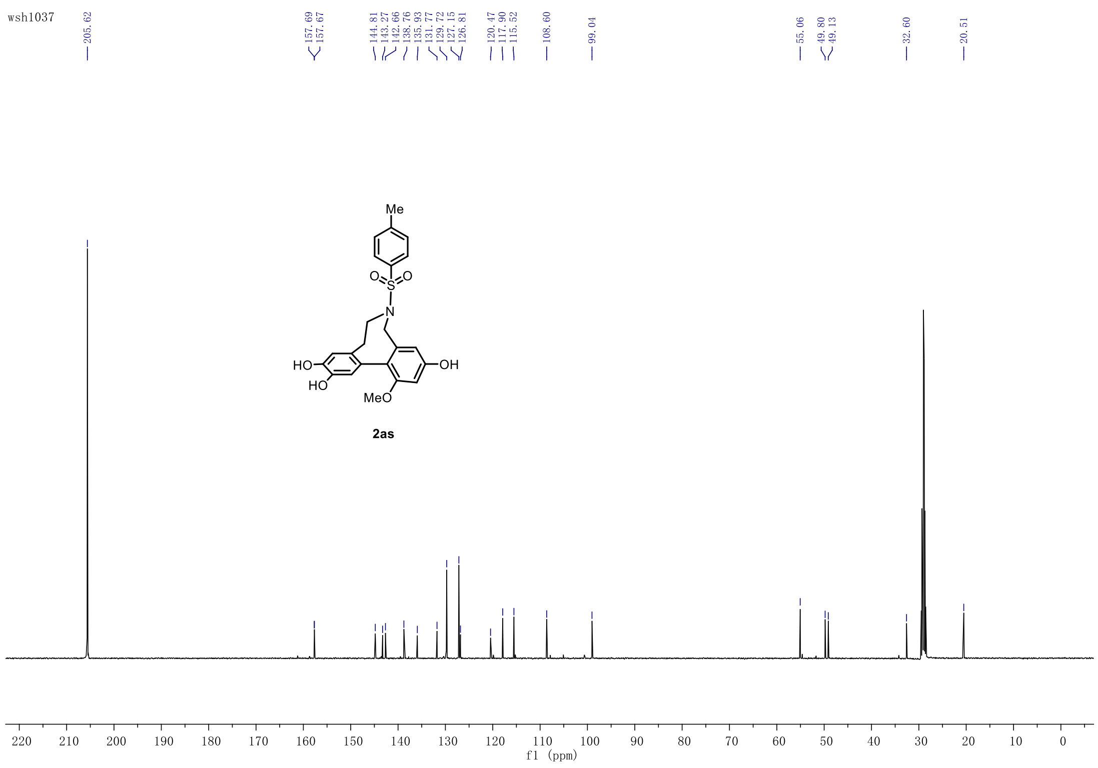
wsh1037
205.62
157.69
157.67
144.81
143.27
142.66
138.76
135.93
131.77
129.72
127.15
126.81
120.47
117.90
115.52
108.60
99.04
55.06
49.80
49.13
32.60
20.51
Me
O
S
O
N
HO
HO
OH
MeO
2as
f1 (ppm)

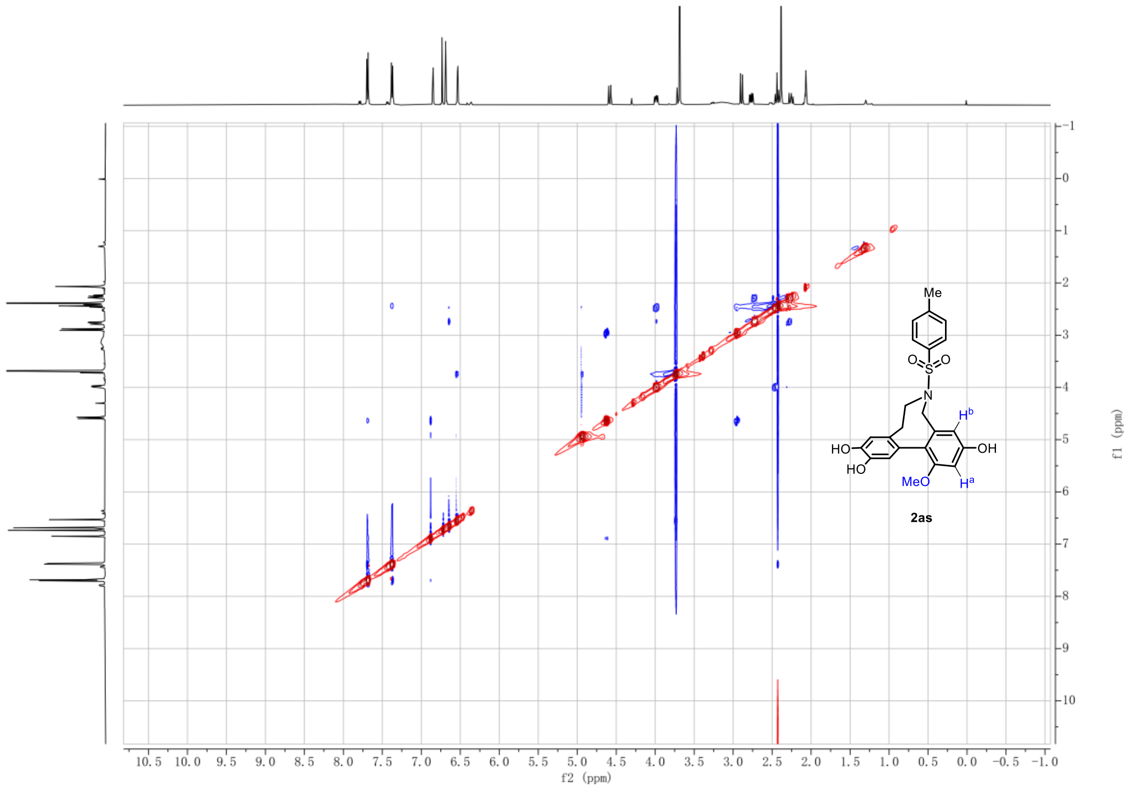
Me
O
S
O
N
H^b
HO
HO
OH
MeO
H^a
2as
f2 (ppm)
f1 (ppm)

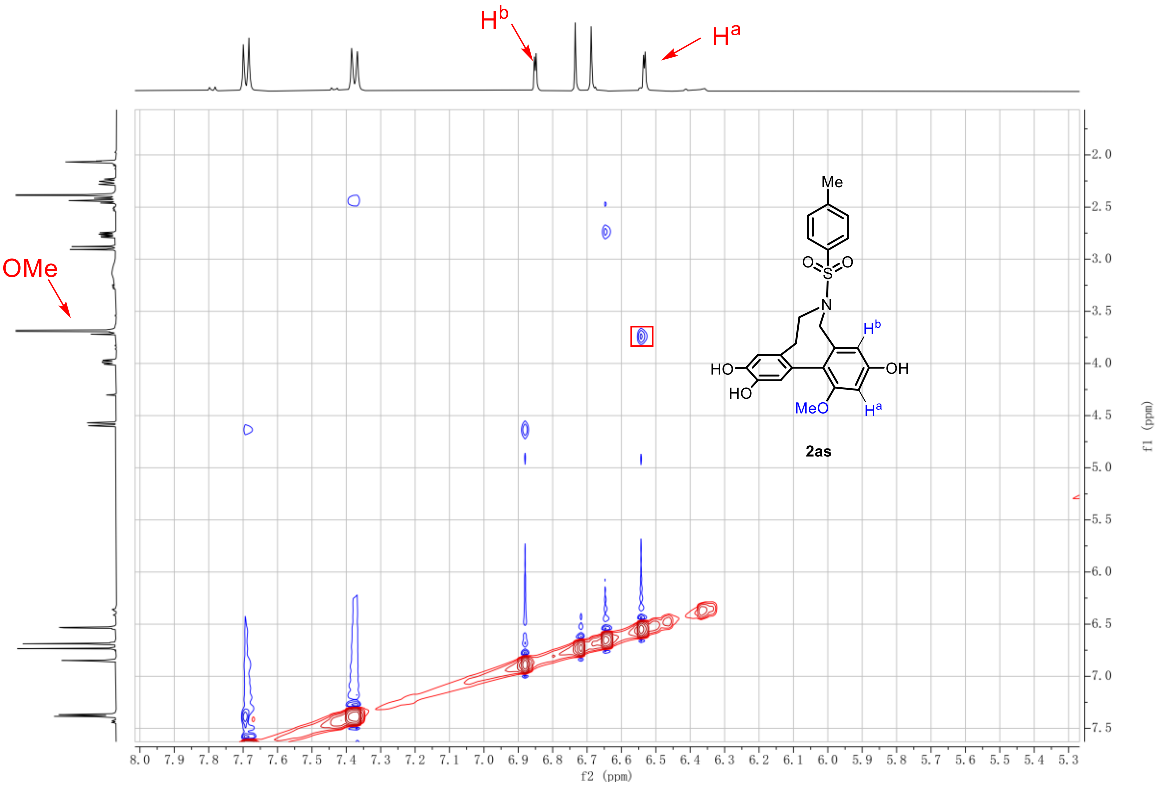

^{1}H NMR spectrum of compound 2at (500 MHz) in Acetone-d_6

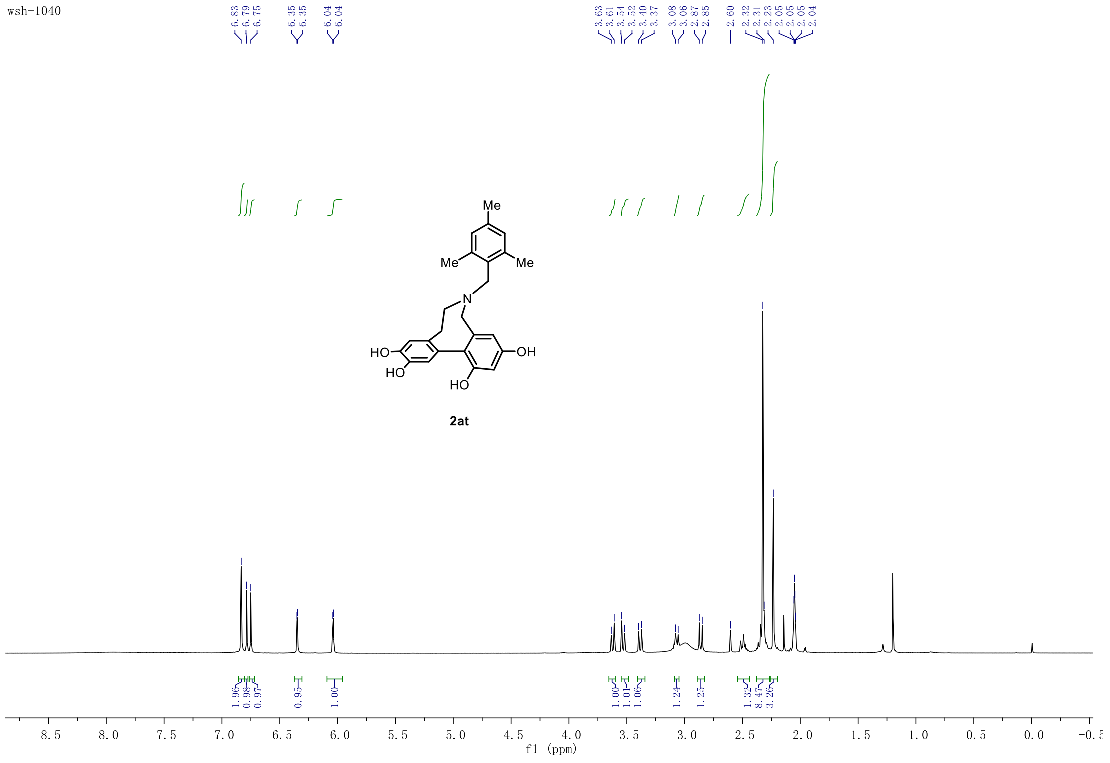

^{13}C NMR spectrum of compound 2at (125 MHz) in Acetone-d_6

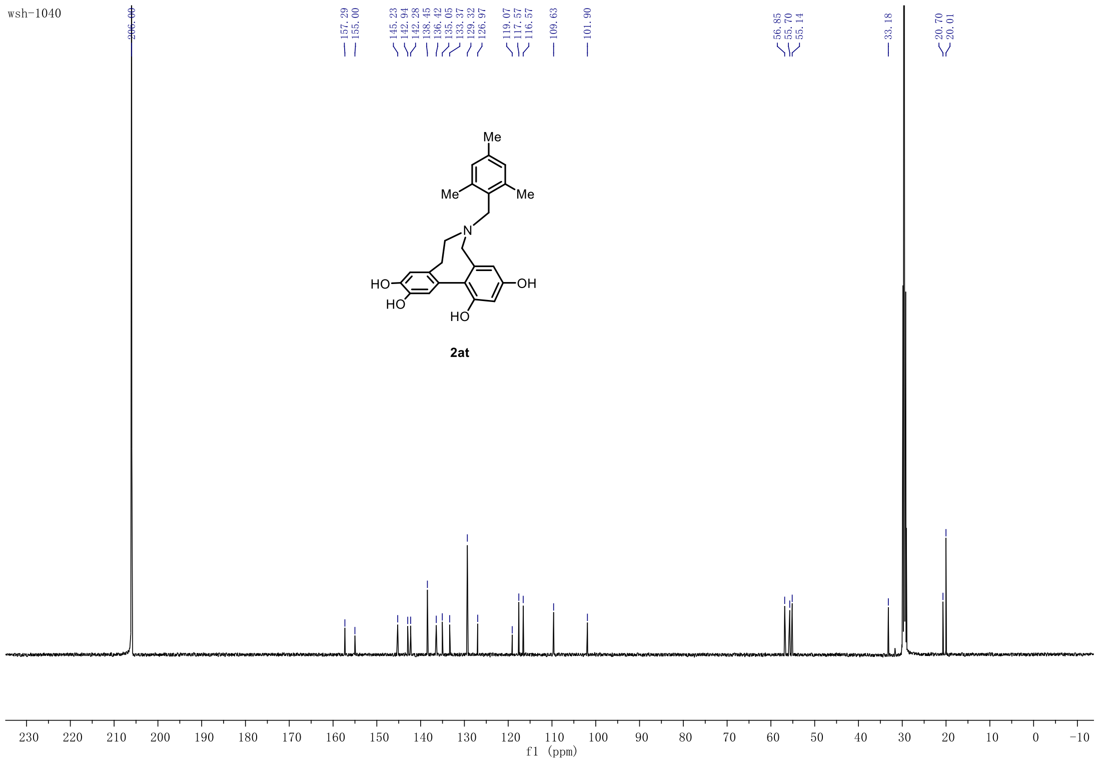

^{1}H NMR spectrum of compound 2az (400 MHz) in CD_3OD

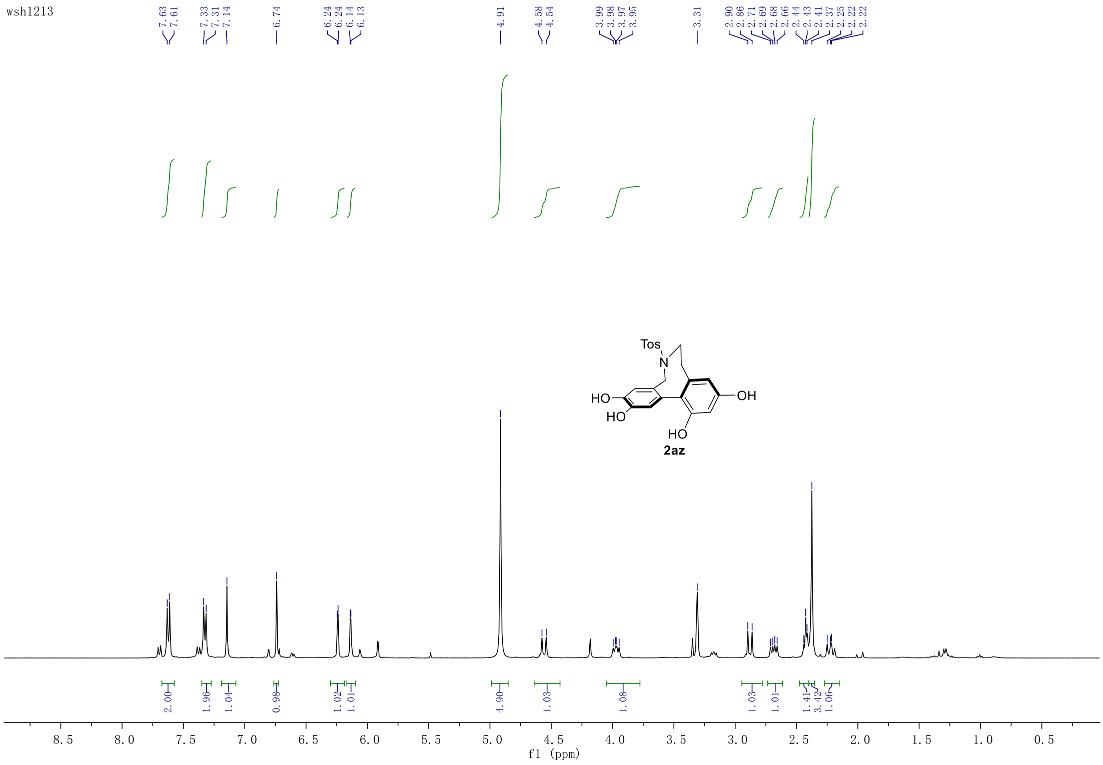

^{13}C NMR spectrum of compound 2az (100 MHz) in CD_3OD

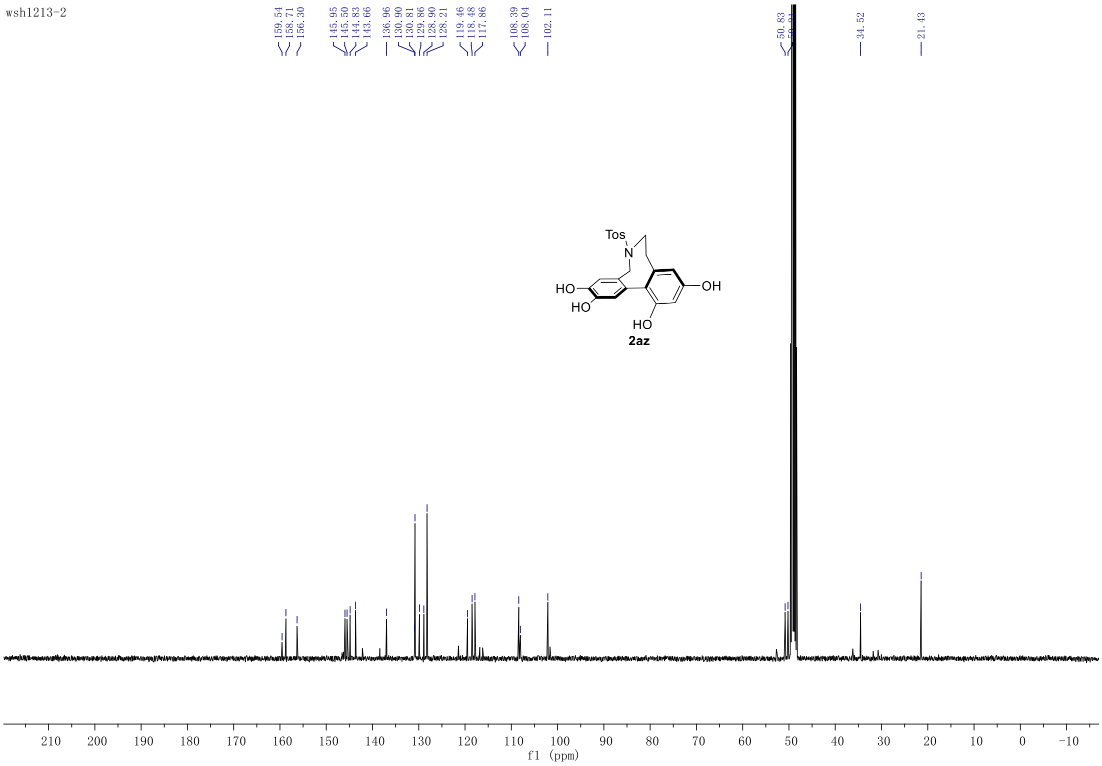

^{1}H NMR spectrum of compound 3a (400 MHz) in $CDCl_3$

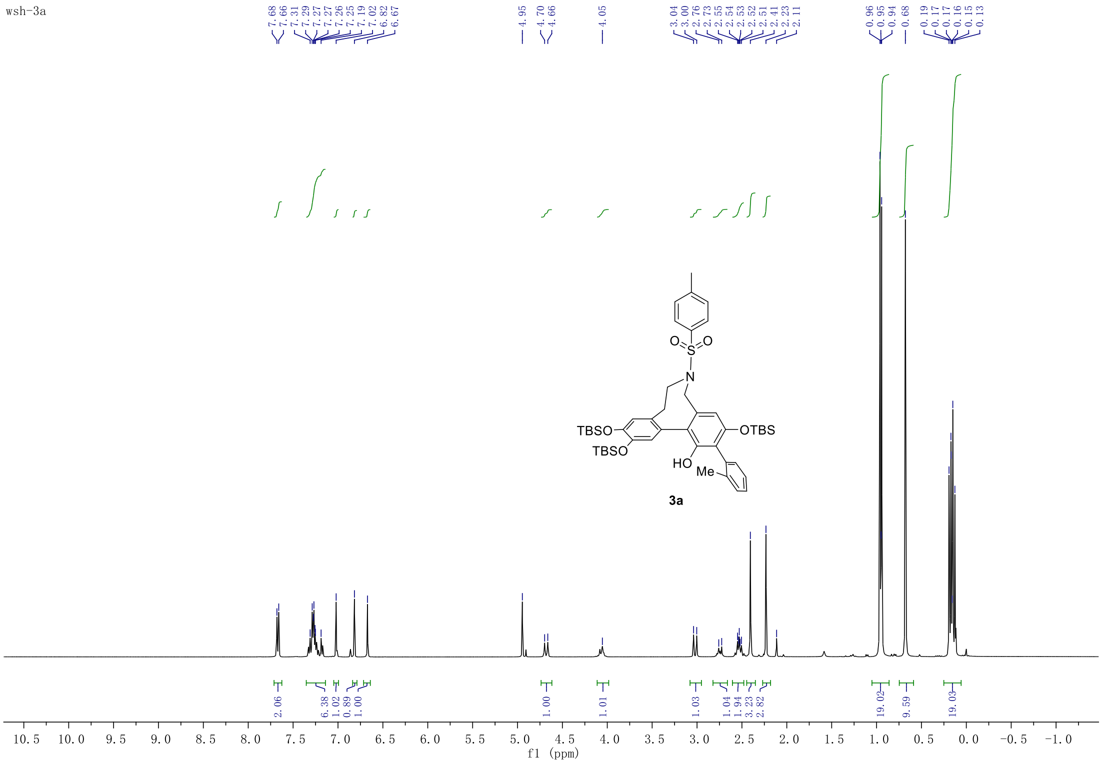

^{13}C NMR spectrum of compound 3a (100 MHz) in $CDCl_3$

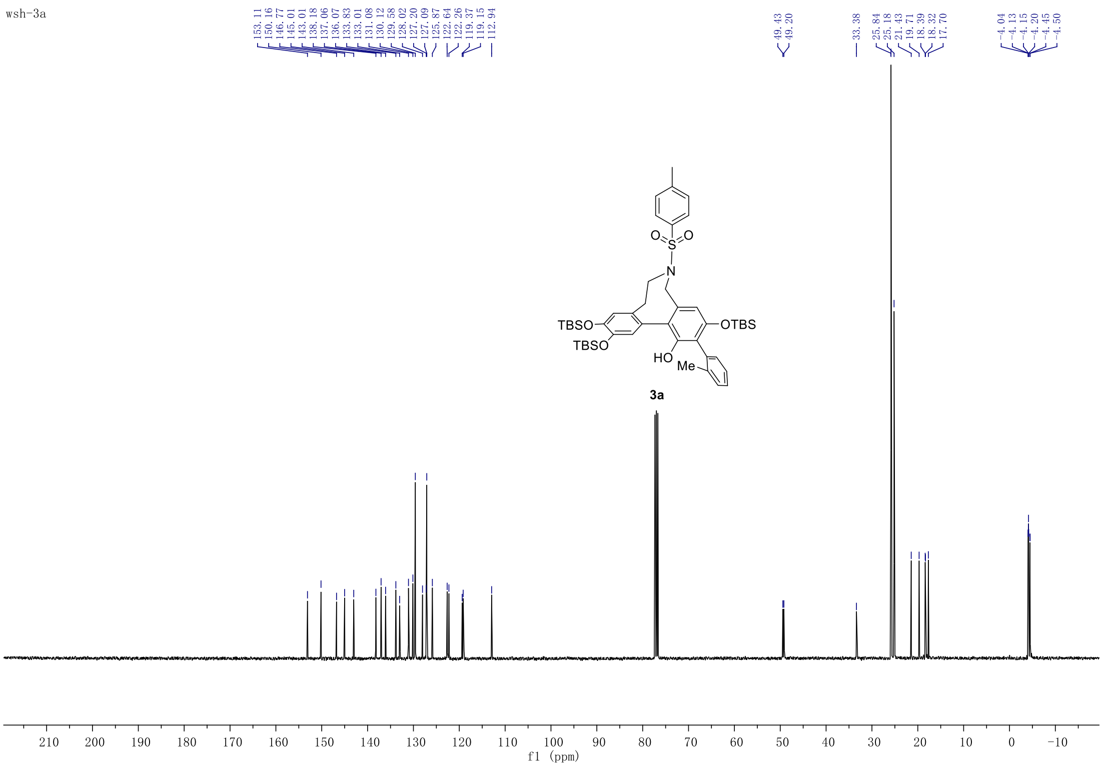

16. Supplementary References

(1) Lang, K.; Torker, S.; Wojtas, L.; Zhang, X. P. Asymmetric induction and enantiodivergence in catalytic radical C-H amination via enantiodifferentiative H-atom abstraction and stereoretentive radical substitution. *J. Am. Chem. Soc.*, **141**, 12388-12396 (2019).

(2) Barder, T. E.; Walker, S. D.; Martinelli, J. R.; Buchwald, S. L. Catalysts for Suzuki−Miyaura coupling processes: scope and studies of the effect of ligand structure. *J. Am. Chem. Soc.*, **127**, 4685-4696 (2005).

(3) Huang, Z.; Lumb, J. P. A Catalyst-controlled aerobic coupling of ortho-quinones and phenols applied to the synthesis of aryl ethers. *Angew. Chem. Int. Ed.*, **55**, 11543-11547 (2016).

(4) Zhang, M. Y.; Barrow, R. A. Accessing polyoxygenated dibenzofurans via the union of phenols and *o*-benzoquinones: rapid syntheses of metabolites isolated from ribes takare. *Org. Lett.*, **19**, 2302-2305 (2017).

(5) Escudero, J.; Mampuys, P.; Mensch, C.; Bheeter, C. B.; Vroemans, R.; Orru, R. V. A.; Harvey, J.; Maes, B. U. W. Synthesis of heterocycles via aerobic Ni-catalyzed imidoylation of

aromatic 1,2-bis-nucleophiles with isocyanides. *ACS. Catal.*, **12**, 6857-6873 (2022).

(6) Sheldrick, G. M., Crystal Structure Refinement with Shelxl. Acta Crystallogr., C: Struct. Chem., **71**, 3-8 (2015).

(7) Berard-Rees, Lasse Jenner and Marat Yusupov. Bulk-solvent correction in large macromolecular Structures. Acta. Cryst , **D61**.1299-1301 (2005).

(8) O. Dolomanov, L. Bourhis, R. Gildea, J. Howard, K. Puschmann, OLEX2: a complete structure solution, refinement and analysis program. J. Appl. Crystallogr., **42**, 339-341 (2009)

(9) Flack, H. D. & Bernardinelli, G. The use of X-ray crystallography to determine absolute configuration. Chirality., **20**, 681-690 (2008).
